# The shaping of cancer genomes with the regional impact of mutation processes

**DOI:** 10.1038/s12276-022-00808-x

**Published:** 2022-07-28

**Authors:** Soo-Youn Lee, Han Wang, Hae Jin Cho, Ruibin Xi, Tae-Min Kim

**Affiliations:** 1 Department of Medical Informatics, College of Medicine, The Catholic University of Korea, Seoul, Korea; 2 Cancer Research Institute, College of Medicine, The Catholic University of Korea, Seoul, Korea; 3 School of Mathematical Sciences and Center for Statistical Science, Peking University, Beijing, China

**Keywords:** Cancer genomics, Tumour biomarkers

## Abstract

Mutation signature analysis has been used to infer the contributions of various DNA mutagenic-repair events in individual cancer genomes. Here, we build a statistical framework using a multinomial distribution to assign individual mutations to their cognate mutation signatures. We applied it to 47 million somatic mutations in 1925 publicly available cancer genomes to obtain a mutation signature map at the resolution of individual somatic mutations. Based on mutation signature-level genetic-epigenetic correlative analyses, mutations with transcriptional and replicative strand asymmetries show different enrichment patterns across genomes, and “transcribed” chromatin states and gene boundaries are particularly vulnerable to transcription-coupled repair activities. While causative processes of cancer-driving mutations can be diverse, as shown for converging effects of multiple mutational processes on *TP53* mutations, the substantial fraction of recurrently mutated amino acids points to specific mutational processes, e.g., age-related C-to-T transition for *KRAS* p.G12 mutations. Our investigation of evolutionary trajectories with respect to mutation signatures further revealed that candidate pairs of early- vs. late-operative mutation processes in cancer genomes represent evolutionary dynamics of multiple mutational processes in the shaping of cancer genomes. We also observed that the local mutation clusters of kataegis often include mutations arising from multiple mutational processes, suggestive of a locally synchronous impact of multiple mutational processes on cancer genomes. Taken together, our examination of the genome-wide landscape of mutation signatures at the resolution of individual somatic mutations shows the spatially and temporally distinct mutagenesis-repair-replication histories of various mutational processes and their effects on shaping cancer genomes.

## Cancer: Modeling mutations to understand disease evolution

A statistical model that assigns non-hereditary DNA alterations known as somatic mutations to mutation “signatures” (groups of mutations arising from a specific biological process) on cancer genomes provides novel insights into disease evolution. Somatic mutations result from exposure to factors often linked to cancer development, such as tobacco or ultraviolet radiation. However, assigning a somatic mutation to a particular mutation “signature” remains challenging. The model created by Ruibin Xi (Peking University, China) and Tae-Min Kim (Catholic University of Korea, Seoul, South Korea) and co-workers grouped 47 million somatic mutations in 1925 cancer genomes into localized clusters before connecting them with mutation signatures. This strategy highlights the spatial and temporal patterns related to the origins of mutations, how the DNA strands are repaired and replicated, and how this influences the emerging cancer genome.

## Introduction

It has been proposed that a collective set of mutations in cancer genomes may be informative in evaluating the effects of variable mutagenic events and their associated DNA repair-replication processes^1–3^. Because exogenous and endogenous mutagenic events often leave characteristic sequence footprints, it is possible to infer which mutational processes have been operative in cancer genomes and to what extent using cancer genome sequencing data^4^. The pioneering work of the PanCancer-scaled mutation database has yielded ~30 mutation signatures of unique trinucleotide sequences that correspond to distinct mutational processes and tumor lineages^5^. The discovery of mutation signatures and their causal associations has enabled measuring the relative contributions of multiple mutational processes in cancer genomes by assuming that the relative abundance of mutation signatures corresponds with the contribution of their cognate mutation events.

Although mutation signature analyses have used genome-wide aggregates of mutations to quantify the type and level of exposure to mutational processes in individual cancer genomes, investigations of mutational causalities for individual somatic mutations have been largely hampered for several reasons. First, mutation signatures as probability distributions over trinucleotide contexts are difficult to present as single-nucleotide changes. Moreover, mutation signatures are not exclusive and often share trinucleotide sequences. For example, the C-to-T transition of CpG dinucleotides (presented as C > T substitutions at NpCpG trinucleotides) is a nucleotide change representative of age-related mutation signature 1 (hereafter, single base substitution 1 or SBS1, COSMIC mutation signatures, Ver. 2), but it accounts for only 44.7% of the trinucleotide contexts in SBS1; it also accounts for 10.6 to 35.4% of the nucleotide changes in other mutation signatures (SBS6, -7, -10, -14, -15, and -20, COSMIC mutation signatures, Ver. 2). Thus, the nonexclusivity of mutation signatures (in terms of their nucleotide changes) limits the general usefulness of single-nucleotide change-based inferences about mutational processes. Second, maximum likelihood has been used to assign individual somatic mutations to mutation signatures^6^. However, this method depends on sample-level mutation signatures and ignores regional variations in somatic mutations across cancer genomes. Previous genomic bin-based correlative analyses have revealed that euchromatic regions that replicate early harbor smaller numbers of somatic or germline mutations than heterochromatic regions that replicate late^7–10^. These mutational features might be related to the accessibility of DNA repair machinery, which could further explain the relative depletion of mutations in transcribed strands^5^, as well as the relatively uniform distribution of mutations in microsatellite instability-high genomes with DNA mismatch repair deficiency^11^. Hence, the regional activity of DNA damage, repair, and replicative processes is not uniform across cancer genomes, which should be taken into account when making regional inferences about the causality of somatic mutations, such as a mutation signature analysis of individual somatic mutations.

In this study, we used whole-genome mutation profiles for 1925 cancer genomes available from International Cancer Genome Consortium (ICGC) to systematically analyze the mutation signatures of individual somatic mutations in cancer genomes^12^. Somatic mutations in individual cancer genomes were transformed into coordinate-sorted, binary matrices of trinucleotide-context mutation calls. The matrices were segmented by minimizing the Bayesian information criterion (BIC) of a multinomial model, and the resulting mutation segments were further clustered by BIC to obtain “mutation clusters” as sets of nonadjacent mutation segments with highly similar trinucleotide mutation contexts. Individual somatic mutations were assigned to the cognate mutation signatures of their mutation clusters by maximizing the posterior probability. The mutation signature profiles of individual somatic mutations were further investigated to determine their relationships to various genomic and epigenetic features and reveal insight into how mutational processes have shaped cancer genomes.

## Materials and methods

### Study cohort

The PCAWG/ICGC cohort was used as a public resource for studying whole-genome sequencing-based somatic mutations. We used SNVs downloaded from the ICGC data portal (dcc.icgc.org) as somatic mutations. To minimize bias in the distribution of somatic mutations, we selected among those available 1925 cancer genomes with no fewer than 3000 mutations^11^. Lineage-specific analyses were performed for 24 tumor types with more than ten tumors.

### Genome segmentation and clustering

For a given cancer genome, mutations along a chromosome were first converted into a binary matrix in the context of 96 trinucleotides (i.e., the substituted base and its immediate 5’ and 3’ vicinity). The matrix was sorted by the genome coordinates of the mutations. To obtain clustered genomic segments with highly similar trinucleotide contexts, we applied a BIC-based local- and distant-merging strategy. First, to identify local regions with homogeneous mutation contexts, we binned the chromosomes into equal-sized bins, with each bin containing at least 30 mutations. Then, we iteratively merge neighboring bins with similar mutation signatures based on the BIC. More specifically, if we suppose that a cancer sample has *N* mutations in a chromosome and let *m*_*j*_ be the *j*th mutation *(j* = 1,...,*N)*, there are 96 possible mutation types, *MT*_1_,..., *MT*_96_, according to the trinucleotide context. If we let *p*_*j,i*_ = *P*(*m*_*j*_ = *MT*_*i*_) be the probability that mutation *m*_*j*_ is of type *MT*_*i*_ (*i* = 1, …, 96), then *m*_*j*_ can be viewed as a random variable from a multinomial distribution *Mult*(1;***p***_*j*_*)*, where ***p***_*j*_ = (p_*j*,1_),*p*_*j*,2_, ..., *p*_*j*,96_). If the two mutations *m*_*j*_ and *m*_*k*_ are from the same mutation signature, then ***p***_*j*_ = ***p***_*k*_. Now suppose that at a certain merging step, there are *T* remaining segments *S* = {*S*_1_,*S*_2_ …, *S*_*T*_}, and assume that there are *n*_*t*_ mutations in segment *S*_*t*_ (*t* = 1,...,*T*); thus, $N = \mathop {\sum}\nolimits_{t = 1}^T {n_t}$. If we denote *m*_*t,j*_ ($j = 1,...,n_t$) as the mutations in segment *S*_*t*_ and their corresponding probabilities as ***p***_*t,j*_, in the merging process, the mutation signatures of the same segments will be homogeneous, and the probabilities ***p***_*t,j*_ in segment *S*_*t*_ should all be equal to each other (${{{\boldsymbol{p}}}}_{t,j} = {{{\boldsymbol{p}}}}_{t,k}$ for all $j,k = 1,...,n_t$). If we denote the common probability vector as ${{{\boldsymbol{q}}}}_t = (q_{t,1}, \cdots ,q_{t,96}) = {{{\boldsymbol{p}}}}_{t,j}$, given the segment *S*_*t*_, the maximum likelihood estimation (MLE) of *q*_*t,i*_ is$\hat q_{t,i} = n_t^{ - 1}\mathop {\sum}\nolimits_{j = 1}^{n_t} {I(m_{t,j} = MT_i)(i = 1,...96)}$, where *I*(⋅) is an indicator function. If we define a modified BIC for segmentation *S* as:

$${\rm{BIC}}({{{\mathcal{S}}}}) = - 2{{{\mathrm{log}}}}(L_{{{\mathcal{S}}}}) + \lambda _1T{{{\mathrm{log}}}}(N)$$

*L*_*S*_ is the likelihood of segmentation *S* and *λ*_1_ is a tuning parameter, which we take as 5. Concretely, given the segmentation *S*, the overall BIC of this segmentation is:

$${\rm{BIC}}({{{\mathcal{S}}}}) = - 2\mathop {\sum}\nolimits_{t = 1}^T {\mathop {\sum}\nolimits_{j = 1}^{n_t} {\mathop {\sum}\nolimits_{i = 1}^{96} {I(m_{t,j} = MT_i){{{\mathrm{log}}}}}}} \left( {\hat q_{t,i}} \right) + \lambda _1T{{{\mathrm{log}}}}(N)$$

The overall merging process minimizes the BIC. Given the current segmentation *S* = {*S*_1_, *S*_2_ …, *S*_T_}, we calculated the BIC change from merging each neighboring pair and merged the pair that produced the largest BIC reduction. More specifically, suppose that *S*_*t*_ and *S*_*t* + 1_ are to be merged, so the merged segment is *S*_*t,t* + 1_. If *S*_*t*_ and *S*_*t* + 1_ are merged, we would have a new segmentation, ${{{\mathcal{S}}}}_t = \{ S_1, \cdots ,S_{t - 1},S_{t,t + 1},S_{t + 2}, \cdots ,S_T\}$, and its BIC would be BIC(*S*_*t*_). We defined the similarity score between *S*_*t*_ and *S*_*t* = 1_ as ${\rm{Sim}}\left( {S_t,S_{t + 1}} \right) = {\rm{BIC}}\left( {{{{\mathcal{S}}}}_t} \right) - {\rm{BIC}}({{{\mathcal{S}}}})$. If ${\rm{Sim}}\left( {S_t,S_{t + 1}} \right) < 0$, merging *S*_*t*_ and *S*_*t*+1_ will make the overall BIC smaller. We chose *t*_0_ such that ${\rm{Sim}}\left( {S_{t_0},S_{t_0 + 1}} \right) = {\rm{min}} \{ {\rm{Sim}}\left( {S_t,S_{t + 1}} \right),t = 1, \cdots ,T - 1\}$. If ${\rm{Sim}}\left( {S_{t_0},S_{t_0 + 1}} \right) > 0$, we stopped the merging process because merging all of the neighboring pairs would increase the BIC. If ${\rm{Sim}}\left( {S_{t_0},S_{t_0 + 1}} \right) \le 0$, we merged the neighboring pair *S*_*t*0_ and *S*_*t*0+1_ and repeated the merging process until no neighboring segments could be merged. We denoted the resulting segments with homogeneous mutation contexts as “mutation segments”.

After the local merging of neighboring segments is complete, the mutation signatures of the remaining neighboring segment pairs will be heterogeneous. However, distant, non-neighboring segments might still share similar mutation signatures due to the nonuniform activity of mutational processes in cancer genomes. We therefore further performed clustering analysis of the segments such that segments in the same cluster have similar mutation signatures. If we assume *T* segments $S_1,S_2,...,S_T$ after segmentation, we again denoted *m*_*i,j*_ ($j = 1,...,n_t$*)* as the mutations in segment *S*_*t*_ and their corresponding common probabilities as ***q***_*t*_. At the initial step of the clustering procedure, each segment is itself a cluster. We iteratively merged the clusters again based on the BIC. The merging process was similar to the neighboring segment merging process except that the merged segments need not be neighbors. Next, suppose that at a certain step, we have a clustering *C* with *K* clusters, $C_1, \cdots ,C_K$, and cluster *C*_*k*_ contains *s*_*k*_ segments. The segments *S*_*t*_ in *C*_*k*_ have a common probability vector, ${{{\boldsymbol{q}}}}_k^C = (q_{k,1}^C, \cdots ,q_{k,96}^C)$. The MLE of $q_{k,i}^C$ is $\hat q_{k,i}^C = N_k^{ - 1}\mathop {\sum}\nolimits_{S_t \in C_k} {\mathop {\sum}\nolimits_{j = 1}^{n_t} {I(m_{t,j} = MT_i)} }$, where *N*_*k*_ is the number of mutations in cluster *C*_*k*_. The overall BIC of this clustering is:

$${\rm{BIC}}\left( {{{\mathcal{C}}}} \right) = - 2\mathop {\sum }\limits_{k = 1}^K \mathop {\sum }\limits_{S_t \in C_k} \mathop {\sum }\limits_{j = 1}^{n_t} \mathop {\sum }\limits_{i = 1}^{96} I\left( {m_{t,j} = MT_i} \right){{{\mathrm{log}}}}\left( {\hat q_{k,i}^C} \right) + \lambda _2K{\rm{log}}\left( N \right),$$

where *λ*_2_ is also a tuning parameter, and we also take *λ*_2_ as 5. Similar to the neighboring segment merging process, we calculated the BIC changes by merging all pairs of clusters. If merging pairs of clusters did not decrease the BIC, we stopped the merging process and returned to clustering. Otherwise, we merged the cluster pair that produced the largest BIC reduction and repeated the merging process until no cluster pairs could be merged. We denoted the sets of non-neighboring mutation segments as “mutation clusters” and used them as units for mutation signature analysis.

### Mutation signatures and assignment to individual mutations

We assigned an individual somatic mutation to its cognate mutation signature based on its posterior probability. Given a mutation *i*, we let *MT*_*i*_ be its trinucleotide context. Given that mutation *i* belongs to the genomic region *gf*_*r*_, we calculated its posterior probability of being generated from the *v*th mutation signature *MS*_*v*_. We denoted this posterior probability as $P(MT_i \in MS_v|MT_i \in gf_r)$, where $MT_i \in MS_v$ is understood to indicate that mutation *i* is generated by the *v*th mutation signature *MS*_*v*_ and $MT_i \in gf_r$ indicates that mutation *i* derives from genomic region *gf*_*r*_. We denoted $MS_v \in gf_r$ if the mutation signature *MS*_*v*_ is in the genomic region *gf*_*r*_ and $MS_v \,\notin\, gf_r$ if not. In this paper, *gf*_*r*_ refers to the genomic cluster obtained above. We calculated $P(MT_i \in MS_v|MT_i \in gf_r)$ for each *v* = 1, ..., 30:

$$\begin{array}{l}P\left( {MT_i \,\in\, MS_v{{{\mathrm{|}}}}MT_i \,\in\, gf_r} \right) = \frac{{P\left( {MT_i\, \in\, MS_v,MT_i \,\in\, gf_r} \right)}}{{P\left( {MT_i \,\in\, gf_r} \right)}}\\ = \frac{{P\left( {MT_i \,\in\, MS_v,MT_i \,\in\, gf_r,MS_v \,\in\, gf_r} \right) + P\left( {MT_i \,\in\, MS_v,MT_i \,\in\, gf_r,MS_v \,\notin\, gf_r} \right)}}{{P\left( {MT_i \,\in\, gf_r} \right)}}\\ = \frac{{P\left( {MT_i \,\in\, MS_v,MT_i \,\in\, gf_r,MS_v \,\in\, gf_r} \right)}}{{\mathop {\sum }\nolimits_{v = 1}^{30} P\left( {MT_i \,\in\, MS_v,MS_v \,\in\, gf_r} \right)}} = \frac{{P\left( {MT_i \,\in \, MS_v,MS_v \,\in\, gf_r} \right)}}{{\mathop {\sum }\nolimits_{v = 1}^{30} P\left( {MT_i \,\in\, MS_v,MS_v \,\in\, gf_r} \right)}}\\ = \frac{{P\left( {MT_i \,\in\, MS_v{{{\mathrm{|}}}}MS_v \,\in\, gf_r} \right)P\left( {MS_v \,\in\, gf_r} \right)}}{{\mathop {\sum }\nolimits_{v = 1}^{30} P\left( {MT_i \,\in\, MS_v{{{\mathrm{|}}}}MS_v \,\in\, gf_r} \right)P\left( {MS_v \,\in\, gf_r} \right)}} = \frac{{h_{vi}d_{rv}}}{{\mathop {\sum }\nolimits_{v = 1}^{30} h_{vi}d_{rv}}},\end{array}$$

where $d_{rv} = P\left( {MS_v \in gf_r} \right)$ and $h_{vi} = P(MT_i \in MS_v|MS_v \in gf_r)$. *d*_*rv*_ is the proportion of mutation signature *MS*_*v*_ in region *gf*_*r*_ and can be calculated by the R package “deconstructSigs”. *h*_*vi*_ ($i = 1, \cdots 30$) is the probability pattern for mutation signature *MS*_*v*_, which can be downloaded from COSMIC. Here, the numerator *h*_*vi*_*d*_*rv*_ is proportional to the expected number of mutations *i* contributed by mutation signature *MS*_*v*_ in genomic region *gf*_*r*_, and the denominator is proportional to the expected number of mutations *i* contributed by all 30 mutation signatures in *gf*_*r*_, which can be regarded as a normalization term such that summation of the posterior probabilities across all 30 mutation signatures is equal to 1. If $\hat v = \mathop {{{{{\mathrm{argmax}}}}}}\limits_{{{\mathrm{v}}}} P(MT_i \in MS_v|MT_i \in gf_r)$, then we assigned *MT*_*i*_ to mutation signature $MS_{\hat v}$.

### Concordance of mutation segments in a cluster

To evaluate whether the mutation segments grouped as mutation clusters are more concordant than random at epigenetic and evolutionary scales, we used the two features of replication timing and cancer cell fraction (CCF), and we evaluated the association of mutation clusters with replication timing and CCF by permutation. For replication time, we downloaded the replication timing profile (GM12878 Repli-Seq data of ENCODE) from UCSC Genome Browser (https://genome.ucsc.edu) and calculated the Shannon entropy with respect to the Repli-seq signals in each mutation cluster of an individual genome. To calculate the Shannon entropy of Repli-seq signals in a genomic region, we classified the signals into three levels (low, middle, and high) based on signal intensity. Given a genomic region *gf*_*r*_, we denoted *m*_*r,j*_ *(*$j = 1,...,n_{{{\mathrm{r}}}}$*)* as the number of mutations in *gf*_*r*_, where *n*_*r*_ is the number of mutations in *gf*_*r*_. We further denoted *L*_*r,j*_ as the level of the Repli-seq signal for mutation *m*_*r,j*_ and let $P(L_{r,j} = l) = O_{rl}$ be the probability that mutation *m*_*r,j*_ is of Repli-seq signal level *l*. Here, we assumed that *l* = 1, 2, and 3 represent low, middle, and high signal levels, respectively. Then, *L*_*r,j*_ is a random variable from multinomial distribution $Mult(1;{{{\boldsymbol{O}}}}_{{{\mathrm{r}}}})$, where ${{{\boldsymbol{O}}}}_{{{\mathrm{r}}}} = (O_{r1},O_{r2},O_{r3})$. The MLE of *O*_*rl*_ is $\hat O_{rl} = n_{{{\mathrm{r}}}}^{ - 1}\mathop {\sum}\nolimits_{j = 1}^{n_{{{\mathrm{r}}}}} {I({{{\mathrm{L}}}}_{r,j} = l)(l = 1,2,3)}$, and the Shannon entropy of Repli-seq signals in genomic region *gf*_*r*_ can be calculated as follows:

1 $$E_r = - \mathop {\sum}\nolimits_{l = 1}^3 {\hat O_{rl}\,{{{\mathrm{log}}}}(\hat O_{rl})}$$

For mutation cluster *C*_*k*_ in a cancer genome, suppose that the Shannon entropy of the Repli-seq signals calculated by (1) is denoted as *E*_*k*_. To evaluate the significance of the concordance of Repli-seq signals in this cluster, we randomly chose the same number of mutations along the genome as those in that cluster and calculated the Shannon entropy of the Repli-seq signals in that new set. We then repeated that process 10,000 times to construct the distribution under the null hypothesis that there is no concordance among the Repli-seq signals in that cluster. If we suppose that the Shannon entropy returned by this procedure is ${{{\mathrm{E}}}}_{{{{\mathrm{k}}}},1},...,{{{\mathrm{E}}}}_{{{{\mathrm{k}}}},\,10,000}$ and define $H_k = \{ i|E_{k,i} \le E_k,i = 1,...,10,\!000\}$, then the *p* value is calculated as $p_k = |H_k|/10,\!000$, where $|H_k|$ is the cardinality of the set *H*_*k*_. Evolutionary concordance was similarly calculated using the CCF of the PCAWG study from the ICGC data portal (dcc.icgc.org).

### Bin-based genomic and epigenomic correlation analyses

For genetic correlative analyses, we obtained the GC content, recombination rate, conservation level, and repeat elements of major categories (Alu, MIR, L1, L2, LTR, and DNA transposons) from the UCSC Genome Browser with table functions (hg19, http://genome.ucsc.edu). For the genomic bins, we averaged the genetic features into bin-level densities and subjected them to correlative analyses with the mutation density of individual mutation signatures. Single-nucleotide polymorphism (SNPs) were obtained from dnSNP129 and classified as common or rare according to population frequency (>1% and <1% in 1000 Genomes Project, respectively). For bin-based epigenetic correlation, we obtained ChIP-seq data representing various histone methylation and acetylation marks from public resources^13,14^ and calculated the bin-based sequencing read depth for correlative analysis. ChromHMM annotations for lymphoblastoid cell lines (GM12878) mapped to the hg19 genome were also obtained from UCSC Genome Browser. The mutation number was counted with respect to mutation signatures and twelve chromHMM chromatin states. For nucleosome positioning, we obtained MNase-seq signals representing the nucleosome positioning of the lymphoblastoid and K562 cell lines from the ENCODE project, as available in Gene Expression Omnibus (GSM920557 and GSM920558, respectively; https://www.ncbi.nlm.nih.gov/geo/). MNase-seq signals in the indexed binary (bigwig) format were downloaded, and nucleosome density signals were aggregated into bp-resolution mutation-centric 2 kb windows (1 kb up- and 1 kb-downstream of individual mutations), as previously described^6^. To estimate the mutation-centric local density of histone marks, we obtained high-resolution ChIP-seq data for 11 histone marks from the Roadmap Epigenomics dataset (http://www.roadmapepigenomics.org/data/). The datasets consolidated as continuous ChIP-seq/DNase counts in bigwig format were used to measure the local density of histone marks around the mutations in 2-kb windows.

### Mutational transcriptional and replicative strand asymmetry

We used AsymTools (Ver. 1.0.3; www.broadinstitute.org/cancer/cga/AsymTools) to estimate the level of mutational strand asymmetry^15^. Mutations were classified into different mutation signatures. As the tool first determines the direction of RNA transcription according to the RefSeq definitions, the mutations in RefSeq were further classified into tx (+) and tx (−) with respect to the direction of transcription. For the DNA replication direction, replication-timing profiles were used, and genomic regions were discriminated into “left replicating” and “right replicating”. According to the direction of DNA replication, mutations were also categorized and measured for strand asymmetry levels. Maximal transcriptional and replicative strand asymmetries were estimated according to the mutation signature and used to classify mutation signatures.

### Coding genes

For coding genes, we obtained RefSeq annotation with transcription start and end sites from UCSC Genome Browser and obtained 426,720 exonic mutations in coding regions (0.89% of total mutations). The mutation density with respect to mutation signatures was compared across exonic and nonexonic regions (intronic and intergenic regions, respectively) and for the functional categories of mutations annotated by ANNOVAR^16^. Exonic mutation densities in transcribed gene bodies were also calculated for 14 bins of individual genes, where 10 equally divided “gene-body” bins were defined between the transcription start and end sites of a given gene. Two upstream (5’ of the transcription start sites) and two downstream (3’ of the transcription end sites) bins were further identified as gene boundaries and combined with the 10 gene-body bins to estimate the mutation density with respect to genes. Frequently mutated genes among known cancer-related genes (COSMIC Cancer Census Genes)^17^ were identified. Mutation hotspots, namely, frequently mutated amino acid residues, were also obtained from the literature^18^. Protein domain information was obtained from UniProt and InterPro using biomaRt (Ver. 2.46.3), and we used the trackViewer R package (Ver. 1.26.2) for lollipop visualization. We used Fisher’s exact test to estimate the level of significance for mutation signatures per hotspot. The numbers of mutations in each hotpot and mutation signature were evaluated for enrichment against the number of corresponding mutations in genes harboring the hotspot. To identify functionally relevant genes per mutation signature, we used two methods: MutSigCV^19^ and dNdSCV^20^. MutSigCV (https://software.broadinstitute.org/cancer/cga/mutsig) identifies genes with significantly recurrent mutations, taking into account several covariates for epigenetic features (HiC and replication time) and gene expression levels. dNdSCV (https://github.com/im3sanger/dndscv) calculates the ratio of dNdS, which represents the level of evolutionary selection of mutations while taking into account trinucleotide contexts and other covariates. For both methods, we deconvoluted the mutations with respect to mutation signatures and used tools to identify potential cancer-driving mutations per mutation signature.

### Evolutionary trajectories of mutational processes

We used CCF to measure the evolutionary concordance of mutation segments belonging to the same mutation clusters. The CCF values of individual mutations were obtained from the ICGC data portal, as were mutation data. We also performed CCF-based evolutionary concordance analysis between mutation signatures. The Kolmogorov–Smirnov test was employed to estimate significance levels under the null hypothesis that the CCF values of mutations belonging to two mutation signatures would have the same distribution.

### Kataegis

For Kataegis, we used the R package maftools in R (Ver. 3.12) to identify kataegis, as defined as no fewer than six consecutive mutations separated by fewer than 1000 bp^21^. Overall, 7482 kataegic events were observed for nonhypermutated cancer genomes (those with <30,000 mutations). For individual kataegic events, member mutations were examined for six mutation spectra and mutation signatures. Significant enrichment of a mutation signature in a kataegic event was examined by Fisher’s exact test, given the number of mutations for each specific mutation signature against the total number of mutations in a kataegic event and the case.

## Results

### Mutation signature profiles at the resolution of individual somatic mutations

**Fig. 1:**
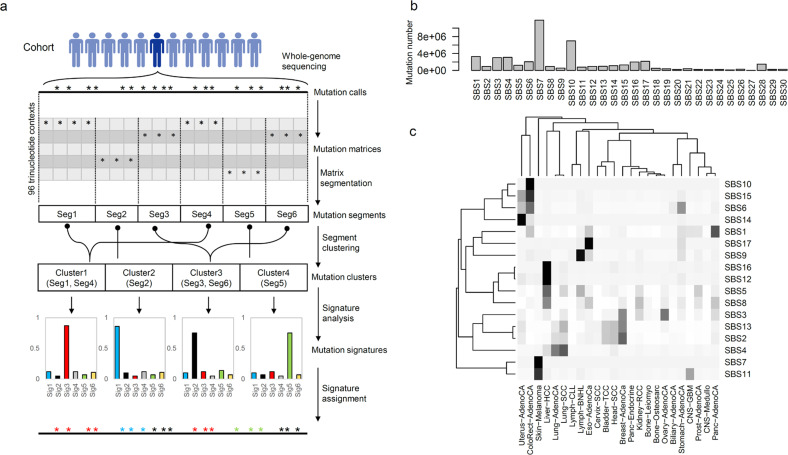
Schematics of mutation signature analysis for individual somatic mutations. **a** Schematic for mutation segmentation and clustering, mutation signature analysis, and assignment of mutation signatures to individual somatic mutations. **b** The abundance of mutation signatures for the whole set of somatic mutations is shown. **c** The mutation signature abundance is shown with respect to tumor lineage (24 tumor types with >10 cases).

A total of 47,838,326 single-nucleotide variants (SNVs) were obtained from 1925 ICGC/PanCancer Analysis of Whole Genomes (PCAWG) cancer genomes harboring no fewer than 3000 SNVs. The tumor types of the cohort are summarized in Supplementary Table 1. For each genome, a binary matrix of SNVs (sorted in order of their genomic coordinates) was constructed with respect to 96 trinucleotide contexts (i.e., the substituted base and two additional bases in the immediate 5’ and 3’ vicinities). These mutation matrices were then segmented and further clustered using BIC-based local and distant-merging algorithms, resulting in “mutation segments” and “mutation clusters”, respectively. The mutation clusters, as sets of non-neighboring mutation segments with similar trinucleotide contexts for somatic mutations, were then subjected to mutation signature analysis, and their somatic mutations were assigned to their cognate mutation signatures using a maximum posterior approach. Figure 1a presents schematics of the overall processes used to assign mutation signatures to individual somatic mutations.

The genome-wide frequencies of the 30 mutation signatures (SBS1 to SBS30 in COSMIC Ver. 2) for the entire cohort are shown in Fig. 1b. SBS7 and SBS10 were the most frequent signatures, mostly found for skin melanomas and *POLE*-mutated hypermutated genomes, respectively. The frequencies of mutation signatures in our database, as well as their frequently associated tumor types and known etiologies, are listed in Supplementary Table 2. We mainly used 17 mutation signatures (SBS1 to SBS17, 90.6% of initial mutation sets) for downstream analyses because of their abundance and relatively well-established etiologies. To further delineate the tumor-type-specific preponderance of mutation signatures, the abundance of mutation signatures against the tumor lineages was evaluated (24 tumor types with >10 cases, Fig. 1c and Supplementary Fig. 1). Known associations between mutation signatures and tumor lineages were identified. For example, DNA mismatch repair deficiency is represented by SBS6 and SBS15 (in colorectal and uterine cancers), *POLE*-deficiency-associated SBS10 (in colorectal cancers), ultraviolet (UV)-related SBS7 (in skin melanomas), tobacco-related SBS4 (in lung cancers), BRCA-deficient SBS3 (in breast and ovary cancers), SBS17 (in esophageal cancers), and SBS9 (in hematologic cancers).

We also found that clustering-based mutation signature assignments were more sensitive in identifying lineage-specific mutation signatures than were those based on individual cancer genomes or mutation segments (Supplementary Fig. 2). For example, lineage-specific mutations belonging to SBS4, SBS7, and SBS16 were more frequently recovered in their respective associated cancer types of lung cancers, skin melanomas, and hepatocellular carcinomas by using mutation signature assignments based on mutation clusters (“cluster-based” in Supplementary Fig. 2) than they were by using mutation segments or sample-level mutation signatures (“segment-” and “sample-based”, respectively; Supplementary Fig. 2). We observed that mutation segments belonging to the same mutation clusters were more concordant than randomly assigned mutation segments in terms of the genetic and epigenetic features represented by Repli-seq and CCF, respectively (Supplementary Fig. 3). The level of concordance was estimated as of nominal significance to reflect the Repli-seq and CCF scores of segments belonging to clusters biased due to the distribution of randomly clustered mutation segments. If there is no concordance, the −log_10_ *p* values should follow an exponential distribution; however, we observed that for all cancer types, the distributions of −log_10_ *p* values deviated vastly from the exponential distribution (Supplementary Fig. 3), suggesting that mutation clusters represent sets of (epi)genetically concordant, nonadjacent mutation segments. These results indicate that mutation clusters may serve as a better proxy than genome or mutation segments when assigning mutation signatures to somatic mutations.

### Genomic and epigenetic correlation of mutation signatures

**Fig. 2:**
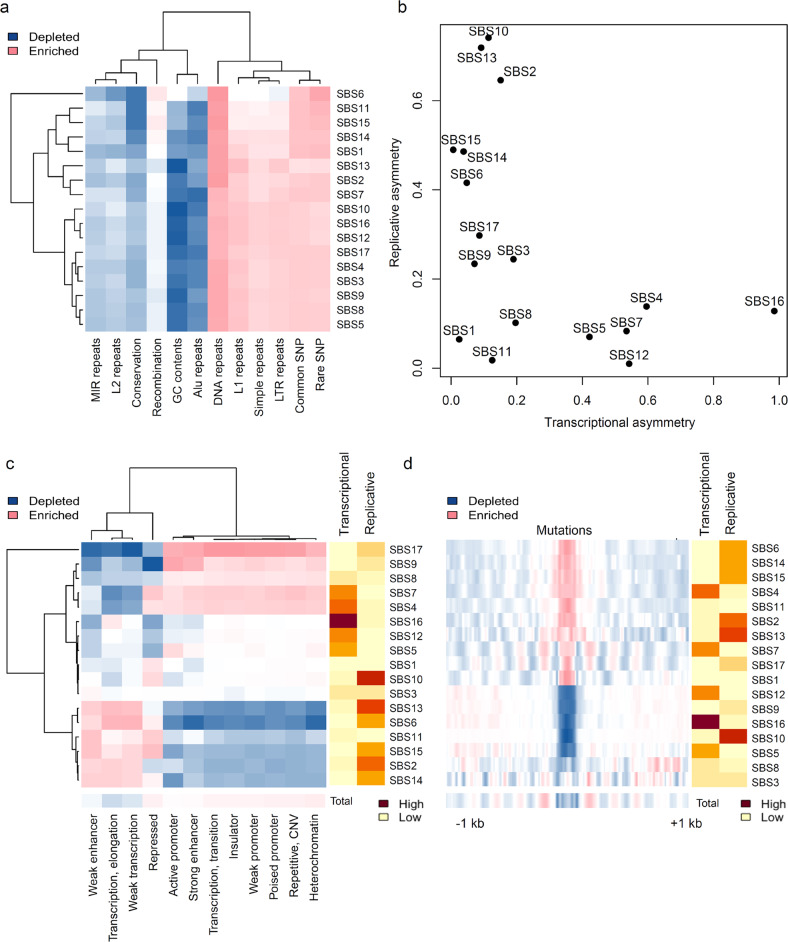
Genetic and epigenetic correlation of mutation signatures with respect to mutational strand asymmetries. **a** Mb-bin-scaled correlations of 12 genetic features with the densities of mutation signatures are shown in a heatmap. **b** Levels of transcriptional and replicative strand asymmetries (*x-* and *y*-axes, respectively) are plotted for the mutation signatures. **c** The mutation density with respect to 12 chromHMM states and mutation signatures is shown in a heatmap. Levels of mutational strand asymmetries (transcriptional and replicative asymmetries) are also shown in a heatmap. **d** Nucleosome positioning signals measured across mutation-centric 2-kb windows (1 kb up- and downstream) are shown in a heatmap aligned with mutational strand asymmetries.

The regional distribution of mutation signatures was examined to determine how the signatures correlate with various genomic and epigenetic features. First, we investigated the correlation of regional mutation density in megabase (Mb) bins (i.e., mutations per Mb) with various genomic features, including GC content, recombination rate, conservation level, and the density of repeat elements and germline variants (e.g., common and rare SNPs) (Fig. 2a). We also explored the Mb-scaled correlation of mutation signatures against the various levels of histone methylation and acetylation^14^ (Supplementary Fig. 4). We observed that the mutations in most mutation signatures consistently showed enrichment in GC-poor/late-replicating heterochromatic regions and depletion in GC-rich/early-replicating euchromatic regions (e.g., mutation numbers correlated inversely with GC content and Alu repeats and positively with the repressive histone markers H3K9me2/me3 and H3K27me3). This pattern was largely consistent on different scales (Supplementary Fig. 4), suggesting that the global, Mb-based distribution of mutation signatures largely accommodates the known features of mutations. However, the observed correlation patterns were relatively weak for SBS6 mutations related to DNA mismatch repair (MMR) deficiency^22^. This is consistent with the previous finding that Mb-scaled regional variations in somatic mutations are driven largely by regionally differential MMR activity; thus, genomes with MMR deficiencies do not exhibit characteristic regional variations in their mutation rates^11^.

It has been proposed that transcriptional and replicative mutation strand asymmetries are widespread in cancer genomes and are associated with the underlying mechanisms of DNA mutagenesis and repair^15^. We measured two-strand asymmetries across mutation signatures (Fig. 2b) and observed the SBS2/13, 6, 10, 14, and 15 mutations to be substantially biased toward a high level of replicative asymmetry but the SBS4, 5, 7, 12, and 16 mutations to be biased toward a high level of transcriptional asymmetry. We further evaluated the correlation between mutation signatures and local-scale epigenetic features by examining the enrichment level of mutation signatures in 12 distinct chromatin states (i.e., chromHMM states inferred from multiple epigenetic marks)^23^ (Fig. 2c). The mutation signatures largely segregated into those showing relative depletion or enrichment of mutations at chromatin states of “weak enhancer/transcription” and “transcription elongation,” representing “transcribed” chromatin states with H3K36me3 enrichment^23^. Notably, we observed that the relationship between mutation signatures and chromHMM states was largely concordant with each signature’s mutation strand asymmetries. For example, mutation signatures with transcriptional strand asymmetries (SBS4, 5, 7, 12, and 16) were depleted in transcribed chromatin states compared with those with an “active promoter” or “heterochromatin.” In contrast, mutation signatures with replicative strand asymmetries (SBS2/13, 6, 14, and 15) were relatively enriched in transcribed chromatin states. The distinction in mutation signatures with respect to mutational strand asymmetries and epigenetic configurations might be caused by the distinct susceptibility of chromatin states to DNA mutagenesis and repair. For example, the epigenetic states of “transcribed” chromatin could be more amenable to the activity of transcription-coupled repair (TCR)-mediated nucleotide-excision repair (NER) than to the activity of active promoter/enhancers and heterochromatins^24^. We further examined 11 ChIP-seq (chromatin immunoprecipitation sequencing) datasets representing various histone marks, as available in the ENCODE project. The read density of the ChIP-seq data at somatic mutations was measured across mutation signatures (Supplementary Fig. 5). We observed that mutations with replicative asymmetries and hypermutation-related SBS4 and SBS7 mutations showed overall enrichment in ChIP-seq signals compared to those with transcriptional asymmetries. We also observed that mutations arising in MMR deficiency (SBS6 and SBS15) were distinguishable from other mutation signatures in that they were enriched for H3K36me3/H3K20me1 signals, suggesting that these epigenetic modifications might be repair targets for MMR activity.

Nucleosome-bound and linker DNAs show differential mutation rates, possibly because nucleosome occupancy can influence access of DNA to DNA mutagenesis and repair machinery^25^. We examined the average level of MNase-seq (micrococcal nuclease sequencing) signals in 2-kb wide windows centered at each mutation with respect to mutation signatures (Fig. 2d) and observed that mutation signatures can be clearly classified into two distinct classes: one with enriched nucleosome positioning signals and one with depleted nucleosome positioning signals. Most mutation signatures with replicative strand asymmetries showed enriched nucleosome positioning signals. In addition, mutations SBS7 and SBS17 showed highly periodic patterns of nucleosome positioning signals representing alternation of nucleosome-covered and linker DNA, consistent with a previous report^26^ (Supplementary Fig. 6). Such enrichment of nucleosome positioning at mutations with signatures SBS7 and SBS17, along with a periodic pattern of nucleosome positioning signals, might result from decreased NER activity, as previously reported^26^. Mutation signatures with depleted nucleosome positioning signatures include SBS5, 8, 9, 10, 12, and 16. Except for hypermutation-related SBS4 and SBS7, mutation signatures with transcriptional asymmetries were depleted at nucleosome cores, probably due to decreased de novo mutation rates with relatively constant NER activity.

### Effects of mutation processes on coding genes

**Fig. 3:**
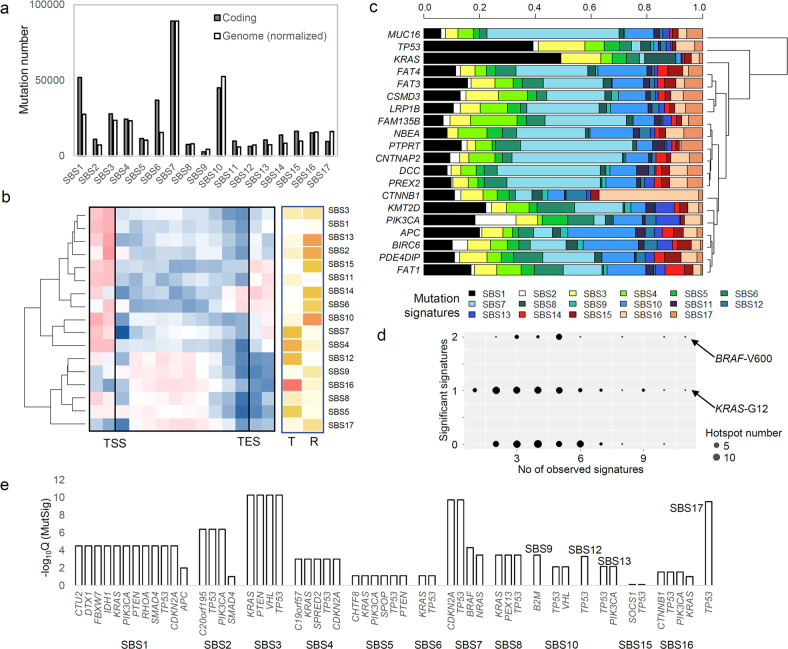
Mutation signatures in coding sequences. **a** The abundances of exonic and genomic (normalized) mutations are shown. **b** Mutation densities in individual mutation signatures are shown in 10 equal-sized bins from the transcription start and end sites (TSS and TSE, respectively), along with additional bins up- and downstream of the TSS and TSE, respectively. **c** For 20 cancer-related genes with recurrent mutations, the relative proportions of mutation signatures are shown. **d** Shown are 107 amino acid residues in cancer-related genes with no fewer than 5 occurrences, with their number of mutation signatures and significant enrichment (*x-* and *y-*axes, respectively). **e** Genes with recurrent mutations are shown (*Q* < 0.1 MutSigCV) across mutation signatures.

We examined the abundance of mutation signature calls for 426,720 coding exonic mutations and found that the signature-level coding mutation frequencies were largely concordant with those of all mutations (*r* = 0.93) (Fig. 3a). These results support that exome-scaled mutation signature profiles can be used as a proxy for genome-wide estimates of mutation processes because mutation signatures associated with high GC content, such as SBS1 and SBS6, were relatively enriched in coding mutations compared with intergenic or intronic mutations (Supplementary Fig. 7).

The mutation abundance in transcribed gene bodies was examined across mutation signatures (Fig. 3b), revealing that mutation signatures enriched toward the 5’ and 3’ gene ends but depleted within gene bodies can be distinguished from those enriched in the middle of gene bodies. This distinction is also largely concordant with the distinction between mutation signatures with replicative and transcriptional strand asymmetries. For example, mutation signatures with replicative strand asymmetries showed depletion of mutations within gene bodies, whereas those with transcriptional strand asymmetries showed enriched mutations within gene bodies, except for the hypermutation-associated SBS4 and SBS7 mutations.

Gene-level mutation signatures for 20 frequently mutated cancer genes are depicted in Fig. 3c. We observed the SBS1 and SBS7 signatures to contribute to a substantial number of mutations across cancer-driving genes; however, *TP53* and *KRAS* mutations were relatively enriched for SBS1 mutations. An association between *CTNNB1* and *PIK3CA* and enrichment of SBS16 and SBS2 was also noted. Four genes (*TP53*, *KRAS*, *PIK3CA*, and *CTNNB1*) were further investigated using previously reported hotspot amino acids^18^ to determine the composition of the mutation signatures, and we detected heterogeneous etiologies for mutation processes at those residues (Supplementary Fig. 8). Known *TP53* hotspot mutations at five residues (R175, G245, R248, R273, and R282) were mainly attributed to SBS1, which was also true for *KRAS*-G12 mutations; conversely, *KRAS-*G13 and *KRAS*-A146 mutations were largely associated with SBS15 and SBS10, respectively. Although *CTNNB1* mutations were mainly attributed to SBS16, the associations between mutation signatures and *PIK3CA* hotspot mutations were more complicated. For example, hotspot mutation E542 of *PIK3CA* was largely attributed to APOBEC-associated SBS2, whereas H1047 displayed heterogeneous etiologies. In addition, the *PIK3CA* E726 and Y1021 amino acid substitutions were exclusively associated with SBS15. The associations between mutation signatures and hotspot mutations for 107 frequently mutated (≥5 occurrences) amino acid residues in the cohort were further assessed, showing that even at the amino acid level, varying numbers of mutation signatures can be associated with individual hotspots (Supplementary Fig. 9, arrows for the *PIK3CA*-E542 and *PIK3CA*-H1047 mutations). Among the mutation signatures observed at given hotspots, we selected those with a significant enrichment of mutations per hotspot (*p* < 0.05, Fisher’s exact test; Fig. 3d). Overall, 42.9% and 14.9% of hotspots showed one and two mutation signatures, respectively, with significant enrichment suggesting the presence of a few dominant mutational processes for recurrent mutations at the amino acid level. For instance, 11 mutation signatures were called at least once for *KRAS*-G12 and *BRAF*-V600, though only one and two mutation signatures were significantly dominant (SBS1 and SBS7/SBS11 for *KRAS*-G12 and *BRAF*-V600, respectively; arrows in Fig. 3d). In addition, we used MutSigCV^19^ to identify significantly recurrent mutations (Supplementary Table 3) across mutation signatures (Fig. 3e). We observed that *TP53* mutations were commonly observed as recurrent mutation targets across mutation signatures, i.e., *TP53* mutations were observed in 12 mutation signatures, except for SBS9 with *B2M* mutations. This suggests that evolutionary selection of diverse mutational processes might be converging toward *TP53* mutations. Potential cancer drivers among the mutation signatures were also identified using dNdSCV, an evolutionary measure reflecting the level of selection for mutations^20^, to obtain genes with high dNdSCV levels in individual mutation signatures as candidate cancer drivers (Supplementary Table 4). These results highlighted *TP53*, *PIK3CA*, and *KRAS* as cancer-driving mutations under positive selection across different mutation signatures.

### Clonality and selection of mutation signatures

**Fig. 4:**
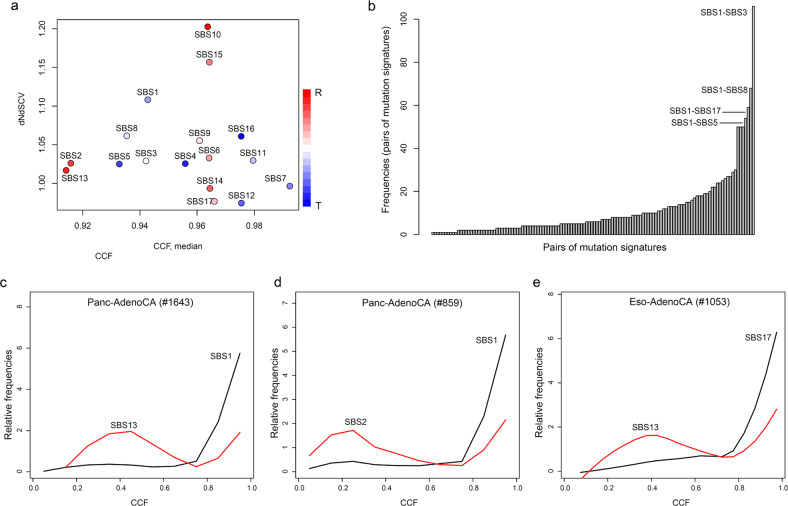
Evolutionary analysis of mutation signatures. **a** Two types of evolutionary measures for mutation signatures are shown in a scatterplot. The cancer cell fraction (CCF, *x*-axis) and dNdSCV (*y*-axis) represent levels of mutational clonality and functional selection during cancer evolution, respectively. The dot color indicates the level of replicative and transcriptional strand asymmetries (red and blue, respectively). **b** For possible pairs of mutation signatures, CCF distributions were compared for concordance using the Kolmogorov–Smirnov test. Significant pairs are shown with their frequencies in a plot highlighting recurrent pairs with SBS1 mutation signatures. The top four most frequent mutation signature pairs are shown. **c**–**e** Evolutionary trajectories are shown as relative frequencies of the CCF corresponding to two types of mutation signatures in a single genome. Three example cancer genomes (e.g., two pancreatic adenocarcinomas and one esophageal adenocarcinoma) are shown to illustrate evolutionarily discordant mutation signature pairs, including SBS1-SBS13, SBS1-SBS2, and SBS17-SBS13, which indicate early-late mutations, respectively.

Mutations belonging to different mutation signatures were separately analyzed for levels of clonality and evolutionary selection (Fig. 4a). We used the CCF as a surrogate measure of the level of clonality in mutations (*x*-axis, Fig. 4a) and dNdSCV^27^ (*y*-axis, ratio of nonsynonymous to synonymous substitutions, Fig. 4a) to measure the selective pressure to which mutations have been subjected. Mutation signature-level CCF and dNdSCV are shown separately in Supplementary Fig. 10. In general, the combined picture of mutation selection and clonality provides information about the evolutionary history of mutations. For example, SBS10 and SBS7 mutations, which are associated with the hypermutated genomes of *POLE*-mutated colorectal cancers and melanoma, respectively, might have undergone distinct evolutionary trajectories. SBS10 mutations display the highest level of selection (i.e., dNdSCV = 1.20), suggesting that mutations generated via loss of proofreading activity by DNA polymerase ε (Pol ε) are abundant but also subject to a substantial level of positive selection. In contrast, SBS7 mutations might have been fixed early in cancer genomes, as inferred from their high CCF values but relatively low evolutionary selection pressures (i.e., dNdSCV = 0.98). Additionally, the SBS2 and SBS13 mutations generated by APOBEC cytidine deaminase are subclonal, with low CCF values, indicative of their younger evolutionary age.

We next identified pairs of mutation processes that have been operative in an evolutionarily exclusive or discordant manner by using pairwise comparison of the distribution of mutation signature-specific CCF values for individual cancer genomes. A total of 1412 mutation signature pairs with CCF values that were significantly discordant in given cancer genomes (Kolmogorov–Smirnov test, false discovery rate <0.05) were taken as candidate mutation signatures that have been operative on different time scales within a given cancer genome (Supplementary Table 5). The frequencies of the signature pairs are provided in Fig. 4b. The top six most frequent mutation signature pairs are (in order of frequency) SBS1 with SBS3 (i.e., SBS1–SBS3), SBS8, SBS17, SBS5, SBS2, and SBS13, suggesting that SBS1 mutations occurred early in cancer genome evolution and were followed by other mutation signatures. This is consistent with the previous notion that SBS1 is an age-related mutation signature attributable to lifelong accumulation of C-to-T transition errors at CpG dinucleotides^28^. Case-specific examples of evolutionarily discordant mutation signature pairs are illustrated in Fig. 4c–e. We observed that SBS1, as a founder clone mutation, was followed by relatively younger APOBEC-related mutations, such as SBS13 and SBS2, in two pancreatic adenocarcinoma genomes (Fig. 4c, d). An esophageal cancer genome showed that reactive oxygen-related mutagenesis of SBS17 might have occurred early, followed by APOBEC-related mutagenesis producing SBS13 mutations. These results show how different mutational processes have been operative over different time scales in individual cancer genomes.

### Kataegic events associated with multiple mutagenic processes

**Fig. 5:**
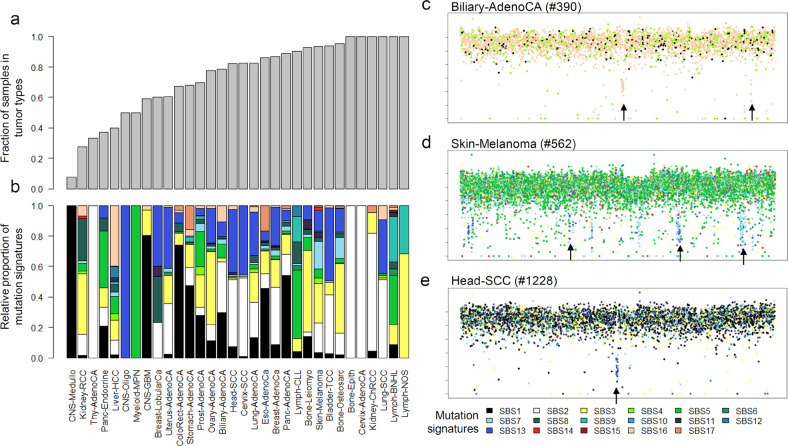
Kataegis with respect to mutation signature. **a** Relative frequencies of kataegic events are shown across tumor types. **b** For kataegic events in given tumor types, the relative frequencies of mutation signatures are shown. **c**–**e** Kataegic events with respect to mutation signatures are shown for three cancer genomes. In biliary adenocarcinomas (**c**), two kataegic events (arrows) are attributed to SBS16 mutations, suggesting that kataegis may arise with tumor-type-specific mutations. In one melanoma genome (**d**), three kataegic events (arrows) include a significantly enriched number of mutations arising from two mutational processes (SBS7 and SBS13). In one head and neck cancer genome (**e**), the selected example of kataegis (an arrow) includes mutations enriched for three mutation signatures (SBS7, SBS11 and SBS13).

Kataegis represents a single, catastrophic event in which a DNA segment becomes vulnerable to a single mutational process such as APOBEC activity. It has mainly been assumed that single-stranded DNA is the target of APOBEC activity. We identified 7482 kataegic events in 1149 nonhypermutated genomes (observed in 68.3% of 1683 cases examined, Supplementary Table 6), consistent with the previous observation that kataegis is prevalent across cancer genomes^2^. The frequency of kataegic events was particularly high for some tumor types. For instance, all cases of B-cell lymphomas, lung small cell carcinomas, and renal cell carcinomas examined in this study harbored kataegic events (Fig. 5a). Across all tumor types, the APOBEC-associated mutation signatures of SBS2 and SBS13 contributed to most mutations in kataegic events. The AID (activation-induced cytidine deaminase)-associated mutation signature SBS9 contributed to a large proportion of kataegic events in hematologic cancers (Fig. 5b). The composition of mutation signatures in kataegic events can be variable. For example, the kataegic events on chromosomes 7 and 20 of one biliary adenocarcinoma genome are largely attributed to SBS16 mutations (arrows, Fig. 5c), suggesting that lineage-specific mutagenic events can occur in a localized manner. Although we excluded hypermutated genomes, a large proportion of mutations in the kataegis of melanoma genomes are attributed to UV-associated SBS7 mutations. Figure 5d shows multiple kataegic events in a single melanoma genome, with SBS7 and SBS13 mutations being mainly observed in kataegic loci across the genome. To identify a robust association between mutagenic processes and kataegic events, we used Fisher’s exact test to determine the extent of enrichment of mutations belonging to individual mutation signatures in given kataegic events and cases. Among the 21 kataegic events observed in the melanoma genome illustrated in Fig. 5d, three events were enriched with both the SBS7 and SBS13 mutations (*p* < 0.05, Fisher’s exact test, arrows indicated, Fig. 5d). By examining all kataegic events for enrichment of multiple mutation signatures, we identified 4 and 189 kataegic events with significant enrichment for 3 and 2 mutation signatures, respectively. The mutation signature pairs most frequently observed in single kataegic events were SBS2 and SBS13 (62 of 189 events, 34.6%), followed by SBS7 and SBS2/13 (41 of 1389 events, 21.7%). Among the genomes with three enriched mutation signatures, one example of a head and neck cancer case is depicted with an arrow in Fig. 5e. This kataegic event occurred in chromosome 8 and harbored 7 SBS7, 8 SBS11, and 13 SBS13 mutations in a single locus.

## Discussion

Mutation signatures have been used to infer the types and relative contributions of mutational processes that have been operative in individual cancer genomes^4,5^. Recent efforts have identified novel mutation signatures and their associated causalities, facilitating etiology-based mutation analyses^29–31^. Although mutation signature analyses have become indispensable components of cancer mutation analyses; most studies have analyzed mutation signatures at the level of individual cancer genomes. Several studies have proposed algorithms to assign individual mutations to mutation signatures^6,26,32^, but those methods are based on sample-level mutation signatures and largely ignore regional variations in mutation spectra. Instead of applying regression methods^19,20^, we performed segmentation and clustering of somatic mutations using a multinomial model. Then we used mutation clusters, as sets of nonadjacent genomic segments, as templates for assigning mutation signatures to individual somatic mutations. Compared with sample- or segment-level assignments, cluster-based mutation signature assignments are more sensitive for discovering lineage-specific mutation signatures. We also demonstrate that measures of mutation clonality (CCF) and replication timing (Repli-seq) are more concordant for mutation clusters than for randomly assigned sets of mutation segments.

In this study, relative enrichment of somatic mutations in late-replicating, GC-poor, heterochromatic regions compared to early-replicating, GC-rich, euchromatic regions^8–10^ was observed for all mutation signatures except SBS6 mutations arising from MMR deficiencies. Because DNA MMR corrects DNA mismatch with 5-methylcytosines in early-replicating regions, SBS6 mutations that arise from a deficiency in the MMR pathway will show a more uniform mutation distribution across genomes than mutations that arise without MMR deficiencies (SBS1)^11^. This indicates that the observed Mb-scaled correlation of most mutation signatures (including SBS1) with replication timing might largely be due to differential DNA repair activities^11,33^.

We also investigated the mutation densities of different mutation signatures in the chromatin states defined by chromHMM^23^. We distinguished mutation signatures according to their mutational strand asymmetries^15^ and found that mutations with transcriptional and replicative strand asymmetries had distinct mutation densities in different chromatin states. Signatures with replicative strand asymmetry were more enriched in transcribed states, such as “weak enhancer/transcription” and “transcription elongation” whereas signatures with transcriptional strand asymmetry were more enriched in other epigenetic states, including promoter or repressed chromatin states. The observed relationship between mutation signatures and chromatin states suggests that transcribed chromatin might be more accessible to TCR-NER activity. For example, SBS4 and SBS7 mutations arise from well-recognized exogeneous mutagens, tobacco and UV, respectively^34,35^. Their resulting DNA adducts are recognized and repaired by TCR-NER, such that those mutation signatures exhibit a marked level of transcriptional strand asymmetry and relative depletion in transcribed chromatin states. In addition, mutations related to MMR defects (SBS6) showed contrasting epigenetic correlation with mutations with transcriptional asymmetries, suggesting that MMR activity targets epigenetic configurations that are also vulnerable to NER activity; thus, MMR deficiency leads to accumulation of mutations at transcribed chromatin. It was recently reported that the H3K36me3 histone mark activates MMR^36^ and that this histone mark is associated with transcribed chromatin states^23^. These findings suggest that MMR activity shares target epigenetic configurations, such as transcribed chromatin states, with TCR-NER. Thus, it is assumed that the H3K36me3 histone mark and its preferred epigenetic configurations might favor the activity of both MMR and TCR-NER.

Mutations that arise through activity of the APOBEC deaminase (SBS2 and SBS13) are known to occur mainly on lagging strands. Mutations on lagging strands might be relatively free from the effects of TCR-NER, leading to the marked replicative strand asymmetry of SBS2 and SBS13 mutations (Supplementary Fig. 11), along with relative enrichment of those mutations in the transcribed chromatin state (Fig. 2c). SBS10 mutations, on the other hand, have marked replicative strand asymmetry (Supplementary Fig. 11) and show a relatively flat distribution across chromatin states (Fig. 2c). The replicative strand asymmetries of SBS10 mutations are largely attributed to the role of *POLE* as a proofreading polymerase for leading strands. In addition, it has been proposed that MMR activity uses lagging strands as “parental” strands to correct replication errors on the leading strand^15^. This particular behavior of MMR facilitates fixation of SBS2/SBS13 mutations that occur on lagging strands, but it may also facilitate repair of SBS10 mutations that occur on leading strands, producing differential epigenetic correlative patterns for SBS2/SBS13 and SBS10 mutations.

Interestingly, other than the transcribed states, the remaining chromatin states, including active promoter, enhancer, and heterochromatin, behave in a similar manner. One possible explanation is that binding of transcription factors renders open chromatin similar to closed chromatin, thus reducing local NER and base-excision repair (BER) activities and leading to elevated mutation rates in promoter regions^37^. Furthermore, our mutation-centric examination revealed that most of the examined ChIP-seq signals corresponding to various histone marks are consistently elevated and depressed for mutation signatures with replicative and transcriptional strand asymmetries, respectively. More investigation is needed to reveal the relationships between mutational strand asymmetries and local-scale epigenetic features.

Nucleosome positioning might affect the local genomic distribution of mutation signatures^26,38^. Consistent with a previous report that nucleosome-bound DNA has higher mutation rates than DNA in linker regions^25^, our results show nucleosome positioning signals to be enriched at the mutations of most signatures. It has previously been assumed that mutations with transcriptional strand asymmetry, such as SBS7, are targets of global NER and that NER is less efficient at nucleosomes than at linkers, leading to elevation of UV-related SBS7 mutations at nucleosomes^39,40^. Nevertheless, our results show that among the signatures with elevated nucleosome positioning signals, most have replicative strand asymmetry, with only SBS4/SBS7 having transcriptional strand asymmetry. The elevated mutation frequency of SBS17 at nucleosomes was proposed to be associated with other DNA repair mechanisms, such as BER^26^. Thus, mutation signatures showing elevated nucleosome positioning signals might be associated with either decreased NER or another repair mechanism, such as BER, at nucleosome-bound DNA. The SBS2/SBS13 and SBS10 mutation signatures with marked replicative strand asymmetries also exhibited contrasting patterns of enrichment and depletion, respectively, for nucleosome positioning signatures. It is assumed that the preferences of those mutation signatures for lagging and leading strands might explain this contrasting pattern, e.g., MMR preferentially repairs replicative errors on leading strands^15^. Regardless, determining relative depletion of nucleosome positioning signals for AID-associated SBS9 and hepatocellular carcinoma-associated SBS12/16 will require further investigation.

We observed that the abundances of mutation signatures in exonic regions are largely concordant with those of genome-scale estimates, indicating that mutation signatures obtained based on whole-exome data can largely represent the mutation signatures of the entire genome. We also observed that mutation signatures enriched toward the 5’ and 3’ gene ends can be distinguished from those enriched in the middle of gene bodies. This distinction is associated with replicative strand asymmetry. Indeed, the relative depletion of mutations with transcriptional strand asymmetry at the 5’ and 3’ ends suggests that gene boundaries are more vulnerable than gene bodies to TCR-NER. On the other hand, enrichment of mutations with transcriptional strand asymmetry at gene bodies suggests that they might be subject to DNA repair mechanisms other than TCR-NER. It was previously reported that exonic regions are depleted of mutations in MMR-proficient, but not MMR-deficient, tumors^41^, suggesting that gene body-specific depletion might be related to MMR instead of TCR-NER.

The relative homogeneity of mutational processes at the amino acid level highlights that specific hotspot-level mutations can be evaluated in terms of a limited number of causative mutational processes (e.g., *KRAS*-pG12 associated with SBS1). However, homogeneity at hotspots should not be overstated because ~45% of the most frequently mutated hotspots do not have a major mutation signature but are rather associated with multiple mutation signatures (e.g., *PIK3CA*-p.H1047). In addition, we observed *TP53* mutations to commonly be associated with diverse mutation signatures, highlighting the effects of multiple mutational processes as they converge on *TP53* mutations.

In this study, we demonstrate that CCF-based mutation signatures can be delineated to show the time-scaled activity of various mutations. Despite an effort to perform mutation signature analyses using bins of mutations with similar CCFs^42^, our mutation signature map at the resolution of individual mutations can directly assess the evolutionary concordance of pairs of mutational processes. Although it is expected that SBS1, the lifelong accumulation of C-to-T at methylated cytosines, might be as an early mutagenic process that is followed by later mutation processes such as APOBEC activity, our example case of esophageal cancers further demonstrates that oxidative attacks (SBS17) may precede APOBEC activity (SBS13).

According to our study, most kataegic events have APOBEC-related mutation signatures (SBS2 and SBS13), but some events harbor more than one mutation signature. Whether these unique events represent separate evolutionary events (e.g., localized APOBEC activity in the prevalence of SBS7 mutations in melanoma genomes) or indicate that multiple mutational processes can simultaneously act on a DNA segment in a localized manner to generate kataegis events will require further evidence.

One limitation of this study is that we used a subset of mutation signatures, i.e., the 17 mutation signatures available in COSMIC Ver. 2 database. Although the mutation signatures used in the study cover more than 90% of the somatic mutations present in the dataset, it should be noted that the remaining mutation signatures, as well as newly reported ones (e.g., 70 SBS signatures in COSMIC Ver. 3^27^ and novel mutation signatures^36^), might provide additional genomic or epigenomic insights beyond those in our study. Epigenetic features often show tissue- or cell type-specific patterns, and thus, cell type-specific analyses of cancer mutations can leverage their tumor-type-matching epigenetic features. In this study, epigenetic features ranged from a limited number to tissue or cell types, and such issue should be further explored by using extended epigenetic features that can cover major tumor lineages.

## Supplementary information

Supplementary data

## Data Availability

All mutation data used in the study were obtained from the ICGC portal (https://dcc.icgc.org/). All datasets generated for this study are included in the manuscript and the Supplementary Files.

## Code availability

Codes are available at https://github.com/wanghanmath/regional_mutation_signature.
