# Supplementary material for: The shaping of cancer genomes with the regional impact of mutation processes
Source: Exp Mol Med. 2022 Jul 28;54(7):1049–60. doi: 10.1038/s12276-022-00808-x (PMC9355972; doi:10.1038/s12276-022-00808-x)
Supplement: Supplementary file 1 — Supplementary data [file 12276_2022_808_MOESM1_ESM.pdf]

Supplementary figures and tables

Supplementary Fig. 1. Frequencies of mutation signatures with respect to tumor types.

Supplementary Fig. 2. The sensitivity of discovery for the lineage-specific mutation signatures.

Supplementary Fig. 3. Concordance of epigenetic and genetic features of mutation clusters.

Supplementary Fig. 4. Genomic and epigenetic correlation.

Supplementary Fig. 5. Histone marks enrichment and depletion around the mutations.

Supplementary Fig. 6. Mutation density around nucleosome positioning.

Supplementary Fig. 7. Mutation signature composition per genomic region.

Supplementary Fig. 8. Composition of mutation signatures at amino acid hotspots.

Supplementary Fig. 9. Frequency of mutation signatures in 107 recurrently mutated amino acid hotspots.

Supplementary Fig. 10. The level of evolutionary selections and clonality of mutation signatures.

Supplementary Fig. 11. Strand asymmetry related to DNA replication.

Supplementary Table 1. Tumor types.

Supplementary Table 2. Composition of mutation sigantures of ICGC data.

Supplementary Table 3. MutSig results.

Supplementary Table 4. Driver mutations with high level of positive selections.

Supplementary Table 5. Evolutionarily distinct mutational processes in individual cancer genomes.

Supplementary Table 6. Kataegis events and mutation signatures.

Supplementary Fig. 1. Frequencies of mutation signatures with respect to tumor types. For 17 mutation signatures (SBS1 to SBS17), the mutation frequencies (*y*-axis) are plotted across 24 tumor types supported by more than 10 samples.

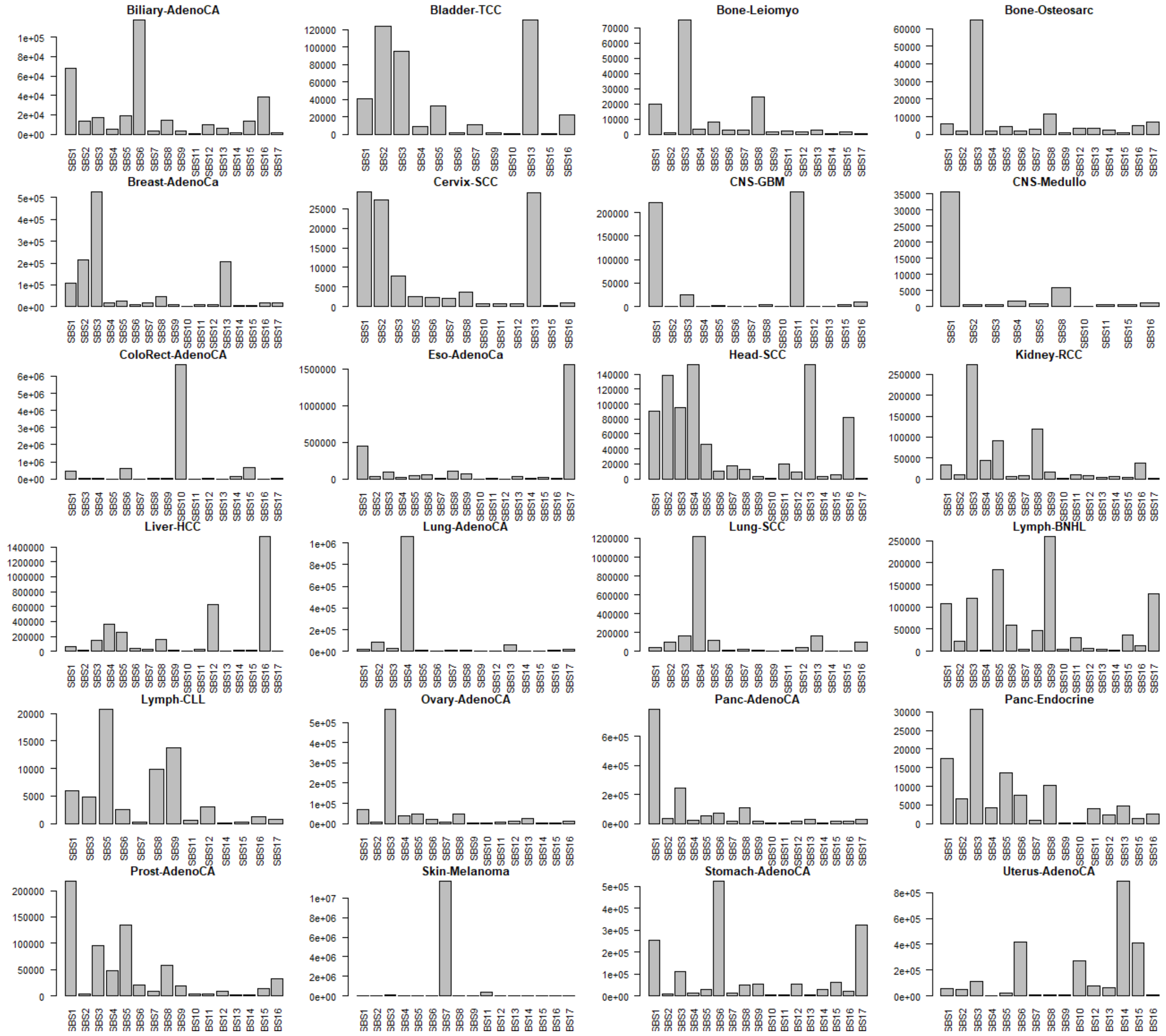

Supplementary Fig. 2. The sensitivity of discovery for the lineage-specific mutation signatures. For three tumor types of lung adenocarcinomas (Lung-AdenoCA), cutaneous melanomas (Skin-Melanoma) and hepatocellular carcinomas (Liver-HCC), the extent of recoveries of 17 mutation signatures are compared for sample *vs.* cluster (upper panels) and segment vs. cluster scales (lower panels). Both comparison reveals that the lineage-specific mutation signatures such as SBS4 (lung cancers), SBS7 (melanomas) and SBS16 (liver cancers) are more discovered in mutation cluster-based assignments than those based on sample- or segments.

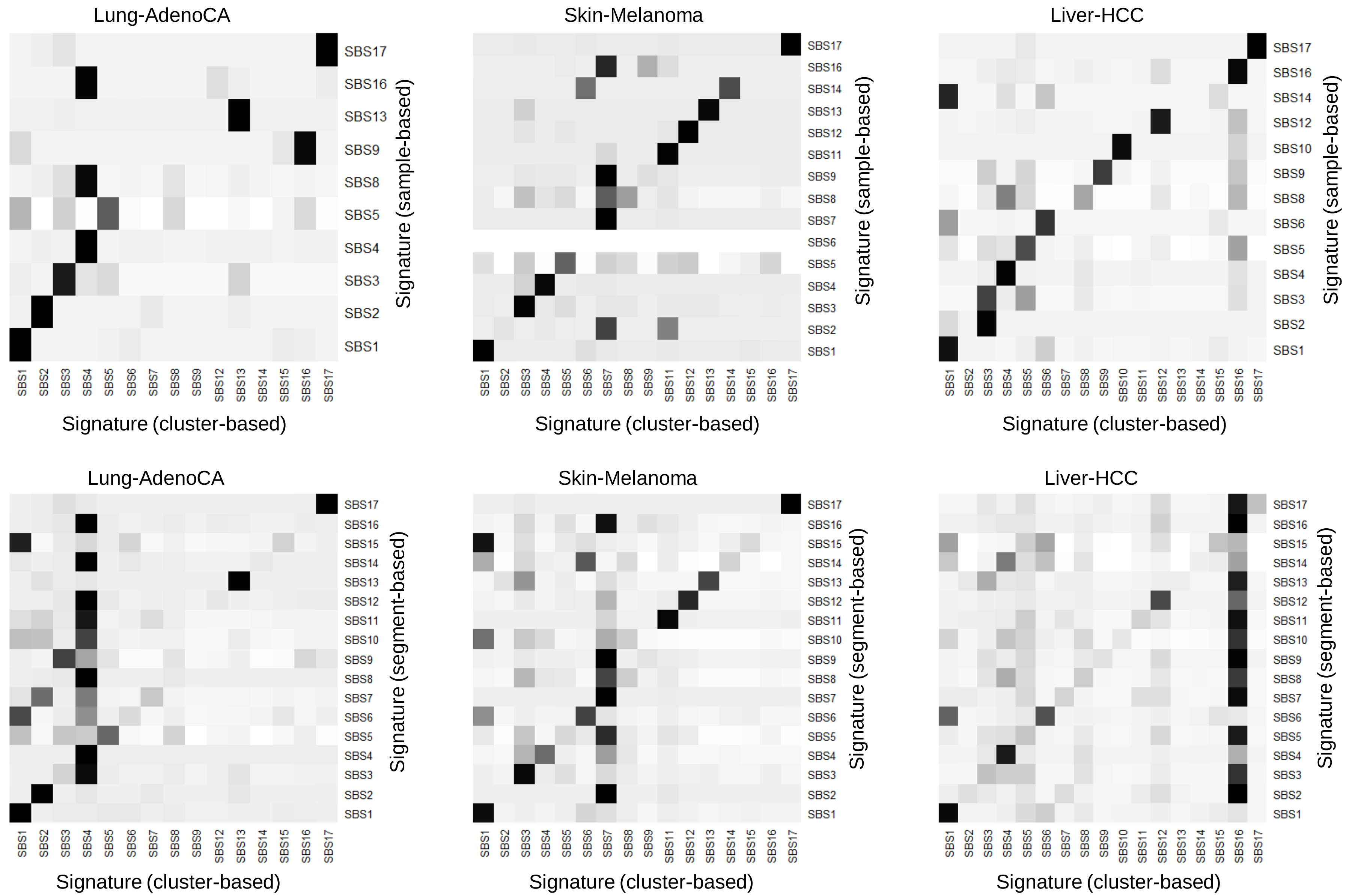

Supplementary Fig. 3. Concordance of epigenetic and genetic features of mutation clusters. Density plot of *P* value (-log10 scales) were estimated for epigenetic (Repli-seq, left) and cancer cell fractions (CCF, right) are shown for individual tumor types. for each cancer type.

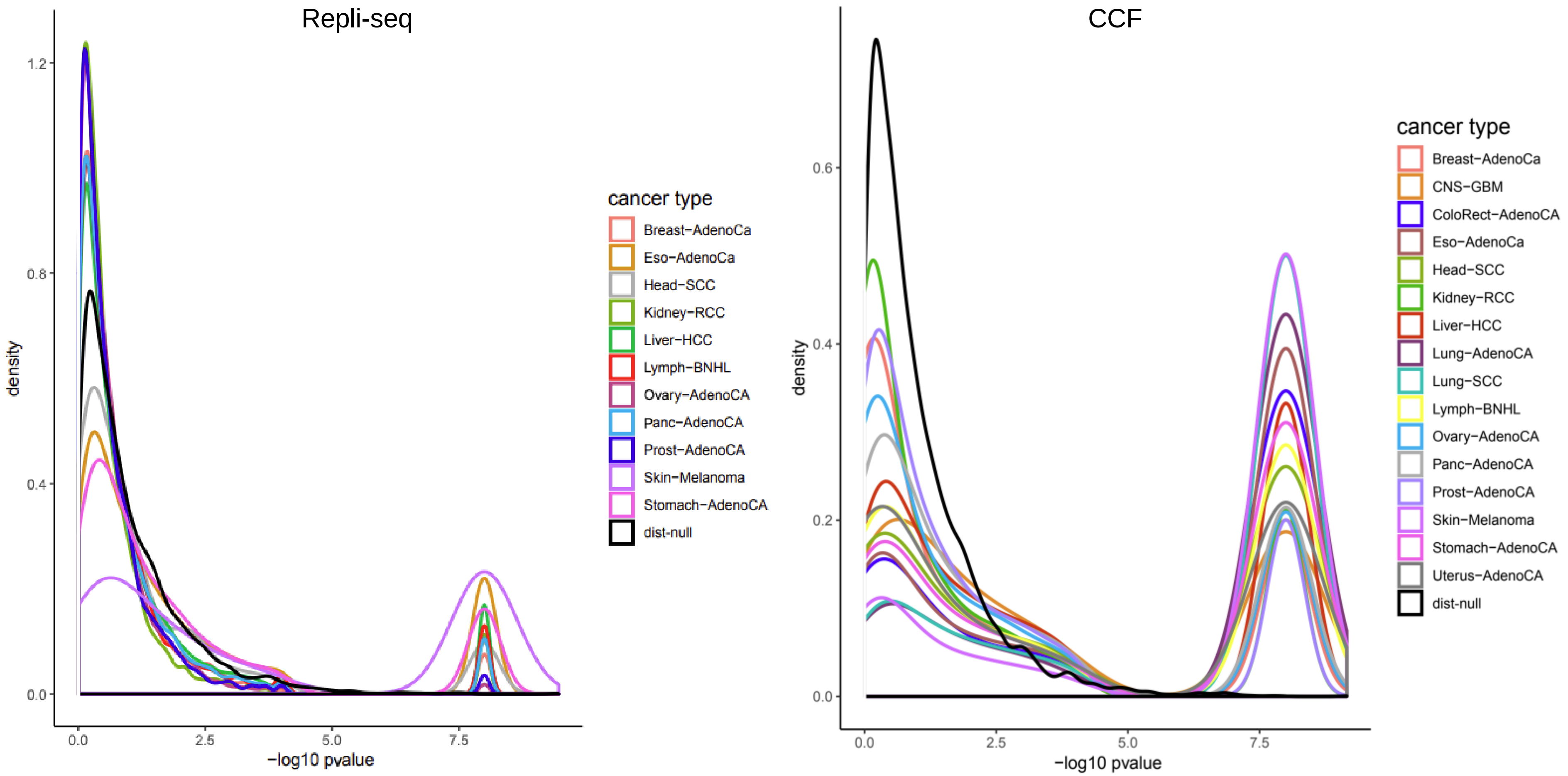

Supplementary Fig. 4. Genomic and epigenetic correlation. Various genetic and epigenetic features were measured in 1Mb and 100kb bins across genomes and correlated with the mutation density of mutation signatures. Heatmap shows scaled Pearson correlation coefficients (red and blue represent the high and low level of correlations, respectively).

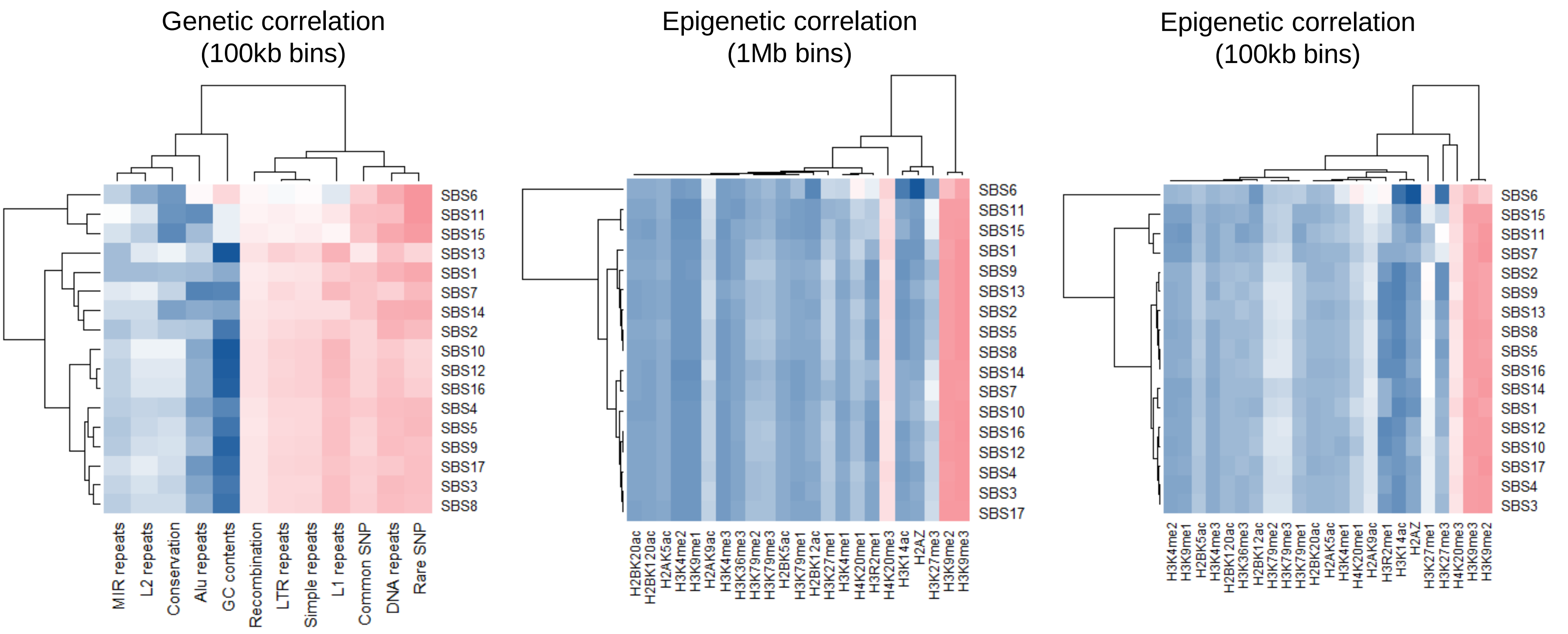

Supplementary Fig. 5. Histone marks enrichment and depletion around the mutations. (a) A total of 11 ChIP-seq datasets were obtained and the mutation-centric ChIP-seq signals are visualized with a heatmap visualization. Mutational strand asymmetries are shown in the order of mutation signatures. (b) ChIP-seq signals are shown in mutation-centric 2Kb windows. The signals are colored according to the level of replicative/transcriptional strand asymmetries as shown in a color bar (red and blue represent the replicative- and transcriptional mutation strand asymmetries, respectively)..

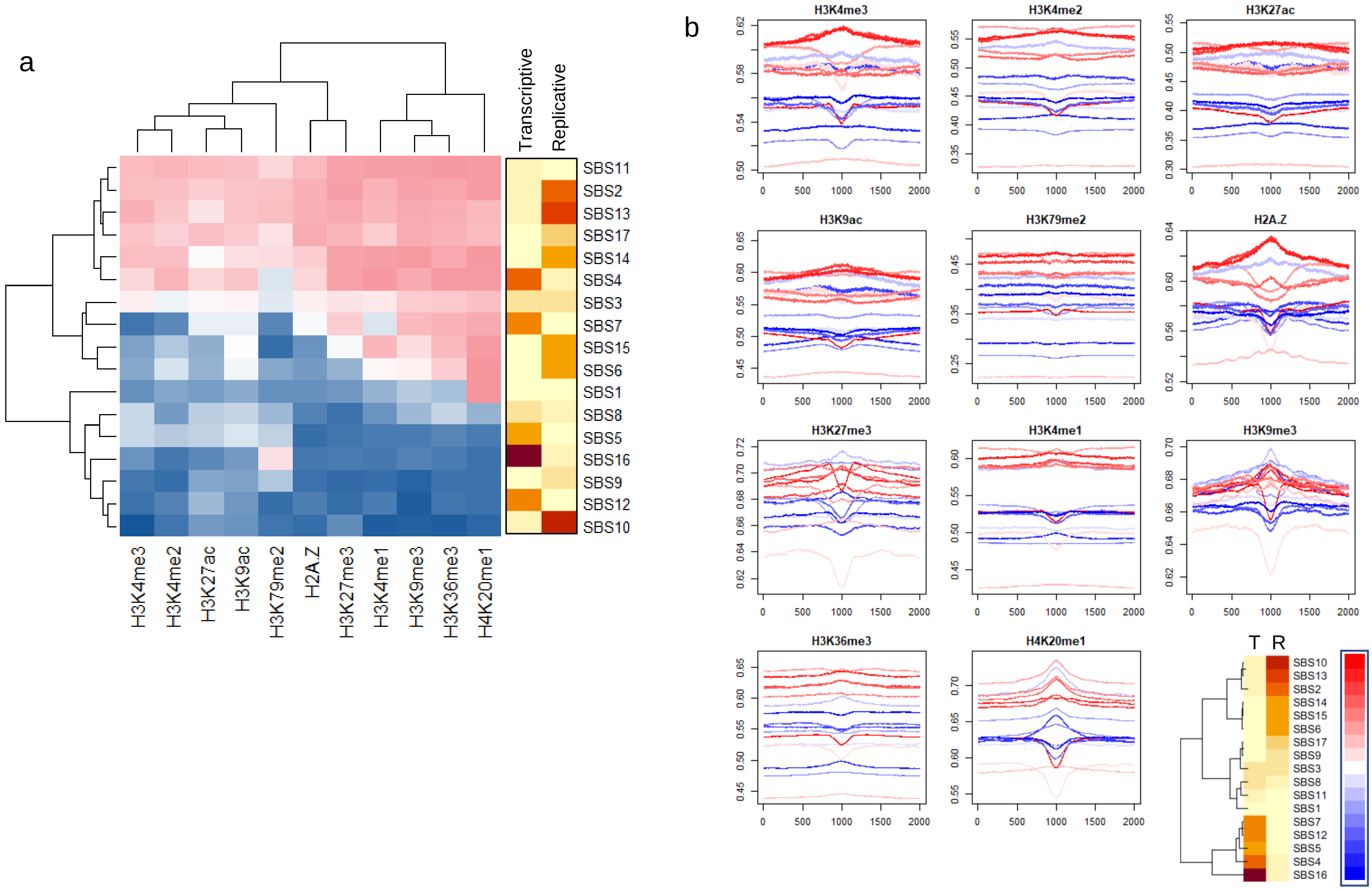

Supplementary Fig. 6. Mutation density around nucleosome positioning. The density of MNase-seq signals (K562 celllines) at the mutations are averaged across the mutation signatures and plotted in mutation-centric 2Kb windows.

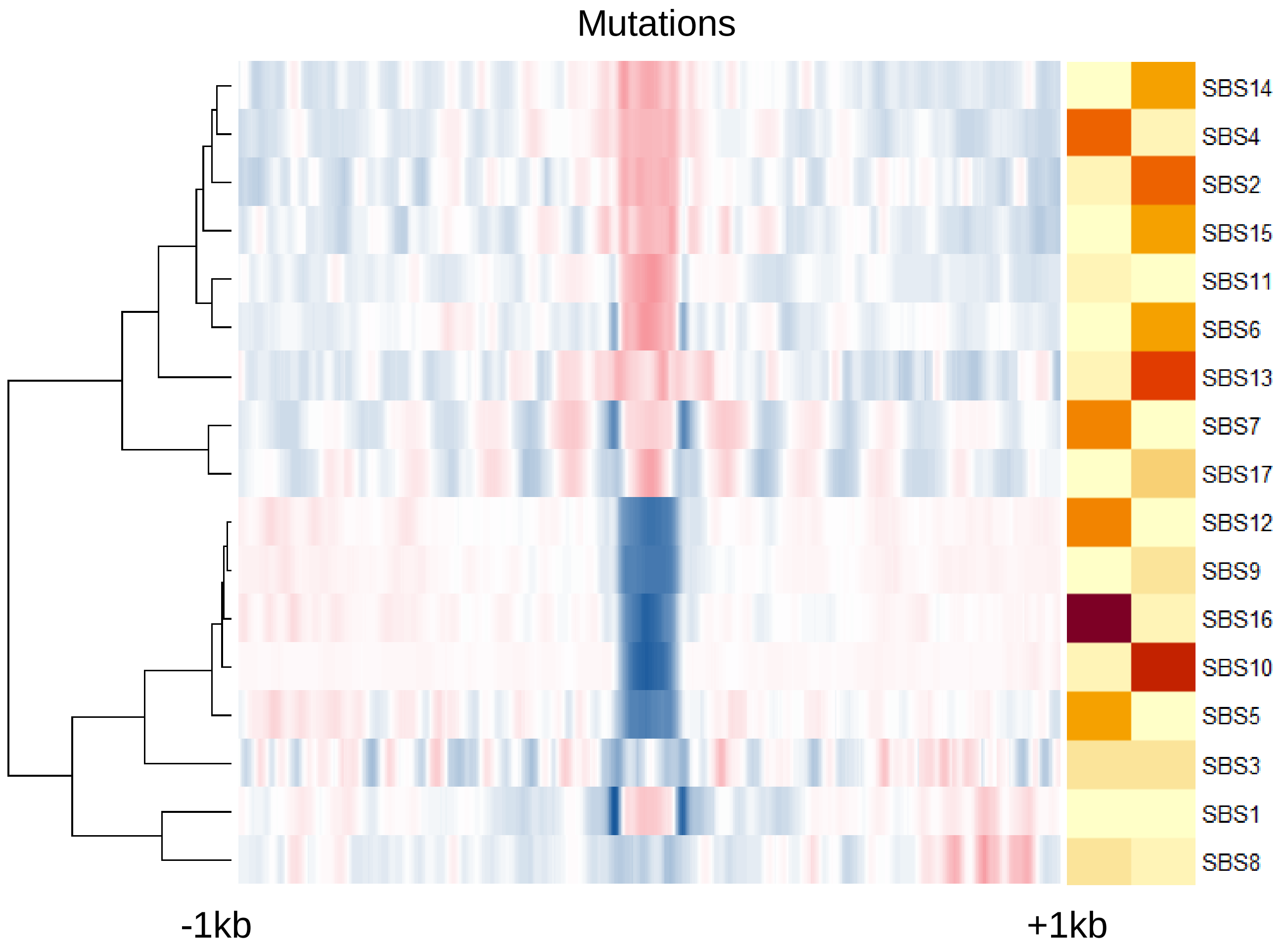

Supplementary Fig. 7. Mutation signature composition per genomic region. Relative abundance of mutations belonging to different mutation signatures are estimated across exonic-intronic-intergenic regions (left) and also for functional categories (right).

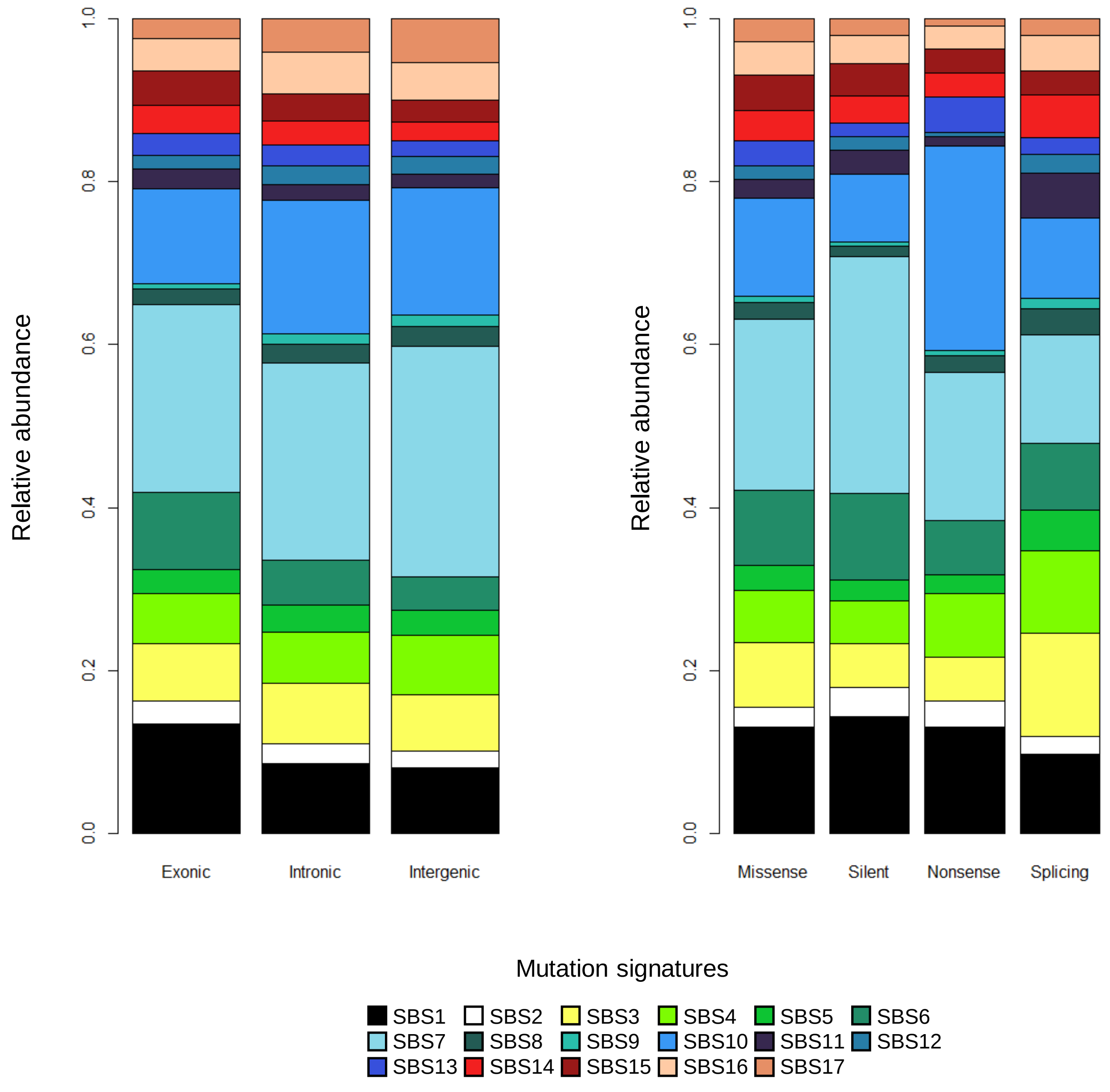

Supplementary Fig. 8. Composition of mutation signatures at amino acid hotspots. Four cancer-related genes of *TP53*, *KRAS*, *PIK3CA*, and *CTNNB1* are shown.

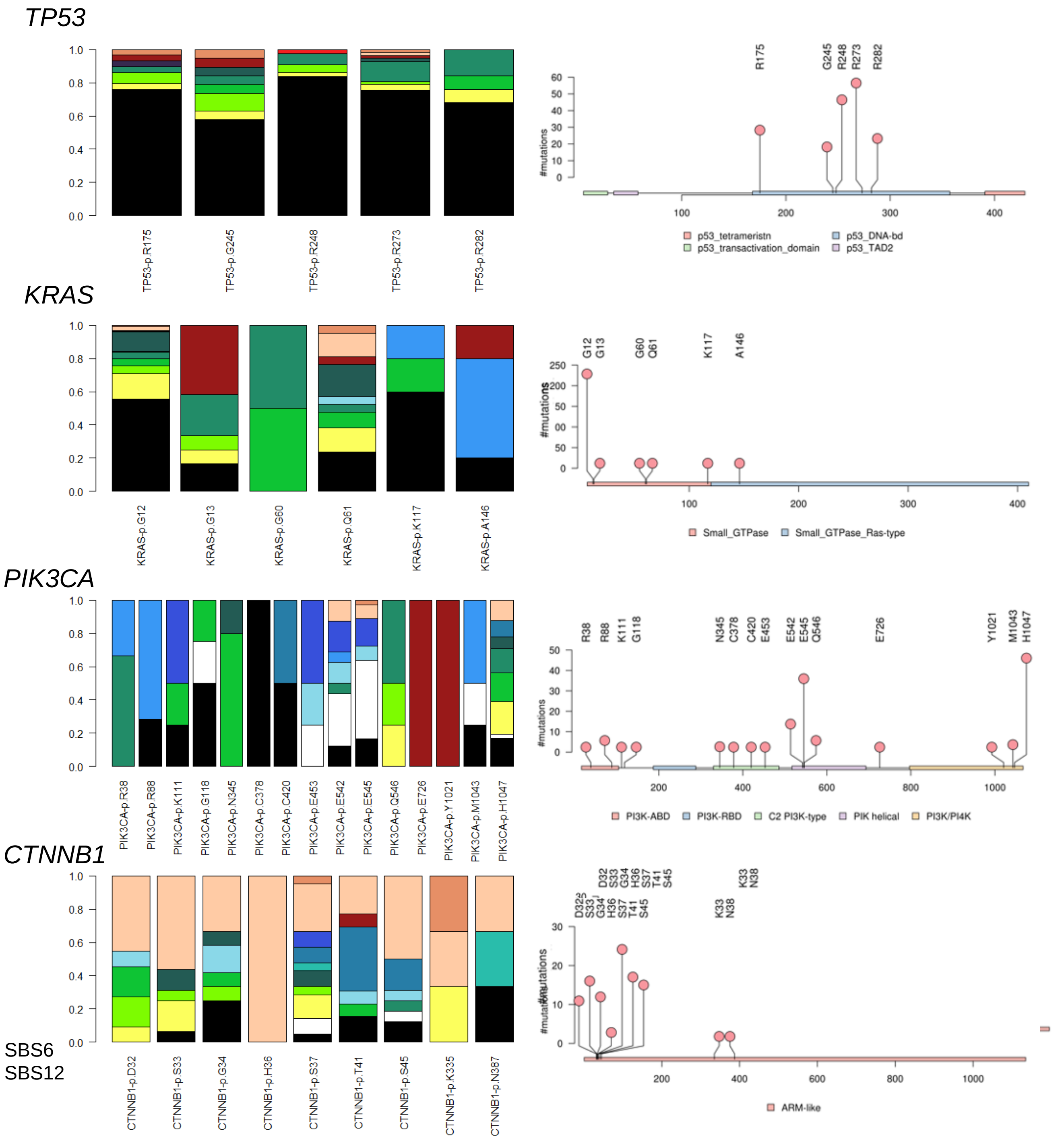

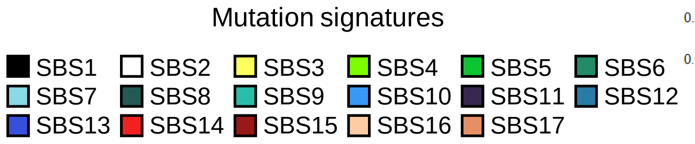

Supplementary Fig. 9. Frequency of mutation signatures in 107 recurrently mutated amino acid hotspots. Arrows indicate two examples of heterogeneous etiologies of mutational processes (*PIK3CA*-E542 and –H1047) along with those with dominant etiologies (*KRAS*-G12 and *BRAF*-V600 with SBS1 and SBS7/SBS11, respectively).

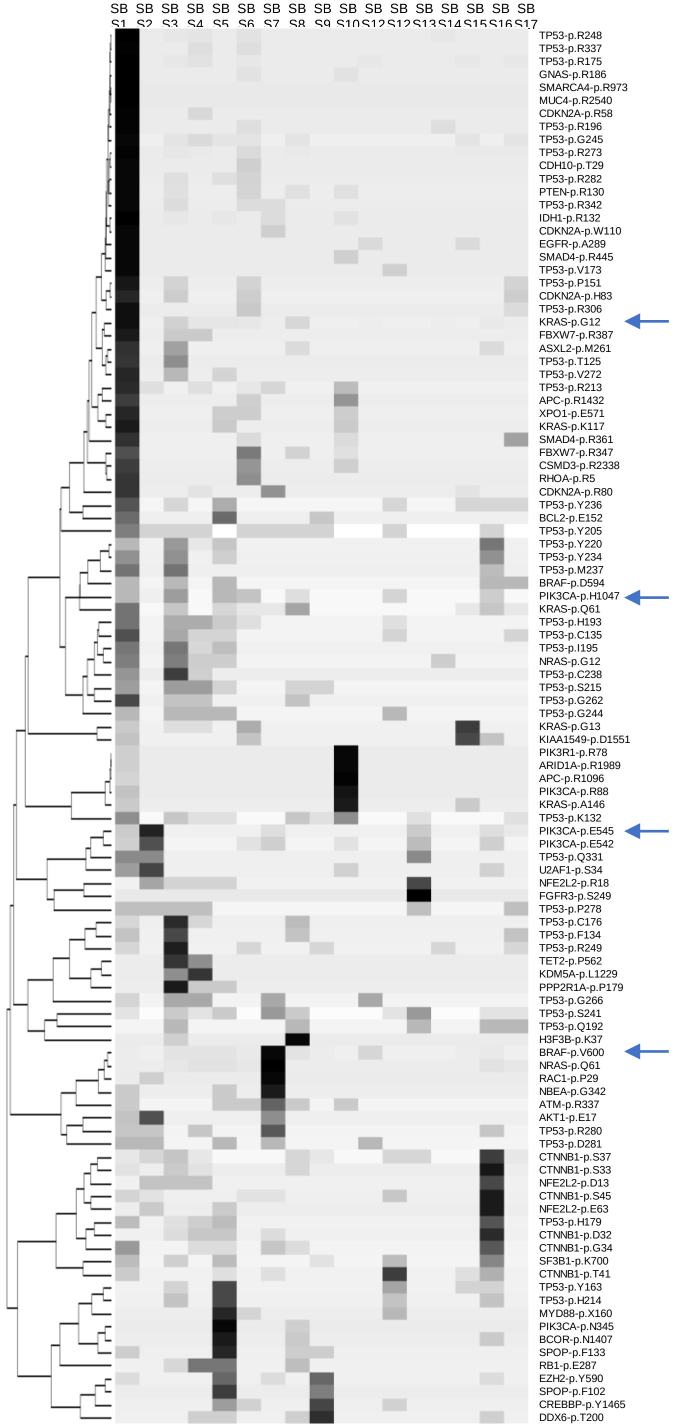

Supplementary Fig. 10. The level of evolutionary selections and clonality of mutation signatures. Two measures of the evolutionary selection (dNdSCV) and mutation clonality (cancer cell fraction, CCF) are plotted across 17 mutation signatures.

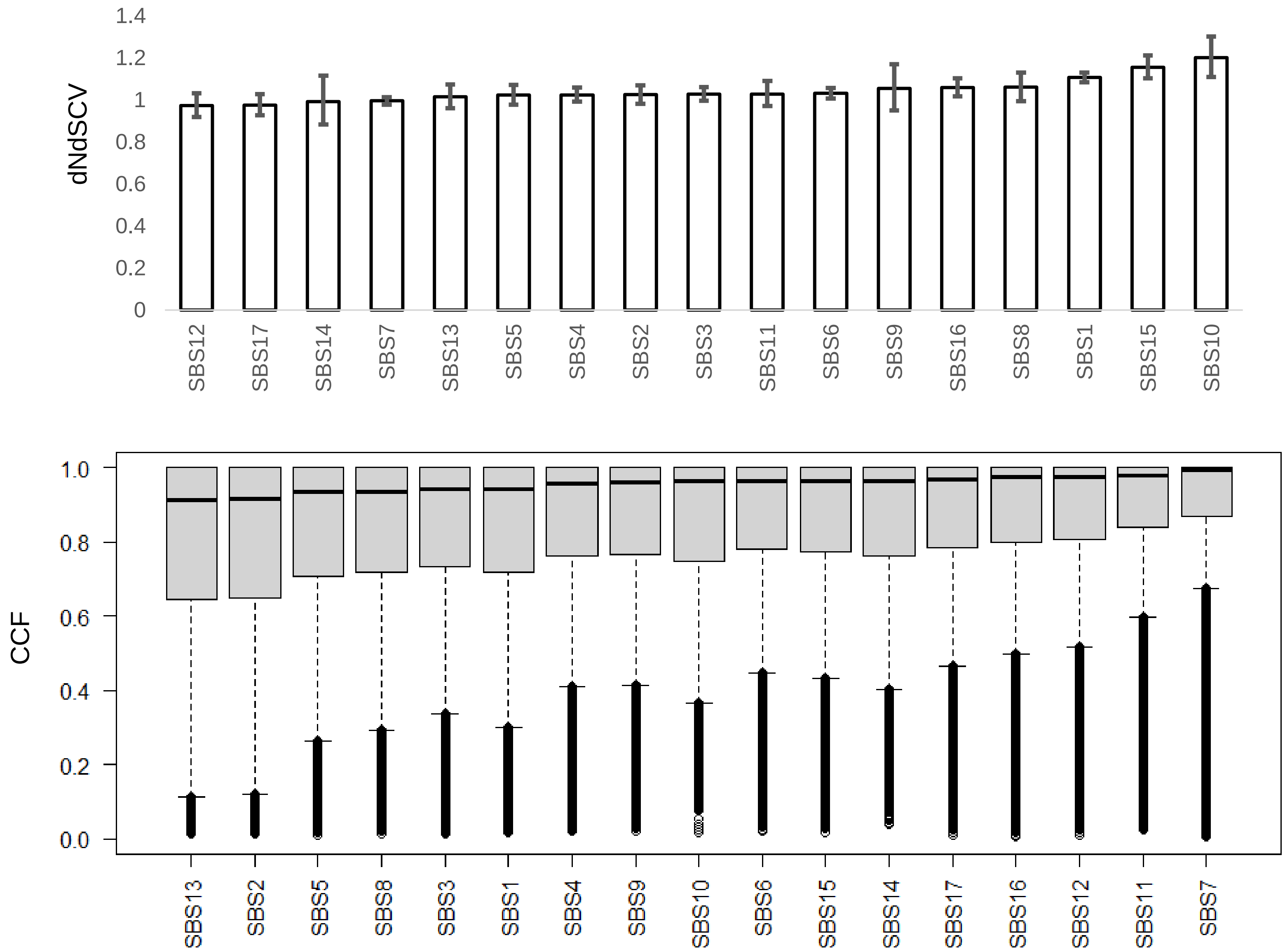

Supplementary Fig. 11. Strand asymmetry related to DNA replication. APOBEC associated signatures of SBS2 and SBS13 show a relatively higher density of C>A, C>G, C>T type of mutations in right-replicating regions representing lagging strands, whereas POLE associated signatures (SBS10 and SBS14) had the same pattern for left-replicating regions representing leading strands. Error bars represent 95% confidence intervals. *Y*-axis shows the log2 ratio of mutation density per mutation type.

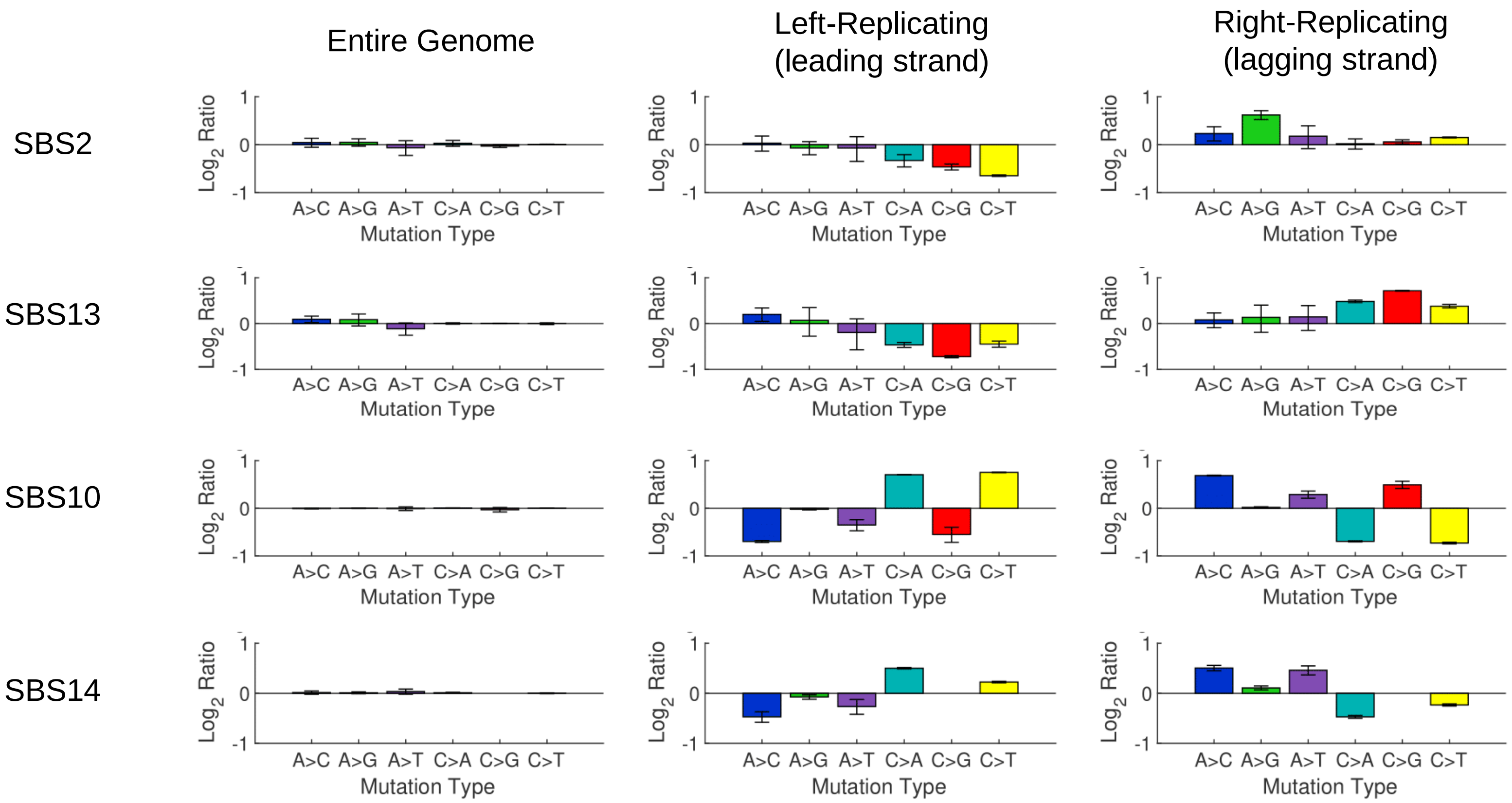

Supplementary Table 1. Tumor types. Tumor types as available in International Cancer Genome Consorium are shown with the number of samples. A total of 1925 cases are those with > 3000 mutations were used for the study.

Tumor type	Case no.
Biliary-AdenoCA	29
Bladder-TCC	23
Bone-Epith	2
Bone-Leiomyo	28
Bone-Osteosarc	22
Breast-AdenoCa	141
Breast-LobularCa	6
Cervix-AdenoCA	1
Cervix-SCC	17
CNS-GBM	40
CNS-Medullo	13
CNS-Oligo	4
ColoRect-AdenoCA	60
Eso-AdenoCa	97
Head-SCC	53
Kidney-ChRCC	4
Kidney-RCC	135
Liver-HCC	320
Lung-AdenoCA	33
Lung-SCC	48
Lymph-BNHL	92
Lymph-CLL	21
Lymph-NOS	2
Myeloid-AML	1
Myeloid-MPN	2
Ovary-AdenoCA	108
Panc-AdenoCA	222
Panc-Endocrine	27
Prost-AdenoCA	148
Skin-Melanoma	105
Stomach-AdenoCA	68
Thy-AdenoCA	3
Uterus-AdenoCA	50
Total	1925

Supplementary Table 2. Composition of mutation sigantures of ICGC data. For 30 mutation signatures (SBS1 to SBS30), the mutation abundance, top frequent tumor types and etioligies are listed.

Mutation signature	Abundance	Percent	Top three tumor types	Abundance of top three tumor types	Frequency (%) of top three tumor types	Etiology	Reference
SBS1	3662504	7.458	Panc-AdenoCA/ColoRect-AdenoCA/Eso-AdenoCa	811716/460144/448005	22.163/12.564/12.232	Age	[1]
SBS2	955498	1.946	Breast-AdenoCa/Head-SCC/Bladder-TCC	222058/138718/124128	23.24/14.518/12.991	APOBEC	[1]
SBS3	3137073	6.388	Ovary-AdenoCA/Breast-AdenoCa/Kidney-RCC	566675/537151/280333	18.064/17.123/8.936	*BRCA1/2* Mutations	[1]
SBS4	3088378	6.289	Lung-SCC/Lung-AdenoCA/Liver-HCC	1215386/1057574/365926	39.354/34.244/11.848	Smoking	[1]
SBS5	1379937	2.810	Liver-HCC/Lymph-BNHL/Prost-AdenoCA	255151/189048/154538	18.49/13.7/11.199		
SBS6	2060235	4.195	ColoRect-AdenoCA/Stomach-AdenoCA/Uterus-AdenoCA	627171/522685/414909	30.442/25.37/20.139	DNA MMR deficiency	[1]
SBS7	11898040	24.227	Skin-Melanoma/Liver-HCC/Lung-SCC	11691478/26409/22552	98.264/0.222/0.19	Ultraviolet light	[1]
SBS8	1059440	2.157	Liver-HCC/Kidney-RCC/Panc-AdenoCA	156400/119607/117177	14.763/11.29/11.06		
SBS9	580531	1.182	Lymph-BNHL/Eso-AdenoCa/Stomach-AdenoCA	261111/74833/54394	44.978/12.89/9.37	Immunoglobulin gene hypermutation	[1]
SBS10	6992054	14.237	ColoRect-AdenoCA/Uterus-AdenoCA/Lymph-BNHL	6685783/274167/4871	95.62/3.921/0.07	*POLE* mutations	[1]
SBS11	787086	1.603	Skin-Melanoma/CNS-GBM/Lymph-BNHL	392818/243675/31004	49.908/30.959/3.939	Temozolomide	[1]
SBS12	968164	1.971	Liver-HCC/Uterus-AdenoCA/Stomach-AdenoCA	628662/76713/54830	64.933/7.924/5.663		
SBS13	976612	1.989	Breast-AdenoCa/Lung-SCC/Head-SCC	211189/164718/152755	21.625/16.866/15.641	APOBEC	[1]
SBS14	1109425	2.259	Uterus-AdenoCA/ColoRect-AdenoCA/Stomach-AdenoCA	885611/142079/27634	79.826/12.807/2.491	POLE mutations	[2]
SBS15	1308722	2.665	ColoRect-AdenoCA/Uterus-AdenoCA/Stomach-AdenoCA	664576/413284/63319	50.781/31.579/4.838	DNA MMR deficiency	[3]
SBS16	2096772	4.270	Liver-HCC/Lung-SCC/Head-SCC	1539890/99394/82469	73.441/4.74/3.933		
SBS17	2152382	4.383	Eso-AdenoCa/Stomach-AdenoCA/Lymph-BNHL	1562646/325327/130790	72.601/15.115/6.077		
SBS18	534441	1.088	ColoRect-AdenoCA/Eso-AdenoCa/Stomach-AdenoCA	119882/86901/48761	22.431/16.26/9.124		
SBS19	412188	0.839	Liver-HCC/Lymph-BNHL/Lung-SCC	150102/43138/31642	36.416/10.466/7.677	DNA MMR deficiency	[3]
SBS20	223136	0.454	Stomach-AdenoCA/Liver-HCC/Uterus-AdenoCA	36177/30980/28762	16.213/13.884/12.89	DNA MMR deficiency	[3]
SBS21	409327	0.833	Stomach-AdenoCA/Biliary-AdenoCA/ColoRect-AdenoCA	153439/75713/56893	37.486/18.497/13.899		
SBS22	207223	0.422	Kidney-RCC/Liver-HCC/Ovary-AdenoCA	86604/83944/7489	41.793/40.509/3.614	aristolochic acid	[3]
SBS23	183076	0.373	Skin-Melanoma/Liver-HCC/ColoRect-AdenoCA	70955/40540/18118	38.757/22.144/9.896		
SBS24	224395	0.457	Liver-HCC/Lung-SCC/Kidney-RCC	88124/39508/14942	39.272/17.606/6.659	aflatoxin	[3]

SBS25	121089	0.247	Liver-HCC/Prost-AdenoCA/Lung-SCC	36068/21970/10917	29.786/18.144/9.016	DNA MMR deficiency	[3]
SBS26	297576	0.606	Uterus-AdenoCA/Panc-AdenoCA/Liver-HCC	90434/57607/50686	30.39/19.359/17.033	DNA MMR deficiency	[3]
SBS27	4093	0.008	Kidney-RCC/Liver-HCC/Bone-Osteosarc	1355/567/452	33.105/13.853/11.043		
SBS28	1493787	3.042	ColoRect-AdenoCA/Lymph-BNHL/Eso-AdenoCa	1338118/50409/21899	89.579/3.375/1.466		
SBS29	262906	0.535	Liver-HCC/Eso-AdenoCa/Lung-SCC	61453/44051/20926	23.375/16.755/7.959	Smoking	[3]
SBS30	278498	0.567	Liver-HCC/Kidney-RCC/Lymph-BNHL	61227/33168/19361	21.985/11.91/6.952		
Total	48864588	100.000					

reference

1. Alexandrov, Ludmil B., et al. "Signatures of mutational processes in human cancer." Nature 500.7463 (2013): 415
2. Tomkova, Marketa, et al. "Mutational signature distribution varies with DNA replication timing and strand asymmet
3. https://cancer.sanger.ac.uk/signatures/signatures_v2/

Top three tumor types : The top three cancer types which each mutation signature occurs frequently.
Frequency : #mutations of i th signature/#total mutation with signature

Supplementary Table 3. MutSig results. Significantly recurrent genes across mutation signatures were identified. Mutations with significant recurrence (MutSig Q < 0.1) are listed with the used co-variates (expression/Expr, replication time/Reptime and HiC)

	Gene	Expr*	Reptime*	HiC*	Nonsilent	Silent	*P* value	*Q* value (FDR)
SBS1	*CTU2*	802673	189	41	4	2	0	0
	DTX1	562320	387	44	9	0	0	0
	FBXW7	117295	501	51	11	1	0	0
	IDH1	260367	539	17	21	0	0	0
	KRAS	259193	512	16	132	2	0	0
	PIK3CA	401889	613	11	28	0	0	0
	PTEN	259678	300	34	25	0	0	0
	RHOA	1623187	201	33	6	0	0	0
	SMAD4	156084	422	41	28	0	0	0
	TP53	2069567	213	34	246	6	0	0
	CDKN2A	225405	357	-15	31	2	1.83E-08	3.14E-05
	APC	247811	399	28	18	2	6.27E-06	0.009854
SBS2	*C20orf195*	1262904	298	54	2	0	0	0
	TP53	2069567	213	34	13	0	0	0
	PIK3CA	401889	613	11	30	1	6.41E-11	4.03E-07
	SMAD4	156084	422	41	5	0	2.04E-05	0.096361
SBS3	*KRAS*	259193	512	16	39	0	0	0
	PTEN	259678	300	34	10	1	0	0
	VHL	825905	170	39	6	0	0	0
	TP53	2069567	213	34	89	3	1.18E-14	5.55E-11
SBS4	*C19orf57*	842601	185	32	3	1	0	0
	KRAS	259193	512	16	11	0	0	0
	SPRED2	545446	346	36	3	0	0	0
	TP53	2069567	213	34	45	0	0	0
	CDKN2A	225405	357	-15	6	0	2.59E-07	0.000975
SBS5	*CHTF8*	1179391	201	24	2	0	0	0
	KRAS	259193	512	16	14	0	0	0
	PIK3CA	401889	613	11	17	0	0	0
	SPOP	943930	348	28	12	0	0	0
	TP53	2069567	213	34	38	0	0	0
	PTEN	259678	300	34	5	0	2.89E-05	0.07785
SBS6	*KRAS*	259193	512	16	13	0	0	0
	TP53	2069567	213	34	34	0	0	0
SBS7	*CDKN2A*	225405	357	-15	20	1	0	0
	TP53	2069567	213	34	49	0	2.11E-14	1.99E-10
	BRAF	305191	616	25	45	1	7.63E-09	4.8E-05
	NRAS	600650	472	11	20	1	7.35E-08	0.000347
SBS8	*KRAS*	259193	512	16	30	1	0	0
	PEX13	635177	350	33	2	0	0	0
	TP53	2069567	213	34	23	0	0	0
SBS9	*B2M*	673122	428	36	8	0	0	0
SBS10	*TP53*	2069567	213	34	21	0	4.45E-07	0.007383
	VHL	825905	170	39	5	0	7.83E-07	0.007383
SBS12	*TP53*	2069567	213	34	11	0	2.62E-08	0.000494
SBS13	*TP53*	2069567	213	34	10	0	0	0
	PIK3CA	401889	613	11	15	1	7.68E-07	0.007242
SBS15	*SOCS1*	632841	308	37	4	0	0	0
	TP53	2069567	213	34	9	0	8.32E-05	0.785116
SBS16	*CTNNB1*	305811	448	8	46	1	0	0
	TP53	2069567	213	34	37	1	0	0

	PIK3CA	401889	613	11	14	2	4.55E-06	0.028623
	KRAS	259193	512	16	8	0	1.99E-05	0.093798
SBS17	*TP53*	2069567	213	34	21	0	1.59E-14	2.99E-10

* indicates co-variate used for MutSig
False discovery rate (Q values) < 0.1 were significant

Supplementary Table 4. Driver mutations with high level of positive selections. Genes with significantly elevated dNdSCV values for missense mutations (Qmissense < 0.2) are listed across mutation signatures. Wmis represents the dNdSCV values of missense mutations.

Mutation signature	Gene	Synonymous	Missense	Nonsense	Splicing	Wmis	Wtru	Pmissense	Ptrunc	Pallsubs_cv	Qmissense	Qtrunc_cv	Qallsubs_cv
SBS1	*TP53*	4	222	47	11	119.52	370.24	0.0E+00	0.0E+00	0.0E+00	0.0E+00	0.0E+00	0.0E+00
SBS1	*SMAD4*	1	26	9	2	33.002	139.32	0.0E+00	0.0E+00	0.0E+00	0.0E+00	0.0E+00	0.0E+00
SBS1	*PIK3CA*	1	34	1	0	26.464	3.3061	0.0E+00	3.4E-01	0.0E+00	0.0E+00	9.4E-01	0.0E+00
SBS1	*IDH1*	0	24	0	0	40.424	0	0.0E+00	7.2E-01	0.0E+00	0.0E+00	9.4E-01	0.0E+00
SBS1	*KRAS*	1	133	0	0	636.2	0	0.0E+00	8.1E-01	0.0E+00	0.0E+00	9.4E-01	0.0E+00
SBS1	*PTEN*	1	18	9	2	37.488	140.51	3.3E-16	0.0E+00	0.0E+00	1.1E-12	0.0E+00	0.0E+00
SBS1	*CDKN2A.p16INK4*	1	13	19	0	10.251	416.79	1.3E-06	0.0E+00	0.0E+00	2.1E-03	0.0E+00	0.0E+00
SBS1	*FBXW7*	0	15	6	2	22.281	58.588	1.1E-10	2.0E-10	1.4E-15	2.7E-07	4.4E-07	2.9E-12
SBS1	*CDKN2A.p14arf*	2	25	0	0	17.077	0	8.9E-14	6.5E-01	5.7E-13	2.6E-10	9.4E-01	9.5E-10
SBS1	*DDX3X*	0	14	0	0	14.262	0	5.2E-08	7.1E-01	2.9E-07	1.2E-04	9.4E-01	3.6E-04
SBS1	*SMARCA4*	3	30	1	0	6.4456	3.2407	1.6E-07	3.4E-01	1.1E-06	3.2E-04	9.4E-01	1.1E-03
SBS1	*CTNNB1*	1	14	0	0	10.002	0	9.0E-07	5.7E-01	3.6E-06	1.7E-03	9.4E-01	3.3E-03
SBS1	*IGLL5*	6	9	0	2	7.4389	24.381	5.5E-05	3.3E-03	9.6E-06	5.0E-02	8.7E-01	7.5E-03
SBS1	*C17orf80*	0	9	0	0	14.203	0	3.0E-06	7.0E-01	1.5E-05	4.7E-03	9.4E-01	1.1E-02
SBS1	*OR8B8*	0	9	0	0	12.556	0	7.7E-06	8.7E-01	4.4E-05	1.1E-02	9.4E-01	2.9E-02
SBS1	*GNAS*	10	23	0	0	4.1705	0	1.6E-05	4.5E-01	5.3E-05	1.9E-02	9.4E-01	3.4E-02
SBS1	*SPANXD*	1	5	0	0	24.602	0	1.1E-05	8.6E-01	6.4E-05	1.5E-02	9.4E-01	3.9E-02
SBS1	*S100A4*	0	4	0	0	40.043	0	1.2E-05	9.2E-01	7.0E-05	1.5E-02	9.5E-01	4.1E-02
SBS1	*MAP2K7*	1	8	1	0	9.431	28.253	7.8E-05	3.2E-02	7.9E-05	5.6E-02	9.4E-01	4.5E-02
SBS1	*EGFR*	5	22	1	0	4.6048	2.3888	3.0E-05	4.6E-01	1.6E-04	3.3E-02	9.4E-01	8.9E-02
SBS1	*EMILIN1*	1	13	0	0	7.1141	0	4.0E-05	6.6E-01	1.7E-04	4.2E-02	9.4E-01	9.0E-02
SBS1	*ZNF527*	1	10	0	0	8.2146	0	5.4E-05	6.0E-01	2.1E-04	5.0E-02	9.4E-01	1.1E-01
SBS1	*BRAF*	1	10	0	0	7.9515	0	6.9E-05	4.9E-01	2.1E-04	5.4E-02	9.4E-01	1.1E-01
SBS1	*PRRX1*	0	7	1	0	9.7345	19.165	2.1E-04	5.0E-02	2.9E-04	1.2E-01	9.4E-01	1.3E-01
SBS1	*KRTAP2-1*	0	3	0	0	51.954	0	5.5E-05	9.7E-01	2.9E-04	5.0E-02	9.7E-01	1.3E-01
SBS1	*ZNF595*	0	4	0	0	26.899	0	5.9E-05	8.5E-01	3.1E-04	5.2E-02	9.4E-01	1.3E-01
SBS1	*PPP1R35*	0	7	0	0	11.896	0	6.3E-05	8.0E-01	3.1E-04	5.3E-02	9.4E-01	1.3E-01
SBS1	*SCRN1*	0	8	0	0	10.307	0	6.8E-05	7.7E-01	3.3E-04	5.4E-02	9.4E-01	1.3E-01
SBS1	*PPP2R1A*	1	9	0	0	8.579	0	7.3E-05	7.0E-01	3.3E-04	5.5E-02	9.4E-01	1.3E-01
SBS1	*C19orf35*	0	9	0	0	8.807	0	1.0E-04	8.1E-01	4.8E-04	6.9E-02	9.4E-01	1.8E-01
SBS1	*PXDN*	4	22	2	0	3.8915	7.1156	3.1E-04	4.3E-02	5.6E-04	1.6E-01	9.4E-01	2.0E-01
SBS1	*GPR4*	0	11	0	0	7.376	0	1.3E-04	7.9E-01	6.1E-04	8.8E-02	9.4E-01	2.1E-01
SBS1	*SF3B1*	0	9	0	0	7.9208	0	2.1E-04	5.6E-01	6.9E-04	1.2E-01	9.4E-01	2.3E-01
SBS1	*PTPN11*	1	8	0	0	7.9035	0	2.4E-04	6.4E-01	9.5E-04	1.4E-01	9.4E-01	2.9E-01
SBS1	*RPL19*	0	5	0	0	14.052	0	2.1E-04	7.7E-01	9.6E-04	1.2E-01	9.4E-01	2.9E-01
SBS1	*KHDRBS3*	2	8	0	0	7.4768	0	2.5E-04	6.1E-01	9.6E-04	1.4E-01	9.4E-01	2.9E-01
SBS1	*KRTAP4-12*	0	4	0	0	19.47	0	2.1E-04	8.9E-01	1.0E-03	1.2E-01	9.4E-01	2.9E-01
SBS1	*DDX50*	1	7	0	0	8.5544	0	3.0E-04	5.6E-01	1.1E-03	1.6E-01	9.4E-01	2.9E-01
SBS1	*HCK*	0	9	0	0	7.5385	0	2.9E-04	6.3E-01	1.1E-03	1.6E-01	9.4E-01	2.9E-01
SBS1	*SLC18A2*	0	9	0	0	7.4501	0	3.1E-04	6.4E-01	1.2E-03	1.6E-01	9.4E-01	3.1E-01
SBS1	*KCNA6*	0	9	0	0	7.3929	0	3.3E-04	7.5E-01	1.4E-03	1.6E-01	9.4E-01	3.5E-01
SBS1	*CAPN8*	0	9	0	0	7.1674	0	4.0E-04	6.2E-01	1.4E-03	1.9E-01	9.4E-01	3.6E-01
SBS1	*CTSD*	1	6	0	0	9.6292	0	3.6E-04	8.1E-01	1.6E-03	1.7E-01	9.4E-01	4.0E-01
SBS2	*PIK3CA*	1	29	0	0	53.554	0	0.0E+00	6.5E-01	0.0E+00	0.0E+00	9.9E-01	0.0E+00
SBS2	*TP53*	0	8	4	0	41.346	184.17	1.7E-09	2.1E-08	3.1E-14	1.7E-05	4.1E-04	3.1E-10
SBS2	*C16orf92*	0	3	0	0	62.912	0	2.7E-05	8.7E-01	1.5E-04	1.8E-01	9.9E-01	6.2E-01
SBS3	*TP53*	4	85	13	19	102.26	356.17	0.0E+00	0.0E+00	0.0E+00	0.0E+00	0.0E+00	0.0E+00
SBS3	*KRAS*	1	38	0	0	124.88	0	0.0E+00	8.0E-01	0.0E+00	0.0E+00	9.3E-01	0.0E+00
SBS3	*VHL*	1	9	2	2	27.806	164.49	2.0E-09	2.2E-08	2.6E-14	1.0E-05	2.3E-04	1.7E-10
SBS3	*SOCS1*	1	10	0	0	33.279	0	6.5E-11	9.0E-01	5.3E-10	4.3E-07	9.3E-01	2.7E-06
SBS3	*PTEN*	0	10	0	1	17.8	15.505	3.3E-08	6.1E-02	7.9E-08	1.0E-04	9.3E-01	3.2E-04
SBS3	*BCL2*	1	9	0	0	20.594	0	2.6E-08	8.4E-01	1.8E-07	1.0E-04	9.3E-01	4.6E-04
SBS3	*PIK3CA*	1	15	0	0	10.596	0	3.6E-08	5.9E-01	1.8E-07	1.0E-04	9.3E-01	4.6E-04
SBS3	*HIST1H1E*	1	9	1	0	13.415	38.865	8.9E-07	2.2E-02	7.4E-07	2.0E-03	9.3E-01	1.5E-03
SBS3	*ACTB*	1	10	0	0	12.56	0	5.2E-07	7.4E-01	3.0E-06	1.3E-03	9.3E-01	5.5E-03
SBS3	*IGLL5*	1	7	0	1	15.121	28.218	4.4E-06	3.1E-02	4.3E-06	8.9E-03	9.3E-01	7.1E-03
SBS3	*TNFRSF18*	0	6	0	0	19.105	0	5.5E-06	8.3E-01	3.1E-05	1.0E-02	9.3E-01	4.8E-02

SBS3	*ACTG1*	0	9	0	0	10.455	0	8.3E-06	7.1E-01	4.3E-05	1.4E-02	9.3E-01	6.1E-02
SBS3	*DUSP2*	1	7	0	0	12.631	0	1.4E-05	7.9E-01	7.5E-05	2.2E-02	9.3E-01	9.2E-02
SBS3	*CTNNB1*	1	10	0	0	8.29	0	1.7E-05	6.5E-01	8.0E-05	2.5E-02	9.3E-01	9.2E-02
SBS3	*LCE2B*	0	4	0	0	31.087	0	2.1E-05	9.2E-01	1.2E-04	2.9E-02	9.4E-01	1.3E-01
SBS3	*HIST1H2AC*	0	5	0	0	15.869	0	6.6E-05	8.7E-01	3.4E-04	8.3E-02	9.3E-01	3.4E-01
SBS3	*HIST1H3C*	0	5	0	0	15.584	0	7.2E-05	8.7E-01	3.7E-04	8.5E-02	9.3E-01	3.4E-01
SBS3	*BRAF*	0	8	1	0	7.718	8.0448	1.6E-04	1.3E-01	4.0E-04	1.7E-01	9.3E-01	3.5E-01
SBS3	*HELZ2*	1	13	0	0	5.158	0	1.9E-04	5.8E-01	6.9E-04	1.9E-01	9.3E-01	5.8E-01
SBS3	*HIST1H2BC*	0	5	0	0	13.28	0	1.5E-04	8.4E-01	7.5E-04	1.7E-01	9.3E-01	5.8E-01
SBS4	*TP53*	1	41	4	5	91.555	149.26	0.0E+00	2.1E-14	0.0E+00	0.0E+00	4.1E-10	0.0E+00
SBS4	*KRAS*	0	11	0	0	103.28	0	4.0E-14	8.5E-01	3.5E-13	4.0E-10	9.4E-01	3.5E-09
SBS4	*KEAP1*	0	7	0	1	21.476	30.7	9.1E-06	3.2E-02	1.5E-05	4.6E-02	9.4E-01	3.8E-02
SBS4	*HRAS*	0	4	0	0	61.349	0	5.3E-06	8.9E-01	3.1E-05	3.5E-02	9.4E-01	6.8E-02
SBS4	*BCAT1*	0	6	0	0	22.277	0	2.2E-05	7.5E-01	1.0E-04	8.8E-02	9.4E-01	1.7E-01
SBS4	*NFE2L2*	0	6	0	0	20.428	0	3.5E-05	7.8E-01	1.7E-04	1.2E-01	9.4E-01	2.6E-01
SBS5	*TP53*	0	36	1	3	293.39	318.09	0.0E+00	6.9E-09	0.0E+00	0.0E+00	1.4E-04	0.0E+00
SBS5	*IGLL5*	8	21	1	0	43.581	24.828	0.0E+00	3.6E-02	0.0E+00	0.0E+00	9.8E-01	0.0E+00
SBS5	*KRAS*	1	13	0	0	104.84	0	0.0E+00	8.8E-01	5.6E-16	0.0E+00	9.8E-01	3.7E-12
SBS5	*PIK3CA*	0	19	0	0	46.376	0	2.0E-14	7.7E-01	1.4E-13	1.0E-10	9.8E-01	7.2E-10
SBS5	*SPOP*	0	12	0	0	81.561	0	9.3E-14	8.6E-01	8.1E-13	3.8E-10	9.8E-01	3.3E-09
SBS5	*PIM1*	1	11	1	1	41.963	81.267	6.6E-11	3.7E-04	9.9E-12	2.2E-07	5.5E-01	3.3E-08
SBS5	*BCL2*	5	11	0	0	27.554	0	1.3E-10	8.4E-01	1.0E-09	3.7E-07	9.8E-01	2.9E-06
SBS5	*MYC*	3	11	0	0	17.356	0	4.5E-08	7.6E-01	2.8E-07	1.1E-04	9.8E-01	6.2E-04
SBS5	*SGK1*	3	7	0	3	11.597	45.224	5.4E-05	7.6E-05	6.6E-07	5.2E-02	1.9E-01	1.3E-03
SBS5	*PTEN*	0	6	0	1	31.884	54.293	3.2E-06	1.7E-02	3.2E-06	5.4E-03	9.8E-01	5.5E-03
SBS5	*SLC25A32*	0	6	0	0	32.812	0	2.7E-06	8.5E-01	1.6E-05	5.4E-03	9.8E-01	2.0E-02
SBS5	*SOCS1*	2	6	0	0	25.351	0	2.8E-06	9.0E-01	1.7E-05	5.4E-03	9.8E-01	2.0E-02
SBS5	*ASNA1*	0	5	0	0	44.044	0	3.0E-06	8.9E-01	1.8E-05	5.4E-03	9.8E-01	2.0E-02
SBS5	*NUDT13*	0	5	0	0	38.284	0	6.0E-06	8.6E-01	3.4E-05	9.2E-03	9.8E-01	3.5E-02
SBS5	*BRAF*	0	7	0	0	22.784	0	6.8E-06	7.9E-01	3.5E-05	9.7E-03	9.8E-01	3.5E-02
SBS5	*EPSTI1*	0	6	0	0	26.485	0	9.1E-06	8.0E-01	4.7E-05	1.2E-02	9.8E-01	4.5E-02
SBS5	*HIST1H1E*	1	7	0	0	18.083	0	1.0E-05	8.6E-01	5.7E-05	1.2E-02	9.8E-01	5.2E-02
SBS5	*TMSB4X*	0	3	0	0	108.46	0	1.0E-05	9.3E-01	5.9E-05	1.2E-02	9.8E-01	5.2E-02
SBS5	*PAX5*	0	5	0	0	32.889	0	1.2E-05	8.6E-01	6.8E-05	1.4E-02	9.8E-01	5.7E-02
SBS5	*ALDH9A1*	1	6	0	0	19.793	0	1.9E-05	8.1E-01	1.0E-04	2.0E-02	9.8E-01	7.8E-02
SBS5	*TRIM38*	0	4	1	0	24.744	67.031	1.9E-04	1.4E-02	1.0E-04	1.3E-01	9.8E-01	7.8E-02
SBS5	*RAPGEFL1*	0	5	0	0	28.636	0	2.4E-05	8.4E-01	1.3E-04	2.4E-02	9.8E-01	9.1E-02
SBS5	*NOL9*	1	5	1	0	15.173	34.505	2.4E-04	2.7E-02	2.0E-04	1.5E-01	9.8E-01	1.4E-01
SBS5	*SNCB*	0	3	0	0	62.276	0	5.7E-05	9.1E-01	3.0E-04	5.2E-02	9.8E-01	1.8E-01
SBS5	*EZH2*	0	5	0	1	16.925	29.652	2.7E-04	3.4E-02	3.2E-04	1.6E-01	9.8E-01	1.9E-01
SBS5	*TAOK3*	0	6	0	0	18.007	0	7.2E-05	7.8E-01	3.4E-04	6.0E-02	9.8E-01	1.9E-01
SBS5	*CTNNB1*	0	6	0	0	17.882	0	7.5E-05	8.1E-01	3.6E-04	6.0E-02	9.8E-01	2.0E-01
SBS5	*IL20RB*	0	4	0	0	31.748	0	7.4E-05	8.7E-01	3.7E-04	6.0E-02	9.8E-01	2.0E-01
SBS5	*RUNDC1*	0	5	0	0	22.008	0	8.2E-05	8.6E-01	4.1E-04	6.3E-02	9.8E-01	2.1E-01
SBS5	*SF3B1*	1	8	0	0	10.599	0	1.3E-04	7.0E-01	5.2E-04	9.1E-02	9.8E-01	2.4E-01
SBS5	*POLR1D*	0	4	0	0	28.823	0	1.1E-04	8.9E-01	5.4E-04	8.0E-02	9.8E-01	2.5E-01
SBS5	*NAA30*	0	4	0	0	23.932	0	2.2E-04	8.7E-01	1.0E-03	1.5E-01	9.8E-01	4.3E-01
SBS5	*NRAS*	0	3	0	0	39.461	0	2.3E-04	9.0E-01	1.1E-03	1.5E-01	9.8E-01	4.4E-01
SBS5	*MYD88*	0	4	0	0	22.478	0	2.8E-04	8.6E-01	1.3E-03	1.6E-01	9.8E-01	5.1E-01
SBS5	*SRSF7*	0	4	0	0	22.094	0	3.0E-04	8.2E-01	1.3E-03	1.7E-01	9.8E-01	5.1E-01
SBS5	*FOXA1*	0	4	0	0	21.75	0	3.1E-04	8.9E-01	1.5E-03	1.8E-01	9.8E-01	5.4E-01
SBS5	*IRF4*	0	4	0	0	21.203	0	3.5E-04	8.5E-01	1.6E-03	1.9E-01	9.8E-01	5.7E-01
SBS6	*TP53*	0	23	6	2	24.466	113.32	0.0E+00	1.2E-13	0.0E+00	0.0E+00	2.5E-09	0.0E+00
SBS6	*KRAS*	1	12	0	0	77.106	0	0.0E+00	8.5E-01	3.3E-16	0.0E+00	9.6E-01	3.3E-12
SBS6	*PIK3CA*	0	17	0	0	19.314	0	5.2E-13	5.4E-01	3.1E-12	3.5E-09	9.6E-01	2.1E-08
SBS6	*HIST1H1B*	1	8	0	0	19.274	0	1.1E-07	9.7E-01	7.8E-07	5.7E-04	9.7E-01	3.1E-03
SBS6	*IGLL5*	10	7	1	0	9.0245	22.787	4.4E-05	3.8E-02	3.5E-05	1.5E-01	9.6E-01	8.9E-02
SBS6	*ZNF800*	0	6	0	0	11.704	0	5.8E-05	7.0E-01	2.7E-04	1.5E-01	9.6E-01	3.9E-01
SBS6	*HIST1H1E*	1	6	0	0	11.631	0	5.5E-05	9.5E-01	2.9E-04	1.5E-01	9.6E-01	3.9E-01
SBS6	*PI15*	0	6	0	0	11.404	0	6.7E-05	7.9E-01	3.3E-04	1.5E-01	9.6E-01	4.1E-01
SBS6	*SOX11*	1	7	0	0	9.2272	0	6.8E-05	8.9E-01	3.5E-04	1.5E-01	9.6E-01	4.1E-01
SBS6	*HHIP*	0	7	0	0	8.8668	0	9.6E-05	7.4E-01	4.6E-04	1.9E-01	9.6E-01	4.7E-01
SBS7	*BRAF*	2	44	0	0	22.713	0	0.0E+00	4.8E-01	0.0E+00	0.0E+00	9.3E-01	0.0E+00
SBS7	*NRAS*	0	21	0	0	106.42	0	0.0E+00	8.5E-01	0.0E+00	0.0E+00	9.3E-01	0.0E+00

SBS7	*CDKN2A.p14arf*	2	12	0	3	16.153	67.198	4.0E-08	2.3E-05	5.0E-10	2.7E-04	1.2E-01	2.5E-06
SBS7	*TP53*	1	13	4	1	10.916	47.824	2.5E-06	6.8E-07	3.9E-09	1.2E-02	6.8E-03	1.6E-05
SBS7	*SERPINB3*	1	14	1	0	9.2604	5.6872	7.4E-06	1.9E-01	3.8E-05	3.0E-02	9.3E-01	1.1E-01
SBS7	*EMB*	1	8	1	0	11.749	9.5455	3.7E-05	1.1E-01	1.1E-04	1.2E-01	9.3E-01	2.8E-01
SBS8	*TP53*	0	21	1	5	155.11	329.52	0.0E+00	1.1E-13	0.0E+00	0.0E+00	2.2E-09	0.0E+00
SBS8	*KRAS*	0	31	0	0	427.75	0	0.0E+00	8.8E-01	0.0E+00	0.0E+00	9.8E-01	0.0E+00
SBS8	*VHL*	0	5	1	2	70.301	457.25	2.6E-08	4.0E-08	1.3E-13	1.3E-04	4.1E-04	8.4E-10
SBS8	*MYC*	1	8	0	0	38.354	0	3.9E-10	8.5E-01	3.1E-09	2.6E-06	9.8E-01	1.6E-05
SBS8	*CD80*	0	5	0	0	41.805	0	3.5E-07	8.7E-01	2.3E-06	1.4E-03	9.8E-01	9.1E-03
SBS8	*H3F3B*	0	4	0	0	68.007	0	6.4E-07	9.1E-01	4.1E-06	2.1E-03	9.8E-01	1.0E-02
SBS8	*PASD1*	1	7	0	0	17.162	0	9.7E-07	7.4E-01	5.7E-06	2.8E-03	9.8E-01	1.3E-02
SBS8	*IGLL5*	1	4	0	0	48.516	0	2.4E-06	9.0E-01	1.5E-05	6.1E-03	9.8E-01	3.0E-02
SBS8	*RRAGC*	0	4	0	0	31.029	0	1.5E-05	8.7E-01	8.5E-05	3.4E-02	9.8E-01	1.6E-01
SBS8	*BRAF*	0	5	0	0	17.203	0	2.8E-05	7.7E-01	1.5E-04	5.6E-02	9.8E-01	2.1E-01
SBS8	*EEF1A1*	0	5	0	0	15.953	0	4.0E-05	8.0E-01	2.1E-04	7.4E-02	9.8E-01	2.8E-01
SBS8	*HIST1H1C*	0	4	0	0	23.502	0	4.6E-05	8.9E-01	2.5E-04	7.8E-02	9.8E-01	3.1E-01
SBS8	*CTNNB1*	0	5	0	0	15.177	0	5.1E-05	7.9E-01	2.6E-04	7.9E-02	9.8E-01	3.1E-01
SBS8	*FCRL3*	0	5	0	0	14.617	0	6.2E-05	7.6E-01	3.1E-04	8.8E-02	9.8E-01	3.4E-01
SBS8	*LRRN4CL*	0	3	0	0	41.334	0	6.8E-05	9.3E-01	3.6E-04	9.1E-02	9.8E-01	3.8E-01
SBS8	*NELFA*	0	4	0	0	20.28	0	8.4E-05	8.4E-01	4.3E-04	1.0E-01	9.8E-01	4.2E-01
SBS8	*OR6F1*	0	4	0	0	19.403	0	1.0E-04	8.8E-01	5.1E-04	1.1E-01	9.8E-01	4.3E-01
SBS8	*MEF2B*	0	3	0	0	36.351	0	1.0E-04	8.9E-01	5.1E-04	1.1E-01	9.8E-01	4.3E-01
SBS8	*NUDT21*	0	3	0	0	35.075	0	1.1E-04	8.7E-01	5.7E-04	1.2E-01	9.8E-01	4.5E-01
SBS8	*MEF2BNB-MEF2E*	0	3	0	0	34.74	0	1.2E-04	8.9E-01	5.9E-04	1.2E-01	9.8E-01	4.5E-01
SBS8	*ZNF107*	0	5	0	0	11.59	0	1.9E-04	7.4E-01	8.6E-04	1.8E-01	9.8E-01	6.1E-01
SBS8	*ATF1*	0	3	0	0	28.318	0	2.2E-04	8.6E-01	1.0E-03	2.0E-01	9.8E-01	7.2E-01
SBS9	*B2M*	0	7	3	0	156.9	644.66	3.0E-10	5.1E-08	2.0E-13	2.0E-06	5.1E-04	4.1E-09
SBS9	*IGLL5*	2	9	0	1	100.22	149.29	2.2E-11	6.0E-03	2.1E-11	4.5E-07	9.9E-01	1.4E-07
SBS9	*TP53*	0	6	1	0	254.99	373.62	1.4E-10	1.7E-03	6.8E-11	1.4E-06	9.9E-01	3.4E-07
SBS9	*SOCS1*	0	4	0	0	172.38	0	1.8E-07	9.8E-01	1.2E-06	9.0E-04	9.9E-01	4.9E-03
SBS9	*SPOP*	0	4	0	0	91.15	0	2.2E-06	9.2E-01	1.3E-05	9.0E-03	9.9E-01	3.9E-02
SBS9	*RHOA*	0	3	0	0	165.15	0	3.4E-06	9.6E-01	2.1E-05	1.2E-02	9.9E-01	5.2E-02
SBS9	*ACTB*	0	4	0	0	74.498	0	4.9E-06	9.4E-01	2.9E-05	1.4E-02	9.9E-01	6.5E-02
SBS9	*EZH2*	0	5	0	0	39.175	0	1.5E-05	8.6E-01	7.9E-05	3.8E-02	9.9E-01	1.4E-01
SBS9	*GEMIN2*	0	3	0	0	89.862	0	2.2E-05	9.1E-01	1.2E-04	4.8E-02	9.9E-01	2.0E-01
SBS9	*BCR*	0	4	0	0	36.902	0	6.9E-05	9.0E-01	3.5E-04	1.3E-01	9.9E-01	3.6E-01
SBS9	*MYD88*	0	3	0	0	61.063	0	6.8E-05	9.3E-01	3.6E-04	1.3E-01	9.9E-01	3.6E-01
SBS9	*P2RY8*	0	3	0	0	59.19	0	7.5E-05	9.5E-01	3.9E-04	1.3E-01	9.9E-01	3.6E-01
SBS10	*TP53*	0	4	1	0	66.72	110.26	8.4E-07	6.8E-03	1.9E-07	9.0E-03	9.9E-01	3.9E-03
SBS10	*PIK3CA*	0	6	0	0	24.886	0	9.0E-07	7.2E-01	5.2E-06	9.0E-03	9.9E-01	5.2E-02
SBS11	*BRAF*	0	10	0	0	62.794	0	4.3E-11	8.2E-01	3.2E-10	8.5E-07	9.9E-01	6.4E-06
SBS12	*TP53*	0	14	0	1	127.1	113.37	0.0E+00	6.5E-03	0.0E+00	0.0E+00	9.9E-01	0.0E+00
SBS12	*CTNNB1*	0	10	0	0	33.275	0	2.7E-11	8.3E-01	2.2E-10	2.7E-07	9.9E-01	2.2E-06
SBS12	*PIK3CA*	1	7	0	0	17.056	0	1.2E-06	8.1E-01	7.0E-06	7.7E-03	9.9E-01	4.7E-02
SBS13	*PIK3CA*	0	17	0	0	30.875	0	0.0E+00	7.4E-01	3.3E-16	0.0E+00	9.8E-01	6.7E-12
SBS13	*TP53*	0	7	4	0	26.16	119.46	5.0E-08	5.9E-08	5.1E-13	5.0E-04	1.2E-03	5.1E-09
SBS13	*FGFR3*	0	6	0	0	18.819	0	2.7E-06	8.0E-01	1.6E-05	1.8E-02	9.8E-01	6.8E-02
SBS13	*DNTTIP1*	0	5	0	0	23.649	0	5.3E-06	7.8E-01	3.0E-05	2.6E-02	9.8E-01	1.0E-01
SBS13	*NFE2L2*	0	6	0	0	11.902	0	3.6E-05	7.2E-01	1.8E-04	1.5E-01	9.8E-01	4.1E-01
SBS15	*KRAS*	0	7	0	0	153.41	0	1.1E-11	9.4E-01	9.4E-11	2.2E-07	9.9E-01	1.9E-06
SBS15	*TP53*	0	6	0	2	41.457	182.6	4.7E-07	7.5E-05	7.4E-09	4.7E-03	5.0E-01	7.4E-05
SBS15	*PIM1*	2	5	1	0	22.013	120.17	2.4E-05	6.5E-03	6.2E-06	9.8E-02	9.9E-01	4.1E-02
SBS15	*SRP72*	0	5	0	0	31.744	0	1.1E-05	8.7E-01	5.9E-05	7.1E-02	9.9E-01	2.4E-01
SBS15	*TOP2B*	1	7	0	0	15.267	0	2.2E-05	7.7E-01	1.1E-04	9.8E-02	9.9E-01	3.4E-01
SBS15	*BTG2*	0	3	0	0	66.216	0	4.0E-05	9.7E-01	2.2E-04	1.3E-01	9.9E-01	5.4E-01
SBS15	*SLC30A2*	0	4	0	0	32.954	0	5.0E-05	9.1E-01	2.7E-04	1.4E-01	9.9E-01	5.9E-01
SBS15	*CDRT15L2*	0	3	0	0	56.405	0	6.5E-05	9.6E-01	3.4E-04	1.6E-01	9.9E-01	6.3E-01
SBS16	*TP53*	0	36	9	5	128.69	526.75	0.0E+00	0.0E+00	0.0E+00	0.0E+00	0.0E+00	0.0E+00
SBS16	*CTNNB1*	3	45	1	0	45.936	13.061	0.0E+00	7.1E-02	0.0E+00	0.0E+00	9.7E-01	0.0E+00
SBS16	*KRAS*	0	8	0	0	37.257	0	4.0E-10	8.6E-01	3.1E-09	2.7E-06	9.7E-01	1.6E-05
SBS16	*NFE2L2*	0	10	1	0	17.351	28.001	6.3E-09	3.1E-02	6.8E-09	3.1E-05	9.7E-01	2.7E-05
SBS16	*PIK3CA*	0	12	0	0	11.029	0	4.2E-08	6.8E-01	2.6E-07	1.7E-04	9.7E-01	8.6E-04
SBS16	*CAMK1*	0	5	1	0	20.204	39.149	1.1E-05	2.1E-02	5.7E-06	3.2E-02	9.7E-01	1.4E-02
SBS16	*SF3B1*	1	10	0	0	7.4962	0	1.0E-05	6.4E-01	5.1E-05	3.2E-02	9.7E-01	9.3E-02

SBS16	*RHOA*	0	4	0	0	28.807	0	1.9E-05	8.9E-01	1.0E-04	4.7E-02	9.7E-01	1.7E-01
SBS17	*TP53*	0	14	4	2	182.46	1414.9	6.3E-15	3.4E-14	0.0E+00	1.3E-10	6.8E-10	0.0E+00
SBS17	*IVL*	0	10	0	0	35.087	0	5.0E-06	9.0E-01	2.7E-05	5.0E-02	9.9E-01	1.8E-01

Supplementary Table 5. Evolutionarily distinct mutational processes in individual cancer genomes. Pairs of mutation signatures with significant (FDR < 0.05, KS test) discordant CCF value distributions are shown. For individual mutaiton signature pairs, tumor type (with ICGC reference ID), mutation signatures, mutation numbers, and KS test-related statistics (D value and P value) are listed.

Tumor type	ICGC ref. ID	Signature (#1 in pairs)	Signature (#2 in pairs)	Signature 1 (mutation no	Signature 2 (mutation no)	*D* value (KS test)	*P* value (KS test)	FDR
Ovary-AdenoCA	0009b464-b376-4fbc-8a56-da53	SBS1	SBS3	813	8901	0.1436121	9.03E-14	2.19E-12
Ovary-AdenoCA	0009b464-b376-4fbc-8a56-da53	SBS1	SBS4	813	252	0.1649241	5.70E-05	0.0005106
Ovary-AdenoCA	0009b464-b376-4fbc-8a56-da53	SBS2	SBS3	1045	8901	0.1067871	1.09E-09	1.86E-08
Ovary-AdenoCA	0009b464-b376-4fbc-8a56-da53	SBS2	SBS4	1045	252	0.1363105	0.0010574	0.0069149
Lymph-CLL	5f53bcda-2fd3-4574-9cc2-5efe3	SBS1	SBS3	427	194	0.1662281	0.0012574	0.0080149
Lymph-CLL	5f53bcda-2fd3-4574-9cc2-5efe3	SBS1	SBS5	427	2412	0.1346468	3.87E-06	4.29E-05
Eso-AdenoCa	5fd632ea-085e-4e9b-8dcc-ec94	SBS9	SBS17	1536	8728	0.0481126	0.00473	0.0246002
Panc-AdenoCA	09cb8bc5-13ac-44ac-9b7d-6de1	SBS1	SBS6	5710	1335	0.1229665	1.23E-14	3.22E-13
Panc-AdenoCA	09cb8bc5-13ac-44ac-9b7d-6de1	SBS1	SBS13	5710	567	0.083272	0.0015648	0.0096715
Panc-AdenoCA	09cb8bc5-13ac-44ac-9b7d-6de1	SBS6	SBS13	1335	567	0.0917781	0.0024509	0.0141697
Panc-Endocrine	606d7f5e-ff98-4245-a9ac-281f5	SBS2	SBS7	1173	413	0.1599281	3.27E-07	4.25E-06
Panc-Endocrine	606d7f5e-ff98-4245-a9ac-281f5	SBS2	SBS13	1173	1436	0.0705444	0.0032382	0.017943
Panc-Endocrine	606d7f5e-ff98-4245-a9ac-281f5	SBS2	SBS16	1173	916	0.2333527	0	0
Panc-Endocrine	606d7f5e-ff98-4245-a9ac-281f5	SBS7	SBS13	413	1436	0.0924565	0.008308	0.0392064
Panc-Endocrine	606d7f5e-ff98-4245-a9ac-281f5	SBS13	SBS16	1436	916	0.1709929	1.25E-14	3.26E-13
Biliary-AdenoCA	60f81dba-c623-11e3-bf01-24c6	SBS2	SBS5	794	728	0.1298994	5.43E-06	5.84E-05
Biliary-AdenoCA	60f81dba-c623-11e3-bf01-24c6	SBS2	SBS8	794	2577	0.0750946	0.0021278	0.0125566
Biliary-AdenoCA	60f81dba-c623-11e3-bf01-24c6	SBS2	SBS12	794	672	0.1162776	0.0001064	0.0008899
Liver-HCC	6172ca02-c622-11e3-bf01-24c6	SBS4	SBS16	2067	1532	0.0567554	0.0069067	0.0335798
Liver-HCC	6172ca02-c622-11e3-bf01-24c6	SBS8	SBS16	889	1532	0.0752973	0.0033935	0.0186392
Prost-AdenoCA	61a48c69-4f7d-4dc6-aff7-88a6c	SBS1	SBS16	1600	3060	0.1689297	0	0
Prost-AdenoCA	61d941f1-87df-43af-8a29-b6809	SBS1	SBS8	1337	348	0.1102077	0.0024433	0.0141507
Prost-AdenoCA	6218f366-d5d2-4289-a95e-eeed	SBS1	SBS3	2328	244	0.1196975	0.0035694	0.0195194
Prost-AdenoCA	6218f366-d5d2-4289-a95e-eeed	SBS1	SBS9	2328	652	0.1301125	6.48E-08	9.14E-07
Prost-AdenoCA	6218f366-d5d2-4289-a95e-eeed	SBS1	SBS16	2328	1413	0.0873863	2.94E-06	3.33E-05
Prost-AdenoCA	6218f366-d5d2-4289-a95e-eeed	SBS4	SBS9	2239	652	0.1035355	3.98E-05	0.0003686
Prost-AdenoCA	6218f366-d5d2-4289-a95e-eeed	SBS4	SBS16	2239	1413	0.0665049	0.0009397	0.0061992
Lymph-BNHL	626c9788-d70c-449b-b9dc-5e3f	SBS5	SBS6	3773	1032	0.0660474	0.0017006	0.0103877
Lymph-BNHL	626c9788-d70c-449b-b9dc-5e3f	SBS6	SBS11	1032	2286	0.06757	0.0030292	0.0169502
Liver-HCC	63fef3fe-c622-11e3-bf01-24c65	SBS4	SBS16	4851	27185	0.0710508	0	0
Eso-AdenoCa	64623aaa-a274-4baa-8b71-958	SBS1	SBS3	2342	23756	0.0843039	1.38E-13	3.28E-12
Eso-AdenoCa	64623aaa-a274-4baa-8b71-958	SBS1	SBS13	2342	9261	0.3435987	0	0
Eso-AdenoCa	64623aaa-a274-4baa-8b71-958	SBS1	SBS14	2342	1717	0.0676169	0.0002327	0.0018025
Eso-AdenoCa	64623aaa-a274-4baa-8b71-958	SBS1	SBS16	2342	699	0.0933883	0.0001671	0.0013359
Eso-AdenoCa	64623aaa-a274-4baa-8b71-958	SBS1	SBS17	2342	16433	0.0447075	0.0005524	0.0039164
Eso-AdenoCa	64623aaa-a274-4baa-8b71-958	SBS3	SBS13	23756	9261	0.269368	0	0
Eso-AdenoCa	64623aaa-a274-4baa-8b71-958	SBS3	SBS17	23756	16433	0.1121823	0	0
Eso-AdenoCa	64623aaa-a274-4baa-8b71-958	SBS13	SBS14	9261	1717	0.2966002	0	0
Eso-AdenoCa	64623aaa-a274-4baa-8b71-958	SBS13	SBS16	9261	699	0.3101673	0	0
Eso-AdenoCa	64623aaa-a274-4baa-8b71-958	SBS13	SBS17	9261	16433	0.3765405	0	0
Eso-AdenoCa	64623aaa-a274-4baa-8b71-958	SBS14	SBS17	1717	16433	0.0924649	5.71E-12	1.16E-10
Eso-AdenoCa	64623aaa-a274-4baa-8b71-958	SBS16	SBS17	699	16433	0.0889586	4.92E-05	0.000446
Eso-AdenoCa	6495e68e-1434-45d3-a03d-f286	SBS1	SBS5	9660	528	0.0945535	0.000259	0.0019869
Eso-AdenoCa	6495e68e-1434-45d3-a03d-f286	SBS1	SBS17	9660	5412	0.1133203	0	0
Eso-AdenoCa	6495e68e-1434-45d3-a03d-f286	SBS5	SBS17	528	5412	0.2029287	0	0
Stomach-AdenoCA	65be412d-46c3-4cbf-9b46-b353	SBS1	SBS5	4601	5043	0.0495794	1.46E-05	0.0001456
Panc-AdenoCA	65d2dbc3-a163-4696-b246-47a	SBS1	SBS13	4327	638	0.0863485	0.0005013	0.003603
Panc-AdenoCA	65d2dbc3-a163-4696-b246-47a	SBS1	SBS17	4327	482	0.0937585	0.0009764	0.0064273
Panc-AdenoCA	65d2dbc3-a163-4696-b246-47a	SBS6	SBS13	792	638	0.0886015	0.0077919	0.0372146
Panc-AdenoCA	65d2dbc3-a163-4696-b246-47a	SBS6	SBS17	792	482	0.0960958	0.0079001	0.0376213
Ovary-AdenoCA	669f0e01-28f6-4ed8-bdb5-73f84	SBS1	SBS3	439	7392	0.1479353	2.65E-08	3.91E-07
Ovary-AdenoCA	669f0e01-28f6-4ed8-bdb5-73f84	SBS1	SBS13	439	751	0.2436296	1.04E-14	2.75E-13
Ovary-AdenoCA	669f0e01-28f6-4ed8-bdb5-73f84	SBS3	SBS13	7392	751	0.109318	1.68E-07	2.29E-06
Panc-AdenoCA	0b29c893-03bf-4131-b192-c14a	SBS1	SBS3	11181	919	0.0875202	4.48E-06	4.88E-05
Panc-AdenoCA	0b29c893-03bf-4131-b192-c14a	SBS1	SBS13	11181	660	0.068507	0.0057613	0.0289079

Panc-AdenoCA	0b29c893-03bf-4131-b192-c14a	SBS1	SBS17	11181	6804	0.2431854	0	0
Panc-AdenoCA	0b29c893-03bf-4131-b192-c14a	SBS3	SBS13	919	660	0.1473225	1.15E-07	1.59E-06
Panc-AdenoCA	0b29c893-03bf-4131-b192-c14a	SBS3	SBS17	919	6804	0.1776762	0	0
Panc-AdenoCA	0b29c893-03bf-4131-b192-c14a	SBS13	SBS17	660	6804	0.3095639	0	0
Liver-HCC	67189cd8-c623-11e3-bf01-24c6	SBS5	SBS11	4626	1213	0.0644198	0.0006871	0.0047008
Lymph-CLL	0b6cd7df-6970-4d60-b7b5-8500	SBS1	SBS9	513	1871	0.2090906	9.99E-16	2.89E-14
Stomach-AdenoCA	68509ede-3dcf-4a6e-9af0-4a9b	SBS1	SBS17	3498	6521	0.0404201	0.0011753	0.0075625
Stomach-AdenoCA	68509ede-3dcf-4a6e-9af0-4a9b	SBS2	SBS17	1521	6521	0.0535495	0.0016949	0.0103628
Stomach-AdenoCA	68509ede-3dcf-4a6e-9af0-4a9b	SBS15	SBS17	682	6521	0.0682919	0.006308	0.0310544
Liver-HCC	690e481c-c623-11e3-bf01-24c6	SBS3	SBS4	7666	3177	0.0431454	0.0004669	0.003383
Liver-HCC	690e481c-c623-11e3-bf01-24c6	SBS3	SBS16	7666	6919	0.035823	0.0001768	0.0014005
Eso-AdenoCa	6a0d0c42-1884-4c6a-936a-678	SBS8	SBS12	11403	879	0.0654872	0.0018242	0.0110033
Eso-AdenoCa	6a0d0c42-1884-4c6a-936a-678	SBS8	SBS17	11403	23494	0.0727844	0	0
Liver-HCC	6a129b50-c623-11e3-bf01-24c6	SBS3	SBS4	971	3857	0.0610916	0.0061147	0.0303891
Liver-HCC	6a129b50-c623-11e3-bf01-24c6	SBS3	SBS12	971	7370	0.0682326	0.0006785	0.0046571
Kidney-RCC	6bd4bca3-e568-4d1b-8b42-401	SBS5	SBS8	3325	3717	0.0386101	0.010679	0.0479063
Lymph-CLL	6cfce053-bfd6-4ca0-b74b-b2e4	SBS1	SBS9	277	1576	0.1525614	3.45E-05	0.000324
Lymph-CLL	6cfce053-bfd6-4ca0-b74b-b2e4	SBS5	SBS9	1015	1576	0.1293429	2.14E-09	3.52E-08
Stomach-AdenoCA	6dbac113-d4cf-4db5-97c9-50fa4	SBS1	SBS2	1967	1463	0.224355	0	0
Stomach-AdenoCA	6dbac113-d4cf-4db5-97c9-50fa4	SBS1	SBS3	1967	333	0.207627	4.34E-11	8.22E-10
Stomach-AdenoCA	6dbac113-d4cf-4db5-97c9-50fa4	SBS1	SBS6	1967	357	0.1303269	6.97E-05	0.0006092
Stomach-AdenoCA	6dbac113-d4cf-4db5-97c9-50fa4	SBS1	SBS9	1967	2514	0.2070031	0	0
Stomach-AdenoCA	6dbac113-d4cf-4db5-97c9-50fa4	SBS1	SBS17	1967	4740	0.2581858	0	0
Stomach-AdenoCA	6dbac113-d4cf-4db5-97c9-50fa4	SBS2	SBS6	1463	357	0.1207162	0.0004663	0.0033825
Stomach-AdenoCA	6dbac113-d4cf-4db5-97c9-50fa4	SBS6	SBS9	357	2514	0.1063089	0.0017074	0.010419
Stomach-AdenoCA	6dbac113-d4cf-4db5-97c9-50fa4	SBS6	SBS17	357	4740	0.1627256	4.63E-08	6.63E-07
Stomach-AdenoCA	6dbac113-d4cf-4db5-97c9-50fa4	SBS9	SBS17	2514	4740	0.0571463	4.38E-05	0.0004005
Panc-Endocrine	6e776415-ddf4-4060-953f-08ef2	SBS1	SBS2	1033	570	0.0902294	0.005053	0.0259371
Panc-Endocrine	6e776415-ddf4-4060-953f-08ef2	SBS1	SBS3	1033	8554	0.2255567	0	0
Panc-Endocrine	6e776415-ddf4-4060-953f-08ef2	SBS1	SBS13	1033	802	0.2244522	0	0
Panc-Endocrine	6e776415-ddf4-4060-953f-08ef2	SBS2	SBS3	570	8554	0.1450139	3.47E-10	6.23E-09
Panc-Endocrine	6e776415-ddf4-4060-953f-08ef2	SBS2	SBS13	570	802	0.1451809	1.59E-06	1.89E-05
Ovary-AdenoCA	6f981023-4269-4e8e-a4ab-2c92	SBS1	SBS3	576	5125	0.0857009	0.0009947	0.0065415
Ovary-AdenoCA	6f981023-4269-4e8e-a4ab-2c92	SBS1	SBS4	576	878	0.1189612	0.0001061	0.0008888
Ovary-AdenoCA	6f981023-4269-4e8e-a4ab-2c92	SBS1	SBS8	576	2181	0.0848259	0.0028393	0.0160306
Panc-AdenoCA	6fc0cb07-9713-4355-8543-f85d1	SBS1	SBS3	6295	3183	0.1554449	0	0
Panc-AdenoCA	6fc0cb07-9713-4355-8543-f85d1	SBS1	SBS9	6295	2448	0.1954053	0	0
Panc-AdenoCA	6fc0cb07-9713-4355-8543-f85d1	SBS1	SBS10	6295	664	0.2568318	0	0
Panc-AdenoCA	6fc0cb07-9713-4355-8543-f85d1	SBS1	SBS14	6295	565	0.2860748	0	0
Panc-AdenoCA	6fc0cb07-9713-4355-8543-f85d1	SBS3	SBS9	3183	2448	0.048218	0.0032106	0.017806
Panc-AdenoCA	6fc0cb07-9713-4355-8543-f85d1	SBS3	SBS10	3183	664	0.1187933	3.69E-07	4.78E-06
Panc-AdenoCA	6fc0cb07-9713-4355-8543-f85d1	SBS3	SBS14	3183	565	0.1391886	1.69E-08	2.55E-07
Panc-AdenoCA	6fc0cb07-9713-4355-8543-f85d1	SBS9	SBS10	2448	664	0.0741692	0.0063867	0.0313925
Panc-AdenoCA	6fc0cb07-9713-4355-8543-f85d1	SBS9	SBS14	2448	565	0.0946903	0.000532	0.0038063
Panc-AdenoCA	70dd8453-11d3-4105-a8d0-6a7	SBS1	SBS6	3626	401	0.0888884	0.0066538	0.0325266
Panc-AdenoCA	70dd8453-11d3-4105-a8d0-6a7	SBS3	SBS6	465	401	0.1181991	0.0048768	0.0252376
Skin-Melanoma	72108583-5320-46ec-945f-5ff17	SBS7	SBS17	148647	12706	0.1039086	0	0
Skin-Melanoma	72108583-5320-46ec-945f-5ff17	SBS11	SBS17	2237	12706	0.1039915	0	0
Panc-Endocrine	724bf2b8-a246-4b6f-85f8-95824	SBS1	SBS6	5178	5931	0.0412999	0.0001605	0.0012867
Kidney-RCC	72a27124-24b9-41f7-a065-774f	SBS1	SBS3	368	3653	0.0895192	0.009418	0.0433351
Panc-AdenoCA	72a5e569-99d4-47bc-be1b-629	SBS1	SBS8	2922	2701	0.0526574	0.0008329	0.0055415
Panc-AdenoCA	72f82fbd-9838-4082-b605-bc3d	SBS1	SBS3	7117	5045	0.0676336	3.73E-12	7.73E-11
Panc-AdenoCA	7410c00d-ed0b-48dc-a4bf-eb76	SBS1	SBS5	3395	1068	0.057334	0.0095803	0.043844
Panc-AdenoCA	7410c00d-ed0b-48dc-a4bf-eb76	SBS1	SBS8	3395	645	0.0721193	0.0071176	0.0344713
Panc-AdenoCA	7410c00d-ed0b-48dc-a4bf-eb76	SBS1	SBS17	3395	1460	0.1301279	1.89E-15	5.38E-14
Panc-AdenoCA	7410c00d-ed0b-48dc-a4bf-eb76	SBS5	SBS17	1068	1460	0.1000385	8.70E-06	9.07E-05
Panc-AdenoCA	7410c00d-ed0b-48dc-a4bf-eb76	SBS7	SBS17	1123	1460	0.1242397	6.18E-09	9.65E-08
Panc-AdenoCA	7410c00d-ed0b-48dc-a4bf-eb76	SBS8	SBS17	645	1460	0.0969364	0.0004464	0.0032644
Kidney-RCC	741c4656-0fd0-416e-86c8-f653	SBS3	SBS5	3151	242	0.1666348	7.60E-06	7.96E-05
Kidney-RCC	741c4656-0fd0-416e-86c8-f653	SBS4	SBS5	1133	242	0.1825804	3.37E-06	3.79E-05
Kidney-RCC	741c4656-0fd0-416e-86c8-f653	SBS5	SBS16	242	1069	0.1651965	4.20E-05	0.0003863
Ovary-AdenoCA	745b8756-0eab-423f-8cde-e0ff1	SBS3	SBS4	6061	397	0.0886429	0.0057289	0.0287687
Ovary-AdenoCA	745b8756-0eab-423f-8cde-e0ff1	SBS3	SBS6	6061	583	0.0813522	0.0017529	0.0106756
Panc-AdenoCA	754d26af-f184-497c-856a-cd1e	SBS1	SBS8	2582	2156	0.0525215	0.0030613	0.017099

Prost-AdenoCA	0bfd1043-7ec1-aaec-e050-11ac	SBS1	SBS8	2071	1269	0.0677577	0.0014562	0.0090715
Biliary-AdenoCA	760881cc-c623-11e3-bf01-24c6	SBS6	SBS15	112459	11822	0.017113	0.0038013	0.0205189
Head-SCC	7625877a-7a2b-466f-944b-9e56	SBS1	SBS11	1709	628	0.0808105	0.0049673	0.0256426
Head-SCC	7625877a-7a2b-466f-944b-9e56	SBS1	SBS13	1709	507	0.091166	0.0030085	0.0168796
Eso-AdenoCa	77c022d3-2f29-49d3-a1df-cc2e	SBS1	SBS3	2395	2001	0.0893422	5.53E-08	7.90E-07
Eso-AdenoCa	77c022d3-2f29-49d3-a1df-cc2e	SBS2	SBS3	1340	2001	0.1174629	4.82E-10	8.55E-09
Eso-AdenoCa	77c022d3-2f29-49d3-a1df-cc2e	SBS3	SBS17	2001	940	0.1293401	1.02E-09	1.74E-08
Panc-AdenoCA	78100212-65aa-4365-8b64-4b3	SBS1	SBS3	5049	1316	0.1300631	8.88E-16	2.59E-14
Panc-AdenoCA	78100212-65aa-4365-8b64-4b3	SBS1	SBS9	5049	1734	0.2442301	0	0
Panc-AdenoCA	78100212-65aa-4365-8b64-4b3	SBS1	SBS14	5049	145	0.1563874	0.0020266	0.0120615
Panc-AdenoCA	78100212-65aa-4365-8b64-4b3	SBS3	SBS9	1316	1734	0.1308639	1.49E-11	2.89E-10
Prost-AdenoCA	0bfd1043-816e-e3e4-e050-11ac	SBS7	SBS8	1114	3330	0.0721314	0.0003378	0.0025443
Prost-AdenoCA	0bfd1043-816e-e3e4-e050-11ac	SBS8	SBS15	3330	699	0.0821508	0.0008212	0.0054866
Panc-AdenoCA	78103d9b-0b8a-431e-bb58-5c9	SBS3	SBS8	4153	6878	0.0380713	0.0010991	0.0071451
Panc-AdenoCA	78103d9b-0b8a-431e-bb58-5c9	SBS3	SBS12	4153	10439	0.0718809	9.28E-14	2.24E-12
Panc-AdenoCA	78103d9b-0b8a-431e-bb58-5c9	SBS8	SBS12	6878	10439	0.0368543	2.57E-05	0.000248
Prost-AdenoCA	783579bd-e29d-457c-8236-1a8	SBS1	SBS3	441	1903	0.0880541	0.0077595	0.037121
Prost-AdenoCA	783579bd-e29d-457c-8236-1a8	SBS1	SBS16	441	1053	0.154447	7.26E-07	8.94E-06
Prost-AdenoCA	783579bd-e29d-457c-8236-1a8	SBS3	SBS16	1903	1053	0.0731543	0.0014124	0.0088428
Prost-AdenoCA	783579bd-e29d-457c-8236-1a8	SBS14	SBS16	79	1053	0.2321156	0.0007278	0.0049472
Eso-AdenoCa	7866dfb2-46b3-42b4-905b-12f8	SBS1	SBS5	5122	1217	0.0534222	0.0073013	0.0352251
Eso-AdenoCa	7866dfb2-46b3-42b4-905b-12f8	SBS1	SBS6	5122	2809	0.0406175	0.0050286	0.0258738
Eso-AdenoCa	7866dfb2-46b3-42b4-905b-12f8	SBS5	SBS6	1217	2809	0.0778133	6.84E-05	0.000599
Prost-AdenoCA	0bfd1043-8170-e3e4-e050-11ac	SBS1	SBS3	323	1174	0.1523331	1.57E-05	0.000156
Prost-AdenoCA	0bfd1043-8170-e3e4-e050-11ac	SBS1	SBS5	323	1064	0.1099071	0.0050265	0.0258738
Prost-AdenoCA	0bfd1043-8170-e3e4-e050-11ac	SBS1	SBS9	323	1305	0.1173719	0.0015955	0.0098207
Prost-AdenoCA	0bfd1043-8170-e3e4-e050-11ac	SBS1	SBS10	323	527	0.1749725	9.46E-06	9.79E-05
Prost-AdenoCA	0bfd1043-8170-e3e4-e050-11ac	SBS3	SBS5	1174	1064	0.074413	0.0041356	0.0218901
Prost-AdenoCA	0bfd1043-8170-e3e4-e050-11ac	SBS3	SBS6	1174	1202	0.0933708	6.36E-05	0.0005608
Prost-AdenoCA	0bfd1043-8170-e3e4-e050-11ac	SBS3	SBS9	1174	1305	0.0664552	0.0085181	0.0399056
Prost-AdenoCA	0bfd1043-8170-e3e4-e050-11ac	SBS5	SBS10	1064	527	0.0929898	0.0045076	0.0235411
Prost-AdenoCA	0bfd1043-8170-e3e4-e050-11ac	SBS6	SBS10	1202	527	0.1183432	6.99E-05	0.0006098
Prost-AdenoCA	0bfd1043-8170-e3e4-e050-11ac	SBS9	SBS10	1305	527	0.0963423	0.0018815	0.0112836
Panc-Endocrine	79353875-9d86-4063-a8ea-1d9	SBS1	SBS4	163	536	0.1987455	0.000103	0.0008662
Panc-Endocrine	79353875-9d86-4063-a8ea-1d9	SBS1	SBS5	163	1693	0.1964024	2.09E-05	0.0002033
Panc-Endocrine	79353875-9d86-4063-a8ea-1d9	SBS1	SBS7	163	326	0.291411	1.93E-08	2.89E-07
Panc-Endocrine	79353875-9d86-4063-a8ea-1d9	SBS5	SBS7	1693	326	0.1012089	0.0073941	0.035563
Prost-AdenoCA	79914ce9-04d8-4b0d-9f16-2e52	SBS1	SBS16	1329	2713	0.0617854	0.0022042	0.0129466
Eso-AdenoCa	7a4f6014-f7a2-459b-bc43-df164	SBS1	SBS10	11938	907	0.0573964	0.007744	0.0370755
Eso-AdenoCa	7a4f6014-f7a2-459b-bc43-df164	SBS1	SBS17	11938	5378	0.056319	1.22E-10	2.27E-09
Eso-AdenoCa	7a4f6014-f7a2-459b-bc43-df164	SBS8	SBS10	1830	907	0.0709979	0.0044247	0.023224
Eso-AdenoCa	7a4f6014-f7a2-459b-bc43-df164	SBS8	SBS17	1830	5378	0.0620974	5.34E-05	0.0004798
Eso-AdenoCa	7a4f6014-f7a2-459b-bc43-df164	SBS10	SBS15	907	1640	0.0700783	0.0064545	0.031676
Eso-AdenoCa	7a4f6014-f7a2-459b-bc43-df164	SBS15	SBS17	1640	5378	0.0603826	0.0002094	0.0016342
Eso-AdenoCa	7adcea71-1aed-450c-b0ff-b9d9	SBS1	SBS2	8043	2678	0.0380564	0.0059387	0.0297267
Eso-AdenoCa	7adcea71-1aed-450c-b0ff-b9d9	SBS1	SBS17	8043	7039	0.049467	2.10E-08	3.13E-07
Prost-AdenoCA	0bfd1043-8173-e3e4-e050-11ac	SBS3	SBS6	669	2564	0.0701381	0.0108139	0.0483386
Prost-AdenoCA	0bfd1043-8173-e3e4-e050-11ac	SBS4	SBS6	2132	2564	0.0547661	0.0018554	0.0111589
Biliary-AdenoCA	7ae872a6-c623-11e3-bf01-24c6	SBS1	SBS3	424	1491	0.0951764	0.0050533	0.0259371
Prost-AdenoCA	7ae9b843-488f-459c-8c0d-c81d	SBS1	SBS8	564	3015	0.1864278	9.10E-15	2.41E-13
Prost-AdenoCA	7ae9b843-488f-459c-8c0d-c81d	SBS1	SBS9	564	341	0.167041	1.41E-05	0.0001418
Prost-AdenoCA	7ae9b843-488f-459c-8c0d-c81d	SBS1	SBS12	564	1180	0.1608907	5.26E-09	8.32E-08
Prost-AdenoCA	7ae9b843-488f-459c-8c0d-c81d	SBS1	SBS13	564	306	0.201189	2.12E-07	2.86E-06
Prost-AdenoCA	7ae9b843-488f-459c-8c0d-c81d	SBS8	SBS12	3015	1180	0.0633963	0.0021899	0.0128746
Prost-AdenoCA	7ae9b843-488f-459c-8c0d-c81d	SBS8	SBS15	3015	527	0.1402085	4.38E-08	6.29E-07
Prost-AdenoCA	7ae9b843-488f-459c-8c0d-c81d	SBS9	SBS15	341	527	0.1319648	0.0014769	0.0091734
Prost-AdenoCA	7ae9b843-488f-459c-8c0d-c81d	SBS12	SBS15	1180	527	0.1245827	2.45E-05	0.0002377
Prost-AdenoCA	7ae9b843-488f-459c-8c0d-c81d	SBS13	SBS15	306	527	0.1531731	0.0002269	0.0017621
Skin-Melanoma	7ba70717-d65e-4a45-af79-60a9	SBS4	SBS7	7120	73496	0.0327616	1.78E-06	2.09E-05
Kidney-RCC	7bd3d112-d411-4ea8-a9a9-bd9	SBS3	SBS6	2476	548	0.1192676	5.72E-06	6.11E-05
Kidney-RCC	7bd3d112-d411-4ea8-a9a9-bd9	SBS6	SBS9	548	296	0.1671434	4.34E-05	0.0003976
Prost-AdenoCA	0bfd1043-8175-e3e4-e050-11ac	SBS1	SBS3	954	278	0.1180867	0.0049395	0.0255202
Prost-AdenoCA	0bfd1043-8175-e3e4-e050-11ac	SBS1	SBS7	954	668	0.0889918	0.0039663	0.0211548
Prost-AdenoCA	0bfd1043-8175-e3e4-e050-11ac	SBS1	SBS8	954	4111	0.0876964	1.34E-05	0.0001355

Prost-AdenoCA	0bfd1043-8175-e3e4-e050-11ac	SBS1	SBS12	954	497	0.1019471	0.0022448	0.0131353
Eso-AdenoCa	7c5c2293-3f40-4ae3-8346-6a32	SBS1	SBS5	4693	1558	0.1054217	1.02E-11	2.02E-10
Eso-AdenoCa	7c5c2293-3f40-4ae3-8346-6a32	SBS1	SBS9	4693	4834	0.0443879	0.0001682	0.0013413
Eso-AdenoCa	7c5c2293-3f40-4ae3-8346-6a32	SBS5	SBS9	1558	4834	0.0719911	9.93E-06	0.0001017
Eso-AdenoCa	7c5c2293-3f40-4ae3-8346-6a32	SBS5	SBS17	1558	63133	0.0911176	2.17E-11	4.16E-10
Eso-AdenoCa	7c5c2293-3f40-4ae3-8346-6a32	SBS9	SBS17	4834	63133	0.0312875	0.0003042	0.0023052
Prost-AdenoCA	0bfd1043-8177-e3e4-e050-11ac	SBS1	SBS8	1497	1563	0.0685107	0.0015263	0.0094614
Eso-AdenoCa	7d2da7ba-f57c-4083-9572-6fa9	SBS1	SBS11	10381	2078	0.0447706	0.0019347	0.0115807
Eso-AdenoCa	7d2da7ba-f57c-4083-9572-6fa9	SBS1	SBS17	10381	7522	0.0359131	2.60E-05	0.0002502
Eso-AdenoCa	7d2da7ba-f57c-4083-9572-6fa9	SBS11	SBS17	2078	7522	0.072062	9.06E-08	1.27E-06
Prost-AdenoCA	0bfd1043-817c-e3e4-e050-11ac	SBS6	SBS8	1461	1639	0.0871318	1.61E-05	0.0001593
Prost-AdenoCA	0bfd1043-817c-e3e4-e050-11ac	SBS6	SBS16	1461	770	0.0876939	0.0008568	0.0056818
Liver-HCC	7df3a542-c623-11e3-bf01-24c6	SBS5	SBS8	114	2899	0.1631839	0.0058079	0.029095
Liver-HCC	7df3a542-c623-11e3-bf01-24c6	SBS5	SBS16	114	8836	0.1993539	0.0002606	0.0019964
Liver-HCC	7df3a542-c623-11e3-bf01-24c6	SBS8	SBS16	2899	8836	0.0388769	0.0027253	0.0154986
Stomach-AdenoCA	7e036d40-b669-4929-a1e2-c47	SBS1	SBS3	2300	6109	0.1079426	0	0
Stomach-AdenoCA	7e036d40-b669-4929-a1e2-c47	SBS1	SBS17	2300	12619	0.1149123	0	0
Prost-AdenoCA	7e10e325-16d2-42df-b957-df4d	SBS1	SBS4	876	444	0.0940392	0.0109071	0.0486513
Prost-AdenoCA	7e10e325-16d2-42df-b957-df4d	SBS4	SBS5	444	1366	0.0961181	0.0040935	0.0217064
Prost-AdenoCA	7e10e325-16d2-42df-b957-df4d	SBS4	SBS13	444	134	0.1726166	0.0043351	0.0227728
Skin-Melanoma	7e22401d-f4cd-44c5-8a01-b08a	SBS7	SBS11	44245	3132	0.0377844	0.000472	0.0034079
Liver-HCC	7eb0ee38-fbe2-49ea-ba63-aa13	SBS8	SBS16	2599	5293	0.0513178	0.000206	0.0016095
Panc-AdenoCA	7f5fd36f-6e30-45ca-92a5-d8a8a	SBS1	SBS5	1002	1545	0.0808441	0.0007089	0.0048339
Eso-AdenoCa	7f94d650-41b9-4664-bcde-dc85	SBS6	SBS17	4530	41233	0.0628783	1.92E-14	4.89E-13
Prost-AdenoCA	0bfd1043-8181-e3e4-e050-11ac	SBS1	SBS3	986	4950	0.0862303	9.79E-06	0.0001007
Prost-AdenoCA	0bfd1043-8181-e3e4-e050-11ac	SBS1	SBS4	986	4819	0.0899023	3.59E-06	4.00E-05
Prost-AdenoCA	0bfd1043-8181-e3e4-e050-11ac	SBS1	SBS8	986	1208	0.1064791	9.01E-06	9.38E-05
Prost-AdenoCA	0bfd1043-8181-e3e4-e050-11ac	SBS3	SBS4	4950	4819	0.0368553	0.0026311	0.0150174
Liver-HCC	819b4304-c622-11e3-bf01-24c6	SBS4	SBS12	8115	4584	0.0326701	0.003849	0.0207537
Prost-AdenoCA	0bfd1043-8187-e3e4-e050-11ac	SBS1	SBS5	2278	1548	0.2250989	0	0
Prost-AdenoCA	0bfd1043-8187-e3e4-e050-11ac	SBS1	SBS6	2278	179	0.2563701	6.71E-10	1.17E-08
Prost-AdenoCA	0bfd1043-8187-e3e4-e050-11ac	SBS1	SBS8	2278	825	0.236676	0	0
Prost-AdenoCA	0bfd1043-8187-e3e4-e050-11ac	SBS5	SBS8	1548	825	0.0734132	0.0060488	0.0301336
Panc-AdenoCA	8282283d-247a-431d-9421-0fcc	SBS1	SBS3	882	7903	0.0970287	6.50E-07	8.05E-06
Panc-AdenoCA	8282283d-247a-431d-9421-0fcc	SBS1	SBS13	882	1925	0.0680726	0.0073536	0.0353954
Panc-AdenoCA	8282283d-247a-431d-9421-0fcc	SBS2	SBS3	1382	7903	0.1060728	6.39E-12	1.29E-10
Panc-AdenoCA	8282283d-247a-431d-9421-0fcc	SBS2	SBS13	1382	1925	0.0871265	9.93E-06	0.0001017
Panc-AdenoCA	8282283d-247a-431d-9421-0fcc	SBS3	SBS13	7903	1925	0.0467597	0.0022975	0.0134186
Liver-HCC	83d57c5c-c622-11e3-bf01-24c6	SBS1	SBS2	377	416	0.1156333	0.0100956	0.0457809
Panc-AdenoCA	8454fe53-869d-41c8-b0c8-a792	SBS3	SBS6	852	1509	0.0778288	0.002729	0.0155058
Panc-AdenoCA	8454fe53-869d-41c8-b0c8-a792	SBS3	SBS8	852	319	0.1274854	0.0010581	0.0069149
Panc-AdenoCA	8454fe53-869d-41c8-b0c8-a792	SBS4	SBS6	2656	1509	0.0664552	0.0004072	0.0030093
Panc-AdenoCA	8454fe53-869d-41c8-b0c8-a792	SBS4	SBS8	2656	319	0.1115095	0.0016794	0.0102779
Panc-AdenoCA	8454fe53-869d-41c8-b0c8-a792	SBS4	SBS17	2656	2434	0.0577425	0.0004196	0.00309
Panc-AdenoCA	8454fe53-869d-41c8-b0c8-a792	SBS6	SBS8	1509	319	0.1414273	5.32E-05	0.0004785
Panc-AdenoCA	8454fe53-869d-41c8-b0c8-a792	SBS6	SBS10	1509	345	0.1232431	0.0003949	0.0029321
Panc-AdenoCA	8454fe53-869d-41c8-b0c8-a792	SBS8	SBS17	319	2434	0.1552368	2.50E-06	2.89E-05
Panc-AdenoCA	8454fe53-869d-41c8-b0c8-a792	SBS10	SBS17	345	2434	0.1099199	0.0013486	0.0085196
Panc-AdenoCA	84a6ea88-eeb8-4060-a90c-e4a	SBS1	SBS3	941	8352	0.1030507	3.16E-08	4.63E-07
Prost-AdenoCA	0bfd1068-3fc3-a95b-e050-11ac	SBS1	SBS2	2352	839	0.1124694	3.21E-07	4.17E-06
Prost-AdenoCA	0bfd1068-3fc3-a95b-e050-11ac	SBS1	SBS4	2352	1386	0.0807437	2.30E-05	0.0002239
Prost-AdenoCA	0bfd1068-3fc3-a95b-e050-11ac	SBS1	SBS14	2352	231	0.1564239	6.77E-05	0.0005943
Prost-AdenoCA	0bfd1068-3fc3-a95b-e050-11ac	SBS2	SBS12	839	1246	0.1178704	1.78E-06	2.09E-05
Prost-AdenoCA	0bfd1068-3fc3-a95b-e050-11ac	SBS4	SBS12	1386	1246	0.0872935	9.08E-05	0.0007723
Prost-AdenoCA	0bfd1068-3fc3-a95b-e050-11ac	SBS12	SBS14	1246	231	0.151807	0.0002514	0.0019304
Liver-HCC	850389d4-c622-11e3-bf01-24c6	SBS4	SBS12	10266	4835	0.0817001	0	0
Liver-HCC	850389d4-c622-11e3-bf01-24c6	SBS4	SBS14	10266	1305	0.0750763	4.29E-06	4.71E-05
Liver-HCC	850389d4-c622-11e3-bf01-24c6	SBS8	SBS12	618	4835	0.0748604	0.0043029	0.0226415
Liver-HCC	850389d4-c622-11e3-bf01-24c6	SBS12	SBS14	4835	1305	0.051952	0.0077968	0.0372146
Prost-AdenoCA	0bfd1068-3fc5-a95b-e050-11ac	SBS1	SBS3	1124	2477	0.072527	0.0005868	0.0041323
Prost-AdenoCA	0bfd1068-3fc5-a95b-e050-11ac	SBS1	SBS6	1124	336	0.1107969	0.0034902	0.0191032
Prost-AdenoCA	0bfd1068-3fc5-a95b-e050-11ac	SBS3	SBS6	2477	336	0.0937434	0.0110327	0.0491063
Liver-HCC	8659a35e-c622-11e3-bf01-24c6	SBS1	SBS8	600	330	0.1125758	0.009066	0.042024
Prost-AdenoCA	0bfd1068-3fca-a95b-e050-11ac	SBS6	SBS8	54	1012	0.228883	0.0092968	0.0428718

Ovary-AdenoCA	86f23897-dba0-4e89-8381-d174	SBS1	SBS3	1039	8639	0.0706891	0.0001886	0.0014813
Kidney-RCC	00db1b95-8ca3-4cc4-bb46-6b8c	SBS2	SBS9	881	159	0.1506364	0.0044293	0.023229
Prost-AdenoCA	0bfd1068-3fcd-a95b-e050-11ac0	SBS1	SBS8	2608	1108	0.0589259	0.0090309	0.0418925
Prost-AdenoCA	887616c5-06a7-4e83-948c-3546	SBS1	SBS3	1422	604	0.1114651	5.32E-05	0.0004785
Prost-AdenoCA	887616c5-06a7-4e83-948c-3546	SBS1	SBS5	1422	339	0.1567965	2.85E-06	3.26E-05
Ovary-AdenoCA	8888e808-594b-4c76-b2e4-62aa	SBS1	SBS3	537	11732	0.1162296	1.89E-06	2.21E-05
Ovary-AdenoCA	8888e808-594b-4c76-b2e4-62aa	SBS1	SBS4	537	828	0.1180336	0.0002287	0.0017736
Ovary-AdenoCA	8888e808-594b-4c76-b2e4-62aa	SBS1	SBS13	537	1086	0.1037378	0.0008756	0.0057946
Lymph-BNHL	2439ec74-1713-4d47-a5d9-886f	SBS5	SBS6	915	2520	0.0655673	0.0062293	0.0307879
Lymph-BNHL	2439ec74-1713-4d47-a5d9-886f	SBS6	SBS9	2520	10852	0.04624	0.0003184	0.0024069
Panc-Endocrine	aa215f18-ae1f-4f0e-89dd-ac59e	SBS3	SBS11	2872	1164	0.1002247	1.19E-07	1.64E-06
Panc-Endocrine	aa215f18-ae1f-4f0e-89dd-ac59e	SBS4	SBS11	395	1164	0.1025512	0.0040459	0.0214879
Eso-AdenoCa	3b590d75-ccfb-4da8-a2e9-10fb0	SBS1	SBS17	8558	17667	0.062164	0	0
Bone-Osteosarc	f86b7e84-7040-c751-e040-11ac	SBS3	SBS14	4872	490	0.0885351	0.0018612	0.0111749
Bone-Osteosarc	f86b7e84-7040-c751-e040-11ac	SBS13	SBS14	724	490	0.1142237	0.0009759	0.0064273
Eso-AdenoCa	b8f3137e-5e92-4a56-90d4-884a	SBS1	SBS2	5403	869	0.0863544	2.83E-05	0.0002698
Eso-AdenoCa	b8f3137e-5e92-4a56-90d4-884a	SBS1	SBS13	5403	843	0.0777537	0.0002963	0.0022482
Eso-AdenoCa	b8f3137e-5e92-4a56-90d4-884a	SBS1	SBS17	5403	1814	0.0738915	7.25E-07	8.94E-06
Eso-AdenoCa	3bb4c27b-4d0d-4d13-b910-41a9	SBS1	SBS17	5603	10093	0.0821139	0	0
Eso-AdenoCa	3bb4c27b-4d0d-4d13-b910-41a9	SBS2	SBS13	1836	2030	0.0591707	0.0023401	0.013642
Eso-AdenoCa	3bb4c27b-4d0d-4d13-b910-41a9	SBS2	SBS17	1836	10093	0.1117097	0	0
Eso-AdenoCa	3bb4c27b-4d0d-4d13-b910-41a9	SBS3	SBS17	3946	10093	0.0852262	0	0
Eso-AdenoCa	3bb4c27b-4d0d-4d13-b910-41a9	SBS13	SBS17	2030	10093	0.0631858	2.76E-06	3.16E-05
Liver-HCC	1ea7e01c-c623-11e3-bf01-24c69	SBS12	SBS16	4225	4022	0.0441884	0.0006403	0.0044635
Eso-AdenoCa	9c00828e-e9ae-4b9c-959e-3483	SBS1	SBS17	2768	10058	0.0518866	1.68E-05	0.0001655
Eso-AdenoCa	9c00828e-e9ae-4b9c-959e-3483	SBS4	SBS17	3803	10058	0.0463923	1.39E-05	0.0001393
Liver-HCC	dbfa4e8a-c622-11e3-bf01-24c65	SBS8	SBS12	3017	978	0.0965124	2.12E-06	2.46E-05
Liver-HCC	dbfa4e8a-c622-11e3-bf01-24c65	SBS12	SBS16	978	6435	0.0641359	0.0018525	0.0111525
Prost-AdenoCA	41027c0b-85af-4c78-bba0-0da63	SBS1	SBS9	1232	723	0.0889388	0.0014813	0.0091912
Breast-AdenoCa	f393bb0a-9b20-a0e5-e040-11ac	SBS1	SBS3	507	2481	0.1058435	0.0001602	0.0012867
Breast-AdenoCa	f393bb0a-9b20-a0e5-e040-11ac	SBS1	SBS12	507	224	0.1377853	0.0054847	0.027787
Breast-AdenoCa	f393bb0a-9b20-a0e5-e040-11ac	SBS3	SBS13	2481	1049	0.0729448	0.0007826	0.0052532
Skin-Melanoma	351db483-a70e-496d-b70a-7449	SBS1	SBS3	484	852	0.0943235	0.0082373	0.0389023
Skin-Melanoma	351db483-a70e-496d-b70a-7449	SBS1	SBS14	484	475	0.1167856	0.0028911	0.0163084
Liver-HCC	4f1fc78e-733d-4c6e-86b2-dbe1f	SBS5	SBS16	593	9070	0.0866236	0.0004713	0.0034066
Panc-AdenoCA	cafe9603-a804-48fb-9217-e2d1c	SBS1	SBS2	6446	801	0.1879622	0	0
Panc-AdenoCA	cafe9603-a804-48fb-9217-e2d1c	SBS1	SBS6	6446	261	0.1276244	0.0005652	0.0039979
Panc-AdenoCA	cafe9603-a804-48fb-9217-e2d1c	SBS1	SBS8	6446	1174	0.1023978	1.80E-09	3.00E-08
Panc-AdenoCA	cafe9603-a804-48fb-9217-e2d1c	SBS2	SBS8	801	1174	0.0978249	0.0002204	0.001714
Eso-AdenoCa	15e7d981-8c27-4b2b-b4f8-626e	SBS14	SBS17	2908	6272	0.0434549	0.0011023	0.0071557
Eso-AdenoCa	b53dcb4d-f894-47b8-8a4e-17c79	SBS1	SBS3	11269	5445	0.0490421	4.28E-08	6.18E-07
Eso-AdenoCa	b53dcb4d-f894-47b8-8a4e-17c79	SBS1	SBS11	11269	2896	0.0534883	3.76E-06	4.19E-05
Eso-AdenoCa	b53dcb4d-f894-47b8-8a4e-17c79	SBS1	SBS17	11269	19371	0.0696481	0	0
Eso-AdenoCa	b53dcb4d-f894-47b8-8a4e-17c79	SBS3	SBS17	5445	19371	0.1138716	0	0
Eso-AdenoCa	b53dcb4d-f894-47b8-8a4e-17c79	SBS11	SBS17	2896	19371	0.1179387	0	0
Liver-HCC	5bb37ffc-c623-11e3-bf01-24c65	SBS3	SBS12	4090	12990	0.0307854	0.0055004	0.027844
Kidney-RCC	d5c87d16-172f-4790-8867-949d	SBS3	SBS6	6246	794	0.1662025	0	0
Kidney-RCC	d5c87d16-172f-4790-8867-949d	SBS4	SBS6	1322	794	0.1651189	3.58E-12	7.45E-11
Prost-AdenoCA	07f16397-71bb-4594-ad4d-caa7	SBS3	SBS8	306	1404	0.1018519	0.0108942	0.0486282
Breast-AdenoCa	fc93b9ab-760a-5223-e040-11ac	SBS1	SBS7	790	507	0.1066087	0.0017884	0.010829
Breast-AdenoCa	fc93b9ab-760a-5223-e040-11ac	SBS3	SBS7	6980	507	0.0745918	0.0103929	0.0468913
Prost-AdenoCA	0bfe2ac9-0af8-c248-e050-11ac0	SBS1	SBS7	296	623	0.1142304	0.0106356	0.0477457
Prost-AdenoCA	0bfe2ac9-0af8-c248-e050-11ac0	SBS1	SBS9	296	1191	0.1077479	0.0081346	0.0385332
Prost-AdenoCA	0bfe2ac9-0af8-c248-e050-11ac0	SBS7	SBS13	623	541	0.1038354	0.0038847	0.0208614
Head-SCC	bcc819eb-f4d8-4ddc-9fdc-a6307	SBS1	SBS2	1161	845	0.0809015	0.0033172	0.0183
Head-SCC	bcc819eb-f4d8-4ddc-9fdc-a6307	SBS1	SBS13	1161	1004	0.0947545	0.0001265	0.0010463
Panc-AdenoCA	b37d6283-6f95-4975-a794-f3d5c	SBS1	SBS2	1220	414	0.0941118	0.008376	0.0393843
Breast-AdenoCa	f7c7373c-1972-4968-e040-11ac	SBS2	SBS13	32407	22970	0.0335555	1.43E-13	3.37E-12
Eso-AdenoCa	a92023de-5c97-4bf2-aa3c-0e76	SBS6	SBS17	4473	80305	0.0752222	0	0
Eso-AdenoCa	a92023de-5c97-4bf2-aa3c-0e76	SBS7	SBS17	3985	80305	0.0593034	5.05E-12	1.03E-10
Liver-HCC	1df8939c-c622-11e3-bf01-24c65	SBS4	SBS17	2913	1496	0.0561761	0.0039064	0.0209242
Panc-Endocrine	589e0aea-9057-4b8c-8af1-74c7	SBS1	SBS2	282	203	0.1648499	0.0032729	0.0181038
Panc-Endocrine	589e0aea-9057-4b8c-8af1-74c7	SBS2	SBS3	203	473	0.1525531	0.0026903	0.0153136
Panc-Endocrine	589e0aea-9057-4b8c-8af1-74c7	SBS2	SBS5	203	2421	0.1777468	1.45E-05	0.0001449

Panc-Endocrine	589e0aea-9057-4b8c-8af1-74c74	SBS5	SBS11	2421	776	0.0993977	1.81E-05	0.0001777
Skin-Melanoma	b42d183c-bc9c-4652-9e56-10c5	SBS7	SBS11	91208	6601	0.0308703	1.61E-05	0.000159
Eso-AdenoCa	ec474dfa-527c-44f3-9224-bf1c8	SBS7	SBS17	1150	10624	0.0528634	0.0060572	0.0301513
Breast-AdenoCa	fc8130e3-023d-c7d4-e040-11ac(	SBS1	SBS3	900	5485	0.0668014	0.0020153	0.0120053
Ovary-AdenoCA	8b28f6d2-4b7d-493b-826e-b119	SBS1	SBS3	1152	11232	0.0666845	0.0001842	0.0014506
Panc-AdenoCA	d05ea63c-86a3-463a-a790-2eda	SBS1	SBS8	4543	1408	0.1499405	0	0
Ovary-AdenoCA	0d0793c1-df1b-4db1-ba36-adcb	SBS3	SBS14	10731	545	0.0851293	0.001087	0.0070816
Eso-AdenoCa	fab0be4b-c84a-45cd-a76f-44d9a	SBS1	SBS2	3911	2099	0.0843899	7.11E-09	1.11E-07
Eso-AdenoCa	fab0be4b-c84a-45cd-a76f-44d9a	SBS1	SBS6	3911	835	0.0885536	4.11E-05	0.0003786
Eso-AdenoCa	fab0be4b-c84a-45cd-a76f-44d9a	SBS1	SBS8	3911	6223	0.0786914	2.42E-13	5.57E-12
Eso-AdenoCa	fab0be4b-c84a-45cd-a76f-44d9a	SBS1	SBS17	3911	3590	0.0637676	4.90E-07	6.26E-06
Eso-AdenoCa	fab0be4b-c84a-45cd-a76f-44d9a	SBS2	SBS17	2099	3590	0.1404999	0	0
Eso-AdenoCa	fab0be4b-c84a-45cd-a76f-44d9a	SBS6	SBS17	835	3590	0.1313779	1.40E-10	2.57E-09
Eso-AdenoCa	fab0be4b-c84a-45cd-a76f-44d9a	SBS8	SBS17	6223	3590	0.1349484	0	0
Eso-AdenoCa	fab0be4b-c84a-45cd-a76f-44d9a	SBS9	SBS17	616	3590	0.1109693	4.76E-06	5.17E-05
Panc-Endocrine	c9e7cff0-aec2-4ff2-8091-7cbea2	SBS3	SBS6	8847	695	0.067052	0.0060903	0.0302918
Liver-HCC	e75fbf08-c622-11e3-bf01-24c65	SBS1	SBS16	291	6779	0.1055889	0.0039719	0.0211668
Breast-AdenoCa	fca07e09-1fd2-9ef5-e040-11ac0	SBS3	SBS13	9837	1118	0.0757909	1.96E-05	0.0001916
Breast-AdenoCa	fca07e09-1fd2-9ef5-e040-11ac0	SBS13	SBS14	1118	630	0.1058665	0.000239	0.0018471
Panc-AdenoCA	ef3a0ccd-85bc-4e5f-a8c6-5a2e2	SBS1	SBS3	5184	1056	0.1417999	9.99E-16	2.89E-14
Skin-Melanoma	4e8396f7-9506-4401-96b6-bb2e	SBS3	SBS7	8090	16657	0.0512229	7.78E-13	1.73E-11
Liver-HCC	2e0bbaf6-c623-11e3-bf01-24c65	SBS8	SBS16	900	2314	0.1007231	3.90E-06	4.31E-05
Panc-AdenoCA	ec646623-6d33-4fbd-888e-89f55	SBS1	SBS8	2311	2680	0.0703801	9.16E-06	9.52E-05
Panc-AdenoCA	ec646623-6d33-4fbd-888e-89f55	SBS7	SBS8	1419	2680	0.0953736	9.35E-08	1.31E-06
Liver-HCC	10cb8ac6-c622-11e3-bf01-24c6	SBS12	SBS16	15155	4013	0.0404724	6.12E-05	0.0005441
Panc-AdenoCA	97c46ede-b280-4344-8dbb-e860	SBS1	SBS16	3031	1733	0.0515011	0.005766	0.0289085
Panc-AdenoCA	97c46ede-b280-4344-8dbb-e860	SBS2	SBS16	567	1733	0.0848596	0.0042542	0.0224422
Panc-AdenoCA	97c46ede-b280-4344-8dbb-e860	SBS11	SBS16	296	1733	0.1032813	0.0090908	0.042077
Eso-AdenoCa	f48c3c82-bebe-4b8e-909e-e1a5	SBS1	SBS14	2867	1103	0.0628119	0.003727	0.0201702
Eso-AdenoCa	f48c3c82-bebe-4b8e-909e-e1a5	SBS1	SBS17	2867	14676	0.0863109	5.55E-16	1.65E-14
Eso-AdenoCa	f48c3c82-bebe-4b8e-909e-e1a5	SBS9	SBS17	1746	14676	0.0517023	0.0004765	0.0034366
Liver-HCC	ba940092-c622-11e3-bf01-24c6	SBS3	SBS12	2826	7285	0.0394138	0.0035781	0.0195501
Kidney-RCC	f9a81200-5381-496a-8062-099f	SBS1	SBS3	624	7573	0.0738417	0.0037213	0.0201568
Kidney-RCC	f9a81200-5381-496a-8062-099f	SBS1	SBS8	624	113	0.1760551	0.005312	0.0270874
Kidney-RCC	f9a81200-5381-496a-8062-099f	SBS1	SBS11	624	711	0.101516	0.0021195	0.0125194
Liver-HCC	9563a264-c622-11e3-bf01-24c6	SBS1	SBS4	285	417	0.1438596	0.0018105	0.0109308
Liver-HCC	9563a264-c622-11e3-bf01-24c6	SBS1	SBS8	285	173	0.1573471	0.0096827	0.0442284
Liver-HCC	9563a264-c622-11e3-bf01-24c6	SBS4	SBS5	417	4431	0.1387185	8.53E-07	1.04E-05
Liver-HCC	9563a264-c622-11e3-bf01-24c6	SBS5	SBS8	4431	173	0.1487875	0.0012574	0.0080149
Stomach-AdenoCA	25f07374-313a-4100-9a60-3d21	SBS1	SBS17	3346	28543	0.0396795	0.0001604	0.0012867
Stomach-AdenoCA	25f07374-313a-4100-9a60-3d21	SBS2	SBS17	2468	28543	0.0388357	0.0021146	0.0125019
Stomach-AdenoCA	25f07374-313a-4100-9a60-3d21	SBS8	SBS12	7769	3467	0.0440772	0.0001802	0.0014219
Stomach-AdenoCA	25f07374-313a-4100-9a60-3d21	SBS8	SBS17	7769	28543	0.0485313	6.42E-13	1.44E-11
Skin-Melanoma	2790b964-63e3-49aa-bf8c-9a00	SBS7	SBS11	374490	21223	0.0271113	3.01E-13	6.88E-12
Kidney-RCC	c19a1388-95e6-4708-a24c-3738	SBS1	SBS8	474	4095	0.0883974	0.002616	0.0149447
Kidney-RCC	c19a1388-95e6-4708-a24c-3738	SBS1	SBS16	474	599	0.1682375	6.25E-07	7.80E-06
Kidney-RCC	c19a1388-95e6-4708-a24c-3738	SBS3	SBS16	45	599	0.2966797	0.0012621	0.0080298
Kidney-RCC	c19a1388-95e6-4708-a24c-3738	SBS8	SBS16	4095	599	0.0968925	0.0001096	0.0009134
Kidney-RCC	c19a1388-95e6-4708-a24c-3738	SBS13	SBS16	462	599	0.1013702	0.0093972	0.0432714
Eso-AdenoCa	526b3796-2cbd-4eec-8273-064b	SBS5	SBS8	4974	1309	0.0551361	0.0036705	0.0199662
Prost-AdenoCA	8c0a7dfa-6ec3-4262-b718-c360	SBS1	SBS5	1639	1188	0.0758706	0.0007199	0.0048988
Biliary-AdenoCA	f0e0d978-3e32-4444-a07a-2604	SBS1	SBS7	3485	1585	0.0491263	0.0104037	0.0469065
Biliary-AdenoCA	f0e0d978-3e32-4444-a07a-2604	SBS3	SBS7	2354	1585	0.0736996	6.80E-05	0.0005956
Biliary-AdenoCA	f0e0d978-3e32-4444-a07a-2604	SBS7	SBS8	1585	3063	0.0593717	0.0012676	0.0080571
Lymph-BNHL	9c27fedd-b1b3-4af0-9e9b-20271	SBS3	SBS9	6157	5831	0.0347592	0.0014395	0.0089943
Lymph-BNHL	9c27fedd-b1b3-4af0-9e9b-20271	SBS3	SBS11	6157	2400	0.0747795	8.19E-09	1.27E-07
Lymph-BNHL	9c27fedd-b1b3-4af0-9e9b-20271	SBS3	SBS13	6157	1858	0.1008372	4.96E-13	1.12E-11
Lymph-BNHL	9c27fedd-b1b3-4af0-9e9b-20271	SBS6	SBS11	3804	2400	0.0977379	1.23E-12	2.67E-11
Lymph-BNHL	9c27fedd-b1b3-4af0-9e9b-20271	SBS6	SBS13	3804	1858	0.1287399	0	0
Lymph-BNHL	9c27fedd-b1b3-4af0-9e9b-20271	SBS9	SBS11	5831	2400	0.097408	1.94E-14	4.93E-13
Lymph-BNHL	9c27fedd-b1b3-4af0-9e9b-20271	SBS9	SBS13	5831	1858	0.1335311	0	0
Panc-AdenoCA	d5796835-d6cd-427e-a398-5fbe	SBS1	SBS17	2887	931	0.0842099	9.22E-05	0.0007822
Kidney-RCC	3afae3fb-2d87-4724-a589-d31c	SBS3	SBS8	491	2724	0.0809969	0.0085198	0.0399056
Kidney-RCC	494701ce-05d4-440b-a65e-d92	SBS3	SBS6	3704	264	0.1251882	0.0008839	0.0058371

Kidney-RCC	494701ce-05d4-440b-a65e-d925	SBS4	SBS6	495	264	0.1227273	0.0111824	0.0497022
Eso-AdenoCa	28e81540-4744-4865-b627-c7c9	SBS1	SBS17	2281	8409	0.0489116	0.0003737	0.002795
Eso-AdenoCa	28e81540-4744-4865-b627-c7c9	SBS3	SBS17	1542	8409	0.0702916	5.11E-06	5.54E-05
Eso-AdenoCa	cb753e6f-5ea3-4b58-9db3-7a62	SBS1	SBS3	9264	3601	0.0652758	5.06E-10	8.88E-09
Eso-AdenoCa	cb753e6f-5ea3-4b58-9db3-7a62	SBS1	SBS17	9264	5906	0.132041	0	0
Eso-AdenoCa	cb753e6f-5ea3-4b58-9db3-7a62	SBS3	SBS7	3601	1938	0.0533208	0.0015476	0.0095836
Eso-AdenoCa	cb753e6f-5ea3-4b58-9db3-7a62	SBS3	SBS17	3601	5906	0.1922977	0	0
Eso-AdenoCa	cb753e6f-5ea3-4b58-9db3-7a62	SBS7	SBS17	1938	5906	0.1503094	0	0
Liver-HCC	446437de-c622-11e3-bf01-24c6	SBS3	SBS4	1063	5087	0.0674172	0.0006759	0.0046444
Liver-HCC	446437de-c622-11e3-bf01-24c6	SBS4	SBS16	5087	5252	0.0453998	4.73E-05	0.0004301
Panc-AdenoCA	28f41a20-b6d6-4ecc-888f-72f77	SBS1	SBS3	412	3088	0.099656	0.0014634	0.0091077
Panc-AdenoCA	28f41a20-b6d6-4ecc-888f-72f77	SBS1	SBS5	412	1074	0.1150585	0.0007534	0.0050934
Biliary-AdenoCA	ec399861-7a56-4ffd-8619-700b6	SBS1	SBS2	2455	4419	0.0679339	9.43E-07	1.15E-05
Biliary-AdenoCA	ec399861-7a56-4ffd-8619-700b6	SBS1	SBS13	2455	2919	0.0546199	0.0007008	0.0047894
Biliary-AdenoCA	ec399861-7a56-4ffd-8619-700b6	SBS2	SBS8	4419	4076	0.0793909	4.93E-12	1.01E-10
Biliary-AdenoCA	ec399861-7a56-4ffd-8619-700b6	SBS8	SBS13	4076	2919	0.0671364	4.39E-07	5.64E-06
Eso-AdenoCa	23fa90d5-b273-4e2c-9c88-68aa	SBS1	SBS8	841	3869	0.0843852	0.0001067	0.0008904
Eso-AdenoCa	23fa90d5-b273-4e2c-9c88-68aa	SBS3	SBS4	172	76	0.2337821	0.0062922	0.0310008
Eso-AdenoCa	23fa90d5-b273-4e2c-9c88-68aa	SBS3	SBS15	172	571	0.1545636	0.0036155	0.0197027
Eso-AdenoCa	23fa90d5-b273-4e2c-9c88-68aa	SBS4	SBS8	76	3869	0.1942294	0.0072222	0.0348702
Eso-AdenoCa	23fa90d5-b273-4e2c-9c88-68aa	SBS5	SBS8	1643	3869	0.0483079	0.0091916	0.0424806
Eso-AdenoCa	23fa90d5-b273-4e2c-9c88-68aa	SBS5	SBS15	1643	571	0.0871361	0.0032101	0.017806
Eso-AdenoCa	23fa90d5-b273-4e2c-9c88-68aa	SBS8	SBS15	3869	571	0.1220374	7.32E-07	8.99E-06
Bone-Osteosarc	f86e2d80-911b-7a19-e040-11ac	SBS3	SBS17	5571	2517	0.0445374	0.0020606	0.0122407
Liver-HCC	c7d798b8-c622-11e3-bf01-24c6	SBS8	SBS12	1142	6346	0.0663504	0.0003983	0.0029538
Liver-HCC	c7d798b8-c622-11e3-bf01-24c6	SBS12	SBS16	6346	5193	0.0377045	0.000595	0.0041847
Eso-AdenoCa	2e69c886-535b-4ff0-9236-abf67	SBS1	SBS3	984	4140	0.0950778	1.14E-06	1.38E-05
Eso-AdenoCa	2e69c886-535b-4ff0-9236-abf67	SBS1	SBS9	984	5875	0.0944615	5.87E-07	7.40E-06
Eso-AdenoCa	2e69c886-535b-4ff0-9236-abf67	SBS1	SBS17	984	9426	0.0785283	3.38E-05	0.0003181
Eso-AdenoCa	2e69c886-535b-4ff0-9236-abf67	SBS9	SBS17	5875	9426	0.0282651	0.0061595	0.0305325
Breast-LobularCa	fc8130e0-a38a-23b9-e040-11ac	SBS1	SBS8	463	8585	0.1110056	3.97E-05	0.0003686
Breast-LobularCa	fc8130e0-a38a-23b9-e040-11ac	SBS1	SBS11	463	972	0.1156663	0.0004536	0.003313
Skin-Melanoma	bcf76f1a-7109-422d-94c9-5e836	SBS1	SBS13	1816	1057	0.0880984	6.27E-05	0.0005538
Skin-Melanoma	bcf76f1a-7109-422d-94c9-5e836	SBS1	SBS16	1816	3873	0.0589039	0.000376	0.0028049
Prost-AdenoCA	0bfebf9f-c781-e57d-e050-11ac0	SBS1	SBS16	1854	1965	0.0615702	0.0014453	0.0090216
Stomach-AdenoCA	dbd834cb-b14f-4380-9741-f9655	SBS8	SBS17	805	1870	0.0912612	0.0001698	0.0013525
Stomach-AdenoCA	dbd834cb-b14f-4380-9741-f9655	SBS13	SBS17	914	1870	0.1343662	4.72E-10	8.40E-09
CNS-Medullo	49fa7e44-c86f-4b1f-8849-e5fe5	SBS1	SBS11	2959	744	0.0696113	0.0062913	0.0310008
Head-SCC	1ff155dd-deb4-44e0-b3a2-e487	SBS1	SBS2	1247	3368	0.0863629	2.54E-06	2.93E-05
Head-SCC	1ff155dd-deb4-44e0-b3a2-e487	SBS1	SBS13	1247	3938	0.1023148	4.89E-09	7.82E-08
Head-SCC	1ff155dd-deb4-44e0-b3a2-e487	SBS2	SBS3	3368	3620	0.0561111	3.39E-05	0.0003181
Head-SCC	1ff155dd-deb4-44e0-b3a2-e487	SBS3	SBS13	3620	3938	0.0708139	1.22E-08	1.87E-07
Panc-AdenoCA	b54b9433-ec10-4cb5-a860-4555	SBS1	SBS3	5187	1122	0.0571771	0.004804	0.0249116
Bone-Osteosarc	f82d2146-70f5-8114-e040-11ac	SBS1	SBS2	202	988	0.1246843	0.010874	0.0485725
Bone-Osteosarc	f82d2146-70f5-8114-e040-11ac	SBS2	SBS5	988	1850	0.0876562	0.0001006	0.0008511
Skin-Melanoma	51893d3f-e7f3-43f9-9fd0-c0f25a	SBS7	SBS11	323008	12897	0.0169879	0.0015572	0.0096335
Head-SCC	fb8b5ff5-4164-4276-b75b-b0ee6	SBS1	SBS2	2641	2103	0.0496771	0.0061874	0.0306293
Bone-Osteosarc	f85add3d-e2e6-31f8-e040-11ac	SBS1	SBS3	486	13460	0.0843163	0.0025384	0.0145546
Prost-AdenoCA	07531318-87e8-4db8-aa61-9b9	SBS1	SBS12	2472	634	0.07963	0.0033261	0.0183328
Prost-AdenoCA	07531318-87e8-4db8-aa61-9b9	SBS5	SBS12	155	634	0.1460161	0.0098741	0.0448088
Liver-HCC	19085f7d-e6a6-406f-bd09-520a	SBS4	SBS5	2293	6818	0.0516479	0.0002115	0.0016486
Liver-HCC	19085f7d-e6a6-406f-bd09-520a	SBS4	SBS12	2293	1003	0.0966238	4.39E-06	4.80E-05
Liver-HCC	19085f7d-e6a6-406f-bd09-520a	SBS5	SBS12	6818	1003	0.0606751	0.0031994	0.0177912
Prost-AdenoCA	c14b2c74-447b-4526-a6e9-415	SBS1	SBS16	2044	999	0.0810997	0.0002934	0.0022291
Bone-Osteosarc	f843ed5c-0336-3d33-e040-11ac	SBS4	SBS14	1090	185	0.1364493	0.0055381	0.027967
Panc-AdenoCA	94652d14-2e4d-4f4a-a4f7-8df77	SBS1	SBS3	2455	492	0.0927964	0.0017197	0.0104838
Panc-AdenoCA	8be6b14d-286a-471b-a282-ab9	SBS1	SBS2	1955	408	0.183312	2.81E-10	5.06E-09
Panc-AdenoCA	8be6b14d-286a-471b-a282-ab9	SBS1	SBS5	1955	1100	0.0690235	0.0024438	0.0141507
Panc-AdenoCA	8be6b14d-286a-471b-a282-ab9	SBS2	SBS5	408	1100	0.1422193	1.18E-05	0.0001198
Panc-AdenoCA	8be6b14d-286a-471b-a282-ab9	SBS2	SBS6	408	364	0.1384131	0.0012584	0.0080149
Panc-AdenoCA	8be6b14d-286a-471b-a282-ab9	SBS2	SBS7	408	207	0.1684783	0.0008227	0.005491
Lymph-BNHL	5bcf473f-417d-47a5-9745-ac7fa	SBS1	SBS5	698	4895	0.0893277	0.0001167	0.0009699
Lymph-BNHL	5bcf473f-417d-47a5-9745-ac7fa	SBS1	SBS8	698	2621	0.0969375	6.34E-05	0.0005596
Liver-HCC	c271b7d2-c622-11e3-bf01-24c6	SBS1	SBS13	218	285	0.1687591	0.0017601	0.0106938

Liver-HCC	c271b7d2-c622-11e3-bf01-24c6!	SBS1	SBS17	218	349	0.1408612	0.0097377	0.0444472
Liver-HCC	532259b8-c622-11e3-bf01-24c6!	SBS5	SBS8	12641	15388	0.0363489	2.17E-08	3.22E-07
Liver-HCC	532259b8-c622-11e3-bf01-24c6!	SBS5	SBS9	12641	1065	0.0563595	0.0038997	0.0209063
Panc-AdenoCA	0fbd94b1-bb34-4620-841b-861a	SBS1	SBS3	3412	2252	0.0522938	0.0011987	0.007697
Panc-AdenoCA	0fbd94b1-bb34-4620-841b-861a	SBS1	SBS7	3412	676	0.0707076	0.0070939	0.0343832
Panc-AdenoCA	0fbd94b1-bb34-4620-841b-861a	SBS1	SBS17	3412	2854	0.1183253	0	0
Panc-AdenoCA	0fbd94b1-bb34-4620-841b-861a	SBS3	SBS7	2252	676	0.1052083	2.01E-05	0.0001958
Panc-AdenoCA	0fbd94b1-bb34-4620-841b-861a	SBS3	SBS17	2252	2854	0.0728329	3.17E-06	3.57E-05
Panc-AdenoCA	0fbd94b1-bb34-4620-841b-861a	SBS6	SBS7	722	676	0.1009687	0.0016202	0.0099448
Panc-AdenoCA	0fbd94b1-bb34-4620-841b-861a	SBS6	SBS17	722	2854	0.1030308	9.73E-06	0.0001004
Panc-AdenoCA	0fbd94b1-bb34-4620-841b-861a	SBS7	SBS17	676	2854	0.1678004	8.59E-14	2.10E-12
Liver-HCC	34030a28-c622-11e3-bf01-24c6!	SBS8	SBS12	1986	7092	0.0749259	5.44E-08	7.78E-07
Lymph-NOS	1494bb16-f1f0-42a4-b10e-c3835	SBS1	SBS3	546	8401	0.0938882	0.0002375	0.0018375
Lymph-NOS	1494bb16-f1f0-42a4-b10e-c3835	SBS1	SBS9	546	1630	0.1401582	2.10E-07	2.84E-06
Lymph-NOS	1494bb16-f1f0-42a4-b10e-c3835	SBS1	SBS12	546	119	0.2207498	0.0001464	0.0011945
Lymph-NOS	1494bb16-f1f0-42a4-b10e-c3835	SBS3	SBS9	8401	1630	0.0559065	0.0003935	0.0029255
Lymph-NOS	1494bb16-f1f0-42a4-b10e-c3835	SBS6	SBS9	259	1630	0.1333112	0.0007098	0.0048354
Lymph-NOS	1494bb16-f1f0-42a4-b10e-c3835	SBS6	SBS12	259	119	0.2048603	0.002132	0.0125692
Liver-HCC	30a8f292-c623-11e3-bf01-24c65	SBS1	SBS5	298	1507	0.1271093	0.0006448	0.0044803
Liver-HCC	30a8f292-c623-11e3-bf01-24c65	SBS1	SBS11	298	1140	0.1161839	0.003397	0.0186421
Liver-HCC	30a8f292-c623-11e3-bf01-24c65	SBS4	SBS5	1949	1507	0.0686347	0.0006663	0.0045985
Liver-HCC	30a8f292-c623-11e3-bf01-24c65	SBS4	SBS12	1949	4032	0.052687	0.0013585	0.0085739
Liver-HCC	30a8f292-c623-11e3-bf01-24c65	SBS5	SBS12	1507	4032	0.1027916	1.71E-10	3.11E-09
Liver-HCC	30a8f292-c623-11e3-bf01-24c65	SBS11	SBS12	1140	4032	0.0967314	1.20E-07	1.65E-06
Panc-AdenoCA	53534b3c-cd15-4d68-a9b1-6902	SBS1	SBS3	4490	6520	0.0456847	3.03E-05	0.0002869
Panc-AdenoCA	01b0848c-ae36-4ade-a3da-d297	SBS3	SBS4	26378	2858	0.0322685	0.0093083	0.0428936
Panc-AdenoCA	01b0848c-ae36-4ade-a3da-d297	SBS3	SBS6	26378	4050	0.1038443	0	0
Panc-AdenoCA	01b0848c-ae36-4ade-a3da-d297	SBS4	SBS6	2858	4050	0.092105	9.01E-13	1.98E-11
Eso-AdenoCa	42629ae0-9410-4fac-8df1-2c276	SBS15	SBS17	1705	45213	0.1162718	0	0
Panc-AdenoCA	a3edc9cc-f54a-4459-a5d0-09787	SBS1	SBS8	3108	720	0.0714125	0.0051475	0.0263937
Eso-AdenoCa	b757a040-3c9e-45e7-a045-9cd0	SBS2	SBS8	4407	6956	0.0433639	7.85E-05	0.0006783
Eso-AdenoCa	b757a040-3c9e-45e7-a045-9cd0	SBS2	SBS17	4407	42227	0.0612824	1.92E-13	4.50E-12
Eso-AdenoCa	b757a040-3c9e-45e7-a045-9cd0	SBS8	SBS17	6956	42227	0.0346519	1.18E-06	1.43E-05
Liver-HCC	10db2142-c623-11e3-bf01-24c6!	SBS3	SBS12	1193	4169	0.0542205	0.0085597	0.0400324
Liver-HCC	10db2142-c623-11e3-bf01-24c6!	SBS4	SBS12	1720	4169	0.0589335	0.0004244	0.0031214
Lymph-BNHL	282b45ee-4af2-4c61-9acc-286ea	SBS1	SBS9	2797	1473	0.0679729	0.0002685	0.0020517
Lymph-BNHL	282b45ee-4af2-4c61-9acc-286ea	SBS9	SBS14	1473	645	0.0988554	0.0003115	0.0023575
Lymph-BNHL	124d5b04-4588-44c3-9c00-61e0	SBS5	SBS7	3625	782	0.0682328	0.00501	0.0258416
Lymph-BNHL	124d5b04-4588-44c3-9c00-61e0	SBS5	SBS8	3625	4318	0.0438159	0.0010349	0.0067772
Panc-AdenoCA	31c75873-abb4-4d88-9e2f-0749	SBS1	SBS2	5465	1762	0.1531097	0	0
Panc-AdenoCA	31c75873-abb4-4d88-9e2f-0749	SBS1	SBS17	5465	1870	0.1414074	0	0
Panc-AdenoCA	31c75873-abb4-4d88-9e2f-0749	SBS2	SBS4	1762	558	0.1427142	6.37E-08	9.00E-07
Panc-AdenoCA	31c75873-abb4-4d88-9e2f-0749	SBS2	SBS8	1762	1843	0.1595561	0	0
Panc-AdenoCA	31c75873-abb4-4d88-9e2f-0749	SBS2	SBS17	1762	1870	0.2912939	0	0
Panc-AdenoCA	31c75873-abb4-4d88-9e2f-0749	SBS4	SBS17	558	1870	0.1570927	1.23E-09	2.09E-08
Panc-AdenoCA	31c75873-abb4-4d88-9e2f-0749	SBS8	SBS17	1843	1870	0.1390093	5.55E-16	1.65E-14
Lymph-BNHL	4a4309b0-2aca-4d6a-ac50-039b	SBS1	SBS9	2338	2628	0.0543557	0.0013361	0.0084496
Lymph-BNHL	4a4309b0-2aca-4d6a-ac50-039b	SBS1	SBS12	2338	817	0.0729096	0.0032032	0.0177965
Kidney-RCC	959efb81-1d56-4659-9b7a-09f2b	SBS1	SBS3	394	973	0.2067967	7.66E-11	1.44E-09
Kidney-RCC	959efb81-1d56-4659-9b7a-09f2b	SBS1	SBS8	394	2666	0.172069	2.97E-09	4.86E-08
Kidney-RCC	959efb81-1d56-4659-9b7a-09f2b	SBS1	SBS11	394	528	0.1234137	0.0020704	0.0122872
Kidney-RCC	959efb81-1d56-4659-9b7a-09f2b	SBS3	SBS11	973	528	0.0987554	0.0025216	0.0144978
Liver-HCC	8a6d6b2e-c622-11e3-bf01-24c6!	SBS1	SBS8	337	5842	0.0955361	0.0059579	0.0297573
Liver-HCC	8a6d6b2e-c622-11e3-bf01-24c6!	SBS4	SBS8	750	5842	0.0679817	0.0042945	0.0226166
Skin-Melanoma	deb9fbb6-656b-41ce-8299-554e	SBS7	SBS11	450378	27093	0.0190653	1.71E-08	2.58E-07
Panc-AdenoCA	2b3837b3-ee29-4a08-848e-1206	SBS1	SBS2	2519	486	0.0836311	0.0066998	0.0327262
Panc-AdenoCA	2b3837b3-ee29-4a08-848e-1206	SBS2	SBS5	486	1179	0.1068859	0.0007692	0.0051777
Panc-AdenoCA	e5193d7e-e8b7-4098-bf98-8b3a	SBS1	SBS7	4884	378	0.0958426	0.003175	0.0176867
Liver-HCC	b994762c-c622-11e3-bf01-24c6!	SBS3	SBS12	1756	8275	0.060516	4.93E-05	0.0004461
Liver-HCC	b994762c-c622-11e3-bf01-24c6!	SBS4	SBS12	2015	8275	0.0423423	0.0059925	0.0298767
Liver-HCC	b994762c-c622-11e3-bf01-24c6!	SBS12	SBS16	8275	7460	0.0386086	1.67E-05	0.0001644
Breast-AdenoCa	fc447d55-95d8-0b34-e040-11ac	SBS2	SBS12	989	310	0.1130337	0.0048058	0.0249116
Skin-Melanoma	ca004926-f3ac-4824-8ed5-d84e	SBS1	SBS13	268	918	0.120094	0.0050393	0.0259075
Skin-Melanoma	ca004926-f3ac-4824-8ed5-d84e	SBS3	SBS13	2898	918	0.0684042	0.002935	0.0164963

Skin-Melanoma	ca004926-f3ac-4824-8ed5-d84e	SBS13	SBS15	918	210	0.1555867	0.00051	0.003657
Eso-AdenoCa	96a2896c-1e32-4827-a526-6b71	SBS9	SBS17	2018	35533	0.0592037	3.07E-06	3.47E-05
Ovary-AdenoCA	25f73b03-4ba8-47ba-ad80-6ccb	SBS1	SBS3	4509	7229	0.0320047	0.0067672	0.0330297
Liver-HCC	520b8ab2-c623-11e3-bf01-24c6	SBS5	SBS12	4871	7601	0.045922	7.30E-06	7.69E-05
Liver-HCC	520b8ab2-c623-11e3-bf01-24c6	SBS12	SBS15	7601	967	0.0611741	0.003255	0.0180204
Prost-AdenoCA	f9c3eaad-a0d9-8bf8-e040-11ac	SBS1	SBS3	259	1247	0.1161645	0.0061291	0.0304364
Prost-AdenoCA	f9c3eaad-a0d9-8bf8-e040-11ac	SBS1	SBS8	259	1487	0.1655168	1.13E-05	0.0001149
Prost-AdenoCA	f9c3eaad-a0d9-8bf8-e040-11ac	SBS1	SBS11	259	592	0.1206564	0.0105378	0.047409
Prost-AdenoCA	f9c3eaad-a0d9-8bf8-e040-11ac	SBS8	SBS15	1487	536	0.0961605	0.0013698	0.0086364
Lymph-BNHL	3e94aa64-7dd3-4d63-a5de-b40	SBS6	SBS9	821	2151	0.0663086	0.0107584	0.0481593
Panc-AdenoCA	e4c8c7f0-5bac-4d59-91c4-c982	SBS1	SBS3	672	7898	0.1568326	1.17E-13	2.81E-12
Panc-AdenoCA	02c97e2b-914e-4afc-bf50-78f0c	SBS1	SBS4	1063	459	0.1151364	0.0004072	0.0030093
Panc-AdenoCA	02c97e2b-914e-4afc-bf50-78f0c	SBS1	SBS8	1063	1101	0.0942895	0.0001332	0.0010972
Panc-AdenoCA	96cca60e-17dc-44c3-90f7-1b57	SBS1	SBS5	3958	171	0.1430296	0.0024454	0.0141507
Lymph-BNHL	232b7754-b3c3-4530-9d24-3af4	SBS1	SBS8	487	366	0.1365335	0.0008272	0.0055152
Lymph-BNHL	232b7754-b3c3-4530-9d24-3af4	SBS1	SBS9	487	3010	0.0961108	0.0008665	0.0057405
Liver-HCC	cc792058-c622-11e3-bf01-24c6	SBS3	SBS4	1371	2563	0.1004037	3.02E-08	4.43E-07
Liver-HCC	cc792058-c622-11e3-bf01-24c6	SBS4	SBS12	2563	5543	0.0977568	5.66E-15	1.52E-13
Liver-HCC	a846f5e8-c622-11e3-bf01-24c6	SBS4	SBS17	3688	1417	0.1213145	1.64E-13	3.87E-12
Liver-HCC	a846f5e8-c622-11e3-bf01-24c6	SBS11	SBS17	1177	1417	0.1420936	1.06E-11	2.08E-10
Liver-HCC	a846f5e8-c622-11e3-bf01-24c6	SBS12	SBS17	4628	1417	0.1475621	0	0
Lymph-CLL	915cbb43-9e00-433d-818f-5310	SBS1	SBS17	900	719	0.1860238	1.94E-12	4.16E-11
Lymph-CLL	915cbb43-9e00-433d-818f-5310	SBS8	SBS17	1452	719	0.1685058	2.76E-12	5.81E-11
Lymph-CLL	915cbb43-9e00-433d-818f-5310	SBS12	SBS17	818	719	0.1409558	4.98E-07	6.36E-06
Liver-HCC	f410b432-c622-11e3-bf01-24c6	SBS8	SBS9	3715	792	0.06729	0.0054141	0.0275405
Liver-HCC	f410b432-c622-11e3-bf01-24c6	SBS8	SBS16	3715	3258	0.0423655	0.0039357	0.0210276
Panc-AdenoCA	12f038e1-00af-4c64-a2e0-9e63	SBS1	SBS3	1263	1283	0.1639313	2.78E-15	7.77E-14
Panc-AdenoCA	12f038e1-00af-4c64-a2e0-9e63	SBS1	SBS8	1263	3684	0.0714917	0.0001335	0.0010981
Panc-AdenoCA	12f038e1-00af-4c64-a2e0-9e63	SBS1	SBS15	1263	978	0.0739791	0.0047956	0.0249116
Panc-AdenoCA	12f038e1-00af-4c64-a2e0-9e63	SBS3	SBS6	1283	546	0.1167822	5.81E-05	0.0005186
Panc-AdenoCA	12f038e1-00af-4c64-a2e0-9e63	SBS3	SBS7	1283	1134	0.1262308	9.33E-09	1.44E-07
Panc-AdenoCA	12f038e1-00af-4c64-a2e0-9e63	SBS3	SBS8	1283	3684	0.104755	1.70E-09	2.84E-08
Panc-AdenoCA	12f038e1-00af-4c64-a2e0-9e63	SBS3	SBS15	1283	978	0.1261375	4.28E-08	6.18E-07
Eso-AdenoCa	31f3ff14-7d74-447c-a5da-9ad83	SBS1	SBS17	11595	13297	0.0898031	0	0
Eso-AdenoCa	31f3ff14-7d74-447c-a5da-9ad83	SBS5	SBS17	7428	13297	0.0760805	0	0
Kidney-RCC	f37971bd-ec65-4840-8d4f-6786	SBS1	SBS5	154	1906	0.1874872	8.92E-05	0.000763
Kidney-RCC	f37971bd-ec65-4840-8d4f-6786	SBS5	SBS11	1906	426	0.1271571	2.58E-05	0.0002485
Lymph-CLL	8cce153d-953d-40c5-976b-60f6	SBS1	SBS9	600	974	0.0884771	0.0059772	0.029824
Lymph-CLL	8cce153d-953d-40c5-976b-60f6	SBS5	SBS9	1500	974	0.117117	1.84E-07	2.50E-06
Lymph-CLL	8cce153d-953d-40c5-976b-60f6	SBS6	SBS9	229	974	0.1675663	6.01E-05	0.0005361
Lymph-BNHL	abedd46c-47b8-4242-adb6-128	SBS6	SBS9	4497	11948	0.047841	6.39E-07	7.95E-06
Lymph-BNHL	abedd46c-47b8-4242-adb6-128	SBS6	SBS17	4497	4876	0.0456618	0.000116	0.0009651
Liver-HCC	b2d9aaff-5a48-48f0-bf55-bfd4dd	SBS1	SBS8	354	1272	0.0983371	0.0094402	0.0434027
Liver-HCC	b2d9aaff-5a48-48f0-bf55-bfd4dd	SBS1	SBS12	354	1181	0.1697355	3.06E-07	4.00E-06
Liver-HCC	b2d9aaff-5a48-48f0-bf55-bfd4dd	SBS1	SBS16	354	1565	0.1272757	0.0001734	0.0013788
Liver-HCC	b2d9aaff-5a48-48f0-bf55-bfd4dd	SBS3	SBS12	812	1181	0.1076684	2.86E-05	0.0002718
Liver-HCC	b2d9aaff-5a48-48f0-bf55-bfd4dd	SBS8	SBS12	1272	1181	0.0879431	0.0001538	0.0012409
Breast-AdenoCa	fc8130e5-18b8-ef38-e040-11ac	SBS1	SBS13	1844	598	0.0834162	0.0037313	0.0201759
Panc-AdenoCA	2bf5b018-9f19-4fbd-9e1f-7d958	SBS1	SBS3	618	10749	0.1073006	2.86E-06	3.27E-05
Panc-AdenoCA	2bf5b018-9f19-4fbd-9e1f-7d958	SBS1	SBS4	618	159	0.157019	0.0039157	0.0209564
Panc-AdenoCA	2bf5b018-9f19-4fbd-9e1f-7d958	SBS1	SBS13	618	1553	0.1199797	5.94E-06	6.33E-05
Panc-AdenoCA	2bf5b018-9f19-4fbd-9e1f-7d958	SBS3	SBS6	10749	1666	0.0722808	5.69E-07	7.21E-06
Panc-AdenoCA	2bf5b018-9f19-4fbd-9e1f-7d958	SBS6	SBS13	1666	1553	0.0887219	6.39E-06	6.78E-05
Breast-AdenoCa	fc9dbf68-4599-3c64-e040-11ac	SBS1	SBS3	287	2298	0.1093877	0.0044601	0.0233512
Kidney-RCC	f6e758a5-150f-4424-90b7-1c1a	SBS3	SBS8	1898	1545	0.0637015	0.0019911	0.0118727
Breast-AdenoCa	fc8130e0-096a-b991-e040-11ac	SBS2	SBS13	24143	12065	0.0216203	0.0010834	0.007065
Lymph-BNHL	ce85ccf3-6621-4976-b187-2f28	SBS3	SBS7	7125	3105	0.0508307	2.80E-05	0.0002678
Lymph-BNHL	ce85ccf3-6621-4976-b187-2f28	SBS7	SBS15	3105	2658	0.0441166	0.0075872	0.036408
Stomach-AdenoCA	37e5d47b-851b-47de-ba6a-fd28	SBS8	SBS17	537	2171	0.0777242	0.0110166	0.0490698
Eso-AdenoCa	3a551616-4840-4111-a8c5-b4a	SBS5	SBS6	1332	1028	0.0983863	2.65E-05	0.0002544
Eso-AdenoCa	3a551616-4840-4111-a8c5-b4a	SBS5	SBS17	1332	2293	0.0753632	0.0001395	0.0011411
Eso-AdenoCa	3a551616-4840-4111-a8c5-b4a	SBS6	SBS9	1028	673	0.1360408	5.79E-07	7.32E-06
Eso-AdenoCa	3a551616-4840-4111-a8c5-b4a	SBS6	SBS17	1028	2293	0.1684996	0	0
Eso-AdenoCa	3a551616-4840-4111-a8c5-b4a	SBS13	SBS17	469	2293	0.1064917	0.0002922	0.0022226

Liver-HCC	53df19a8-c623-11e3-bf01-24c65	SBS4	SBS5	7964	6996	0.0488085	3.93E-08	5.72E-07
Liver-HCC	53df19a8-c623-11e3-bf01-24c65	SBS5	SBS9	6996	524	0.1012443	9.14E-05	0.0007758
Lymph-BNHL	0e7f46ca-6f5c-4538-b6d6-00af6	SBS3	SBS6	4391	3494	0.053593	2.80E-05	0.0002677
Lymph-BNHL	0e7f46ca-6f5c-4538-b6d6-00af6	SBS3	SBS9	4391	5501	0.067267	5.06E-10	8.88E-09
Lymph-BNHL	0e7f46ca-6f5c-4538-b6d6-00af6	SBS6	SBS17	3494	37873	0.067326	5.09E-13	1.15E-11
Lymph-BNHL	0e7f46ca-6f5c-4538-b6d6-00af6	SBS9	SBS17	5501	37873	0.0798644	0	0
Kidney-RCC	b7fbd99c-dea0-4448-a430-7f94	SBS1	SBS3	328	1304	0.1183226	0.0013003	0.0082562
Kidney-RCC	b7fbd99c-dea0-4448-a430-7f94	SBS1	SBS9	328	772	0.1233887	0.0018067	0.0109189
Skin-Melanoma	25103371-28ac-4f43-b0e2-2d37	SBS7	SBS11	312810	5008	0.0526952	2.59E-12	5.48E-11
Panc-AdenoCA	1327bbd4-a466-4e26-905f-cf91	SBS1	SBS7	3459	274	0.1061465	0.0065518	0.0320783
Panc-AdenoCA	1327bbd4-a466-4e26-905f-cf91	SBS1	SBS16	3459	676	0.0697396	0.0081686	0.0386358
Skin-Melanoma	eb9a8f12-6451-43cc-95b3-2e86	SBS3	SBS14	1876	829	0.0741221	0.0036089	0.0197002
Skin-Melanoma	eb9a8f12-6451-43cc-95b3-2e86	SBS3	SBS17	1876	11546	0.0415192	0.007668	0.0367678
Skin-Melanoma	eb9a8f12-6451-43cc-95b3-2e86	SBS4	SBS6	2870	1291	0.0645263	0.0012042	0.0077246
Skin-Melanoma	eb9a8f12-6451-43cc-95b3-2e86	SBS6	SBS14	1291	829	0.0970998	0.0001468	0.0011965
Skin-Melanoma	eb9a8f12-6451-43cc-95b3-2e86	SBS6	SBS17	1291	11546	0.065208	0.0001029	0.0008662
Ovary-AdenoCA	acd510de-b732-4a1a-8b72-6d22	SBS3	SBS12	2365	1790	0.051836	0.0083781	0.0393843
Lymph-BNHL	fce8d8c6-f2a0-43a8-9a7a-b9c51	SBS1	SBS3	1402	11644	0.0512594	0.0027873	0.015794
Lymph-BNHL	fce8d8c6-f2a0-43a8-9a7a-b9c51	SBS1	SBS8	1402	3393	0.0609808	0.0012492	0.0079806
Lymph-BNHL	fce8d8c6-f2a0-43a8-9a7a-b9c51	SBS1	SBS9	1402	1210	0.068845	0.0042393	0.022382
Lymph-BNHL	fce8d8c6-f2a0-43a8-9a7a-b9c51	SBS2	SBS9	8049	1210	0.0561838	0.0026122	0.0149366
Lymph-BNHL	fce8d8c6-f2a0-43a8-9a7a-b9c51	SBS3	SBS17	11644	6381	0.0300398	0.0011752	0.0075625
Lymph-BNHL	fce8d8c6-f2a0-43a8-9a7a-b9c51	SBS8	SBS17	3393	6381	0.0404957	0.0013989	0.0087792
Lymph-BNHL	fce8d8c6-f2a0-43a8-9a7a-b9c51	SBS9	SBS11	1210	5499	0.0578911	0.0025943	0.0148478
Lymph-BNHL	fce8d8c6-f2a0-43a8-9a7a-b9c51	SBS9	SBS17	1210	6381	0.0696848	0.0001026	0.000865
Lymph-BNHL	2b02e3ad-4423-491e-993a-561	SBS6	SBS12	1020	726	0.0825717	0.0061563	0.0305325
Lymph-BNHL	2b02e3ad-4423-491e-993a-561	SBS6	SBS17	1020	1619	0.089966	7.98E-05	0.0006885
Lymph-BNHL	2b02e3ad-4423-491e-993a-561	SBS9	SBS17	1723	1619	0.0659213	0.001414	0.0088436
Liver-HCC	8d7592e2-c622-11e3-bf01-24c6	SBS4	SBS16	2833	2186	0.0495915	0.0046265	0.0241019
Eso-AdenoCa	ce799e7b-30e7-44a5-a185-3e5	SBS1	SBS8	2088	2992	0.0470296	0.0086787	0.0404683
Eso-AdenoCa	ce799e7b-30e7-44a5-a185-3e5	SBS1	SBS17	2088	3934	0.0461781	0.0059508	0.0297573
Eso-AdenoCa	ce799e7b-30e7-44a5-a185-3e5	SBS3	SBS17	709	3934	0.0790738	0.0010926	0.0071103
Eso-AdenoCa	ce799e7b-30e7-44a5-a185-3e5	SBS8	SBS17	2992	3934	0.0826389	1.66E-10	3.02E-09
Liver-HCC	d2b7bb0a-c622-11e3-bf01-24c6	SBS4	SBS12	2688	5037	0.0444849	0.0019428	0.0116179
Eso-AdenoCa	a284fe9d-9afd-4472-8d70-243c	SBS1	SBS2	5563	5362	0.0931861	0	0
Eso-AdenoCa	a284fe9d-9afd-4472-8d70-243c	SBS1	SBS8	5563	2458	0.0469748	0.0010804	0.0070533
Eso-AdenoCa	a284fe9d-9afd-4472-8d70-243c	SBS1	SBS13	5563	2942	0.0876956	2.80E-13	6.42E-12
Eso-AdenoCa	a284fe9d-9afd-4472-8d70-243c	SBS2	SBS8	5362	2458	0.0566289	4.04E-05	0.0003735
Eso-AdenoCa	a284fe9d-9afd-4472-8d70-243c	SBS2	SBS17	5362	12587	0.0783793	0	0
Eso-AdenoCa	a284fe9d-9afd-4472-8d70-243c	SBS8	SBS13	2458	2942	0.0479631	0.0042189	0.0222931
Eso-AdenoCa	a284fe9d-9afd-4472-8d70-243c	SBS8	SBS17	2458	12587	0.0359559	0.0098131	0.0446256
Eso-AdenoCa	a284fe9d-9afd-4472-8d70-243c	SBS13	SBS17	2942	12587	0.072773	2.15E-11	4.13E-10
Prost-AdenoCA	48c33a30-557b-4ecf-8066-5b4b	SBS1	SBS3	935	485	0.1373394	1.17E-05	0.0001193
Prost-AdenoCA	48c33a30-557b-4ecf-8066-5b4b	SBS1	SBS5	935	1380	0.157196	2.18E-12	4.64E-11
Panc-AdenoCA	ec77847e-48fd-4ba5-bc3e-3cd1	SBS1	SBS17	5589	396	0.1245059	2.10E-05	0.0002042
Panc-AdenoCA	ec77847e-48fd-4ba5-bc3e-3cd1	SBS6	SBS17	71	396	0.2417129	0.001761	0.0106938
Panc-AdenoCA	96ba50eb-3c12-41ad-ac7b-d23	SBS1	SBS6	5700	3043	0.0368184	0.0092277	0.0425849
Panc-AdenoCA	96ba50eb-3c12-41ad-ac7b-d23	SBS1	SBS9	5700	1178	0.0737521	4.88E-05	0.0004429
Liver-HCC	030695f6-c623-11e3-bf01-24c65	SBS2	SBS4	1455	4972	0.147178	0	0
Liver-HCC	030695f6-c623-11e3-bf01-24c65	SBS2	SBS9	1455	1157	0.0663572	0.0068557	0.0333835
Liver-HCC	030695f6-c623-11e3-bf01-24c65	SBS2	SBS12	1455	4788	0.1888643	0	0
Liver-HCC	030695f6-c623-11e3-bf01-24c65	SBS3	SBS4	246	4972	0.1992344	1.66E-08	2.51E-07
Liver-HCC	030695f6-c623-11e3-bf01-24c65	SBS3	SBS9	246	1157	0.1188032	0.0065163	0.0319292
Liver-HCC	030695f6-c623-11e3-bf01-24c65	SBS3	SBS12	246	4788	0.2338927	1.52E-11	2.96E-10
Liver-HCC	030695f6-c623-11e3-bf01-24c65	SBS4	SBS9	4972	1157	0.0921666	2.38E-07	3.19E-06
Liver-HCC	030695f6-c623-11e3-bf01-24c65	SBS4	SBS12	4972	4788	0.0500724	9.75E-06	0.0001005
Liver-HCC	030695f6-c623-11e3-bf01-24c65	SBS9	SBS12	1157	4788	0.1354997	2.78E-15	7.77E-14
Biliary-AdenoCA	e4fd1b3e-c622-11e3-bf01-24c65	SBS5	SBS8	2081	609	0.0773367	0.0071373	0.03454
Panc-AdenoCA	0554ffe5-31f7-43f5-8372-2b73c	SBS1	SBS2	955	703	0.1117589	8.09E-05	0.0006977
Panc-AdenoCA	0554ffe5-31f7-43f5-8372-2b73c	SBS1	SBS8	955	1891	0.1308391	7.34E-10	1.27E-08
Panc-AdenoCA	0554ffe5-31f7-43f5-8372-2b73c	SBS1	SBS12	955	397	0.1381592	4.48E-05	0.0004091
Lymph-BNHL	db9ce6c6-529c-4da6-92c4-f4f3f	SBS1	SBS5	290	1853	0.1166422	0.0021763	0.0128066
Lymph-BNHL	db9ce6c6-529c-4da6-92c4-f4f3f	SBS1	SBS9	290	1359	0.1329883	0.0004262	0.0031311
Kidney-RCC	a89a2341-263d-4e7f-87fa-18d9	SBS1	SBS3	550	3135	0.1265391	6.21E-07	7.77E-06

Kidney-RCC	a89a2341-263d-4e7f-87fa-18d9(	SBS1	SBS4	550	1869	0.1396556	1.27E-07	1.74E-06
Kidney-RCC	a89a2341-263d-4e7f-87fa-18d9(	SBS1	SBS16	550	1097	0.1046739	0.0006527	0.0045249
Panc-AdenoCA	2102558b-87b0-447b-bb9a-e4ae	SBS1	SBS9	4359	578	0.0797233	0.0030459	0.017028
Lymph-CLL	ffa976f0-aa60-4867-842e-361af	SBS5	SBS9	1446	754	0.1891232	7.77E-16	2.29E-14
Lymph-CLL	4808f9e1-452d-45eb-9b87-2f87(	SBS3	SBS9	1862	156	0.3133176	1.07E-12	2.33E-11
Lymph-CLL	4808f9e1-452d-45eb-9b87-2f87(	SBS3	SBS16	1862	128	0.1612094	0.0039582	0.0211295
Lymph-CLL	4808f9e1-452d-45eb-9b87-2f87(	SBS9	SBS12	156	468	0.2991453	1.61E-09	2.72E-08
Panc-Endocrine	d515c7d2-2314-46bf-9736-ff3f3(	SBS2	SBS3	3181	1083	0.2381972	0	0
Panc-Endocrine	d515c7d2-2314-46bf-9736-ff3f3(	SBS2	SBS16	3181	250	0.4155322	0	0
Panc-Endocrine	d515c7d2-2314-46bf-9736-ff3f3(	SBS3	SBS13	1083	1497	0.2490466	0	0
Panc-Endocrine	d515c7d2-2314-46bf-9736-ff3f3(	SBS3	SBS16	1083	250	0.1996122	1.87E-07	2.53E-06
Panc-Endocrine	d515c7d2-2314-46bf-9736-ff3f3(	SBS13	SBS16	1497	250	0.4245157	0	0
Ovary-AdenoCA	bbb2cf2f-8f32-43d4-846c-d1020	SBS3	SBS6	2799	1518	0.0551474	0.0050249	0.0258738
Panc-AdenoCA	fa9a3247-a465-4fdf-bb64-5afaa8	SBS1	SBS2	1550	338	0.1042565	0.0047999	0.0249116
Panc-AdenoCA	fa9a3247-a465-4fdf-bb64-5afaa8	SBS1	SBS12	1550	293	0.1273698	0.0006741	0.004637
Panc-AdenoCA	fa9a3247-a465-4fdf-bb64-5afaa8	SBS1	SBS15	1550	118	0.1661017	0.0047131	0.0245322
Panc-AdenoCA	fa9a3247-a465-4fdf-bb64-5afaa8	SBS5	SBS12	86	293	0.2026748	0.0084896	0.0398236
Skin-Melanoma	9e0009d1-c993-4247-9706-88ee	SBS1	SBS3	722	11648	0.0828987	0.0001749	0.0013895
Skin-Melanoma	9e0009d1-c993-4247-9706-88ee	SBS1	SBS12	722	909	0.1085269	0.0001529	0.0012365
Eso-AdenoCa	48a0da38-8147-40fe-b683-3f07(	SBS1	SBS17	3427	4156	0.0659147	1.63E-07	2.23E-06
Eso-AdenoCa	48a0da38-8147-40fe-b683-3f07(	SBS5	SBS17	7376	4156	0.0608491	5.65E-09	8.90E-08
Eso-AdenoCa	384ef419-ee94-4563-9237-236f(	SBS1	SBS17	4744	2891	0.0492378	0.0003299	0.002488
Eso-AdenoCa	384ef419-ee94-4563-9237-236f(	SBS5	SBS17	362	2891	0.0929729	0.0076838	0.036815
Eso-AdenoCa	384ef419-ee94-4563-9237-236f(	SBS7	SBS17	1249	2891	0.0892242	1.86E-06	2.18E-05
Stomach-AdenoCA	f50fb278-1f0f-406e-b341-eb3d1l	SBS12	SBS17	7192	13089	0.0315354	0.0001957	0.0015352
Liver-HCC	96abdbc8-c622-11e3-bf01-24c6!	SBS4	SBS7	11320	762	0.1208613	1.75E-09	2.91E-08
Liver-HCC	96abdbc8-c622-11e3-bf01-24c6!	SBS4	SBS10	11320	1354	0.1184102	3.77E-15	1.04E-13
Liver-HCC	96abdbc8-c622-11e3-bf01-24c6!	SBS4	SBS14	11320	514	0.1821854	1.33E-14	3.45E-13
Liver-HCC	96abdbc8-c622-11e3-bf01-24c6!	SBS10	SBS14	1354	514	0.1025898	0.0007855	0.0052651
Panc-AdenoCA	241abdfd-e6de-4830-a233-4bbc	SBS1	SBS2	3378	2235	0.1581206	0	0
Panc-AdenoCA	241abdfd-e6de-4830-a233-4bbc	SBS1	SBS5	3378	1076	0.1900137	0	0
Panc-AdenoCA	241abdfd-e6de-4830-a233-4bbc	SBS1	SBS8	3378	3046	0.1024634	5.00E-15	1.36E-13
Panc-AdenoCA	241abdfd-e6de-4830-a233-4bbc	SBS1	SBS13	3378	1254	0.10363	5.90E-09	9.24E-08
Panc-AdenoCA	241abdfd-e6de-4830-a233-4bbc	SBS2	SBS5	2235	1076	0.0617749	0.0078255	0.0373232
Panc-AdenoCA	241abdfd-e6de-4830-a233-4bbc	SBS2	SBS8	2235	3046	0.0609556	0.0001382	0.0011325
Panc-AdenoCA	241abdfd-e6de-4830-a233-4bbc	SBS2	SBS13	2235	1254	0.0777517	0.0001211	0.0010036
Panc-AdenoCA	241abdfd-e6de-4830-a233-4bbc	SBS5	SBS8	1076	3046	0.1088789	1.30E-08	1.99E-07
Panc-AdenoCA	241abdfd-e6de-4830-a233-4bbc	SBS5	SBS13	1076	1254	0.1163592	3.10E-07	4.04E-06
Eso-AdenoCa	eb1531b0-8af1-4b2b-9192-644c	SBS1	SBS17	12643	10894	0.0460644	3.28E-11	6.23E-10
Panc-AdenoCA	bc395326-1656-4ef2-bb19-0cb2!	SBS1	SBS6	40919	7676	0.040893	8.18E-10	1.41E-08
Biliary-AdenoCA	5c9b8a2c-c623-11e3-bf01-24c6!	SBS1	SBS16	1304	879	0.0786606	0.0030139	0.0168947
Biliary-AdenoCA	5c9b8a2c-c623-11e3-bf01-24c6!	SBS8	SBS16	789	879	0.1211799	9.95E-06	0.0001017
Ovary-AdenoCA	e45f3391-2e74-4767-817a-280c	SBS1	SBS5	547	4375	0.0919354	0.000539	0.0038426
Liver-HCC	b32449c0-c622-11e3-bf01-24c6!	SBS12	SBS16	759	10426	0.0678312	0.0029755	0.0167095
Bone-Osteosarc	f87348df-7186-4c6b-e040-11ac(	SBS3	SBS7	3093	1297	0.1047706	3.88E-09	6.24E-08
Bone-Osteosarc	f87348df-7186-4c6b-e040-11ac(	SBS3	SBS17	3093	464	0.1206569	1.58E-05	0.0001568
Bone-Osteosarc	f87348df-7186-4c6b-e040-11ac(	SBS6	SBS7	1362	1297	0.1034365	1.34E-06	1.60E-05
Bone-Osteosarc	f87348df-7186-4c6b-e040-11ac(	SBS6	SBS17	1362	464	0.1184016	0.0001221	0.0010109
Skin-Melanoma	142b6dbf-c943-4a7d-8ab6-13a9	SBS7	SBS11	56235	3982	0.0537366	9.42E-10	1.62E-08
Eso-AdenoCa	2b41746a-95c0-4875-afb5-c6d3(	SBS1	SBS17	14738	7213	0.0233074	0.0103739	0.0468397
Prost-AdenoCA	1eb37b28-fac2-477a-88b3-e042	SBS1	SBS5	319	2670	0.1212344	0.0004605	0.0033478
Prost-AdenoCA	1eb37b28-fac2-477a-88b3-e042	SBS1	SBS8	319	1420	0.1192724	0.0012089	0.0077385
Prost-AdenoCA	1eb37b28-fac2-477a-88b3-e042	SBS1	SBS9	319	137	0.2122966	0.0003541	0.0026547
Prost-AdenoCA	1eb37b28-fac2-477a-88b3-e042	SBS1	SBS15	319	354	0.1364965	0.0038514	0.0207537
Prost-AdenoCA	1eb37b28-fac2-477a-88b3-e042	SBS3	SBS9	248	137	0.1918413	0.0030198	0.0169128
Panc-AdenoCA	cb4608a7-6aec-4cba-b20f-489e.	SBS1	SBS5	932	2075	0.1438544	5.51E-12	1.12E-10
Panc-AdenoCA	cb4608a7-6aec-4cba-b20f-489e.	SBS1	SBS6	932	271	0.1514063	0.000132	0.0010885
Panc-AdenoCA	cb4608a7-6aec-4cba-b20f-489e.	SBS1	SBS7	932	607	0.1185879	6.47E-05	0.0005695
Eso-AdenoCa	3da169e8-844a-4eee-b794-a2el	SBS1	SBS4	1186	2841	0.0565443	0.0094929	0.043584
Eso-AdenoCa	3da169e8-844a-4eee-b794-a2el	SBS1	SBS17	1186	8566	0.0973938	5.22E-09	8.29E-08
Eso-AdenoCa	3da169e8-844a-4eee-b794-a2el	SBS4	SBS17	2841	8566	0.124855	0	0
Eso-AdenoCa	3da169e8-844a-4eee-b794-a2el	SBS9	SBS17	2391	8566	0.095313	3.55E-15	9.90E-14
Bone-Osteosarc	f87d7c27-eeef-920e-e040-11ac(	SBS1	SBS3	364	2585	0.0920484	0.0089709	0.0417068
Liver-HCC	b070af2a-c622-11e3-bf01-24c6!	SBS2	SBS12	983	2440	0.1186994	5.32E-09	8.40E-08

Liver-HCC	b070af2a-c622-11e3-bf01-24c65	SBS3	SBS12	510	2440	0.1063243	0.0001442	0.0011782
Liver-HCC	b070af2a-c622-11e3-bf01-24c65	SBS5	SBS12	2832	2440	0.0775898	2.80E-07	3.73E-06
Liver-HCC	01dc6872-c623-11e3-bf01-24c65	SBS12	SBS16	2837	5770	0.0562702	1.18E-05	0.0001194
Breast-AdenoCa	fca3f7d0-2231-661c-e040-11ac0	SBS1	SBS3	1492	5371	0.0820823	2.94E-07	3.87E-06
Breast-AdenoCa	fca3f7d0-2231-661c-e040-11ac0	SBS1	SBS11	1492	1390	0.0802949	0.0001868	0.0014685
Lymph-BNHL	f9dc999f-6dde-448d-9cf1-2897d	SBS2	SBS15	14237	16946	0.0345701	1.86E-08	2.80E-07
Lymph-BNHL	f9dc999f-6dde-448d-9cf1-2897d	SBS3	SBS15	27691	16946	0.0283423	9.25E-08	1.29E-06
Lymph-BNHL	f9dc999f-6dde-448d-9cf1-2897d	SBS9	SBS15	28300	16946	0.0342688	3.09E-11	5.88E-10
Lymph-BNHL	f9dc999f-6dde-448d-9cf1-2897d	SBS15	SBS17	16946	18163	0.0412531	2.20E-13	5.12E-12
Eso-AdenoCa	36680797-36de-413d-b2a1-8804	SBS1	SBS17	3644	3149	0.1131445	0	0
Eso-AdenoCa	36680797-36de-413d-b2a1-8804	SBS5	SBS17	2081	3149	0.1186696	8.88E-16	2.59E-14
Eso-AdenoCa	36680797-36de-413d-b2a1-8804	SBS8	SBS17	2404	3149	0.1339646	0	0
Eso-AdenoCa	36680797-36de-413d-b2a1-8804	SBS16	SBS17	308	3149	0.17922	2.98E-08	4.38E-07
Ovary-AdenoCA	123c9d1c-72ba-4d82-8e57-d5ca	SBS1	SBS3	797	5827	0.1064872	2.49E-07	3.34E-06
Ovary-AdenoCA	123c9d1c-72ba-4d82-8e57-d5ca	SBS1	SBS13	797	769	0.1420052	2.79E-07	3.72E-06
Ovary-AdenoCA	f6c811ff-f22e-490b-9b23-b527d2	SBS1	SBS3	378	4656	0.1011189	0.0015702	0.0096857
Ovary-AdenoCA	f6c811ff-f22e-490b-9b23-b527d2	SBS1	SBS13	378	596	0.1191275	0.0028177	0.0159374
Liver-HCC	9ba2c970-c622-11e3-bf01-24c65	SBS4	SBS12	2491	4767	0.0443666	0.0031901	0.0177552
Prost-AdenoCA	36962459-e81f-4b8c-a08d-df60e	SBS3	SBS9	637	643	0.0949459	0.0062439	0.0308356
Panc-AdenoCA	03c3c692-8a86-4843-85ae-e045	SBS1	SBS3	2014	12422	0.1786671	0	0
Panc-AdenoCA	441d2f21-b448-4fc8-8c54-9b85a	SBS1	SBS3	3299	352	0.0967339	0.0051986	0.0265957
Panc-AdenoCA	441d2f21-b448-4fc8-8c54-9b85a	SBS1	SBS6	3299	320	0.1131138	0.0011461	0.0073969
Panc-AdenoCA	441d2f21-b448-4fc8-8c54-9b85a	SBS1	SBS7	3299	856	0.0790334	0.0004108	0.0030323
Panc-AdenoCA	441d2f21-b448-4fc8-8c54-9b85a	SBS6	SBS8	320	603	0.1188796	0.0054301	0.0275997
Liver-HCC	5b178f44-c622-11e3-bf01-24c65	SBS4	SBS12	1907	4374	0.0693641	5.64E-06	6.04E-05
Lymph-BNHL	f9837a56-7244-4846-a63d-266e	SBS5	SBS8	2112	155	0.1427878	0.0055437	0.0279727
Liver-HCC	145f6dbb-9744-44f7-8485-e4406	SBS5	SBS6	2513	634	0.0812287	0.0025093	0.0144407
Skin-Melanoma	3433b1f3-59d1-4ac2-9bbf-e0c40	SBS3	SBS7	1819	2485	0.0529997	0.005478	0.0277758
Skin-Melanoma	3433b1f3-59d1-4ac2-9bbf-e0c40	SBS5	SBS7	6458	2485	0.0449995	0.0013957	0.0087792
Skin-Melanoma	3433b1f3-59d1-4ac2-9bbf-e0c40	SBS7	SBS13	2485	931	0.0665769	0.0049381	0.0255202
Stomach-AdenoCA	b49d5310-3cc5-4386-9444-cf0d0	SBS1	SBS3	4682	1762	0.0836946	3.25E-08	4.75E-07
Stomach-AdenoCA	b49d5310-3cc5-4386-9444-cf0d0	SBS1	SBS9	4682	2254	0.054347	0.0002498	0.0019235
Stomach-AdenoCA	b49d5310-3cc5-4386-9444-cf0d0	SBS3	SBS15	1762	699	0.0905485	0.0005457	0.0038817
Stomach-AdenoCA	b49d5310-3cc5-4386-9444-cf0d0	SBS3	SBS17	1762	4446	0.084204	3.38E-08	4.93E-07
Stomach-AdenoCA	b49d5310-3cc5-4386-9444-cf0d0	SBS9	SBS17	2254	4446	0.0562964	0.0001526	0.0012365
Kidney-RCC	45348eff-4b09-4776-825e-b18de	SBS1	SBS4	379	2099	0.1438667	3.39E-06	3.80E-05
Kidney-RCC	45348eff-4b09-4776-825e-b18de	SBS1	SBS5	379	539	0.1775985	1.60E-06	1.90E-05
Kidney-RCC	45348eff-4b09-4776-825e-b18de	SBS1	SBS8	379	182	0.196396	0.0001519	0.0012335
Eso-AdenoCa	a37a825c-d74e-46cc-92ef-b6501	SBS1	SBS2	2547	2122	0.1454436	0	0
Eso-AdenoCa	a37a825c-d74e-46cc-92ef-b6501	SBS1	SBS17	2547	10103	0.1537235	0	0
Eso-AdenoCa	a37a825c-d74e-46cc-92ef-b6501	SBS2	SBS3	2122	1418	0.1171141	1.50E-10	2.74E-09
Eso-AdenoCa	a37a825c-d74e-46cc-92ef-b6501	SBS2	SBS8	2122	4116	0.1317724	0	0
Eso-AdenoCa	a37a825c-d74e-46cc-92ef-b6501	SBS2	SBS9	2122	317	0.2100393	5.40E-11	1.02E-09
Eso-AdenoCa	a37a825c-d74e-46cc-92ef-b6501	SBS2	SBS10	2122	975	0.1233935	2.92E-09	4.80E-08
Eso-AdenoCa	a37a825c-d74e-46cc-92ef-b6501	SBS2	SBS15	2122	1858	0.1552895	0	0
Eso-AdenoCa	a37a825c-d74e-46cc-92ef-b6501	SBS2	SBS17	2122	10103	0.2919096	0	0
Eso-AdenoCa	a37a825c-d74e-46cc-92ef-b6501	SBS3	SBS9	1418	317	0.1116982	0.0031145	0.0173651
Eso-AdenoCa	a37a825c-d74e-46cc-92ef-b6501	SBS3	SBS17	1418	10103	0.1896741	0	0
Eso-AdenoCa	a37a825c-d74e-46cc-92ef-b6501	SBS8	SBS17	4116	10103	0.1636088	0	0
Eso-AdenoCa	a37a825c-d74e-46cc-92ef-b6501	SBS9	SBS10	317	975	0.1050392	0.0101972	0.0462024
Eso-AdenoCa	a37a825c-d74e-46cc-92ef-b6501	SBS9	SBS17	317	10103	0.0967558	0.0063355	0.0311651
Eso-AdenoCa	a37a825c-d74e-46cc-92ef-b6501	SBS10	SBS17	975	10103	0.1851015	0	0
Eso-AdenoCa	a37a825c-d74e-46cc-92ef-b6501	SBS15	SBS17	1858	10103	0.1550152	0	0
Lymph-CLL	ebc1a26b-9582-4756-acd5-b02d	SBS3	SBS9	1031	1520	0.147944	4.19E-12	8.64E-11
Lymph-CLL	ebc1a26b-9582-4756-acd5-b02d	SBS6	SBS9	576	1520	0.1156433	2.81E-05	0.0002682
Breast-AdenoCa	fc7eb420-5c13-20bd-e040-11ac0	SBS1	SBS7	349	463	0.1603471	7.19E-05	0.0006269
Breast-AdenoCa	fc7eb420-5c13-20bd-e040-11ac0	SBS1	SBS13	349	1396	0.2979943	0	0
Breast-AdenoCa	fc7eb420-5c13-20bd-e040-11ac0	SBS3	SBS7	4323	463	0.125237	4.01E-06	4.42E-05
Breast-AdenoCa	fc7eb420-5c13-20bd-e040-11ac0	SBS3	SBS13	4323	1396	0.2635107	0	0
Breast-AdenoCa	fc7eb420-5c13-20bd-e040-11ac0	SBS7	SBS13	463	1396	0.1539496	1.39E-07	1.91E-06
Panc-AdenoCA	ffe4bb51-e98a-41a7-a4e1-c3970	SBS1	SBS2	1641	689	0.1981729	1.11E-16	3.39E-15
Panc-AdenoCA	ffe4bb51-e98a-41a7-a4e1-c3970	SBS1	SBS7	1641	341	0.1802867	2.14E-08	3.18E-07
Panc-AdenoCA	ffe4bb51-e98a-41a7-a4e1-c3970	SBS2	SBS8	689	707	0.1841629	1.05E-10	1.97E-09
Panc-AdenoCA	ffe4bb51-e98a-41a7-a4e1-c3970	SBS7	SBS8	341	707	0.1719338	2.48E-06	2.88E-05

Kidney-RCC	a335b03d-41ac-4d41-a2a9-3134	SBS1	SBS2	273	475	0.1311047	0.0051616	0.0264279
Kidney-RCC	a335b03d-41ac-4d41-a2a9-3134	SBS1	SBS3	273	132	0.1728272	0.0098307	0.044644
Kidney-RCC	a335b03d-41ac-4d41-a2a9-3134	SBS1	SBS8	273	2022	0.1099553	0.0059591	0.0297573
Kidney-RCC	a335b03d-41ac-4d41-a2a9-3134	SBS1	SBS9	273	779	0.1561502	0.0001046	0.000879
Kidney-RCC	a335b03d-41ac-4d41-a2a9-3134	SBS1	SBS15	273	541	0.1296338	0.0044938	0.0234885
Kidney-RCC	a335b03d-41ac-4d41-a2a9-3134	SBS8	SBS9	2022	779	0.0711106	0.0067776	0.0330548
Breast-AdenoCa	f7f3e156-0dde-72b9-e040-11ac(	SBS1	SBS2	686	2762	0.1465527	1.12E-10	2.09E-09
Breast-AdenoCa	f7f3e156-0dde-72b9-e040-11ac(	SBS1	SBS13	686	2898	0.1776172	1.22E-15	3.51E-14
Breast-AdenoCa	f7f3e156-0dde-72b9-e040-11ac(	SBS2	SBS3	2762	2532	0.1263866	0	0
Breast-AdenoCa	f7f3e156-0dde-72b9-e040-11ac(	SBS2	SBS13	2762	2898	0.0431127	0.0104208	0.0469497
Breast-AdenoCa	f7f3e156-0dde-72b9-e040-11ac(	SBS3	SBS13	2532	2898	0.1558642	0	0
Panc-AdenoCA	e56b0990-ff67-47c1-b9ad-87ef1	SBS1	SBS3	6550	1211	0.0514119	0.0090072	0.0418132
Prost-AdenoCA	43b675e0-22e0-42d6-a060-afc9	SBS1	SBS8	1494	1766	0.0583618	0.0080656	0.0382933
Eso-AdenoCa	3e6a17f9-c249-41b6-ba49-55aa	SBS5	SBS17	3945	37369	0.0288041	0.0053646	0.0273335
Biliary-AdenoCA	5a51a6fc-c623-11e3-bf01-24c65	SBS1	SBS14	6440	735	0.0705117	0.0028318	0.0160025
Biliary-AdenoCA	5a51a6fc-c623-11e3-bf01-24c65	SBS6	SBS14	975	735	0.1215071	8.45E-06	8.84E-05
Lymph-CLL	55c82198-5be6-4cc5-b5b3-b707	SBS5	SBS9	1877	780	0.2604688	0	0
Lymph-CLL	55c82198-5be6-4cc5-b5b3-b707	SBS8	SBS9	171	780	0.231534	5.89E-07	7.42E-06
Lymph-CLL	55c82198-5be6-4cc5-b5b3-b707	SBS9	SBS14	780	136	0.2354827	5.29E-06	5.70E-05
Panc-AdenoCA	3c86ba21-7b11-4ec7-9d20-a232	SBS1	SBS2	4720	2955	0.1429852	0	0
Panc-AdenoCA	3c86ba21-7b11-4ec7-9d20-a232	SBS1	SBS13	4720	2139	0.1196919	0	0
Lymph-BNHL	56e57223-264e-489d-bc9a-a077	SBS3	SBS6	2734	811	0.0722617	0.002912	0.016402
Skin-Melanoma	a6e24c2a-26b9-409c-bbcd-a8fc(	SBS2	SBS7	264	10295	0.1370149	0.000127	0.0010487
Skin-Melanoma	a6e24c2a-26b9-409c-bbcd-a8fc(	SBS7	SBS12	10295	1041	0.0653686	0.0006196	0.004334
Breast-AdenoCa	fc8130e0-a8b4-d80d-e040-11ac	SBS7	SBS13	688	1381	0.0804513	0.0052403	0.0267651
Breast-AdenoCa	fc8130e0-a8b4-d80d-e040-11ac	SBS13	SBS16	1381	1996	0.0661007	0.0015968	0.0098207
Prost-AdenoCA	0bfd1068-3fd3-a95b-e050-11ac(	SBS1	SBS3	3050	2068	0.0516793	0.0027678	0.0156977
Breast-AdenoCa	fc68c24d-47ad-7961-e040-11ac(	SBS1	SBS3	363	5972	0.1561022	1.14E-07	1.59E-06
Breast-AdenoCa	fc68c24d-47ad-7961-e040-11ac(	SBS1	SBS9	363	54	0.2757882	0.0015687	0.0096857
Panc-AdenoCA	c9e7c629-7b57-4ede-b315-0cea	SBS3	SBS15	795	157	0.1449505	0.0080976	0.038416
Lymph-CLL	de99a4de-e916-4572-ac9e-73e3	SBS1	SBS9	162	2898	0.1432789	0.0036755	0.0199662
Lymph-CLL	de99a4de-e916-4572-ac9e-73e3	SBS7	SBS9	314	2898	0.2689643	0	0
Lymph-CLL	de99a4de-e916-4572-ac9e-73e3	SBS7	SBS17	314	12	0.4771762	0.0103535	0.0467812
Lymph-CLL	de99a4de-e916-4572-ac9e-73e3	SBS9	SBS15	2898	327	0.1857086	3.15E-09	5.11E-08
Panc-AdenoCA	3ed783cf-2248-44a1-a2a2-d6b6	SBS1	SBS3	688	8344	0.0671211	0.0065137	0.0319292
Stomach-AdenoCA	eda1d8d9-e8f5-46f2-bb16-15d0k	SBS1	SBS7	2233	1162	0.0721165	0.0007055	0.0048162
Stomach-AdenoCA	eda1d8d9-e8f5-46f2-bb16-15d0k	SBS1	SBS16	2233	2386	0.0550562	0.0018369	0.0110689
Breast-AdenoCa	fc68e599-6a40-61dc-e040-11ac(	SBS1	SBS3	2132	4691	0.0460166	0.0040274	0.021444
Breast-AdenoCa	fc447d4f-2532-c8ea-e040-11ac0	SBS3	SBS13	5723	1893	0.1053054	3.97E-14	9.88E-13
Breast-AdenoCa	fc447d4f-2532-c8ea-e040-11ac0	SBS3	SBS14	5723	1068	0.0624262	0.0017967	0.0108686
Breast-AdenoCa	fc447d4f-2532-c8ea-e040-11ac0	SBS4	SBS13	478	1893	0.1610613	5.04E-09	8.01E-08
Breast-AdenoCa	fc447d4f-2532-c8ea-e040-11ac0	SBS11	SBS13	1030	1893	0.1129711	8.06E-08	1.14E-06
Breast-AdenoCa	fc447d4f-2532-c8ea-e040-11ac0	SBS13	SBS14	1893	1068	0.1618159	5.55E-16	1.65E-14
Prost-AdenoCA	08e1d976-6c39-428e-a4c2-f655l	SBS1	SBS5	521	1872	0.1016485	0.0004398	0.0032199
Breast-AdenoCa	fc8130e0-0f1a-b6eb-e040-11ac(	SBS1	SBS2	586	996	0.1664234	2.66E-09	4.38E-08
Breast-AdenoCa	fc8130e0-0f1a-b6eb-e040-11ac(	SBS1	SBS4	586	1797	0.0921302	0.0011044	0.0071572
Breast-AdenoCa	fc8130e0-0f1a-b6eb-e040-11ac(	SBS1	SBS8	586	3085	0.1026264	6.25E-05	0.0005533
Breast-AdenoCa	fc8130e0-0f1a-b6eb-e040-11ac(	SBS1	SBS13	586	1358	0.1562024	4.22E-09	6.79E-08
Breast-AdenoCa	fc8130e0-0f1a-b6eb-e040-11ac(	SBS2	SBS4	996	1797	0.0935562	2.69E-05	0.0002578
Breast-AdenoCa	fc8130e0-0f1a-b6eb-e040-11ac(	SBS2	SBS8	996	3085	0.0730894	0.0006419	0.0044695
Breast-AdenoCa	fc8130e0-0f1a-b6eb-e040-11ac(	SBS4	SBS13	1797	1358	0.0925376	3.53E-06	3.95E-05
Breast-AdenoCa	fc8130e0-0f1a-b6eb-e040-11ac(	SBS8	SBS13	3085	1358	0.0761906	3.52E-05	0.0003293
Ovary-AdenoCA	0ead45d8-d785-4404-8319-2ef9	SBS3	SBS6	6503	1789	0.0749273	2.88E-07	3.81E-06
Ovary-AdenoCA	0ead45d8-d785-4404-8319-2ef9	SBS3	SBS12	6503	2113	0.0772417	1.09E-08	1.67E-07
Ovary-AdenoCA	0ead45d8-d785-4404-8319-2ef9	SBS6	SBS8	1789	1734	0.066956	0.0007452	0.005054
Ovary-AdenoCA	0ead45d8-d785-4404-8319-2ef9	SBS8	SBS12	1734	2113	0.0759518	3.38E-05	0.0003181
Panc-AdenoCA	25c32aca-6738-43ef-a103-243f2	SBS1	SBS3	3753	792	0.1440545	3.26E-12	6.83E-11
Panc-AdenoCA	25c32aca-6738-43ef-a103-243f2	SBS1	SBS9	3753	204	0.118517	0.0087188	0.0406248
Panc-AdenoCA	88d5a8b2-daba-45ce-90bf-480fa	SBS1	SBS3	3249	1578	0.0579863	0.0015813	0.0097444
Liver-HCC	a94d63fa-c622-11e3-bf01-24c65	SBS3	SBS12	276	4896	0.1026392	0.0081336	0.0385332
Liver-HCC	a94d63fa-c622-11e3-bf01-24c65	SBS4	SBS12	5446	4896	0.0863239	0	0
Skin-Melanoma	98e8f23c-5970-4fce-9551-4b11a	SBS7	SBS11	424954	36672	0.0224233	3.66E-15	1.02E-13
Breast-AdenoCa	f393bafd-1baa-e5f4-e040-11ac0	SBS1	SBS2	268	549	0.1309233	0.0041668	0.0220368
Breast-AdenoCa	f393bafd-1baa-e5f4-e040-11ac0	SBS2	SBS3	549	4204	0.1008546	0.0001025	0.000865

Bone-Epith	fc968a86-32e3-ee88-e040-11ac	SBS2	SBS8	854	1596	0.0753215	0.0036275	0.0197509
Bone-Epith	fc968a86-32e3-ee88-e040-11ac	SBS2	SBS16	854	1156	0.0805896	0.0033909	0.0186392
Skin-Melanoma	d2620dab-4319-499a-b8f1-9575	SBS7	SBS11	47018	3226	0.0396484	0.000151	0.0012275
Skin-Melanoma	1daefc49-5248-4948-87ce-0ec1	SBS7	SBS9	97828	12364	0.0187656	0.0008781	0.0058052
Breast-AdenoCa	fc8130df-6bec-7627-e040-11ac0	SBS1	SBS13	387	1153	0.0958381	0.0097599	0.0445011
Prost-AdenoCA	f640d377-98e9-41d3-8761-61eb	SBS1	SBS5	3870	2896	0.1137072	0	0
Prost-AdenoCA	f640d377-98e9-41d3-8761-61eb	SBS1	SBS9	3870	531	0.1499058	1.54E-09	2.61E-08
Liver-HCC	d0469256-c622-11e3-bf01-24c6	SBS4	SBS12	5714	12350	0.069306	1.11E-16	3.39E-15
Ovary-AdenoCA	ae82fead-2671-4335-a342-67bb	SBS10	SBS16	767	2508	0.0825738	0.0006643	0.0045902
Bone-Osteosarc	f82d213f-9ba5-7b6b-e040-11ac0	SBS8	SBS12	2851	1508	0.0708902	9.90E-05	0.0008387
Panc-AdenoCA	2e43e0ca-54ea-482e-acf2-0048	SBS1	SBS8	3396	3282	0.0669736	6.29E-07	7.83E-06
Panc-Endocrine	dc4ba4bc-6333-4fe9-8805-e058	SBS1	SBS3	3212	3697	0.0737984	1.48E-08	2.26E-07
Panc-Endocrine	dc4ba4bc-6333-4fe9-8805-e058	SBS1	SBS4	3212	1518	0.0699145	8.40E-05	0.0007214
Breast-AdenoCa	f393bb05-ec1c-17be-e040-11ac	SBS1	SBS2	1082	1487	0.0652979	0.0095845	0.043844
Breast-AdenoCa	f393bb05-ec1c-17be-e040-11ac	SBS1	SBS8	1082	2655	0.0621685	0.0052531	0.0268087
Lymph-BNHL	9e842227-2bc6-4185-bedb-abel	SBS1	SBS17	1373	1031	0.1468772	1.85E-11	3.58E-10
Lymph-BNHL	9e842227-2bc6-4185-bedb-abel	SBS5	SBS17	2495	1031	0.1468504	4.33E-14	1.07E-12
Lymph-BNHL	9e842227-2bc6-4185-bedb-abel	SBS9	SBS17	1523	1031	0.1515457	1.09E-12	2.37E-11
Lymph-BNHL	9e842227-2bc6-4185-bedb-abel	SBS15	SBS17	368	1031	0.1280416	0.0002748	0.002098
Lymph-BNHL	d733cf1a-4c42-4def-b6cb-5ef2c	SBS1	SBS9	2752	10904	0.044582	0.0003217	0.0024293
Lymph-BNHL	d733cf1a-4c42-4def-b6cb-5ef2c	SBS1	SBS11	2752	2265	0.0571301	0.0006009	0.0042218
Lymph-BNHL	d733cf1a-4c42-4def-b6cb-5ef2c	SBS1	SBS17	2752	8271	0.0605361	5.35E-07	6.80E-06
Breast-AdenoCa	fc8130df-2e39-3814-e040-11ac0	SBS1	SBS2	920	383	0.1435861	2.87E-05	0.0002728
Breast-AdenoCa	fc8130df-2e39-3814-e040-11ac0	SBS2	SBS5	383	1323	0.1539463	1.54E-06	1.83E-05
Breast-AdenoCa	f7d7b3db-02af-61cb-e040-11ac0	SBS2	SBS3	1974	879	0.2330398	0	0
Breast-AdenoCa	f7d7b3db-02af-61cb-e040-11ac0	SBS2	SBS13	1974	324	0.1112237	0.002044	0.0121534
Breast-AdenoCa	f7d7b3db-02af-61cb-e040-11ac0	SBS3	SBS13	879	324	0.3364935	0	0
Panc-AdenoCA	aa4a868a-df23-4eef-a618-e945a	SBS1	SBS12	7909	2959	0.0552996	3.81E-06	4.24E-05
Panc-AdenoCA	aa4a868a-df23-4eef-a618-e945a	SBS1	SBS17	7909	1341	0.0541164	0.0024233	0.0140748
Eso-AdenoCa	9de495d1-55b2-4535-9b0a-a999	SBS1	SBS17	8331	5012	0.1134756	0	0
Eso-AdenoCa	9de495d1-55b2-4535-9b0a-a999	SBS6	SBS17	192	5012	0.1456504	0.0007829	0.0052532
Eso-AdenoCa	9de495d1-55b2-4535-9b0a-a999	SBS8	SBS17	879	5012	0.115111	4.94E-09	7.88E-08
Eso-AdenoCa	9de495d1-55b2-4535-9b0a-a999	SBS13	SBS17	967	5012	0.1266392	1.02E-11	2.02E-10
Eso-AdenoCa	9de495d1-55b2-4535-9b0a-a999	SBS15	SBS17	598	5012	0.1257978	9.07E-08	1.27E-06
Liver-HCC	b5f90cb8-7304-48fb-a1d3-ff459	SBS4	SBS6	1596	12450	0.0451318	0.0062836	0.0310008
Skin-Melanoma	08b5d0e4-4661-460e-a9f7-f2e6	SBS7	SBS11	71044	1198	0.0590813	0.0005359	0.0038249
Myeloid-MPN	f8e61a02-8c9e-aaee-e040-11ac	SBS5	SBS6	466	542	0.1293651	0.0004558	0.0033213
Skin-Melanoma	b3befa40-8f44-4eb6-ada0-ec39	SBS3	SBS7	4397	1353	0.0552505	0.0036119	0.0197002
Eso-AdenoCa	dfde4013-9062-42f5-a42b-626ef	SBS1	SBS2	20624	6066	0.2069893	0	0
Eso-AdenoCa	dfde4013-9062-42f5-a42b-626ef	SBS1	SBS7	20624	851	0.0750418	0.0002011	0.0015758
Eso-AdenoCa	dfde4013-9062-42f5-a42b-626ef	SBS1	SBS8	20624	4402	0.0306275	0.0022145	0.0129948
Eso-AdenoCa	dfde4013-9062-42f5-a42b-626ef	SBS2	SBS6	6066	448	0.2284145	0	0
Eso-AdenoCa	dfde4013-9062-42f5-a42b-626ef	SBS2	SBS7	6066	851	0.1465046	2.44E-14	6.15E-13
Eso-AdenoCa	dfde4013-9062-42f5-a42b-626ef	SBS2	SBS8	6066	4402	0.1866498	0	0
Eso-AdenoCa	dfde4013-9062-42f5-a42b-626ef	SBS6	SBS7	448	851	0.0963992	0.0085519	0.0400257
Liver-HCC	3faeb03e-c622-11e3-bf01-24c6	SBS4	SBS14	6690	637	0.0955697	4.86E-05	0.0004416
Liver-HCC	3faeb03e-c622-11e3-bf01-24c6	SBS4	SBS16	6690	13040	0.087588	0	0
Skin-Melanoma	c95a2b1b-726c-4608-9fff-d57b6	SBS7	SBS11	108992	2508	0.0671898	4.87E-10	8.60E-09
Kidney-RCC	155873b2-e5de-405f-87f6-6de0	SBS3	SBS16	1791	811	0.0793493	0.0017707	0.0107321
Lymph-BNHL	3e012b50-06d1-4120-971b-5e5	SBS1	SBS5	2347	19928	0.0437112	0.0006552	0.0045373
Lymph-BNHL	3e012b50-06d1-4120-971b-5e5	SBS1	SBS9	2347	2539	0.0677482	2.75E-05	0.0002632
Head-SCC	9650640f-154d-4696-aa96-3611	SBS1	SBS3	1142	2016	0.0856432	4.53E-05	0.000413
Head-SCC	9650640f-154d-4696-aa96-3611	SBS1	SBS9	1142	1572	0.106975	5.32E-07	6.78E-06
Head-SCC	9650640f-154d-4696-aa96-3611	SBS5	SBS9	1162	1572	0.0622928	0.0111976	0.0497345
Panc-AdenoCA	53e6a756-bdcc-4217-afe7-27d7	SBS1	SBS4	2664	462	0.0842596	0.0074658	0.0358806
Liver-HCC	5dce221a-c623-11e3-bf01-24c6	SBS4	SBS16	1537	1975	0.0618835	0.0026665	0.0152058
Prost-AdenoCA	1a319682-f55e-4e0b-8476-48a0	SBS1	SBS3	1073	887	0.2219362	0	0
Prost-AdenoCA	1a319682-f55e-4e0b-8476-48a0	SBS1	SBS16	1073	648	0.1878747	8.22E-13	1.82E-11
Liver-HCC	59632e7e-c622-11e3-bf01-24c6	SBS3	SBS12	1953	12054	0.03977	0.0098195	0.0446256
Lymph-CLL	132f7f2a-b902-4343-aa08-cf6a7	SBS1	SBS9	754	390	0.2267905	6.57E-12	1.32E-10
Lymph-CLL	132f7f2a-b902-4343-aa08-cf6a7	SBS1	SBS12	754	765	0.0847055	0.0085999	0.0401608
Lymph-CLL	132f7f2a-b902-4343-aa08-cf6a7	SBS8	SBS9	1148	390	0.2261056	2.37E-13	5.50E-12
Lymph-CLL	132f7f2a-b902-4343-aa08-cf6a7	SBS8	SBS12	1148	765	0.0856494	0.0023761	0.0138262
Lymph-CLL	132f7f2a-b902-4343-aa08-cf6a7	SBS9	SBS12	390	765	0.1575163	5.42E-06	5.84E-05

Eso-AdenoCa	569393c8-e2fe-4580-a45b-81f1b	SBS8	SBS9	2817	5296	0.0386058	0.0083274	0.0392457
Eso-AdenoCa	569393c8-e2fe-4580-a45b-81f1b	SBS8	SBS17	2817	32510	0.0844029	2.22E-16	6.72E-15
Eso-AdenoCa	569393c8-e2fe-4580-a45b-81f1b	SBS9	SBS17	5296	32510	0.0526753	2.12E-11	4.08E-10
Lymph-BNHL	068f4f69-d2fe-4f25-912e-ca7d4	SBS1	SBS3	1596	4735	0.0468027	0.0107134	0.0479918
Prost-AdenoCA	b3f1d232-4392-4258-9256-d38ff	SBS1	SBS3	565	3338	0.1994051	0	0
Prost-AdenoCA	b3f1d232-4392-4258-9256-d38ff	SBS3	SBS6	3338	119	0.1684952	0.0029345	0.0164963
Skin-Melanoma	5c3def3a-b515-41f6-8157-681b	SBS7	SBS11	493078	20683	0.017207	1.57E-05	0.000156
Skin-Melanoma	9fc5b5c7-3973-42b4-8710-454d	SBS1	SBS6	6550	11923	0.1194864	0	0
Skin-Melanoma	9fc5b5c7-3973-42b4-8710-454d	SBS1	SBS7	6550	167076	0.2394576	0	0
Skin-Melanoma	9fc5b5c7-3973-42b4-8710-454d	SBS1	SBS11	6550	8078	0.064897	1.17E-13	2.81E-12
Skin-Melanoma	9fc5b5c7-3973-42b4-8710-454d	SBS6	SBS7	11923	167076	0.3566712	0	0
Skin-Melanoma	9fc5b5c7-3973-42b4-8710-454d	SBS6	SBS11	11923	8078	0.1811812	0	0
Skin-Melanoma	9fc5b5c7-3973-42b4-8710-454d	SBS7	SBS11	167076	8078	0.1771562	0	0
Kidney-RCC	cbb788dd-964b-4cfd-80e1-979c	SBS1	SBS3	460	6173	0.1091292	7.46E-05	0.0006466
Panc-AdenoCA	5d9ff58c-5702-48fc-a66a-d1276	SBS3	SBS4	7500	3961	0.0900454	0	0
Panc-AdenoCA	5d9ff58c-5702-48fc-a66a-d1276	SBS3	SBS5	7500	1610	0.0749822	6.73E-07	8.32E-06
Panc-AdenoCA	5d9ff58c-5702-48fc-a66a-d1276	SBS3	SBS15	7500	1927	0.1490094	0	0
Panc-AdenoCA	5d9ff58c-5702-48fc-a66a-d1276	SBS4	SBS15	3961	1927	0.0743105	1.21E-06	1.46E-05
Panc-AdenoCA	5d9ff58c-5702-48fc-a66a-d1276	SBS5	SBS15	1610	1927	0.084981	6.29E-06	6.69E-05
Breast-AdenoCa	fc5dc6d8-62d2-76d8-e040-11ac	SBS1	SBS3	4898	1416	0.0517832	0.0055287	0.0279647
Breast-AdenoCa	f7b84bac-f161-9eee-e040-11ac0	SBS2	SBS3	1105	913	0.125553	2.86E-07	3.79E-06
Breast-AdenoCa	f7b84bac-f161-9eee-e040-11ac0	SBS2	SBS12	1105	605	0.1023298	0.0005562	0.0039385
Breast-AdenoCa	f7b84bac-f161-9eee-e040-11ac0	SBS2	SBS15	1105	373	0.1578664	1.84E-06	2.16E-05
Breast-AdenoCa	f7b84bac-f161-9eee-e040-11ac0	SBS3	SBS13	913	1013	0.1830043	2.15E-14	5.44E-13
Breast-AdenoCa	f7b84bac-f161-9eee-e040-11ac0	SBS12	SBS13	605	1013	0.1589224	9.81E-09	1.51E-07
Breast-AdenoCa	f7b84bac-f161-9eee-e040-11ac0	SBS13	SBS15	1013	373	0.2169332	1.44E-11	2.81E-10
Eso-AdenoCa	9258860c-a336-4075-996d-2ee0	SBS6	SBS17	2266	18808	0.059446	1.24E-06	1.49E-05
Prost-AdenoCA	0bfebf9f-c783-e57d-e050-11ac0	SBS1	SBS9	771	1741	0.1247446	1.20E-07	1.65E-06
Prost-AdenoCA	0bfebf9f-c783-e57d-e050-11ac0	SBS4	SBS9	961	1741	0.0769553	0.0013058	0.0082748
Prost-AdenoCA	0bfebf9f-c783-e57d-e050-11ac0	SBS9	SBS15	1741	438	0.0889559	0.0078646	0.0374809
Kidney-RCC	005794f1-5a87-45b5-9811-83dd	SBS4	SBS16	1473	92	0.1776174	0.0084767	0.0397929
Head-SCC	0f9e70b3-24cf-4d76-af7b-84ace	SBS3	SBS4	2685	610	0.0865555	0.0011652	0.0075129
Skin-Melanoma	1ac15380-04a2-42dd-8ade-2855	SBS2	SBS3	722	3238	0.0880404	0.000212	0.0016507
Skin-Melanoma	1ac15380-04a2-42dd-8ade-2855	SBS3	SBS11	3238	331	0.0931415	0.0109185	0.0486673
Skin-Melanoma	1ac15380-04a2-42dd-8ade-2855	SBS3	SBS13	3238	659	0.1200853	2.77E-07	3.70E-06
Skin-Melanoma	1ac15380-04a2-42dd-8ade-2855	SBS4	SBS13	861	659	0.1093199	0.0002668	0.0020414
Prost-AdenoCA	5d6ad982-bb01-4233-b8fa-d129	SBS1	SBS8	1604	1480	0.072021	0.0006808	0.0046677
Skin-Melanoma	e2b2357c-da6f-4ca3-9c07-a22f0	SBS7	SBS11	143113	6236	0.0230654	0.0034649	0.0189813
Prost-AdenoCA	120f01d1-8884-4aca-a1cb-36b2	SBS2	SBS5	475	415	0.1090679	0.0102916	0.0465351
Prost-AdenoCA	120f01d1-8884-4aca-a1cb-36b2	SBS2	SBS16	475	1436	0.102957	0.0010344	0.0067772
Prost-AdenoCA	f5378545-17d4-4a64-a57e-f6c91	SBS1	SBS5	2437	4263	0.2351864	0	0
Prost-AdenoCA	f5378545-17d4-4a64-a57e-f6c91	SBS1	SBS9	2437	739	0.205087	0	0
Prost-AdenoCA	f5378545-17d4-4a64-a57e-f6c91	SBS5	SBS6	4263	102	0.184529	0.0022633	0.0132315
Prost-AdenoCA	f5378545-17d4-4a64-a57e-f6c91	SBS6	SBS9	102	739	0.1926424	0.0025817	0.0147892
Panc-AdenoCA	54195db3-94a9-4538-8bb8-9953	SBS3	SBS6	5364	871	0.0725261	0.0007542	0.0050935
Panc-AdenoCA	54195db3-94a9-4538-8bb8-9953	SBS3	SBS13	5364	714	0.0837109	0.0002922	0.0022226
Panc-AdenoCA	54195db3-94a9-4538-8bb8-9953	SBS6	SBS8	871	3871	0.0607224	0.0105653	0.0474983
Panc-AdenoCA	54195db3-94a9-4538-8bb8-9953	SBS8	SBS13	3871	714	0.068207	0.0073309	0.0353406
Breast-AdenoCa	f393bb07-270c-2c93-e040-11ac	SBS3	SBS13	7931	1138	0.1833886	0	0
Ovary-AdenoCA	4e913899-eeb8-4630-ba9d-41a	SBS1	SBS13	860	1587	0.2496073	0	0
Ovary-AdenoCA	4e913899-eeb8-4630-ba9d-41a	SBS3	SBS13	2918	1587	0.2125464	0	0
Ovary-AdenoCA	cd9efdef-a7fb-49e5-9515-63606	SBS1	SBS5	565	1271	0.1163128	5.07E-05	0.0004577
Ovary-AdenoCA	cd9efdef-a7fb-49e5-9515-63606	SBS1	SBS8	565	2220	0.1070717	6.55E-05	0.0005758
Eso-AdenoCa	532bec86-b8d3-41a1-ba17-913a	SBS6	SBS8	5562	7897	0.0686131	9.05E-14	2.19E-12
Eso-AdenoCa	532bec86-b8d3-41a1-ba17-913a	SBS6	SBS17	5562	15340	0.0259614	0.0081536	0.0385939
Eso-AdenoCa	532bec86-b8d3-41a1-ba17-913a	SBS8	SBS17	7897	15340	0.0692725	0	0
Panc-AdenoCA	40378b7a-a65d-4510-aaba-a34	SBS1	SBS2	5450	909	0.0631059	0.0040386	0.0214715
Eso-AdenoCa	1f967003-82d6-4932-a445-24fc	SBS1	SBS17	7536	2952	0.0691844	3.04E-09	4.96E-08
Panc-AdenoCA	b47aa163-eec9-4225-940b-4373	SBS1	SBS8	2581	1626	0.0919161	9.56E-08	1.33E-06
Eso-AdenoCa	f37de4fa-52f6-4fe4-a3fa-418030	SBS1	SBS9	3658	2619	0.0469796	0.002372	0.0138156
Eso-AdenoCa	f37de4fa-52f6-4fe4-a3fa-418030	SBS1	SBS17	3658	20237	0.0572155	3.10E-09	5.04E-08
Eso-AdenoCa	f37de4fa-52f6-4fe4-a3fa-418030	SBS8	SBS17	8442	20237	0.0686057	0	0
Eso-AdenoCa	f37de4fa-52f6-4fe4-a3fa-418030	SBS9	SBS17	2619	20237	0.067196	1.61E-09	2.72E-08
Eso-AdenoCa	f37de4fa-52f6-4fe4-a3fa-418030	SBS15	SBS17	454	20237	0.1104836	3.92E-05	0.0003643

Lymph-CLL	99e7016a-c3c1-431c-8838-beb7	SBS1	SBS5	434	997	0.1219488	0.0002484	0.001915
Lymph-CLL	99e7016a-c3c1-431c-8838-beb7	SBS1	SBS8	434	1570	0.1098565	0.0005456	0.0038817
Eso-AdenoCa	f2e639cc-c30c-459f-8afc-7ace7c	SBS1	SBS7	4700	1115	0.0688427	0.0003901	0.0029061
Eso-AdenoCa	f2e639cc-c30c-459f-8afc-7ace7c	SBS1	SBS17	4700	1938	0.0846402	5.79E-09	9.09E-08
Eso-AdenoCa	f2e639cc-c30c-459f-8afc-7ace7c	SBS5	SBS17	767	1938	0.1182391	4.25E-07	5.49E-06
Eso-AdenoCa	f2e639cc-c30c-459f-8afc-7ace7c	SBS7	SBS17	1115	1938	0.1503061	2.59E-14	6.48E-13
Eso-AdenoCa	f2e639cc-c30c-459f-8afc-7ace7c	SBS8	SBS17	1019	1938	0.1109746	1.44E-07	1.96E-06
Eso-AdenoCa	f2e639cc-c30c-459f-8afc-7ace7c	SBS15	SBS17	358	1938	0.1332336	4.38E-05	0.0004005
Eso-AdenoCa	4283773c-c04b-4f33-a398-f3566	SBS9	SBS17	2666	34917	0.0667365	5.24E-10	9.15E-09
Eso-AdenoCa	4283773c-c04b-4f33-a398-f3566	SBS15	SBS17	2276	34917	0.0486473	8.11E-05	0.0006979
Prost-AdenoCA	ab8a55ed-ff47-4cad-ad91-52b9c	SBS1	SBS3	2196	1446	0.1068332	4.55E-09	7.29E-08
Prost-AdenoCA	ab8a55ed-ff47-4cad-ad91-52b9c	SBS1	SBS9	2196	316	0.1155784	0.0012466	0.0079721
Panc-AdenoCA	2b6d4d66-7f0b-4bc0-b3d6-1719	SBS1	SBS4	2048	649	0.0920775	0.0004697	0.0033989
Panc-AdenoCA	2b6d4d66-7f0b-4bc0-b3d6-1719	SBS1	SBS7	2048	260	0.1714017	2.59E-06	2.99E-05
Panc-AdenoCA	2b6d4d66-7f0b-4bc0-b3d6-1719	SBS1	SBS13	2048	376	0.1432326	4.37E-06	4.78E-05
Panc-AdenoCA	2b6d4d66-7f0b-4bc0-b3d6-1719	SBS1	SBS17	2048	863	0.0949048	3.56E-05	0.0003322
Kidney-RCC	e053d377-e4f2-4aee-af7d-e616(	SBS1	SBS5	431	5094	0.1018427	0.0005261	0.0037681
Kidney-RCC	3afb3f57-545a-4ed5-9461-2fcb2	SBS5	SBS8	50	4824	0.2323549	0.0095583	0.0438198
Liver-HCC	ed52e836-c622-11e3-bf01-24c6	SBS15	SBS16	130	4008	0.1492208	0.0073405	0.0353595
Breast-AdenoCa	fc8130df-90ba-5d94-e040-11ac(	SBS1	SBS3	373	3912	0.137714	4.91E-06	5.33E-05
Breast-AdenoCa	fc8130df-90ba-5d94-e040-11ac(	SBS1	SBS7	373	985	0.1158776	0.0013983	0.0087792
Breast-AdenoCa	fc8130df-90ba-5d94-e040-11ac(	SBS1	SBS8	373	1106	0.1446824	1.70E-05	0.0001669
Breast-AdenoCa	fc8130df-90ba-5d94-e040-11ac(	SBS1	SBS13	373	1403	0.147797	5.13E-06	5.55E-05
Breast-AdenoCa	fc8130df-90ba-5d94-e040-11ac(	SBS1	SBS16	373	1525	0.1764884	1.56E-08	2.37E-07
Breast-AdenoCa	fc8130df-90ba-5d94-e040-11ac(	SBS7	SBS16	985	1525	0.072251	0.0038681	0.020792
Liver-HCC	8cf713ff-256b-4411-a1b4-5bf6f4	SBS5	SBS12	5349	1947	0.0592314	8.94E-05	0.0007631
Panc-Endocrine	d404ac28-da11-4746-9c3b-cf0b	SBS1	SBS2	720	1066	0.0875912	0.0027367	0.0155351
Prost-AdenoCA	5c8da06c-7964-4ad1-ac9e-d1b5	SBS1	SBS12	2514	2711	0.1607177	0	0
Breast-AdenoCa	fc8130e0-a538-dda3-e040-11ac	SBS1	SBS2	1813	1696	0.1601556	0	0
Breast-AdenoCa	fc8130e0-a538-dda3-e040-11ac	SBS2	SBS3	1696	1204	0.1573528	1.44E-15	4.13E-14
Breast-AdenoCa	fc8130e0-a538-dda3-e040-11ac	SBS2	SBS16	1696	412	0.1834929	4.05E-10	7.24E-09
Panc-AdenoCA	1168944c-5451-4a18-8758-88bf	SBS1	SBS3	563	9283	0.094041	0.0001673	0.001336
Panc-AdenoCA	1168944c-5451-4a18-8758-88bf	SBS3	SBS6	9283	470	0.0820589	0.0048369	0.0250521
Prost-AdenoCA	0bfe2ac9-0afa-c248-e050-11ac(	SBS4	SBS9	1586	541	0.0801258	0.011259	0.0499718
Panc-AdenoCA	c741fb12-8160-43a7-bc70-2c54	SBS1	SBS5	3783	2849	0.1255489	0	0
Panc-AdenoCA	c741fb12-8160-43a7-bc70-2c54	SBS1	SBS15	3783	359	0.0926488	0.0071845	0.0347415
Panc-AdenoCA	c741fb12-8160-43a7-bc70-2c54	SBS4	SBS5	316	2849	0.0960819	0.0104748	0.0471591
Liver-HCC	1295947c-c623-11e3-bf01-24c6	SBS4	SBS16	962	30532	0.0527821	0.0110725	0.0492487
Panc-AdenoCA	f7702c0c-9636-4700-a8ae-7a58	SBS1	SBS2	5398	641	0.1135328	7.70E-07	9.44E-06
Liver-HCC	cdcc04fc-c622-11e3-bf01-24c65	SBS12	SBS16	6944	8274	0.042368	2.60E-06	2.99E-05
Eso-AdenoCa	f0a33adc-ea90-46ef-a24a-a8d7e	SBS6	SBS8	4132	4864	0.0345084	0.0097767	0.0445283
Eso-AdenoCa	f0a33adc-ea90-46ef-a24a-a8d7e	SBS6	SBS17	4132	26719	0.027797	0.0079307	0.0377099
Eso-AdenoCa	f0a33adc-ea90-46ef-a24a-a8d7e	SBS8	SBS17	4864	26719	0.0593528	5.13E-13	1.15E-11
Head-SCC	f35f7712-d5c6-47f6-98ed-704ed	SBS1	SBS2	1519	7313	0.0938969	4.67E-10	8.33E-09
Head-SCC	f35f7712-d5c6-47f6-98ed-704ed	SBS1	SBS13	1519	9150	0.0594047	0.0002032	0.0015898
Head-SCC	f35f7712-d5c6-47f6-98ed-704ed	SBS2	SBS5	7313	3753	0.1068276	0	0
Head-SCC	f35f7712-d5c6-47f6-98ed-704ed	SBS2	SBS7	7313	1991	0.0694815	5.48E-07	6.95E-06
Head-SCC	f35f7712-d5c6-47f6-98ed-704ed	SBS2	SBS13	7313	9150	0.0389702	8.70E-06	9.07E-05
Head-SCC	f35f7712-d5c6-47f6-98ed-704ed	SBS5	SBS13	3753	9150	0.0688389	2.22E-11	4.24E-10
Liver-HCC	4ed52e0c-c623-11e3-bf01-24c6	SBS8	SBS12	581	2352	0.1091304	3.03E-05	0.0002869
Liver-HCC	4ed52e0c-c623-11e3-bf01-24c6	SBS12	SBS16	2352	4217	0.0546164	0.0002449	0.0018902
Ovary-AdenoCA	f858d813-f3c5-4ad9-8c20-9f231	SBS1	SBS13	950	2104	0.0813908	0.0003429	0.0025797
Skin-Melanoma	12f1ae2f-2666-45be-9742-f502d	SBS7	SBS11	86779	4391	0.0574159	2.16E-12	4.61E-11
Ovary-AdenoCA	efbec43c-0c16-4006-abe8-c3ec	SBS3	SBS6	4991	558	0.0827378	0.0020741	0.0122971
Breast-AdenoCa	f393bb0b-08ed-3335-e040-11ac	SBS1	SBS3	914	6599	0.1447832	4.88E-15	1.33E-13
Breast-AdenoCa	f393bb0b-08ed-3335-e040-11ac	SBS1	SBS10	914	1300	0.1212052	2.84E-07	3.77E-06
Breast-AdenoCa	f393bb0b-08ed-3335-e040-11ac	SBS1	SBS13	914	1661	0.1572825	4.29E-13	9.75E-12
Breast-AdenoCa	f393bb0b-08ed-3335-e040-11ac	SBS1	SBS14	914	668	0.1277729	6.73E-06	7.10E-05
Ovary-AdenoCA	504cdfc5-6721-43ef-b31e-fdf52f	SBS1	SBS2	1198	1521	0.2697187	0	0
Ovary-AdenoCA	504cdfc5-6721-43ef-b31e-fdf52f	SBS1	SBS3	1198	6342	0.1510396	0	0
Ovary-AdenoCA	504cdfc5-6721-43ef-b31e-fdf52f	SBS1	SBS13	1198	1733	0.2964842	0	0
Ovary-AdenoCA	504cdfc5-6721-43ef-b31e-fdf52f	SBS2	SBS3	1521	6342	0.1396718	0	0
Ovary-AdenoCA	504cdfc5-6721-43ef-b31e-fdf52f	SBS2	SBS4	1521	81	0.2022062	0.0037138	0.0201337
Ovary-AdenoCA	504cdfc5-6721-43ef-b31e-fdf52f	SBS3	SBS13	6342	1733	0.1601041	0	0

Ovary-AdenoCA	504cdfc5-6721-43ef-b31e-fdf52f	SBS4	SBS13	81	1733	0.2334495	0.0004345	0.0031845
Liver-HCC	d60f880a-c622-11e3-bf01-24c65	SBS4	SBS12	5636	5883	0.046063	9.91E-06	0.0001017
Liver-HCC	d60f880a-c622-11e3-bf01-24c65	SBS12	SBS16	5883	4263	0.0329129	0.0094465	0.0434027
Lymph-BNHL	df814571-57f1-4e55-bc1a-c892c	SBS1	SBS8	497	1664	0.0905529	0.0037614	0.0203215
Lymph-BNHL	df814571-57f1-4e55-bc1a-c892c	SBS8	SBS15	1664	116	0.1645806	0.0056198	0.0283115
Prost-AdenoCA	c08f65a0-bf4c-462e-9d07-ad56b	SBS1	SBS3	3617	1281	0.0649302	0.000687	0.0047008
Eso-AdenoCa	9ae33a63-6b1a-43bf-9205-463c	SBS1	SBS17	10541	14331	0.051668	1.65E-14	4.27E-13
Prost-AdenoCA	008aef39-0c97-48ce-9dfd-f12d6	SBS1	SBS16	903	2009	0.0898553	8.55E-05	0.0007322
Panc-AdenoCA	5187e77d-f412-4303-8049-11d1	SBS1	SBS3	1232	619	0.1682529	1.48E-10	2.72E-09
Panc-AdenoCA	5187e77d-f412-4303-8049-11d1	SBS1	SBS8	1232	10025	0.1419474	0	0
Panc-AdenoCA	5187e77d-f412-4303-8049-11d1	SBS1	SBS13	1232	1287	0.1288781	1.66E-09	2.80E-08
Panc-AdenoCA	5187e77d-f412-4303-8049-11d1	SBS8	SBS13	10025	1287	0.054849	0.0020923	0.012382
Prost-AdenoCA	0bfd1068-3fe1-a95b-e050-11ac0	SBS1	SBS3	889	7859	0.0955773	9.21E-07	1.12E-05
Prost-AdenoCA	0bfd1068-3fe1-a95b-e050-11ac0	SBS1	SBS4	889	3506	0.1053829	2.89E-07	3.81E-06
Prost-AdenoCA	0bfd1068-3fe1-a95b-e050-11ac0	SBS1	SBS11	889	827	0.1057504	0.0001378	0.0011306
Eso-AdenoCa	0250e6f7-2538-4d1f-a8ff-ca34a7	SBS1	SBS9	10086	3146	0.0565587	4.35E-07	5.60E-06
Eso-AdenoCa	0250e6f7-2538-4d1f-a8ff-ca34a7	SBS6	SBS9	2237	3146	0.0571543	0.0003904	0.0029061
Eso-AdenoCa	0250e6f7-2538-4d1f-a8ff-ca34a7	SBS9	SBS17	3146	7880	0.0620023	6.22E-08	8.81E-07
Panc-AdenoCA	0cf9bbc2-cbd5-4b64-8d90-cfa41	SBS1	SBS15	3688	385	0.093534	0.0044872	0.0234738
Panc-AdenoCA	0cf9bbc2-cbd5-4b64-8d90-cfa41	SBS1	SBS17	3688	1077	0.2070592	0	0
Panc-AdenoCA	0cf9bbc2-cbd5-4b64-8d90-cfa41	SBS3	SBS17	1198	1077	0.2081967	0	0
Panc-AdenoCA	0cf9bbc2-cbd5-4b64-8d90-cfa41	SBS8	SBS15	3144	385	0.1165948	0.0001782	0.0014084
Panc-AdenoCA	0cf9bbc2-cbd5-4b64-8d90-cfa41	SBS8	SBS17	3144	1077	0.1833434	0	0
Panc-AdenoCA	0cf9bbc2-cbd5-4b64-8d90-cfa41	SBS15	SBS17	385	1077	0.2878342	0	0
Stomach-AdenoCA	d91e1a92-837d-44d6-92d8-419f	SBS7	SBS17	494	1155	0.1100216	0.0004603	0.0033478
Stomach-AdenoCA	d91e1a92-837d-44d6-92d8-419f	SBS8	SBS17	2182	1155	0.0845751	4.06E-05	0.000375
Skin-Melanoma	22d67778-61fc-4f15-95b8-7e7c6	SBS7	SBS11	83541	7603	0.0196693	0.009104	0.0421067
Lymph-BNHL	fdf7dfb7-9285-46b3-be60-67216	SBS1	SBS3	1115	1865	0.0618041	0.0096793	0.0442284
Lymph-BNHL	fdf7dfb7-9285-46b3-be60-67216	SBS3	SBS10	1865	1453	0.0797241	6.20E-05	0.0005494
Lymph-BNHL	fdf7dfb7-9285-46b3-be60-67216	SBS8	SBS9	7423	18998	0.0251635	0.0023197	0.0135361
Lymph-BNHL	fdf7dfb7-9285-46b3-be60-67216	SBS9	SBS10	18998	1453	0.0639261	3.24E-05	0.0003059
Head-SCC	9fb6b7be-0084-48f7-a256-6d170	SBS1	SBS2	1596	387	0.1095617	0.0011311	0.0073154
Head-SCC	9fb6b7be-0084-48f7-a256-6d170	SBS1	SBS13	1596	553	0.1860902	8.87E-13	1.96E-11
Head-SCC	9fb6b7be-0084-48f7-a256-6d170	SBS2	SBS14	387	641	0.11278	0.0043158	0.0226907
Head-SCC	9fb6b7be-0084-48f7-a256-6d170	SBS13	SBS14	553	641	0.1914419	7.08E-10	1.23E-08
Ovary-AdenoCA	d4bf6034-aeae-48a6-907b-10e2	SBS1	SBS3	811	14623	0.0920675	4.40E-06	4.81E-05
Liver-HCC	a1a75f66-c622-11e3-bf01-24c65	SBS3	SBS12	2921	2505	0.0720184	1.68E-06	1.99E-05
Liver-HCC	a1a75f66-c622-11e3-bf01-24c65	SBS11	SBS12	2283	2505	0.0497654	0.0053907	0.0274442
Liver-HCC	a1a75f66-c622-11e3-bf01-24c65	SBS12	SBS16	2505	2111	0.07315	9.48E-06	9.80E-05
Eso-AdenoCa	cb381d49-4546-400c-af02-f8770	SBS1	SBS17	14350	7940	0.1839501	0	0
Liver-HCC	3c78970e-c622-11e3-bf01-24c65	SBS1	SBS16	583	9462	0.0888363	0.000344	0.0025853
Breast-AdenoCa	097a7d36-905b-72be-e050-11ac	SBS2	SBS13	35574	29820	0.0157932	0.0006118	0.0042886
Eso-AdenoCa	ba096d4f-5a6c-4c31-ae03-e748	SBS1	SBS13	4589	1159	0.0635165	0.0011444	0.007394
Panc-AdenoCA	ac02c38e-5fca-4995-b0cc-39b61	SBS1	SBS5	8865	270	0.1038917	0.0069915	0.0339655
Panc-AdenoCA	ac02c38e-5fca-4995-b0cc-39b61	SBS1	SBS14	8865	493	0.1134463	1.20E-05	0.0001218
Panc-AdenoCA	ac02c38e-5fca-4995-b0cc-39b61	SBS5	SBS14	270	493	0.2146946	2.07E-07	2.80E-06
Prost-AdenoCA	b33978c6-a855-4f9d-a0b0-d794	SBS1	SBS5	915	10397	0.2263216	0	0
Prost-AdenoCA	b33978c6-a855-4f9d-a0b0-d794	SBS1	SBS9	915	784	0.2614698	0	0
Prost-AdenoCA	b33978c6-a855-4f9d-a0b0-d794	SBS5	SBS9	10397	784	0.0599414	0.0106139	0.0476826
Breast-AdenoCa	fc8130df-3225-3f96-e040-11ac0	SBS2	SBS5	5387	1455	0.0967771	9.59E-10	1.65E-08
Breast-AdenoCa	fc8130df-3225-3f96-e040-11ac0	SBS5	SBS13	1455	5929	0.115102	7.19E-14	1.76E-12
Ovary-AdenoCA	f988e698-9b34-45ce-ba4c-74e0	SBS9	SBS14	264	547	0.1458091	0.0010301	0.0067596
Liver-HCC	c6bb32f0-c622-11e3-bf01-24c65	SBS1	SBS6	23702	8983	0.0736844	0	0
Liver-HCC	c6bb32f0-c622-11e3-bf01-24c65	SBS1	SBS12	23702	8392	0.0835131	0	0
Liver-HCC	c6bb32f0-c622-11e3-bf01-24c65	SBS1	SBS15	23702	3567	0.0410455	5.81E-05	0.0005186
Liver-HCC	c6bb32f0-c622-11e3-bf01-24c65	SBS6	SBS12	8983	8392	0.0251328	0.0083288	0.0392457
Liver-HCC	c6bb32f0-c622-11e3-bf01-24c65	SBS6	SBS15	8983	3567	0.0416448	0.0002851	0.0021734
Liver-HCC	c6bb32f0-c622-11e3-bf01-24c65	SBS12	SBS15	8392	3567	0.0606634	2.00E-08	2.98E-07
Skin-Melanoma	897ba508-d74b-46bf-8554-1210	SBS7	SBS11	62647	1651	0.1113042	0	0
Eso-AdenoCa	abe290ed-67c8-42bb-84f7-0d32	SBS1	SBS3	3052	2815	0.1189882	0	0
Eso-AdenoCa	abe290ed-67c8-42bb-84f7-0d32	SBS1	SBS5	3052	1810	0.0903901	1.73E-08	2.61E-07
Eso-AdenoCa	abe290ed-67c8-42bb-84f7-0d32	SBS1	SBS6	3052	2007	0.1037496	9.57E-12	1.90E-10
Eso-AdenoCa	abe290ed-67c8-42bb-84f7-0d32	SBS3	SBS15	2815	1432	0.1477765	0	0
Eso-AdenoCa	abe290ed-67c8-42bb-84f7-0d32	SBS5	SBS15	1810	1432	0.1138577	1.99E-09	3.29E-08

Eso-AdenoCa	abe290ed-67c8-42bb-84f7-0d32	SBS6	SBS15	2007	1432	0.1262303	5.43E-12	1.11E-10
Prost-AdenoCA	9ed8f0b9-d615-44c4-9ec9-e07e	SBS1	SBS5	432	2299	0.0958952	0.0024905	0.0143454
Breast-AdenoCa	f393baf9-2710-9203-e040-11ac0	SBS1	SBS3	313	4889	0.0952931	0.0095675	0.0438299
Prost-AdenoCA	f601cf2f-081f-484d-ab0e-21a8e	SBS1	SBS6	7435	1960	0.0569617	8.50E-05	0.0007291
Breast-AdenoCa	fc8130df-8ec8-5b1e-e040-11ac0	SBS2	SBS3	5368	8224	0.2746791	0	0
Breast-AdenoCa	fc8130df-8ec8-5b1e-e040-11ac0	SBS2	SBS7	5368	1900	0.1414044	0	0
Breast-AdenoCa	fc8130df-8ec8-5b1e-e040-11ac0	SBS2	SBS13	5368	7627	0.0385013	0.0001756	0.0013929
Breast-AdenoCa	fc8130df-8ec8-5b1e-e040-11ac0	SBS3	SBS7	8224	1900	0.1433494	0	0
Breast-AdenoCa	fc8130df-8ec8-5b1e-e040-11ac0	SBS3	SBS13	8224	7627	0.2426548	0	0
Breast-AdenoCa	fc8130df-8ec8-5b1e-e040-11ac0	SBS7	SBS13	1900	7627	0.1101656	2.22E-16	6.72E-15
Eso-AdenoCa	ded8b673-53df-4038-a375-7cd0	SBS2	SBS3	1139	4774	0.0535708	0.0102033	0.0462024
Liver-HCC	98d27916-c622-11e3-bf01-24c6	SBS4	SBS16	5705	12880	0.1034309	0	0
Panc-AdenoCA	46e166fe-3b20-49ad-98db-42c8	SBS1	SBS5	2459	1197	0.143452	8.10E-15	2.15E-13
Breast-AdenoCa	f221cbb5-eefa-187f-e040-11ac0	SBS1	SBS3	462	9327	0.097977	0.0004272	0.0031353
Eso-AdenoCa	d707940e-0f9d-4da3-9380-90a4	SBS6	SBS17	2033	18127	0.0436928	0.0018616	0.0111749
Eso-AdenoCa	d707940e-0f9d-4da3-9380-90a4	SBS8	SBS17	4010	18127	0.039774	6.15E-05	0.0005462
Eso-AdenoCa	371235d9-55b8-40a7-8cd6-baa7	SBS1	SBS17	3684	20513	0.0666786	1.74E-12	3.74E-11
Eso-AdenoCa	371235d9-55b8-40a7-8cd6-baa7	SBS8	SBS17	4030	20513	0.0729187	5.55E-16	1.65E-14
Eso-AdenoCa	371235d9-55b8-40a7-8cd6-baa7	SBS15	SBS17	566	20513	0.105579	9.29E-06	9.64E-05
Skin-Melanoma	eeddf701-93f9-4f10-85cb-9dce1	SBS7	SBS11	19399	625	0.0829876	0.0004776	0.00344
Kidney-RCC	04db8bef-8777-48ac-bc2e-3c9a	SBS2	SBS3	524	5228	0.0909095	0.0007624	0.0051375
Kidney-RCC	04db8bef-8777-48ac-bc2e-3c9a	SBS2	SBS16	524	844	0.1144767	0.000418	0.0030816
Kidney-RCC	04db8bef-8777-48ac-bc2e-3c9a	SBS15	SBS16	532	844	0.0905106	0.0095301	0.0437229
Liver-HCC	56c846d6-c622-11e3-bf01-24c6	SBS4	SBS16	2501	3951	0.1027385	1.82E-14	4.66E-13
Ovary-AdenoCA	da43386c-47f8-4e03-b6ca-8b94	SBS1	SBS16	781	234	0.1205281	0.0106934	0.0479366
Skin-Melanoma	561fd34c-7c7d-4df0-bbfc-3d311	SBS4	SBS7	5472	68528	0.1549474	0	0
Skin-Melanoma	561fd34c-7c7d-4df0-bbfc-3d311	SBS4	SBS11	5472	4494	0.138336	0	0
Liver-HCC	bba106ce-c622-11e3-bf01-24c6	SBS4	SBS16	437	6870	0.0909609	0.00223	0.0130611
Liver-HCC	bba106ce-c622-11e3-bf01-24c6	SBS14	SBS16	775	6870	0.0669756	0.0038684	0.020792
Prost-AdenoCA	4eac784b-084a-4106-9a76-2d9f	SBS1	SBS12	2353	483	0.091429	0.0024623	0.0142226
Prost-AdenoCA	4eac784b-084a-4106-9a76-2d9f	SBS9	SBS15	141	36	0.3144208	0.0068941	0.0335446
Prost-AdenoCA	4eac784b-084a-4106-9a76-2d9f	SBS12	SBS15	483	36	0.2926156	0.0064469	0.0316638
Skin-Melanoma	1d4a091d-fe65-49c0-8810-5a95	SBS7	SBS11	144306	8381	0.025413	7.21E-05	0.0006273
Bone-Osteosarc	f82d213f-bc99-5b1d-e040-11ac0	SBS3	SBS17	3228	2076	0.0787313	3.15E-07	4.11E-06
Bone-Osteosarc	f82d213f-bc99-5b1d-e040-11ac0	SBS6	SBS17	637	2076	0.1055412	3.85E-05	0.0003587
Bone-Osteosarc	f82d213f-bc99-5b1d-e040-11ac0	SBS13	SBS17	1263	2076	0.0975371	6.49E-07	8.05E-06
Bone-Osteosarc	f82d213f-bc99-5b1d-e040-11ac0	SBS16	SBS17	2083	2076	0.0779024	6.61E-06	6.99E-05
Liver-HCC	3b02a7a7-e5bc-4e77-b691-5216	SBS4	SBS16	1387	5224	0.0499877	0.008361	0.0393679
Liver-HCC	3b02a7a7-e5bc-4e77-b691-5216	SBS8	SBS16	5581	5224	0.0486128	5.79E-06	6.18E-05
Eso-AdenoCa	c971ffa7-ce37-4138-b028-598a	SBS1	SBS8	1094	2034	0.0767887	0.0004546	0.0033164
Eso-AdenoCa	e7a0889c-dd8c-4230-9c63-384f	SBS1	SBS6	5547	4055	0.0366497	0.0036984	0.0200673
Eso-AdenoCa	e7a0889c-dd8c-4230-9c63-384f	SBS1	SBS17	5547	8326	0.1015344	0	0
Eso-AdenoCa	e7a0889c-dd8c-4230-9c63-384f	SBS6	SBS17	4055	8326	0.0863894	0	0
Eso-AdenoCa	e7a0889c-dd8c-4230-9c63-384f	SBS8	SBS17	1728	8326	0.1197564	0	0
Skin-Melanoma	1cd0acf2-3116-4dfa-a063-0a43	SBS1	SBS3	730	2240	0.0752813	0.0038978	0.0209063
Skin-Melanoma	1cd0acf2-3116-4dfa-a063-0a43	SBS1	SBS8	730	474	0.1061673	0.0030717	0.0171419
Liver-HCC	d3ad5b5a-c622-11e3-bf01-24c6	SBS8	SBS12	3424	3919	0.046782	0.0006718	0.0046263
Eso-AdenoCa	9ba6be6d-2032-444b-ab3c-dbe	SBS1	SBS8	2173	3447	0.0548411	0.0006597	0.0045631
Eso-AdenoCa	9ba6be6d-2032-444b-ab3c-dbe	SBS1	SBS9	2173	445	0.1218168	3.47E-05	0.000325
Eso-AdenoCa	9ba6be6d-2032-444b-ab3c-dbe	SBS8	SBS17	3447	5074	0.0458187	0.0003616	0.0027073
Eso-AdenoCa	9ba6be6d-2032-444b-ab3c-dbe	SBS9	SBS17	445	5074	0.1117059	7.36E-05	0.0006397
Lymph-CLL	3e8a2c90-e747-4a22-bc9e-0b06	SBS3	SBS9	213	667	0.1699573	0.000178	0.0014084
Lymph-CLL	3e8a2c90-e747-4a22-bc9e-0b06	SBS9	SBS12	667	997	0.1002994	0.0006442	0.0044803
Panc-AdenoCA	046d7386-95c8-4501-9e55-c85	SBS1	SBS13	1083	271	0.1252636	0.0022223	0.0130283
Panc-AdenoCA	046d7386-95c8-4501-9e55-c85	SBS5	SBS13	1648	271	0.1114579	0.006163	0.0305325
Ovary-AdenoCA	5b560f4c-d2a3-43fa-b394-abef7	SBS1	SBS7	615	221	0.1297576	0.0083834	0.0393843
Ovary-AdenoCA	5b560f4c-d2a3-43fa-b394-abef7	SBS1	SBS8	615	3350	0.129062	6.07E-08	8.63E-07
Ovary-AdenoCA	5b560f4c-d2a3-43fa-b394-abef7	SBS1	SBS12	615	360	0.1597561	1.85E-05	0.0001811
Lymph-BNHL	dbae3c4e-6d66-483a-a611-728	SBS3	SBS6	2403	4943	0.0498983	0.000637	0.0044458
Lymph-BNHL	dbae3c4e-6d66-483a-a611-728	SBS6	SBS9	4943	18525	0.059207	2.63E-12	5.56E-11
Panc-AdenoCA	129459c1-38be-45da-bb87-188	SBS1	SBS5	2073	3423	0.0485242	0.0045762	0.0238791
Lymph-BNHL	f04aecf0-eb12-4ab9-928e-7bcf2	SBS8	SBS9	1129	926	0.0793827	0.0032844	0.0181475
Lymph-BNHL	f04aecf0-eb12-4ab9-928e-7bcf2	SBS9	SBS11	926	301	0.1072164	0.0107868	0.0482517
Kidney-RCC	b30dfb8b-8288-4e5a-afc2-3d5b	SBS1	SBS3	380	64	0.3146382	3.90E-05	0.0003632

Kidney-RCC	b30dfb8b-8288-4e5a-afc2-3d5bc	SBS1	SBS5	380	5472	0.2587719	0	0
Skin-Melanoma	8ca665f8-fe78-48bf-8c0f-c606d9	SBS7	SBS11	162751	10673	0.0323007	1.68E-09	2.81E-08
Skin-Melanoma	f07b9192-c86f-4ee4-a3b5-feb9c	SBS7	SBS11	78460	2616	0.0932481	0	0
Bone-Osteosarc	f86ae246-2492-9785-e040-11ac	SBS3	SBS9	307	281	0.1372483	0.0079536	0.0377901
Bone-Osteosarc	f86ae246-2492-9785-e040-11ac	SBS9	SBS14	281	920	0.1138132	0.0075711	0.0363587
Panc-AdenoCA	a3210fd0-344c-468e-8ff2-2d086	SBS1	SBS2	1576	753	0.5283848	0	0
Panc-AdenoCA	a3210fd0-344c-468e-8ff2-2d086	SBS1	SBS13	1576	597	0.5480852	0	0
Panc-AdenoCA	a3210fd0-344c-468e-8ff2-2d086	SBS2	SBS5	753	259	0.4894553	0	0
Panc-AdenoCA	a3210fd0-344c-468e-8ff2-2d086	SBS2	SBS6	753	229	0.5075245	0	0
Panc-AdenoCA	a3210fd0-344c-468e-8ff2-2d086	SBS2	SBS8	753	1106	0.4943973	0	0
Panc-AdenoCA	a3210fd0-344c-468e-8ff2-2d086	SBS5	SBS13	259	597	0.5031722	0	0
Panc-AdenoCA	a3210fd0-344c-468e-8ff2-2d086	SBS6	SBS13	229	597	0.5247782	0	0
Panc-AdenoCA	a3210fd0-344c-468e-8ff2-2d086	SBS8	SBS13	1106	597	0.5113785	0	0
Kidney-RCC	d4c6061b-5019-4564-806d-4e75	SBS3	SBS16	5483	1418	0.0590502	0.0007741	0.0052052
Kidney-RCC	d4c6061b-5019-4564-806d-4e75	SBS7	SBS16	1276	1418	0.0773176	0.0006511	0.0045187
Eso-AdenoCa	d4cf11be-3bd7-403b-9722-91d8	SBS9	SBS17	5082	14285	0.1020829	0	0
Eso-AdenoCa	d4cf11be-3bd7-403b-9722-91d8	SBS16	SBS17	691	14285	0.0772848	0.0007612	0.0051352
Eso-AdenoCa	d1842491-29ff-4801-8c8a-4d619	SBS1	SBS17	3403	17133	0.1014191	0	0
Eso-AdenoCa	d1842491-29ff-4801-8c8a-4d619	SBS15	SBS17	2561	17133	0.0730084	9.68E-11	1.82E-09
Breast-AdenoCa	fc8130e0-a774-d834-e040-11ac	SBS1	SBS7	1463	584	0.1352716	4.65E-07	5.97E-06
Breast-AdenoCa	fc8130e0-a774-d834-e040-11ac	SBS1	SBS12	1463	542	0.1865297	2.23E-12	4.75E-11
Breast-AdenoCa	fc8130e0-a774-d834-e040-11ac	SBS1	SBS15	1463	470	0.1374878	2.89E-06	3.28E-05
Breast-AdenoCa	fc8130e0-a774-d834-e040-11ac	SBS5	SBS7	499	584	0.1970654	1.68E-09	2.81E-08
Breast-AdenoCa	fc8130e0-a774-d834-e040-11ac	SBS5	SBS12	499	542	0.1223961	0.0008325	0.0055415
Breast-AdenoCa	fc8130e0-a774-d834-e040-11ac	SBS7	SBS12	584	542	0.3137606	0	0
Breast-AdenoCa	fc8130e0-a774-d834-e040-11ac	SBS7	SBS15	584	470	0.2668901	1.11E-16	3.39E-15
Liver-HCC	c5ad7238-c622-11e3-bf01-24c65	SBS2	SBS4	1500	4409	0.0798104	1.28E-06	1.54E-05
Liver-HCC	c5ad7238-c622-11e3-bf01-24c65	SBS2	SBS16	1500	6773	0.0552706	0.001103	0.0071557
Liver-HCC	c5ad7238-c622-11e3-bf01-24c65	SBS4	SBS8	4409	858	0.0641311	0.0054359	0.0276067
Liver-HCC	c5ad7238-c622-11e3-bf01-24c65	SBS4	SBS16	4409	6773	0.0367857	0.0014527	0.0090585
Prost-AdenoCA	0c0038ff-6cc8-b0b0-e050-11ac0	SBS8	SBS9	863	1265	0.0776618	0.0041064	0.0217541
Prost-AdenoCA	a1af7c42-47af-435c-bb51-b932a	SBS1	SBS8	3070	199	0.1586712	0.0001638	0.0013107
Panc-AdenoCA	079bcb09-a075-4313-9a3e-dd67	SBS1	SBS2	5953	970	0.1847477	0	0
Panc-AdenoCA	079bcb09-a075-4313-9a3e-dd67	SBS1	SBS3	5953	1206	0.0639388	0.0005496	0.0039048
Panc-AdenoCA	079bcb09-a075-4313-9a3e-dd67	SBS2	SBS3	970	1206	0.143328	5.11E-10	8.95E-09
Lymph-BNHL	4adc846a-42cb-4ebd-a954-e31e	SBS1	SBS5	5691	5903	0.0343264	0.0021653	0.0127539
Lymph-BNHL	4adc846a-42cb-4ebd-a954-e31e	SBS1	SBS9	5691	6730	0.049362	5.96E-07	7.48E-06
Liver-HCC	cf2d34c4-c622-11e3-bf01-24c65	SBS3	SBS12	600	349	0.1295989	0.0012078	0.0077385
Liver-HCC	cf2d34c4-c622-11e3-bf01-24c65	SBS5	SBS12	13119	349	0.0911524	0.0070405	0.0341506
Liver-HCC	cf2d34c4-c622-11e3-bf01-24c65	SBS12	SBS17	349	834	0.1185917	0.0019744	0.0117958
Eso-AdenoCa	33992441-4f34-42f2-ba87-6ddd2	SBS1	SBS3	755	5603	0.0732082	0.0015986	0.0098222
Eso-AdenoCa	33992441-4f34-42f2-ba87-6ddd2	SBS3	SBS13	5603	675	0.0714485	0.0042643	0.0224763
Biliary-AdenoCA	56cc8740-b132-4cbc-b513-f0b5b	SBS1	SBS2	6238	2980	0.0588155	1.74E-06	2.06E-05
Biliary-AdenoCA	56cc8740-b132-4cbc-b513-f0b5b	SBS2	SBS3	2980	890	0.0735993	0.0011928	0.007667
Biliary-AdenoCA	56cc8740-b132-4cbc-b513-f0b5b	SBS2	SBS4	2980	730	0.0790521	0.001313	0.0083114
Lymph-CLL	c13d7ee5-2bf0-43e4-9cd3-9326	SBS1	SBS3	726	489	0.1522841	2.60E-06	2.99E-05
Lymph-CLL	c13d7ee5-2bf0-43e4-9cd3-9326	SBS1	SBS5	726	277	0.1830564	2.92E-06	3.31E-05
Lymph-CLL	c13d7ee5-2bf0-43e4-9cd3-9326	SBS1	SBS8	726	2156	0.1449935	2.42E-10	4.37E-09
Lymph-CLL	c13d7ee5-2bf0-43e4-9cd3-9326	SBS1	SBS9	726	772	0.4388337	0	0
Lymph-CLL	c13d7ee5-2bf0-43e4-9cd3-9326	SBS3	SBS9	489	772	0.2906323	0	0
Lymph-CLL	c13d7ee5-2bf0-43e4-9cd3-9326	SBS3	SBS11	489	619	0.1405592	4.10E-05	0.000378
Lymph-CLL	c13d7ee5-2bf0-43e4-9cd3-9326	SBS5	SBS9	277	772	0.2632714	1.07E-12	2.33E-11
Lymph-CLL	c13d7ee5-2bf0-43e4-9cd3-9326	SBS5	SBS11	277	619	0.1758513	1.45E-05	0.0001449
Lymph-CLL	c13d7ee5-2bf0-43e4-9cd3-9326	SBS8	SBS9	2156	772	0.3054159	0	0
Lymph-CLL	c13d7ee5-2bf0-43e4-9cd3-9326	SBS8	SBS11	2156	619	0.1223051	1.13E-06	1.37E-05
Lymph-CLL	c13d7ee5-2bf0-43e4-9cd3-9326	SBS9	SBS11	772	619	0.4237112	0	0
Liver-HCC	4c8afa82-c623-11e3-bf01-24c65	SBS3	SBS12	954	2377	0.0637358	0.0079244	0.0377088
Bone-Osteosarc	f866123a-9eb0-e856-e040-11ac	SBS1	SBS2	386	343	0.1281137	0.0051507	0.0263937
Bone-Osteosarc	f866123a-9eb0-e856-e040-11ac	SBS1	SBS3	386	4992	0.1022714	0.0011116	0.0071964
Bone-Osteosarc	f866123a-9eb0-e856-e040-11ac	SBS3	SBS4	4992	361	0.1022067	0.0017636	0.0106995
Liver-HCC	3b41cb48-c623-11e3-bf01-24c65	SBS3	SBS8	1502	2632	0.0650344	0.0006137	0.0042974
Bone-Epith	fc95d5ce-6899-62f1-e040-11ac0	SBS1	SBS5	483	2965	0.0967457	0.0008403	0.0055783
Bone-Epith	fc95d5ce-6899-62f1-e040-11ac0	SBS1	SBS8	483	609	0.1090883	0.0032866	0.0181475
Bone-Epith	fc95d5ce-6899-62f1-e040-11ac0	SBS2	SBS3	1046	92	0.183785	0.0066087	0.0323315

Bone-Epith	fc95d5ce-6899-62f1-e040-11ac0	SBS2	SBS5	1046	2965	0.114223	3.46E-09	5.58E-08
Bone-Epith	fc95d5ce-6899-62f1-e040-11ac0	SBS2	SBS8	1046	609	0.1321886	2.88E-06	3.28E-05
Bone-Epith	fc95d5ce-6899-62f1-e040-11ac0	SBS3	SBS13	92	536	0.2064406	0.0024794	0.0142948
Bone-Epith	fc95d5ce-6899-62f1-e040-11ac0	SBS5	SBS13	2965	536	0.1315723	2.99E-07	3.92E-06
Bone-Epith	fc95d5ce-6899-62f1-e040-11ac0	SBS8	SBS13	609	536	0.1487728	6.61E-06	6.99E-05
Ovary-AdenoCA	bbe59385-5f83-43f6-a485-517c8	SBS4	SBS6	4161	3810	0.091626	6.33E-15	1.69E-13
Ovary-AdenoCA	bbe59385-5f83-43f6-a485-517c8	SBS4	SBS12	4161	2247	0.0662375	5.50E-06	5.91E-05
Eso-AdenoCa	35dc84c1-476c-4d48-ae23-622b	SBS1	SBS13	5628	1963	0.0534182	0.0004941	0.0035554
Eso-AdenoCa	35dc84c1-476c-4d48-ae23-622b	SBS1	SBS17	5628	21764	0.0759598	0	0
Eso-AdenoCa	35dc84c1-476c-4d48-ae23-622b	SBS3	SBS13	966	1963	0.0646552	0.008919	0.0414962
Eso-AdenoCa	35dc84c1-476c-4d48-ae23-622b	SBS3	SBS17	966	21764	0.0678035	0.0004051	0.0030009
Eso-AdenoCa	35dc84c1-476c-4d48-ae23-622b	SBS9	SBS13	4654	1963	0.0718402	1.29E-06	1.55E-05
Eso-AdenoCa	35dc84c1-476c-4d48-ae23-622b	SBS9	SBS17	4654	21764	0.055315	1.29E-10	2.40E-09
Eso-AdenoCa	35dc84c1-476c-4d48-ae23-622b	SBS13	SBS17	1963	21764	0.1240884	0	0
Eso-AdenoCa	0e872e0d-4711-4364-a5d0-6bee	SBS6	SBS17	5043	26108	0.0334	0.0001606	0.0012867
Eso-AdenoCa	0e872e0d-4711-4364-a5d0-6bee	SBS9	SBS17	10307	26108	0.046119	4.45E-14	1.09E-12
Breast-AdenoCa	fc8130df-35ac-2304-e040-11ac0	SBS1	SBS4	701	1051	0.0857997	0.004094	0.0217064
Breast-AdenoCa	fc8130df-35ac-2304-e040-11ac0	SBS1	SBS8	701	2032	0.0884416	0.0005754	0.0040612
Liver-HCC	3f99ae0e-c623-11e3-bf01-24c65	SBS3	SBS4	3820	5787	0.0710984	1.58E-10	2.88E-09
Liver-HCC	3f99ae0e-c623-11e3-bf01-24c65	SBS3	SBS12	3820	8183	0.1188719	0	0
Liver-HCC	3f99ae0e-c623-11e3-bf01-24c65	SBS4	SBS12	5787	8183	0.0571415	4.87E-10	8.60E-09
Liver-HCC	3f99ae0e-c623-11e3-bf01-24c65	SBS12	SBS15	8183	976	0.0890923	1.95E-06	2.27E-05
Liver-HCC	4255582e-c622-11e3-bf01-24c65	SBS3	SBS12	1025	4244	0.0574614	0.0085756	0.0400772
Eso-AdenoCa	47874ff4-0b1c-4a31-9192-2a10e	SBS1	SBS3	11302	3204	0.0534702	1.26E-06	1.52E-05
Eso-AdenoCa	47874ff4-0b1c-4a31-9192-2a10e	SBS1	SBS4	11302	1691	0.0429791	0.0087297	0.0406457
Eso-AdenoCa	47874ff4-0b1c-4a31-9192-2a10e	SBS3	SBS4	3204	1691	0.0911689	2.04E-08	3.05E-07
Eso-AdenoCa	47874ff4-0b1c-4a31-9192-2a10e	SBS3	SBS6	3204	3215	0.059305	2.50E-05	0.0002422
Liver-HCC	47050918-c623-11e3-bf01-24c65	SBS8	SBS12	1814	3293	0.1358108	0	0
Liver-HCC	a4ca18dc-c622-11e3-bf01-24c65	SBS12	SBS16	4742	8129	0.0692085	6.93E-13	1.55E-11
Lymph-BNHL	9b3e7a03-cf8c-4da8-bc36-20129	SBS1	SBS3	779	288	0.1687438	1.26E-05	0.0001272
Lymph-BNHL	9b3e7a03-cf8c-4da8-bc36-20129	SBS1	SBS5	779	11743	0.0708591	0.0013032	0.0082663
Lymph-BNHL	9b3e7a03-cf8c-4da8-bc36-20129	SBS1	SBS17	779	3305	0.0804239	0.0005746	0.0040597
Lymph-BNHL	9b3e7a03-cf8c-4da8-bc36-20129	SBS3	SBS5	288	11743	0.1160975	0.0010233	0.0067224
Lymph-BNHL	9b3e7a03-cf8c-4da8-bc36-20129	SBS3	SBS12	288	176	0.1688763	0.0039352	0.0210276
Lymph-BNHL	9b3e7a03-cf8c-4da8-bc36-20129	SBS3	SBS17	288	3305	0.1219942	0.0007525	0.0050934
Skin-Melanoma	bcefbf6a-80ff-4825-9d3a-492e2c	SBS7	SBS11	114492	6999	0.0510305	2.44E-15	6.93E-14
Prost-AdenoCA	51adf6cf-f422-4d7e-aa46-cc9bf0	SBS1	SBS5	2025	1624	0.0552122	0.0082176	0.0388384
Panc-AdenoCA	4aa7eabc-0419-4897-b7af-5dccc	SBS2	SBS3	1056	8588	0.0792424	1.49E-05	0.0001481
Panc-AdenoCA	4aa7eabc-0419-4897-b7af-5dccc	SBS2	SBS4	1056	1119	0.0868445	0.0005521	0.0039164
Panc-AdenoCA	4aa7eabc-0419-4897-b7af-5dccc	SBS3	SBS6	8588	1593	0.0565141	0.0003744	0.0027964
Eso-AdenoCa	dd7d623b-b9af-4147-9aa6-e097	SBS1	SBS17	3832	15783	0.0660988	3.98E-12	8.23E-11
Biliary-AdenoCA	da5b9926-c622-11e3-bf01-24c65	SBS3	SBS6	1361	1376	0.138547	7.82E-12	1.56E-10
Biliary-AdenoCA	da5b9926-c622-11e3-bf01-24c65	SBS3	SBS12	1361	514	0.1029442	0.0007358	0.0049957
Biliary-AdenoCA	da5b9926-c622-11e3-bf01-24c65	SBS6	SBS7	1376	1012	0.1525272	3.29E-12	6.88E-11
Biliary-AdenoCA	da5b9926-c622-11e3-bf01-24c65	SBS6	SBS12	1376	514	0.1021938	0.0008063	0.0053925
Biliary-AdenoCA	da5b9926-c622-11e3-bf01-24c65	SBS6	SBS17	1376	562	0.1930967	2.39E-13	5.52E-12
Biliary-AdenoCA	da5b9926-c622-11e3-bf01-24c65	SBS7	SBS12	1012	514	0.1101721	0.0005097	0.003657
Biliary-AdenoCA	da5b9926-c622-11e3-bf01-24c65	SBS9	SBS17	62	562	0.2338997	0.0044416	0.023274
Biliary-AdenoCA	da5b9926-c622-11e3-bf01-24c65	SBS12	SBS17	514	562	0.1573383	3.38E-06	3.79E-05
Lymph-NOS	f075dcdb-4b6a-4186-bfc5-c1787	SBS9	SBS17	10643	2567	0.0559608	4.74E-06	5.15E-05
Prost-AdenoCA	4c5228b5-bf31-4abd-a47c-d088	SBS8	SBS9	1364	1051	0.1035238	5.96E-06	6.34E-05
Lymph-BNHL	dd8f3e1c-6ed6-41c2-957d-6f679	SBS1	SBS9	330	1207	0.1055459	0.006216	0.0307462
Lymph-BNHL	dd8f3e1c-6ed6-41c2-957d-6f679	SBS1	SBS16	330	1650	0.1321212	0.0001353	0.0011117
Kidney-RCC	15a93657-0521-430a-a816-bf08	SBS3	SBS14	1961	684	0.0985467	0.0001055	0.0008853
Kidney-RCC	15a93657-0521-430a-a816-bf08	SBS4	SBS14	556	684	0.1043165	0.0025244	0.0145006
Skin-Melanoma	a84915de-6562-4836-86f9-f2a05	SBS7	SBS11	113487	7351	0.0261483	0.0001588	0.0012795
Stomach-AdenoCA	bc0dee07-de20-44d6-be65-05af	SBS1	SBS3	5632	4631	0.0367471	0.0020907	0.012382
Stomach-AdenoCA	bc0dee07-de20-44d6-be65-05af	SBS1	SBS8	5632	7834	0.0468425	1.14E-06	1.38E-05
Liver-HCC	fbd899a0-c622-11e3-bf01-24c65	SBS4	SBS5	832	1724	0.1079751	4.15E-06	4.56E-05
Liver-HCC	fbd899a0-c622-11e3-bf01-24c65	SBS4	SBS16	832	12964	0.115375	1.83E-09	3.03E-08
Liver-HCC	fbd899a0-c622-11e3-bf01-24c65	SBS4	SBS17	832	336	0.1180746	0.0025276	0.0145058
Prost-AdenoCA	f9c26646-d2b0-cf30-e040-11ac0	SBS3	SBS6	1562	855	0.0938188	0.0001193	0.0009902
Ovary-AdenoCA	f1504811-8363-41e6-b43c-6245	SBS1	SBS2	760	586	0.3163059	0	0
Ovary-AdenoCA	f1504811-8363-41e6-b43c-6245	SBS1	SBS3	760	1236	0.1292156	2.99E-07	3.92E-06

Ovary-AdenoCA	f1504811-8363-41e6-b43c-6245	SBS1	SBS5	760	2172	0.0893283	0.0002506	0.0019266
Ovary-AdenoCA	f1504811-8363-41e6-b43c-6245	SBS1	SBS13	760	762	0.3705899	0	0
Ovary-AdenoCA	f1504811-8363-41e6-b43c-6245	SBS2	SBS3	586	1236	0.2065123	3.77E-15	1.04E-13
Ovary-AdenoCA	f1504811-8363-41e6-b43c-6245	SBS2	SBS5	586	2172	0.2558548	0	0
Ovary-AdenoCA	f1504811-8363-41e6-b43c-6245	SBS2	SBS8	586	280	0.2885422	3.97E-14	9.88E-13
Ovary-AdenoCA	f1504811-8363-41e6-b43c-6245	SBS2	SBS11	586	1055	0.2693221	0	0
Ovary-AdenoCA	f1504811-8363-41e6-b43c-6245	SBS3	SBS11	1236	1055	0.0857467	0.0004636	0.0033663
Ovary-AdenoCA	f1504811-8363-41e6-b43c-6245	SBS3	SBS13	1236	762	0.2660156	0	0
Ovary-AdenoCA	f1504811-8363-41e6-b43c-6245	SBS5	SBS13	2172	762	0.3115819	0	0
Ovary-AdenoCA	f1504811-8363-41e6-b43c-6245	SBS8	SBS13	280	762	0.3474316	0	0
Ovary-AdenoCA	f1504811-8363-41e6-b43c-6245	SBS11	SBS13	1055	762	0.3239007	0	0
Ovary-AdenoCA	e4aaca83-3ae9-47f6-a975-c144	SBS1	SBS3	1361	9948	0.0791447	6.13E-07	7.68E-06
Ovary-AdenoCA	e4aaca83-3ae9-47f6-a975-c144	SBS1	SBS13	1361	7083	0.0778288	1.97E-06	2.30E-05
Skin-Melanoma	108749d2-5c62-4ef1-92df-aec69	SBS7	SBS11	108670	955	0.0914727	2.64E-07	3.53E-06
Panc-AdenoCA	d91f487e-0895-44ef-aeb1-a62bc	SBS1	SBS9	3013	603	0.0832633	0.0018856	0.0112972
Liver-HCC	1dbdbb2c-c623-11e3-bf01-24c6	SBS4	SBS12	482	5541	0.0790973	0.0077866	0.0372146
Panc-AdenoCA	51458c86-0fdd-470e-b059-1ffbff	SBS1	SBS2	940	2919	0.4791261	0	0
Panc-AdenoCA	51458c86-0fdd-470e-b059-1ffbff	SBS1	SBS3	940	548	0.1250039	4.00E-05	0.0003706
Panc-AdenoCA	51458c86-0fdd-470e-b059-1ffbff	SBS1	SBS11	940	405	0.0996979	0.0072	0.03479
Panc-AdenoCA	51458c86-0fdd-470e-b059-1ffbff	SBS2	SBS3	2919	548	0.3654599	0	0
Panc-AdenoCA	51458c86-0fdd-470e-b059-1ffbff	SBS2	SBS8	2919	532	0.4342781	0	0
Panc-AdenoCA	51458c86-0fdd-470e-b059-1ffbff	SBS2	SBS11	2919	405	0.4033675	0	0
Panc-AdenoCA	51458c86-0fdd-470e-b059-1ffbff	SBS3	SBS8	548	532	0.1042204	0.005679	0.0285405
Breast-AdenoCa	f393bb01-6ed7-9533-e040-11ac	SBS2	SBS3	1121	3750	0.298576	0	0
Breast-AdenoCa	f393bb01-6ed7-9533-e040-11ac	SBS2	SBS11	1121	367	0.2046295	1.76E-10	3.19E-09
Breast-AdenoCa	f393bb01-6ed7-9533-e040-11ac	SBS2	SBS13	1121	1682	0.0869473	7.66E-05	0.0006626
Breast-AdenoCa	f393bb01-6ed7-9533-e040-11ac	SBS3	SBS11	3750	367	0.1094133	0.0006685	0.0046089
Breast-AdenoCa	f393bb01-6ed7-9533-e040-11ac	SBS3	SBS13	3750	1682	0.2226013	0	0
Breast-AdenoCa	f393bb01-6ed7-9533-e040-11ac	SBS11	SBS13	367	1682	0.1243281	0.0001804	0.0014219
Lymph-BNHL	e2fa7251-507e-4d76-95a3-a228	SBS1	SBS5	619	4996	0.0731024	0.0055534	0.0279992
Lymph-BNHL	e2fa7251-507e-4d76-95a3-a228	SBS1	SBS9	619	1020	0.1109094	0.0001532	0.0012369
Lymph-CLL	43d630aa-3890-401c-bf56-e3a9	SBS5	SBS6	2612	586	0.1087851	2.41E-05	0.0002334
Panc-AdenoCA	9011cf17-0783-4f9d-b355-4f0fc1	SBS1	SBS13	3227	346	0.126538	9.01E-05	0.0007676
Panc-AdenoCA	9011cf17-0783-4f9d-b355-4f0fc1	SBS8	SBS13	504	346	0.1261698	0.0029129	0.016402
Panc-AdenoCA	5255d645-d3d2-422e-8449-046c	SBS1	SBS2	4434	3416	0.194145	0	0
Panc-AdenoCA	5255d645-d3d2-422e-8449-046c	SBS1	SBS17	4434	6163	0.2910648	0	0
Panc-AdenoCA	5255d645-d3d2-422e-8449-046c	SBS2	SBS17	3416	6163	0.1030322	0	0
Panc-Endocrine	0e7ac212-0469-4f17-9432-205a	SBS3	SBS4	2624	428	0.1084632	0.0003475	0.0026078
Panc-Endocrine	0e7ac212-0469-4f17-9432-205a	SBS4	SBS15	428	358	0.1331645	0.0019881	0.0118662
Panc-AdenoCA	f4e926fd-006e-4c85-b71c-1433c	SBS1	SBS5	2514	1514	0.1370965	7.77E-16	2.29E-14
Panc-AdenoCA	f4e926fd-006e-4c85-b71c-1433c	SBS1	SBS8	2514	1011	0.1964205	0	0
Panc-AdenoCA	f4e926fd-006e-4c85-b71c-1433c	SBS1	SBS13	2514	803	0.3008483	0	0
Panc-AdenoCA	f4e926fd-006e-4c85-b71c-1433c	SBS5	SBS8	1514	1011	0.0669962	0.008663	0.0404251
Panc-AdenoCA	f4e926fd-006e-4c85-b71c-1433c	SBS5	SBS13	1514	803	0.1733526	4.03E-14	9.98E-13
Panc-AdenoCA	f4e926fd-006e-4c85-b71c-1433c	SBS8	SBS13	1011	803	0.1232002	2.52E-06	2.91E-05
Liver-HCC	b55751c4-c622-11e3-bf01-24c6	SBS1	SBS3	288	346	0.1404945	0.0040394	0.0214715
Liver-HCC	b55751c4-c622-11e3-bf01-24c6	SBS3	SBS12	346	3734	0.0964361	0.0055351	0.027967
Liver-HCC	b55751c4-c622-11e3-bf01-24c6	SBS12	SBS16	3734	2865	0.0404971	0.0098111	0.0446256
Lymph-BNHL	dc4bc4c4-2cc1-4a2e-a9f2-6130	SBS8	SBS17	1928	2566	0.1094378	7.06E-12	1.42E-10
Lymph-BNHL	dc4bc4c4-2cc1-4a2e-a9f2-6130	SBS9	SBS17	2463	2566	0.1020739	8.47E-12	1.68E-10
Lymph-BNHL	dc4bc4c4-2cc1-4a2e-a9f2-6130	SBS10	SBS17	742	2566	0.1080825	2.89E-06	3.28E-05
Lymph-BNHL	dc4bc4c4-2cc1-4a2e-a9f2-6130	SBS16	SBS17	183	2566	0.1519194	0.0007529	0.0050934
Skin-Melanoma	39c476fd-273b-4136-874c-5335	SBS2	SBS7	595	56781	0.1004793	1.37E-05	0.000138
Skin-Melanoma	39c476fd-273b-4136-874c-5335	SBS7	SBS11	56781	3143	0.0583723	3.07E-09	5.00E-08
Lymph-CLL	5b4b2312-acb5-4329-8d46-7f93	SBS1	SBS9	267	196	0.2350378	7.54E-06	7.93E-05
Lymph-CLL	5b4b2312-acb5-4329-8d46-7f93	SBS5	SBS9	2900	196	0.1843983	7.56E-06	7.94E-05
Liver-HCC	cf777ef1-765f-4828-b789-f5aa6	SBS1	SBS16	252	6934	0.1439559	8.40E-05	0.0007214
Panc-AdenoCA	1c28e44a-6e6c-44ed-b58a-e326	SBS1	SBS2	3364	420	0.1248287	1.77E-05	0.0001737
Panc-AdenoCA	9ebac79d-8b38-4469-837e-b834	SBS1	SBS2	3211	1591	0.1554597	0	0
Panc-AdenoCA	9ebac79d-8b38-4469-837e-b834	SBS1	SBS8	3211	3893	0.0642225	9.93E-07	1.21E-05
Panc-AdenoCA	9ebac79d-8b38-4469-837e-b834	SBS1	SBS13	3211	2412	0.131221	0	0
Panc-AdenoCA	9ebac79d-8b38-4469-837e-b834	SBS2	SBS3	1591	714	0.119273	1.63E-06	1.93E-05
Panc-AdenoCA	9ebac79d-8b38-4469-837e-b834	SBS2	SBS8	1591	3893	0.1018956	1.30E-10	2.41E-09
Panc-AdenoCA	9ebac79d-8b38-4469-837e-b834	SBS3	SBS13	714	2412	0.0953182	8.98E-05	0.0007659

Panc-AdenoCA	9ebac79d-8b38-4469-837e-b834	SBS8	SBS13	3893	2412	0.0804859	8.34E-09	1.29E-07
CNS-Medullo	93be2990-bceb-45fd-aca5-3358	SBS1	SBS5	1385	852	0.0791876	0.0026787	0.0152611
Prost-AdenoCA	b33b7c8f-0b0d-4009-88a7-48e9	SBS3	SBS8	743	645	0.1009484	0.0017575	0.0106935
Liver-HCC	5769ae11-5f1b-483f-b228-75845	SBS3	SBS16	1398	465	0.0979371	0.0024772	0.0142948
Liver-HCC	5769ae11-5f1b-483f-b228-75845	SBS4	SBS16	1339	465	0.1015105	0.001629	0.0099892
Liver-HCC	5769ae11-5f1b-483f-b228-75845	SBS6	SBS16	570	465	0.1360498	0.0001527	0.0012365
Liver-HCC	5769ae11-5f1b-483f-b228-75845	SBS11	SBS16	714	465	0.1135538	0.0014031	0.008793
Prost-AdenoCA	0bfe2ac9-0afd-c248-e050-11ac0	SBS1	SBS10	884	562	0.090695	0.0070196	0.0340759
Eso-AdenoCa	9749a9a5-cb7f-4038-a5f9-5e2d6	SBS1	SBS9	8651	2364	0.116608	0	0
Liver-HCC	9aac83e4-c622-11e3-bf01-24c6	SBS4	SBS5	3073	3545	0.0603506	1.24E-05	0.0001254
Liver-HCC	9aac83e4-c622-11e3-bf01-24c6	SBS4	SBS7	3073	1763	0.051525	0.0052204	0.0266854
Liver-HCC	9aac83e4-c622-11e3-bf01-24c6	SBS4	SBS16	3073	1460	0.0518522	0.0097637	0.0445011
Prost-AdenoCA	f9c52187-2e82-d58a-e040-11ac	SBS1	SBS6	1121	1003	0.0768222	0.0038679	0.020792
Liver-HCC	cb86fbb6-c622-11e3-bf01-24c65	SBS12	SBS17	231	264	0.2310606	3.87E-06	4.29E-05
Liver-HCC	cb86fbb6-c622-11e3-bf01-24c65	SBS16	SBS17	6789	264	0.1863543	4.32E-08	6.23E-07
Panc-AdenoCA	dc856038-f5f7-4dfc-a0a4-3e3a6	SBS1	SBS8	2764	1418	0.0584051	0.0033437	0.0184137
Breast-AdenoCa	fc63cbab-d27a-5ebb-e040-11ac	SBS1	SBS3	436	8731	0.0931853	0.0014755	0.0091734
Liver-HCC	dd2e7b3c-c622-11e3-bf01-24c6	SBS12	SBS16	11943	1576	0.0739986	4.78E-07	6.12E-06
Lymph-BNHL	ac2c8928-33a2-4aa0-8bc1-cdfa	SBS1	SBS9	938	5897	0.0577344	0.0090781	0.0420493
Lymph-BNHL	07835447-5d7e-4828-80fd-89b0	SBS1	SBS5	838	5102	0.0735123	0.0008365	0.0055592
Panc-AdenoCA	0972bfcf-c6c6-48cc-b820-cdfa62	SBS1	SBS5	4906	1537	0.1121628	3.25E-13	7.42E-12
Panc-AdenoCA	0972bfcf-c6c6-48cc-b820-cdfa62	SBS1	SBS13	4906	1159	0.0583792	0.0033553	0.0184614
Panc-AdenoCA	0972bfcf-c6c6-48cc-b820-cdfa62	SBS4	SBS5	143	1537	0.158446	0.0028067	0.0158892
Panc-AdenoCA	0972bfcf-c6c6-48cc-b820-cdfa62	SBS5	SBS7	1537	625	0.1396955	5.88E-08	8.38E-07
Panc-AdenoCA	0972bfcf-c6c6-48cc-b820-cdfa62	SBS5	SBS13	1537	1159	0.156452	1.79E-14	4.59E-13
Panc-AdenoCA	8c233a11-3b2e-4273-bbe1-b5a5	SBS1	SBS8	1622	1587	0.0712606	0.0005793	0.0040839
Panc-AdenoCA	1b0fe1d5-b286-4f49-ae92-94d9	SBS1	SBS8	1464	1229	0.0906786	3.38E-05	0.0003181
Panc-AdenoCA	39c6c3b3-4683-4d11-984c-ed58	SBS1	SBS8	6610	781	0.0748497	0.0007982	0.005344
Prost-AdenoCA	dc85552c-2488-48d9-9da1-67a3	SBS3	SBS15	1474	134	0.14654	0.0102314	0.0462962
Eso-AdenoCa	8ff9b1b6-a498-47fe-a970-ab7cf	SBS9	SBS17	4312	40897	0.0580873	7.40E-12	1.48E-10
Prost-AdenoCA	39aee9fc-fa27-4b71-b9e8-43ff3	SBS1	SBS16	1836	178	0.1496169	0.0013995	0.0087792
Prost-AdenoCA	39aee9fc-fa27-4b71-b9e8-43ff3	SBS4	SBS16	972	178	0.186896	5.45E-05	0.0004887
Eso-AdenoCa	da910f22-126e-41dc-be6b-47c8	SBS1	SBS13	13911	3405	0.130346	0	0
Eso-AdenoCa	da910f22-126e-41dc-be6b-47c8	SBS1	SBS17	13911	40599	0.071009	0	0
Eso-AdenoCa	da910f22-126e-41dc-be6b-47c8	SBS13	SBS17	3405	40599	0.0665499	1.64E-12	3.55E-11
Stomach-AdenoCA	dc107863-2c7d-4b19-8afb-666c	SBS1	SBS15	12923	30968	0.0424566	1.05E-14	2.77E-13
Stomach-AdenoCA	dc107863-2c7d-4b19-8afb-666c	SBS6	SBS15	21181	30968	0.032032	1.23E-11	2.42E-10
Ovary-AdenoCA	941fcb56-e059-403d-aab1-0692	SBS1	SBS3	716	5873	0.0877979	0.0001067	0.0008904
Ovary-AdenoCA	941fcb56-e059-403d-aab1-0692	SBS1	SBS9	716	260	0.1468414	0.0005355	0.0038249
Eso-AdenoCa	e91ab7da-79a6-4ea0-bc24-7aac	SBS1	SBS14	8720	970	0.0581564	0.0054538	0.0276754
Eso-AdenoCa	e91ab7da-79a6-4ea0-bc24-7aac	SBS1	SBS17	8720	19999	0.0525465	5.44E-15	1.47E-13
Eso-AdenoCa	e91ab7da-79a6-4ea0-bc24-7aac	SBS9	SBS17	4391	19999	0.0646072	1.77E-13	4.15E-12
Eso-AdenoCa	e91ab7da-79a6-4ea0-bc24-7aac	SBS14	SBS17	970	19999	0.0993595	2.33E-08	3.45E-07
Prost-AdenoCA	dcc938da-3e45-4c2f-ae0f-47817	SBS1	SBS5	434	2342	0.2128572	7.77E-15	2.07E-13
Prost-AdenoCA	dcc938da-3e45-4c2f-ae0f-47817	SBS1	SBS9	434	177	0.3688589	2.78E-15	7.77E-14
Prost-AdenoCA	dcc938da-3e45-4c2f-ae0f-47817	SBS5	SBS9	2342	177	0.1778358	6.03E-05	0.0005371
Prost-AdenoCA	a08ec059-7592-4698-bb45-25a9	SBS1	SBS5	600	6361	0.2067804	0	0
Prost-AdenoCA	a08ec059-7592-4698-bb45-25a9	SBS1	SBS8	600	391	0.1132353	0.0046185	0.0240801
Prost-AdenoCA	a08ec059-7592-4698-bb45-25a9	SBS5	SBS8	6361	391	0.1048613	0.0006066	0.0042568
Breast-AdenoCa	fc9f6cb0-ceb9-790d-e040-11ac0	SBS1	SBS2	486	952	0.0995089	0.0034178	0.0187398
Breast-AdenoCa	fc9f6cb0-ceb9-790d-e040-11ac0	SBS1	SBS3	486	6686	0.0944003	0.0006225	0.0043491
Breast-AdenoCa	fc9f6cb0-ceb9-790d-e040-11ac0	SBS1	SBS13	486	1146	0.1243186	5.24E-05	0.000473
Breast-AdenoCa	fc9f6cb0-ceb9-790d-e040-11ac0	SBS2	SBS3	952	6686	0.1619884	0	0
Breast-AdenoCa	fc9f6cb0-ceb9-790d-e040-11ac0	SBS2	SBS12	952	134	0.158049	0.0056543	0.0284396
Breast-AdenoCa	fc9f6cb0-ceb9-790d-e040-11ac0	SBS3	SBS13	6686	1146	0.1908888	0	0
Breast-AdenoCa	fc9f6cb0-ceb9-790d-e040-11ac0	SBS12	SBS13	134	1146	0.1990701	0.0001484	0.0012079
Panc-AdenoCA	bbdd7393-024b-4073-b5bc-ef7a	SBS1	SBS3	893	13465	0.1420819	4.11E-15	1.12E-13
Panc-AdenoCA	bbdd7393-024b-4073-b5bc-ef7a	SBS1	SBS7	893	1675	0.1061176	4.02E-06	4.42E-05
Panc-AdenoCA	bbdd7393-024b-4073-b5bc-ef7a	SBS1	SBS13	893	2210	0.0746566	0.0016673	0.010214
Panc-AdenoCA	bbdd7393-024b-4073-b5bc-ef7a	SBS3	SBS7	13465	1675	0.0474823	0.0024198	0.0140677
Panc-AdenoCA	bbdd7393-024b-4073-b5bc-ef7a	SBS3	SBS13	13465	2210	0.0895361	1.21E-13	2.88E-12
Eso-AdenoCa	8e48aa43-6cd1-4aef-83ce-2451	SBS8	SBS17	5459	23841	0.0704541	0	0
Panc-Endocrine	4b5d9d8c-ff95-45f4-9287-2804e	SBS1	SBS12	219	358	0.1406979	0.0092186	0.0425738
Liver-HCC	17de5b12-c623-11e3-bf01-24c6	SBS3	SBS8	1410	4431	0.0787272	3.49E-06	3.90E-05

Liver-HCC	17de5b12-c623-11e3-bf01-24c6!	SBS3	SBS12	1410	4476	0.1410587	0	0
Liver-HCC	17de5b12-c623-11e3-bf01-24c6!	SBS8	SBS12	4431	4476	0.0662232	6.59E-09	1.03E-07
Eso-AdenoCa	b02b4bba-6e66-44fb-a48f-38c3(	SBS1	SBS17	11739	3339	0.0949959	0	0
Eso-AdenoCa	b02b4bba-6e66-44fb-a48f-38c3(	SBS6	SBS17	2016	3339	0.0838668	4.18E-08	6.07E-07
Eso-AdenoCa	b02b4bba-6e66-44fb-a48f-38c3(	SBS10	SBS17	573	3339	0.1021389	7.40E-05	0.0006425
Ovary-AdenoCA	01df36af-3617-40fc-9892-f54ce4	SBS1	SBS2	1988	530	0.0837705	0.0056297	0.0283382
Ovary-AdenoCA	01df36af-3617-40fc-9892-f54ce4	SBS1	SBS5	1988	2176	0.0522834	0.0068295	0.0332818
Lymph-BNHL	02917220-6a7a-46a1-8656-907e	SBS9	SBS15	6076	1917	0.0644584	1.10E-05	0.0001124
Lymph-BNHL	05616329-e7ba-4efd-87b1-d79c	SBS6	SBS16	2199	5042	0.0453527	0.0036766	0.0199662
Lymph-BNHL	05616329-e7ba-4efd-87b1-d79c	SBS6	SBS17	2199	947	0.0712122	0.0024286	0.0140926
Prost-AdenoCA	0cb4356c-b5d6-4541-b048-ff6d4	SBS1	SBS7	767	688	0.100213	0.0013722	0.0086428
Prost-AdenoCA	0cb4356c-b5d6-4541-b048-ff6d4	SBS1	SBS8	767	1126	0.0769543	0.0090011	0.0418132
Eso-AdenoCa	0ef92ff8-829f-425a-91d8-c594b6	SBS1	SBS17	7741	5884	0.0400796	4.33E-05	0.0003975

Supplementary Table 6. Kataegis events and mutation signatures. A total of 7482 kataegic events identified in nonhypermutated genomes are listed. MafTool-based output are available for individual kataegic events (genomic coordinates and average intermediate size). The numbe rof mutations in terms of six mutation spectra are available. Significance of enrichment was estimated by Fisher's exact test across mutation signatures.

							Six mutation spectra					
Tumor type	ICGC ref ID	Chr	Start	End	Mutations	Size (bp)	C-to-A	C-to-G	C-to-T	T-to-C	T-to-G	T-to-A
Ovary-AdenoCA	0009b464-b376-4fbc-8a56-da538269a02f	6	152489270	152489836	6	566	2	3	1	NA	NA	NA
Ovary-AdenoCA	0009b464-b376-4fbc-8a56-da538269a02f	6	154665662	154666756	10	1094	1	4	5	NA	NA	NA
Ovary-AdenoCA	0009b464-b376-4fbc-8a56-da538269a02f	7	86703542	86706397	17	2855	3	4	10	NA	NA	NA
Ovary-AdenoCA	0009b464-b376-4fbc-8a56-da538269a02f	7	87247570	87248197	9	627	2	1	6	NA	NA	NA
Ovary-AdenoCA	0009b464-b376-4fbc-8a56-da538269a02f	8	68622138	68628057	8	5919	3	2	3	NA	NA	NA
Ovary-AdenoCA	0009b464-b376-4fbc-8a56-da538269a02f	8	95198600	95203600	8	5000	1	1	6	NA	NA	NA
Ovary-AdenoCA	0009b464-b376-4fbc-8a56-da538269a02f	8	126314418	126324739	14	10321	2	NA	11	1	NA	NA
Ovary-AdenoCA	0009b464-b376-4fbc-8a56-da538269a02f	19	9492623	9493131	6	508	NA	3	3	NA	NA	NA
Ovary-AdenoCA	0009b464-b376-4fbc-8a56-da538269a02f	20	40393279	40395622	8	2343	1	2	5	NA	NA	NA
Ovary-AdenoCA	0009b464-b376-4fbc-8a56-da538269a02f	20	40644670	40646723	7	2053	NA	3	4	NA	NA	NA
Ovary-AdenoCA	0009b464-b376-4fbc-8a56-da538269a02f	20	42672605	42677990	9	5385	2	4	3	NA	NA	NA
Ovary-AdenoCA	0009b464-b376-4fbc-8a56-da538269a02f	20	50115928	50123941	13	8013	2	4	7	NA	NA	NA
Ovary-AdenoCA	0009b464-b376-4fbc-8a56-da538269a02f	20	60699024	60702525	9	3501	2	5	2	NA	NA	NA
Kidney-RCC	005794f1-5a87-45b5-9811-83ddf6924568	3	78322918	78323308	8	390	3	2	3	NA	NA	NA
Kidney-RCC	005794f1-5a87-45b5-9811-83ddf6924568	3	79377488	79380507	9	3019	2	5	2	NA	NA	NA
Kidney-RCC	005794f1-5a87-45b5-9811-83ddf6924568	3	83601421	83604610	10	3189	4	3	3	NA	NA	NA
Lymph-BNHL	00b9d0e6-69dc-4345-bffd-ce32880c8eef	2	89157943	89165713	32	7770	2	7	8	9	3	3
Lymph-BNHL	00b9d0e6-69dc-4345-bffd-ce32880c8eef	8	128748722	128751971	13	3249	NA	3	8	2	NA	NA
Lymph-BNHL	00b9d0e6-69dc-4345-bffd-ce32880c8eef	14	106324422	106330300	76	5878	2	20	27	14	5	8
Lymph-BNHL	00b9d0e6-69dc-4345-bffd-ce32880c8eef	14	107083392	107084387	12	995	NA	1	7	3	1	NA
Liver-HCC	00c27940-c623-11e3-bf01-24c6515278c0	10	49741959	49743806	6	1847	1	NA	5	NA	NA	NA
Kidney-RCC	00db1b95-8ca3-4cc4-bb46-6b8c8019a7c7	8	114711968	114713243	7	1275	NA	NA	6	NA	1	NA
Kidney-RCC	00db1b95-8ca3-4cc4-bb46-6b8c8019a7c7	10	9670842	9672753	6	1911	NA	6	NA	NA	NA	NA
Kidney-RCC	00db1b95-8ca3-4cc4-bb46-6b8c8019a7c7	10	25601481	25607855	14	6374	7	3	4	NA	NA	NA
Kidney-RCC	00db1b95-8ca3-4cc4-bb46-6b8c8019a7c7	10	32190748	32200493	24	9745	6	9	8	NA	1	NA
Uterus-AdenoCA	00db4dc2-3ec7-4ff9-9233-d69c8c8a607f	4	45680606	45681275	8	669	2	2	4	NA	NA	NA
Uterus-AdenoCA	00db4dc2-3ec7-4ff9-9233-d69c8c8a607f	16	68384859	68386849	16	1990	4	6	6	NA	NA	NA
Uterus-AdenoCA	00db4dc2-3ec7-4ff9-9233-d69c8c8a607f	17	19720021	19721089	7	1068	2	3	2	NA	NA	NA
Uterus-AdenoCA	00db4dc2-3ec7-4ff9-9233-d69c8c8a607f	19	41642722	41646606	9	3884	1	3	3	NA	NA	2
Breast-AdenoCa	01658141-8398-4585-9f0f-8355dd9b0604	1	16259336	16263369	11	4033	1	5	5	NA	NA	NA
Breast-AdenoCa	01658141-8398-4585-9f0f-8355dd9b0604	1	41880948	41884811	9	3863	NA	5	4	NA	NA	NA
Breast-AdenoCa	01658141-8398-4585-9f0f-8355dd9b0604	1	174835785	174836692	7	907	1	NA	6	NA	NA	NA
Breast-AdenoCa	01658141-8398-4585-9f0f-8355dd9b0604	2	62731333	62735782	8	4449	NA	7	1	NA	NA	NA
Breast-AdenoCa	01658141-8398-4585-9f0f-8355dd9b0604	2	65174550	65176816	7	2266	NA	1	6	NA	NA	NA
Breast-AdenoCa	01658141-8398-4585-9f0f-8355dd9b0604	2	128775791	128778480	7	2689	NA	3	4	NA	NA	NA
Breast-AdenoCa	01658141-8398-4585-9f0f-8355dd9b0604	2	242532542	242537171	13	4629	1	5	7	NA	NA	NA
Breast-AdenoCa	01658141-8398-4585-9f0f-8355dd9b0604	3	112944035	112945519	8	1484	NA	2	6	NA	NA	NA
Breast-AdenoCa	01658141-8398-4585-9f0f-8355dd9b0604	3	192157595	192163308	11	5713	NA	4	7	NA	NA	NA
Breast-AdenoCa	01658141-8398-4585-9f0f-8355dd9b0604	3	192168780	192172814	9	4034	1	6	1	NA	1	NA
Breast-AdenoCa	01658141-8398-4585-9f0f-8355dd9b0604	4	169677113	169682344	9	5231	NA	5	3	NA	1	NA
Breast-AdenoCa	01658141-8398-4585-9f0f-8355dd9b0604	5	71008218	71012224	12	4006	NA	5	7	NA	NA	NA
Breast-AdenoCa	01658141-8398-4585-9f0f-8355dd9b0604	6	44361443	44367776	15	6333	3	7	5	NA	NA	NA
Breast-AdenoCa	01658141-8398-4585-9f0f-8355dd9b0604	6	44378143	44453598	95	75455	13	17	65	NA	NA	NA
Breast-AdenoCa	01658141-8398-4585-9f0f-8355dd9b0604	6	44619410	44628077	10	8667	3	4	3	NA	NA	NA
Breast-AdenoCa	01658141-8398-4585-9f0f-8355dd9b0604	6	44629412	44634546	7	5134	NA	2	4	NA	NA	1
Breast-AdenoCa	01658141-8398-4585-9f0f-8355dd9b0604	6	71808641	71809870	11	1229	2	8	1	NA	NA	NA
Breast-AdenoCa	01658141-8398-4585-9f0f-8355dd9b0604	6	151906218	151912189	8	5971	NA	3	5	NA	NA	NA
Breast-AdenoCa	01658141-8398-4585-9f0f-8355dd9b0604	7	2040759	2046714	7	5955	NA	1	6	NA	NA	NA
Breast-AdenoCa	01658141-8398-4585-9f0f-8355dd9b0604	8	129124851	129127643	6	2792	1	1	4	NA	NA	NA
Breast-AdenoCa	01658141-8398-4585-9f0f-8355dd9b0604	9	127212934	127217554	8	4620	1	6	1	NA	NA	NA
Breast-AdenoCa	01658141-8398-4585-9f0f-8355dd9b0604	10	83877093	83879807	8	2714	1	4	3	NA	NA	NA

Breast-AdenoCa	01658141-8398-4585-9f0f-8355dd9b0604	11	64937301	64939235	7	1934	NA	4	3	NA	NA	NA
Breast-AdenoCa	01658141-8398-4585-9f0f-8355dd9b0604	12	9249429	9272490	31	23061	2	10	19	NA	NA	NA
Breast-AdenoCa	01658141-8398-4585-9f0f-8355dd9b0604	12	9282496	9298670	23	16174	1	3	17	NA	NA	2
Breast-AdenoCa	01658141-8398-4585-9f0f-8355dd9b0604	12	33900621	33903588	11	2967	1	3	7	NA	NA	NA
Breast-AdenoCa	01658141-8398-4585-9f0f-8355dd9b0604	12	34012709	34015324	7	2615	1	5	1	NA	NA	NA
Breast-AdenoCa	01658141-8398-4585-9f0f-8355dd9b0604	14	34282153	34282331	6	178	NA	3	3	NA	NA	NA
Breast-AdenoCa	01658141-8398-4585-9f0f-8355dd9b0604	14	51730750	51731888	12	1138	4	3	5	NA	NA	NA
Breast-AdenoCa	01658141-8398-4585-9f0f-8355dd9b0604	14	55517809	55519868	7	2059	1	5	1	NA	NA	NA
Breast-AdenoCa	01658141-8398-4585-9f0f-8355dd9b0604	17	20934109	20938625	6	4516	3	2	1	NA	NA	NA
Breast-AdenoCa	01658141-8398-4585-9f0f-8355dd9b0604	17	64983584	64987556	8	3972	NA	3	5	NA	NA	NA
Breast-AdenoCa	01658141-8398-4585-9f0f-8355dd9b0604	19	49428778	49432870	6	4092	NA	2	4	NA	NA	NA
Breast-AdenoCa	01658141-8398-4585-9f0f-8355dd9b0604	22	38493315	38498842	7	5527	1	2	3	NA	NA	1
Breast-AdenoCa	01658141-8398-4585-9f0f-8355dd9b0604	22	38503191	38512084	10	8893	NA	3	7	NA	NA	NA
Breast-AdenoCa	01658141-8398-4585-9f0f-8355dd9b0604	22	38588073	38590606	7	2533	1	1	5	NA	NA	NA
Breast-AdenoCa	01658141-8398-4585-9f0f-8355dd9b0604	X	68691208	68693179	11	1971	NA	5	5	1	NA	NA
Panc-Endocrine	01c8e465-2b4e-4519-bdef-c3ac06b43eeb	2	148978804	148981913	6	3109	NA	2	NA	3	NA	1
Liver-HCC	01dc6872-c623-11e3-bf01-24c6515278c0	3	110361112	110362154	8	1042	NA	5	3	NA	NA	NA
Liver-HCC	01dc6872-c623-11e3-bf01-24c6515278c0	18	24627940	24628704	10	764	NA	4	3	1	2	NA
Ovary-AdenoCA	01df36af-3617-40fc-9892-f54ce433cf71	12	47326945	47328112	7	1167	NA	3	4	NA	NA	NA
Ovary-AdenoCA	01df36af-3617-40fc-9892-f54ce433cf71	19	34062845	34063872	8	1027	NA	4	4	NA	NA	NA
Lung-AdenoCA	020fab36-c7de-4933-b2bf-dc7b019a1326	4	48359941	48360051	6	110	NA	4	2	NA	NA	NA
Lung-AdenoCA	020fab36-c7de-4933-b2bf-dc7b019a1326	7	25190313	25194418	12	4105	NA	1	11	NA	NA	NA
Lung-AdenoCA	020fab36-c7de-4933-b2bf-dc7b019a1326	8	64624458	64625906	8	1448	1	6	1	NA	NA	NA
Lung-AdenoCA	020fab36-c7de-4933-b2bf-dc7b019a1326	8	67101066	67108021	9	6955	1	1	7	NA	NA	NA
Lung-AdenoCA	020fab36-c7de-4933-b2bf-dc7b019a1326	8	100017742	100020427	12	2685	NA	8	4	NA	NA	NA
Lung-AdenoCA	020fab36-c7de-4933-b2bf-dc7b019a1326	8	100960854	100962220	14	1366	NA	NA	9	2	2	1
Lung-AdenoCA	020fab36-c7de-4933-b2bf-dc7b019a1326	8	118700611	118708880	12	8269	2	4	6	NA	NA	NA
Lung-AdenoCA	020fab36-c7de-4933-b2bf-dc7b019a1326	16	49268361	49273693	9	5332	NA	3	6	NA	NA	NA
Lung-AdenoCA	020fab36-c7de-4933-b2bf-dc7b019a1326	16	49327513	49330221	11	2708	NA	1	10	NA	NA	NA
Lung-AdenoCA	020fab36-c7de-4933-b2bf-dc7b019a1326	16	57952471	57960460	15	7989	2	1	12	NA	NA	NA
Lung-AdenoCA	020fab36-c7de-4933-b2bf-dc7b019a1326	16	62155780	62157535	8	1755	NA	1	7	NA	NA	NA
Lung-AdenoCA	020fab36-c7de-4933-b2bf-dc7b019a1326	16	62164806	62167919	20	3113	NA	1	19	NA	NA	NA
Lung-AdenoCA	020fab36-c7de-4933-b2bf-dc7b019a1326	17	58074967	58076299	8	1332	NA	NA	1	2	4	1
Lung-AdenoCA	020fab36-c7de-4933-b2bf-dc7b019a1326	22	45283801	45284658	8	857	NA	NA	8	NA	NA	NA
Lymph-BNHL	02917220-6a7a-46a1-8656-907e96bef88e	2	109503584	109503677	7	93	NA	NA	NA	4	NA	3
Lymph-BNHL	02917220-6a7a-46a1-8656-907e96bef88e	3	27422936	27427358	6	4422	NA	NA	1	1	NA	4
Lymph-BNHL	02917220-6a7a-46a1-8656-907e96bef88e	3	39893952	39899157	8	5205	NA	NA	2	3	2	1
Lymph-BNHL	02917220-6a7a-46a1-8656-907e96bef88e	3	60356594	60359493	7	2899	NA	1	NA	NA	2	4
Lymph-BNHL	02917220-6a7a-46a1-8656-907e96bef88e	3	60510982	60518899	9	7917	NA	1	2	1	1	4
Lymph-BNHL	02917220-6a7a-46a1-8656-907e96bef88e	3	60636379	60640315	7	3936	1	1	NA	1	2	2
Lymph-BNHL	02917220-6a7a-46a1-8656-907e96bef88e	3	60657154	60664070	8	6916	2	NA	1	2	1	2
Lymph-BNHL	02917220-6a7a-46a1-8656-907e96bef88e	3	60665717	60670686	7	4969	2	1	2	1	1	NA
Lymph-BNHL	02917220-6a7a-46a1-8656-907e96bef88e	3	60673374	60692771	27	19397	4	NA	4	7	7	5
Lymph-BNHL	02917220-6a7a-46a1-8656-907e96bef88e	3	60887622	60893018	7	5396	NA	NA	NA	1	2	4
Lymph-BNHL	02917220-6a7a-46a1-8656-907e96bef88e	3	76444957	76460038	40	15081	NA	1	NA	15	9	15
Lymph-BNHL	02917220-6a7a-46a1-8656-907e96bef88e	3	149638427	149638714	7	287	NA	NA	NA	5	1	1
Lymph-BNHL	02917220-6a7a-46a1-8656-907e96bef88e	4	14194885	14196904	6	2019	NA	1	1	4	NA	NA
Lymph-BNHL	02917220-6a7a-46a1-8656-907e96bef88e	4	136538498	136541864	9	3366	NA	1	NA	3	4	1
Lymph-BNHL	02917220-6a7a-46a1-8656-907e96bef88e	7	52184197	52188706	6	4509	NA	NA	2	2	NA	2
Lymph-BNHL	02917220-6a7a-46a1-8656-907e96bef88e	9	11221917	11226114	7	4197	NA	NA	NA	1	2	4
Lymph-BNHL	02917220-6a7a-46a1-8656-907e96bef88e	10	58176246	58176789	6	543	NA	NA	NA	3	2	1
Lymph-BNHL	02917220-6a7a-46a1-8656-907e96bef88e	10	84747682	84749029	7	1347	1	NA	NA	2	3	1
Lymph-BNHL	02917220-6a7a-46a1-8656-907e96bef88e	13	35979390	35982170	9	2780	NA	NA	NA	2	4	3
Lymph-BNHL	02917220-6a7a-46a1-8656-907e96bef88e	14	38408661	38411626	12	2965	1	1	NA	1	6	3
Lymph-BNHL	02917220-6a7a-46a1-8656-907e96bef88e	14	106326040	106330379	41	4339	2	11	10	10	5	3
Lymph-BNHL	02917220-6a7a-46a1-8656-907e96bef88e	14	107170096	107179611	21	9515	NA	6	4	5	5	1
Lymph-BNHL	02917220-6a7a-46a1-8656-907e96bef88e	15	55820017	55821998	7	1981	NA	NA	1	5	1	NA
Lymph-BNHL	02917220-6a7a-46a1-8656-907e96bef88e	18	60984802	60988554	42	3752	1	8	5	13	7	8
Lymph-BNHL	02917220-6a7a-46a1-8656-907e96bef88e	22	22516598	22517365	12	767	NA	NA	2	3	4	3
Lymph-BNHL	02917220-6a7a-46a1-8656-907e96bef88e	22	23039606	23040615	8	1009	2	1	NA	2	2	1

Head-SCC	02c6a893-49c5-49d1-8eb1-195021e70d52	10	89669909	89674620	7	4711	2	2	2	NA	1	NA
Head-SCC	02c6a893-49c5-49d1-8eb1-195021e70d52	12	1550635	1556505	14	5870	2	4	8	NA	NA	NA
Panc-AdenoCA	02c97e2b-914e-4afc-bf50-78f0cfbfa67b	7	46045837	46059972	34	14135	3	13	17	NA	NA	1
Panc-AdenoCA	02e5c36f-5bec-45e2-a048-875653b85ca1	6	63101335	63102511	12	1176	1	5	5	NA	NA	1
Panc-AdenoCA	02e5c36f-5bec-45e2-a048-875653b85ca1	8	38349037	38350478	6	1441	NA	2	4	NA	NA	NA
Panc-AdenoCA	02e5c36f-5bec-45e2-a048-875653b85ca1	14	61147479	61147599	6	120	NA	2	4	NA	NA	NA
Panc-AdenoCA	02e5c36f-5bec-45e2-a048-875653b85ca1	18	22520919	22525681	6	4762	NA	2	4	NA	NA	NA
Panc-AdenoCA	02e5c36f-5bec-45e2-a048-875653b85ca1	18	24596245	24602197	41	5952	7	12	22	NA	NA	NA
Panc-AdenoCA	02e5c36f-5bec-45e2-a048-875653b85ca1	18	25666546	25676048	13	9502	1	7	5	NA	NA	NA
Panc-AdenoCA	02e5c36f-5bec-45e2-a048-875653b85ca1	18	26232812	26234350	10	1538	2	2	6	NA	NA	NA
Liver-HCC	030695f6-c623-11e3-bf01-24c6515278c0	7	2833577	2835845	10	2268	NA	9	1	NA	NA	NA
Liver-HCC	030695f6-c623-11e3-bf01-24c6515278c0	8	35446715	35449391	9	2676	1	5	3	NA	NA	NA
Lymph-BNHL	03ad38a6-0902-4aaa-84a3-91ea88fa9883	14	106210978	106213823	6	2845	1	1	4	NA	NA	NA
Lymph-BNHL	03ad38a6-0902-4aaa-84a3-91ea88fa9883	14	106325625	106330111	75	4486	2	15	27	19	9	3
Lymph-BNHL	03ad38a6-0902-4aaa-84a3-91ea88fa9883	18	60983692	60988293	17	4601	NA	4	5	4	2	2
Lymph-BNHL	03ad38a6-0902-4aaa-84a3-91ea88fa9883	22	22749951	22750340	6	389	NA	2	NA	2	1	1
Lymph-BNHL	03ad38a6-0902-4aaa-84a3-91ea88fa9883	22	22758914	22768645	14	9731	1	2	4	4	2	1
Lymph-BNHL	03ad38a6-0902-4aaa-84a3-91ea88fa9883	22	23223227	23231759	28	8532	1	3	7	7	5	5
Panc-AdenoCA	03c3c692-8a86-4843-85ae-e045f0fa6f88	1	32000484	32003537	11	3053	NA	NA	1	10	NA	NA
Panc-AdenoCA	03c3c692-8a86-4843-85ae-e045f0fa6f88	1	217752959	217753591	10	632	1	3	6	NA	NA	NA
Panc-AdenoCA	03c3c692-8a86-4843-85ae-e045f0fa6f88	2	78916046	78924194	11	8148	1	4	6	NA	NA	NA
Panc-AdenoCA	03c3c692-8a86-4843-85ae-e045f0fa6f88	3	134287405	134311503	28	24098	7	7	13	NA	NA	1
Panc-AdenoCA	03c3c692-8a86-4843-85ae-e045f0fa6f88	4	184457776	184460536	9	2760	1	5	2	NA	NA	1
Panc-AdenoCA	03c3c692-8a86-4843-85ae-e045f0fa6f88	4	187359796	187365414	7	5618	NA	2	5	NA	NA	NA
Panc-AdenoCA	03c3c692-8a86-4843-85ae-e045f0fa6f88	4	189770187	189772938	13	2751	3	6	4	NA	NA	NA
Panc-AdenoCA	03c3c692-8a86-4843-85ae-e045f0fa6f88	5	178680806	178690668	12	9862	1	2	9	NA	NA	NA
Panc-AdenoCA	03c3c692-8a86-4843-85ae-e045f0fa6f88	6	13363875	13364459	7	584	2	2	3	NA	NA	NA
Panc-AdenoCA	03c3c692-8a86-4843-85ae-e045f0fa6f88	6	31561194	31599640	41	38446	11	9	21	NA	NA	NA
Panc-AdenoCA	03c3c692-8a86-4843-85ae-e045f0fa6f88	9	27716355	27717415	8	1060	NA	2	6	NA	NA	NA
Panc-AdenoCA	03c3c692-8a86-4843-85ae-e045f0fa6f88	15	87409500	87415192	7	5692	NA	NA	6	1	NA	NA
Panc-AdenoCA	03c3c692-8a86-4843-85ae-e045f0fa6f88	15	87418917	87433166	16	14249	7	3	6	NA	NA	NA
Liver-HCC	03c88506-d72e-4a44-a34e-a7f0564f1799	9	21007271	21008235	6	964	NA	3	2	NA	1	NA
Liver-HCC	03c88506-d72e-4a44-a34e-a7f0564f1799	9	21097751	21100293	18	2542	2	7	9	NA	NA	NA
Bone-Leiomyo	03ced0ce-186a-4349-8d98-572c2bc90382	3	141084599	141087365	8	2766	NA	2	6	NA	NA	NA
Bone-Leiomyo	03ced0ce-186a-4349-8d98-572c2bc90382	3	145511316	145512622	9	1306	1	3	5	NA	NA	NA
Bone-Leiomyo	03ced0ce-186a-4349-8d98-572c2bc90382	3	172473785	172481110	28	7325	2	19	6	NA	NA	1
Bone-Leiomyo	03ced0ce-186a-4349-8d98-572c2bc90382	10	113458726	113467917	13	9191	NA	NA	13	NA	NA	NA
Bone-Leiomyo	03ced0ce-186a-4349-8d98-572c2bc90382	16	78675497	78681191	19	5694	6	11	2	NA	NA	NA
Bone-Leiomyo	03ced0ce-186a-4349-8d98-572c2bc90382	X	22843801	22844117	6	316	3	3	NA	NA	NA	NA
ColoRect-AdenoCA	03cff38d-7e29-4409-a508-749bddb1b3df	4	67820677	67821986	9	1309	NA	7	2	NA	NA	NA
ColoRect-AdenoCA	03cff38d-7e29-4409-a508-749bddb1b3df	5	39153316	39153644	6	328	2	2	2	NA	NA	NA
ColoRect-AdenoCA	03cff38d-7e29-4409-a508-749bddb1b3df	10	28165507	28168205	7	2698	NA	3	3	1	NA	NA
ColoRect-AdenoCA	03cff38d-7e29-4409-a508-749bddb1b3df	16	69461018	69462594	10	1576	1	3	6	NA	NA	NA
CNS-GBM	04339769-517c-448d-a7ca-951f83608c60	12	58496920	58497284	6	364	NA	NA	6	NA	NA	NA
CNS-GBM	04339769-517c-448d-a7ca-951f83608c60	X	80821276	80825050	6	3774	NA	3	3	NA	NA	NA
Breast-AdenoCa	0448206f-3ade-4087-b1a9-4fb2d14e1367	2	117253143	117257980	6	4837	2	3	1	NA	NA	NA
Breast-AdenoCa	0448206f-3ade-4087-b1a9-4fb2d14e1367	2	117261083	117270327	18	9244	2	13	2	NA	1	NA
Breast-AdenoCa	0448206f-3ade-4087-b1a9-4fb2d14e1367	2	134116676	134117603	7	927	2	4	1	NA	NA	NA
Breast-AdenoCa	0448206f-3ade-4087-b1a9-4fb2d14e1367	3	160952012	160957592	13	5580	5	5	2	NA	NA	1
Breast-AdenoCa	0448206f-3ade-4087-b1a9-4fb2d14e1367	6	115573852	115581389	11	7537	3	7	1	NA	NA	NA
Breast-AdenoCa	0448206f-3ade-4087-b1a9-4fb2d14e1367	11	3678889	3680391	7	1502	2	4	1	NA	NA	NA
Breast-AdenoCa	0448206f-3ade-4087-b1a9-4fb2d14e1367	11	41387450	41391202	8	3752	1	6	1	NA	NA	NA
Breast-AdenoCa	0448206f-3ade-4087-b1a9-4fb2d14e1367	11	42914614	42917438	10	2824	2	7	1	NA	NA	NA
Breast-AdenoCa	0448206f-3ade-4087-b1a9-4fb2d14e1367	13	19511446	19517074	10	5628	NA	5	5	NA	NA	NA
Breast-AdenoCa	0448206f-3ade-4087-b1a9-4fb2d14e1367	16	51865840	51866894	7	1054	1	2	4	NA	NA	NA
Breast-AdenoCa	0448206f-3ade-4087-b1a9-4fb2d14e1367	20	4074223	4078598	11	4375	3	2	6	NA	NA	NA
Breast-AdenoCa	0448206f-3ade-4087-b1a9-4fb2d14e1367	21	48003171	48005079	7	1908	2	4	1	NA	NA	NA
Panc-AdenoCA	046d7386-95c8-4501-9e55-c85bec272a7a	1	4091340	4092487	19	1147	5	9	5	NA	NA	NA
Panc-AdenoCA	046d7386-95c8-4501-9e55-c85bec272a7a	1	5782556	5784975	9	2419	NA	3	6	NA	NA	NA
Panc-AdenoCA	046d7386-95c8-4501-9e55-c85bec272a7a	4	29301483	29301935	10	452	2	5	3	NA	NA	NA

Panc-AdenoCA	046d7386-95c8-4501-9e55-c85bec272a7a	9	18786650	18787252	7	602	NA	2	5	NA	NA	NA
Panc-AdenoCA	046d7386-95c8-4501-9e55-c85bec272a7a	9	21930139	21930486	7	347	NA	2	5	NA	NA	NA
Panc-AdenoCA	046d7386-95c8-4501-9e55-c85bec272a7a	11	119409261	119413179	19	3918	3	4	12	NA	NA	NA
Panc-AdenoCA	046d7386-95c8-4501-9e55-c85bec272a7a	X	77579618	77580230	7	612	NA	2	5	NA	NA	NA
Panc-AdenoCA	046d7386-95c8-4501-9e55-c85bec272a7a	X	144302192	144304283	19	2091	2	4	13	NA	NA	NA
Cervix-SCC	047f9e4d-86b5-4943-aef5-68199bf29e8c	2	29105823	29108799	7	2976	NA	5	1	1	NA	NA
Cervix-SCC	047f9e4d-86b5-4943-aef5-68199bf29e8c	6	3837899	3843496	8	5597	1	1	6	NA	NA	NA
Cervix-SCC	047f9e4d-86b5-4943-aef5-68199bf29e8c	6	7407302	7410453	12	3151	1	3	8	NA	NA	NA
Cervix-SCC	047f9e4d-86b5-4943-aef5-68199bf29e8c	9	134563771	134567287	7	3516	1	3	3	NA	NA	NA
Cervix-SCC	047f9e4d-86b5-4943-aef5-68199bf29e8c	12	86381814	86385676	12	3862	2	1	9	NA	NA	NA
Cervix-SCC	047f9e4d-86b5-4943-aef5-68199bf29e8c	16	80281696	80282642	6	946	2	2	2	NA	NA	NA
Cervix-SCC	047f9e4d-86b5-4943-aef5-68199bf29e8c	17	26775069	26776227	7	1158	1	6	NA	NA	NA	NA
Cervix-SCC	047f9e4d-86b5-4943-aef5-68199bf29e8c	17	73873476	73875438	8	1962	1	1	6	NA	NA	NA
Cervix-SCC	047f9e4d-86b5-4943-aef5-68199bf29e8c	19	4520460	4522427	6	1967	2	NA	4	NA	NA	NA
Panc-AdenoCA	05070acd-3ec1-44bb-9c32-26d3a483a72a	1	236325198	236327886	11	2688	NA	1	9	NA	1	NA
Panc-AdenoCA	05070acd-3ec1-44bb-9c32-26d3a483a72a	6	20822608	20823326	7	718	1	2	4	NA	NA	NA
Panc-AdenoCA	05070acd-3ec1-44bb-9c32-26d3a483a72a	6	56436436	56437619	13	1183	1	5	7	NA	NA	NA
Panc-AdenoCA	05070acd-3ec1-44bb-9c32-26d3a483a72a	11	118524488	118524905	14	417	4	1	9	NA	NA	NA
Panc-AdenoCA	05070acd-3ec1-44bb-9c32-26d3a483a72a	18	61179557	61180188	12	631	NA	1	11	NA	NA	NA
Panc-AdenoCA	05070acd-3ec1-44bb-9c32-26d3a483a72a	18	69489532	69491249	9	1717	2	4	3	NA	NA	NA
Prost-AdenoCA	052015bc-8c24-467c-8ff7-9e0dc4ad39fd	6	17166712	17168489	17	1777	NA	12	5	NA	NA	NA
Ovary-AdenoCA	052665d1-ab75-4f40-be5a-b88154c8beed	20	57974934	57982887	16	7953	4	3	9	NA	NA	NA
Lymph-BNHL	05616329-e7ba-4efd-87b1-d79cd0f7af3d	1	203274816	203276041	8	1225	1	1	4	NA	1	1
Lymph-BNHL	05616329-e7ba-4efd-87b1-d79cd0f7af3d	3	60604546	60608516	6	3970	3	NA	2	NA	NA	1
Lymph-BNHL	05616329-e7ba-4efd-87b1-d79cd0f7af3d	3	187460756	187462859	9	2103	NA	4	1	3	NA	1
Lymph-BNHL	05616329-e7ba-4efd-87b1-d79cd0f7af3d	7	53530231	53532254	7	2023	NA	NA	NA	4	1	2
Lymph-BNHL	05616329-e7ba-4efd-87b1-d79cd0f7af3d	8	94595118	94595428	8	310	1	NA	1	4	1	1
Lymph-BNHL	05616329-e7ba-4efd-87b1-d79cd0f7af3d	14	106325228	106356363	74	31135	3	17	23	18	7	6
Lymph-BNHL	05616329-e7ba-4efd-87b1-d79cd0f7af3d	14	106829678	106830504	10	826	1	2	4	NA	2	1
Lymph-BNHL	05616329-e7ba-4efd-87b1-d79cd0f7af3d	18	4674052	4675396	6	1344	NA	NA	1	2	1	2
Lymph-BNHL	05616329-e7ba-4efd-87b1-d79cd0f7af3d	22	23227993	23235606	18	7613	NA	2	4	2	6	4
Lymph-BNHL	05616329-e7ba-4efd-87b1-d79cd0f7af3d	X	123605894	123606364	6	470	NA	NA	NA	3	1	2
Liver-HCC	062e96d4-c623-11e3-bf01-24c6515278c0	1	47959748	47962873	12	3125	2	3	7	NA	NA	NA
Liver-HCC	062e96d4-c623-11e3-bf01-24c6515278c0	7	146021802	146026651	6	4849	2	1	1	2	NA	NA
Lymph-BNHL	068f4f69-d2fe-4f25-912e-ca7d4623efb6	2	89156057	89161049	114	4992	7	11	27	37	15	17
Lymph-BNHL	068f4f69-d2fe-4f25-912e-ca7d4623efb6	3	187462327	187463059	12	732	NA	3	2	3	3	1
Lymph-BNHL	068f4f69-d2fe-4f25-912e-ca7d4623efb6	4	35346478	35350504	16	4026	NA	NA	NA	10	4	2
Lymph-BNHL	068f4f69-d2fe-4f25-912e-ca7d4623efb6	7	104769904	104771442	6	1538	NA	4	2	NA	NA	NA
Lymph-BNHL	068f4f69-d2fe-4f25-912e-ca7d4623efb6	14	106208624	106213556	6	4932	NA	1	4	1	NA	NA
Lymph-BNHL	068f4f69-d2fe-4f25-912e-ca7d4623efb6	14	106238741	106240410	7	1669	1	1	4	1	NA	NA
Lymph-BNHL	068f4f69-d2fe-4f25-912e-ca7d4623efb6	14	106322259	106330122	83	7863	4	9	25	21	12	12
Lymph-BNHL	068f4f69-d2fe-4f25-912e-ca7d4623efb6	14	106586780	106591076	9	4296	NA	1	1	3	1	3
Lymph-BNHL	068f4f69-d2fe-4f25-912e-ca7d4623efb6	14	106994079	106994796	9	717	NA	1	2	5	1	NA
Lymph-BNHL	068f4f69-d2fe-4f25-912e-ca7d4623efb6	18	42363592	42367367	6	3775	NA	NA	NA	2	3	1
Lymph-BNHL	068f4f69-d2fe-4f25-912e-ca7d4623efb6	18	60984530	60986968	20	2438	NA	3	8	4	2	3
Lymph-BNHL	068f4f69-d2fe-4f25-912e-ca7d4623efb6	22	23223104	23231951	20	8847	1	2	12	2	1	2
Cervix-SCC	06dad93a-4b67-49b3-8fa5-f2546e22bae7	1	16937261	16941618	6	4357	NA	5	1	NA	NA	NA
Cervix-SCC	06dad93a-4b67-49b3-8fa5-f2546e22bae7	1	63880162	63883194	11	3032	NA	7	4	NA	NA	NA
Cervix-SCC	06dad93a-4b67-49b3-8fa5-f2546e22bae7	2	201324105	201326984	8	2879	NA	5	3	NA	NA	NA
Cervix-SCC	06dad93a-4b67-49b3-8fa5-f2546e22bae7	3	22398451	22405818	9	7367	2	2	5	NA	NA	NA
Cervix-SCC	06dad93a-4b67-49b3-8fa5-f2546e22bae7	15	41558266	41561296	14	3030	1	4	9	NA	NA	NA
Cervix-SCC	06dad93a-4b67-49b3-8fa5-f2546e22bae7	16	131001	132530	10	1529	NA	3	6	NA	NA	1
Cervix-SCC	06dad93a-4b67-49b3-8fa5-f2546e22bae7	16	1748420	1761170	34	12750	6	10	17	NA	1	NA
Cervix-SCC	06dad93a-4b67-49b3-8fa5-f2546e22bae7	16	15704411	15707502	8	3091	NA	4	4	NA	NA	NA
Cervix-SCC	06dad93a-4b67-49b3-8fa5-f2546e22bae7	20	40250451	40252386	10	1935	1	6	3	NA	NA	NA
Cervix-SCC	06dad93a-4b67-49b3-8fa5-f2546e22bae7	21	23621784	23626495	8	4711	NA	4	4	NA	NA	NA
Cervix-SCC	06dad93a-4b67-49b3-8fa5-f2546e22bae7	21	26732279	26738856	13	6577	2	5	6	NA	NA	NA
Cervix-SCC	06dad93a-4b67-49b3-8fa5-f2546e22bae7	X	17806071	17811622	8	5551	NA	3	5	NA	NA	NA
Prost-AdenoCA	07531318-87e8-4db8-aa61-9b93597d063b	5	98258784	98261060	7	2276	NA	4	3	NA	NA	NA
Prost-AdenoCA	07531318-87e8-4db8-aa61-9b93597d063b	5	101743354	101746395	10	3041	NA	4	4	1	1	NA

Bone-Leiomyo	075fc96d-6742-4ef3-9369-482592ad3a2f	3	979749	980891	7	1142	NA	5	2	NA	NA	NA
Bone-Leiomyo	075fc96d-6742-4ef3-9369-482592ad3a2f	3	18086167	18088654	8	2487	NA	NA	NA	1	6	1
Bone-Leiomyo	075fc96d-6742-4ef3-9369-482592ad3a2f	4	149819204	149824504	10	5300	1	7	2	NA	NA	NA
Bone-Leiomyo	075fc96d-6742-4ef3-9369-482592ad3a2f	5	2447581	2452617	21	5036	NA	9	12	NA	NA	NA
Bone-Leiomyo	075fc96d-6742-4ef3-9369-482592ad3a2f	6	30749346	30749657	6	311	1	4	1	NA	NA	NA
Bone-Leiomyo	075fc96d-6742-4ef3-9369-482592ad3a2f	6	119462708	119471810	13	9102	1	7	5	NA	NA	NA
Bone-Leiomyo	075fc96d-6742-4ef3-9369-482592ad3a2f	6	119514250	119514953	11	703	NA	NA	11	NA	NA	NA
Bone-Leiomyo	075fc96d-6742-4ef3-9369-482592ad3a2f	7	132552266	132554044	7	1778	1	4	1	NA	1	NA
Bone-Leiomyo	075fc96d-6742-4ef3-9369-482592ad3a2f	7	132899324	132901528	16	2204	2	1	13	NA	NA	NA
Bone-Leiomyo	075fc96d-6742-4ef3-9369-482592ad3a2f	7	132935486	132938050	10	2564	1	1	8	NA	NA	NA
Bone-Leiomyo	075fc96d-6742-4ef3-9369-482592ad3a2f	7	132949281	132953548	7	4267	NA	NA	7	NA	NA	NA
Bone-Leiomyo	075fc96d-6742-4ef3-9369-482592ad3a2f	8	43159267	43160047	8	780	NA	5	3	NA	NA	NA
Bone-Leiomyo	075fc96d-6742-4ef3-9369-482592ad3a2f	9	115041344	115042439	6	1095	1	4	1	NA	NA	NA
Bone-Leiomyo	075fc96d-6742-4ef3-9369-482592ad3a2f	9	127387765	127393565	9	5800	1	7	1	NA	NA	NA
Bone-Leiomyo	075fc96d-6742-4ef3-9369-482592ad3a2f	9	132150387	132154585	10	4198	NA	2	8	NA	NA	NA
Bone-Leiomyo	075fc96d-6742-4ef3-9369-482592ad3a2f	12	49869530	49870216	15	686	NA	NA	15	NA	NA	NA
Bone-Leiomyo	075fc96d-6742-4ef3-9369-482592ad3a2f	12	49942364	49949615	33	7251	NA	NA	33	NA	NA	NA
Bone-Leiomyo	075fc96d-6742-4ef3-9369-482592ad3a2f	12	49991930	49992740	12	810	NA	NA	12	NA	NA	NA
Bone-Leiomyo	075fc96d-6742-4ef3-9369-482592ad3a2f	12	50042925	50046948	8	4023	1	4	3	NA	NA	NA
Bone-Leiomyo	075fc96d-6742-4ef3-9369-482592ad3a2f	12	58158510	58163784	16	5274	NA	2	14	NA	NA	NA
Bone-Leiomyo	075fc96d-6742-4ef3-9369-482592ad3a2f	12	69226699	69231851	9	5152	NA	2	7	NA	NA	NA
Bone-Leiomyo	075fc96d-6742-4ef3-9369-482592ad3a2f	12	72399709	72408659	14	8950	2	5	7	NA	NA	NA
Bone-Leiomyo	075fc96d-6742-4ef3-9369-482592ad3a2f	12	73179607	73185784	11	6177	1	9	1	NA	NA	NA
Bone-Leiomyo	075fc96d-6742-4ef3-9369-482592ad3a2f	12	73328014	73333169	18	5155	6	5	7	NA	NA	NA
Bone-Leiomyo	075fc96d-6742-4ef3-9369-482592ad3a2f	12	73399272	73404813	18	5541	4	1	13	NA	NA	NA
Bone-Leiomyo	075fc96d-6742-4ef3-9369-482592ad3a2f	12	79354797	79355496	7	699	NA	4	3	NA	NA	NA
Bone-Leiomyo	075fc96d-6742-4ef3-9369-482592ad3a2f	12	82137482	82138132	8	650	NA	NA	8	NA	NA	NA
Bone-Leiomyo	075fc96d-6742-4ef3-9369-482592ad3a2f	12	85197920	85200246	13	2326	1	5	7	NA	NA	NA
Bone-Leiomyo	075fc96d-6742-4ef3-9369-482592ad3a2f	12	85309173	85313654	9	4481	1	1	7	NA	NA	NA
Bone-Leiomyo	075fc96d-6742-4ef3-9369-482592ad3a2f	12	85604994	85607371	8	2377	2	1	5	NA	NA	NA
Bone-Leiomyo	075fc96d-6742-4ef3-9369-482592ad3a2f	12	87066396	87068294	16	1898	1	5	10	NA	NA	NA
Bone-Leiomyo	075fc96d-6742-4ef3-9369-482592ad3a2f	12	87179279	87180156	7	877	NA	NA	NA	NA	6	1
Bone-Leiomyo	075fc96d-6742-4ef3-9369-482592ad3a2f	12	96637863	96643803	7	5940	NA	1	6	NA	NA	NA
Bone-Leiomyo	075fc96d-6742-4ef3-9369-482592ad3a2f	12	96707981	96708613	7	632	3	3	1	NA	NA	NA
Bone-Leiomyo	075fc96d-6742-4ef3-9369-482592ad3a2f	12	102419810	102421537	10	1727	3	4	3	NA	NA	NA
Bone-Leiomyo	075fc96d-6742-4ef3-9369-482592ad3a2f	12	102458953	102464795	16	5842	NA	7	6	NA	3	NA
Bone-Leiomyo	075fc96d-6742-4ef3-9369-482592ad3a2f	12	102523022	102524371	11	1349	4	3	4	NA	NA	NA
Bone-Leiomyo	075fc96d-6742-4ef3-9369-482592ad3a2f	12	105689225	105689706	8	481	1	2	5	NA	NA	NA
Bone-Leiomyo	075fc96d-6742-4ef3-9369-482592ad3a2f	12	105981604	105983018	19	1414	3	11	5	NA	NA	NA
Bone-Leiomyo	075fc96d-6742-4ef3-9369-482592ad3a2f	12	106003163	106007003	11	3840	NA	2	9	NA	NA	NA
Bone-Leiomyo	075fc96d-6742-4ef3-9369-482592ad3a2f	12	119609386	119609936	11	550	1	4	6	NA	NA	NA
Bone-Leiomyo	075fc96d-6742-4ef3-9369-482592ad3a2f	12	121746944	121747544	9	600	NA	NA	9	NA	NA	NA
Bone-Leiomyo	075fc96d-6742-4ef3-9369-482592ad3a2f	20	49874009	49878735	8	4726	1	3	4	NA	NA	NA
Lymph-BNHL	07835447-5d7e-4828-80fd-89b063989c9a	1	206285343	206289108	7	3765	1	2	4	NA	NA	NA
Lymph-BNHL	07835447-5d7e-4828-80fd-89b063989c9a	2	89157306	89160092	57	2786	3	7	17	16	6	8
Lymph-BNHL	07835447-5d7e-4828-80fd-89b063989c9a	2	89544438	89544855	14	417	NA	2	4	3	3	2
Lymph-BNHL	07835447-5d7e-4828-80fd-89b063989c9a	6	37138279	37140655	15	2376	2	2	11	NA	NA	NA
Lymph-BNHL	07835447-5d7e-4828-80fd-89b063989c9a	8	40428539	40429800	7	1261	NA	2	NA	2	3	NA
Lymph-BNHL	07835447-5d7e-4828-80fd-89b063989c9a	9	37423147	37426137	10	2990	1	2	7	NA	NA	NA
Lymph-BNHL	07835447-5d7e-4828-80fd-89b063989c9a	11	90594505	90599223	8	4718	1	1	1	NA	3	2
Lymph-BNHL	07835447-5d7e-4828-80fd-89b063989c9a	12	92538742	92540541	9	1799	NA	1	8	NA	NA	NA
Lymph-BNHL	07835447-5d7e-4828-80fd-89b063989c9a	14	106110395	106148102	45	37707	6	11	27	NA	NA	1
Lymph-BNHL	07835447-5d7e-4828-80fd-89b063989c9a	14	106173417	106178587	12	5170	NA	2	8	NA	NA	2
Lymph-BNHL	07835447-5d7e-4828-80fd-89b063989c9a	14	106208869	106350166	258	1E+05	35	58	##	19	10	6
Lymph-BNHL	07835447-5d7e-4828-80fd-89b063989c9a	14	106518691	106518829	8	138	1	2	2	2	NA	1
Lymph-BNHL	07835447-5d7e-4828-80fd-89b063989c9a	22	23223100	23235372	61	12272	2	11	29	15	2	2
Panc-AdenoCA	079bcb09-a075-4313-9a3e-dd67734a8c5d	3	58936230	58940158	7	3928	1	4	2	NA	NA	NA
Panc-AdenoCA	079bcb09-a075-4313-9a3e-dd67734a8c5d	10	54161249	54162905	7	1656	2	2	3	NA	NA	NA
Panc-AdenoCA	079bcb09-a075-4313-9a3e-dd67734a8c5d	20	22684696	22686077	14	1381	2	7	5	NA	NA	NA
Cervix-SCC	07d20658-3db4-47e7-877b-66536266edfc	1	57868262	57872707	9	4445	1	5	3	NA	NA	NA

Cervix-SCC	07d20658-3db4-47e7-877b-66536266edfc	1	187535245	187536236	7	991	3	4	NA	NA	NA	NA
Cervix-SCC	07d20658-3db4-47e7-877b-66536266edfc	7	3323093	3324567	10	1474	2	4	3	NA	NA	1
Cervix-SCC	07d20658-3db4-47e7-877b-66536266edfc	7	14014991	14016295	7	1304	1	1	5	NA	NA	NA
Cervix-SCC	07d20658-3db4-47e7-877b-66536266edfc	12	34007088	34010259	10	3171	1	6	3	NA	NA	NA
Cervix-SCC	07d20658-3db4-47e7-877b-66536266edfc	15	67859412	67861675	28	2263	5	11	12	NA	NA	NA
Kidney-ChRCC	07e0e938-f61f-477a-92a2-5b7e74b78943	3	188391464	188393675	6	2211	1	NA	3	1	1	NA
Prost-AdenoCA	07f16397-71bb-4594-ad4d-caa7d2baeabd	4	171214661	171215204	9	543	NA	NA	NA	3	5	1
Prost-AdenoCA	07f16397-71bb-4594-ad4d-caa7d2baeabd	5	2399325	2400377	7	1052	NA	3	4	NA	NA	NA
Prost-AdenoCA	07f16397-71bb-4594-ad4d-caa7d2baeabd	5	3049216	3052284	7	3068	NA	4	3	NA	NA	NA
ColoRect-AdenoCA	080ecc31-756a-4a1b-a51e-d632ac8219f7	2	82332612	82336631	12	4019	4	3	4	NA	1	NA
ColoRect-AdenoCA	080ecc31-756a-4a1b-a51e-d632ac8219f7	20	33047423	33047954	7	531	NA	6	1	NA	NA	NA
Head-SCC	08227616-02a5-46e8-9db1-f2d1d691ab23	3	152038969	152042343	17	3374	2	1	14	NA	NA	NA
Head-SCC	08227616-02a5-46e8-9db1-f2d1d691ab23	14	23838726	23839603	9	877	3	2	4	NA	NA	NA
Head-SCC	08227616-02a5-46e8-9db1-f2d1d691ab23	X	57167725	57168282	10	557	1	7	2	NA	NA	NA
CNS-GBM	08ac57ec-0036-4134-a9bb-f22eaa27ab0d	12	38178420	38178591	6	171	NA	3	3	NA	NA	NA
Eso-AdenoCa	09497b9b-6fca-48cb-af97-161a3e434a51	3	171730258	171730983	8	725	2	6	NA	NA	NA	NA
Eso-AdenoCa	09497b9b-6fca-48cb-af97-161a3e434a51	8	34653842	34654742	7	900	NA	5	2	NA	NA	NA
Eso-AdenoCa	09497b9b-6fca-48cb-af97-161a3e434a51	12	21722142	21726331	9	4189	4	3	1	NA	NA	1
Eso-AdenoCa	09497b9b-6fca-48cb-af97-161a3e434a51	12	43471331	43476984	13	5653	3	1	9	NA	NA	NA
Ovary-AdenoCA	09508a0d-ebe0-4fa1-b7b2-1710814181cd	19	30780642	30781680	9	1038	NA	8	1	NA	NA	NA
Panc-AdenoCA	0972bfcf-c6c6-48cc-b820-cdfa6279a4f3	1	113708815	113711424	7	2609	1	NA	6	NA	NA	NA
Panc-AdenoCA	0972bfcf-c6c6-48cc-b820-cdfa6279a4f3	3	26956743	26958619	6	1876	2	1	3	NA	NA	NA
Panc-AdenoCA	0972bfcf-c6c6-48cc-b820-cdfa6279a4f3	3	169849715	169850214	11	499	4	6	1	NA	NA	NA
Panc-AdenoCA	0972bfcf-c6c6-48cc-b820-cdfa6279a4f3	6	121350232	121351545	6	1313	2	1	2	NA	NA	1
Panc-AdenoCA	0972bfcf-c6c6-48cc-b820-cdfa6279a4f3	7	31340657	31340915	7	258	NA	NA	7	NA	NA	NA
Panc-AdenoCA	0972bfcf-c6c6-48cc-b820-cdfa6279a4f3	7	35819295	35819409	7	114	3	NA	4	NA	NA	NA
Panc-AdenoCA	0972bfcf-c6c6-48cc-b820-cdfa6279a4f3	7	43851155	43851508	7	353	NA	NA	7	NA	NA	NA
Panc-AdenoCA	0972bfcf-c6c6-48cc-b820-cdfa6279a4f3	7	69954359	69954821	7	462	NA	1	6	NA	NA	NA
Panc-AdenoCA	0972bfcf-c6c6-48cc-b820-cdfa6279a4f3	11	46767606	46768199	7	593	NA	2	5	NA	NA	NA
Panc-AdenoCA	0972bfcf-c6c6-48cc-b820-cdfa6279a4f3	11	82194661	82198545	17	3884	NA	7	9	1	NA	NA
Panc-AdenoCA	0972bfcf-c6c6-48cc-b820-cdfa6279a4f3	11	123113562	123115283	7	1721	NA	3	4	NA	NA	NA
Panc-AdenoCA	0972bfcf-c6c6-48cc-b820-cdfa6279a4f3	18	11336449	11344305	11	7856	2	3	6	NA	NA	NA
Panc-AdenoCA	0972bfcf-c6c6-48cc-b820-cdfa6279a4f3	18	19958417	19960614	9	2197	3	4	2	NA	NA	NA
Panc-AdenoCA	0972bfcf-c6c6-48cc-b820-cdfa6279a4f3	X	125986599	125987358	8	759	NA	NA	NA	4	2	2
Lung-AdenoCA	09bc8542-4ea1-4599-91b3-d606425b37e2	1	24806623	24808449	6	1826	NA	2	4	NA	NA	NA
Lung-AdenoCA	09bc8542-4ea1-4599-91b3-d606425b37e2	1	222024966	222027113	7	2147	3	4	NA	NA	NA	NA
Lung-AdenoCA	09bc8542-4ea1-4599-91b3-d606425b37e2	2	66809185	66813073	24	3888	5	15	4	NA	NA	NA
Lung-AdenoCA	09bc8542-4ea1-4599-91b3-d606425b37e2	3	122565655	122575671	15	10016	5	5	5	NA	NA	NA
Lung-AdenoCA	09bc8542-4ea1-4599-91b3-d606425b37e2	3	151066543	151067784	8	1241	4	2	2	NA	NA	NA
Lung-AdenoCA	09bc8542-4ea1-4599-91b3-d606425b37e2	4	15736592	15738890	10	2298	NA	8	2	NA	NA	NA
Lung-AdenoCA	09bc8542-4ea1-4599-91b3-d606425b37e2	8	4789723	4790543	9	820	NA	6	3	NA	NA	NA
Lung-AdenoCA	09bc8542-4ea1-4599-91b3-d606425b37e2	12	66453558	66457042	16	3484	1	10	5	NA	NA	NA
Lung-AdenoCA	09bc8542-4ea1-4599-91b3-d606425b37e2	16	79821475	79826083	15	4608	3	8	4	NA	NA	NA
Panc-AdenoCA	09cb8bc5-13ac-44ac-9b7d-6de143373570	3	144600673	144603712	6	3039	NA	NA	NA	2	3	1
Panc-AdenoCA	09cb8bc5-13ac-44ac-9b7d-6de143373570	18	22365904	22369200	12	3296	3	6	3	NA	NA	NA
Panc-AdenoCA	09cb8bc5-13ac-44ac-9b7d-6de143373570	18	22465656	22467360	7	1704	1	6	NA	NA	NA	NA
Panc-AdenoCA	09cb8bc5-13ac-44ac-9b7d-6de143373570	18	22634768	22639977	8	5209	1	3	4	NA	NA	NA
Panc-AdenoCA	09cb8bc5-13ac-44ac-9b7d-6de143373570	18	23118645	23124378	8	5733	NA	3	5	NA	NA	NA
Panc-AdenoCA	09cb8bc5-13ac-44ac-9b7d-6de143373570	18	24207055	24210389	7	3334	3	3	1	NA	NA	NA
Panc-AdenoCA	09e1fe3e-bfd8-4175-ac42-0e1bf0ba5523	3	26005375	26006556	6	1181	NA	3	3	NA	NA	NA
Panc-AdenoCA	09e1fe3e-bfd8-4175-ac42-0e1bf0ba5523	4	2610371	2614373	15	4002	NA	6	7	1	NA	1
Panc-AdenoCA	09e1fe3e-bfd8-4175-ac42-0e1bf0ba5523	9	31719478	31721769	7	2291	NA	4	3	NA	NA	NA
Panc-AdenoCA	09e1fe3e-bfd8-4175-ac42-0e1bf0ba5523	11	97415207	97416350	9	1143	NA	1	8	NA	NA	NA
Panc-AdenoCA	09e1fe3e-bfd8-4175-ac42-0e1bf0ba5523	11	122131437	122136644	21	5207	NA	10	11	NA	NA	NA
Prost-AdenoCA	0a6be23a-d5a0-4e95-ada2-a61b2b5d9485	8	26176112	26185078	16	8966	NA	11	5	NA	NA	NA
Panc-AdenoCA	0b29c893-03bf-4131-b192-c14a2788d411	19	53780055	53780067	6	12	NA	2	1	2	NA	1
Panc-AdenoCA	0b29c893-03bf-4131-b192-c14a2788d411	X	142189623	142190918	9	1295	2	1	6	NA	NA	NA
Lymph-CLL	0b6cd7df-6970-4d60-b7b5-85002a7d8781	13	89363885	89368662	10	4777	NA	NA	1	3	4	2
Lymph-CLL	0b6cd7df-6970-4d60-b7b5-85002a7d8781	14	106325382	106372225	53	46843	2	11	13	15	7	5
Lymph-CLL	0b6cd7df-6970-4d60-b7b5-85002a7d8781	14	107169530	107179032	15	9502	NA	3	4	4	1	3

Lymph-CLL	0b6cd7df-6970-4d60-b7b5-85002a7d8781	22	23055021	23055595	19	574	4	3	6	2	3	1
Lymph-CLL	0b6cd7df-6970-4d60-b7b5-85002a7d8781	22	23101421	23102254	15	833	3	5	3	2	NA	2
Prost-AdenoCA	0bfd1043-5142-3662-e050-11ac0c486501	7	113961488	113963382	8	1894	1	1	6	NA	NA	NA
Prost-AdenoCA	0bfd1043-5142-3662-e050-11ac0c486501	7	114050280	114052288	7	2008	NA	NA	7	NA	NA	NA
Prost-AdenoCA	0bfd1043-5142-3662-e050-11ac0c486501	8	56634262	56638657	20	4395	2	9	9	NA	NA	NA
Prost-AdenoCA	0bfd1043-5142-3662-e050-11ac0c486501	8	56694956	56695349	7	393	1	2	4	NA	NA	NA
Prost-AdenoCA	0bfd1043-5142-3662-e050-11ac0c486501	8	124281863	124284377	17	2514	3	7	7	NA	NA	NA
Prost-AdenoCA	0bfd1043-5142-3662-e050-11ac0c486501	8	127391614	127392595	10	981	1	2	7	NA	NA	NA
Prost-AdenoCA	0bfd1043-5142-3662-e050-11ac0c486501	8	128391806	128392721	7	915	2	5	NA	NA	NA	NA
Prost-AdenoCA	0bfd1043-5142-3662-e050-11ac0c486501	10	34496955	34498368	17	1413	NA	11	6	NA	NA	NA
Prost-AdenoCA	0bfd1043-5142-3662-e050-11ac0c486501	19	59097579	59098153	8	574	1	6	1	NA	NA	NA
Prost-AdenoCA	0bfd1043-70fb-d2dc-e050-11ac0c4860cb	7	113961488	113963382	8	1894	1	1	6	NA	NA	NA
Prost-AdenoCA	0bfd1043-70fb-d2dc-e050-11ac0c4860cb	7	114050280	114052288	7	2008	NA	NA	7	NA	NA	NA
Prost-AdenoCA	0bfd1043-70fb-d2dc-e050-11ac0c4860cb	10	34496955	34498368	17	1413	NA	11	6	NA	NA	NA
Prost-AdenoCA	0bfd1043-7343-fdd0-e050-11ac0c484cab	5	43503216	43504063	11	847	4	5	2	NA	NA	NA
Prost-AdenoCA	0bfd1043-7343-fdd0-e050-11ac0c484cab	5	74451444	74458515	20	7071	3	13	4	NA	NA	NA
Prost-AdenoCA	0bfd1043-7343-fdd0-e050-11ac0c484cab	5	165722404	165730376	12	7972	NA	8	4	NA	NA	NA
Prost-AdenoCA	0bfd1043-7343-fdd0-e050-11ac0c484cab	6	26673526	26676102	10	2576	NA	6	4	NA	NA	NA
Prost-AdenoCA	0bfd1043-7343-fdd0-e050-11ac0c484cab	6	26799400	26800079	8	679	1	5	2	NA	NA	NA
Prost-AdenoCA	0bfd1043-7343-fdd0-e050-11ac0c484cab	6	52632975	52634630	9	1655	NA	5	4	NA	NA	NA
Prost-AdenoCA	0bfd1043-7343-fdd0-e050-11ac0c484cab	6	142658336	142661263	9	2927	NA	5	4	NA	NA	NA
Prost-AdenoCA	0bfd1043-7343-fdd0-e050-11ac0c484cab	8	105179323	105180959	10	1636	NA	1	9	NA	NA	NA
Prost-AdenoCA	0bfd1043-7343-fdd0-e050-11ac0c484cab	8	107817289	107818977	9	1688	3	3	3	NA	NA	NA
Prost-AdenoCA	0bfd1043-7343-fdd0-e050-11ac0c484cab	11	25654530	25659793	20	5263	2	12	6	NA	NA	NA
Prost-AdenoCA	0bfd1043-7343-fdd0-e050-11ac0c484cab	12	2406503	2407682	8	1179	NA	3	5	NA	NA	NA
Prost-AdenoCA	0bfd1043-7343-fdd0-e050-11ac0c484cab	12	25280904	25287088	14	6184	NA	7	7	NA	NA	NA
Prost-AdenoCA	0bfd1043-7343-fdd0-e050-11ac0c484cab	20	33664883	33665434	9	551	1	2	6	NA	NA	NA
Prost-AdenoCA	0bfd1043-7343-fdd0-e050-11ac0c484cab	20	33687815	33691649	14	3834	1	7	6	NA	NA	NA
Prost-AdenoCA	0bfd1043-7343-fdd0-e050-11ac0c484cab	20	33702859	33711838	36	8979	2	17	17	NA	NA	NA
Prost-AdenoCA	0bfd1043-7343-fdd0-e050-11ac0c484cab	X	63734126	63734496	8	370	3	2	3	NA	NA	NA
Prost-AdenoCA	0bfd1043-7344-fdd0-e050-11ac0c484cab	4	170945689	170946925	6	1236	2	1	3	NA	NA	NA
Prost-AdenoCA	0bfd1043-7344-fdd0-e050-11ac0c484cab	15	78761484	78763011	6	1527	1	4	1	NA	NA	NA
Prost-AdenoCA	0bfd1043-7344-fdd0-e050-11ac0c484cab	X	44998797	45002395	7	3598	1	4	2	NA	NA	NA
Prost-AdenoCA	0bfd1043-7ebf-aaec-e050-11ac0c482f39	1	219182955	219186381	7	3426	NA	4	3	NA	NA	NA
Prost-AdenoCA	0bfd1043-7ebf-aaec-e050-11ac0c482f39	2	132370918	132372756	11	1838	1	5	5	NA	NA	NA
Prost-AdenoCA	0bfd1043-7ebf-aaec-e050-11ac0c482f39	2	132986596	132990709	8	4113	2	6	NA	NA	NA	NA
Prost-AdenoCA	0bfd1043-7ebf-aaec-e050-11ac0c482f39	2	138045866	138050977	7	5111	NA	3	4	NA	NA	NA
Prost-AdenoCA	0bfd1043-7ebf-aaec-e050-11ac0c482f39	2	138057584	138064457	9	6873	1	5	3	NA	NA	NA
Prost-AdenoCA	0bfd1043-7ebf-aaec-e050-11ac0c482f39	2	138489812	138490949	9	1137	NA	5	4	NA	NA	NA
Prost-AdenoCA	0bfd1043-7ebf-aaec-e050-11ac0c482f39	4	108530561	108531085	11	524	NA	NA	11	NA	NA	NA
Prost-AdenoCA	0bfd1043-7ebf-aaec-e050-11ac0c482f39	6	118517440	118526547	18	9107	NA	14	4	NA	NA	NA
Prost-AdenoCA	0bfd1043-7ebf-aaec-e050-11ac0c482f39	8	39915996	39917101	8	1105	1	2	5	NA	NA	NA
Prost-AdenoCA	0bfd1043-7ebf-aaec-e050-11ac0c482f39	8	39953854	39963547	16	9693	1	7	8	NA	NA	NA
Prost-AdenoCA	0bfd1043-7ebf-aaec-e050-11ac0c482f39	10	55166349	55167516	18	1167	NA	4	14	NA	NA	NA
Prost-AdenoCA	0bfd1043-7ebf-aaec-e050-11ac0c482f39	17	25558277	25563151	7	4874	1	1	4	1	NA	NA
Prost-AdenoCA	0bfd1043-7ebf-aaec-e050-11ac0c482f39	X	72829542	72832540	6	2998	1	2	3	NA	NA	NA
Prost-AdenoCA	0bfd1043-7ebf-aaec-e050-11ac0c482f39	X	73024862	73025076	7	214	NA	2	5	NA	NA	NA
Prost-AdenoCA	0bfd1043-7ec1-aaec-e050-11ac0c482f39	2	163184265	163187340	6	3075	1	2	3	NA	NA	NA
Prost-AdenoCA	0bfd1043-7ec1-aaec-e050-11ac0c482f39	2	180992013	180992486	7	473	NA	2	5	NA	NA	NA
Prost-AdenoCA	0bfd1043-7ec1-aaec-e050-11ac0c482f39	2	191475730	191477101	14	1371	7	5	1	NA	NA	1
Prost-AdenoCA	0bfd1043-7ec1-aaec-e050-11ac0c482f39	4	138279515	138279776	6	261	2	3	1	NA	NA	NA
Prost-AdenoCA	0bfd1043-7ec1-aaec-e050-11ac0c482f39	6	140575899	140577888	14	1989	2	2	10	NA	NA	NA
Prost-AdenoCA	0bfd1043-7ec1-aaec-e050-11ac0c482f39	8	75215998	75216332	6	334	1	3	2	NA	NA	NA
Prost-AdenoCA	0bfd1043-816e-e3e4-e050-11ac0c4860c5	4	170945689	170946925	6	1236	2	1	3	NA	NA	NA
Prost-AdenoCA	0bfd1043-816e-e3e4-e050-11ac0c4860c5	15	78761484	78763225	7	1741	1	4	1	NA	1	NA
Prost-AdenoCA	0bfd1043-816e-e3e4-e050-11ac0c4860c5	X	44998797	45002395	7	3598	1	4	2	NA	NA	NA
Prost-AdenoCA	0bfd1043-8170-e3e4-e050-11ac0c4860c5	1	174416077	174423563	18	7486	3	6	8	NA	1	NA
Prost-AdenoCA	0bfd1043-8170-e3e4-e050-11ac0c4860c5	4	52873723	52879886	15	6163	1	8	5	NA	1	NA
Prost-AdenoCA	0bfd1043-8170-e3e4-e050-11ac0c4860c5	5	6224913	6225255	6	342	NA	1	5	NA	NA	NA
Prost-AdenoCA	0bfd1043-8170-e3e4-e050-11ac0c4860c5	5	9057545	9059103	11	1558	1	7	3	NA	NA	NA

Prost-AdenoCA	0bfd1043-8170-e3e4-e050-11ac0c4860c5	5	9148181	9150712	9	2531	NA	7	2	NA	NA	NA
Prost-AdenoCA	0bfd1043-8170-e3e4-e050-11ac0c4860c5	5	14703507	14705063	8	1556	NA	7	1	NA	NA	NA
Prost-AdenoCA	0bfd1043-8170-e3e4-e050-11ac0c4860c5	5	50372307	50374349	10	2042	1	6	3	NA	NA	NA
Prost-AdenoCA	0bfd1043-8170-e3e4-e050-11ac0c4860c5	7	132635300	132636140	6	840	NA	4	2	NA	NA	NA
Prost-AdenoCA	0bfd1043-8170-e3e4-e050-11ac0c4860c5	9	88785861	88788780	13	2919	4	7	2	NA	NA	NA
Prost-AdenoCA	0bfd1043-8170-e3e4-e050-11ac0c4860c5	18	4656427	4656788	9	361	1	6	2	NA	NA	NA
Prost-AdenoCA	0bfd1043-8172-e3e4-e050-11ac0c4860c5	4	52873723	52879886	15	6163	1	8	5	NA	1	NA
Prost-AdenoCA	0bfd1043-8172-e3e4-e050-11ac0c4860c5	5	6224913	6225255	6	342	NA	1	5	NA	NA	NA
Prost-AdenoCA	0bfd1043-8172-e3e4-e050-11ac0c4860c5	5	9057545	9059103	11	1558	1	7	3	NA	NA	NA
Prost-AdenoCA	0bfd1043-8172-e3e4-e050-11ac0c4860c5	5	9148181	9150712	9	2531	NA	7	2	NA	NA	NA
Prost-AdenoCA	0bfd1043-8172-e3e4-e050-11ac0c4860c5	5	14703507	14705063	8	1556	NA	7	1	NA	NA	NA
Prost-AdenoCA	0bfd1043-8172-e3e4-e050-11ac0c4860c5	18	4656427	4656788	9	361	1	6	2	NA	NA	NA
Prost-AdenoCA	0bfd1043-8173-e3e4-e050-11ac0c4860c5	4	52873723	52879886	15	6163	1	8	5	NA	1	NA
Prost-AdenoCA	0bfd1043-8173-e3e4-e050-11ac0c4860c5	5	6224913	6225255	6	342	NA	1	5	NA	NA	NA
Prost-AdenoCA	0bfd1043-8173-e3e4-e050-11ac0c4860c5	5	9057545	9059103	11	1558	1	7	3	NA	NA	NA
Prost-AdenoCA	0bfd1043-8173-e3e4-e050-11ac0c4860c5	5	9148181	9150712	9	2531	NA	7	2	NA	NA	NA
Prost-AdenoCA	0bfd1043-8173-e3e4-e050-11ac0c4860c5	5	14703507	14705063	8	1556	NA	7	1	NA	NA	NA
Prost-AdenoCA	0bfd1043-8173-e3e4-e050-11ac0c4860c5	18	4656427	4656788	9	361	1	6	2	NA	NA	NA
Prost-AdenoCA	0bfd1043-8175-e3e4-e050-11ac0c4860c5	4	52873723	52879886	15	6163	1	8	5	NA	1	NA
Prost-AdenoCA	0bfd1043-8175-e3e4-e050-11ac0c4860c5	5	6224913	6228330	7	3417	1	1	5	NA	NA	NA
Prost-AdenoCA	0bfd1043-8175-e3e4-e050-11ac0c4860c5	5	9057545	9059103	11	1558	1	7	3	NA	NA	NA
Prost-AdenoCA	0bfd1043-8175-e3e4-e050-11ac0c4860c5	5	9148181	9150712	9	2531	NA	7	2	NA	NA	NA
Prost-AdenoCA	0bfd1043-8175-e3e4-e050-11ac0c4860c5	5	14703507	14705063	8	1556	NA	7	1	NA	NA	NA
Prost-AdenoCA	0bfd1043-8175-e3e4-e050-11ac0c4860c5	10	38562796	38565246	8	2450	1	2	4	NA	NA	1
Prost-AdenoCA	0bfd1043-8175-e3e4-e050-11ac0c4860c5	18	4656427	4656788	9	361	1	6	2	NA	NA	NA
Prost-AdenoCA	0bfd1043-8177-e3e4-e050-11ac0c4860c5	5	168670368	168671207	6	839	NA	4	2	NA	NA	NA
Prost-AdenoCA	0bfd1043-8177-e3e4-e050-11ac0c4860c5	7	1981655	1984863	8	3208	2	1	4	NA	1	NA
Prost-AdenoCA	0bfd1043-8177-e3e4-e050-11ac0c4860c5	7	2045259	2046493	14	1234	2	9	3	NA	NA	NA
Prost-AdenoCA	0bfd1043-8177-e3e4-e050-11ac0c4860c5	7	4407976	4414903	16	6927	1	9	5	NA	1	NA
Prost-AdenoCA	0bfd1043-8177-e3e4-e050-11ac0c4860c5	7	156212796	156213727	7	931	NA	5	2	NA	NA	NA
Prost-AdenoCA	0bfd1043-817c-e3e4-e050-11ac0c4860c5	2	199337264	199337792	6	528	1	NA	5	NA	NA	NA
Prost-AdenoCA	0bfd1043-817c-e3e4-e050-11ac0c4860c5	6	140575899	140577888	14	1989	2	2	10	NA	NA	NA
Prost-AdenoCA	0bfd1043-817c-e3e4-e050-11ac0c4860c5	8	75215998	75216332	6	334	1	3	2	NA	NA	NA
Prost-AdenoCA	0bfd1043-817e-e3e4-e050-11ac0c4860c5	2	209243596	209244201	11	605	2	6	3	NA	NA	NA
Prost-AdenoCA	0bfd1043-817e-e3e4-e050-11ac0c4860c5	10	76702210	76707043	13	4833	1	8	4	NA	NA	NA
Prost-AdenoCA	0bfd1043-8180-e3e4-e050-11ac0c4860c5	1	61307445	61310046	11	2601	NA	5	6	NA	NA	NA
Prost-AdenoCA	0bfd1043-8180-e3e4-e050-11ac0c4860c5	1	63565371	63566341	10	970	1	4	5	NA	NA	NA
Prost-AdenoCA	0bfd1043-8180-e3e4-e050-11ac0c4860c5	3	142796540	142800809	6	4269	NA	2	3	NA	NA	1
Prost-AdenoCA	0bfd1043-8180-e3e4-e050-11ac0c4860c5	13	35770662	35772925	11	2263	NA	4	7	NA	NA	NA
Prost-AdenoCA	0bfd1043-8180-e3e4-e050-11ac0c4860c5	22	28343756	28344512	7	756	NA	1	NA	2	2	2
Prost-AdenoCA	0bfd1043-8181-e3e4-e050-11ac0c4860c5	1	61307445	61310046	11	2601	NA	5	6	NA	NA	NA
Prost-AdenoCA	0bfd1043-8181-e3e4-e050-11ac0c4860c5	1	63565371	63566341	10	970	1	4	5	NA	NA	NA
Prost-AdenoCA	0bfd1043-8181-e3e4-e050-11ac0c4860c5	13	35770662	35772925	11	2263	NA	4	7	NA	NA	NA
Prost-AdenoCA	0bfd1043-8181-e3e4-e050-11ac0c4860c5	22	28343756	28344512	7	756	NA	1	NA	2	2	2
Prost-AdenoCA	0bfd1043-8181-e3e4-e050-11ac0c4860c5	X	69416693	69423267	12	6574	NA	12	NA	NA	NA	NA
Prost-AdenoCA	0bfd1043-8183-e3e4-e050-11ac0c4860c5	2	209243596	209244201	11	605	2	6	3	NA	NA	NA
Prost-AdenoCA	0bfd1043-8183-e3e4-e050-11ac0c4860c5	4	178721787	178721794	6	7	3	1	2	NA	NA	NA
Prost-AdenoCA	0bfd1043-8183-e3e4-e050-11ac0c4860c5	10	63252801	63252813	8	12	1	2	NA	2	1	2
Prost-AdenoCA	0bfd1043-8183-e3e4-e050-11ac0c4860c5	10	76702210	76711837	14	9627	1	8	5	NA	NA	NA
Prost-AdenoCA	0bfd1043-8187-e3e4-e050-11ac0c4860c5	1	46529669	46532702	6	3033	NA	4	2	NA	NA	NA
Prost-AdenoCA	0bfd1043-8187-e3e4-e050-11ac0c4860c5	4	103213423	103222706	20	9283	2	9	7	NA	1	1
Prost-AdenoCA	0bfd1043-8189-e3e4-e050-11ac0c4860c5	7	113961488	113963382	8	1894	1	1	6	NA	NA	NA
Prost-AdenoCA	0bfd1043-8189-e3e4-e050-11ac0c4860c5	7	114050280	114052288	7	2008	NA	NA	7	NA	NA	NA
Prost-AdenoCA	0bfd1043-8189-e3e4-e050-11ac0c4860c5	8	56634262	56638657	20	4395	2	9	9	NA	NA	NA
Prost-AdenoCA	0bfd1043-8189-e3e4-e050-11ac0c4860c5	8	56694956	56695349	7	393	1	2	4	NA	NA	NA
Prost-AdenoCA	0bfd1043-8189-e3e4-e050-11ac0c4860c5	8	124281863	124284377	17	2514	3	7	7	NA	NA	NA
Prost-AdenoCA	0bfd1043-8189-e3e4-e050-11ac0c4860c5	8	128391118	128392721	7	1603	1	6	NA	NA	NA	NA
Prost-AdenoCA	0bfd1043-8189-e3e4-e050-11ac0c4860c5	10	34496955	34498368	17	1413	NA	11	6	NA	NA	NA
Prost-AdenoCA	0bfd1043-8189-e3e4-e050-11ac0c4860c5	19	59097579	59098153	7	574	1	5	1	NA	NA	NA
Prost-AdenoCA	0bfd1068-3fc3-a95b-e050-11ac0c4860c3	4	52873723	52879886	15	6163	1	8	5	NA	1	NA

Prost-AdenoCA	0bfd1068-3fc3-a95b-e050-11ac0c4860c3	5	6224913	6225255	6	342	NA	1	5	NA	NA	NA
Prost-AdenoCA	0bfd1068-3fc3-a95b-e050-11ac0c4860c3	5	9057545	9059103	11	1558	1	7	3	NA	NA	NA
Prost-AdenoCA	0bfd1068-3fc3-a95b-e050-11ac0c4860c3	5	9148181	9150712	8	2531	NA	6	2	NA	NA	NA
Prost-AdenoCA	0bfd1068-3fc3-a95b-e050-11ac0c4860c3	5	14703507	14705063	8	1556	NA	7	1	NA	NA	NA
Prost-AdenoCA	0bfd1068-3fc3-a95b-e050-11ac0c4860c3	18	4656427	4656788	9	361	1	6	2	NA	NA	NA
Prost-AdenoCA	0bfd1068-3fca-a95b-e050-11ac0c4860c3	5	168670368	168671207	6	839	NA	4	2	NA	NA	NA
Prost-AdenoCA	0bfd1068-3fcd-a95b-e050-11ac0c4860c3	1	155099419	155099745	8	326	1	5	2	NA	NA	NA
Prost-AdenoCA	0bfd1068-3fcd-a95b-e050-11ac0c4860c3	8	40203415	40208671	7	5256	1	5	1	NA	NA	NA
Prost-AdenoCA	0bfd1068-3fcd-a95b-e050-11ac0c4860c3	X	77200568	77202399	8	1831	NA	5	3	NA	NA	NA
Prost-AdenoCA	0bfd1068-3fcf-a95b-e050-11ac0c4860c3	2	180992013	180992486	7	473	NA	2	5	NA	NA	NA
Prost-AdenoCA	0bfd1068-3fcf-a95b-e050-11ac0c4860c3	2	191475730	191477101	14	1371	7	5	1	NA	NA	1
Prost-AdenoCA	0bfd1068-3fcf-a95b-e050-11ac0c4860c3	6	140575899	140577888	14	1989	2	2	10	NA	NA	NA
Prost-AdenoCA	0bfd1068-3fcf-a95b-e050-11ac0c4860c3	8	75215998	75216332	6	334	1	3	2	NA	NA	NA
Prost-AdenoCA	0bfd1068-3fcf-a95b-e050-11ac0c4860c3	X	65802544	65804555	6	2011	1	4	1	NA	NA	NA
Prost-AdenoCA	0bfd1068-3fd3-a95b-e050-11ac0c4860c3	1	117418082	117419550	10	1468	1	5	4	NA	NA	NA
Prost-AdenoCA	0bfd1068-3fd3-a95b-e050-11ac0c4860c3	1	117734181	117735463	11	1282	1	5	4	NA	1	NA
Prost-AdenoCA	0bfd1068-3fd3-a95b-e050-11ac0c4860c3	1	242070563	242073859	13	3296	1	7	5	NA	NA	NA
Prost-AdenoCA	0bfd1068-3fd3-a95b-e050-11ac0c4860c3	2	193587397	193593986	8	6589	NA	7	1	NA	NA	NA
Prost-AdenoCA	0bfd1068-3fd3-a95b-e050-11ac0c4860c3	2	199909346	199912318	8	2972	1	5	2	NA	NA	NA
Prost-AdenoCA	0bfd1068-3fd3-a95b-e050-11ac0c4860c3	2	233145457	233157583	15	12126	3	5	7	NA	NA	NA
Prost-AdenoCA	0bfd1068-3fd3-a95b-e050-11ac0c4860c3	5	43503216	43504063	11	847	4	5	2	NA	NA	NA
Prost-AdenoCA	0bfd1068-3fd3-a95b-e050-11ac0c4860c3	5	177108730	177109647	7	917	3	3	1	NA	NA	NA
Prost-AdenoCA	0bfd1068-3fd3-a95b-e050-11ac0c4860c3	6	52632975	52634630	6	1655	NA	3	3	NA	NA	NA
Prost-AdenoCA	0bfd1068-3fd3-a95b-e050-11ac0c4860c3	6	142658336	142661263	9	2927	NA	5	4	NA	NA	NA
Prost-AdenoCA	0bfd1068-3fd3-a95b-e050-11ac0c4860c3	8	27615033	27616309	10	1276	4	4	1	NA	NA	1
Prost-AdenoCA	0bfd1068-3fd3-a95b-e050-11ac0c4860c3	17	21450071	21454362	6	4291	NA	5	NA	NA	1	NA
Prost-AdenoCA	0bfd1068-3fd3-a95b-e050-11ac0c4860c3	17	67704590	67710382	10	5792	1	7	2	NA	NA	NA
Prost-AdenoCA	0bfd1068-3fd3-a95b-e050-11ac0c4860c3	17	67768116	67776676	13	8560	3	9	1	NA	NA	NA
Prost-AdenoCA	0bfd1068-3fd3-a95b-e050-11ac0c4860c3	20	33664883	33665434	9	551	1	2	6	NA	NA	NA
Prost-AdenoCA	0bfd1068-3fd3-a95b-e050-11ac0c4860c3	20	33687815	33691649	14	3834	1	7	6	NA	NA	NA
Prost-AdenoCA	0bfd1068-3fd3-a95b-e050-11ac0c4860c3	20	33702859	33711838	33	8979	1	17	15	NA	NA	NA
Prost-AdenoCA	0bfd1068-3fd3-a95b-e050-11ac0c4860c3	X	65578467	65579855	19	1388	NA	13	6	NA	NA	NA
Prost-AdenoCA	0bfd1068-3fd3-a95b-e050-11ac0c4860c3	X	66304027	66307155	8	3128	1	6	NA	NA	1	NA
Prost-AdenoCA	0bfd1068-3fd3-a95b-e050-11ac0c4860c3	X	67054951	67064935	19	9984	NA	14	4	NA	1	NA
Prost-AdenoCA	0bfd1068-3fd3-a95b-e050-11ac0c4860c3	X	67101196	67106780	11	5584	NA	8	3	NA	NA	NA
Prost-AdenoCA	0bfd1068-3fd3-a95b-e050-11ac0c4860c3	X	67208532	67211855	10	3323	NA	9	NA	NA	NA	1
Prost-AdenoCA	0bfd1068-3fd5-a95b-e050-11ac0c4860c3	2	163184265	163187340	7	3075	2	2	3	NA	NA	NA
Prost-AdenoCA	0bfd1068-3fd5-a95b-e050-11ac0c4860c3	2	180992013	180992486	7	473	NA	2	5	NA	NA	NA
Prost-AdenoCA	0bfd1068-3fd5-a95b-e050-11ac0c4860c3	2	181104799	181112679	14	7880	NA	3	10	NA	NA	1
Prost-AdenoCA	0bfd1068-3fd5-a95b-e050-11ac0c4860c3	2	191475730	191477101	14	1371	7	5	1	NA	NA	1
Prost-AdenoCA	0bfd1068-3fd5-a95b-e050-11ac0c4860c3	4	138279515	138279776	6	261	2	3	1	NA	NA	NA
Prost-AdenoCA	0bfd1068-3fd5-a95b-e050-11ac0c4860c3	6	140575899	140577888	14	1989	2	2	10	NA	NA	NA
Prost-AdenoCA	0bfd1068-3fd5-a95b-e050-11ac0c4860c3	8	75215998	75216332	6	334	1	3	2	NA	NA	NA
Prost-AdenoCA	0bfd1068-3fd8-a95b-e050-11ac0c4860c3	1	219182955	219186381	7	3426	NA	4	3	NA	NA	NA
Prost-AdenoCA	0bfd1068-3fd8-a95b-e050-11ac0c4860c3	2	132370918	132372756	13	1838	1	6	6	NA	NA	NA
Prost-AdenoCA	0bfd1068-3fd8-a95b-e050-11ac0c4860c3	2	132986596	132989033	7	2437	1	6	NA	NA	NA	NA
Prost-AdenoCA	0bfd1068-3fd8-a95b-e050-11ac0c4860c3	2	138045866	138050977	7	5111	NA	3	4	NA	NA	NA
Prost-AdenoCA	0bfd1068-3fd8-a95b-e050-11ac0c4860c3	2	138057584	138064457	9	6873	1	5	3	NA	NA	NA
Prost-AdenoCA	0bfd1068-3fd8-a95b-e050-11ac0c4860c3	2	138489812	138490949	9	1137	NA	5	4	NA	NA	NA
Prost-AdenoCA	0bfd1068-3fd8-a95b-e050-11ac0c4860c3	3	62225392	62231451	12	6059	1	7	4	NA	NA	NA
Prost-AdenoCA	0bfd1068-3fd8-a95b-e050-11ac0c4860c3	4	108530561	108531085	11	524	NA	NA	11	NA	NA	NA
Prost-AdenoCA	0bfd1068-3fd8-a95b-e050-11ac0c4860c3	6	118517440	118526547	18	9107	NA	14	4	NA	NA	NA
Prost-AdenoCA	0bfd1068-3fd8-a95b-e050-11ac0c4860c3	8	39915996	39917101	8	1105	1	2	5	NA	NA	NA
Prost-AdenoCA	0bfd1068-3fd8-a95b-e050-11ac0c4860c3	8	39953854	39963547	14	9693	1	6	7	NA	NA	NA
Prost-AdenoCA	0bfd1068-3fd8-a95b-e050-11ac0c4860c3	10	55166305	55167516	19	1211	NA	5	14	NA	NA	NA
Prost-AdenoCA	0bfd1068-3fd8-a95b-e050-11ac0c4860c3	X	66970033	66970685	6	652	NA	3	3	NA	NA	NA
Prost-AdenoCA	0bfd1068-3fd8-a95b-e050-11ac0c4860c3	X	68747256	68749563	10	2307	2	3	4	NA	1	NA
Prost-AdenoCA	0bfd1068-3fdd-a95b-e050-11ac0c4860c3	1	219182955	219186381	7	3426	NA	4	3	NA	NA	NA
Prost-AdenoCA	0bfd1068-3fdd-a95b-e050-11ac0c4860c3	2	132370918	132372689	12	1771	NA	6	6	NA	NA	NA
Prost-AdenoCA	0bfd1068-3fdd-a95b-e050-11ac0c4860c3	2	132986596	132990709	8	4113	2	6	NA	NA	NA	NA

Prost-AdenoCA	0bfd1068-3fdd-a95b-e050-11ac0c4860c3	2	138045866	138050977	7	5111	NA	3	4	NA	NA	NA
Prost-AdenoCA	0bfd1068-3fdd-a95b-e050-11ac0c4860c3	2	138057584	138064457	9	6873	1	5	3	NA	NA	NA
Prost-AdenoCA	0bfd1068-3fdd-a95b-e050-11ac0c4860c3	2	138489812	138490949	9	1137	NA	5	4	NA	NA	NA
Prost-AdenoCA	0bfd1068-3fdd-a95b-e050-11ac0c4860c3	4	108530561	108531085	11	524	NA	NA	11	NA	NA	NA
Prost-AdenoCA	0bfd1068-3fdd-a95b-e050-11ac0c4860c3	6	118517440	118526547	18	9107	NA	14	4	NA	NA	NA
Prost-AdenoCA	0bfd1068-3fdd-a95b-e050-11ac0c4860c3	8	39915996	39917101	8	1105	1	2	5	NA	NA	NA
Prost-AdenoCA	0bfd1068-3fdd-a95b-e050-11ac0c4860c3	8	39953854	39963547	16	9693	1	7	8	NA	NA	NA
Prost-AdenoCA	0bfd1068-3fdd-a95b-e050-11ac0c4860c3	10	106663893	106667643	11	3750	NA	8	2	NA	1	NA
Prost-AdenoCA	0bfd1068-3fdd-a95b-e050-11ac0c4860c3	X	73024842	73025076	8	234	NA	2	6	NA	NA	NA
Prost-AdenoCA	0bfd1068-3fdf-a95b-e050-11ac0c4860c3	1	61307445	61310046	11	2601	NA	5	6	NA	NA	NA
Prost-AdenoCA	0bfd1068-3fdf-a95b-e050-11ac0c4860c3	1	63565371	63566341	10	970	1	4	5	NA	NA	NA
Prost-AdenoCA	0bfd1068-3fdf-a95b-e050-11ac0c4860c3	3	142796540	142800809	6	4269	NA	2	3	NA	NA	1
Prost-AdenoCA	0bfd1068-3fdf-a95b-e050-11ac0c4860c3	13	35770662	35772925	11	2263	NA	4	7	NA	NA	NA
Prost-AdenoCA	0bfd1068-3fdf-a95b-e050-11ac0c4860c3	22	28343756	28344512	7	756	NA	1	NA	2	2	2
Prost-AdenoCA	0bfd1068-3fe1-a95b-e050-11ac0c4860c3	1	61307445	61310046	11	2601	NA	5	6	NA	NA	NA
Prost-AdenoCA	0bfd1068-3fe1-a95b-e050-11ac0c4860c3	1	63565371	63566341	10	970	1	4	5	NA	NA	NA
Prost-AdenoCA	0bfd1068-3fe1-a95b-e050-11ac0c4860c3	3	142796540	142800809	6	4269	NA	2	3	NA	NA	1
Prost-AdenoCA	0bfd1068-3fe1-a95b-e050-11ac0c4860c3	5	51939118	51941470	11	2352	4	4	3	NA	NA	NA
Prost-AdenoCA	0bfd1068-3fe1-a95b-e050-11ac0c4860c3	13	35770662	35772925	11	2263	NA	4	7	NA	NA	NA
Prost-AdenoCA	0bfd1068-3fe1-a95b-e050-11ac0c4860c3	22	28343756	28344512	7	756	NA	1	NA	2	2	2
Prost-AdenoCA	0bfd1068-3fe4-a95b-e050-11ac0c4860c3	7	113961488	113963382	8	1894	1	1	6	NA	NA	NA
Prost-AdenoCA	0bfd1068-3fe4-a95b-e050-11ac0c4860c3	7	114050280	114052288	7	2008	NA	NA	7	NA	NA	NA
Prost-AdenoCA	0bfd1068-3fe4-a95b-e050-11ac0c4860c3	8	56634262	56638657	17	4395	2	9	6	NA	NA	NA
Prost-AdenoCA	0bfd1068-3fe4-a95b-e050-11ac0c4860c3	8	124281863	124286833	16	4970	4	6	6	NA	NA	NA
Prost-AdenoCA	0bfd1068-3fe4-a95b-e050-11ac0c4860c3	10	34496955	34498368	17	1413	NA	11	6	NA	NA	NA
Prost-AdenoCA	0bfd1068-3fe4-a95b-e050-11ac0c4860c3	19	59097579	59098153	8	574	1	6	1	NA	NA	NA
Prost-AdenoCA	0bfe2ac9-0af3-c248-e050-11ac0d487e1c	2	209243596	209244201	11	605	2	6	3	NA	NA	NA
Prost-AdenoCA	0bfe2ac9-0af3-c248-e050-11ac0d487e1c	4	178721787	178721794	6	7	3	1	2	NA	NA	NA
Prost-AdenoCA	0bfe2ac9-0af3-c248-e050-11ac0d487e1c	10	63252801	63252813	7	12	NA	2	NA	2	1	2
Prost-AdenoCA	0bfe2ac9-0af3-c248-e050-11ac0d487e1c	10	76702210	76707043	13	4833	1	8	4	NA	NA	NA
Prost-AdenoCA	0bfe2ac9-0af5-c248-e050-11ac0d487e1c	1	219182955	219186381	7	3426	NA	4	3	NA	NA	NA
Prost-AdenoCA	0bfe2ac9-0af5-c248-e050-11ac0d487e1c	2	132370918	132372756	13	1838	1	6	6	NA	NA	NA
Prost-AdenoCA	0bfe2ac9-0af5-c248-e050-11ac0d487e1c	2	132986596	132989033	7	2437	1	6	NA	NA	NA	NA
Prost-AdenoCA	0bfe2ac9-0af5-c248-e050-11ac0d487e1c	2	138045866	138050977	7	5111	NA	3	4	NA	NA	NA
Prost-AdenoCA	0bfe2ac9-0af5-c248-e050-11ac0d487e1c	2	138059516	138064457	7	4941	1	4	2	NA	NA	NA
Prost-AdenoCA	0bfe2ac9-0af5-c248-e050-11ac0d487e1c	2	138489812	138490949	9	1137	NA	5	4	NA	NA	NA
Prost-AdenoCA	0bfe2ac9-0af5-c248-e050-11ac0d487e1c	4	108530561	108531085	11	524	NA	NA	11	NA	NA	NA
Prost-AdenoCA	0bfe2ac9-0af5-c248-e050-11ac0d487e1c	6	118517440	118526547	18	9107	NA	14	4	NA	NA	NA
Prost-AdenoCA	0bfe2ac9-0af5-c248-e050-11ac0d487e1c	8	39915996	39917101	8	1105	1	2	5	NA	NA	NA
Prost-AdenoCA	0bfe2ac9-0af5-c248-e050-11ac0d487e1c	8	39953854	39963547	16	9693	1	7	8	NA	NA	NA
Prost-AdenoCA	0bfe2ac9-0af5-c248-e050-11ac0d487e1c	10	55166305	55167516	19	1211	NA	5	14	NA	NA	NA
Prost-AdenoCA	0bfe2ac9-0af5-c248-e050-11ac0d487e1c	X	72829542	72832540	6	2998	1	2	3	NA	NA	NA
Prost-AdenoCA	0bfe2ac9-0af8-c248-e050-11ac0d487e1c	1	219182955	219186381	7	3426	NA	4	3	NA	NA	NA
Prost-AdenoCA	0bfe2ac9-0af8-c248-e050-11ac0d487e1c	2	132370918	132372689	12	1771	NA	6	6	NA	NA	NA
Prost-AdenoCA	0bfe2ac9-0af8-c248-e050-11ac0d487e1c	2	132986596	132990709	8	4113	2	6	NA	NA	NA	NA
Prost-AdenoCA	0bfe2ac9-0af8-c248-e050-11ac0d487e1c	2	138045866	138050977	7	5111	NA	3	4	NA	NA	NA
Prost-AdenoCA	0bfe2ac9-0af8-c248-e050-11ac0d487e1c	2	138059516	138064457	7	4941	NA	4	3	NA	NA	NA
Prost-AdenoCA	0bfe2ac9-0af8-c248-e050-11ac0d487e1c	2	138489812	138490949	9	1137	NA	5	4	NA	NA	NA
Prost-AdenoCA	0bfe2ac9-0af8-c248-e050-11ac0d487e1c	4	108530561	108531085	11	524	NA	NA	11	NA	NA	NA
Prost-AdenoCA	0bfe2ac9-0af8-c248-e050-11ac0d487e1c	6	118517440	118526547	18	9107	NA	14	4	NA	NA	NA
Prost-AdenoCA	0bfe2ac9-0af8-c248-e050-11ac0d487e1c	8	39915996	39917101	8	1105	1	2	5	NA	NA	NA
Prost-AdenoCA	0bfe2ac9-0af8-c248-e050-11ac0d487e1c	8	39953854	39963547	15	9693	1	7	7	NA	NA	NA
Prost-AdenoCA	0bfe2ac9-0af8-c248-e050-11ac0d487e1c	10	55166305	55167516	19	1211	NA	5	14	NA	NA	NA
Prost-AdenoCA	0bfe2ac9-0af8-c248-e050-11ac0d487e1c	X	73024842	73025076	8	234	NA	2	6	NA	NA	NA
Prost-AdenoCA	0bfe2ac9-0afa-c248-e050-11ac0d487e1c	7	113961488	113963382	8	1894	1	1	6	NA	NA	NA
Prost-AdenoCA	0bfe2ac9-0afa-c248-e050-11ac0d487e1c	7	114050280	114052288	7	2008	NA	NA	7	NA	NA	NA
Prost-AdenoCA	0bfe2ac9-0afa-c248-e050-11ac0d487e1c	10	34496955	34498368	17	1413	NA	11	6	NA	NA	NA
Prost-AdenoCA	0bfe2ac9-0afd-c248-e050-11ac0d487e1c	7	113961488	113963382	8	1894	1	1	6	NA	NA	NA
Prost-AdenoCA	0bfe2ac9-0afd-c248-e050-11ac0d487e1c	7	114050280	114052288	7	2008	NA	NA	7	NA	NA	NA
Prost-AdenoCA	0bfe2ac9-0afd-c248-e050-11ac0d487e1c	8	56634262	56638657	20	4395	2	9	9	NA	NA	NA

Prost-AdenoCA	0bfe2ac9-0afd-c248-e050-11ac0d487e1c	8	56694956	56695349	7	393	1	2	4	NA	NA	NA
Prost-AdenoCA	0bfe2ac9-0afd-c248-e050-11ac0d487e1c	8	124281863	124284377	17	2514	3	7	7	NA	NA	NA
Prost-AdenoCA	0bfe2ac9-0afd-c248-e050-11ac0d487e1c	8	128391806	128392721	7	915	2	5	NA	NA	NA	NA
Prost-AdenoCA	0bfe2ac9-0afd-c248-e050-11ac0d487e1c	10	34496955	34498368	17	1413	NA	11	6	NA	NA	NA
Prost-AdenoCA	0bfe2ac9-0afd-c248-e050-11ac0d487e1c	19	59097579	59098153	8	574	1	6	1	NA	NA	NA
Prost-AdenoCA	0bfe2ac9-0aff-c248-e050-11ac0d487e1c	7	113961488	113963382	8	1894	1	1	6	NA	NA	NA
Prost-AdenoCA	0bfe2ac9-0aff-c248-e050-11ac0d487e1c	7	114050280	114052288	7	2008	NA	NA	7	NA	NA	NA
Prost-AdenoCA	0bfe2ac9-0aff-c248-e050-11ac0d487e1c	8	56634262	56638657	20	4395	2	9	9	NA	NA	NA
Prost-AdenoCA	0bfe2ac9-0aff-c248-e050-11ac0d487e1c	8	56694956	56695349	7	393	1	2	4	NA	NA	NA
Prost-AdenoCA	0bfe2ac9-0aff-c248-e050-11ac0d487e1c	8	124281863	124284377	17	2514	3	7	7	NA	NA	NA
Prost-AdenoCA	0bfe2ac9-0aff-c248-e050-11ac0d487e1c	8	128391118	128392721	7	1603	1	6	NA	NA	NA	NA
Prost-AdenoCA	0bfe2ac9-0aff-c248-e050-11ac0d487e1c	10	34496955	34498368	17	1413	NA	11	6	NA	NA	NA
Prost-AdenoCA	0bfe2ac9-0aff-c248-e050-11ac0d487e1c	19	59097579	59098063	6	484	1	4	1	NA	NA	NA
Prost-AdenoCA	0bfebf9f-c779-e57d-e050-11ac0d487827	1	219182955	219186381	7	3426	NA	4	3	NA	NA	NA
Prost-AdenoCA	0bfebf9f-c779-e57d-e050-11ac0d487827	2	132988345	132989033	6	688	1	5	NA	NA	NA	NA
Prost-AdenoCA	0bfebf9f-c779-e57d-e050-11ac0d487827	2	138045866	138050977	7	5111	NA	3	4	NA	NA	NA
Prost-AdenoCA	0bfebf9f-c779-e57d-e050-11ac0d487827	2	138489812	138490949	9	1137	NA	5	4	NA	NA	NA
Prost-AdenoCA	0bfebf9f-c779-e57d-e050-11ac0d487827	4	108530561	108531085	10	524	NA	NA	10	NA	NA	NA
Prost-AdenoCA	0bfebf9f-c779-e57d-e050-11ac0d487827	6	118517440	118526547	18	9107	NA	14	4	NA	NA	NA
Prost-AdenoCA	0bfebf9f-c779-e57d-e050-11ac0d487827	8	39915996	39917101	8	1105	1	2	5	NA	NA	NA
Prost-AdenoCA	0bfebf9f-c779-e57d-e050-11ac0d487827	8	39953854	39954433	14	579	NA	6	8	NA	NA	NA
Prost-AdenoCA	0bfebf9f-c779-e57d-e050-11ac0d487827	10	55166305	55167516	19	1211	NA	5	14	NA	NA	NA
Prost-AdenoCA	0bfebf9f-c77d-e57d-e050-11ac0d487827	1	61307445	61310046	11	2601	NA	5	6	NA	NA	NA
Prost-AdenoCA	0bfebf9f-c77d-e57d-e050-11ac0d487827	1	63565371	63566341	10	970	1	4	5	NA	NA	NA
Prost-AdenoCA	0bfebf9f-c77d-e57d-e050-11ac0d487827	3	142796540	142800809	6	4269	NA	2	3	NA	NA	1
Prost-AdenoCA	0bfebf9f-c77d-e57d-e050-11ac0d487827	13	35770662	35772925	11	2263	NA	4	7	NA	NA	NA
Prost-AdenoCA	0bfebf9f-c77d-e57d-e050-11ac0d487827	22	28343756	28344512	7	756	NA	1	NA	2	2	2
Prost-AdenoCA	0bfebf9f-c77f-e57d-e050-11ac0d487827	1	219182955	219186381	7	3426	NA	4	3	NA	NA	NA
Prost-AdenoCA	0bfebf9f-c77f-e57d-e050-11ac0d487827	2	132370918	132372756	12	1838	1	6	5	NA	NA	NA
Prost-AdenoCA	0bfebf9f-c77f-e57d-e050-11ac0d487827	2	132986596	132990709	8	4113	2	6	NA	NA	NA	NA
Prost-AdenoCA	0bfebf9f-c77f-e57d-e050-11ac0d487827	2	138045866	138050977	7	5111	NA	3	4	NA	NA	NA
Prost-AdenoCA	0bfebf9f-c77f-e57d-e050-11ac0d487827	2	138057584	138064457	9	6873	1	5	3	NA	NA	NA
Prost-AdenoCA	0bfebf9f-c77f-e57d-e050-11ac0d487827	2	138489812	138490949	9	1137	NA	5	4	NA	NA	NA
Prost-AdenoCA	0bfebf9f-c77f-e57d-e050-11ac0d487827	4	108530561	108531085	11	524	NA	NA	11	NA	NA	NA
Prost-AdenoCA	0bfebf9f-c77f-e57d-e050-11ac0d487827	6	118517440	118526547	18	9107	NA	14	4	NA	NA	NA
Prost-AdenoCA	0bfebf9f-c77f-e57d-e050-11ac0d487827	8	35480058	35483588	8	3530	NA	5	3	NA	NA	NA
Prost-AdenoCA	0bfebf9f-c77f-e57d-e050-11ac0d487827	8	35503855	35507549	11	3694	1	3	7	NA	NA	NA
Prost-AdenoCA	0bfebf9f-c77f-e57d-e050-11ac0d487827	8	36813475	36836329	28	22854	1	14	12	NA	1	NA
Prost-AdenoCA	0bfebf9f-c77f-e57d-e050-11ac0d487827	8	39915996	39917101	8	1105	1	2	5	NA	NA	NA
Prost-AdenoCA	0bfebf9f-c77f-e57d-e050-11ac0d487827	8	39953854	39954612	15	758	NA	7	8	NA	NA	NA
Prost-AdenoCA	0bfebf9f-c77f-e57d-e050-11ac0d487827	8	84531463	84540666	15	9203	NA	8	7	NA	NA	NA
Prost-AdenoCA	0bfebf9f-c77f-e57d-e050-11ac0d487827	8	85380962	85384918	9	3956	1	4	4	NA	NA	NA
Prost-AdenoCA	0bfebf9f-c77f-e57d-e050-11ac0d487827	10	55166305	55167516	19	1211	NA	5	14	NA	NA	NA
Prost-AdenoCA	0bfebf9f-c781-e57d-e050-11ac0d487827	7	113961488	113963382	8	1894	1	1	6	NA	NA	NA
Prost-AdenoCA	0bfebf9f-c781-e57d-e050-11ac0d487827	7	114050280	114052288	7	2008	NA	NA	7	NA	NA	NA
Prost-AdenoCA	0bfebf9f-c781-e57d-e050-11ac0d487827	8	56634262	56638657	20	4395	2	9	9	NA	NA	NA
Prost-AdenoCA	0bfebf9f-c781-e57d-e050-11ac0d487827	8	56694956	56695349	7	393	1	2	4	NA	NA	NA
Prost-AdenoCA	0bfebf9f-c781-e57d-e050-11ac0d487827	8	124281863	124284377	17	2514	3	7	7	NA	NA	NA
Prost-AdenoCA	0bfebf9f-c781-e57d-e050-11ac0d487827	8	127391614	127392595	10	981	1	2	7	NA	NA	NA
Prost-AdenoCA	0bfebf9f-c781-e57d-e050-11ac0d487827	10	34496955	34498368	17	1413	NA	11	6	NA	NA	NA
Prost-AdenoCA	0bfebf9f-c781-e57d-e050-11ac0d487827	19	59097579	59098153	8	574	1	6	1	NA	NA	NA
Prost-AdenoCA	0bfebf9f-c783-e57d-e050-11ac0d487827	7	113961488	113963382	8	1894	1	1	6	NA	NA	NA
Prost-AdenoCA	0bfebf9f-c783-e57d-e050-11ac0d487827	7	114050280	114052288	7	2008	NA	NA	7	NA	NA	NA
Prost-AdenoCA	0bfebf9f-c783-e57d-e050-11ac0d487827	8	56634262	56638657	20	4395	2	9	9	NA	NA	NA
Prost-AdenoCA	0bfebf9f-c783-e57d-e050-11ac0d487827	8	56694956	56695349	7	393	1	2	4	NA	NA	NA
Prost-AdenoCA	0bfebf9f-c783-e57d-e050-11ac0d487827	8	124281863	124286833	18	4970	4	7	7	NA	NA	NA
Prost-AdenoCA	0bfebf9f-c783-e57d-e050-11ac0d487827	10	34496955	34498368	17	1413	NA	11	6	NA	NA	NA
Prost-AdenoCA	0bfebf9f-c783-e57d-e050-11ac0d487827	19	59097579	59098153	8	574	1	6	1	NA	NA	NA
Prost-AdenoCA	0c0038ff-6cc4-b0b0-e050-11ac0d483d73	1	219182955	219186381	7	3426	NA	4	3	NA	NA	NA
Prost-AdenoCA	0c0038ff-6cc4-b0b0-e050-11ac0d483d73	2	132370918	132374883	14	3965	1	6	6	NA	NA	1

Prost-AdenoCA	0c0038ff-6cc4-b0b0-e050-11ac0d483d73	2	132986596	132989033	7	2437	1	6	NA	NA	NA	NA
Prost-AdenoCA	0c0038ff-6cc4-b0b0-e050-11ac0d483d73	2	138045866	138050977	7	5111	NA	3	4	NA	NA	NA
Prost-AdenoCA	0c0038ff-6cc4-b0b0-e050-11ac0d483d73	2	138059516	138064457	7	4941	NA	4	3	NA	NA	NA
Prost-AdenoCA	0c0038ff-6cc4-b0b0-e050-11ac0d483d73	2	138489812	138490949	9	1137	NA	5	4	NA	NA	NA
Prost-AdenoCA	0c0038ff-6cc4-b0b0-e050-11ac0d483d73	4	108530561	108531085	11	524	NA	NA	11	NA	NA	NA
Prost-AdenoCA	0c0038ff-6cc4-b0b0-e050-11ac0d483d73	6	118517440	118526547	18	9107	NA	14	4	NA	NA	NA
Prost-AdenoCA	0c0038ff-6cc4-b0b0-e050-11ac0d483d73	8	39915996	39917101	8	1105	1	2	5	NA	NA	NA
Prost-AdenoCA	0c0038ff-6cc4-b0b0-e050-11ac0d483d73	8	39953854	39963547	15	9693	1	7	7	NA	NA	NA
Prost-AdenoCA	0c0038ff-6cc4-b0b0-e050-11ac0d483d73	10	55166305	55167516	19	1211	NA	5	14	NA	NA	NA
Prost-AdenoCA	0c0038ff-6cc4-b0b0-e050-11ac0d483d73	X	72829542	72832540	6	2998	1	2	3	NA	NA	NA
Prost-AdenoCA	0c0038ff-6cc4-b0b0-e050-11ac0d483d73	X	73024842	73025076	8	234	NA	2	6	NA	NA	NA
Prost-AdenoCA	0c0038ff-6cc6-b0b0-e050-11ac0d483d73	1	219182955	219186381	7	3426	NA	4	3	NA	NA	NA
Prost-AdenoCA	0c0038ff-6cc6-b0b0-e050-11ac0d483d73	2	132370918	132372756	13	1838	1	6	6	NA	NA	NA
Prost-AdenoCA	0c0038ff-6cc6-b0b0-e050-11ac0d483d73	2	132986596	132990709	8	4113	2	6	NA	NA	NA	NA
Prost-AdenoCA	0c0038ff-6cc6-b0b0-e050-11ac0d483d73	2	138045866	138050977	7	5111	NA	3	4	NA	NA	NA
Prost-AdenoCA	0c0038ff-6cc6-b0b0-e050-11ac0d483d73	2	138057584	138064457	9	6873	1	5	3	NA	NA	NA
Prost-AdenoCA	0c0038ff-6cc6-b0b0-e050-11ac0d483d73	2	138489812	138490949	9	1137	NA	5	4	NA	NA	NA
Prost-AdenoCA	0c0038ff-6cc6-b0b0-e050-11ac0d483d73	4	108530561	108531085	11	524	NA	NA	11	NA	NA	NA
Prost-AdenoCA	0c0038ff-6cc6-b0b0-e050-11ac0d483d73	6	118517440	118526547	17	9107	NA	13	4	NA	NA	NA
Prost-AdenoCA	0c0038ff-6cc6-b0b0-e050-11ac0d483d73	8	39915996	39917101	8	1105	1	2	5	NA	NA	NA
Prost-AdenoCA	0c0038ff-6cc6-b0b0-e050-11ac0d483d73	8	39953854	39963547	14	9693	1	5	8	NA	NA	NA
Prost-AdenoCA	0c0038ff-6cc6-b0b0-e050-11ac0d483d73	10	55166305	55167516	19	1211	NA	5	14	NA	NA	NA
Prost-AdenoCA	0c0038ff-6cc6-b0b0-e050-11ac0d483d73	X	72829542	72832540	6	2998	1	2	3	NA	NA	NA
Prost-AdenoCA	0c0038ff-6cc6-b0b0-e050-11ac0d483d73	X	73024842	73025076	8	234	NA	2	6	NA	NA	NA
Prost-AdenoCA	0c0038ff-6cc8-b0b0-e050-11ac0d483d73	7	113961488	113963382	8	1894	1	1	6	NA	NA	NA
Prost-AdenoCA	0c0038ff-6cc8-b0b0-e050-11ac0d483d73	7	114050280	114052288	7	2008	NA	NA	7	NA	NA	NA
Prost-AdenoCA	0c0038ff-6cc8-b0b0-e050-11ac0d483d73	8	56634262	56638657	20	4395	2	9	9	NA	NA	NA
Prost-AdenoCA	0c0038ff-6cc8-b0b0-e050-11ac0d483d73	8	56694956	56695349	7	393	1	2	4	NA	NA	NA
Prost-AdenoCA	0c0038ff-6cc8-b0b0-e050-11ac0d483d73	8	124281863	124286833	18	4970	4	7	7	NA	NA	NA
Prost-AdenoCA	0c0038ff-6cc8-b0b0-e050-11ac0d483d73	10	34496955	34498368	17	1413	NA	11	6	NA	NA	NA
Prost-AdenoCA	0c0038ff-6cc8-b0b0-e050-11ac0d483d73	19	59097579	59098153	8	574	1	6	1	NA	NA	NA
Bladder-TCC	0c7aca3f-e006-4de3-afc2-20b4f727d4fd	1	16648839	16651089	6	2250	1	3	1	1	NA	NA
Bladder-TCC	0c7aca3f-e006-4de3-afc2-20b4f727d4fd	1	178763849	178766013	8	2164	NA	2	6	NA	NA	NA
Bladder-TCC	0c7aca3f-e006-4de3-afc2-20b4f727d4fd	2	112917212	112920033	6	2821	1	2	3	NA	NA	NA
Bladder-TCC	0c7aca3f-e006-4de3-afc2-20b4f727d4fd	3	3202801	3207062	11	4261	1	3	7	NA	NA	NA
Bladder-TCC	0c7aca3f-e006-4de3-afc2-20b4f727d4fd	3	26951379	26952907	7	1528	NA	1	6	NA	NA	NA
Bladder-TCC	0c7aca3f-e006-4de3-afc2-20b4f727d4fd	3	187206686	187210432	9	3746	2	2	5	NA	NA	NA
Bladder-TCC	0c7aca3f-e006-4de3-afc2-20b4f727d4fd	6	47239033	47244651	7	5618	3	NA	4	NA	NA	NA
Bladder-TCC	0c7aca3f-e006-4de3-afc2-20b4f727d4fd	7	128618249	128622469	9	4220	1	NA	8	NA	NA	NA
Bladder-TCC	0c7aca3f-e006-4de3-afc2-20b4f727d4fd	9	100270264	100271853	8	1589	NA	3	5	NA	NA	NA
Bladder-TCC	0c7aca3f-e006-4de3-afc2-20b4f727d4fd	10	65178946	65181938	7	2992	1	4	2	NA	NA	NA
Bladder-TCC	0c7aca3f-e006-4de3-afc2-20b4f727d4fd	10	101986228	101991056	11	4828	1	9	1	NA	NA	NA
Bladder-TCC	0c7aca3f-e006-4de3-afc2-20b4f727d4fd	11	65920792	65923365	9	2573	NA	5	3	NA	NA	1
Bladder-TCC	0c7aca3f-e006-4de3-afc2-20b4f727d4fd	11	71103642	71106531	19	2889	NA	6	12	1	NA	NA
Bladder-TCC	0c7aca3f-e006-4de3-afc2-20b4f727d4fd	12	46764010	46765071	6	1061	NA	6	NA	NA	NA	NA
Bladder-TCC	0c7aca3f-e006-4de3-afc2-20b4f727d4fd	13	31326918	31330656	11	3738	3	3	5	NA	NA	NA
Bladder-TCC	0c7aca3f-e006-4de3-afc2-20b4f727d4fd	15	90906775	90910099	6	3324	NA	5	1	NA	NA	NA
Bladder-TCC	0c7aca3f-e006-4de3-afc2-20b4f727d4fd	16	1052634	1055158	6	2524	1	2	3	NA	NA	NA
Bladder-TCC	0c7aca3f-e006-4de3-afc2-20b4f727d4fd	16	85619102	85621599	8	2497	2	3	3	NA	NA	NA
Bladder-TCC	0c7aca3f-e006-4de3-afc2-20b4f727d4fd	18	52946123	52948914	13	2791	2	8	3	NA	NA	NA
Bladder-TCC	0c7aca3f-e006-4de3-afc2-20b4f727d4fd	18	59379242	59381646	7	2404	1	3	2	NA	NA	1
Bladder-TCC	0c7aca3f-e006-4de3-afc2-20b4f727d4fd	19	19617561	19622695	10	5134	1	NA	9	NA	NA	NA
Bladder-TCC	0c7aca3f-e006-4de3-afc2-20b4f727d4fd	20	12949102	12951104	6	2002	1	2	3	NA	NA	NA
Bladder-TCC	0c7aca3f-e006-4de3-afc2-20b4f727d4fd	21	32579158	32582672	6	3514	1	1	4	NA	NA	NA
Bladder-TCC	0c7aca3f-e006-4de3-afc2-20b4f727d4fd	22	18434413	18438631	6	4218	1	3	2	NA	NA	NA
Bladder-TCC	0c7aca3f-e006-4de3-afc2-20b4f727d4fd	X	75959127	75963280	6	4153	NA	2	4	NA	NA	NA
Stomach-AdenoCA	0cd60b96-eb2d-4687-9709-d1455ec45de7	8	11694501	11695640	12	1139	1	6	5	NA	NA	NA
Liver-HCC	0cdbabf2-c623-11e3-bf01-24c6515278c0	6	136357456	136362068	17	4612	2	12	3	NA	NA	NA
Liver-HCC	0cdbabf2-c623-11e3-bf01-24c6515278c0	8	41893303	41895146	7	1843	NA	3	4	NA	NA	NA
Panc-AdenoCA	0cf9bbc2-cbd5-4b64-8d90-cfa416307b39	6	62763065	62763663	6	598	NA	4	2	NA	NA	NA

Panc-AdenoCA	0cf9bbc2-cbd5-4b64-8d90-cfa416307b39	8	96506693	96507676	8	983	NA	6	2	NA	NA	NA
Panc-AdenoCA	0cf9bbc2-cbd5-4b64-8d90-cfa416307b39	19	22379099	22379760	7	661	1	5	1	NA	NA	NA
Panc-AdenoCA	0cf9bbc2-cbd5-4b64-8d90-cfa416307b39	19	32409334	32409525	8	191	NA	3	5	NA	NA	NA
Liver-HCC	0d259ac2-03d1-4814-9b0b-d05e3a6029b7	3	107503487	107505309	6	1822	1	4	1	NA	NA	NA
Liver-HCC	0d259ac2-03d1-4814-9b0b-d05e3a6029b7	5	40310838	40314144	6	3306	1	5	NA	NA	NA	NA
Liver-HCC	0d259ac2-03d1-4814-9b0b-d05e3a6029b7	8	40898211	40898786	6	575	NA	3	3	NA	NA	NA
Lymph-BNHL	0d569bd5-418d-4d72-87ca-8e14668c2119	1	2449065	2453446	6	4381	1	NA	2	1	NA	2
Lymph-BNHL	0d569bd5-418d-4d72-87ca-8e14668c2119	1	2467547	2480394	16	12847	3	2	4	2	3	2
Lymph-BNHL	0d569bd5-418d-4d72-87ca-8e14668c2119	1	188300293	188305466	7	5173	NA	3	NA	2	2	NA
Lymph-BNHL	0d569bd5-418d-4d72-87ca-8e14668c2119	2	5262970	5267621	6	4651	NA	NA	NA	1	3	2
Lymph-BNHL	0d569bd5-418d-4d72-87ca-8e14668c2119	2	89157326	89158953	48	1627	2	7	6	13	12	8
Lymph-BNHL	0d569bd5-418d-4d72-87ca-8e14668c2119	2	176480744	176486936	9	6192	1	3	NA	1	2	2
Lymph-BNHL	0d569bd5-418d-4d72-87ca-8e14668c2119	3	25702005	25703685	6	1680	NA	NA	NA	5	NA	1
Lymph-BNHL	0d569bd5-418d-4d72-87ca-8e14668c2119	3	41228028	41231155	8	3127	NA	NA	2	NA	4	2
Lymph-BNHL	0d569bd5-418d-4d72-87ca-8e14668c2119	3	60463330	60468638	7	5308	2	NA	NA	3	1	1
Lymph-BNHL	0d569bd5-418d-4d72-87ca-8e14668c2119	3	60884163	60888844	7	4681	NA	NA	1	1	2	3
Lymph-BNHL	0d569bd5-418d-4d72-87ca-8e14668c2119	3	186783598	186783821	7	223	1	NA	NA	1	2	3
Lymph-BNHL	0d569bd5-418d-4d72-87ca-8e14668c2119	3	187461546	187464117	9	2571	2	NA	NA	4	2	1
Lymph-BNHL	0d569bd5-418d-4d72-87ca-8e14668c2119	4	34133620	34136966	7	3346	NA	NA	NA	NA	3	4
Lymph-BNHL	0d569bd5-418d-4d72-87ca-8e14668c2119	4	43486828	43491690	7	4862	NA	NA	NA	1	3	3
Lymph-BNHL	0d569bd5-418d-4d72-87ca-8e14668c2119	4	66826996	66839626	16	12630	NA	2	1	9	2	2
Lymph-BNHL	0d569bd5-418d-4d72-87ca-8e14668c2119	4	84727956	84731479	7	3523	NA	NA	NA	3	3	1
Lymph-BNHL	0d569bd5-418d-4d72-87ca-8e14668c2119	4	135486444	135491889	11	5445	NA	2	1	3	2	3
Lymph-BNHL	0d569bd5-418d-4d72-87ca-8e14668c2119	5	4886312	4891096	6	4784	NA	2	NA	3	1	NA
Lymph-BNHL	0d569bd5-418d-4d72-87ca-8e14668c2119	5	4892446	4898169	8	5723	2	1	1	3	NA	1
Lymph-BNHL	0d569bd5-418d-4d72-87ca-8e14668c2119	5	22291717	22297460	9	5743	1	NA	3	1	2	2
Lymph-BNHL	0d569bd5-418d-4d72-87ca-8e14668c2119	6	91004064	91006412	7	2348	NA	2	3	NA	1	1
Lymph-BNHL	0d569bd5-418d-4d72-87ca-8e14668c2119	7	95339560	95344012	6	4452	NA	NA	2	1	1	2
Lymph-BNHL	0d569bd5-418d-4d72-87ca-8e14668c2119	7	109584395	109589824	7	5429	NA	NA	1	3	1	2
Lymph-BNHL	0d569bd5-418d-4d72-87ca-8e14668c2119	7	110637148	110668764	35	31616	3	2	2	12	6	10
Lymph-BNHL	0d569bd5-418d-4d72-87ca-8e14668c2119	7	110676169	110681830	7	5661	NA	1	1	2	NA	3
Lymph-BNHL	0d569bd5-418d-4d72-87ca-8e14668c2119	7	110687136	110693151	8	6015	1	2	NA	4	NA	1
Lymph-BNHL	0d569bd5-418d-4d72-87ca-8e14668c2119	7	110708519	110763581	57	55062	4	4	2	21	9	17
Lymph-BNHL	0d569bd5-418d-4d72-87ca-8e14668c2119	7	110768453	110773878	10	5425	1	2	2	2	NA	3
Lymph-BNHL	0d569bd5-418d-4d72-87ca-8e14668c2119	7	110801384	110821793	25	20409	1	NA	4	7	6	7
Lymph-BNHL	0d569bd5-418d-4d72-87ca-8e14668c2119	7	110832534	110837002	8	4468	NA	NA	1	2	NA	5
Lymph-BNHL	0d569bd5-418d-4d72-87ca-8e14668c2119	7	110935445	110938143	7	2698	NA	NA	NA	3	2	2
Lymph-BNHL	0d569bd5-418d-4d72-87ca-8e14668c2119	7	118252007	118260759	10	8752	NA	1	2	2	2	3
Lymph-BNHL	0d569bd5-418d-4d72-87ca-8e14668c2119	7	132991418	132996745	7	5327	NA	NA	NA	1	3	3
Lymph-BNHL	0d569bd5-418d-4d72-87ca-8e14668c2119	7	133090017	133092615	13	2598	NA	1	NA	7	2	3
Lymph-BNHL	0d569bd5-418d-4d72-87ca-8e14668c2119	8	11352385	11356695	6	4310	1	NA	NA	4	NA	1
Lymph-BNHL	0d569bd5-418d-4d72-87ca-8e14668c2119	8	16652646	16656506	7	3860	1	1	1	2	1	1
Lymph-BNHL	0d569bd5-418d-4d72-87ca-8e14668c2119	8	88402804	88408284	8	5480	NA	2	1	5	NA	NA
Lymph-BNHL	0d569bd5-418d-4d72-87ca-8e14668c2119	8	141866765	141878075	14	11310	NA	NA	NA	6	6	2
Lymph-BNHL	0d569bd5-418d-4d72-87ca-8e14668c2119	9	9161558	9167018	7	5460	NA	NA	NA	3	3	1
Lymph-BNHL	0d569bd5-418d-4d72-87ca-8e14668c2119	9	12310896	12313803	7	2907	2	1	NA	3	NA	1
Lymph-BNHL	0d569bd5-418d-4d72-87ca-8e14668c2119	9	18458834	18466547	9	7713	1	1	NA	2	1	4
Lymph-BNHL	0d569bd5-418d-4d72-87ca-8e14668c2119	9	23926204	23934147	10	7943	1	1	NA	2	3	3
Lymph-BNHL	0d569bd5-418d-4d72-87ca-8e14668c2119	9	23950962	23959164	11	8202	NA	NA	1	3	NA	7
Lymph-BNHL	0d569bd5-418d-4d72-87ca-8e14668c2119	9	28249139	28249709	7	570	NA	NA	NA	4	2	1
Lymph-BNHL	0d569bd5-418d-4d72-87ca-8e14668c2119	9	105642527	105661639	24	19112	1	3	2	10	5	3
Lymph-BNHL	0d569bd5-418d-4d72-87ca-8e14668c2119	10	130842954	130855529	15	12575	1	1	3	5	3	2
Lymph-BNHL	0d569bd5-418d-4d72-87ca-8e14668c2119	11	17145272	17153595	10	8323	NA	1	NA	2	4	3
Lymph-BNHL	0d569bd5-418d-4d72-87ca-8e14668c2119	11	40525600	40538248	23	12648	1	1	3	8	5	5
Lymph-BNHL	0d569bd5-418d-4d72-87ca-8e14668c2119	11	41315479	41318779	11	3300	1	NA	2	3	4	1
Lymph-BNHL	0d569bd5-418d-4d72-87ca-8e14668c2119	12	122458286	122463179	21	4893	1	4	9	4	2	1
Lymph-BNHL	0d569bd5-418d-4d72-87ca-8e14668c2119	13	77664704	77667060	6	2356	NA	NA	1	1	3	1
Lymph-BNHL	0d569bd5-418d-4d72-87ca-8e14668c2119	13	81755261	81763765	13	8504	NA	1	NA	4	4	4
Lymph-BNHL	0d569bd5-418d-4d72-87ca-8e14668c2119	13	94486467	94493700	11	7233	2	NA	NA	3	6	NA
Lymph-BNHL	0d569bd5-418d-4d72-87ca-8e14668c2119	14	31273523	31281437	14	7914	1	2	2	2	4	3

Lymph-BNHL	0d569bd5-418d-4d72-87ca-8e14668c2119	14	58700714	58711048	12	10334	2	2	NA	1	3	4
Lymph-BNHL	0d569bd5-418d-4d72-87ca-8e14668c2119	14	106112658	106114619	16	1961	3	1	12	NA	NA	NA
Lymph-BNHL	0d569bd5-418d-4d72-87ca-8e14668c2119	14	106161269	106165735	8	4466	1	1	1	1	4	NA
Lymph-BNHL	0d569bd5-418d-4d72-87ca-8e14668c2119	14	106210750	106213808	28	3058	2	6	19	NA	NA	1
Lymph-BNHL	0d569bd5-418d-4d72-87ca-8e14668c2119	14	106240210	106240734	9	524	1	1	7	NA	NA	NA
Lymph-BNHL	0d569bd5-418d-4d72-87ca-8e14668c2119	14	106321417	106329280	83	7863	4	12	9	17	23	18
Lymph-BNHL	0d569bd5-418d-4d72-87ca-8e14668c2119	14	106816695	106821145	12	4450	1	1	2	4	2	2
Lymph-BNHL	0d569bd5-418d-4d72-87ca-8e14668c2119	14	107176430	107179030	7	2600	NA	2	1	2	1	1
Lymph-BNHL	0d569bd5-418d-4d72-87ca-8e14668c2119	17	7537920	7542713	7	4793	NA	NA	3	1	NA	3
Lymph-BNHL	0d569bd5-418d-4d72-87ca-8e14668c2119	17	30956909	30959811	9	2902	NA	NA	1	3	3	2
Lymph-BNHL	0d569bd5-418d-4d72-87ca-8e14668c2119	18	60983089	60988652	39	5563	1	8	7	13	5	5
Lymph-BNHL	0d569bd5-418d-4d72-87ca-8e14668c2119	18	71968363	71978715	15	10352	1	2	NA	8	3	1
Lymph-BNHL	0d569bd5-418d-4d72-87ca-8e14668c2119	19	32278547	32282380	6	3833	1	NA	NA	1	2	2
Lymph-BNHL	0d569bd5-418d-4d72-87ca-8e14668c2119	20	10893290	10898531	10	5241	NA	NA	2	2	5	1
Lymph-BNHL	0d569bd5-418d-4d72-87ca-8e14668c2119	20	46229035	46233233	8	4198	NA	NA	3	1	NA	4
Lymph-BNHL	0d569bd5-418d-4d72-87ca-8e14668c2119	21	31923889	31934449	15	10560	2	NA	4	1	2	6
Lymph-BNHL	0d569bd5-418d-4d72-87ca-8e14668c2119	22	22378218	22453249	186	75031	5	31	42	64	19	25
Lymph-BNHL	0d569bd5-418d-4d72-87ca-8e14668c2119	22	23236057	23282642	50	46585	3	12	21	5	6	3
Lymph-BNHL	0d569bd5-418d-4d72-87ca-8e14668c2119	X	9334760	9337785	7	3025	NA	1	3	1	1	1
Lymph-BNHL	0d569bd5-418d-4d72-87ca-8e14668c2119	X	84541826	84547373	8	5547	1	NA	1	3	1	2
Lymph-BNHL	0d569bd5-418d-4d72-87ca-8e14668c2119	X	133376801	133384653	9	7852	NA	1	NA	1	3	4
Lymph-BNHL	0d569bd5-418d-4d72-87ca-8e14668c2119	X	143931182	143935712	7	4530	NA	NA	NA	3	3	1
Stomach-AdenoCA	0e54cea2-d568-4a33-b9db-b698844e6ad9	17	37867750	37869367	15	1617	1	5	9	NA	NA	NA
Ovary-AdenoCA	0ead45d8-d785-4404-8319-2ef951e02e03	11	66446232	66447730	6	1498	NA	1	1	4	NA	NA
Eso-AdenoCa	0ef92ff8-829f-425a-91d8-c594b6e22a2b	1	199269400	199270805	6	1405	NA	NA	NA	4	NA	2
Eso-AdenoCa	0ef92ff8-829f-425a-91d8-c594b6e22a2b	11	104354509	104358297	6	3788	NA	NA	NA	1	2	3
Eso-AdenoCa	0ef92ff8-829f-425a-91d8-c594b6e22a2b	13	71046975	71057552	14	10577	NA	NA	2	1	2	9
Eso-AdenoCa	0ef92ff8-829f-425a-91d8-c594b6e22a2b	13	71061009	71064081	9	3072	NA	NA	NA	3	3	3
Eso-AdenoCa	0ef92ff8-829f-425a-91d8-c594b6e22a2b	X	39911507	39918894	23	7387	4	9	10	NA	NA	NA
Panc-AdenoCA	0efee3f3-313b-44eb-b3b0-af975a1ded82	1	110179072	110179215	6	143	2	NA	4	NA	NA	NA
Panc-AdenoCA	0efee3f3-313b-44eb-b3b0-af975a1ded82	1	111297628	111297854	11	226	NA	NA	11	NA	NA	NA
Panc-AdenoCA	0efee3f3-313b-44eb-b3b0-af975a1ded82	1	238070699	238072367	35	1668	NA	NA	35	NA	NA	NA
Panc-AdenoCA	0efee3f3-313b-44eb-b3b0-af975a1ded82	1	245589429	245593983	16	4554	NA	4	10	NA	NA	2
Kidney-RCC	0f53e757-1823-409f-a0ea-249270728e15	7	7145424	7147397	9	1973	3	2	3	NA	1	NA
Kidney-RCC	0f53e757-1823-409f-a0ea-249270728e15	7	88812972	88817807	17	4835	4	6	3	1	1	2
Kidney-RCC	0f53e757-1823-409f-a0ea-249270728e15	7	98439626	98440838	12	1212	1	7	3	NA	NA	1
Kidney-RCC	0f53e757-1823-409f-a0ea-249270728e15	7	111355998	111359289	20	3291	6	7	6	NA	1	NA
Head-SCC	0f9e70b3-24cf-4d76-af7b-84ace66ec5a7	1	45060640	45065379	16	4739	3	4	7	2	NA	NA
Head-SCC	0f9e70b3-24cf-4d76-af7b-84ace66ec5a7	3	161992358	161994626	12	2268	NA	4	8	NA	NA	NA
Head-SCC	0f9e70b3-24cf-4d76-af7b-84ace66ec5a7	3	162434141	162440275	12	6134	2	4	6	NA	NA	NA
Head-SCC	0f9e70b3-24cf-4d76-af7b-84ace66ec5a7	8	35853286	35853631	8	345	1	1	6	NA	NA	NA
Head-SCC	0f9e70b3-24cf-4d76-af7b-84ace66ec5a7	16	1780489	1782305	10	1816	3	3	4	NA	NA	NA
Head-SCC	0f9e70b3-24cf-4d76-af7b-84ace66ec5a7	16	26538242	26539116	10	874	1	2	7	NA	NA	NA
Head-SCC	0f9e70b3-24cf-4d76-af7b-84ace66ec5a7	17	79269706	79274386	10	4680	2	2	6	NA	NA	NA
Stomach-AdenoCA	0fa9cfba-0f49-47d0-ab54-9dd2144e0a73	2	24734595	24736599	7	2004	2	3	2	NA	NA	NA
Stomach-AdenoCA	0fa9cfba-0f49-47d0-ab54-9dd2144e0a73	4	100961248	100962924	6	1676	NA	4	2	NA	NA	NA
Stomach-AdenoCA	0fa9cfba-0f49-47d0-ab54-9dd2144e0a73	13	100287406	100294370	38	6964	3	20	15	NA	NA	NA
Stomach-AdenoCA	0fa9cfba-0f49-47d0-ab54-9dd2144e0a73	13	108700009	108700464	7	455	NA	NA	7	NA	NA	NA
Stomach-AdenoCA	0fa9cfba-0f49-47d0-ab54-9dd2144e0a73	13	108707066	108707753	14	687	3	4	7	NA	NA	NA
Panc-AdenoCA	0fbd94b1-bb34-4620-841b-861a0b5e0c12	3	6328685	6329091	6	406	NA	NA	1	2	1	2
Panc-AdenoCA	0fbd94b1-bb34-4620-841b-861a0b5e0c12	7	98397577	98398131	7	554	1	NA	6	NA	NA	NA
Panc-AdenoCA	0fbd94b1-bb34-4620-841b-861a0b5e0c12	8	37732021	37735008	12	2987	5	4	3	NA	NA	NA
Panc-AdenoCA	0fbd94b1-bb34-4620-841b-861a0b5e0c12	16	32614030	32620562	9	6532	NA	2	NA	NA	3	4
Panc-AdenoCA	0fc63b79-7fff-441d-88fc-922d53c787ab	7	37328872	37329953	10	1081	1	3	6	NA	NA	NA
Panc-AdenoCA	0fc63b79-7fff-441d-88fc-922d53c787ab	8	92700844	92702647	10	1803	2	4	4	NA	NA	NA
Panc-AdenoCA	0fc63b79-7fff-441d-88fc-922d53c787ab	17	25445093	25446464	8	1371	1	4	3	NA	NA	NA
Uterus-AdenoCA	105a51c4-cc7e-4d0f-9cf8-e4d64a31d14d	2	18924549	18926086	11	1537	3	3	5	NA	NA	NA
Uterus-AdenoCA	105a51c4-cc7e-4d0f-9cf8-e4d64a31d14d	3	172207865	172208401	7	536	NA	3	3	NA	1	NA
ColoRect-AdenoCA	10ad692b-4c3d-42de-9b5e-4968441388b3	4	119941502	119942794	6	1292	2	4	NA	NA	NA	NA
ColoRect-AdenoCA	10ad692b-4c3d-42de-9b5e-4968441388b3	17	79636688	79636705	6	17	1	1	3	NA	1	NA

Liver-HCC	10db2142-c623-11e3-bf01-24c6515278c0	11	115072700	115073174	6	474	2	2	2	NA	NA	NA
Breast-AdenoCa	110b5f05-ded7-45ec-b0f6-a941c92d77dd	2	76396574	76405914	15	9340	1	7	7	NA	NA	NA
Breast-AdenoCa	110b5f05-ded7-45ec-b0f6-a941c92d77dd	3	192369302	192369920	7	618	NA	1	6	NA	NA	NA
Breast-AdenoCa	110b5f05-ded7-45ec-b0f6-a941c92d77dd	4	41206862	41211336	9	4474	2	4	3	NA	NA	NA
Breast-AdenoCa	110b5f05-ded7-45ec-b0f6-a941c92d77dd	4	41305320	41306039	7	719	3	3	1	NA	NA	NA
Breast-AdenoCa	110b5f05-ded7-45ec-b0f6-a941c92d77dd	7	70211860	70212444	6	584	2	1	3	NA	NA	NA
Breast-AdenoCa	110b5f05-ded7-45ec-b0f6-a941c92d77dd	8	66771343	66773901	10	2558	2	3	4	NA	NA	1
Breast-AdenoCa	110b5f05-ded7-45ec-b0f6-a941c92d77dd	8	78053545	78056447	8	2902	1	4	3	NA	NA	NA
Breast-AdenoCa	110b5f05-ded7-45ec-b0f6-a941c92d77dd	8	78139990	78142535	7	2545	4	2	1	NA	NA	NA
Breast-AdenoCa	110b5f05-ded7-45ec-b0f6-a941c92d77dd	14	44538248	44543771	12	5523	3	6	2	NA	NA	1
Liver-HCC	1127b561-ea40-4d5e-95df-daa0a5ebc1e4	9	124755061	124755146	6	85	NA	NA	NA	3	3	NA
Panc-AdenoCA	1168944c-5451-4a18-8758-88bf5723bdd9	2	204085673	204088219	10	2546	1	4	5	NA	NA	NA
Prost-AdenoCA	120f01d1-8884-4aca-a1cb-36b207b2aa3a	2	40136342	40137951	7	1609	NA	3	4	NA	NA	NA
Prost-AdenoCA	120f01d1-8884-4aca-a1cb-36b207b2aa3a	3	32551659	32552687	10	1028	NA	7	3	NA	NA	NA
Prost-AdenoCA	120f01d1-8884-4aca-a1cb-36b207b2aa3a	3	40117312	40118738	15	1426	2	12	1	NA	NA	NA
Prost-AdenoCA	120f01d1-8884-4aca-a1cb-36b207b2aa3a	3	40218585	40219560	13	975	NA	8	5	NA	NA	NA
Prost-AdenoCA	120f01d1-8884-4aca-a1cb-36b207b2aa3a	3	45114244	45121141	12	6897	1	9	2	NA	NA	NA
Prost-AdenoCA	120f01d1-8884-4aca-a1cb-36b207b2aa3a	3	103006677	103011241	14	4564	NA	10	4	NA	NA	NA
Prost-AdenoCA	120f01d1-8884-4aca-a1cb-36b207b2aa3a	3	103310023	103317693	16	7670	1	11	4	NA	NA	NA
Prost-AdenoCA	120f01d1-8884-4aca-a1cb-36b207b2aa3a	3	113131642	113134310	7	2668	1	4	1	NA	1	NA
Prost-AdenoCA	120f01d1-8884-4aca-a1cb-36b207b2aa3a	3	118716218	118718606	12	2388	1	2	9	NA	NA	NA
Prost-AdenoCA	120f01d1-8884-4aca-a1cb-36b207b2aa3a	3	165847711	165849590	10	1879	2	6	2	NA	NA	NA
Prost-AdenoCA	120f01d1-8884-4aca-a1cb-36b207b2aa3a	3	165918824	165930127	15	11303	1	6	8	NA	NA	NA
Lymph-BNHL	124d5b04-4588-44c3-9c00-61e064cf1bdb	6	37138438	37141935	7	3497	NA	1	6	NA	NA	NA
Lymph-BNHL	124d5b04-4588-44c3-9c00-61e064cf1bdb	7	12940032	12944874	7	4842	NA	NA	NA	3	2	2
Lymph-BNHL	124d5b04-4588-44c3-9c00-61e064cf1bdb	11	35160904	35162626	13	1722	1	1	11	NA	NA	NA
Lymph-BNHL	124d5b04-4588-44c3-9c00-61e064cf1bdb	14	106094084	106094717	8	633	1	2	5	NA	NA	NA
Lymph-BNHL	124d5b04-4588-44c3-9c00-61e064cf1bdb	14	106110634	106114219	30	3585	2	2	25	NA	1	NA
Lymph-BNHL	124d5b04-4588-44c3-9c00-61e064cf1bdb	14	106174165	106175167	8	1002	NA	NA	8	NA	NA	NA
Lymph-BNHL	124d5b04-4588-44c3-9c00-61e064cf1bdb	14	106208559	106240262	42	31703	1	4	37	NA	NA	NA
Lymph-BNHL	124d5b04-4588-44c3-9c00-61e064cf1bdb	14	106326544	106357499	68	30955	3	10	41	10	NA	4
Lymph-BNHL	124d5b04-4588-44c3-9c00-61e064cf1bdb	14	106452876	106453335	8	459	NA	NA	5	2	NA	1
Lymph-BNHL	124d5b04-4588-44c3-9c00-61e064cf1bdb	15	36762667	36778049	28	15382	NA	NA	6	10	2	10
Lymph-BNHL	124d5b04-4588-44c3-9c00-61e064cf1bdb	22	23090009	23090387	8	378	NA	NA	4	1	NA	3
Lymph-BNHL	124d5b04-4588-44c3-9c00-61e064cf1bdb	22	23235999	23242186	11	6187	1	3	4	2	1	NA
Lymph-BNHL	124d5b04-4588-44c3-9c00-61e064cf1bdb	X	48774421	48776185	6	1764	1	NA	5	NA	NA	NA
Ovary-AdenoCA	127b0f7d-d24e-48b7-ac25-d3f14a43952d	5	26401409	26403671	13	2262	NA	9	4	NA	NA	NA
Ovary-AdenoCA	12874085-a596-41ae-b4cd-817cb1ebb278	13	59109120	59111819	7	2699	NA	1	6	NA	NA	NA
Ovary-AdenoCA	12874085-a596-41ae-b4cd-817cb1ebb278	13	63293437	63306105	18	12668	5	11	2	NA	NA	NA
Ovary-AdenoCA	12874085-a596-41ae-b4cd-817cb1ebb278	21	10836335	10838376	10	2041	1	9	NA	NA	NA	NA
Ovary-AdenoCA	1292e13b-d7c6-447b-a227-9a8113215580	12	69087004	69087020	6	16	NA	1	4	1	NA	NA
Panc-AdenoCA	129459c1-38be-45da-bb87-18848c97c544	1	234369859	234372326	15	2467	2	6	7	NA	NA	NA
Panc-AdenoCA	129459c1-38be-45da-bb87-18848c97c544	6	46178513	46180183	13	1670	1	8	4	NA	NA	NA
Panc-AdenoCA	129459c1-38be-45da-bb87-18848c97c544	10	42799029	42800833	18	1804	1	14	3	NA	NA	NA
Panc-AdenoCA	129459c1-38be-45da-bb87-18848c97c544	11	72875369	72875898	9	529	1	2	5	NA	NA	1
Panc-AdenoCA	129459c1-38be-45da-bb87-18848c97c544	15	84439343	84442681	6	3338	1	4	1	NA	NA	NA
Ovary-AdenoCA	129de5b2-d9b0-4762-9ef8-72d98231fb50	19	14856806	14857874	8	1068	NA	4	4	NA	NA	NA
Panc-AdenoCA	12f038e1-00af-4c64-a2e0-9e63323492ef	1	177387872	177391546	7	3674	2	2	3	NA	NA	NA
Panc-AdenoCA	12f038e1-00af-4c64-a2e0-9e63323492ef	2	30654305	30654528	7	223	NA	NA	7	NA	NA	NA
Panc-AdenoCA	12f038e1-00af-4c64-a2e0-9e63323492ef	2	31023723	31027802	7	4079	1	NA	6	NA	NA	NA
Panc-AdenoCA	12f038e1-00af-4c64-a2e0-9e63323492ef	2	38326416	38328040	8	1624	NA	NA	8	NA	NA	NA
Panc-AdenoCA	12f038e1-00af-4c64-a2e0-9e63323492ef	2	38462252	38467581	7	5329	NA	NA	6	1	NA	NA
Panc-AdenoCA	12f038e1-00af-4c64-a2e0-9e63323492ef	2	183188372	183194726	24	6354	3	13	7	NA	NA	1
Panc-AdenoCA	12f038e1-00af-4c64-a2e0-9e63323492ef	4	52752646	52753215	7	569	2	1	3	NA	NA	1
Panc-AdenoCA	12f038e1-00af-4c64-a2e0-9e63323492ef	6	92947783	92998384	52	50601	4	22	25	1	NA	NA
Panc-AdenoCA	12f038e1-00af-4c64-a2e0-9e63323492ef	6	121726741	121727241	7	500	NA	5	2	NA	NA	NA
Panc-AdenoCA	12f038e1-00af-4c64-a2e0-9e63323492ef	6	139309748	139311614	7	1866	1	6	NA	NA	NA	NA
Panc-AdenoCA	12f038e1-00af-4c64-a2e0-9e63323492ef	6	139718768	139720153	13	1385	2	9	2	NA	NA	NA
Panc-AdenoCA	12f038e1-00af-4c64-a2e0-9e63323492ef	13	90975395	90977150	6	1755	1	4	1	NA	NA	NA
Panc-AdenoCA	12f038e1-00af-4c64-a2e0-9e63323492ef	18	32678986	32679221	6	235	NA	6	NA	NA	NA	NA

Panc-AdenoCA	12f038e1-00af-4c64-a2e0-9e63323492ef	18	47978488	47978890	8	402	2	3	3	NA	NA	NA
Panc-AdenoCA	1327bbd4-a466-4e26-905f-cf91315f1cc8	3	162910888	162912403	20	1515	1	7	11	NA	NA	1
Panc-AdenoCA	1327bbd4-a466-4e26-905f-cf91315f1cc8	3	167441422	167443238	7	1816	NA	1	6	NA	NA	NA
Panc-AdenoCA	1327bbd4-a466-4e26-905f-cf91315f1cc8	3	168669548	168671841	17	2293	2	5	10	NA	NA	NA
Panc-AdenoCA	1327bbd4-a466-4e26-905f-cf91315f1cc8	18	18623623	18624236	9	613	NA	3	6	NA	NA	NA
Panc-AdenoCA	1327bbd4-a466-4e26-905f-cf91315f1cc8	18	22189728	22206665	22	16937	NA	10	11	NA	NA	1
Panc-AdenoCA	1327bbd4-a466-4e26-905f-cf91315f1cc8	18	22344298	22360355	23	16057	8	8	7	NA	NA	NA
Panc-AdenoCA	1327bbd4-a466-4e26-905f-cf91315f1cc8	18	22437538	22445829	11	8291	NA	5	6	NA	NA	NA
Panc-AdenoCA	1327bbd4-a466-4e26-905f-cf91315f1cc8	18	22741829	22779044	48	37215	9	12	27	NA	NA	NA
Panc-AdenoCA	1327bbd4-a466-4e26-905f-cf91315f1cc8	18	23039599	23046580	12	6981	1	4	7	NA	NA	NA
Panc-AdenoCA	1327bbd4-a466-4e26-905f-cf91315f1cc8	18	23535014	23541685	14	6671	4	2	8	NA	NA	NA
Panc-AdenoCA	1327bbd4-a466-4e26-905f-cf91315f1cc8	18	24416376	24418073	9	1697	1	3	5	NA	NA	NA
Lymph-CLL	132f7f2a-b902-4343-aa08-cf6a7af10b9a	4	182934246	182935350	6	1104	NA	NA	NA	2	1	3
Lymph-CLL	132f7f2a-b902-4343-aa08-cf6a7af10b9a	5	161217458	161220063	6	2605	NA	NA	1	3	1	1
Lymph-CLL	132f7f2a-b902-4343-aa08-cf6a7af10b9a	8	91186657	91193022	8	6365	NA	NA	1	3	1	3
Lymph-CLL	132f7f2a-b902-4343-aa08-cf6a7af10b9a	8	91202430	91213989	14	11559	NA	1	2	9	1	1
Lymph-CLL	132f7f2a-b902-4343-aa08-cf6a7af10b9a	14	106327223	106329359	39	2136	1	5	8	10	7	8
Lymph-CLL	132f7f2a-b902-4343-aa08-cf6a7af10b9a	18	60983860	60988646	18	4786	NA	3	4	7	2	2
Lymph-CLL	132f7f2a-b902-4343-aa08-cf6a7af10b9a	22	22730783	22735539	16	4756	1	1	3	7	NA	4
Lymph-CLL	132f7f2a-b902-4343-aa08-cf6a7af10b9a	22	23227800	23232541	9	4741	NA	1	4	2	1	1
Lymph-BNHL	140d5fa9-afbe-444e-a7e7-6a4cb4ab2923	1	203275080	203276101	10	1021	NA	1	7	NA	1	1
Lymph-BNHL	140d5fa9-afbe-444e-a7e7-6a4cb4ab2923	2	89157083	89185661	62	28578	2	8	20	17	8	7
Lymph-BNHL	140d5fa9-afbe-444e-a7e7-6a4cb4ab2923	3	187459364	187464254	6	4890	NA	1	3	NA	2	NA
Lymph-BNHL	140d5fa9-afbe-444e-a7e7-6a4cb4ab2923	6	392242	393466	7	1224	NA	NA	7	NA	NA	NA
Lymph-BNHL	140d5fa9-afbe-444e-a7e7-6a4cb4ab2923	7	53949471	53950233	11	762	NA	NA	1	5	NA	5
Lymph-BNHL	140d5fa9-afbe-444e-a7e7-6a4cb4ab2923	14	106237547	106239520	7	1973	NA	1	6	NA	NA	NA
Lymph-BNHL	140d5fa9-afbe-444e-a7e7-6a4cb4ab2923	14	106322630	106330276	99	7646	7	11	40	14	12	15
Lymph-BNHL	140d5fa9-afbe-444e-a7e7-6a4cb4ab2923	14	106725558	106726120	13	562	NA	2	1	7	1	2
Lymph-BNHL	140d5fa9-afbe-444e-a7e7-6a4cb4ab2923	22	23198299	23199186	9	887	1	2	3	2	1	NA
Lymph-BNHL	140d5fa9-afbe-444e-a7e7-6a4cb4ab2923	22	23227813	23248353	74	20540	8	15	32	12	1	6
Kidney-RCC	141918cd-3e23-45bc-96e1-b208c80f69e7	5	96446094	96447728	6	1634	4	2	NA	NA	NA	NA
Kidney-RCC	141918cd-3e23-45bc-96e1-b208c80f69e7	19	56366757	56370913	6	4156	NA	5	1	NA	NA	NA
Panc-AdenoCA	1447c8cb-25d4-4092-8919-4df08f898d2d	9	87918102	87922616	6	4514	1	2	3	NA	NA	NA
Liver-HCC	145f6dbb-9744-44f7-8485-e440689de3fd	4	185302403	185303263	7	860	1	3	3	NA	NA	NA
Lymph-NOS	1494bb16-f1f0-42a4-b10e-c383574cbc8b	1	28408514	28415422	15	6908	NA	1	2	6	3	3
Lymph-NOS	1494bb16-f1f0-42a4-b10e-c383574cbc8b	1	31202362	31204990	7	2628	NA	NA	4	1	2	NA
Lymph-NOS	1494bb16-f1f0-42a4-b10e-c383574cbc8b	1	31216019	31227557	46	11538	1	5	9	15	7	9
Lymph-NOS	1494bb16-f1f0-42a4-b10e-c383574cbc8b	3	80616008	80618005	15	1997	NA	NA	1	6	3	5
Lymph-NOS	1494bb16-f1f0-42a4-b10e-c383574cbc8b	3	128996107	128998908	7	2801	1	1	4	NA	NA	1
Lymph-NOS	1494bb16-f1f0-42a4-b10e-c383574cbc8b	3	187461933	187464777	19	2844	1	3	1	8	3	3
Lymph-NOS	1494bb16-f1f0-42a4-b10e-c383574cbc8b	3	187658389	187664421	13	6032	NA	NA	2	6	3	2
Lymph-NOS	1494bb16-f1f0-42a4-b10e-c383574cbc8b	3	187954365	187958948	11	4583	NA	1	5	2	NA	3
Lymph-NOS	1494bb16-f1f0-42a4-b10e-c383574cbc8b	4	15581060	15583853	7	2793	NA	NA	1	5	NA	1
Lymph-NOS	1494bb16-f1f0-42a4-b10e-c383574cbc8b	7	5568465	5570818	27	2353	1	8	8	8	1	1
Lymph-NOS	1494bb16-f1f0-42a4-b10e-c383574cbc8b	7	13723056	13734778	17	11722	NA	NA	1	8	4	4
Lymph-NOS	1494bb16-f1f0-42a4-b10e-c383574cbc8b	12	122457380	122463237	38	5857	NA	6	11	11	5	5
Lymph-NOS	1494bb16-f1f0-42a4-b10e-c383574cbc8b	14	69258594	69259292	11	698	NA	3	4	1	1	2
Lymph-NOS	1494bb16-f1f0-42a4-b10e-c383574cbc8b	14	106050735	106055960	9	5225	NA	NA	5	2	NA	2
Lymph-NOS	1494bb16-f1f0-42a4-b10e-c383574cbc8b	14	106071821	106096725	40	24904	1	6	14	10	3	6
Lymph-NOS	1494bb16-f1f0-42a4-b10e-c383574cbc8b	14	106323658	106329256	37	5598	2	10	8	11	3	3
Lymph-NOS	1494bb16-f1f0-42a4-b10e-c383574cbc8b	14	107209249	107232983	33	23734	1	4	6	16	4	2
Lymph-NOS	1494bb16-f1f0-42a4-b10e-c383574cbc8b	14	107258781	107259550	22	769	1	3	5	9	1	3
Lymph-NOS	1494bb16-f1f0-42a4-b10e-c383574cbc8b	16	85932062	85933387	11	1325	NA	2	NA	6	NA	3
Lymph-NOS	1494bb16-f1f0-42a4-b10e-c383574cbc8b	17	79478380	79482554	17	4174	1	4	1	8	2	1
Lymph-NOS	1494bb16-f1f0-42a4-b10e-c383574cbc8b	19	10304821	10308678	9	3857	NA	1	4	2	NA	2
Lymph-NOS	1494bb16-f1f0-42a4-b10e-c383574cbc8b	19	10340696	10353269	17	12573	NA	2	5	5	2	3
Lymph-NOS	1494bb16-f1f0-42a4-b10e-c383574cbc8b	22	22516823	22521836	12	5013	NA	2	5	1	NA	4
Lymph-NOS	1494bb16-f1f0-42a4-b10e-c383574cbc8b	22	22697876	22712051	41	14175	NA	1	7	17	11	5
Lymph-NOS	1494bb16-f1f0-42a4-b10e-c383574cbc8b	22	23028325	23029196	12	871	NA	4	3	3	NA	2
Lymph-NOS	1494bb16-f1f0-42a4-b10e-c383574cbc8b	22	23244121	23247591	12	3470	NA	4	NA	3	4	1

Cervix-SCC	14b8bbf2-310b-459b-b52d-a7ef510ce1cf	1	234488817	234496195	10	7378	3	1	6	NA	NA	NA
Cervix-SCC	14b8bbf2-310b-459b-b52d-a7ef510ce1cf	5	137767497	137769564	7	2067	1	3	3	NA	NA	NA
Cervix-SCC	14b8bbf2-310b-459b-b52d-a7ef510ce1cf	8	137207885	137212886	8	5001	1	2	5	NA	NA	NA
Cervix-SCC	14b8bbf2-310b-459b-b52d-a7ef510ce1cf	12	121372940	121374088	7	1148	NA	4	3	NA	NA	NA
Lung-AdenoCA	14bf9bfb-14d6-4cac-a556-828f680e8a15	1	32643702	32646291	6	2589	1	3	2	NA	NA	NA
Lung-AdenoCA	14bf9bfb-14d6-4cac-a556-828f680e8a15	1	167222582	167228647	10	6065	1	NA	9	NA	NA	NA
Lung-AdenoCA	14bf9bfb-14d6-4cac-a556-828f680e8a15	4	99329089	99333175	8	4086	NA	NA	8	NA	NA	NA
Lung-AdenoCA	14bf9bfb-14d6-4cac-a556-828f680e8a15	4	101906182	101915340	18	9158	NA	2	16	NA	NA	NA
Lung-AdenoCA	14bf9bfb-14d6-4cac-a556-828f680e8a15	4	166512348	166517698	20	5350	2	10	8	NA	NA	NA
Lung-AdenoCA	14bf9bfb-14d6-4cac-a556-828f680e8a15	5	161294279	161299238	7	4959	NA	NA	6	1	NA	NA
Lung-AdenoCA	14bf9bfb-14d6-4cac-a556-828f680e8a15	5	171674237	171675102	8	865	1	6	1	NA	NA	NA
Ovary-AdenoCA	14ed7388-41ed-43d4-afb2-04cd6410d5d2	8	42909953	42912234	8	2281	1	4	3	NA	NA	NA
Ovary-AdenoCA	14ed7388-41ed-43d4-afb2-04cd6410d5d2	11	40143235	40149417	17	6182	NA	10	7	NA	NA	NA
Ovary-AdenoCA	14ed7388-41ed-43d4-afb2-04cd6410d5d2	19	23114885	23117781	16	2896	2	8	6	NA	NA	NA
Ovary-AdenoCA	14ed7388-41ed-43d4-afb2-04cd6410d5d2	19	28494814	28496275	7	1461	3	3	1	NA	NA	NA
Ovary-AdenoCA	14ed7388-41ed-43d4-afb2-04cd6410d5d2	19	33883830	33884891	8	1061	1	1	6	NA	NA	NA
Prost-AdenoCA	1558629a-770a-4dcb-b0bf-9a8bcea9d80b	1	143383848	143384543	7	695	NA	3	4	NA	NA	NA
Prost-AdenoCA	1558629a-770a-4dcb-b0bf-9a8bcea9d80b	6	17166546	17168489	18	1943	NA	13	5	NA	NA	NA
Prost-AdenoCA	1558629a-770a-4dcb-b0bf-9a8bcea9d80b	10	92787655	92788276	8	621	NA	3	5	NA	NA	NA
Prost-AdenoCA	1558629a-770a-4dcb-b0bf-9a8bcea9d80b	10	92975712	92977269	9	1557	1	5	3	NA	NA	NA
Prost-AdenoCA	1558629a-770a-4dcb-b0bf-9a8bcea9d80b	X	120528044	120534497	12	6453	NA	7	5	NA	NA	NA
Kidney-RCC	155873b2-e5de-405f-87f6-6de0d238b160	1	51899830	51900410	8	580	NA	4	2	NA	NA	2
Lymph-BNHL	15678c3d-ce61-454d-849e-1d4ca358909b	1	20751459	20756123	7	4664	NA	NA	NA	4	2	1
Lymph-BNHL	15678c3d-ce61-454d-849e-1d4ca358909b	1	72188041	72190109	10	2068	NA	1	1	2	2	4
Lymph-BNHL	15678c3d-ce61-454d-849e-1d4ca358909b	2	58177060	58186985	22	9925	NA	2	4	12	2	2
Lymph-BNHL	15678c3d-ce61-454d-849e-1d4ca358909b	2	81402903	81406432	13	3529	NA	NA	1	4	4	4
Lymph-BNHL	15678c3d-ce61-454d-849e-1d4ca358909b	2	89126423	89128612	7	2189	2	1	3	NA	1	NA
Lymph-BNHL	15678c3d-ce61-454d-849e-1d4ca358909b	2	89155484	89160200	49	4716	3	7	7	16	11	5
Lymph-BNHL	15678c3d-ce61-454d-849e-1d4ca358909b	3	147719936	147723407	6	3471	NA	NA	1	3	1	1
Lymph-BNHL	15678c3d-ce61-454d-849e-1d4ca358909b	3	164340006	164341044	7	1038	NA	NA	1	NA	2	4
Lymph-BNHL	15678c3d-ce61-454d-849e-1d4ca358909b	4	166402103	166402242	6	139	NA	NA	NA	3	2	1
Lymph-BNHL	15678c3d-ce61-454d-849e-1d4ca358909b	5	129799108	129802164	14	3056	1	1	4	1	4	3
Lymph-BNHL	15678c3d-ce61-454d-849e-1d4ca358909b	6	37130020	37134307	6	4287	1	1	1	1	1	1
Lymph-BNHL	15678c3d-ce61-454d-849e-1d4ca358909b	6	91005079	91007026	13	1947	NA	2	8	2	NA	1
Lymph-BNHL	15678c3d-ce61-454d-849e-1d4ca358909b	11	87246583	87250856	7	4273	1	NA	NA	1	1	4
Lymph-BNHL	15678c3d-ce61-454d-849e-1d4ca358909b	12	122459020	122463315	8	4295	NA	4	3	NA	1	NA
Lymph-BNHL	15678c3d-ce61-454d-849e-1d4ca358909b	13	69272722	69275330	7	2608	1	NA	2	NA	2	2
Lymph-BNHL	15678c3d-ce61-454d-849e-1d4ca358909b	14	106112939	106114419	13	1480	2	2	9	NA	NA	NA
Lymph-BNHL	15678c3d-ce61-454d-849e-1d4ca358909b	14	106326608	106330044	162	3436	9	32	41	24	28	28
Lymph-BNHL	15678c3d-ce61-454d-849e-1d4ca358909b	14	106994195	106995031	22	836	1	2	5	5	5	4
Lymph-BNHL	15678c3d-ce61-454d-849e-1d4ca358909b	14	107175890	107179639	22	3749	2	6	4	6	2	2
Lymph-BNHL	15678c3d-ce61-454d-849e-1d4ca358909b	17	68001315	68005345	6	4030	NA	2	1	NA	2	1
Lymph-BNHL	15678c3d-ce61-454d-849e-1d4ca358909b	18	60805267	60809501	10	4234	NA	2	NA	3	3	2
Lymph-BNHL	15678c3d-ce61-454d-849e-1d4ca358909b	18	60984221	60988552	56	4331	3	12	21	8	7	5
Lymph-BNHL	15678c3d-ce61-454d-849e-1d4ca358909b	18	72804180	72807104	7	2924	NA	1	1	2	2	1
Lymph-BNHL	15678c3d-ce61-454d-849e-1d4ca358909b	22	22724243	22724576	6	333	NA	NA	4	2	NA	NA
Lymph-BNHL	15678c3d-ce61-454d-849e-1d4ca358909b	22	22730431	22735384	25	4953	1	3	13	4	2	2
Lymph-BNHL	15678c3d-ce61-454d-849e-1d4ca358909b	22	23223154	23247604	48	24450	1	8	23	6	3	7
Lymph-BNHL	15678c3d-ce61-454d-849e-1d4ca358909b	X	46543184	46543429	8	245	NA	NA	NA	6	1	1
Liver-HCC	15895218-c623-11e3-bf01-24c6515278c0	4	45345005	45346316	9	1311	1	5	3	NA	NA	NA
Liver-HCC	15895218-c623-11e3-bf01-24c6515278c0	14	24261042	24264179	10	3137	2	1	7	NA	NA	NA
Liver-HCC	15895218-c623-11e3-bf01-24c6515278c0	14	32049613	32051922	10	2309	NA	5	4	1	NA	NA
Kidney-RCC	15a93657-0521-430a-a816-bf0842374b75	1	192094352	192096457	9	2105	2	2	5	NA	NA	NA
Eso-AdenoCa	15e7d981-8c27-4b2b-b4f8-626e22021895	10	87122788	87123375	31	587	NA	NA	NA	31	NA	NA
Eso-AdenoCa	15e7d981-8c27-4b2b-b4f8-626e22021895	10	116523822	116525314	7	1492	2	5	NA	NA	NA	NA
Eso-AdenoCa	15e7d981-8c27-4b2b-b4f8-626e22021895	19	30397855	30402083	8	4228	1	NA	6	1	NA	NA
Breast-AdenoCa	15f90ef0-831b-40a3-98bd-ec226a9e8b26	1	239243091	239244448	8	1357	2	2	4	NA	NA	NA
Breast-AdenoCa	15f90ef0-831b-40a3-98bd-ec226a9e8b26	9	36640510	36640811	6	301	NA	3	2	1	NA	NA
Liver-HCC	15fd8dc8-c622-11e3-bf01-24c6515278c0	9	43459840	43460190	6	350	NA	NA	6	NA	NA	NA
Liver-HCC	15fd8dc8-c622-11e3-bf01-24c6515278c0	9	136610777	136615626	8	4849	NA	1	7	NA	NA	NA

Ovary-AdenoCA	1659bae5-3140-4d05-891c-81b48277b2fc	6	167018772	167019893	9	1121	3	3	3	NA	NA	NA
Ovary-AdenoCA	1659bae5-3140-4d05-891c-81b48277b2fc	11	81448219	81458725	22	10506	10	8	4	NA	NA	NA
Ovary-AdenoCA	1659bae5-3140-4d05-891c-81b48277b2fc	11	83659695	83662240	18	2545	3	11	4	NA	NA	NA
Bone-Leiomyo	16d33e09-2e21-4da2-8e57-e78ce28c4408	1	196584116	196584733	7	617	NA	5	2	NA	NA	NA
Bone-Leiomyo	16d33e09-2e21-4da2-8e57-e78ce28c4408	2	111887286	111887877	11	591	1	1	9	NA	NA	NA
Bone-Leiomyo	16d33e09-2e21-4da2-8e57-e78ce28c4408	2	118459914	118461910	11	1996	NA	9	2	NA	NA	NA
Bone-Leiomyo	16d33e09-2e21-4da2-8e57-e78ce28c4408	4	129852096	129855825	8	3729	2	3	3	NA	NA	NA
Bone-Leiomyo	16d33e09-2e21-4da2-8e57-e78ce28c4408	7	57291428	57294889	9	3461	3	3	2	NA	1	NA
Bone-Leiomyo	16d33e09-2e21-4da2-8e57-e78ce28c4408	8	1768250	1768928	6	678	2	1	3	NA	NA	NA
Bone-Leiomyo	16d33e09-2e21-4da2-8e57-e78ce28c4408	8	3490865	3491582	12	717	1	9	2	NA	NA	NA
Bone-Leiomyo	16d33e09-2e21-4da2-8e57-e78ce28c4408	10	58566056	58574882	37	8826	3	21	13	NA	NA	NA
Bone-Leiomyo	16d33e09-2e21-4da2-8e57-e78ce28c4408	10	67294267	67303896	42	9629	4	25	13	NA	NA	NA
Bone-Leiomyo	16d33e09-2e21-4da2-8e57-e78ce28c4408	12	30226614	30227835	7	1221	2	2	3	NA	NA	NA
Bone-Leiomyo	16d33e09-2e21-4da2-8e57-e78ce28c4408	12	33663896	33669813	20	5917	2	13	4	NA	NA	1
Bone-Leiomyo	16d33e09-2e21-4da2-8e57-e78ce28c4408	13	69765631	69766619	6	988	NA	3	3	NA	NA	NA
Bone-Leiomyo	16d33e09-2e21-4da2-8e57-e78ce28c4408	15	24306118	24308408	12	2290	NA	6	6	NA	NA	NA
Bone-Leiomyo	16d33e09-2e21-4da2-8e57-e78ce28c4408	18	19685647	19686695	6	1048	NA	4	2	NA	NA	NA
Bone-Leiomyo	16d33e09-2e21-4da2-8e57-e78ce28c4408	18	23348064	23351605	8	3541	2	3	3	NA	NA	NA
Bone-Leiomyo	16d33e09-2e21-4da2-8e57-e78ce28c4408	18	23633137	23634194	11	1057	1	6	4	NA	NA	NA
Bone-Leiomyo	16d33e09-2e21-4da2-8e57-e78ce28c4408	18	24327383	24327828	10	445	NA	7	3	NA	NA	NA
Bone-Leiomyo	16d33e09-2e21-4da2-8e57-e78ce28c4408	18	58589881	58591234	8	1353	2	1	5	NA	NA	NA
Bone-Leiomyo	16d33e09-2e21-4da2-8e57-e78ce28c4408	18	74587263	74592142	14	4879	2	8	4	NA	NA	NA
Bone-Leiomyo	16d33e09-2e21-4da2-8e57-e78ce28c4408	21	38126441	38128141	9	1700	1	4	4	NA	NA	NA
Bone-Leiomyo	16d33e09-2e21-4da2-8e57-e78ce28c4408	X	81810293	81810530	8	237	NA	5	2	1	NA	NA
Bone-Leiomyo	16d33e09-2e21-4da2-8e57-e78ce28c4408	X	117937685	117940683	17	2998	NA	9	8	NA	NA	NA
Ovary-AdenoCA	16df7888-2480-4394-8856-d57a6ef371d2	1	217629037	217634470	23	5433	5	16	2	NA	NA	NA
Ovary-AdenoCA	16df7888-2480-4394-8856-d57a6ef371d2	2	42281199	42286224	8	5025	1	4	3	NA	NA	NA
Ovary-AdenoCA	16df7888-2480-4394-8856-d57a6ef371d2	2	230212180	230217811	18	5631	2	10	6	NA	NA	NA
Ovary-AdenoCA	16df7888-2480-4394-8856-d57a6ef371d2	4	186798485	186802261	6	3776	1	2	3	NA	NA	NA
Ovary-AdenoCA	16df7888-2480-4394-8856-d57a6ef371d2	19	5102643	5106594	15	3951	2	9	4	NA	NA	NA
Ovary-AdenoCA	16df7888-2480-4394-8856-d57a6ef371d2	19	27799575	27801221	7	1646	2	1	4	NA	NA	NA
Ovary-AdenoCA	16df7888-2480-4394-8856-d57a6ef371d2	19	28323458	28325690	9	2232	2	3	4	NA	NA	NA
Ovary-AdenoCA	16df7888-2480-4394-8856-d57a6ef371d2	19	29591199	29592610	13	1411	1	2	10	NA	NA	NA
Ovary-AdenoCA	16df7888-2480-4394-8856-d57a6ef371d2	20	2160863	2164280	10	3417	2	4	3	NA	1	NA
Ovary-AdenoCA	16df7888-2480-4394-8856-d57a6ef371d2	20	6609152	6616597	12	7445	3	7	2	NA	NA	NA
Ovary-AdenoCA	16df7888-2480-4394-8856-d57a6ef371d2	20	9416043	9427410	15	11367	2	8	5	NA	NA	NA
Ovary-AdenoCA	16df7888-2480-4394-8856-d57a6ef371d2	20	14521950	14530961	15	9011	4	9	2	NA	NA	NA
Ovary-AdenoCA	16df7888-2480-4394-8856-d57a6ef371d2	20	14583351	14586033	14	2682	9	5	NA	NA	NA	NA
Ovary-AdenoCA	16df7888-2480-4394-8856-d57a6ef371d2	20	17208204	17211354	16	3150	4	7	5	NA	NA	NA
Ovary-AdenoCA	16df7888-2480-4394-8856-d57a6ef371d2	20	22589316	22604236	17	14920	4	9	4	NA	NA	NA
Ovary-AdenoCA	16df7888-2480-4394-8856-d57a6ef371d2	20	24009798	24012571	7	2773	3	2	2	NA	NA	NA
Ovary-AdenoCA	16df7888-2480-4394-8856-d57a6ef371d2	20	24627380	24631408	7	4028	1	5	1	NA	NA	NA
Panc-AdenoCA	170ad241-9e24-469b-b7ca-3fd783a89b21	1	234026453	234029080	43	2627	8	20	15	NA	NA	NA
Panc-AdenoCA	170ad241-9e24-469b-b7ca-3fd783a89b21	2	100816182	100818820	14	2638	1	6	7	NA	NA	NA
Panc-AdenoCA	170ad241-9e24-469b-b7ca-3fd783a89b21	2	107265163	107266852	7	1689	3	2	2	NA	NA	NA
Panc-AdenoCA	170ad241-9e24-469b-b7ca-3fd783a89b21	2	167493127	167496440	8	3313	NA	NA	8	NA	NA	NA
Panc-AdenoCA	170ad241-9e24-469b-b7ca-3fd783a89b21	12	7450738	7453870	9	3132	2	4	2	NA	NA	1
Panc-AdenoCA	170ad241-9e24-469b-b7ca-3fd783a89b21	12	55668169	55670629	7	2460	NA	NA	7	NA	NA	NA
Panc-AdenoCA	170ad241-9e24-469b-b7ca-3fd783a89b21	12	55974012	55976277	19	2265	NA	NA	19	NA	NA	NA
Panc-AdenoCA	170ad241-9e24-469b-b7ca-3fd783a89b21	17	38101622	38103058	7	1436	1	4	2	NA	NA	NA
Panc-AdenoCA	170ad241-9e24-469b-b7ca-3fd783a89b21	19	22668388	22671790	22	3402	1	NA	21	NA	NA	NA
Panc-AdenoCA	170ad241-9e24-469b-b7ca-3fd783a89b21	19	22760572	22761155	7	583	NA	NA	7	NA	NA	NA
Panc-AdenoCA	170ad241-9e24-469b-b7ca-3fd783a89b21	19	22969449	22979975	36	10526	2	5	27	2	NA	NA
Panc-AdenoCA	170ad241-9e24-469b-b7ca-3fd783a89b21	19	28914316	28915466	10	1150	2	3	5	NA	NA	NA
Panc-AdenoCA	170ad241-9e24-469b-b7ca-3fd783a89b21	19	32076681	32086025	14	9344	1	9	3	1	NA	NA
Head-SCC	1727e88b-df0a-4af2-9191-8c6061d98ad0	7	54334813	54336715	9	1902	1	2	6	NA	NA	NA
Head-SCC	1727e88b-df0a-4af2-9191-8c6061d98ad0	18	21577936	21580553	10	2617	1	4	5	NA	NA	NA
Stomach-AdenoCA	172c9864-c26d-4e9b-947a-e8ce761e996d	9	3457828	3458578	31	750	NA	NA	31	NA	NA	NA
Stomach-AdenoCA	172c9864-c26d-4e9b-947a-e8ce761e996d	12	69706431	69706926	7	495	3	4	NA	NA	NA	NA
Panc-AdenoCA	1776f326-b12f-4355-8107-80fa6fcd2159	10	35638751	35638963	6	212	NA	2	4	NA	NA	NA

Panc-AdenoCA	1776f326-b12f-4355-8107-80fa6fcd2159	15	98205509	98207004	12	1495	NA	7	5	NA	NA	NA
Liver-HCC	17de5b12-c623-11e3-bf01-24c6515278c0	1	38808882	38817102	18	8220	3	3	11	NA	NA	1
Liver-HCC	17de5b12-c623-11e3-bf01-24c6515278c0	6	3962243	3962933	11	690	1	6	4	NA	NA	NA
Liver-HCC	17de5b12-c623-11e3-bf01-24c6515278c0	6	27264260	27264817	7	557	2	1	4	NA	NA	NA
Liver-HCC	17de5b12-c623-11e3-bf01-24c6515278c0	6	28800150	28802851	7	2701	2	2	3	NA	NA	NA
Ovary-AdenoCA	17ed8831-a261-42d9-8ff3-cf75a6cb2a24	7	38698836	38702340	6	3504	NA	6	NA	NA	NA	NA
Ovary-AdenoCA	17ed8831-a261-42d9-8ff3-cf75a6cb2a24	8	118565026	118567593	6	2567	NA	3	3	NA	NA	NA
Ovary-AdenoCA	17ed8831-a261-42d9-8ff3-cf75a6cb2a24	10	6125464	6126072	6	608	4	2	NA	NA	NA	NA
Ovary-AdenoCA	17ed8831-a261-42d9-8ff3-cf75a6cb2a24	11	78147407	78147889	7	482	NA	2	5	NA	NA	NA
Ovary-AdenoCA	17ed8831-a261-42d9-8ff3-cf75a6cb2a24	12	38307950	38309709	6	1759	NA	3	2	1	NA	NA
Ovary-AdenoCA	17ed8831-a261-42d9-8ff3-cf75a6cb2a24	18	5913327	5913678	7	351	1	2	4	NA	NA	NA
Ovary-AdenoCA	17ed8831-a261-42d9-8ff3-cf75a6cb2a24	19	29106972	29107897	7	925	1	2	4	NA	NA	NA
Ovary-AdenoCA	17ed8831-a261-42d9-8ff3-cf75a6cb2a24	21	45234891	45235979	9	1088	NA	4	5	NA	NA	NA
Panc-AdenoCA	183b04f5-107f-496c-8e9a-8263dd00cffa	2	157189342	157190268	6	926	NA	NA	6	NA	NA	NA
Panc-AdenoCA	183b04f5-107f-496c-8e9a-8263dd00cffa	18	22458988	22461210	15	2222	4	7	4	NA	NA	NA
Bone-Leiomyo	18ec066e-8510-4921-9e35-45d85fb01e38	1	164366233	164370534	8	4301	NA	NA	7	NA	1	NA
Bone-Leiomyo	18ec066e-8510-4921-9e35-45d85fb01e38	1	167375692	167381363	7	5671	NA	NA	7	NA	NA	NA
Bone-Leiomyo	18ec066e-8510-4921-9e35-45d85fb01e38	1	171889798	171890974	8	1176	NA	1	7	NA	NA	NA
Bone-Leiomyo	18ec066e-8510-4921-9e35-45d85fb01e38	1	171979194	171985028	8	5834	2	NA	6	NA	NA	NA
Bone-Leiomyo	18ec066e-8510-4921-9e35-45d85fb01e38	5	273765	274619	6	854	NA	1	4	NA	1	NA
Bone-Leiomyo	18ec066e-8510-4921-9e35-45d85fb01e38	5	10932990	10938624	14	5634	5	1	8	NA	NA	NA
Bone-Leiomyo	18ec066e-8510-4921-9e35-45d85fb01e38	5	12400492	12402855	9	2363	2	3	4	NA	NA	NA
Bone-Leiomyo	18ec066e-8510-4921-9e35-45d85fb01e38	5	13351376	13355598	8	4222	NA	2	6	NA	NA	NA
Bone-Leiomyo	18ec066e-8510-4921-9e35-45d85fb01e38	5	32088579	32090098	8	1519	NA	2	6	NA	NA	NA
Bone-Leiomyo	18ec066e-8510-4921-9e35-45d85fb01e38	12	18835046	18835224	6	178	NA	NA	6	NA	NA	NA
Bone-Leiomyo	18ec066e-8510-4921-9e35-45d85fb01e38	12	58167474	58168791	9	1317	4	3	2	NA	NA	NA
Bone-Leiomyo	18ec066e-8510-4921-9e35-45d85fb01e38	12	58311828	58312083	7	255	1	2	4	NA	NA	NA
Bone-Leiomyo	18ec066e-8510-4921-9e35-45d85fb01e38	12	58547509	58550563	9	3054	3	3	3	NA	NA	NA
Bone-Leiomyo	18ec066e-8510-4921-9e35-45d85fb01e38	12	69921692	69932123	14	10431	5	2	6	NA	1	NA
Bone-Leiomyo	18ec066e-8510-4921-9e35-45d85fb01e38	12	85049364	85051646	8	2282	2	2	4	NA	NA	NA
Bone-Leiomyo	18ec066e-8510-4921-9e35-45d85fb01e38	12	85175407	85179707	11	4300	1	2	8	NA	NA	NA
Bone-Leiomyo	18ec066e-8510-4921-9e35-45d85fb01e38	19	7512100	7515008	11	2908	3	NA	8	NA	NA	NA
Liver-HCC	18f5e75e-c623-11e3-bf01-24c6515278c0	19	22936045	22936599	7	554	NA	4	3	NA	NA	NA
Head-SCC	190eb791-383d-4e27-870d-e470630843e7	2	97023041	97024809	6	1768	1	3	2	NA	NA	NA
Head-SCC	190eb791-383d-4e27-870d-e470630843e7	2	197989072	197991746	42	2674	4	17	19	NA	NA	2
Head-SCC	190eb791-383d-4e27-870d-e470630843e7	8	32080547	32082782	15	2235	5	4	6	NA	NA	NA
Head-SCC	190eb791-383d-4e27-870d-e470630843e7	11	27509316	27510673	14	1357	2	5	7	NA	NA	NA
Uterus-AdenoCA	19c1c97f-a3ec-44a8-8a20-6f97caed1a4f	1	116888377	116894188	17	5811	4	7	6	NA	NA	NA
Panc-AdenoCA	1a0ef226-41a5-4b56-9078-4b7834f0c976	2	96765877	96768253	8	2376	1	4	3	NA	NA	NA
Panc-AdenoCA	1a0ef226-41a5-4b56-9078-4b7834f0c976	2	240654718	240654750	9	32	NA	2	3	4	NA	NA
Panc-AdenoCA	1a0ef226-41a5-4b56-9078-4b7834f0c976	4	65113518	65115209	9	1691	2	4	3	NA	NA	NA
Panc-AdenoCA	1a0ef226-41a5-4b56-9078-4b7834f0c976	9	30205059	30205365	8	306	2	1	5	NA	NA	NA
Panc-AdenoCA	1a0ef226-41a5-4b56-9078-4b7834f0c976	9	31877468	31882661	15	5193	1	1	13	NA	NA	NA
Panc-AdenoCA	1a0ef226-41a5-4b56-9078-4b7834f0c976	16	4395617	4396571	7	954	NA	2	4	NA	NA	1
Stomach-AdenoCA	1a391468-fbf3-451a-b88e-a257a26d047f	19	28396652	28410875	27	14223	8	9	10	NA	NA	NA
Stomach-AdenoCA	1a391468-fbf3-451a-b88e-a257a26d047f	19	28448850	28453412	11	4562	3	5	3	NA	NA	NA
Stomach-AdenoCA	1a391468-fbf3-451a-b88e-a257a26d047f	19	28881787	28886071	9	4284	1	4	3	NA	1	NA
Stomach-AdenoCA	1a391468-fbf3-451a-b88e-a257a26d047f	19	29371828	29374252	13	2424	4	5	4	NA	NA	NA
Stomach-AdenoCA	1a391468-fbf3-451a-b88e-a257a26d047f	19	29461338	29462907	8	1569	NA	1	7	NA	NA	NA
Stomach-AdenoCA	1a391468-fbf3-451a-b88e-a257a26d047f	19	29642933	29646873	14	3940	2	6	6	NA	NA	NA
Stomach-AdenoCA	1a391468-fbf3-451a-b88e-a257a26d047f	19	30272261	30277404	15	5143	5	5	5	NA	NA	NA
Lymph-BNHL	1a4633c4-72a0-4e30-8c4c-345e04337627	1	203274956	203275552	7	596	2	1	3	NA	NA	1
Lymph-BNHL	1a4633c4-72a0-4e30-8c4c-345e04337627	6	37138285	37140654	12	2369	NA	1	11	NA	NA	NA
Lymph-BNHL	1a4633c4-72a0-4e30-8c4c-345e04337627	9	37025352	37026569	7	1217	1	NA	6	NA	NA	NA
Lymph-BNHL	1a4633c4-72a0-4e30-8c4c-345e04337627	14	106091059	106094991	9	3932	1	2	6	NA	NA	NA
Lymph-BNHL	1a4633c4-72a0-4e30-8c4c-345e04337627	14	106108302	106114499	54	6197	7	5	42	NA	NA	NA
Lymph-BNHL	1a4633c4-72a0-4e30-8c4c-345e04337627	14	106208777	106375213	254	2E+05	18	44	##	22	6	16
Lymph-BNHL	1a4633c4-72a0-4e30-8c4c-345e04337627	14	106926355	106934314	28	7959	NA	6	8	5	3	6
Lymph-BNHL	1a4633c4-72a0-4e30-8c4c-345e04337627	22	22568740	22569526	10	786	NA	1	3	4	NA	2
Lymph-BNHL	1a4633c4-72a0-4e30-8c4c-345e04337627	22	23227979	23231983	18	4004	2	2	12	1	NA	1

Panc-AdenoCA	1a841850-fca0-48ab-8c78-db0bd5a64435	3	84475238	84481241	12	6003	NA	3	9	NA	NA	NA
Panc-AdenoCA	1a841850-fca0-48ab-8c78-db0bd5a64435	7	16270991	16272857	8	1866	NA	3	5	NA	NA	NA
Panc-AdenoCA	1a841850-fca0-48ab-8c78-db0bd5a64435	7	27225955	27227213	11	1258	2	3	6	NA	NA	NA
Panc-AdenoCA	1a841850-fca0-48ab-8c78-db0bd5a64435	9	26622789	26627014	19	4225	1	5	13	NA	NA	NA
Panc-AdenoCA	1a841850-fca0-48ab-8c78-db0bd5a64435	10	84786127	84790035	12	3908	1	4	7	NA	NA	NA
Panc-AdenoCA	1a841850-fca0-48ab-8c78-db0bd5a64435	18	19064985	19067205	8	2220	NA	1	7	NA	NA	NA
Panc-AdenoCA	1a841850-fca0-48ab-8c78-db0bd5a64435	18	19297358	19298162	10	804	1	NA	9	NA	NA	NA
Panc-AdenoCA	1a841850-fca0-48ab-8c78-db0bd5a64435	18	22429021	22434787	8	5766	1	2	3	NA	1	1
Skin-Melanoma	1ac15380-04a2-42dd-8ade-28556a570e80	1	19680187	19686251	15	6064	4	2	9	NA	NA	NA
Skin-Melanoma	1ac15380-04a2-42dd-8ade-28556a570e80	1	21849931	21852094	10	2163	2	3	5	NA	NA	NA
Skin-Melanoma	1ac15380-04a2-42dd-8ade-28556a570e80	1	22438518	22439353	8	835	NA	4	4	NA	NA	NA
Skin-Melanoma	1ac15380-04a2-42dd-8ade-28556a570e80	1	30028117	30032867	8	4750	1	2	5	NA	NA	NA
Skin-Melanoma	1ac15380-04a2-42dd-8ade-28556a570e80	1	30619637	30623945	14	4308	2	9	2	NA	1	NA
Skin-Melanoma	1ac15380-04a2-42dd-8ade-28556a570e80	1	48065546	48068069	7	2523	NA	NA	7	NA	NA	NA
Skin-Melanoma	1ac15380-04a2-42dd-8ade-28556a570e80	1	49105852	49110786	10	4934	NA	4	6	NA	NA	NA
Skin-Melanoma	1ac15380-04a2-42dd-8ade-28556a570e80	1	50161663	50162644	9	981	2	4	3	NA	NA	NA
Skin-Melanoma	1ac15380-04a2-42dd-8ade-28556a570e80	1	237591836	237592560	7	724	NA	3	4	NA	NA	NA
Skin-Melanoma	1ac15380-04a2-42dd-8ade-28556a570e80	2	1525905	1525935	6	30	NA	1	2	2	1	NA
Skin-Melanoma	1ac15380-04a2-42dd-8ade-28556a570e80	2	232441250	232442589	7	1339	NA	NA	7	NA	NA	NA
Skin-Melanoma	1ac15380-04a2-42dd-8ade-28556a570e80	5	3796904	3797360	7	456	NA	NA	7	NA	NA	NA
Skin-Melanoma	1ac15380-04a2-42dd-8ade-28556a570e80	5	17945974	17951591	9	5617	1	2	6	NA	NA	NA
Skin-Melanoma	1ac15380-04a2-42dd-8ade-28556a570e80	5	42487372	42490540	14	3168	5	4	5	NA	NA	NA
Skin-Melanoma	1ac15380-04a2-42dd-8ade-28556a570e80	5	167162274	167165050	12	2776	2	3	7	NA	NA	NA
Skin-Melanoma	1ac15380-04a2-42dd-8ade-28556a570e80	5	174187497	174190005	12	2508	2	5	4	NA	1	NA
Skin-Melanoma	1ac15380-04a2-42dd-8ade-28556a570e80	9	24544568	24547168	15	2600	2	7	6	NA	NA	NA
Skin-Melanoma	1ac15380-04a2-42dd-8ade-28556a570e80	12	48311968	48320722	14	8754	1	4	8	NA	NA	1
Skin-Melanoma	1ac15380-04a2-42dd-8ade-28556a570e80	12	49910278	49910741	8	463	2	1	5	NA	NA	NA
Skin-Melanoma	1ac15380-04a2-42dd-8ade-28556a570e80	12	58008522	58010400	9	1878	NA	NA	9	NA	NA	NA
Skin-Melanoma	1ac15380-04a2-42dd-8ade-28556a570e80	12	67259642	67267193	11	7551	1	6	4	NA	NA	NA
Skin-Melanoma	1ac15380-04a2-42dd-8ade-28556a570e80	12	69288427	69292085	7	3658	1	1	5	NA	NA	NA
Skin-Melanoma	1ac15380-04a2-42dd-8ade-28556a570e80	12	69559045	69565395	11	6350	NA	4	7	NA	NA	NA
Skin-Melanoma	1ac15380-04a2-42dd-8ade-28556a570e80	12	69712493	69713712	8	1219	NA	5	3	NA	NA	NA
Skin-Melanoma	1ac15380-04a2-42dd-8ade-28556a570e80	12	69958424	69958684	7	260	NA	2	5	NA	NA	NA
Skin-Melanoma	1ac15380-04a2-42dd-8ade-28556a570e80	12	70523831	70529033	7	5202	1	1	5	NA	NA	NA
Skin-Melanoma	1ac15380-04a2-42dd-8ade-28556a570e80	12	70532174	70543292	13	11118	2	6	5	NA	NA	NA
Skin-Melanoma	1ac15380-04a2-42dd-8ade-28556a570e80	12	70592971	70593396	8	425	NA	4	4	NA	NA	NA
Skin-Melanoma	1ac15380-04a2-42dd-8ade-28556a570e80	12	70703599	70704610	11	1011	NA	1	10	NA	NA	NA
Skin-Melanoma	1ac15380-04a2-42dd-8ade-28556a570e80	12	71827719	71844071	29	16352	1	9	19	NA	NA	NA
Skin-Melanoma	1ac15380-04a2-42dd-8ade-28556a570e80	12	71944033	71948795	8	4762	NA	2	6	NA	NA	NA
Skin-Melanoma	1ac15380-04a2-42dd-8ade-28556a570e80	12	71984327	71986404	8	2077	NA	5	3	NA	NA	NA
Skin-Melanoma	1ac15380-04a2-42dd-8ade-28556a570e80	12	71997829	71998704	10	875	2	6	2	NA	NA	NA
Skin-Melanoma	1ac15380-04a2-42dd-8ade-28556a570e80	12	73011327	73012077	8	750	2	1	5	NA	NA	NA
Skin-Melanoma	1ac15380-04a2-42dd-8ade-28556a570e80	12	83320759	83320956	7	197	NA	4	2	NA	NA	1
Skin-Melanoma	1ac15380-04a2-42dd-8ade-28556a570e80	12	84107696	84108138	7	442	NA	5	2	NA	NA	NA
Skin-Melanoma	1ac15380-04a2-42dd-8ade-28556a570e80	12	84657435	84680030	35	22595	4	8	21	1	1	NA
Skin-Melanoma	1ac15380-04a2-42dd-8ade-28556a570e80	12	85734195	85739998	8	5803	NA	2	6	NA	NA	NA
Skin-Melanoma	1ac15380-04a2-42dd-8ade-28556a570e80	12	86155711	86156534	8	823	NA	NA	8	NA	NA	NA
Skin-Melanoma	1ac15380-04a2-42dd-8ade-28556a570e80	12	86336848	86337712	7	864	1	6	NA	NA	NA	NA
Skin-Melanoma	1ac15380-04a2-42dd-8ade-28556a570e80	12	115678667	115681400	15	2733	2	7	5	NA	1	NA
Skin-Melanoma	1ac15380-04a2-42dd-8ade-28556a570e80	12	122494623	122495238	7	615	NA	NA	7	NA	NA	NA
Panc-AdenoCA	1ac54c6e-a1e4-4f3b-b4a5-cdb327e0dca6	4	66250943	66251913	6	970	1	2	3	NA	NA	NA
Panc-AdenoCA	1ac54c6e-a1e4-4f3b-b4a5-cdb327e0dca6	4	66691733	66692794	10	1061	2	4	4	NA	NA	NA
Panc-AdenoCA	1ac54c6e-a1e4-4f3b-b4a5-cdb327e0dca6	X	72590829	72594406	6	3577	1	2	3	NA	NA	NA
Head-SCC	1aff91a6-1b0f-4575-8f4b-4e064a50b886	6	97556427	97557547	6	1120	3	1	2	NA	NA	NA
Head-SCC	1aff91a6-1b0f-4575-8f4b-4e064a50b886	7	22487775	22488439	11	664	NA	6	5	NA	NA	NA
Head-SCC	1aff91a6-1b0f-4575-8f4b-4e064a50b886	8	100774612	100778662	8	4050	2	1	5	NA	NA	NA
Head-SCC	1aff91a6-1b0f-4575-8f4b-4e064a50b886	9	37094954	37095165	8	211	2	2	4	NA	NA	NA
Panc-AdenoCA	1b0fe1d5-b286-4f49-ae92-94d9c6db9657	4	81478176	81480263	13	2087	1	3	9	NA	NA	NA
Panc-AdenoCA	1b0fe1d5-b286-4f49-ae92-94d9c6db9657	21	22365346	22365740	8	394	3	2	3	NA	NA	NA
Lymph-BNHL	1b1780d5-06da-40ee-9e15-02631a68027b	2	168981735	168982183	7	448	NA	NA	NA	2	3	2

Lymph-BNHL	1b1780d5-06da-40ee-9e15-02631a68027b	3	176913235	176913441	7	206	NA	NA	NA	5	NA	2
Lymph-BNHL	1b1780d5-06da-40ee-9e15-02631a68027b	3	187461365	187464230	30	2865	2	4	13	5	3	3
Lymph-BNHL	1b1780d5-06da-40ee-9e15-02631a68027b	3	187957842	187959776	15	1934	NA	1	NA	6	2	6
Lymph-BNHL	1b1780d5-06da-40ee-9e15-02631a68027b	5	35269716	35274061	29	4345	1	1	1	12	5	9
Lymph-BNHL	1b1780d5-06da-40ee-9e15-02631a68027b	5	62903764	62905926	8	2162	1	2	1	1	1	2
Lymph-BNHL	1b1780d5-06da-40ee-9e15-02631a68027b	6	70870652	70874193	6	3541	NA	NA	NA	3	3	NA
Lymph-BNHL	1b1780d5-06da-40ee-9e15-02631a68027b	7	40846502	40846641	6	139	NA	NA	1	3	1	1
Lymph-BNHL	1b1780d5-06da-40ee-9e15-02631a68027b	8	71217507	71217997	8	490	NA	NA	1	4	2	1
Lymph-BNHL	1b1780d5-06da-40ee-9e15-02631a68027b	11	49224297	49226582	6	2285	NA	4	1	NA	NA	1
Lymph-BNHL	1b1780d5-06da-40ee-9e15-02631a68027b	12	113493295	113495691	8	2396	NA	2	2	4	NA	NA
Lymph-BNHL	1b1780d5-06da-40ee-9e15-02631a68027b	13	46958274	46959803	12	1529	NA	2	NA	5	1	4
Lymph-BNHL	1b1780d5-06da-40ee-9e15-02631a68027b	14	106111576	106114574	15	2998	1	3	11	NA	NA	NA
Lymph-BNHL	1b1780d5-06da-40ee-9e15-02631a68027b	14	106209640	106285134	82	75494	8	24	44	3	NA	3
Lymph-BNHL	1b1780d5-06da-40ee-9e15-02631a68027b	14	106323366	106330399	148	7033	9	35	47	25	16	16
Lymph-BNHL	1b1780d5-06da-40ee-9e15-02631a68027b	14	107219069	107225251	23	6182	NA	3	2	11	4	3
Lymph-BNHL	1b1780d5-06da-40ee-9e15-02631a68027b	18	37222838	37226222	6	3384	NA	1	NA	1	2	2
Lymph-BNHL	1b1780d5-06da-40ee-9e15-02631a68027b	22	23154492	23165563	34	11071	1	6	14	7	3	3
Lymph-BNHL	1b1780d5-06da-40ee-9e15-02631a68027b	22	23223180	23248876	38	25696	3	4	16	7	6	2
Stomach-AdenoCA	1b8fe07d-859c-4d91-ade1-6b2bcb5774ce	6	53323000	53325568	12	2568	5	2	4	1	NA	NA
Stomach-AdenoCA	1b8fe07d-859c-4d91-ade1-6b2bcb5774ce	6	53422046	53426058	20	4012	5	10	5	NA	NA	NA
Stomach-AdenoCA	1b8fe07d-859c-4d91-ade1-6b2bcb5774ce	11	82754591	82758423	19	3832	8	7	4	NA	NA	NA
Eso-AdenoCa	1bb2bdb7-8a89-4e7d-903d-03a6f8018efb	8	18069417	18071928	7	2511	1	4	2	NA	NA	NA
Eso-AdenoCa	1bb2bdb7-8a89-4e7d-903d-03a6f8018efb	21	17061318	17062366	6	1048	1	2	3	NA	NA	NA
Ovary-AdenoCA	1be8fa2c-8fea-4e8c-90db-c04d9fcdbf49	1	43180718	43180864	8	146	NA	NA	8	NA	NA	NA
Ovary-AdenoCA	1be8fa2c-8fea-4e8c-90db-c04d9fcdbf49	7	96104085	96111749	13	7664	1	3	9	NA	NA	NA
Ovary-AdenoCA	1be8fa2c-8fea-4e8c-90db-c04d9fcdbf49	7	128591373	128595108	7	3735	1	1	5	NA	NA	NA
Ovary-AdenoCA	1be8fa2c-8fea-4e8c-90db-c04d9fcdbf49	8	2134658	2139130	23	4472	3	11	9	NA	NA	NA
Ovary-AdenoCA	1be8fa2c-8fea-4e8c-90db-c04d9fcdbf49	8	4458318	4460262	9	1944	1	1	7	NA	NA	NA
Ovary-AdenoCA	1be8fa2c-8fea-4e8c-90db-c04d9fcdbf49	9	139409310	139410737	12	1427	1	2	9	NA	NA	NA
Liver-HCC	1c00925b-7328-4db0-b930-04aab2d80719	8	69055300	69058031	9	2731	NA	6	3	NA	NA	NA
Liver-HCC	1c00925b-7328-4db0-b930-04aab2d80719	10	573904	604640	36	30736	4	2	7	13	7	3
Liver-HCC	1c00925b-7328-4db0-b930-04aab2d80719	10	615923	631592	19	15669	1	1	3	9	2	3
Liver-HCC	1c00925b-7328-4db0-b930-04aab2d80719	10	635719	700033	85	64314	8	6	32	25	8	6
Breast-AdenoCa	1c0e384f-7254-4afe-93c0-b3fc6c6a7894	2	76240492	76240843	6	351	1	3	2	NA	NA	NA
Breast-AdenoCa	1c0e384f-7254-4afe-93c0-b3fc6c6a7894	2	76599000	76601024	16	2024	2	7	7	NA	NA	NA
Breast-AdenoCa	1c0e384f-7254-4afe-93c0-b3fc6c6a7894	3	99148014	99156049	13	8035	NA	1	NA	3	6	3
Breast-AdenoCa	1c0e384f-7254-4afe-93c0-b3fc6c6a7894	7	65729448	65729996	7	548	2	4	1	NA	NA	NA
Breast-AdenoCa	1c0e384f-7254-4afe-93c0-b3fc6c6a7894	9	25062168	25064206	15	2038	5	9	1	NA	NA	NA
Breast-AdenoCa	1c0e384f-7254-4afe-93c0-b3fc6c6a7894	10	27730823	27735273	7	4450	1	3	3	NA	NA	NA
Breast-AdenoCa	1c0e384f-7254-4afe-93c0-b3fc6c6a7894	11	38287551	38290013	6	2462	2	2	2	NA	NA	NA
Breast-AdenoCa	1c0e384f-7254-4afe-93c0-b3fc6c6a7894	12	38873420	38874527	6	1107	1	2	3	NA	NA	NA
Breast-AdenoCa	1c0e384f-7254-4afe-93c0-b3fc6c6a7894	12	38926990	38932287	11	5297	NA	7	4	NA	NA	NA
Breast-AdenoCa	1c0e384f-7254-4afe-93c0-b3fc6c6a7894	12	131297512	131300302	7	2790	NA	4	3	NA	NA	NA
Breast-AdenoCa	1c0e384f-7254-4afe-93c0-b3fc6c6a7894	20	60489750	60491675	21	1925	1	2	18	NA	NA	NA
Bone-Leiomyo	1c188bf5-2c99-4eb4-a774-59c75d53e643	1	160124877	160127319	6	2442	1	3	2	NA	NA	NA
Bone-Leiomyo	1c188bf5-2c99-4eb4-a774-59c75d53e643	1	164968268	164973164	10	4896	2	4	4	NA	NA	NA
Bone-Leiomyo	1c188bf5-2c99-4eb4-a774-59c75d53e643	1	169365444	169368212	7	2768	1	2	4	NA	NA	NA
Bone-Leiomyo	1c188bf5-2c99-4eb4-a774-59c75d53e643	1	174499126	174502062	7	2936	1	NA	6	NA	NA	NA
Bone-Leiomyo	1c188bf5-2c99-4eb4-a774-59c75d53e643	1	176420947	176433894	17	12947	4	5	7	NA	NA	1
Bone-Leiomyo	1c188bf5-2c99-4eb4-a774-59c75d53e643	1	186209064	186215143	12	6079	3	2	6	NA	NA	1
Bone-Leiomyo	1c188bf5-2c99-4eb4-a774-59c75d53e643	1	190758615	190763139	12	4524	NA	3	9	NA	NA	NA
Bone-Leiomyo	1c188bf5-2c99-4eb4-a774-59c75d53e643	1	190929941	190942091	21	12150	1	14	6	NA	NA	NA
Bone-Leiomyo	1c188bf5-2c99-4eb4-a774-59c75d53e643	1	210133005	210133405	7	400	1	5	1	NA	NA	NA
Bone-Leiomyo	1c188bf5-2c99-4eb4-a774-59c75d53e643	1	211232305	211237449	7	5144	2	2	1	NA	NA	2
Bone-Leiomyo	1c188bf5-2c99-4eb4-a774-59c75d53e643	1	223842909	223845269	10	2360	1	5	4	NA	NA	NA
Bone-Leiomyo	1c188bf5-2c99-4eb4-a774-59c75d53e643	1	238649417	238650021	9	604	1	3	4	NA	1	NA
Bone-Leiomyo	1c188bf5-2c99-4eb4-a774-59c75d53e643	1	238711093	238725080	17	13987	5	4	8	NA	NA	NA
Bone-Leiomyo	1c188bf5-2c99-4eb4-a774-59c75d53e643	2	104274851	104279539	6	4688	1	4	1	NA	NA	NA
Bone-Leiomyo	1c188bf5-2c99-4eb4-a774-59c75d53e643	2	122314500	122318151	14	3651	1	6	7	NA	NA	NA
Bone-Leiomyo	1c188bf5-2c99-4eb4-a774-59c75d53e643	3	51135761	51138983	7	3222	2	2	3	NA	NA	NA

Bone-Leiomyo	1c188bf5-2c99-4eb4-a774-59c75d53e643	3	80818277	80823439	11	5162	NA	7	3	1	NA	NA
Bone-Leiomyo	1c188bf5-2c99-4eb4-a774-59c75d53e643	3	163218043	163223876	9	5833	2	NA	7	NA	NA	NA
Bone-Leiomyo	1c188bf5-2c99-4eb4-a774-59c75d53e643	5	53419576	53422104	11	2528	1	5	5	NA	NA	NA
Bone-Leiomyo	1c188bf5-2c99-4eb4-a774-59c75d53e643	6	99342747	99345160	6	2413	1	3	2	NA	NA	NA
Bone-Leiomyo	1c188bf5-2c99-4eb4-a774-59c75d53e643	6	101035319	101036304	8	985	NA	8	NA	NA	NA	NA
Bone-Leiomyo	1c188bf5-2c99-4eb4-a774-59c75d53e643	7	30130932	30132141	6	1209	1	4	1	NA	NA	NA
Bone-Leiomyo	1c188bf5-2c99-4eb4-a774-59c75d53e643	7	32171598	32172939	9	1341	2	4	3	NA	NA	NA
Bone-Leiomyo	1c188bf5-2c99-4eb4-a774-59c75d53e643	9	1955243	1958001	7	2758	1	5	1	NA	NA	NA
Bone-Leiomyo	1c188bf5-2c99-4eb4-a774-59c75d53e643	9	7983535	7989378	7	5843	NA	5	2	NA	NA	NA
Bone-Leiomyo	1c188bf5-2c99-4eb4-a774-59c75d53e643	9	22730602	22731420	7	818	NA	3	4	NA	NA	NA
Bone-Leiomyo	1c188bf5-2c99-4eb4-a774-59c75d53e643	11	114512117	114512857	8	740	2	3	3	NA	NA	NA
Bone-Leiomyo	1c188bf5-2c99-4eb4-a774-59c75d53e643	11	114532333	114532714	7	381	NA	4	3	NA	NA	NA
Bone-Leiomyo	1c188bf5-2c99-4eb4-a774-59c75d53e643	11	125452412	125453833	12	1421	NA	7	5	NA	NA	NA
Bone-Leiomyo	1c188bf5-2c99-4eb4-a774-59c75d53e643	12	59991922	59998172	13	6250	2	8	3	NA	NA	NA
Bone-Leiomyo	1c188bf5-2c99-4eb4-a774-59c75d53e643	12	60995841	61002255	10	6414	NA	NA	9	NA	NA	1
Bone-Leiomyo	1c188bf5-2c99-4eb4-a774-59c75d53e643	12	61905021	61908335	10	3314	NA	2	8	NA	NA	NA
Bone-Leiomyo	1c188bf5-2c99-4eb4-a774-59c75d53e643	12	66385562	66388379	13	2817	3	4	6	NA	NA	NA
Bone-Leiomyo	1c188bf5-2c99-4eb4-a774-59c75d53e643	12	67341824	67345683	7	3859	1	4	1	NA	1	NA
Bone-Leiomyo	1c188bf5-2c99-4eb4-a774-59c75d53e643	12	69160149	69161690	7	1541	NA	NA	7	NA	NA	NA
Bone-Leiomyo	1c188bf5-2c99-4eb4-a774-59c75d53e643	12	69324364	69328360	7	3996	3	NA	3	1	NA	NA
Bone-Leiomyo	1c188bf5-2c99-4eb4-a774-59c75d53e643	12	69845209	69850028	7	4819	2	2	2	NA	NA	1
Bone-Leiomyo	1c188bf5-2c99-4eb4-a774-59c75d53e643	12	74987014	74988706	7	1692	1	5	NA	NA	NA	1
Bone-Leiomyo	1c188bf5-2c99-4eb4-a774-59c75d53e643	12	76509163	76514018	12	4855	1	5	6	NA	NA	NA
Bone-Leiomyo	1c188bf5-2c99-4eb4-a774-59c75d53e643	13	84194035	84196241	15	2206	4	11	NA	NA	NA	NA
Bone-Leiomyo	1c188bf5-2c99-4eb4-a774-59c75d53e643	13	85044130	85048580	26	4450	4	14	8	NA	NA	NA
Bone-Leiomyo	1c188bf5-2c99-4eb4-a774-59c75d53e643	14	73467267	73468121	6	854	NA	2	4	NA	NA	NA
Bone-Leiomyo	1c188bf5-2c99-4eb4-a774-59c75d53e643	14	83010367	83011600	8	1233	2	3	3	NA	NA	NA
Bone-Leiomyo	1c188bf5-2c99-4eb4-a774-59c75d53e643	14	99256967	99259908	15	2941	3	7	5	NA	NA	NA
Bone-Leiomyo	1c188bf5-2c99-4eb4-a774-59c75d53e643	14	99726175	99726899	7	724	1	2	4	NA	NA	NA
Bone-Leiomyo	1c188bf5-2c99-4eb4-a774-59c75d53e643	15	31125465	31128556	11	3091	2	4	5	NA	NA	NA
Bone-Leiomyo	1c188bf5-2c99-4eb4-a774-59c75d53e643	15	96524291	96531890	11	7599	NA	6	5	NA	NA	NA
Bone-Leiomyo	1c188bf5-2c99-4eb4-a774-59c75d53e643	X	68129205	68131134	9	1929	NA	6	3	NA	NA	NA
Panc-AdenoCA	1c28e44a-6e6c-44ed-b58a-e3262c0e6759	1	48398000	48399022	6	1022	NA	1	5	NA	NA	NA
Panc-AdenoCA	1c28e44a-6e6c-44ed-b58a-e3262c0e6759	2	230905494	230905978	14	484	2	4	8	NA	NA	NA
Panc-AdenoCA	1c28e44a-6e6c-44ed-b58a-e3262c0e6759	3	22269979	22270356	6	377	NA	1	5	NA	NA	NA
Panc-AdenoCA	1c28e44a-6e6c-44ed-b58a-e3262c0e6759	3	31053637	31054227	15	590	3	3	9	NA	NA	NA
Panc-AdenoCA	1c28e44a-6e6c-44ed-b58a-e3262c0e6759	3	31862750	31863739	10	989	2	4	4	NA	NA	NA
Panc-AdenoCA	1c28e44a-6e6c-44ed-b58a-e3262c0e6759	3	146986466	146988338	11	1872	2	5	4	NA	NA	NA
Panc-AdenoCA	1c28e44a-6e6c-44ed-b58a-e3262c0e6759	3	147937362	147940145	17	2783	1	6	10	NA	NA	NA
Panc-AdenoCA	1c28e44a-6e6c-44ed-b58a-e3262c0e6759	4	160441134	160441363	6	229	NA	2	4	NA	NA	NA
Panc-AdenoCA	1c28e44a-6e6c-44ed-b58a-e3262c0e6759	6	167557404	167560822	17	3418	2	7	8	NA	NA	NA
Panc-AdenoCA	1c28e44a-6e6c-44ed-b58a-e3262c0e6759	9	21980476	21980564	6	88	NA	NA	6	NA	NA	NA
Panc-AdenoCA	1c28e44a-6e6c-44ed-b58a-e3262c0e6759	11	107654277	107654686	7	409	1	3	3	NA	NA	NA
Panc-AdenoCA	1c28e44a-6e6c-44ed-b58a-e3262c0e6759	13	20297547	20303092	13	5545	4	3	6	NA	NA	NA
Panc-AdenoCA	1c28e44a-6e6c-44ed-b58a-e3262c0e6759	13	21080983	21082549	15	1566	1	2	12	NA	NA	NA
Panc-AdenoCA	1c28e44a-6e6c-44ed-b58a-e3262c0e6759	13	22015383	22016270	15	887	2	2	11	NA	NA	NA
Panc-AdenoCA	1c28e44a-6e6c-44ed-b58a-e3262c0e6759	13	22328920	22331949	9	3029	3	2	4	NA	NA	NA
Panc-AdenoCA	1c28e44a-6e6c-44ed-b58a-e3262c0e6759	13	23527754	23528879	7	1125	NA	1	6	NA	NA	NA
Panc-AdenoCA	1c9fbe26-1447-4335-aaab-06d6bb3e2741	17	20982569	20982790	6	221	NA	3	2	NA	1	NA
Panc-AdenoCA	1c9fbe26-1447-4335-aaab-06d6bb3e2741	18	54306481	54306727	9	246	1	4	4	NA	NA	NA
Skin-Melanoma	1cd0acf2-3116-4dfa-a063-0a435b9f6da3	6	9541002	9541912	18	910	3	7	8	NA	NA	NA
Skin-Melanoma	1cd0acf2-3116-4dfa-a063-0a435b9f6da3	6	62560699	62561314	7	615	2	4	1	NA	NA	NA
Skin-Melanoma	1cd0acf2-3116-4dfa-a063-0a435b9f6da3	6	63045614	63049408	7	3794	2	4	1	NA	NA	NA
Skin-Melanoma	1cd0acf2-3116-4dfa-a063-0a435b9f6da3	6	63158642	63161002	9	2360	NA	8	1	NA	NA	NA
Skin-Melanoma	1cd0acf2-3116-4dfa-a063-0a435b9f6da3	6	63190274	63190517	13	243	NA	NA	13	NA	NA	NA
Skin-Melanoma	1cd0acf2-3116-4dfa-a063-0a435b9f6da3	6	64379391	64384167	17	4776	5	4	7	NA	1	NA
Skin-Melanoma	1cd0acf2-3116-4dfa-a063-0a435b9f6da3	11	11250833	11256878	11	6045	1	7	2	NA	NA	1
Skin-Melanoma	1cd0acf2-3116-4dfa-a063-0a435b9f6da3	11	49214646	49216615	11	1969	2	2	6	NA	1	NA
Skin-Melanoma	1cd0acf2-3116-4dfa-a063-0a435b9f6da3	11	68432923	68434654	7	1731	NA	6	NA	NA	NA	1
Skin-Melanoma	1cd0acf2-3116-4dfa-a063-0a435b9f6da3	11	73626621	73627202	8	581	4	2	2	NA	NA	NA

Skin-Melanoma	1cd0acf2-3116-4dfa-a063-0a435b9f6da3	11	73739696	73740580	11	884	2	6	3	NA	NA	NA
Skin-Melanoma	1cd0acf2-3116-4dfa-a063-0a435b9f6da3	11	73927258	73927523	20	265	NA	NA	20	NA	NA	NA
Skin-Melanoma	1cd0acf2-3116-4dfa-a063-0a435b9f6da3	11	77754585	77761395	11	6810	1	3	6	NA	NA	1
Skin-Melanoma	1cd0acf2-3116-4dfa-a063-0a435b9f6da3	11	79882471	79892628	15	10157	7	5	1	1	NA	1
Lymph-BNHL	1d1eeba2-4d7b-4380-b0b6-26d246bc4158	2	89155934	89161386	45	5452	4	5	11	17	3	5
Lymph-BNHL	1d1eeba2-4d7b-4380-b0b6-26d246bc4158	5	161805143	161808750	8	3607	NA	1	1	2	2	2
Lymph-BNHL	1d1eeba2-4d7b-4380-b0b6-26d246bc4158	8	77552637	77556890	9	4253	2	1	3	1	NA	2
Lymph-BNHL	1d1eeba2-4d7b-4380-b0b6-26d246bc4158	14	106210944	106213627	7	2683	NA	1	5	1	NA	NA
Lymph-BNHL	1d1eeba2-4d7b-4380-b0b6-26d246bc4158	14	106324037	106330348	87	6311	9	22	31	13	6	6
Lymph-BNHL	1d1eeba2-4d7b-4380-b0b6-26d246bc4158	14	106518576	106519272	14	696	NA	1	6	6	NA	1
Lymph-BNHL	1d1eeba2-4d7b-4380-b0b6-26d246bc4158	18	60983484	60987041	24	3557	2	4	11	4	1	2
Lymph-BNHL	1d1eeba2-4d7b-4380-b0b6-26d246bc4158	22	23230262	23231920	16	1658	NA	2	4	7	NA	3
Stomach-AdenoCA	1d2d355a-8409-4c8a-9f07-ac62885e228d	18	23245488	23250323	6	4835	2	1	3	NA	NA	NA
Stomach-AdenoCA	1d2d355a-8409-4c8a-9f07-ac62885e228d	18	25471050	25484364	25	13314	7	10	7	NA	1	NA
Kidney-RCC	1d325d84-a3a0-4802-9842-76cb21cecb1c	3	55941787	55948123	17	6336	3	4	10	NA	NA	NA
Head-SCC	1db83e80-bdf7-4d8d-8008-bd8406d641fc	1	68748853	68750425	6	1572	1	NA	5	NA	NA	NA
Head-SCC	1db83e80-bdf7-4d8d-8008-bd8406d641fc	1	168659505	168662149	8	2644	1	NA	7	NA	NA	NA
Head-SCC	1db83e80-bdf7-4d8d-8008-bd8406d641fc	1	195404732	195406839	11	2107	1	3	7	NA	NA	NA
Head-SCC	1db83e80-bdf7-4d8d-8008-bd8406d641fc	3	171243867	171246660	6	2793	1	2	3	NA	NA	NA
Head-SCC	1db83e80-bdf7-4d8d-8008-bd8406d641fc	15	66642609	66645887	7	3278	3	2	2	NA	NA	NA
Head-SCC	1db83e80-bdf7-4d8d-8008-bd8406d641fc	22	38898691	38901756	10	3065	2	2	6	NA	NA	NA
Liver-HCC	1dbdbb2c-c623-11e3-bf01-24c6515278c0	1	160318755	160319200	7	445	3	NA	4	NA	NA	NA
Liver-HCC	1dbdbb2c-c623-11e3-bf01-24c6515278c0	11	69174446	69185524	29	11078	2	12	15	NA	NA	NA
Ovary-AdenoCA	1dc9e7fd-fc62-4b32-9619-4e02a266a385	6	86245127	86246666	9	1539	1	5	3	NA	NA	NA
Ovary-AdenoCA	1dc9e7fd-fc62-4b32-9619-4e02a266a385	12	125097652	125098851	6	1199	3	3	NA	NA	NA	NA
Panc-AdenoCA	1e181878-c640-4e91-a620-3fc4b08a4de1	12	28087899	28088981	13	1082	4	6	3	NA	NA	NA
CNS-GBM	1e27cc8a-5394-4958-9af6-5ece1fe24516	4	165742721	165742857	7	136	NA	NA	7	NA	NA	NA
CNS-GBM	1e27cc8a-5394-4958-9af6-5ece1fe24516	9	34972546	34973360	6	814	NA	4	2	NA	NA	NA
CNS-GBM	1e27cc8a-5394-4958-9af6-5ece1fe24516	9	36098292	36105250	13	6958	5	4	4	NA	NA	NA
Biliary-AdenoCA	1e2dcbcc-771c-43c5-8c8d-e0eb77cb3494	1	153038007	153038689	10	682	2	1	7	NA	NA	NA
Biliary-AdenoCA	1e2dcbcc-771c-43c5-8c8d-e0eb77cb3494	1	158641139	158642139	13	1000	2	5	6	NA	NA	NA
Biliary-AdenoCA	1e2dcbcc-771c-43c5-8c8d-e0eb77cb3494	1	164074892	164077803	8	2911	2	2	4	NA	NA	NA
Biliary-AdenoCA	1e2dcbcc-771c-43c5-8c8d-e0eb77cb3494	1	166483278	166483528	9	250	NA	NA	9	NA	NA	NA
Biliary-AdenoCA	1e2dcbcc-771c-43c5-8c8d-e0eb77cb3494	1	175651191	175652054	13	863	NA	NA	13	NA	NA	NA
Biliary-AdenoCA	1e2dcbcc-771c-43c5-8c8d-e0eb77cb3494	1	182430135	182433859	7	3724	NA	5	2	NA	NA	NA
Biliary-AdenoCA	1e2dcbcc-771c-43c5-8c8d-e0eb77cb3494	1	187844393	187845371	16	978	NA	NA	16	NA	NA	NA
Biliary-AdenoCA	1e2dcbcc-771c-43c5-8c8d-e0eb77cb3494	1	190206418	190209014	9	2596	2	4	3	NA	NA	NA
Biliary-AdenoCA	1e2dcbcc-771c-43c5-8c8d-e0eb77cb3494	1	190236587	190241590	8	5003	2	NA	6	NA	NA	NA
Biliary-AdenoCA	1e2dcbcc-771c-43c5-8c8d-e0eb77cb3494	1	192326278	192326955	8	677	NA	3	5	NA	NA	NA
Biliary-AdenoCA	1e2dcbcc-771c-43c5-8c8d-e0eb77cb3494	1	194466406	194467607	10	1201	NA	6	4	NA	NA	NA
Biliary-AdenoCA	1e2dcbcc-771c-43c5-8c8d-e0eb77cb3494	1	198783223	198785703	14	2480	3	1	9	NA	1	NA
Biliary-AdenoCA	1e2dcbcc-771c-43c5-8c8d-e0eb77cb3494	1	210579659	210580163	8	504	NA	NA	8	NA	NA	NA
Biliary-AdenoCA	1e2dcbcc-771c-43c5-8c8d-e0eb77cb3494	1	231304139	231305046	17	907	NA	NA	17	NA	NA	NA
Biliary-AdenoCA	1e2dcbcc-771c-43c5-8c8d-e0eb77cb3494	1	231594819	231595118	8	299	NA	NA	8	NA	NA	NA
Biliary-AdenoCA	1e2dcbcc-771c-43c5-8c8d-e0eb77cb3494	3	87811566	87814569	6	3003	NA	NA	NA	1	2	3
Biliary-AdenoCA	1e2dcbcc-771c-43c5-8c8d-e0eb77cb3494	7	70537864	70543244	7	5380	1	4	1	NA	1	NA
Biliary-AdenoCA	1e2dcbcc-771c-43c5-8c8d-e0eb77cb3494	8	96973261	96989251	17	15990	NA	NA	NA	4	8	5
Biliary-AdenoCA	1e2dcbcc-771c-43c5-8c8d-e0eb77cb3494	9	25372544	25379105	13	6561	NA	3	10	NA	NA	NA
Liver-HCC	1ea7e01c-c623-11e3-bf01-24c6515278c0	2	44696346	44696529	6	183	NA	NA	NA	2	3	1
Panc-Endocrine	1eb1bc62-18f6-4bcb-a16d-aa6a91914031	20	36649979	36654939	8	4960	NA	NA	8	NA	NA	NA
Prost-AdenoCA	1eb37b28-fac2-477a-88b3-e04291a07926	4	121239423	121244818	16	5395	1	13	2	NA	NA	NA
Prost-AdenoCA	1eb37b28-fac2-477a-88b3-e04291a07926	8	42909040	42911292	6	2252	1	3	2	NA	NA	NA
Breast-AdenoCa	1eb62abc-7928-405b-84cc-f091ca5347b2	2	209282527	209285572	6	3045	2	1	3	NA	NA	NA
Breast-AdenoCa	1eb62abc-7928-405b-84cc-f091ca5347b2	8	39710904	39713957	11	3053	2	6	3	NA	NA	NA
Breast-AdenoCa	1eb62abc-7928-405b-84cc-f091ca5347b2	12	7813677	7814125	6	448	NA	5	1	NA	NA	NA
Breast-AdenoCa	1eb62abc-7928-405b-84cc-f091ca5347b2	12	34336953	34337871	13	918	5	7	NA	NA	1	NA
Stomach-AdenoCA	1f2a8889-4374-4037-bb56-0f20733380e3	7	10275489	10275648	6	159	2	1	3	NA	NA	NA
Stomach-AdenoCA	1f2a8889-4374-4037-bb56-0f20733380e3	7	82923546	82923869	10	323	1	1	8	NA	NA	NA
Stomach-AdenoCA	1f2a8889-4374-4037-bb56-0f20733380e3	7	85563238	85567359	12	4121	7	3	2	NA	NA	NA
Stomach-AdenoCA	1f2a8889-4374-4037-bb56-0f20733380e3	12	9764032	9764078	6	46	1	1	2	1	NA	1

Stomach-AdenoCA	1f2a8889-4374-4037-bb56-0f20733380e3	19	30738991	30744644	9	5653	2	3	4	NA	NA	NA
Panc-AdenoCA	1f81a1b0-8089-44ef-87b5-b3359a407ad2	8	130569744	130573141	8	3397	1	4	3	NA	NA	NA
Panc-AdenoCA	1f81a1b0-8089-44ef-87b5-b3359a407ad2	10	64730490	64731134	10	644	NA	8	2	NA	NA	NA
Panc-AdenoCA	1f81a1b0-8089-44ef-87b5-b3359a407ad2	17	1911636	1913722	7	2086	3	2	2	NA	NA	NA
Panc-AdenoCA	1f81a1b0-8089-44ef-87b5-b3359a407ad2	18	718448	720805	17	2357	4	10	3	NA	NA	NA
Panc-AdenoCA	1f81a1b0-8089-44ef-87b5-b3359a407ad2	18	19938732	19940585	11	1853	2	6	3	NA	NA	NA
Panc-AdenoCA	1f81a1b0-8089-44ef-87b5-b3359a407ad2	19	933044	936971	10	3927	2	4	4	NA	NA	NA
Panc-AdenoCA	1f81a1b0-8089-44ef-87b5-b3359a407ad2	19	27925996	27929855	18	3859	3	8	7	NA	NA	NA
Panc-AdenoCA	1f81a1b0-8089-44ef-87b5-b3359a407ad2	19	40354056	40355243	8	1187	2	3	3	NA	NA	NA
Panc-AdenoCA	1f81a1b0-8089-44ef-87b5-b3359a407ad2	20	11643025	11643994	6	969	NA	4	2	NA	NA	NA
Panc-AdenoCA	1f81a1b0-8089-44ef-87b5-b3359a407ad2	20	23430916	23431978	10	1062	2	3	5	NA	NA	NA
Panc-AdenoCA	1f81a1b0-8089-44ef-87b5-b3359a407ad2	22	34357274	34357775	7	501	1	3	3	NA	NA	NA
Eso-AdenoCa	1f967003-82d6-4932-a445-24fc25723594	18	30254757	30255262	10	505	5	NA	5	NA	NA	NA
Head-SCC	1fdab6a6-7346-4229-aa8d-5dbd258f0c60	3	48830662	48833068	7	2406	1	6	NA	NA	NA	NA
Head-SCC	1fdab6a6-7346-4229-aa8d-5dbd258f0c60	7	133393493	133395204	6	1711	NA	4	2	NA	NA	NA
Head-SCC	1fdab6a6-7346-4229-aa8d-5dbd258f0c60	11	66558179	66559034	7	855	2	2	3	NA	NA	NA
Head-SCC	1fdab6a6-7346-4229-aa8d-5dbd258f0c60	16	67560146	67563864	9	3718	NA	2	7	NA	NA	NA
Head-SCC	1ff155dd-deb4-44e0-b3a2-e4875b618435	4	189181388	189183536	6	2148	NA	1	5	NA	NA	NA
Head-SCC	1ff155dd-deb4-44e0-b3a2-e4875b618435	7	107386353	107387803	8	1450	NA	4	4	NA	NA	NA
Head-SCC	1ff155dd-deb4-44e0-b3a2-e4875b618435	12	104245186	104246939	7	1753	NA	4	2	1	NA	NA
Head-SCC	1ff155dd-deb4-44e0-b3a2-e4875b618435	19	11313476	11321875	11	8399	2	3	6	NA	NA	NA
Head-SCC	1ff155dd-deb4-44e0-b3a2-e4875b618435	X	48779868	48782034	7	2166	1	1	5	NA	NA	NA
Head-SCC	1fff8b62-534b-4d71-a65f-e5f93b8b50ed	6	3217996	3218519	7	523	3	2	2	NA	NA	NA
Head-SCC	1fff8b62-534b-4d71-a65f-e5f93b8b50ed	X	151690187	151692324	16	2137	2	12	2	NA	NA	NA
Breast-AdenoCa	207f8a42-5b05-4876-b0ae-ebfaeea27844	3	89964193	89965074	7	881	5	2	NA	NA	NA	NA
Skin-Melanoma	20e02396-e676-412d-9724-44a428919cdb	6	66959607	66963344	14	3737	2	3	7	1	NA	1
Skin-Melanoma	20e02396-e676-412d-9724-44a428919cdb	6	67400609	67403339	9	2730	NA	3	6	NA	NA	NA
Skin-Melanoma	20e02396-e676-412d-9724-44a428919cdb	6	69145082	69152154	16	7072	1	9	6	NA	NA	NA
Skin-Melanoma	20e02396-e676-412d-9724-44a428919cdb	11	73563517	73564108	11	591	1	6	4	NA	NA	NA
Skin-Melanoma	20e02396-e676-412d-9724-44a428919cdb	11	78735063	78735670	7	607	NA	2	5	NA	NA	NA
Skin-Melanoma	20e02396-e676-412d-9724-44a428919cdb	11	81788836	81793457	7	4621	2	3	2	NA	NA	NA
Skin-Melanoma	20e02396-e676-412d-9724-44a428919cdb	14	78293212	78293473	8	261	1	NA	7	NA	NA	NA
Skin-Melanoma	20e02396-e676-412d-9724-44a428919cdb	21	22324303	22324874	7	571	2	5	NA	NA	NA	NA
Skin-Melanoma	20e02396-e676-412d-9724-44a428919cdb	22	37910034	37910741	6	707	NA	3	3	NA	NA	NA
Panc-AdenoCA	2102558b-87b0-447b-bb9a-e4ae9df8b75e	22	27851816	27852663	13	847	NA	2	9	1	NA	1
Biliary-AdenoCA	2109e1d4-c623-11e3-bf01-24c6515278c0	7	115380308	115384027	11	3719	1	4	6	NA	NA	NA
Biliary-AdenoCA	2109e1d4-c623-11e3-bf01-24c6515278c0	7	122485272	122490493	12	5221	NA	2	10	NA	NA	NA
Biliary-AdenoCA	2109e1d4-c623-11e3-bf01-24c6515278c0	20	21614353	21615869	6	1516	NA	2	4	NA	NA	NA
Panc-AdenoCA	21b1a37f-e91e-4ce1-83cf-bb89b8d0fb35	13	78962732	78965760	6	3028	1	1	4	NA	NA	NA
Panc-AdenoCA	21b1a37f-e91e-4ce1-83cf-bb89b8d0fb35	15	37052347	37052938	6	591	1	1	4	NA	NA	NA
Panc-AdenoCA	21b1a37f-e91e-4ce1-83cf-bb89b8d0fb35	15	37091883	37092219	8	336	1	5	2	NA	NA	NA
Panc-AdenoCA	21b1a37f-e91e-4ce1-83cf-bb89b8d0fb35	15	39549309	39553726	11	4417	NA	1	10	NA	NA	NA
Panc-AdenoCA	228fb827-c05e-494c-8a21-e1d925e100cb	17	70940219	70941932	12	1713	3	2	6	NA	NA	1
Breast-AdenoCa	2290b078-6a5b-4c83-9dfb-b525bbf14e4e	1	53793075	53802580	21	9505	5	14	2	NA	NA	NA
Breast-AdenoCa	2290b078-6a5b-4c83-9dfb-b525bbf14e4e	1	160672688	160673692	10	1004	1	8	1	NA	NA	NA
Breast-AdenoCa	2290b078-6a5b-4c83-9dfb-b525bbf14e4e	1	160710319	160710660	10	341	3	4	2	NA	NA	1
Breast-AdenoCa	2290b078-6a5b-4c83-9dfb-b525bbf14e4e	1	165658600	165659039	7	439	NA	5	2	NA	NA	NA
Breast-AdenoCa	2290b078-6a5b-4c83-9dfb-b525bbf14e4e	8	135606835	135608459	17	1624	4	11	2	NA	NA	NA
Breast-AdenoCa	2290b078-6a5b-4c83-9dfb-b525bbf14e4e	10	69226699	69232327	15	5628	6	7	2	NA	NA	NA
Breast-AdenoCa	2290b078-6a5b-4c83-9dfb-b525bbf14e4e	15	100503132	100503149	8	17	1	2	NA	NA	1	4
Breast-AdenoCa	2290b078-6a5b-4c83-9dfb-b525bbf14e4e	21	25834654	25835101	6	447	NA	4	2	NA	NA	NA
Breast-AdenoCa	2290b078-6a5b-4c83-9dfb-b525bbf14e4e	X	133744907	133745526	6	619	1	2	3	NA	NA	NA
Lymph-BNHL	232b7754-b3c3-4530-9d24-3af40d9b2816	2	89158235	89174793	63	16558	1	8	15	25	11	3
Lymph-BNHL	232b7754-b3c3-4530-9d24-3af40d9b2816	6	140288032	140292923	6	4891	NA	NA	2	2	1	1
Lymph-BNHL	232b7754-b3c3-4530-9d24-3af40d9b2816	8	16073579	16075862	10	2283	NA	NA	NA	5	2	3
Lymph-BNHL	232b7754-b3c3-4530-9d24-3af40d9b2816	14	106112424	106114289	9	1865	2	1	6	NA	NA	NA
Lymph-BNHL	232b7754-b3c3-4530-9d24-3af40d9b2816	14	106208882	106213681	17	4799	2	3	12	NA	NA	NA
Lymph-BNHL	232b7754-b3c3-4530-9d24-3af40d9b2816	14	106239583	106241323	12	1740	NA	1	11	NA	NA	NA
Lymph-BNHL	232b7754-b3c3-4530-9d24-3af40d9b2816	14	106324134	106330080	42	5946	1	8	17	7	2	7
Lymph-BNHL	232b7754-b3c3-4530-9d24-3af40d9b2816	18	60983993	60986731	10	2738	2	NA	2	4	NA	2

Lymph-BNHL	232b7754-b3c3-4530-9d24-3af40d9b2816	22	23223355	23227934	6	4579	NA	4	2	NA	NA	NA
Lymph-BNHL	232b7754-b3c3-4530-9d24-3af40d9b2816	22	23230312	23232527	16	2215	1	3	5	1	1	5
Lung-AdenoCA	23d86395-9e20-4cf9-bf7e-f76544a1f772	5	1172261	1173714	6	1453	NA	NA	6	NA	NA	NA
Lung-AdenoCA	23d86395-9e20-4cf9-bf7e-f76544a1f772	5	15279764	15280610	8	846	NA	NA	8	NA	NA	NA
Lung-AdenoCA	23d86395-9e20-4cf9-bf7e-f76544a1f772	8	79877701	79878368	6	667	NA	3	3	NA	NA	NA
Lung-AdenoCA	23d86395-9e20-4cf9-bf7e-f76544a1f772	8	82208407	82208885	11	478	1	4	5	1	NA	NA
Lung-AdenoCA	23d86395-9e20-4cf9-bf7e-f76544a1f772	8	93354742	93357578	7	2836	NA	NA	6	1	NA	NA
Lung-AdenoCA	23d86395-9e20-4cf9-bf7e-f76544a1f772	9	33251270	33252802	7	1532	1	3	3	NA	NA	NA
Lung-AdenoCA	23d86395-9e20-4cf9-bf7e-f76544a1f772	14	106657126	106657610	12	484	NA	4	8	NA	NA	NA
Lung-AdenoCA	23d86395-9e20-4cf9-bf7e-f76544a1f772	16	16065689	16065928	9	239	NA	3	5	NA	1	NA
Lung-AdenoCA	23d86395-9e20-4cf9-bf7e-f76544a1f772	16	90037377	90039329	8	1952	NA	4	4	NA	NA	NA
Lung-AdenoCA	23d86395-9e20-4cf9-bf7e-f76544a1f772	17	79786759	79793422	8	6663	NA	NA	8	NA	NA	NA
Panc-AdenoCA	241abdfd-e6de-4830-a233-4bbc5f622725	3	149887404	149891074	9	3670	NA	3	5	NA	1	NA
Panc-AdenoCA	241abdfd-e6de-4830-a233-4bbc5f622725	6	90035225	90035881	10	656	NA	NA	10	NA	NA	NA
Lymph-BNHL	2439ec74-1713-4d47-a5d9-886f69dddcb6	2	179155806	179160781	6	4975	NA	NA	1	3	2	NA
Lymph-BNHL	2439ec74-1713-4d47-a5d9-886f69dddcb6	3	186740074	186740346	6	272	1	NA	NA	3	NA	2
Lymph-BNHL	2439ec74-1713-4d47-a5d9-886f69dddcb6	3	188471213	188471754	8	541	NA	1	1	2	2	2
Lymph-BNHL	2439ec74-1713-4d47-a5d9-886f69dddcb6	4	40197647	40200777	6	3130	NA	1	1	4	NA	NA
Lymph-BNHL	2439ec74-1713-4d47-a5d9-886f69dddcb6	4	63573207	63575076	7	1869	NA	NA	NA	5	1	1
Lymph-BNHL	2439ec74-1713-4d47-a5d9-886f69dddcb6	5	170337487	170342847	7	5360	1	NA	NA	5	NA	1
Lymph-BNHL	2439ec74-1713-4d47-a5d9-886f69dddcb6	6	48411056	48412589	6	1533	1	1	NA	2	1	1
Lymph-BNHL	2439ec74-1713-4d47-a5d9-886f69dddcb6	9	37382736	37385020	10	2284	2	2	4	NA	1	1
Lymph-BNHL	2439ec74-1713-4d47-a5d9-886f69dddcb6	11	29187581	29190989	6	3408	NA	NA	NA	2	1	3
Lymph-BNHL	2439ec74-1713-4d47-a5d9-886f69dddcb6	14	106209481	106214196	14	4715	2	2	9	NA	NA	1
Lymph-BNHL	2439ec74-1713-4d47-a5d9-886f69dddcb6	14	106238814	106242016	16	3202	NA	3	11	NA	2	NA
Lymph-BNHL	2439ec74-1713-4d47-a5d9-886f69dddcb6	14	106323274	106329551	64	6277	NA	14	19	5	16	10
Lymph-BNHL	2439ec74-1713-4d47-a5d9-886f69dddcb6	14	106830038	106833016	37	2978	2	4	8	5	11	7
Lymph-BNHL	2439ec74-1713-4d47-a5d9-886f69dddcb6	16	77890152	77895625	7	5473	NA	1	2	2	1	1
Lymph-BNHL	2439ec74-1713-4d47-a5d9-886f69dddcb6	19	38266221	38266575	6	354	NA	NA	NA	NA	3	3
Lymph-BNHL	2439ec74-1713-4d47-a5d9-886f69dddcb6	22	22379511	22385479	39	5968	1	7	13	10	6	2
Lymph-BNHL	2439ec74-1713-4d47-a5d9-886f69dddcb6	22	22758862	22764351	18	5489	2	4	7	3	2	NA
Lymph-BNHL	2439ec74-1713-4d47-a5d9-886f69dddcb6	X	12993511	12994814	16	1303	1	3	6	4	NA	2
Liver-HCC	2468e58c-c623-11e3-bf01-24c6515278c0	3	188555540	188556847	9	1307	1	6	2	NA	NA	NA
Lung-SCC	24badf07-8615-48a3-8d6e-45c3d783f18d	3	163518112	163518602	7	490	NA	2	5	NA	NA	NA
Lung-SCC	24badf07-8615-48a3-8d6e-45c3d783f18d	3	172174490	172175951	7	1461	NA	3	4	NA	NA	NA
Lung-SCC	24badf07-8615-48a3-8d6e-45c3d783f18d	3	186932563	186936040	21	3477	2	4	15	NA	NA	NA
Lung-SCC	24badf07-8615-48a3-8d6e-45c3d783f18d	X	24285929	24292169	15	6240	3	3	9	NA	NA	NA
Kidney-RCC	252a1c43-f954-44d7-8e31-6bcd0157a05c	5	96739488	96741269	12	1781	3	3	6	NA	NA	NA
Panc-AdenoCA	2564a262-03e8-467b-9ea2-f3ab38d75ae1	6	51170042	51171143	8	1101	2	2	4	NA	NA	NA
Panc-AdenoCA	2564a262-03e8-467b-9ea2-f3ab38d75ae1	14	21664031	21664283	7	252	NA	2	5	NA	NA	NA
Panc-AdenoCA	2564a262-03e8-467b-9ea2-f3ab38d75ae1	14	28539328	28541946	23	2618	6	8	9	NA	NA	NA
Panc-AdenoCA	2564a262-03e8-467b-9ea2-f3ab38d75ae1	14	54349030	54351229	14	2199	3	7	4	NA	NA	NA
Panc-AdenoCA	2564a262-03e8-467b-9ea2-f3ab38d75ae1	14	79837471	79839253	14	1782	1	2	11	NA	NA	NA
Panc-AdenoCA	2564a262-03e8-467b-9ea2-f3ab38d75ae1	14	93722951	93723374	7	423	2	NA	5	NA	NA	NA
Biliary-AdenoCA	2584b428-c623-11e3-bf01-24c6515278c0	8	135067371	135068678	11	1307	NA	7	4	NA	NA	NA
Biliary-AdenoCA	2584b428-c623-11e3-bf01-24c6515278c0	9	6761514	6762368	9	854	NA	1	8	NA	NA	NA
Biliary-AdenoCA	2584b428-c623-11e3-bf01-24c6515278c0	11	91626461	91626897	7	436	NA	6	1	NA	NA	NA
Biliary-AdenoCA	2584b428-c623-11e3-bf01-24c6515278c0	X	44832175	44832380	6	205	NA	NA	6	NA	NA	NA
Biliary-AdenoCA	2584b428-c623-11e3-bf01-24c6515278c0	X	49506146	49506714	7	568	NA	1	6	NA	NA	NA
Panc-AdenoCA	25c32aca-6738-43ef-a103-243f2f9a3b43	2	32678394	32681685	9	3291	3	3	3	NA	NA	NA
Panc-AdenoCA	25c32aca-6738-43ef-a103-243f2f9a3b43	2	122837360	122842266	14	4906	2	6	5	NA	NA	1
Panc-AdenoCA	25c32aca-6738-43ef-a103-243f2f9a3b43	4	59193778	59198233	9	4455	2	3	4	NA	NA	NA
Panc-AdenoCA	25c32aca-6738-43ef-a103-243f2f9a3b43	7	31888517	31893182	9	4665	3	2	4	NA	NA	NA
Panc-AdenoCA	25c32aca-6738-43ef-a103-243f2f9a3b43	9	21883988	21884641	12	653	2	2	8	NA	NA	NA
Panc-AdenoCA	25c32aca-6738-43ef-a103-243f2f9a3b43	10	131744355	131747800	25	3445	NA	9	16	NA	NA	NA
Panc-AdenoCA	25c32aca-6738-43ef-a103-243f2f9a3b43	13	94503967	94504418	7	451	1	3	3	NA	NA	NA
Panc-AdenoCA	25c32aca-6738-43ef-a103-243f2f9a3b43	14	48284100	48285059	6	959	2	3	1	NA	NA	NA
Panc-AdenoCA	25c32aca-6738-43ef-a103-243f2f9a3b43	18	69588077	69592216	42	4139	1	13	28	NA	NA	NA
Panc-AdenoCA	25c32aca-6738-43ef-a103-243f2f9a3b43	X	81289180	81291520	8	2340	NA	7	1	NA	NA	NA
Breast-AdenoCa	25c76a8f-77c0-4650-bddf-45ed0c10a2e6	8	109924152	109925280	13	1128	3	7	3	NA	NA	NA

Eso-AdenoCa	25c99312-bf63-4654-a73a-03c3c1cd50b6	8	69467685	69470997	8	3312	NA	NA	NA	2	4	2
Panc-AdenoCA	25cf7449-8c5b-403e-aa75-e2af158598e8	1	218141107	218142371	7	1264	NA	6	1	NA	NA	NA
Panc-AdenoCA	25cf7449-8c5b-403e-aa75-e2af158598e8	3	16196439	16199328	14	2889	1	7	6	NA	NA	NA
Panc-AdenoCA	25cf7449-8c5b-403e-aa75-e2af158598e8	3	49757131	49758015	9	884	NA	2	6	NA	1	NA
Panc-AdenoCA	25cf7449-8c5b-403e-aa75-e2af158598e8	3	156305514	156308405	15	2891	1	7	7	NA	NA	NA
Panc-AdenoCA	25cf7449-8c5b-403e-aa75-e2af158598e8	4	92475643	92476655	9	1012	NA	NA	NA	2	5	2
Panc-AdenoCA	25cf7449-8c5b-403e-aa75-e2af158598e8	5	78451829	78455031	11	3202	2	5	4	NA	NA	NA
Panc-AdenoCA	25cf7449-8c5b-403e-aa75-e2af158598e8	6	4918361	4920125	6	1764	NA	2	4	NA	NA	NA
Panc-AdenoCA	25cf7449-8c5b-403e-aa75-e2af158598e8	8	17045249	17047116	7	1867	1	4	2	NA	NA	NA
Panc-AdenoCA	25cf7449-8c5b-403e-aa75-e2af158598e8	8	27817855	27819594	7	1739	2	3	2	NA	NA	NA
Panc-AdenoCA	25cf7449-8c5b-403e-aa75-e2af158598e8	8	80262081	80262664	10	583	2	5	3	NA	NA	NA
Panc-AdenoCA	25cf7449-8c5b-403e-aa75-e2af158598e8	10	8042913	8043833	8	920	NA	6	2	NA	NA	NA
Panc-AdenoCA	25cf7449-8c5b-403e-aa75-e2af158598e8	10	111152499	111157512	11	5013	NA	1	6	NA	4	NA
Panc-AdenoCA	25cf7449-8c5b-403e-aa75-e2af158598e8	12	11478056	11479119	9	1063	1	5	3	NA	NA	NA
Panc-AdenoCA	25cf7449-8c5b-403e-aa75-e2af158598e8	14	84291103	84291767	6	664	1	4	1	NA	NA	NA
Panc-AdenoCA	25cf7449-8c5b-403e-aa75-e2af158598e8	14	97726463	97727381	12	918	4	5	3	NA	NA	NA
Panc-AdenoCA	25cf7449-8c5b-403e-aa75-e2af158598e8	15	30229385	30233723	16	4338	NA	7	5	NA	3	1
Ovary-AdenoCA	25f73b03-4ba8-47ba-ad80-6ccb82105c98	2	241343849	241349811	11	5962	2	4	5	NA	NA	NA
Ovary-AdenoCA	25f73b03-4ba8-47ba-ad80-6ccb82105c98	6	125360714	125363350	10	2636	1	5	4	NA	NA	NA
Ovary-AdenoCA	25f73b03-4ba8-47ba-ad80-6ccb82105c98	11	133595667	133598434	8	2767	1	5	2	NA	NA	NA
Ovary-AdenoCA	25f73b03-4ba8-47ba-ad80-6ccb82105c98	19	34641842	34643221	14	1379	5	6	3	NA	NA	NA
Ovary-AdenoCA	25f73b03-4ba8-47ba-ad80-6ccb82105c98	X	43622450	43624038	6	1588	1	2	3	NA	NA	NA
Ovary-AdenoCA	25f73b03-4ba8-47ba-ad80-6ccb82105c98	X	55101908	55102706	7	798	1	1	1	4	NA	NA
Lymph-CLL	278b2498-1d64-493b-ac43-3489ec86f313	14	106175702	106177016	14	1314	4	5	5	NA	NA	NA
Lymph-CLL	278b2498-1d64-493b-ac43-3489ec86f313	14	106326603	106330579	7	3976	NA	1	6	NA	NA	NA
Breast-AdenoCa	27f87d1e-2c32-4beb-9677-62f7a286673d	3	79846217	79848553	6	2336	NA	3	3	NA	NA	NA
Breast-AdenoCa	27f87d1e-2c32-4beb-9677-62f7a286673d	8	65715944	65718923	7	2979	1	6	NA	NA	NA	NA
Breast-AdenoCa	27f87d1e-2c32-4beb-9677-62f7a286673d	8	73081542	73087553	10	6011	NA	4	5	NA	NA	1
Breast-AdenoCa	27f87d1e-2c32-4beb-9677-62f7a286673d	8	73377415	73378673	7	1258	NA	4	3	NA	NA	NA
Breast-AdenoCa	27f87d1e-2c32-4beb-9677-62f7a286673d	9	127685757	127686257	7	500	2	2	3	NA	NA	NA
Breast-AdenoCa	27f87d1e-2c32-4beb-9677-62f7a286673d	11	10985049	10985451	7	402	NA	2	5	NA	NA	NA
Breast-AdenoCa	27f87d1e-2c32-4beb-9677-62f7a286673d	11	95217474	95224874	24	7400	5	10	8	NA	NA	1
Breast-AdenoCa	27f87d1e-2c32-4beb-9677-62f7a286673d	12	63753942	63757018	8	3076	2	6	NA	NA	NA	NA
Breast-AdenoCa	27f87d1e-2c32-4beb-9677-62f7a286673d	12	93112418	93116188	8	3770	2	3	3	NA	NA	NA
Breast-AdenoCa	27f87d1e-2c32-4beb-9677-62f7a286673d	13	22848040	22849810	6	1770	NA	3	3	NA	NA	NA
Breast-AdenoCa	27f87d1e-2c32-4beb-9677-62f7a286673d	13	40142575	40144458	12	1883	NA	6	6	NA	NA	NA
Breast-AdenoCa	27f87d1e-2c32-4beb-9677-62f7a286673d	13	40237796	40241564	21	3768	NA	6	15	NA	NA	NA
Breast-AdenoCa	27f87d1e-2c32-4beb-9677-62f7a286673d	13	98836975	98837825	11	850	NA	9	2	NA	NA	NA
Breast-AdenoCa	27f87d1e-2c32-4beb-9677-62f7a286673d	18	66038822	66041811	10	2989	NA	8	2	NA	NA	NA
Breast-AdenoCa	27f87d1e-2c32-4beb-9677-62f7a286673d	20	12088981	12089978	12	997	2	8	2	NA	NA	NA
Breast-AdenoCa	27f87d1e-2c32-4beb-9677-62f7a286673d	20	59282128	59287289	10	5161	NA	10	NA	NA	NA	NA
Lymph-BNHL	282b45ee-4af2-4c61-9acc-286eaf8d661a	2	89157726	89248252	131	90526	5	17	33	43	17	16
Lymph-BNHL	282b45ee-4af2-4c61-9acc-286eaf8d661a	4	138027232	138031656	6	4424	1	1	NA	1	1	2
Lymph-BNHL	282b45ee-4af2-4c61-9acc-286eaf8d661a	5	158371280	158376849	7	5569	2	NA	1	1	1	2
Lymph-BNHL	282b45ee-4af2-4c61-9acc-286eaf8d661a	14	106211740	106241052	52	29312	8	11	31	NA	NA	2
Lymph-BNHL	282b45ee-4af2-4c61-9acc-286eaf8d661a	14	106323084	106330400	99	7316	10	32	36	10	6	5
Lymph-BNHL	282b45ee-4af2-4c61-9acc-286eaf8d661a	14	107113932	107115334	20	1402	2	3	6	3	NA	6
Lymph-BNHL	282b45ee-4af2-4c61-9acc-286eaf8d661a	16	19723096	19725495	8	2399	1	3	4	NA	NA	NA
Lymph-BNHL	282b45ee-4af2-4c61-9acc-286eaf8d661a	18	60983975	60988279	18	4304	1	3	10	2	NA	2
Lymph-BNHL	282b45ee-4af2-4c61-9acc-286eaf8d661a	22	23229994	23232383	13	2389	NA	2	3	3	2	3
Eso-AdenoCa	28e81540-4744-4865-b627-c7c9d8a3c2b8	8	105226115	105228351	9	2236	NA	NA	NA	4	3	2
Eso-AdenoCa	28e81540-4744-4865-b627-c7c9d8a3c2b8	10	50036066	50038101	8	2035	1	3	4	NA	NA	NA
Panc-AdenoCA	28f41a20-b6d6-4ecc-888f-72f779ad9af7	1	110179072	110179215	6	143	2	NA	4	NA	NA	NA
Panc-AdenoCA	28f41a20-b6d6-4ecc-888f-72f779ad9af7	1	111297628	111297854	12	226	NA	NA	12	NA	NA	NA
Panc-AdenoCA	28f41a20-b6d6-4ecc-888f-72f779ad9af7	1	238070699	238072428	37	1729	NA	NA	37	NA	NA	NA
Panc-AdenoCA	28f41a20-b6d6-4ecc-888f-72f779ad9af7	1	245589429	245593983	17	4554	1	4	10	NA	NA	2
Liver-HCC	295aac88-c623-11e3-bf01-24c6515278c0	6	90907022	90907747	6	725	NA	3	3	NA	NA	NA
Liver-HCC	295aac88-c623-11e3-bf01-24c6515278c0	7	85848792	85853366	7	4574	1	1	NA	1	3	1
Liver-HCC	295aac88-c623-11e3-bf01-24c6515278c0	9	36443329	36449814	8	6485	2	4	2	NA	NA	NA
Panc-AdenoCA	29a00d78-b9bb-4c6b-b142-d5b8bfa63455	8	5798960	5801395	8	2435	1	NA	7	NA	NA	NA

Panc-AdenoCA	29a00d78-b9bb-4c6b-b142-d5b8bfa63455	18	41660312	41663139	11	2827	NA	3	8	NA	NA	NA		
Liver-HCC	29b92b51-7ba3-42a9-97d3-6a9b5e43f928	2	234611744	234612283	6	539	NA	NA	NA	2	3	1		
Ovary-AdenoCA	2a8d63eb-0174-4213-9214-413f391f512c	3	50094940	50098524	9	3584	2	4	3	NA	NA	NA		
Ovary-AdenoCA	2a8d63eb-0174-4213-9214-413f391f512c	7	14576375	14577199	6	824	2	3	1	NA	NA	NA		
Ovary-AdenoCA	2a8d63eb-0174-4213-9214-413f391f512c	19	9852603	9853408	8	805	2	3	3	NA	NA	NA		
Ovary-AdenoCA	2a8d63eb-0174-4213-9214-413f391f512c	19	43815212	43818390	12	3178	6	6	NA	NA	NA	NA		
Ovary-AdenoCA	2a8d63eb-0174-4213-9214-413f391f512c	19	46460948	46466226	10	5278	2	8	NA	NA	NA	NA		
CNS-Oligo	2aeaab9f-4459-4be8-91e7-e0746cbd671c	2	214712864	214720999	25	8135	NA	7	18	NA	NA	NA		
CNS-Oligo	2aeaab9f-4459-4be8-91e7-e0746cbd671c	2	215474326	215478978	7	4652	1	4	2	NA	NA	NA		
Breast-AdenoCa	2b000af3-2c9d-4eaa-af3f-8101b7425c37	8	36015029	36025025	27	9996	3	18	6	NA	NA	NA		
Breast-AdenoCa	2b000af3-2c9d-4eaa-af3f-8101b7425c37	8	70807634	70813491	9	5857	2	3	4	NA	NA	NA		
Breast-AdenoCa	2b000af3-2c9d-4eaa-af3f-8101b7425c37	11	59966239	59967570	9	1331	NA	5	3	NA	1	NA		
Breast-AdenoCa	2b000af3-2c9d-4eaa-af3f-8101b7425c37	20	31143248	31143262	6	14	NA	1	NA	4	1	NA		
Lymph-BNHL	2b02e3ad-4423-491e-993a-561e80dc7bcf	8	128748694	128750683	6	1989	1	NA	5	NA	NA	NA		
Lymph-BNHL	2b02e3ad-4423-491e-993a-561e80dc7bcf	14	106323597	106330408	37	6811	3	10	12	9	2	1		
Lymph-BNHL	2b02e3ad-4423-491e-993a-561e80dc7bcf	14	106382966	106385055	13	2089	NA	3	6	3	1	NA		
Lymph-BNHL	2b02e3ad-4423-491e-993a-561e80dc7bcf	14	106725289	106725524	10	235	NA	5	5	NA	NA	NA		
Lymph-BNHL	2b02e3ad-4423-491e-993a-561e80dc7bcf	22	22758762	22764599	24	5837	3	8	10	NA	2	1		
Lymph-BNHL	2b02e3ad-4423-491e-993a-561e80dc7bcf	22	23247368	23247673	9	305	NA	5	1	2	NA	1		
Bladder-TCC	2b142863-b963-4cc9-8f8f-c72503c93390	1	27322609	27328146	7	5537	1	1	5	NA	NA	NA		
Bladder-TCC	2b142863-b963-4cc9-8f8f-c72503c93390	1	32392300	32394483	7	2183	1	1	5	NA	NA	NA		
Bladder-TCC	2b142863-b963-4cc9-8f8f-c72503c93390	1	110086286	110095010	13	8724	3	4	5	NA	NA	1		
Bladder-TCC	2b142863-b963-4cc9-8f8f-c72503c93390	2	10115572	10119675	6	4103	NA	2	4	NA	NA	NA		
Bladder-TCC	2b142863-b963-4cc9-8f8f-c72503c93390	2	32285871	32294460	12	8589	1	6	5	NA	NA	NA		
Bladder-TCC	2b142863-b963-4cc9-8f8f-c72503c93390	2	64987329	64993006	8	5677	NA	2	6	NA	NA	NA		
Bladder-TCC	2b142863-b963-4cc9-8f8f-c72503c93390	2	85849471	85855376	9	5905	NA	2	7	NA	NA	NA		
Bladder-TCC	2b142863-b963-4cc9-8f8f-c72503c93390	3	124192245	124195377	8	3132	1	2	5	NA	NA	NA		
Bladder-TCC	2b142863-b963-4cc9-8f8f-c72503c93390	3	188554261	188556245	8	1984	NA	4	4	NA	NA	NA		
Bladder-TCC	2b142863-b963-4cc9-8f8f-c72503c93390	4	83973290	83974674	10	1384	2	6	2	NA	NA	NA		
Bladder-TCC	2b142863-b963-4cc9-8f8f-c72503c93390	5	31475733	31477026	6	1293	NA	2	4	NA	NA	NA		
Bladder-TCC	2b142863-b963-4cc9-8f8f-c72503c93390	5	124274288	124277879	7	3591	1	2	4	NA	NA	NA		
Bladder-TCC	2b142863-b963-4cc9-8f8f-c72503c93390	6	31766264	31770193	9	3929	1	4	4	NA	NA	NA		
Bladder-TCC	2b142863-b963-4cc9-8f8f-c72503c93390	6	53628410	53631223	8	2813	2	4	2	NA	NA	NA		
Bladder-TCC	2b142863-b963-4cc9-8f8f-c72503c93390	6	71412170	71413034	7	864	2	1	4	NA	NA	NA		
Bladder-TCC	2b142863-b963-4cc9-8f8f-c72503c93390	6	136502541	136507194	11	4653	2	NA	9	NA	NA	NA		
Bladder-TCC	2b142863-b963-4cc9-8f8f-c72503c93390	6	136692875	136699716	18	6841	2	5	11	NA	NA	NA		
Bladder-TCC	2b142863-b963-4cc9-8f8f-c72503c93390	6	142736750	142741269	12	4519	2	3	7	NA	NA	NA		
Bladder-TCC	2b142863-b963-4cc9-8f8f-c72503c93390	6	148063046	148064292	11	1246	2	4	5	NA	NA	NA		
Bladder-TCC	2b142863-b963-4cc9-8f8f-c72503c93390	7	17463858	17465976	6	2118	NA	1	5	NA	NA	NA		
Bladder-TCC	2b142863-b963-4cc9-8f8f-c72503c93390	7	73522174	73523217	7	1043	1	2	4	NA	NA	NA		
Bladder-TCC	2b142863-b963-4cc9-8f8f-c72503c93390	8	126061025	126065932	6	4907	1	3	2	NA	NA	NA		
Bladder-TCC	2b142863-b963-4cc9-8f8f-c72503c93390	8	134893082	134895999	9	2917	NA	5	4	NA	NA	NA		
Bladder-TCC	2b142863-b963-4cc9-8f8f-c72503c93390	10	741556	747842	23	6286	1	5	16	1	NA	NA		
Bladder-TCC	2b142863-b963-4cc9-8f8f-c72503c93390	10	5996273	5998051	7	1778	NA	NA	7	NA	NA	NA		
Bladder-TCC	2b142863-b963-4cc9-8f8f-c72503c93390	10	45558102	45562236	21	4134	2	4	15	NA	NA	NA		
Bladder-TCC	2b142863-b963-4cc9-8f8f-c72503c93390	10	75173712	75177791	10	4079	1	1	8	NA	NA	NA		
Bladder-TCC	2b142863-b963-4cc9-8f8f-c72503c93390	10	95807722	95811961	7	4239	2	4	1	NA	NA	NA		
Bladder-TCC	2b142863-b963-4cc9-8f8f-c72503c93390	11	64585741	64589767	6	4026	NA	5	1	NA	NA	NA		
Bladder-TCC	2b142863-b963-4cc9-8f8f-c72503c93390	11	96997809	97001339	21	3530	3	4	13	NA	NA	1		
Bladder-TCC	2b142863-b963-4cc9-8f8f-c72503c93390	12	2487783	2488494	8	711	NA	1	6	1	NA	NA		
Bladder-TCC	2b142863-b963-4cc9-8f8f-c72503c93390	12	57126723	57132920	12	6197	2	6	4	NA	NA	NA		
Bladder-TCC	2b142863-b963-4cc9-8f8f-c72503c93390	12	125385362	125387577	8	2215	NA	5	3	NA	NA	NA		
Bladder-TCC	2b142863-b963-4cc9-8f8f-c72503c93390	14	68653980	68658894	6	4914	3	1	2	NA	NA	NA		
Bladder-TCC	2b142863-b963-4cc9-8f8f-c72503c93390	15	101040394	101045997	9	5603	NA	2	7	NA	NA	NA		
Bladder-TCC	2b142863-b963-4cc9-8f8f-c72503c93390	16	1688807	1692335	7	3528	1	6	NA	NA	NA	NA		
Bladder-TCC	2b142863-b963-4cc9-8f8f-c72503c93390	16	16231040	16251108	24	20068	4	11	8	NA	1	NA		
Bladder-TCC	2b142863-b963-4cc9-8f8f-c72503c93390	16	23493148	23497135	13	3987	2	3	8	NA	NA	NA		
Bladder-TCC	2b142863-b963-4cc9-8f8f-c72503c93390	16	89402532	89407347	12	4815	NA	4	8	NA	NA	NA		
Bladder-TCC	2b142863-b963-4cc9-8f8f-c72503c93390	17	4413297	4414633	8	1336	NA	2	6	NA	NA	NA		
Bladder-TCC	2b142863-b963-4cc9-8f8f-c72503c93390	17	27070045	27082639	16	12594	1	7	8	NA	NA	NA		

Bladder-TCC	2b142863-b963-4cc9-8f8f-c72503c93390	17	30578079	30579235	9	1156	1	4	4	NA	NA	NA
Bladder-TCC	2b142863-b963-4cc9-8f8f-c72503c93390	19	11100361	11101644	9	1283	1	2	6	NA	NA	NA
Bladder-TCC	2b142863-b963-4cc9-8f8f-c72503c93390	20	5937805	5945216	21	7411	NA	8	13	NA	NA	NA
Bladder-TCC	2b142863-b963-4cc9-8f8f-c72503c93390	20	60765146	60768912	12	3766	NA	4	8	NA	NA	NA
Bladder-TCC	2b142863-b963-4cc9-8f8f-c72503c93390	21	36193368	36198023	6	4655	NA	2	4	NA	NA	NA
Bladder-TCC	2b142863-b963-4cc9-8f8f-c72503c93390	22	30367748	30368524	7	776	NA	2	5	NA	NA	NA
Bladder-TCC	2b142863-b963-4cc9-8f8f-c72503c93390	22	31511884	31514970	7	3086	1	4	2	NA	NA	NA
Bladder-TCC	2b142863-b963-4cc9-8f8f-c72503c93390	22	36249129	36251469	13	2340	2	4	7	NA	NA	NA
Bladder-TCC	2b142863-b963-4cc9-8f8f-c72503c93390	22	46408116	46409156	7	1040	NA	5	2	NA	NA	NA
Lymph-BNHL	2b36ac4b-eed3-4591-8b4f-b44049dfc5ed	14	106325088	106331445	30	6357	2	7	19	1	NA	1
Panc-AdenoCA	2b3837b3-ee29-4a08-848e-1206404da986	16	34775287	34775324	7	37	1	NA	2	4	NA	NA
Panc-AdenoCA	2b3837b3-ee29-4a08-848e-1206404da986	22	36407210	36408198	11	988	4	1	6	NA	NA	NA
Eso-AdenoCa	2b41746a-95c0-4875-afb5-c6d3d3ad30d9	4	179005976	179008817	10	2841	NA	NA	NA	4	5	1
Eso-AdenoCa	2b41746a-95c0-4875-afb5-c6d3d3ad30d9	5	20484077	20490917	8	6840	1	NA	NA	2	3	2
Eso-AdenoCa	2b41746a-95c0-4875-afb5-c6d3d3ad30d9	5	20493458	20497760	13	4302	1	NA	3	2	4	3
Eso-AdenoCa	2b41746a-95c0-4875-afb5-c6d3d3ad30d9	7	87181382	87182540	7	1158	NA	1	3	1	NA	2
Eso-AdenoCa	2b41746a-95c0-4875-afb5-c6d3d3ad30d9	17	49718772	49721356	9	2584	2	5	2	NA	NA	NA
Eso-AdenoCa	2b41746a-95c0-4875-afb5-c6d3d3ad30d9	17	52056564	52056732	7	168	NA	NA	7	NA	NA	NA
Eso-AdenoCa	2b41746a-95c0-4875-afb5-c6d3d3ad30d9	X	12896277	12898286	15	2009	2	10	2	NA	1	NA
Ovary-AdenoCA	2b4feb84-89e4-4c38-8561-5ffab02c8132	2	206074018	206075047	6	1029	NA	4	2	NA	NA	NA
Panc-AdenoCA	2b6d4d66-7f0b-4bc0-b3d6-171956a937c5	2	12463810	12466024	23	2214	2	12	9	NA	NA	NA
Panc-AdenoCA	2b6d4d66-7f0b-4bc0-b3d6-171956a937c5	3	185845997	185846441	7	444	3	NA	4	NA	NA	NA
Panc-AdenoCA	2b6d4d66-7f0b-4bc0-b3d6-171956a937c5	4	143063285	143065032	7	1747	NA	2	5	NA	NA	NA
Panc-AdenoCA	2b6d4d66-7f0b-4bc0-b3d6-171956a937c5	6	116370854	116372941	7	2087	NA	2	2	1	2	NA
Panc-AdenoCA	2b6d4d66-7f0b-4bc0-b3d6-171956a937c5	7	107292299	107295243	11	2944	1	5	5	NA	NA	NA
Panc-AdenoCA	2b6d4d66-7f0b-4bc0-b3d6-171956a937c5	12	93817684	93819637	28	1953	3	8	16	NA	1	NA
Panc-AdenoCA	2b6d4d66-7f0b-4bc0-b3d6-171956a937c5	12	103984122	103985443	9	1321	NA	NA	9	NA	NA	NA
Panc-AdenoCA	2b6d4d66-7f0b-4bc0-b3d6-171956a937c5	12	103998475	103999282	19	807	2	2	14	NA	1	NA
Panc-AdenoCA	2b6d4d66-7f0b-4bc0-b3d6-171956a937c5	12	117253417	117255698	8	2281	1	5	2	NA	NA	NA
Panc-AdenoCA	2b6d4d66-7f0b-4bc0-b3d6-171956a937c5	X	94052100	94053650	10	1550	NA	4	6	NA	NA	NA
Panc-AdenoCA	2b6d4d66-7f0b-4bc0-b3d6-171956a937c5	X	99369515	99371792	10	2277	1	6	3	NA	NA	NA
Panc-AdenoCA	2b6d4d66-7f0b-4bc0-b3d6-171956a937c5	X	144654380	144656359	9	1979	NA	3	6	NA	NA	NA
Panc-AdenoCA	2b6d4d66-7f0b-4bc0-b3d6-171956a937c5	X	154835324	154835897	14	573	1	7	6	NA	NA	NA
Uterus-AdenoCA	2b78de4e-4c8b-4adf-a058-3dae797e7881	8	95839149	95839327	6	178	NA	NA	3	3	NA	NA
Panc-AdenoCA	2bd9ccca-3fae-4b66-a762-6f30d6276222	12	29582053	29582780	7	727	1	1	5	NA	NA	NA
Panc-AdenoCA	2bd9ccca-3fae-4b66-a762-6f30d6276222	20	51183576	51183945	12	369	3	3	6	NA	NA	NA
Panc-AdenoCA	2bd9ccca-3fae-4b66-a762-6f30d6276222	22	46452330	46454358	26	2028	4	9	13	NA	NA	NA
Panc-AdenoCA	2bf5b018-9f19-4fbd-9e1f-7d958aabe5d1	1	153304466	153308111	6	3645	2	1	2	NA	NA	1
Panc-AdenoCA	2bf5b018-9f19-4fbd-9e1f-7d958aabe5d1	2	12230916	12232134	10	1218	2	7	1	NA	NA	NA
Panc-AdenoCA	2bf5b018-9f19-4fbd-9e1f-7d958aabe5d1	3	78089541	78090158	8	617	1	6	1	NA	NA	NA
Panc-AdenoCA	2bf5b018-9f19-4fbd-9e1f-7d958aabe5d1	4	190841640	190843560	12	1920	2	7	3	NA	NA	NA
Panc-AdenoCA	2bf5b018-9f19-4fbd-9e1f-7d958aabe5d1	6	37543463	37547770	8	4307	1	4	3	NA	NA	NA
Panc-AdenoCA	2bf5b018-9f19-4fbd-9e1f-7d958aabe5d1	7	157245955	157247516	11	1561	1	7	3	NA	NA	NA
Panc-AdenoCA	2bf5b018-9f19-4fbd-9e1f-7d958aabe5d1	9	36669493	36672664	12	3171	4	3	5	NA	NA	NA
Panc-AdenoCA	2bf5b018-9f19-4fbd-9e1f-7d958aabe5d1	14	56480216	56483267	7	3051	2	2	2	NA	1	NA
Panc-AdenoCA	2bf5b018-9f19-4fbd-9e1f-7d958aabe5d1	16	67871956	67873971	11	2015	2	3	6	NA	NA	NA
Panc-AdenoCA	2bf5b018-9f19-4fbd-9e1f-7d958aabe5d1	18	9809489	9810239	9	750	3	5	1	NA	NA	NA
Panc-AdenoCA	2bf5b018-9f19-4fbd-9e1f-7d958aabe5d1	18	65448452	65448868	8	416	2	6	NA	NA	NA	NA
Panc-AdenoCA	2bf5b018-9f19-4fbd-9e1f-7d958aabe5d1	18	72338798	72339353	8	555	3	2	3	NA	NA	NA
Panc-AdenoCA	2bf5b018-9f19-4fbd-9e1f-7d958aabe5d1	19	29275391	29280161	6	4770	NA	1	5	NA	NA	NA
Liver-HCC	2bff30d5-be79-4686-8164-7a7d9619d3c0	1	157372985	157374999	19	2014	1	9	9	NA	NA	NA
Liver-HCC	2bff30d5-be79-4686-8164-7a7d9619d3c0	1	171430447	171434100	13	3653	1	3	9	NA	NA	NA
Liver-HCC	2bff30d5-be79-4686-8164-7a7d9619d3c0	4	63327208	63338034	20	10826	2	7	7	2	NA	2
Liver-HCC	2bff30d5-be79-4686-8164-7a7d9619d3c0	8	65151301	65152514	8	1213	2	5	1	NA	NA	NA
Liver-HCC	2bff30d5-be79-4686-8164-7a7d9619d3c0	8	65272388	65289227	22	16839	1	3	18	NA	NA	NA
Lymph-BNHL	2c3ada8b-9650-4859-9857-43209c0f7cea	2	89158119	89159095	16	976	NA	3	1	6	4	2
Lymph-BNHL	2c3ada8b-9650-4859-9857-43209c0f7cea	3	63068265	63069161	6	896	NA	1	1	3	NA	1
Lymph-BNHL	2c3ada8b-9650-4859-9857-43209c0f7cea	4	117718755	117725654	8	6899	NA	1	1	4	1	1
Lymph-BNHL	2c3ada8b-9650-4859-9857-43209c0f7cea	5	24134632	24137859	7	3227	NA	NA	NA	1	3	3
Lymph-BNHL	2c3ada8b-9650-4859-9857-43209c0f7cea	8	84398827	84401493	6	2666	NA	1	NA	2	2	1

Lymph-BNHL	2c3ada8b-9650-4859-9857-43209c0f7cea	9	14117826	14122587	6	4761	NA	NA	2	1	1	2
Lymph-BNHL	2c3ada8b-9650-4859-9857-43209c0f7cea	9	14132560	14140601	10	8041	NA	NA	1	6	1	2
Lymph-BNHL	2c3ada8b-9650-4859-9857-43209c0f7cea	10	58342465	58347303	7	4838	1	1	1	3	NA	1
Lymph-BNHL	2c3ada8b-9650-4859-9857-43209c0f7cea	11	81974014	81976682	6	2668	NA	NA	NA	1	2	3
Lymph-BNHL	2c3ada8b-9650-4859-9857-43209c0f7cea	13	96769498	96775213	12	5715	NA	1	NA	2	6	3
Lymph-BNHL	2c3ada8b-9650-4859-9857-43209c0f7cea	14	106112679	106114354	11	1675	NA	1	10	NA	NA	NA
Lymph-BNHL	2c3ada8b-9650-4859-9857-43209c0f7cea	14	106323782	106330157	82	6375	4	14	39	12	11	2
Lymph-BNHL	2c3ada8b-9650-4859-9857-43209c0f7cea	14	106725618	106726127	14	509	1	2	2	4	3	2
Lymph-BNHL	2c3ada8b-9650-4859-9857-43209c0f7cea	18	60984719	60988235	26	3516	1	5	5	8	2	5
Lymph-BNHL	2c3ada8b-9650-4859-9857-43209c0f7cea	22	22730736	22735610	8	4874	1	2	3	NA	2	NA
Lymph-BNHL	2c3ada8b-9650-4859-9857-43209c0f7cea	22	23230360	23253828	48	23468	2	7	19	9	6	5
Lymph-BNHL	2c3ada8b-9650-4859-9857-43209c0f7cea	X	99901397	99905494	7	4097	1	NA	NA	3	2	1
Lymph-BNHL	2c3ada8b-9650-4859-9857-43209c0f7cea	X	99934734	99940174	8	5440	NA	2	2	1	NA	3
Kidney-RCC	2c581a74-7716-4c20-b366-a8e1d9a901f6	1	241091269	241091848	8	579	2	3	3	NA	NA	NA
Kidney-RCC	2c581a74-7716-4c20-b366-a8e1d9a901f6	2	31056351	31057274	10	923	1	6	3	NA	NA	NA
Breast-AdenoCa	2c6f1862-bb82-4e7e-9cb3-338bdf022ff4	17	11545423	11546728	7	1305	1	4	2	NA	NA	NA
Breast-AdenoCa	2c6f1862-bb82-4e7e-9cb3-338bdf022ff4	17	31863857	31865701	8	1844	NA	6	2	NA	NA	NA
Breast-AdenoCa	2c6f1862-bb82-4e7e-9cb3-338bdf022ff4	17	32389482	32390226	7	744	NA	NA	NA	2	5	NA
Breast-AdenoCa	2c6f1862-bb82-4e7e-9cb3-338bdf022ff4	17	38245808	38249986	7	4178	NA	4	3	NA	NA	NA
Prost-AdenoCA	2c9c50ff-01d9-4703-931b-04119178706f	3	124208799	124211611	17	2812	1	3	13	NA	NA	NA
Ovary-AdenoCA	2c9dc04b-e9ec-4cf1-ab2c-a18edb30dd37	7	396929	399991	6	3062	6	NA	NA	NA	NA	NA
ColoRect-AdenoCA	2cdc4cce-0877-4ec1-85f5-20c909912e65	8	40694527	40696992	7	2465	NA	4	3	NA	NA	NA
Liver-HCC	2d0e4b82-c623-11e3-bf01-24c6515278c0	17	5334415	5335822	10	1407	NA	NA	10	NA	NA	NA
Kidney-RCC	2deb8f9a-9e0a-46d1-bcb5-902db3b5c8ac	1	247996506	247997054	8	548	NA	NA	8	NA	NA	NA
Panc-AdenoCA	2e43e0ca-54ea-482e-acf2-0048d9187a5c	2	232587155	232588211	16	1056	2	7	7	NA	NA	NA
Panc-AdenoCA	2e43e0ca-54ea-482e-acf2-0048d9187a5c	4	121067568	121068383	11	815	3	2	6	NA	NA	NA
Panc-AdenoCA	2e43e0ca-54ea-482e-acf2-0048d9187a5c	6	6329873	6330591	6	718	NA	NA	6	NA	NA	NA
Panc-AdenoCA	2e43e0ca-54ea-482e-acf2-0048d9187a5c	6	116855522	116865873	42	10351	9	17	16	NA	NA	NA
Panc-AdenoCA	2e43e0ca-54ea-482e-acf2-0048d9187a5c	16	80507712	80509084	18	1372	2	9	7	NA	NA	NA
Prost-AdenoCA	2ea2294d-fab9-43ae-a222-370487495b06	6	85348251	85348468	6	217	NA	NA	NA	NA	6	NA
Prost-AdenoCA	2ea2294d-fab9-43ae-a222-370487495b06	8	42924592	42934309	16	9717	NA	6	10	NA	NA	NA
Liver-HCC	2f149d78-c623-11e3-bf01-24c6515278c0	11	74908246	74914412	9	6166	3	3	3	NA	NA	NA
Ovary-AdenoCA	2f2eaecc-6509-423f-b63a-8c3bea1ba4a4	12	133763526	133765003	6	1477	2	3	1	NA	NA	NA
Ovary-AdenoCA	2f2eaecc-6509-423f-b63a-8c3bea1ba4a4	14	106657473	106661663	7	4190	NA	2	4	1	NA	NA
Ovary-AdenoCA	2f2eaecc-6509-423f-b63a-8c3bea1ba4a4	14	106963866	106964257	10	391	NA	3	7	NA	NA	NA
Prost-AdenoCA	2f79fe89-9d24-4fbe-ba5b-0ff6c3c4d663	3	180297527	180301515	13	3988	NA	5	8	NA	NA	NA
Prost-AdenoCA	2f79fe89-9d24-4fbe-ba5b-0ff6c3c4d663	7	79649091	79649913	8	822	NA	NA	8	NA	NA	NA
Prost-AdenoCA	2f79fe89-9d24-4fbe-ba5b-0ff6c3c4d663	12	88071951	88074841	6	2890	1	3	2	NA	NA	NA
Prost-AdenoCA	2f79fe89-9d24-4fbe-ba5b-0ff6c3c4d663	13	103365102	103366196	7	1094	NA	4	3	NA	NA	NA
Prost-AdenoCA	2f79fe89-9d24-4fbe-ba5b-0ff6c3c4d663	19	57077929	57078575	8	646	1	1	6	NA	NA	NA
Panc-AdenoCA	2fdfb06c-0d2f-4e1d-9756-bcb08a7f90af	2	49168500	49169530	6	1030	1	2	3	NA	NA	NA
Panc-AdenoCA	2fdfb06c-0d2f-4e1d-9756-bcb08a7f90af	2	51491370	51494484	26	3114	NA	NA	26	NA	NA	NA
Panc-AdenoCA	2fdfb06c-0d2f-4e1d-9756-bcb08a7f90af	4	42781387	42781863	7	476	1	3	3	NA	NA	NA
Panc-AdenoCA	2fdfb06c-0d2f-4e1d-9756-bcb08a7f90af	5	106725680	106726230	6	550	1	2	3	NA	NA	NA
Panc-AdenoCA	2fdfb06c-0d2f-4e1d-9756-bcb08a7f90af	5	107808501	107811079	8	2578	1	1	6	NA	NA	NA
Panc-AdenoCA	2fdfb06c-0d2f-4e1d-9756-bcb08a7f90af	5	115759098	115769700	20	10602	2	4	13	NA	NA	1
Panc-AdenoCA	2fdfb06c-0d2f-4e1d-9756-bcb08a7f90af	6	30403086	30404606	7	1520	NA	3	3	1	NA	NA
Panc-AdenoCA	2fdfb06c-0d2f-4e1d-9756-bcb08a7f90af	7	98656424	98656432	6	8	2	NA	3	NA	NA	1
Panc-AdenoCA	2fdfb06c-0d2f-4e1d-9756-bcb08a7f90af	8	18739903	18745237	8	5334	NA	2	6	NA	NA	NA
Panc-AdenoCA	2fdfb06c-0d2f-4e1d-9756-bcb08a7f90af	18	25172750	25174569	9	1819	3	1	5	NA	NA	NA
Panc-AdenoCA	2fdfb06c-0d2f-4e1d-9756-bcb08a7f90af	18	25216210	25217185	25	975	4	4	17	NA	NA	NA
Panc-AdenoCA	2fdfb06c-0d2f-4e1d-9756-bcb08a7f90af	X	48614493	48615104	6	611	1	3	2	NA	NA	NA
Prost-AdenoCA	304c3e10-583c-421d-ab01-15c937d5014a	8	38315495	38315890	6	395	1	3	1	1	NA	NA
Prost-AdenoCA	304c3e10-583c-421d-ab01-15c937d5014a	10	14235130	14235823	6	693	1	3	2	NA	NA	NA
Prost-AdenoCA	304c3e10-583c-421d-ab01-15c937d5014a	10	103777753	103778511	7	758	NA	2	5	NA	NA	NA
Prost-AdenoCA	306ef5bc-97d0-4312-ae0e-343c4e31d869	5	85748454	85749267	10	813	NA	NA	NA	1	9	NA
Stomach-AdenoCA	30b9ab02-60f2-4974-85a9-874f247ae932	5	13552	17089	8	3537	4	3	1	NA	NA	NA
Stomach-AdenoCA	30b9ab02-60f2-4974-85a9-874f247ae932	21	20696079	20698479	18	2400	5	9	4	NA	NA	NA
Stomach-AdenoCA	30b9ab02-60f2-4974-85a9-874f247ae932	21	45014125	45021738	9	7613	3	3	3	NA	NA	NA
Stomach-AdenoCA	30b9ab02-60f2-4974-85a9-874f247ae932	21	45027330	45030873	8	3543	1	1	6	NA	NA	NA

Stomach-AdenoCA	30b9ab02-60f2-4974-85a9-874f247ae932	22	22053206	22057044	6	3838	2	NA	4	NA	NA	NA
Stomach-AdenoCA	30b9ab02-60f2-4974-85a9-874f247ae932	22	22070658	22072849	11	2191	3	4	3	NA	1	NA
Stomach-AdenoCA	30b9ab02-60f2-4974-85a9-874f247ae932	22	41786146	41790800	9	4654	1	NA	8	NA	NA	NA
Stomach-AdenoCA	30b9ab02-60f2-4974-85a9-874f247ae932	22	46578740	46587490	13	8750	3	3	7	NA	NA	NA
Uterus-AdenoCA	31bc44b9-35ff-43fd-8a01-a834f3b1ce46	4	187386386	187386958	6	572	NA	NA	3	3	NA	NA
Uterus-AdenoCA	31bc44b9-35ff-43fd-8a01-a834f3b1ce46	20	52915675	52916456	6	781	1	NA	2	3	NA	NA
Uterus-AdenoCA	31bc44b9-35ff-43fd-8a01-a834f3b1ce46	X	19577124	19577245	6	121	NA	1	3	1	NA	1
Panc-AdenoCA	31c75873-abb4-4d88-9e2f-07497a6c892d	1	6002874	6004192	10	1318	2	2	6	NA	NA	NA
Panc-AdenoCA	31c75873-abb4-4d88-9e2f-07497a6c892d	1	72728374	72729365	13	991	4	3	6	NA	NA	NA
Panc-AdenoCA	31c75873-abb4-4d88-9e2f-07497a6c892d	2	146357015	146359838	7	2823	1	NA	2	3	1	NA
Panc-AdenoCA	31c75873-abb4-4d88-9e2f-07497a6c892d	4	1331900	1339009	14	7109	5	6	3	NA	NA	NA
Panc-AdenoCA	31c75873-abb4-4d88-9e2f-07497a6c892d	4	114546618	114549514	10	2896	2	3	5	NA	NA	NA
Panc-AdenoCA	31c75873-abb4-4d88-9e2f-07497a6c892d	5	36974046	36979369	13	5323	2	1	10	NA	NA	NA
Panc-AdenoCA	31c75873-abb4-4d88-9e2f-07497a6c892d	5	179448245	179448674	8	429	1	4	3	NA	NA	NA
Panc-AdenoCA	31c75873-abb4-4d88-9e2f-07497a6c892d	6	131316849	131317331	7	482	2	4	1	NA	NA	NA
Panc-AdenoCA	31c75873-abb4-4d88-9e2f-07497a6c892d	6	146606598	146612066	9	5468	NA	NA	9	NA	NA	NA
Panc-AdenoCA	31c75873-abb4-4d88-9e2f-07497a6c892d	11	64887881	64895471	15	7590	NA	4	11	NA	NA	NA
Panc-AdenoCA	31c75873-abb4-4d88-9e2f-07497a6c892d	11	66000822	66003618	17	2796	NA	4	13	NA	NA	NA
Panc-AdenoCA	31c75873-abb4-4d88-9e2f-07497a6c892d	11	95897417	95901692	8	4275	1	4	3	NA	NA	NA
Panc-AdenoCA	31c75873-abb4-4d88-9e2f-07497a6c892d	11	111194229	111200185	25	5956	5	6	14	NA	NA	NA
Panc-AdenoCA	31c75873-abb4-4d88-9e2f-07497a6c892d	12	5751831	5752746	11	915	3	2	6	NA	NA	NA
Panc-AdenoCA	31c75873-abb4-4d88-9e2f-07497a6c892d	12	23393320	23393782	7	462	NA	3	4	NA	NA	NA
Panc-AdenoCA	31c75873-abb4-4d88-9e2f-07497a6c892d	12	25603984	25609214	8	5230	1	1	6	NA	NA	NA
Panc-AdenoCA	31c75873-abb4-4d88-9e2f-07497a6c892d	12	25725720	25730401	8	4681	1	NA	7	NA	NA	NA
Panc-AdenoCA	31c75873-abb4-4d88-9e2f-07497a6c892d	12	30819449	30826012	9	6563	3	NA	6	NA	NA	NA
Panc-AdenoCA	31c75873-abb4-4d88-9e2f-07497a6c892d	12	39515522	39522493	8	6971	1	2	3	NA	2	NA
Panc-AdenoCA	31c75873-abb4-4d88-9e2f-07497a6c892d	12	39530542	39533903	8	3361	NA	1	6	NA	NA	1
Panc-AdenoCA	31c75873-abb4-4d88-9e2f-07497a6c892d	18	21549509	21549890	14	381	1	1	11	1	NA	NA
Panc-AdenoCA	31c75873-abb4-4d88-9e2f-07497a6c892d	21	26166979	26170926	6	3947	NA	NA	NA	2	3	1
Kidney-RCC	3232f77f-b745-4232-a802-6699b6356efd	5	86960849	86961444	8	595	3	4	1	NA	NA	NA
Cervix-SCC	3269608c-5dd7-452a-976a-31ac971aac2f	5	80182907	80186781	8	3874	1	2	5	NA	NA	NA
Cervix-SCC	3269608c-5dd7-452a-976a-31ac971aac2f	9	8855246	8856661	7	1415	NA	1	6	NA	NA	NA
Cervix-SCC	3269608c-5dd7-452a-976a-31ac971aac2f	12	18525813	18528600	8	2787	NA	NA	8	NA	NA	NA
Prost-AdenoCA	32d8c373-b5c8-420b-9808-8812b5501649	9	617133	617507	10	374	NA	NA	NA	3	6	1
Panc-AdenoCA	32fa6457-c8bd-4d26-8106-925baf13df68	3	79146207	79147530	12	1323	1	4	7	NA	NA	NA
Panc-AdenoCA	32fa6457-c8bd-4d26-8106-925baf13df68	6	12430257	12431188	9	931	2	2	5	NA	NA	NA
Panc-AdenoCA	32fa6457-c8bd-4d26-8106-925baf13df68	6	32215073	32217198	10	2125	2	3	5	NA	NA	NA
Panc-AdenoCA	32fa6457-c8bd-4d26-8106-925baf13df68	6	55556148	55559961	10	3813	1	2	7	NA	NA	NA
Panc-AdenoCA	32fa6457-c8bd-4d26-8106-925baf13df68	8	111576486	111580026	28	3540	3	16	9	NA	NA	NA
Panc-AdenoCA	32fa6457-c8bd-4d26-8106-925baf13df68	8	114839638	114840730	7	1092	NA	6	1	NA	NA	NA
Panc-AdenoCA	32fa6457-c8bd-4d26-8106-925baf13df68	9	215956	217068	11	1112	1	3	7	NA	NA	NA
Panc-AdenoCA	32fa6457-c8bd-4d26-8106-925baf13df68	18	22195308	22196271	12	963	2	3	7	NA	NA	NA
Panc-AdenoCA	32fa6457-c8bd-4d26-8106-925baf13df68	18	22900447	22906247	7	5800	1	1	3	NA	1	1
Panc-AdenoCA	32fa6457-c8bd-4d26-8106-925baf13df68	18	23193596	23201842	12	8246	NA	3	4	NA	4	1
Panc-AdenoCA	32fa6457-c8bd-4d26-8106-925baf13df68	18	23759991	23762018	7	2027	NA	2	5	NA	NA	NA
Panc-AdenoCA	33070975-0f35-4518-9ffb-4c6170c32534	1	60727853	60731921	14	4068	5	2	7	NA	NA	NA
Panc-AdenoCA	33070975-0f35-4518-9ffb-4c6170c32534	1	60752006	60753105	8	1099	2	2	4	NA	NA	NA
Panc-AdenoCA	33070975-0f35-4518-9ffb-4c6170c32534	13	20755224	20768265	17	13041	3	6	8	NA	NA	NA
Eso-AdenoCa	33992441-4f34-42f2-ba87-6ddd227f2250	11	69637567	69643054	19	5487	1	3	15	NA	NA	NA
Eso-AdenoCa	33992441-4f34-42f2-ba87-6ddd227f2250	16	13892109	13892674	12	565	1	6	5	NA	NA	NA
Panc-AdenoCA	33bcd589-80db-4a58-8f83-a48cb60e0fda	9	34178736	34189069	15	10333	1	NA	14	NA	NA	NA
Panc-AdenoCA	33bcd589-80db-4a58-8f83-a48cb60e0fda	9	34413381	34414832	14	1451	NA	NA	14	NA	NA	NA
Panc-AdenoCA	33bcd589-80db-4a58-8f83-a48cb60e0fda	9	35811655	35817261	23	5606	1	NA	22	NA	NA	NA
Panc-AdenoCA	33bcd589-80db-4a58-8f83-a48cb60e0fda	13	48082328	48085919	11	3591	NA	5	6	NA	NA	NA
Panc-AdenoCA	33bcd589-80db-4a58-8f83-a48cb60e0fda	15	81567053	81569648	6	2595	1	4	1	NA	NA	NA
Panc-AdenoCA	33bcd589-80db-4a58-8f83-a48cb60e0fda	22	39071751	39076127	7	4376	NA	4	3	NA	NA	NA
Prost-AdenoCA	33de44a2-bec1-402d-872c-d78c1f2d52b3	1	189428428	189432274	9	3846	NA	7	2	NA	NA	NA
Prost-AdenoCA	33de44a2-bec1-402d-872c-d78c1f2d52b3	1	192282348	192282888	9	540	NA	6	3	NA	NA	NA
Skin-Melanoma	3433b1f3-59d1-4ac2-9bbf-e0c4042b4ddc	1	99099707	99101606	19	1899	NA	16	3	NA	NA	NA
Skin-Melanoma	3433b1f3-59d1-4ac2-9bbf-e0c4042b4ddc	1	145196317	145200962	10	4645	NA	1	9	NA	NA	NA

Skin-Melanoma	3433b1f3-59d1-4ac2-9bbf-e0c4042b4ddc	1	145553426	145555391	9	1965	NA	5	4	NA	NA	NA
Skin-Melanoma	3433b1f3-59d1-4ac2-9bbf-e0c4042b4ddc	1	152283425	152286374	20	2949	1	12	6	NA	1	NA
Skin-Melanoma	3433b1f3-59d1-4ac2-9bbf-e0c4042b4ddc	5	49752443	49754442	7	1999	NA	NA	7	NA	NA	NA
Skin-Melanoma	3433b1f3-59d1-4ac2-9bbf-e0c4042b4ddc	5	52104785	52108184	11	3399	1	7	3	NA	NA	NA
Skin-Melanoma	3433b1f3-59d1-4ac2-9bbf-e0c4042b4ddc	5	85794472	85795239	8	767	1	1	6	NA	NA	NA
Skin-Melanoma	3433b1f3-59d1-4ac2-9bbf-e0c4042b4ddc	6	63351584	63358273	24	6689	2	7	15	NA	NA	NA
Skin-Melanoma	3433b1f3-59d1-4ac2-9bbf-e0c4042b4ddc	8	16957675	16957960	7	285	3	4	NA	NA	NA	NA
Skin-Melanoma	3433b1f3-59d1-4ac2-9bbf-e0c4042b4ddc	8	104321043	104321844	10	801	2	NA	8	NA	NA	NA
Skin-Melanoma	3433b1f3-59d1-4ac2-9bbf-e0c4042b4ddc	11	59244894	59247877	13	2983	NA	NA	13	NA	NA	NA
Skin-Melanoma	3433b1f3-59d1-4ac2-9bbf-e0c4042b4ddc	11	78277095	78278866	8	1771	NA	5	2	NA	1	NA
Skin-Melanoma	3433b1f3-59d1-4ac2-9bbf-e0c4042b4ddc	11	79990358	79992630	13	2272	1	3	9	NA	NA	NA
Skin-Melanoma	3433b1f3-59d1-4ac2-9bbf-e0c4042b4ddc	11	83736076	83736488	7	412	NA	7	NA	NA	NA	NA
Skin-Melanoma	3433b1f3-59d1-4ac2-9bbf-e0c4042b4ddc	11	119842389	119843970	7	1581	3	NA	4	NA	NA	NA
Skin-Melanoma	3433b1f3-59d1-4ac2-9bbf-e0c4042b4ddc	18	39636477	39647104	22	10627	NA	2	19	1	NA	NA
Skin-Melanoma	3433b1f3-59d1-4ac2-9bbf-e0c4042b4ddc	18	44353719	44358675	8	4956	2	1	3	NA	NA	2
Skin-Melanoma	3433b1f3-59d1-4ac2-9bbf-e0c4042b4ddc	18	54258965	54259863	15	898	1	2	11	NA	NA	1
Skin-Melanoma	3433b1f3-59d1-4ac2-9bbf-e0c4042b4ddc	20	13485902	13486642	11	740	2	3	6	NA	NA	NA
Skin-Melanoma	3433b1f3-59d1-4ac2-9bbf-e0c4042b4ddc	22	40353575	40357434	8	3859	4	NA	1	NA	NA	3
Skin-Melanoma	3433b1f3-59d1-4ac2-9bbf-e0c4042b4ddc	X	98066236	98072151	8	5915	NA	NA	3	1	NA	4
Liver-HCC	343fcbc2-f30c-4ffa-a0b3-f5bb21e5e70b	5	8150690	8153720	7	3030	1	4	2	NA	NA	NA
Liver-HCC	343fcbc2-f30c-4ffa-a0b3-f5bb21e5e70b	19	20628562	20629397	6	835	NA	NA	2	2	1	1
Stomach-AdenoCA	34a445c2-1eb4-4a9f-8838-cddc2f82aae4	7	89514888	89515527	10	639	3	4	3	NA	NA	NA
Stomach-AdenoCA	34a445c2-1eb4-4a9f-8838-cddc2f82aae4	9	11007200	11012599	9	5399	1	NA	NA	1	2	5
Stomach-AdenoCA	34a445c2-1eb4-4a9f-8838-cddc2f82aae4	11	24268684	24274247	10	5563	NA	NA	NA	1	7	2
Kidney-RCC	34ab4c57-5240-4af7-a329-a5ab55934fd4	3	76631272	76632321	10	1049	3	5	2	NA	NA	NA
Skin-Melanoma	351db483-a70e-496d-b70a-7449875121ee	6	48519266	48520577	14	1311	NA	NA	14	NA	NA	NA
Skin-Melanoma	351db483-a70e-496d-b70a-7449875121ee	6	56011620	56012129	8	509	NA	7	1	NA	NA	NA
Skin-Melanoma	351db483-a70e-496d-b70a-7449875121ee	7	132176464	132176694	9	230	3	2	4	NA	NA	NA
Skin-Melanoma	351db483-a70e-496d-b70a-7449875121ee	7	133971920	133973224	10	1304	2	2	6	NA	NA	NA
Skin-Melanoma	351db483-a70e-496d-b70a-7449875121ee	7	134167707	134172354	12	4647	3	8	1	NA	NA	NA
Skin-Melanoma	351db483-a70e-496d-b70a-7449875121ee	7	134195163	134199251	9	4088	1	5	3	NA	NA	NA
Skin-Melanoma	351db483-a70e-496d-b70a-7449875121ee	7	134479122	134484696	13	5574	4	4	5	NA	NA	NA
Skin-Melanoma	351db483-a70e-496d-b70a-7449875121ee	11	59614955	59621490	28	6535	8	8	8	2	1	1
Skin-Melanoma	351db483-a70e-496d-b70a-7449875121ee	11	60046782	60047256	8	474	1	6	1	NA	NA	NA
Skin-Melanoma	351db483-a70e-496d-b70a-7449875121ee	11	60917247	60920104	8	2857	1	4	3	NA	NA	NA
Skin-Melanoma	351db483-a70e-496d-b70a-7449875121ee	11	64688220	64694103	7	5883	1	4	2	NA	NA	NA
Skin-Melanoma	351db483-a70e-496d-b70a-7449875121ee	11	69952563	69958447	18	5884	1	6	11	NA	NA	NA
Skin-Melanoma	351db483-a70e-496d-b70a-7449875121ee	11	70012213	70015495	8	3282	NA	3	5	NA	NA	NA
Skin-Melanoma	351db483-a70e-496d-b70a-7449875121ee	11	72414867	72416111	11	1244	NA	5	6	NA	NA	NA
Skin-Melanoma	351db483-a70e-496d-b70a-7449875121ee	11	74204431	74208967	7	4536	1	1	5	NA	NA	NA
Skin-Melanoma	351db483-a70e-496d-b70a-7449875121ee	11	76227059	76228882	20	1823	1	13	5	1	NA	NA
Skin-Melanoma	351db483-a70e-496d-b70a-7449875121ee	11	77878766	77880966	8	2200	1	4	3	NA	NA	NA
Skin-Melanoma	351db483-a70e-496d-b70a-7449875121ee	11	79418530	79419165	7	635	1	4	2	NA	NA	NA
Skin-Melanoma	351db483-a70e-496d-b70a-7449875121ee	11	93338331	93339834	18	1503	3	12	3	NA	NA	NA
Skin-Melanoma	351db483-a70e-496d-b70a-7449875121ee	11	100828950	100836428	9	7478	1	5	3	NA	NA	NA
Skin-Melanoma	351db483-a70e-496d-b70a-7449875121ee	11	102423845	102425038	9	1193	2	4	3	NA	NA	NA
Skin-Melanoma	351db483-a70e-496d-b70a-7449875121ee	11	103840691	103844616	11	3925	2	5	4	NA	NA	NA
Skin-Melanoma	351db483-a70e-496d-b70a-7449875121ee	11	104446515	104447425	7	910	1	4	2	NA	NA	NA
Skin-Melanoma	351db483-a70e-496d-b70a-7449875121ee	11	105254961	105255961	9	1000	1	4	4	NA	NA	NA
Skin-Melanoma	351db483-a70e-496d-b70a-7449875121ee	11	105708249	105709844	8	1595	1	1	6	NA	NA	NA
Skin-Melanoma	351db483-a70e-496d-b70a-7449875121ee	11	108447076	108447699	10	623	NA	4	6	NA	NA	NA
Skin-Melanoma	351db483-a70e-496d-b70a-7449875121ee	11	113864816	113866432	8	1616	NA	6	2	NA	NA	NA
Skin-Melanoma	351db483-a70e-496d-b70a-7449875121ee	11	114853488	114854147	17	659	3	11	3	NA	NA	NA
Skin-Melanoma	351db483-a70e-496d-b70a-7449875121ee	11	119165674	119166369	8	695	2	5	1	NA	NA	NA
Skin-Melanoma	351db483-a70e-496d-b70a-7449875121ee	20	31877101	31881290	8	4189	NA	NA	8	NA	NA	NA
Skin-Melanoma	351db483-a70e-496d-b70a-7449875121ee	20	47238838	47240076	7	1238	NA	NA	7	NA	NA	NA
Prost-AdenoCA	35553150-e4ef-4539-b220-259f2d634bd7	9	103373078	103377495	7	4417	NA	5	2	NA	NA	NA
Breast-AdenoCa	3585e133-b3c1-4d90-b5f2-2b867e0ae0ec	1	216935393	216942618	10	7225	NA	5	4	1	NA	NA
Breast-AdenoCa	3585e133-b3c1-4d90-b5f2-2b867e0ae0ec	6	51693922	51696762	9	2840	2	6	1	NA	NA	NA
Breast-AdenoCa	3585e133-b3c1-4d90-b5f2-2b867e0ae0ec	6	72643589	72644214	7	625	NA	NA	NA	4	3	NA

Breast-AdenoCa	3585e133-b3c1-4d90-b5f2-2b867e0ae0ec	8	38871838	38876379	12	4541	NA	8	4	NA	NA	NA
Breast-AdenoCa	3585e133-b3c1-4d90-b5f2-2b867e0ae0ec	8	39157879	39161454	16	3575	2	9	5	NA	NA	NA
Breast-AdenoCa	3585e133-b3c1-4d90-b5f2-2b867e0ae0ec	11	75778063	75780986	8	2923	NA	5	3	NA	NA	NA
Breast-AdenoCa	3585e133-b3c1-4d90-b5f2-2b867e0ae0ec	11	90367209	90372240	7	5031	NA	3	4	NA	NA	NA
Breast-AdenoCa	3585e133-b3c1-4d90-b5f2-2b867e0ae0ec	18	12050011	12050765	6	754	4	1	1	NA	NA	NA
Breast-AdenoCa	3585e133-b3c1-4d90-b5f2-2b867e0ae0ec	18	26957215	26962773	12	5558	3	6	3	NA	NA	NA
Breast-AdenoCa	3585e133-b3c1-4d90-b5f2-2b867e0ae0ec	18	26980856	26986033	8	5177	2	2	4	NA	NA	NA
Breast-AdenoCa	3585e133-b3c1-4d90-b5f2-2b867e0ae0ec	18	50924496	50927781	10	3285	1	7	2	NA	NA	NA
Breast-AdenoCa	3585e133-b3c1-4d90-b5f2-2b867e0ae0ec	22	51018331	51022841	6	4510	1	NA	4	NA	1	NA
Skin-Melanoma	35a74e53-16ff-4764-8397-6a9b02dfe733	5	8002271	8002599	6	328	1	1	4	NA	NA	NA
Skin-Melanoma	35a74e53-16ff-4764-8397-6a9b02dfe733	5	30935870	30942023	8	6153	1	3	3	NA	1	NA
Skin-Melanoma	35a74e53-16ff-4764-8397-6a9b02dfe733	5	30954325	30955282	10	957	1	1	8	NA	NA	NA
Skin-Melanoma	35a74e53-16ff-4764-8397-6a9b02dfe733	5	41884278	41885025	11	747	NA	2	9	NA	NA	NA
Skin-Melanoma	35a74e53-16ff-4764-8397-6a9b02dfe733	5	50276592	50286575	20	9983	2	10	8	NA	NA	NA
Skin-Melanoma	35a74e53-16ff-4764-8397-6a9b02dfe733	6	12117724	12121023	11	3299	2	4	5	NA	NA	NA
Skin-Melanoma	35a74e53-16ff-4764-8397-6a9b02dfe733	11	56014411	56019007	15	4596	2	9	4	NA	NA	NA
Skin-Melanoma	35a74e53-16ff-4764-8397-6a9b02dfe733	11	66858083	66859446	13	1363	2	2	9	NA	NA	NA
Skin-Melanoma	35a74e53-16ff-4764-8397-6a9b02dfe733	11	68219380	68227598	17	8218	1	NA	15	NA	1	NA
Skin-Melanoma	35a74e53-16ff-4764-8397-6a9b02dfe733	11	76588830	76590361	7	1531	1	2	4	NA	NA	NA
Skin-Melanoma	35a74e53-16ff-4764-8397-6a9b02dfe733	11	95745654	95752418	15	6764	2	5	8	NA	NA	NA
Skin-Melanoma	35a74e53-16ff-4764-8397-6a9b02dfe733	16	74538578	74542723	7	4145	NA	4	2	1	NA	NA
Bone-Leiomyo	35c797fd-ca81-4cef-b6c4-7e3776f661b3	1	193607565	193611046	12	3481	2	6	4	NA	NA	NA
Bone-Leiomyo	35c797fd-ca81-4cef-b6c4-7e3776f661b3	1	233343879	233345939	10	2060	2	5	3	NA	NA	NA
Bone-Leiomyo	35c797fd-ca81-4cef-b6c4-7e3776f661b3	3	72896630	72900624	9	3994	1	6	2	NA	NA	NA
Bone-Leiomyo	35c797fd-ca81-4cef-b6c4-7e3776f661b3	5	2960269	2961607	6	1338	NA	1	5	NA	NA	NA
Bone-Leiomyo	35c797fd-ca81-4cef-b6c4-7e3776f661b3	6	121478404	121494266	18	15862	5	6	7	NA	NA	NA
Bone-Leiomyo	35c797fd-ca81-4cef-b6c4-7e3776f661b3	7	85900444	85901088	7	644	1	2	4	NA	NA	NA
Bone-Leiomyo	35c797fd-ca81-4cef-b6c4-7e3776f661b3	7	103708823	103712173	18	3350	9	7	2	NA	NA	NA
Bone-Leiomyo	35c797fd-ca81-4cef-b6c4-7e3776f661b3	7	133851887	133852870	7	983	NA	2	5	NA	NA	NA
Bone-Leiomyo	35c797fd-ca81-4cef-b6c4-7e3776f661b3	7	137289303	137290410	11	1107	4	4	3	NA	NA	NA
Bone-Leiomyo	35c797fd-ca81-4cef-b6c4-7e3776f661b3	9	21590129	21591162	8	1033	2	6	NA	NA	NA	NA
Bone-Leiomyo	35c797fd-ca81-4cef-b6c4-7e3776f661b3	12	46936551	46939392	7	2841	4	2	1	NA	NA	NA
Bone-Leiomyo	35c797fd-ca81-4cef-b6c4-7e3776f661b3	12	47319061	47323364	9	4303	2	6	1	NA	NA	NA
Bone-Leiomyo	35c797fd-ca81-4cef-b6c4-7e3776f661b3	12	52199907	52205666	8	5759	NA	3	5	NA	NA	NA
Bone-Leiomyo	35c797fd-ca81-4cef-b6c4-7e3776f661b3	12	64197000	64197987	8	987	NA	6	2	NA	NA	NA
Bone-Leiomyo	35c797fd-ca81-4cef-b6c4-7e3776f661b3	12	64286787	64290091	11	3304	3	4	4	NA	NA	NA
Bone-Leiomyo	35c797fd-ca81-4cef-b6c4-7e3776f661b3	12	65727687	65730800	24	3113	7	10	7	NA	NA	NA
Bone-Leiomyo	35c797fd-ca81-4cef-b6c4-7e3776f661b3	12	69786454	69790193	8	3739	2	4	2	NA	NA	NA
Bone-Leiomyo	35c797fd-ca81-4cef-b6c4-7e3776f661b3	12	72659753	72661008	11	1255	NA	5	6	NA	NA	NA
Bone-Leiomyo	35c797fd-ca81-4cef-b6c4-7e3776f661b3	12	79177979	79183759	8	5780	2	2	4	NA	NA	NA
Bone-Leiomyo	35c797fd-ca81-4cef-b6c4-7e3776f661b3	12	79331360	79332118	8	758	1	4	3	NA	NA	NA
Bone-Leiomyo	35c797fd-ca81-4cef-b6c4-7e3776f661b3	12	93132928	93135755	9	2827	1	3	5	NA	NA	NA
Bone-Leiomyo	35c797fd-ca81-4cef-b6c4-7e3776f661b3	13	64087764	64090805	11	3041	6	4	1	NA	NA	NA
Bone-Leiomyo	35c797fd-ca81-4cef-b6c4-7e3776f661b3	15	79077611	79078795	6	1184	2	2	2	NA	NA	NA
Bone-Leiomyo	35c797fd-ca81-4cef-b6c4-7e3776f661b3	16	48589221	48596365	15	7144	7	3	5	NA	NA	NA
Bone-Leiomyo	35c797fd-ca81-4cef-b6c4-7e3776f661b3	19	37743700	37745049	6	1349	1	2	3	NA	NA	NA
Bone-Leiomyo	35c797fd-ca81-4cef-b6c4-7e3776f661b3	X	144476073	144484736	11	8663	2	5	3	NA	1	NA
Liver-HCC	35cca3b8-c623-11e3-bf01-24c6515278c0	1	240983185	240985216	7	2031	1	4	2	NA	NA	NA
Ovary-AdenoCA	35ceba07-0759-4fbe-b076-af821a528cf0	2	152089252	152089261	7	9	NA	NA	2	NA	1	4
Ovary-AdenoCA	35ceba07-0759-4fbe-b076-af821a528cf0	X	82395435	82395445	6	10	NA	NA	2	1	NA	3
Liver-HCC	35e33a7a-c622-11e3-bf01-24c6515278c0	4	32108913	32111129	6	2216	1	NA	2	1	1	1
Prost-AdenoCA	36962459-e81f-4b8c-a08d-df60ef3283a3	1	95700821	95704711	7	3890	2	1	4	NA	NA	NA
Prost-AdenoCA	36962459-e81f-4b8c-a08d-df60ef3283a3	1	100041691	100042103	7	412	1	2	4	NA	NA	NA
Prost-AdenoCA	36962459-e81f-4b8c-a08d-df60ef3283a3	8	22220045	22221816	7	1771	NA	4	3	NA	NA	NA
Lung-SCC	369c06f2-8904-49cb-99d1-dd297ed0cd0c	2	27661368	27664773	13	3405	3	2	8	NA	NA	NA
Lung-SCC	369c06f2-8904-49cb-99d1-dd297ed0cd0c	3	50142181	50151785	33	9604	14	6	13	NA	NA	NA
Lung-SCC	369c06f2-8904-49cb-99d1-dd297ed0cd0c	3	170732417	170735573	8	3156	1	3	4	NA	NA	NA
Lung-SCC	369c06f2-8904-49cb-99d1-dd297ed0cd0c	7	44696893	44699017	6	2124	NA	5	1	NA	NA	NA
Lung-SCC	369c06f2-8904-49cb-99d1-dd297ed0cd0c	10	120926935	120930764	12	3829	NA	3	9	NA	NA	NA
Lung-SCC	369c06f2-8904-49cb-99d1-dd297ed0cd0c	15	97742787	97755127	25	12340	5	9	11	NA	NA	NA

Lung-SCC	369c06f2-8904-49cb-99d1-dd297ed0cd0c	16	233752	237568	8	3816	2	3	2	NA	1	NA
Lung-SCC	369c06f2-8904-49cb-99d1-dd297ed0cd0c	18	20182223	20182898	10	675	1	3	6	NA	NA	NA
Lung-SCC	369c06f2-8904-49cb-99d1-dd297ed0cd0c	20	32394343	32395943	8	1600	NA	3	5	NA	NA	NA
Lung-SCC	369c06f2-8904-49cb-99d1-dd297ed0cd0c	X	95295252	95308215	14	12963	8	1	5	NA	NA	NA
Breast-AdenoCa	36d1a85e-a09b-4537-86e0-eaf1eb03aed8	15	26844965	26857792	14	12827	5	3	6	NA	NA	NA
Breast-AdenoCa	36d1a85e-a09b-4537-86e0-eaf1eb03aed8	15	32214285	32217089	10	2804	5	2	2	NA	NA	1
Liver-HCC	36d9c1aa-c623-11e3-bf01-24c6515278c0	5	162569718	162570149	6	431	NA	NA	6	NA	NA	NA
Liver-HCC	36d9c1aa-c623-11e3-bf01-24c6515278c0	18	3200005	3206569	24	6564	9	8	7	NA	NA	NA
Liver-HCC	36e1d9cc-32ec-4a0a-8fb1-c46f058a6fb8	12	19462262	19463954	6	1692	2	3	1	NA	NA	NA
Eso-AdenoCa	371235d9-55b8-40a7-8cd6-baa7c2ad0493	2	82682791	82683363	6	572	NA	NA	NA	NA	4	2
Eso-AdenoCa	371235d9-55b8-40a7-8cd6-baa7c2ad0493	5	99780565	99784988	6	4423	NA	NA	NA	2	1	3
Eso-AdenoCa	371235d9-55b8-40a7-8cd6-baa7c2ad0493	22	22291707	22293262	12	1555	NA	5	7	NA	NA	NA
CNS-GBM	374cbd87-428e-4509-85c1-b7d3302c30a0	8	128173904	128177243	6	3339	2	NA	2	1	NA	1
CNS-GBM	374cbd87-428e-4509-85c1-b7d3302c30a0	9	21048587	21052552	6	3965	NA	4	2	NA	NA	NA
CNS-GBM	374cbd87-428e-4509-85c1-b7d3302c30a0	9	24306938	24312098	7	5160	3	1	NA	2	NA	1
Ovary-AdenoCA	37522f18-77b2-4414-8df8-3c2c8048adba	2	7775075	7777408	7	2333	NA	1	5	1	NA	NA
Ovary-AdenoCA	37522f18-77b2-4414-8df8-3c2c8048adba	4	42616972	42621217	9	4245	3	2	4	NA	NA	NA
Ovary-AdenoCA	37522f18-77b2-4414-8df8-3c2c8048adba	5	50132813	50133346	8	533	NA	6	2	NA	NA	NA
Ovary-AdenoCA	37522f18-77b2-4414-8df8-3c2c8048adba	7	145379030	145381492	13	2462	2	8	3	NA	NA	NA
Ovary-AdenoCA	37522f18-77b2-4414-8df8-3c2c8048adba	10	36154184	36161642	22	7458	6	12	4	NA	NA	NA
Ovary-AdenoCA	37522f18-77b2-4414-8df8-3c2c8048adba	14	86424955	86426211	6	1256	1	2	3	NA	NA	NA
Ovary-AdenoCA	37522f18-77b2-4414-8df8-3c2c8048adba	17	18675419	18676687	9	1268	1	6	2	NA	NA	NA
Ovary-AdenoCA	37522f18-77b2-4414-8df8-3c2c8048adba	17	21411887	21414340	9	2453	NA	5	4	NA	NA	NA
Ovary-AdenoCA	37522f18-77b2-4414-8df8-3c2c8048adba	19	28624532	28630736	12	6204	2	7	2	1	NA	NA
Ovary-AdenoCA	37522f18-77b2-4414-8df8-3c2c8048adba	19	41973428	41975116	12	1688	3	2	7	NA	NA	NA
Eso-AdenoCa	384ef419-ee94-4563-9237-236fddeb4b64	9	15196835	15197362	7	527	NA	7	NA	NA	NA	NA
Panc-AdenoCA	3933c60d-73d6-4f74-ae02-fd545fc1f092	2	72879284	72879583	7	299	NA	4	2	NA	NA	1
Panc-AdenoCA	3933c60d-73d6-4f74-ae02-fd545fc1f092	2	147224673	147225421	12	748	4	5	3	NA	NA	NA
Panc-AdenoCA	3933c60d-73d6-4f74-ae02-fd545fc1f092	8	41425446	41427182	19	1736	5	4	10	NA	NA	NA
Panc-AdenoCA	3933c60d-73d6-4f74-ae02-fd545fc1f092	8	55101581	55102794	17	1213	6	5	6	NA	NA	NA
Panc-AdenoCA	3933c60d-73d6-4f74-ae02-fd545fc1f092	8	124814994	124817261	16	2267	3	8	5	NA	NA	NA
Panc-AdenoCA	3933c60d-73d6-4f74-ae02-fd545fc1f092	11	30959794	30963849	9	4055	3	6	NA	NA	NA	NA
Panc-AdenoCA	3933c60d-73d6-4f74-ae02-fd545fc1f092	12	25007354	25008813	9	1459	2	NA	5	1	NA	1
Panc-AdenoCA	3933c60d-73d6-4f74-ae02-fd545fc1f092	19	45950243	45952673	8	2430	3	NA	4	NA	NA	1
Panc-AdenoCA	3933c60d-73d6-4f74-ae02-fd545fc1f092	X	126764662	126765014	7	352	1	3	3	NA	NA	NA
CNS-GBM	39520be3-a2af-4189-acf4-9d239363333a	3	184781891	184786318	6	4427	NA	4	2	NA	NA	NA
CNS-GBM	39520be3-a2af-4189-acf4-9d239363333a	9	129997711	129998460	6	749	NA	4	2	NA	NA	NA
Prost-AdenoCA	39aee9fc-fa27-4b71-b9e8-43ff34bd3930	8	37842377	37846469	7	4092	2	3	2	NA	NA	NA
Prost-AdenoCA	39aee9fc-fa27-4b71-b9e8-43ff34bd3930	8	38154105	38155871	9	1766	NA	6	3	NA	NA	NA
Panc-AdenoCA	39c6c3b3-4683-4d11-984c-ed589188da65	1	101125725	101128375	12	2650	NA	6	6	NA	NA	NA
Panc-AdenoCA	39c6c3b3-4683-4d11-984c-ed589188da65	9	9954201	9955143	7	942	NA	5	2	NA	NA	NA
Panc-AdenoCA	39c6c3b3-4683-4d11-984c-ed589188da65	12	44766693	44772378	13	5685	1	4	7	NA	1	NA
Panc-AdenoCA	39c6c3b3-4683-4d11-984c-ed589188da65	12	49443217	49447724	15	4507	2	4	9	NA	NA	NA
Panc-AdenoCA	39c6c3b3-4683-4d11-984c-ed589188da65	12	60189434	60192886	9	3452	4	NA	4	1	NA	NA
Panc-AdenoCA	39c6c3b3-4683-4d11-984c-ed589188da65	15	45794289	45795168	7	879	1	1	5	NA	NA	NA
Panc-AdenoCA	39c6c3b3-4683-4d11-984c-ed589188da65	15	46575171	46586540	45	11369	6	12	27	NA	NA	NA
Panc-AdenoCA	39c6c3b3-4683-4d11-984c-ed589188da65	15	85423900	85427406	8	3506	NA	2	6	NA	NA	NA
Panc-AdenoCA	39c6c3b3-4683-4d11-984c-ed589188da65	17	16789759	16790168	9	409	3	1	5	NA	NA	NA
Panc-AdenoCA	39c6c3b3-4683-4d11-984c-ed589188da65	17	25431928	25434343	20	2415	2	5	13	NA	NA	NA
Panc-AdenoCA	39c6c3b3-4683-4d11-984c-ed589188da65	17	72523752	72524973	7	1221	NA	NA	7	NA	NA	NA
Panc-AdenoCA	39c6c3b3-4683-4d11-984c-ed589188da65	18	19017457	19020993	11	3536	4	3	3	NA	NA	1
Panc-AdenoCA	39c6c3b3-4683-4d11-984c-ed589188da65	19	21038053	21038098	22	45	6	NA	3	7	3	3
Panc-AdenoCA	39d38c06-3779-4ca4-b103-1d096f93dae8	3	90173801	90174725	8	924	NA	3	5	NA	NA	NA
Panc-AdenoCA	39d38c06-3779-4ca4-b103-1d096f93dae8	3	90247584	90250806	24	3222	1	10	13	NA	NA	NA
Panc-AdenoCA	39d38c06-3779-4ca4-b103-1d096f93dae8	4	161920528	161925557	16	5029	NA	1	12	NA	3	NA
Panc-AdenoCA	39d38c06-3779-4ca4-b103-1d096f93dae8	6	20777072	20777604	13	532	3	NA	10	NA	NA	NA
Panc-AdenoCA	39d38c06-3779-4ca4-b103-1d096f93dae8	6	33164675	33167489	16	2814	1	3	12	NA	NA	NA
Panc-AdenoCA	39d38c06-3779-4ca4-b103-1d096f93dae8	6	84638526	84644511	16	5985	NA	12	4	NA	NA	NA
Panc-AdenoCA	39d38c06-3779-4ca4-b103-1d096f93dae8	19	57687806	57689396	14	1590	NA	6	8	NA	NA	NA
Panc-AdenoCA	39d38c06-3779-4ca4-b103-1d096f93dae8	X	67518438	67520015	12	1577	2	4	6	NA	NA	NA

Eso-AdenoCa	3a18aa52-b6e2-4d03-ad05-ef99d653f0e0	17	33052210	33055415	6	3205	2	1	2	NA	NA	1
Eso-AdenoCa	3a551616-4840-4111-a8c5-b4aa773d82c2	6	94989855	94990991	7	1136	2	2	3	NA	NA	NA
Eso-AdenoCa	3a551616-4840-4111-a8c5-b4aa773d82c2	9	18407505	18409291	7	1786	NA	4	3	NA	NA	NA
Eso-AdenoCa	3a551616-4840-4111-a8c5-b4aa773d82c2	9	27100887	27101739	21	852	5	7	9	NA	NA	NA
Eso-AdenoCa	3a551616-4840-4111-a8c5-b4aa773d82c2	9	30113938	30115166	8	1228	1	1	5	NA	NA	1
Eso-AdenoCa	3a551616-4840-4111-a8c5-b4aa773d82c2	9	81385317	81386269	11	952	3	1	7	NA	NA	NA
Eso-AdenoCa	3a551616-4840-4111-a8c5-b4aa773d82c2	9	86660315	86661234	7	919	NA	5	2	NA	NA	NA
Eso-AdenoCa	3a551616-4840-4111-a8c5-b4aa773d82c2	9	93493470	93494200	7	730	3	NA	4	NA	NA	NA
Eso-AdenoCa	3a551616-4840-4111-a8c5-b4aa773d82c2	9	99566586	99567527	10	941	1	8	1	NA	NA	NA
Eso-AdenoCa	3a551616-4840-4111-a8c5-b4aa773d82c2	9	117834870	117835443	19	573	3	2	14	NA	NA	NA
Eso-AdenoCa	3a551616-4840-4111-a8c5-b4aa773d82c2	9	122507896	122509203	9	1307	NA	2	6	NA	NA	1
Eso-AdenoCa	3a551616-4840-4111-a8c5-b4aa773d82c2	9	138753266	138755047	21	1781	4	7	9	NA	NA	1
Eso-AdenoCa	3a551616-4840-4111-a8c5-b4aa773d82c2	17	38995553	38999498	9	3945	2	NA	7	NA	NA	NA
Ovary-AdenoCA	3a5eaed6-fcfd-437a-b61c-fd3725c64717	1	233451223	233455725	17	4502	2	6	9	NA	NA	NA
Ovary-AdenoCA	3a5eaed6-fcfd-437a-b61c-fd3725c64717	3	180734928	180738542	10	3614	1	4	5	NA	NA	NA
Ovary-AdenoCA	3a5eaed6-fcfd-437a-b61c-fd3725c64717	8	65401046	65403705	13	2659	NA	4	9	NA	NA	NA
Ovary-AdenoCA	3a6bce45-0431-49d8-82df-b1d9a738e5a6	8	136197115	136201634	17	4519	2	2	13	NA	NA	NA
Ovary-AdenoCA	3a6bce45-0431-49d8-82df-b1d9a738e5a6	8	138309670	138313285	10	3615	1	3	5	NA	NA	1
Ovary-AdenoCA	3a6bce45-0431-49d8-82df-b1d9a738e5a6	8	141412242	141413690	17	1448	5	4	8	NA	NA	NA
Ovary-AdenoCA	3a6bce45-0431-49d8-82df-b1d9a738e5a6	18	4581924	4582348	9	424	NA	3	5	NA	NA	1
Kidney-RCC	3afb3f57-545a-4ed5-9461-2fcb28d89399	1	113015362	113016430	8	1068	NA	4	4	NA	NA	NA
Kidney-RCC	3afb3f57-545a-4ed5-9461-2fcb28d89399	1	211733412	211735003	16	1591	1	NA	15	NA	NA	NA
Kidney-RCC	3afb3f57-545a-4ed5-9461-2fcb28d89399	3	154392515	154396789	8	4274	NA	2	6	NA	NA	NA
Kidney-RCC	3afb3f57-545a-4ed5-9461-2fcb28d89399	4	23638772	23639252	8	480	NA	NA	8	NA	NA	NA
Kidney-RCC	3afb3f57-545a-4ed5-9461-2fcb28d89399	4	24171435	24174036	10	2601	NA	NA	10	NA	NA	NA
Kidney-RCC	3afb3f57-545a-4ed5-9461-2fcb28d89399	7	75688136	75689019	6	883	NA	2	4	NA	NA	NA
Panc-AdenoCA	3b526846-72d6-4e10-b7cd-8cdb45a92a1c	2	127649858	127654254	12	4396	3	1	7	NA	1	NA
Panc-AdenoCA	3b526846-72d6-4e10-b7cd-8cdb45a92a1c	2	140755226	140761678	10	6452	NA	2	8	NA	NA	NA
Panc-AdenoCA	3b526846-72d6-4e10-b7cd-8cdb45a92a1c	10	131077631	131077812	8	181	NA	2	6	NA	NA	NA
Panc-AdenoCA	3b526846-72d6-4e10-b7cd-8cdb45a92a1c	21	35223928	35225858	16	1930	NA	3	13	NA	NA	NA
Lymph-BNHL	3b55c58d-f95a-4a1d-9aa1-8d2c026656a9	2	89158851	89160391	49	1540	NA	2	5	16	12	14
Lymph-BNHL	3b55c58d-f95a-4a1d-9aa1-8d2c026656a9	3	2219929	2225533	9	5604	NA	1	4	3	NA	1
Lymph-BNHL	3b55c58d-f95a-4a1d-9aa1-8d2c026656a9	9	106684473	106702824	21	18351	NA	NA	4	7	2	8
Lymph-BNHL	3b55c58d-f95a-4a1d-9aa1-8d2c026656a9	14	106325693	106329864	67	4171	4	15	15	13	9	11
Lymph-BNHL	3b55c58d-f95a-4a1d-9aa1-8d2c026656a9	14	106994186	106994767	16	581	1	1	3	6	2	3
Lymph-BNHL	3b55c58d-f95a-4a1d-9aa1-8d2c026656a9	18	60984081	60988383	61	4302	7	7	12	13	6	16
Kidney-RCC	3b7810f7-f8ff-4d62-b766-3ba06170194c	6	168590839	168590897	7	58	2	NA	2	NA	NA	3
Liver-HCC	3bacc189-01b8-46cc-a442-f393c0f428c6	11	58319281	58324439	9	5158	2	4	3	NA	NA	NA
Eso-AdenoCa	3bb4c27b-4d0d-4d13-b910-41a9ebf95880	1	31998769	32001687	15	2918	NA	NA	1	14	NA	NA
Eso-AdenoCa	3bb4c27b-4d0d-4d13-b910-41a9ebf95880	11	77836890	77839063	9	2173	2	1	6	NA	NA	NA
Eso-AdenoCa	3bb4c27b-4d0d-4d13-b910-41a9ebf95880	16	83462229	83483549	26	21320	13	5	6	NA	1	1
Panc-AdenoCA	3bfbc33d-804b-4bb3-97ac-97563d77b13d	5	179695259	179697746	10	2487	NA	6	4	NA	NA	NA
Panc-AdenoCA	3bfbc33d-804b-4bb3-97ac-97563d77b13d	8	41151766	41152229	6	463	NA	3	2	NA	NA	1
Panc-AdenoCA	3bfbc33d-804b-4bb3-97ac-97563d77b13d	10	27128906	27131105	18	2199	1	8	9	NA	NA	NA
Panc-AdenoCA	3bfbc33d-804b-4bb3-97ac-97563d77b13d	15	96560684	96572484	31	11800	1	16	13	NA	NA	1
Panc-AdenoCA	3bfbc33d-804b-4bb3-97ac-97563d77b13d	20	38803183	38808238	13	5055	6	3	4	NA	NA	NA
Panc-AdenoCA	3bfbc33d-804b-4bb3-97ac-97563d77b13d	X	126007927	126008299	11	372	1	NA	10	NA	NA	NA
Panc-AdenoCA	3bfbc33d-804b-4bb3-97ac-97563d77b13d	X	126181160	126186268	7	5108	NA	4	3	NA	NA	NA
Panc-AdenoCA	3bfbc33d-804b-4bb3-97ac-97563d77b13d	X	150910768	150912682	16	1914	NA	8	6	NA	1	1
ColoRect-AdenoCA	3c019b2f-52ec-40a8-99b5-98c1423ce627	19	42723129	42724191	9	1062	4	2	3	NA	NA	NA
Ovary-AdenoCA	3c2b1509-1eb9-4b79-9569-57810f291499	6	1616366	1618960	6	2594	1	3	2	NA	NA	NA
Ovary-AdenoCA	3c2b1509-1eb9-4b79-9569-57810f291499	6	2344203	2348645	10	4442	2	NA	7	1	NA	NA
Ovary-AdenoCA	3c2b1509-1eb9-4b79-9569-57810f291499	6	4864959	4869257	11	4298	NA	5	6	NA	NA	NA
Ovary-AdenoCA	3c2b1509-1eb9-4b79-9569-57810f291499	6	6303554	6309763	12	6209	NA	7	5	NA	NA	NA
Ovary-AdenoCA	3c2b1509-1eb9-4b79-9569-57810f291499	6	6399490	6408726	12	9236	1	3	7	1	NA	NA
Ovary-AdenoCA	3c2b1509-1eb9-4b79-9569-57810f291499	6	16112621	16116065	22	3444	2	13	7	NA	NA	NA
Ovary-AdenoCA	3c2b1509-1eb9-4b79-9569-57810f291499	6	16935721	16943642	15	7921	1	8	5	NA	NA	1
Ovary-AdenoCA	3c2b1509-1eb9-4b79-9569-57810f291499	6	19527068	19528471	8	1403	NA	5	3	NA	NA	NA
Ovary-AdenoCA	3c2b1509-1eb9-4b79-9569-57810f291499	6	20257687	20260135	9	2448	1	4	4	NA	NA	NA
Ovary-AdenoCA	3c2b1509-1eb9-4b79-9569-57810f291499	6	20596736	20604106	18	7370	NA	8	10	NA	NA	NA

Ovary-AdenoCA	3c2b1509-1eb9-4b79-9569-57810f291499	6	22500126	22511210	19	11084	2	4	13	NA	NA	NA
Panc-AdenoCA	3c86ba21-7b11-4ec7-9d20-a2325197c676	1	204475778	204476175	7	397	NA	2	5	NA	NA	NA
Panc-AdenoCA	3c86ba21-7b11-4ec7-9d20-a2325197c676	10	77686995	77693160	8	6165	NA	2	5	NA	NA	1
Panc-AdenoCA	3c86ba21-7b11-4ec7-9d20-a2325197c676	11	18426525	18426874	7	349	5	NA	2	NA	NA	NA
Panc-AdenoCA	3c86ba21-7b11-4ec7-9d20-a2325197c676	11	80100959	80101119	7	160	1	2	4	NA	NA	NA
Panc-AdenoCA	3c86ba21-7b11-4ec7-9d20-a2325197c676	11	80571146	80574178	14	3032	NA	4	10	NA	NA	NA
Panc-AdenoCA	3c86ba21-7b11-4ec7-9d20-a2325197c676	12	38354791	38355395	6	604	NA	NA	6	NA	NA	NA
Panc-AdenoCA	3c86ba21-7b11-4ec7-9d20-a2325197c676	19	38403287	38404047	7	760	1	2	4	NA	NA	NA
Bone-Leiomyo	3c963890-6e79-4b16-a4aa-bac04938b4d7	4	133562268	133563060	6	792	2	1	3	NA	NA	NA
Bone-Leiomyo	3c963890-6e79-4b16-a4aa-bac04938b4d7	5	101645079	101646560	6	1481	3	NA	3	NA	NA	NA
Bone-Leiomyo	3c963890-6e79-4b16-a4aa-bac04938b4d7	5	131924739	131933288	13	8549	2	3	8	NA	NA	NA
Bone-Leiomyo	3c963890-6e79-4b16-a4aa-bac04938b4d7	6	135076815	135079316	6	2501	1	1	3	NA	NA	1
Bone-Leiomyo	3c963890-6e79-4b16-a4aa-bac04938b4d7	6	135942556	135944420	7	1864	1	3	3	NA	NA	NA
Bone-Leiomyo	3c963890-6e79-4b16-a4aa-bac04938b4d7	9	26603196	26605290	6	2094	2	NA	3	NA	NA	1
Bone-Leiomyo	3c963890-6e79-4b16-a4aa-bac04938b4d7	11	59743273	59744325	6	1052	1	5	NA	NA	NA	NA
Bone-Leiomyo	3c963890-6e79-4b16-a4aa-bac04938b4d7	12	29144472	29146048	6	1576	3	3	NA	NA	NA	NA
Bone-Leiomyo	3c963890-6e79-4b16-a4aa-bac04938b4d7	12	69706335	69707940	8	1605	3	1	4	NA	NA	NA
Bone-Leiomyo	3c963890-6e79-4b16-a4aa-bac04938b4d7	12	71303087	71303873	7	786	1	2	4	NA	NA	NA
Bone-Leiomyo	3c963890-6e79-4b16-a4aa-bac04938b4d7	12	71518667	71520240	7	1573	1	2	4	NA	NA	NA
Bone-Leiomyo	3c963890-6e79-4b16-a4aa-bac04938b4d7	12	74079845	74082633	11	2788	2	5	4	NA	NA	NA
Bone-Leiomyo	3c963890-6e79-4b16-a4aa-bac04938b4d7	12	74856310	74857859	7	1549	1	2	4	NA	NA	NA
Bone-Leiomyo	3c963890-6e79-4b16-a4aa-bac04938b4d7	12	77187514	77190013	9	2499	NA	7	2	NA	NA	NA
Bone-Leiomyo	3c963890-6e79-4b16-a4aa-bac04938b4d7	12	78513218	78518566	15	5348	NA	7	8	NA	NA	NA
Bone-Leiomyo	3c963890-6e79-4b16-a4aa-bac04938b4d7	12	83456099	83459012	15	2913	3	8	4	NA	NA	NA
Bone-Leiomyo	3c963890-6e79-4b16-a4aa-bac04938b4d7	12	98487641	98492135	12	4494	2	6	4	NA	NA	NA
Bone-Leiomyo	3c963890-6e79-4b16-a4aa-bac04938b4d7	12	110755708	110759325	7	3617	2	1	4	NA	NA	NA
Bone-Leiomyo	3c963890-6e79-4b16-a4aa-bac04938b4d7	12	113458688	113461631	8	2943	1	4	3	NA	NA	NA
Bone-Leiomyo	3c963890-6e79-4b16-a4aa-bac04938b4d7	18	44632104	44632259	6	155	NA	1	5	NA	NA	NA
Eso-AdenoCa	3da169e8-844a-4eee-b794-a2eb81db779a	4	121630038	121630280	10	242	1	9	NA	NA	NA	NA
Eso-AdenoCa	3da169e8-844a-4eee-b794-a2eb81db779a	18	30889999	30893373	8	3374	NA	3	5	NA	NA	NA
Stomach-AdenoCA	3db3b7b1-da1d-4b9c-a92a-c60fecf4328c	13	89211214	89212549	6	1335	NA	4	2	NA	NA	NA
Stomach-AdenoCA	3db3b7b1-da1d-4b9c-a92a-c60fecf4328c	16	26345174	26345484	7	310	1	2	4	NA	NA	NA
Stomach-AdenoCA	3db3b7b1-da1d-4b9c-a92a-c60fecf4328c	19	11740238	11744429	6	4191	NA	2	4	NA	NA	NA
Stomach-AdenoCA	3db3b7b1-da1d-4b9c-a92a-c60fecf4328c	19	30467290	30473207	25	5917	1	7	15	NA	1	1
Stomach-AdenoCA	3db3b7b1-da1d-4b9c-a92a-c60fecf4328c	19	47443910	47449902	32	5992	6	11	15	NA	NA	NA
Stomach-AdenoCA	3db3b7b1-da1d-4b9c-a92a-c60fecf4328c	X	62516049	62517222	9	1173	NA	8	1	NA	NA	NA
Lymph-BNHL	3e012b50-06d1-4120-971b-5e54139b00ee	1	198714040	198716117	7	2077	NA	NA	NA	NA	3	4
Lymph-BNHL	3e012b50-06d1-4120-971b-5e54139b00ee	1	217017619	217025775	11	8156	3	NA	NA	3	2	3
Lymph-BNHL	3e012b50-06d1-4120-971b-5e54139b00ee	2	24945043	24945148	9	105	NA	1	8	NA	NA	NA
Lymph-BNHL	3e012b50-06d1-4120-971b-5e54139b00ee	2	88905813	88910425	11	4612	2	2	3	1	1	2
Lymph-BNHL	3e012b50-06d1-4120-971b-5e54139b00ee	2	89127507	89159779	131	32272	4	18	36	32	21	20
Lymph-BNHL	3e012b50-06d1-4120-971b-5e54139b00ee	2	136873922	136876061	7	2139	NA	NA	6	NA	NA	1
Lymph-BNHL	3e012b50-06d1-4120-971b-5e54139b00ee	3	6713584	6719332	7	5748	1	NA	1	NA	2	3
Lymph-BNHL	3e012b50-06d1-4120-971b-5e54139b00ee	3	186711794	186715169	8	3375	NA	1	3	2	2	NA
Lymph-BNHL	3e012b50-06d1-4120-971b-5e54139b00ee	3	186782447	186788674	10	6227	NA	NA	6	3	1	NA
Lymph-BNHL	3e012b50-06d1-4120-971b-5e54139b00ee	3	187461767	187474551	72	12784	4	9	27	18	6	8
Lymph-BNHL	3e012b50-06d1-4120-971b-5e54139b00ee	3	187957353	187961527	32	4174	1	7	6	10	2	6
Lymph-BNHL	3e012b50-06d1-4120-971b-5e54139b00ee	3	188298958	188299476	9	518	1	3	4	1	NA	NA
Lymph-BNHL	3e012b50-06d1-4120-971b-5e54139b00ee	3	188469814	188471938	15	2124	NA	2	6	3	2	2
Lymph-BNHL	3e012b50-06d1-4120-971b-5e54139b00ee	4	25863890	25865500	6	1610	NA	1	2	NA	2	1
Lymph-BNHL	3e012b50-06d1-4120-971b-5e54139b00ee	4	30129157	30133598	7	4441	NA	NA	3	2	NA	2
Lymph-BNHL	3e012b50-06d1-4120-971b-5e54139b00ee	4	63326109	63328420	7	2311	NA	NA	2	1	1	3
Lymph-BNHL	3e012b50-06d1-4120-971b-5e54139b00ee	4	153242204	153245911	8	3707	NA	6	2	NA	NA	NA
Lymph-BNHL	3e012b50-06d1-4120-971b-5e54139b00ee	5	100618856	100624397	7	5541	NA	1	NA	2	2	2
Lymph-BNHL	3e012b50-06d1-4120-971b-5e54139b00ee	5	167006080	167011958	7	5878	1	NA	2	1	NA	3
Lymph-BNHL	3e012b50-06d1-4120-971b-5e54139b00ee	6	31548692	31550099	19	1407	3	1	12	1	2	NA
Lymph-BNHL	3e012b50-06d1-4120-971b-5e54139b00ee	6	89338361	89348829	18	10468	NA	5	2	3	2	6
Lymph-BNHL	3e012b50-06d1-4120-971b-5e54139b00ee	7	73981716	73985005	6	3289	1	2	2	1	NA	NA
Lymph-BNHL	3e012b50-06d1-4120-971b-5e54139b00ee	9	37002538	37005072	6	2534	NA	NA	2	NA	1	3
Lymph-BNHL	3e012b50-06d1-4120-971b-5e54139b00ee	9	37024052	37027330	7	3278	1	4	2	NA	NA	NA

Lymph-BNHL	3e012b50-06d1-4120-971b-5e54139b00ee	9	37369269	37371818	11	2549	NA	3	6	1	1	NA
Lymph-BNHL	3e012b50-06d1-4120-971b-5e54139b00ee	9	37399678	37401368	7	1690	NA	1	6	NA	NA	NA
Lymph-BNHL	3e012b50-06d1-4120-971b-5e54139b00ee	11	81571664	81575532	8	3868	NA	NA	1	6	1	NA
Lymph-BNHL	3e012b50-06d1-4120-971b-5e54139b00ee	12	122457636	122465630	33	7994	1	4	20	4	2	2
Lymph-BNHL	3e012b50-06d1-4120-971b-5e54139b00ee	13	74600780	74604275	6	3495	1	NA	NA	2	1	2
Lymph-BNHL	3e012b50-06d1-4120-971b-5e54139b00ee	14	69257343	69262502	11	5159	NA	2	7	1	1	NA
Lymph-BNHL	3e012b50-06d1-4120-971b-5e54139b00ee	14	106048790	106055141	13	6351	2	3	7	1	NA	NA
Lymph-BNHL	3e012b50-06d1-4120-971b-5e54139b00ee	14	106213738	106214681	8	943	1	1	5	NA	NA	1
Lymph-BNHL	3e012b50-06d1-4120-971b-5e54139b00ee	14	106326843	106493674	170	2E+05	13	27	70	27	17	16
Lymph-BNHL	3e012b50-06d1-4120-971b-5e54139b00ee	14	107176215	107179707	11	3492	1	1	4	3	1	1
Lymph-BNHL	3e012b50-06d1-4120-971b-5e54139b00ee	15	86245026	86247887	10	2861	NA	2	1	2	2	3
Lymph-BNHL	3e012b50-06d1-4120-971b-5e54139b00ee	16	10971979	10982892	17	10913	2	1	14	NA	NA	NA
Lymph-BNHL	3e012b50-06d1-4120-971b-5e54139b00ee	16	11349018	11349826	8	808	NA	3	5	NA	NA	NA
Lymph-BNHL	3e012b50-06d1-4120-971b-5e54139b00ee	16	46651758	46654989	8	3231	NA	1	5	2	NA	NA
Lymph-BNHL	3e012b50-06d1-4120-971b-5e54139b00ee	16	85932116	85945811	31	13695	2	3	17	6	1	2
Lymph-BNHL	3e012b50-06d1-4120-971b-5e54139b00ee	17	56408630	56409751	9	1121	NA	NA	8	1	NA	NA
Lymph-BNHL	3e012b50-06d1-4120-971b-5e54139b00ee	19	10304652	10309047	8	4395	1	3	3	1	NA	NA
Lymph-BNHL	3e012b50-06d1-4120-971b-5e54139b00ee	21	36525548	36528984	7	3436	NA	NA	NA	5	NA	2
Lymph-BNHL	3e012b50-06d1-4120-971b-5e54139b00ee	22	23154422	23155710	6	1288	1	1	3	1	NA	NA
Lymph-BNHL	3e012b50-06d1-4120-971b-5e54139b00ee	22	23192439	23470664	304	3E+05	14	38	##	53	36	36
Lymph-BNHL	3e012b50-06d1-4120-971b-5e54139b00ee	X	12993410	12996899	10	3489	NA	1	6	3	NA	NA
Lymph-BNHL	3e012b50-06d1-4120-971b-5e54139b00ee	X	125614397	125619202	7	4805	1	NA	NA	2	3	1
Liver-HCC	3e68233a-c623-11e3-bf01-24c6515278c0	17	55245075	55246169	15	1094	1	10	4	NA	NA	NA
Lymph-BNHL	3e7ccab5-5b1d-4147-b907-77cab8f0837e	2	76429550	76440621	13	11071	NA	1	2	5	3	2
Lymph-BNHL	3e7ccab5-5b1d-4147-b907-77cab8f0837e	6	89652364	89652510	7	146	NA	NA	NA	4	2	1
Lymph-BNHL	3e7ccab5-5b1d-4147-b907-77cab8f0837e	7	112969852	112971465	6	1613	1	1	1	1	1	1
Lymph-BNHL	3e7ccab5-5b1d-4147-b907-77cab8f0837e	8	92486403	92488719	6	2316	NA	NA	NA	NA	1	5
Lymph-BNHL	3e7ccab5-5b1d-4147-b907-77cab8f0837e	13	88415659	88416029	7	370	1	NA	1	2	2	1
Lymph-BNHL	3e7ccab5-5b1d-4147-b907-77cab8f0837e	14	106112754	106113459	10	705	1	2	7	NA	NA	NA
Lymph-BNHL	3e7ccab5-5b1d-4147-b907-77cab8f0837e	14	106209069	106213827	13	4758	1	1	8	NA	2	1
Lymph-BNHL	3e7ccab5-5b1d-4147-b907-77cab8f0837e	14	106239637	106241718	23	2081	3	2	17	1	NA	NA
Lymph-BNHL	3e7ccab5-5b1d-4147-b907-77cab8f0837e	14	106322758	106330585	102	7827	7	28	49	6	6	6
Lymph-BNHL	3e7ccab5-5b1d-4147-b907-77cab8f0837e	14	106691933	106692417	22	484	1	6	6	2	5	2
Lymph-BNHL	3e7ccab5-5b1d-4147-b907-77cab8f0837e	18	52456090	52460681	6	4591	NA	1	2	3	NA	NA
Lymph-BNHL	3e7ccab5-5b1d-4147-b907-77cab8f0837e	18	60984855	60988246	30	3391	2	7	6	4	6	5
Lymph-BNHL	3e7ccab5-5b1d-4147-b907-77cab8f0837e	22	23100392	23101444	6	1052	NA	1	2	2	NA	1
Lymph-BNHL	3e7ccab5-5b1d-4147-b907-77cab8f0837e	X	106128686	106132321	8	3635	NA	NA	3	1	2	2
Lymph-CLL	3e8a2c90-e747-4a22-bc9e-0b062479fec2	2	89158028	89185571	117	27543	6	19	15	41	24	12
Lymph-CLL	3e8a2c90-e747-4a22-bc9e-0b062479fec2	14	106239146	106241640	6	2494	NA	NA	2	4	NA	NA
Lymph-CLL	3e8a2c90-e747-4a22-bc9e-0b062479fec2	14	106323872	106376431	71	52559	2	15	20	24	4	6
Lymph-CLL	3e8a2c90-e747-4a22-bc9e-0b062479fec2	14	106641764	106642447	13	683	2	2	7	2	NA	NA
Lymph-CLL	3e8a2c90-e747-4a22-bc9e-0b062479fec2	22	23223155	23233126	25	9971	NA	5	10	8	2	NA
Lymph-BNHL	3e94aa64-7dd3-4d63-a5de-b4050d3dfafa	2	89159067	89161014	55	1947	NA	13	7	18	5	12
Lymph-BNHL	3e94aa64-7dd3-4d63-a5de-b4050d3dfafa	9	37293264	37293460	6	196	NA	NA	NA	5	1	NA
Lymph-BNHL	3e94aa64-7dd3-4d63-a5de-b4050d3dfafa	14	106323193	106330394	63	7201	NA	13	17	16	5	12
Lymph-BNHL	3e94aa64-7dd3-4d63-a5de-b4050d3dfafa	18	60984948	60988381	12	3433	1	1	8	1	1	NA
Lymph-BNHL	3e94aa64-7dd3-4d63-a5de-b4050d3dfafa	22	23222995	23232292	22	9297	NA	5	10	2	2	3
Skin-Melanoma	3f98d326-5676-4257-9af8-0a5f5d3c2527	8	55434628	55445826	15	11198	1	4	10	NA	NA	NA
Skin-Melanoma	3f98d326-5676-4257-9af8-0a5f5d3c2527	9	35367392	35369014	10	1622	1	2	7	NA	NA	NA
Skin-Melanoma	3f98d326-5676-4257-9af8-0a5f5d3c2527	11	73435845	73436972	7	1127	1	1	5	NA	NA	NA
Skin-Melanoma	3f98d326-5676-4257-9af8-0a5f5d3c2527	11	73466034	73473378	9	7344	NA	5	3	NA	NA	1
Skin-Melanoma	3f98d326-5676-4257-9af8-0a5f5d3c2527	11	74042660	74050487	10	7827	2	NA	8	NA	NA	NA
Skin-Melanoma	3f98d326-5676-4257-9af8-0a5f5d3c2527	11	79065639	79067932	8	2293	3	1	4	NA	NA	NA
Skin-Melanoma	3f98d326-5676-4257-9af8-0a5f5d3c2527	11	86674675	86676805	7	2130	NA	3	4	NA	NA	NA
Skin-Melanoma	3f98d326-5676-4257-9af8-0a5f5d3c2527	11	87297222	87299650	13	2428	NA	2	11	NA	NA	NA
Skin-Melanoma	3f98d326-5676-4257-9af8-0a5f5d3c2527	11	88972144	88972583	7	439	NA	3	4	NA	NA	NA
Skin-Melanoma	3f98d326-5676-4257-9af8-0a5f5d3c2527	11	89020699	89022597	8	1898	NA	2	6	NA	NA	NA
Skin-Melanoma	3f98d326-5676-4257-9af8-0a5f5d3c2527	11	101606209	101606994	11	785	3	4	3	NA	NA	1
Skin-Melanoma	3f98d326-5676-4257-9af8-0a5f5d3c2527	15	55653475	55655660	17	2185	4	9	4	NA	NA	NA
Skin-Melanoma	3f98d326-5676-4257-9af8-0a5f5d3c2527	18	37554282	37558198	14	3916	1	4	8	NA	1	NA

Skin-Melanoma	3f98d326-5676-4257-9af8-0a5f5d3c2527	18	42499437	42500204	9	767	NA	NA	9	NA	NA	NA
Skin-Melanoma	3f98d326-5676-4257-9af8-0a5f5d3c2527	18	43550740	43551667	8	927	NA	NA	8	NA	NA	NA
Skin-Melanoma	3f98d326-5676-4257-9af8-0a5f5d3c2527	18	48060086	48061137	13	1051	1	1	11	NA	NA	NA
Skin-Melanoma	3f98d326-5676-4257-9af8-0a5f5d3c2527	18	54035159	54040039	7	4880	NA	NA	6	NA	1	NA
Skin-Melanoma	3f98d326-5676-4257-9af8-0a5f5d3c2527	18	59847817	59849523	8	1706	NA	1	7	NA	NA	NA
Skin-Melanoma	3f98d326-5676-4257-9af8-0a5f5d3c2527	18	64247598	64249102	9	1504	1	NA	8	NA	NA	NA
ColoRect-AdenoCA	4019c219-c51e-479f-8a9d-cfa6816ed696	17	29001055	29011948	16	10893	5	3	7	1	NA	NA
Panc-AdenoCA	40378b7a-a65d-4510-aaba-a34c4058fac7	12	52157540	52159284	7	1744	NA	3	4	NA	NA	NA
Panc-AdenoCA	40378b7a-a65d-4510-aaba-a34c4058fac7	17	45998203	46000752	11	2549	1	3	7	NA	NA	NA
Panc-AdenoCA	40378b7a-a65d-4510-aaba-a34c4058fac7	19	21883160	21884337	12	1177	1	4	7	NA	NA	NA
Liver-HCC	41840dc1-5ea2-4f01-a0d4-8b65add641c8	5	42174992	42175444	6	452	1	5	NA	NA	NA	NA
CNS-GBM	41d50a3c-49fc-4d8a-9c25-bbbcd3a5da6f	3	109466098	109472031	16	5933	1	13	2	NA	NA	NA
CNS-GBM	41d50a3c-49fc-4d8a-9c25-bbbcd3a5da6f	9	23970394	23972988	6	2594	1	2	3	NA	NA	NA
CNS-GBM	41d50a3c-49fc-4d8a-9c25-bbbcd3a5da6f	22	24038005	24045469	12	7464	5	3	4	NA	NA	NA
CNS-GBM	41d50a3c-49fc-4d8a-9c25-bbbcd3a5da6f	22	29130280	29131190	7	910	NA	2	4	NA	NA	1
Lymph-BNHL	41dba8d1-8aad-474c-a82b-20b28ab818bd	1	27621122	27621331	6	209	NA	NA	NA	3	1	2
Lymph-BNHL	41dba8d1-8aad-474c-a82b-20b28ab818bd	8	113982800	113986767	6	3967	2	NA	NA	3	1	NA
Lymph-BNHL	41dba8d1-8aad-474c-a82b-20b28ab818bd	10	76373689	76373831	6	142	NA	NA	NA	1	3	2
Lymph-BNHL	41dba8d1-8aad-474c-a82b-20b28ab818bd	12	122459335	122462015	7	2680	2	1	2	1	NA	1
Lymph-BNHL	41dba8d1-8aad-474c-a82b-20b28ab818bd	14	106209666	106213684	20	4018	3	6	10	1	NA	NA
Lymph-BNHL	41dba8d1-8aad-474c-a82b-20b28ab818bd	14	106239548	106240704	9	1156	NA	4	5	NA	NA	NA
Lymph-BNHL	41dba8d1-8aad-474c-a82b-20b28ab818bd	14	106326646	106330263	86	3617	5	18	34	9	13	7
Lymph-BNHL	41dba8d1-8aad-474c-a82b-20b28ab818bd	14	106641753	106642538	16	785	NA	5	7	2	2	NA
Lymph-BNHL	41dba8d1-8aad-474c-a82b-20b28ab818bd	18	60983306	60988649	17	5343	2	2	6	3	2	2
Lymph-BNHL	41dba8d1-8aad-474c-a82b-20b28ab818bd	22	23040628	23041630	7	1002	NA	2	2	2	NA	1
Lymph-BNHL	41dba8d1-8aad-474c-a82b-20b28ab818bd	22	23055025	23063375	16	8350	2	6	3	NA	4	1
Liver-HCC	41de507a-c623-11e3-bf01-24c6515278c0	1	177764645	177764711	6	66	NA	NA	NA	2	4	NA
Breast-AdenoCa	41f140b8-72b9-4ae3-8b25-6144d9f94d0f	1	28913577	28918102	6	4525	2	NA	4	NA	NA	NA
Breast-AdenoCa	41f140b8-72b9-4ae3-8b25-6144d9f94d0f	1	161886286	161886499	7	213	NA	4	3	NA	NA	NA
Prost-AdenoCA	42a548f0-d86a-4273-8bda-be0fe6a53b20	6	26189122	26189215	13	93	2	NA	5	5	1	NA
Panc-AdenoCA	42f00950-09c9-461a-8423-d5b04a96af10	7	139913785	139914358	7	573	2	2	3	NA	NA	NA
Panc-AdenoCA	42f00950-09c9-461a-8423-d5b04a96af10	7	146973139	146974257	7	1118	1	3	3	NA	NA	NA
Panc-AdenoCA	42f00950-09c9-461a-8423-d5b04a96af10	12	31692222	31692785	7	563	2	3	2	NA	NA	NA
Panc-AdenoCA	42f00950-09c9-461a-8423-d5b04a96af10	12	53177233	53182524	7	5291	1	1	5	NA	NA	NA
Panc-AdenoCA	42f00950-09c9-461a-8423-d5b04a96af10	12	54750224	54752042	7	1818	1	2	4	NA	NA	NA
Panc-AdenoCA	42f00950-09c9-461a-8423-d5b04a96af10	12	57076561	57078277	16	1716	1	4	11	NA	NA	NA
Panc-AdenoCA	42f00950-09c9-461a-8423-d5b04a96af10	12	57496333	57498995	14	2662	4	3	7	NA	NA	NA
Panc-AdenoCA	42f00950-09c9-461a-8423-d5b04a96af10	12	59042419	59049428	16	7009	3	6	7	NA	NA	NA
Panc-AdenoCA	42f00950-09c9-461a-8423-d5b04a96af10	12	59610166	59614483	19	4317	3	5	11	NA	NA	NA
Panc-AdenoCA	42f00950-09c9-461a-8423-d5b04a96af10	12	59749829	59753157	21	3328	5	3	13	NA	NA	NA
Panc-AdenoCA	42f00950-09c9-461a-8423-d5b04a96af10	12	61416804	61418121	8	1317	2	NA	6	NA	NA	NA
Panc-AdenoCA	42f00950-09c9-461a-8423-d5b04a96af10	12	62147158	62152788	8	5630	NA	4	4	NA	NA	NA
Panc-AdenoCA	42f00950-09c9-461a-8423-d5b04a96af10	12	63301715	63312892	13	11177	1	3	8	NA	1	NA
Panc-AdenoCA	42f00950-09c9-461a-8423-d5b04a96af10	19	39381438	39382954	7	1516	2	1	4	NA	NA	NA
Panc-AdenoCA	42f00950-09c9-461a-8423-d5b04a96af10	X	116544840	116545161	6	321	1	2	3	NA	NA	NA
Liver-HCC	43206cb7-787d-4f68-8019-55e8585c4b4d	1	244044716	244045600	10	884	1	7	2	NA	NA	NA
Lung-SCC	43aa4123-0fe2-4071-8a81-cbdd0410b917	1	34532736	34542721	11	9985	1	2	7	NA	NA	1
Lung-SCC	43aa4123-0fe2-4071-8a81-cbdd0410b917	1	72833685	72837753	8	4068	1	2	5	NA	NA	NA
Lung-SCC	43aa4123-0fe2-4071-8a81-cbdd0410b917	1	72845146	72848659	8	3513	1	3	4	NA	NA	NA
Lung-SCC	43aa4123-0fe2-4071-8a81-cbdd0410b917	1	90191100	90193884	9	2784	3	1	5	NA	NA	NA
Lung-SCC	43aa4123-0fe2-4071-8a81-cbdd0410b917	1	191951665	191954545	9	2880	NA	1	8	NA	NA	NA
Lung-SCC	43aa4123-0fe2-4071-8a81-cbdd0410b917	9	22401664	22403626	16	1962	NA	1	15	NA	NA	NA
Lung-SCC	43aa4123-0fe2-4071-8a81-cbdd0410b917	9	92729993	92733351	14	3358	NA	1	13	NA	NA	NA
Lung-SCC	43aa4123-0fe2-4071-8a81-cbdd0410b917	9	113967313	113971905	15	4592	2	4	9	NA	NA	NA
Lung-SCC	43aa4123-0fe2-4071-8a81-cbdd0410b917	9	118123359	118125713	22	2354	4	13	4	NA	1	NA
Lung-SCC	43aa4123-0fe2-4071-8a81-cbdd0410b917	17	19908402	19911036	10	2634	NA	3	7	NA	NA	NA
Lung-SCC	43aa4123-0fe2-4071-8a81-cbdd0410b917	19	19637523	19637756	6	233	NA	4	2	NA	NA	NA
Cervix-SCC	43be43cb-a367-4a74-94e4-973acafcf576	1	33632406	33635516	6	3110	1	NA	5	NA	NA	NA
Cervix-SCC	43be43cb-a367-4a74-94e4-973acafcf576	2	32555421	32558225	12	2804	1	3	7	NA	NA	1
Cervix-SCC	43be43cb-a367-4a74-94e4-973acafcf576	2	237388982	237394786	9	5804	4	2	3	NA	NA	NA

Cervix-SCC	43be43cb-a367-4a74-94e4-973acafcf576	3	178786361	178787764	6	1403	1	1	3	NA	1	NA
Cervix-SCC	43be43cb-a367-4a74-94e4-973acafcf576	6	113828619	113831815	6	3196	NA	5	1	NA	NA	NA
Cervix-SCC	43be43cb-a367-4a74-94e4-973acafcf576	6	129007539	129012765	9	5226	1	4	4	NA	NA	NA
Cervix-SCC	43be43cb-a367-4a74-94e4-973acafcf576	7	65625829	65629052	6	3223	1	2	3	NA	NA	NA
Cervix-SCC	43be43cb-a367-4a74-94e4-973acafcf576	10	75511672	75516110	6	4438	NA	2	4	NA	NA	NA
Cervix-SCC	43be43cb-a367-4a74-94e4-973acafcf576	10	78173099	78177790	8	4691	NA	1	7	NA	NA	NA
Cervix-SCC	43be43cb-a367-4a74-94e4-973acafcf576	12	65802010	65804546	6	2536	1	1	4	NA	NA	NA
Cervix-SCC	43be43cb-a367-4a74-94e4-973acafcf576	12	92296632	92299275	8	2643	NA	1	7	NA	NA	NA
Cervix-SCC	43be43cb-a367-4a74-94e4-973acafcf576	12	118572044	118576508	7	4464	NA	5	2	NA	NA	NA
Cervix-SCC	43be43cb-a367-4a74-94e4-973acafcf576	15	90903062	90907251	6	4189	NA	3	3	NA	NA	NA
Cervix-SCC	43be43cb-a367-4a74-94e4-973acafcf576	16	28918574	28922576	7	4002	NA	2	5	NA	NA	NA
Cervix-SCC	43be43cb-a367-4a74-94e4-973acafcf576	17	59326095	59335913	27	9818	7	9	11	NA	NA	NA
Cervix-SCC	43be43cb-a367-4a74-94e4-973acafcf576	17	74162851	74167300	7	4449	NA	2	5	NA	NA	NA
Cervix-SCC	43be43cb-a367-4a74-94e4-973acafcf576	21	40007996	40023076	23	15080	4	10	9	NA	NA	NA
Lymph-CLL	43d630aa-3890-401c-bf56-e3a9f325734f	3	186783612	186784093	6	481	NA	1	NA	4	NA	1
Lymph-CLL	43d630aa-3890-401c-bf56-e3a9f325734f	9	94476659	94476755	7	96	2	NA	NA	2	2	1
Lymph-CLL	43d630aa-3890-401c-bf56-e3a9f325734f	13	70939587	70943098	6	3511	NA	2	NA	NA	1	3
Lymph-CLL	43d630aa-3890-401c-bf56-e3a9f325734f	14	106239539	106241570	12	2031	1	4	6	1	NA	NA
Lymph-CLL	43d630aa-3890-401c-bf56-e3a9f325734f	14	106323653	106330831	84	7178	8	18	19	25	7	7
Lymph-CLL	43d630aa-3890-401c-bf56-e3a9f325734f	14	106829735	106830668	11	933	1	2	NA	6	1	1
Lymph-CLL	43d630aa-3890-401c-bf56-e3a9f325734f	22	22734419	22735525	10	1106	2	4	2	2	NA	NA
Lymph-CLL	43d630aa-3890-401c-bf56-e3a9f325734f	22	23164788	23165649	11	861	2	3	1	5	NA	NA
Lymph-CLL	43d630aa-3890-401c-bf56-e3a9f325734f	22	23247191	23247626	11	435	1	NA	5	5	NA	NA
Breast-AdenoCa	43f7a2e0-fec9-4e43-872d-18c6c946fa17	5	85284055	85284386	7	331	1	3	1	1	NA	1
Breast-AdenoCa	43f7a2e0-fec9-4e43-872d-18c6c946fa17	5	98112578	98113209	7	631	2	4	1	NA	NA	NA
Breast-AdenoCa	43f7a2e0-fec9-4e43-872d-18c6c946fa17	7	57948179	57949441	7	1262	NA	4	3	NA	NA	NA
Prost-AdenoCA	44083f54-0953-48e3-a704-11ad0988ad2e	2	146383828	146384462	7	634	1	3	3	NA	NA	NA
Panc-AdenoCA	441d2f21-b448-4fc8-8c54-9b85aca56237	14	60770474	60771242	6	768	NA	5	1	NA	NA	NA
Panc-AdenoCA	441d2f21-b448-4fc8-8c54-9b85aca56237	X	22366297	22366789	9	492	NA	NA	9	NA	NA	NA
Liver-HCC	446437de-c622-11e3-bf01-24c6515278c0	1	100138295	100140412	7	2117	NA	6	1	NA	NA	NA
Skin-Melanoma	450e8eba-a3a9-4dcb-b423-e33dfcd5c34e	1	239233675	239271107	43	37432	NA	2	40	NA	NA	1
Skin-Melanoma	450e8eba-a3a9-4dcb-b423-e33dfcd5c34e	1	240587275	240609828	147	22553	NA	NA	##	NA	NA	NA
Skin-Melanoma	450e8eba-a3a9-4dcb-b423-e33dfcd5c34e	2	181646435	181647356	10	921	1	8	NA	NA	1	NA
Skin-Melanoma	450e8eba-a3a9-4dcb-b423-e33dfcd5c34e	5	11509543	11511282	6	1739	1	2	3	NA	NA	NA
Skin-Melanoma	450e8eba-a3a9-4dcb-b423-e33dfcd5c34e	5	22232154	22236167	7	4013	NA	4	2	1	NA	NA
Skin-Melanoma	450e8eba-a3a9-4dcb-b423-e33dfcd5c34e	5	22398092	22399279	8	1187	NA	NA	8	NA	NA	NA
Skin-Melanoma	450e8eba-a3a9-4dcb-b423-e33dfcd5c34e	5	25842020	25843841	7	1821	2	4	1	NA	NA	NA
Skin-Melanoma	450e8eba-a3a9-4dcb-b423-e33dfcd5c34e	7	50288027	50288468	7	441	1	4	2	NA	NA	NA
Skin-Melanoma	450e8eba-a3a9-4dcb-b423-e33dfcd5c34e	7	50321640	50323128	7	1488	4	2	1	NA	NA	NA
Skin-Melanoma	450e8eba-a3a9-4dcb-b423-e33dfcd5c34e	16	67982152	67985787	7	3635	4	1	2	NA	NA	NA
Skin-Melanoma	450e8eba-a3a9-4dcb-b423-e33dfcd5c34e	16	89015410	89020339	7	4929	3	2	2	NA	NA	NA
Skin-Melanoma	450e8eba-a3a9-4dcb-b423-e33dfcd5c34e	16	89068035	89068449	9	414	2	2	5	NA	NA	NA
Skin-Melanoma	450e8eba-a3a9-4dcb-b423-e33dfcd5c34e	16	89132468	89133008	7	540	2	3	2	NA	NA	NA
Skin-Melanoma	450e8eba-a3a9-4dcb-b423-e33dfcd5c34e	21	45458435	45463422	17	4987	3	6	8	NA	NA	NA
Kidney-RCC	45348eff-4b09-4776-825e-b18de9dba937	9	130633690	130635520	6	1830	NA	2	4	NA	NA	NA
Bladder-TCC	45a7949d-e63f-4956-866c-df51257032de	1	149889351	149893977	6	4626	NA	2	4	NA	NA	NA
Bladder-TCC	45a7949d-e63f-4956-866c-df51257032de	2	32258660	32261913	8	3253	1	4	3	NA	NA	NA
Bladder-TCC	45a7949d-e63f-4956-866c-df51257032de	2	73307050	73309800	7	2750	1	4	2	NA	NA	NA
Bladder-TCC	45a7949d-e63f-4956-866c-df51257032de	5	59005310	59009592	6	4282	1	1	4	NA	NA	NA
Bladder-TCC	45a7949d-e63f-4956-866c-df51257032de	7	66053278	66058549	8	5271	NA	6	2	NA	NA	NA
Bladder-TCC	45a7949d-e63f-4956-866c-df51257032de	8	17315101	17318389	6	3288	NA	1	5	NA	NA	NA
Bladder-TCC	45a7949d-e63f-4956-866c-df51257032de	8	18610894	18617770	10	6876	NA	4	6	NA	NA	NA
Bladder-TCC	45a7949d-e63f-4956-866c-df51257032de	9	37826716	37828129	6	1413	1	3	2	NA	NA	NA
Bladder-TCC	45a7949d-e63f-4956-866c-df51257032de	10	62788187	62789045	8	858	NA	2	6	NA	NA	NA
Bladder-TCC	45a7949d-e63f-4956-866c-df51257032de	10	71311404	71313080	8	1676	3	2	3	NA	NA	NA
Bladder-TCC	45a7949d-e63f-4956-866c-df51257032de	13	101333481	101340509	22	7028	NA	12	9	1	NA	NA
Bladder-TCC	45a7949d-e63f-4956-866c-df51257032de	15	65530304	65533399	8	3095	2	3	3	NA	NA	NA
Bladder-TCC	45a7949d-e63f-4956-866c-df51257032de	15	79020597	79024593	7	3996	1	4	2	NA	NA	NA
Bladder-TCC	45a7949d-e63f-4956-866c-df51257032de	17	46523823	46528001	9	4178	NA	5	4	NA	NA	NA
Bladder-TCC	45a7949d-e63f-4956-866c-df51257032de	17	68250621	68255907	9	5286	4	2	3	NA	NA	NA

Bladder-TCC	45a7949d-e63f-4956-866c-df51257032de	18	366360	371227	8	4867	NA	2	6	NA	NA	NA
Bladder-TCC	45a7949d-e63f-4956-866c-df51257032de	18	33369134	33370024	7	890	1	2	4	NA	NA	NA
Bladder-TCC	45a7949d-e63f-4956-866c-df51257032de	19	1353274	1361265	12	7991	NA	2	10	NA	NA	NA
Bladder-TCC	45a7949d-e63f-4956-866c-df51257032de	20	6057762	6058362	6	600	1	3	2	NA	NA	NA
Bladder-TCC	45a7949d-e63f-4956-866c-df51257032de	Y	14174703	14180252	9	5549	NA	1	8	NA	NA	NA
Uterus-AdenoCA	460f7427-ebcf-404b-9e9e-e0278d0bca95	1	28076952	28090538	21	13586	5	8	8	NA	NA	NA
Uterus-AdenoCA	460f7427-ebcf-404b-9e9e-e0278d0bca95	3	85250261	85251216	9	955	2	3	4	NA	NA	NA
Uterus-AdenoCA	460f7427-ebcf-404b-9e9e-e0278d0bca95	4	137210548	137210678	8	130	NA	NA	NA	8	NA	NA
Uterus-AdenoCA	460f7427-ebcf-404b-9e9e-e0278d0bca95	6	51659480	51660092	7	612	NA	1	6	NA	NA	NA
Uterus-AdenoCA	460f7427-ebcf-404b-9e9e-e0278d0bca95	6	106037480	106043735	9	6255	1	3	5	NA	NA	NA
Uterus-AdenoCA	460f7427-ebcf-404b-9e9e-e0278d0bca95	6	108015372	108023578	18	8206	5	6	7	NA	NA	NA
Uterus-AdenoCA	460f7427-ebcf-404b-9e9e-e0278d0bca95	7	32981117	32983307	7	2190	4	NA	3	NA	NA	NA
Uterus-AdenoCA	460f7427-ebcf-404b-9e9e-e0278d0bca95	7	140910812	140916302	13	5490	4	8	1	NA	NA	NA
Uterus-AdenoCA	460f7427-ebcf-404b-9e9e-e0278d0bca95	8	43690877	43692435	9	1558	1	3	5	NA	NA	NA
Uterus-AdenoCA	460f7427-ebcf-404b-9e9e-e0278d0bca95	8	138629308	138629947	22	639	NA	3	19	NA	NA	NA
Uterus-AdenoCA	460f7427-ebcf-404b-9e9e-e0278d0bca95	12	2649662	2654450	11	4788	2	3	6	NA	NA	NA
Uterus-AdenoCA	460f7427-ebcf-404b-9e9e-e0278d0bca95	17	76456733	76457033	7	300	4	2	1	NA	NA	NA
Uterus-AdenoCA	460f7427-ebcf-404b-9e9e-e0278d0bca95	18	34086585	34091739	9	5154	2	5	2	NA	NA	NA
Uterus-AdenoCA	460f7427-ebcf-404b-9e9e-e0278d0bca95	X	63188280	63188745	6	465	NA	4	2	NA	NA	NA
Lymph-BNHL	461df2ae-fcf1-4b93-be0a-c14954fe7c42	2	187045637	187046165	6	528	NA	NA	NA	1	2	3
Lymph-BNHL	461df2ae-fcf1-4b93-be0a-c14954fe7c42	3	187461794	187474022	40	12228	3	8	5	11	4	9
Lymph-BNHL	461df2ae-fcf1-4b93-be0a-c14954fe7c42	4	179135906	179136723	6	817	NA	1	2	NA	2	1
Lymph-BNHL	461df2ae-fcf1-4b93-be0a-c14954fe7c42	12	113494186	113496353	8	2167	1	1	4	2	NA	NA
Lymph-BNHL	461df2ae-fcf1-4b93-be0a-c14954fe7c42	14	106110374	106115395	64	5021	3	10	41	6	1	3
Lymph-BNHL	461df2ae-fcf1-4b93-be0a-c14954fe7c42	14	106208151	106330036	318	1E+05	24	65	##	61	16	26
Lymph-BNHL	461df2ae-fcf1-4b93-be0a-c14954fe7c42	14	106829910	106830730	36	820	2	4	5	14	4	7
Lymph-BNHL	461df2ae-fcf1-4b93-be0a-c14954fe7c42	14	107169708	107179022	12	9314	NA	2	6	3	NA	1
Lymph-BNHL	461df2ae-fcf1-4b93-be0a-c14954fe7c42	22	22707524	22712226	9	4702	1	NA	1	4	NA	3
Lymph-BNHL	461df2ae-fcf1-4b93-be0a-c14954fe7c42	22	23100275	23101478	36	1203	1	6	12	5	8	4
Lymph-BNHL	461df2ae-fcf1-4b93-be0a-c14954fe7c42	22	23242159	23249358	18	7199	NA	NA	8	6	2	2
Panc-AdenoCA	4652ae03-b096-42d0-bdfa-3f6281d4f023	1	196630671	196633235	6	2564	NA	4	2	NA	NA	NA
Panc-AdenoCA	4652ae03-b096-42d0-bdfa-3f6281d4f023	3	13827285	13832415	15	5130	1	8	6	NA	NA	NA
Panc-AdenoCA	4652ae03-b096-42d0-bdfa-3f6281d4f023	8	35516860	35518011	6	1151	NA	2	4	NA	NA	NA
Panc-AdenoCA	4652ae03-b096-42d0-bdfa-3f6281d4f023	13	48474161	48474441	6	280	1	NA	5	NA	NA	NA
Panc-AdenoCA	4652ae03-b096-42d0-bdfa-3f6281d4f023	17	15136786	15138987	10	2201	NA	5	5	NA	NA	NA
Panc-AdenoCA	4652ae03-b096-42d0-bdfa-3f6281d4f023	17	15888658	15890633	12	1975	1	2	9	NA	NA	NA
Panc-AdenoCA	4652ae03-b096-42d0-bdfa-3f6281d4f023	20	44437071	44442520	23	5449	3	7	13	NA	NA	NA
Breast-AdenoCa	467ef778-f481-44f3-9768-b9900252d2ba	10	44601139	44601792	6	653	1	4	NA	NA	1	NA
Head-SCC	46d35b82-e1b7-4d35-be5e-3a70fd47e421	1	66022133	66023219	6	1086	1	2	3	NA	NA	NA
Head-SCC	46d35b82-e1b7-4d35-be5e-3a70fd47e421	2	86889632	86891329	6	1697	NA	1	5	NA	NA	NA
Head-SCC	46d35b82-e1b7-4d35-be5e-3a70fd47e421	3	114358992	114360503	8	1511	NA	2	6	NA	NA	NA
Head-SCC	46d35b82-e1b7-4d35-be5e-3a70fd47e421	5	33534815	33536504	10	1689	NA	6	4	NA	NA	NA
Head-SCC	46d35b82-e1b7-4d35-be5e-3a70fd47e421	6	149971998	149977619	11	5621	NA	4	7	NA	NA	NA
Head-SCC	46d35b82-e1b7-4d35-be5e-3a70fd47e421	7	111752063	111755752	7	3689	NA	3	4	NA	NA	NA
Head-SCC	46d35b82-e1b7-4d35-be5e-3a70fd47e421	7	151895227	151897794	7	2567	NA	4	3	NA	NA	NA
Head-SCC	46d35b82-e1b7-4d35-be5e-3a70fd47e421	7	153388528	153392566	8	4038	NA	3	5	NA	NA	NA
Head-SCC	46d35b82-e1b7-4d35-be5e-3a70fd47e421	8	23605251	23607418	6	2167	1	1	4	NA	NA	NA
Head-SCC	46d35b82-e1b7-4d35-be5e-3a70fd47e421	9	127064104	127066798	7	2694	NA	3	4	NA	NA	NA
Head-SCC	46d35b82-e1b7-4d35-be5e-3a70fd47e421	12	4500699	4505109	6	4410	NA	1	5	NA	NA	NA
Head-SCC	46d35b82-e1b7-4d35-be5e-3a70fd47e421	15	60260846	60261639	7	793	NA	3	4	NA	NA	NA
Head-SCC	46d35b82-e1b7-4d35-be5e-3a70fd47e421	17	66013703	66016009	7	2306	1	3	3	NA	NA	NA
Head-SCC	46d35b82-e1b7-4d35-be5e-3a70fd47e421	19	20702885	20703104	6	219	1	2	3	NA	NA	NA
Head-SCC	46d35b82-e1b7-4d35-be5e-3a70fd47e421	22	31776706	31779439	9	2733	NA	5	4	NA	NA	NA
Head-SCC	46d35b82-e1b7-4d35-be5e-3a70fd47e421	X	100412349	100414647	6	2298	NA	1	5	NA	NA	NA
Panc-AdenoCA	46e166fe-3b20-49ad-98db-42c854c61c93	1	161948367	161950058	6	1691	NA	3	3	NA	NA	NA
Panc-AdenoCA	46e166fe-3b20-49ad-98db-42c854c61c93	3	136389701	136390675	6	974	NA	1	5	NA	NA	NA
Panc-AdenoCA	46e166fe-3b20-49ad-98db-42c854c61c93	10	125214896	125216137	9	1241	NA	NA	9	NA	NA	NA
Ovary-AdenoCA	46f19b5c-3eba-4b23-a1ab-9748090ca4e5	1	203270780	203276706	8	5926	2	2	3	NA	NA	1
Ovary-AdenoCA	46f19b5c-3eba-4b23-a1ab-9748090ca4e5	12	10052173	10058575	10	6402	NA	6	4	NA	NA	NA
Ovary-AdenoCA	46f19b5c-3eba-4b23-a1ab-9748090ca4e5	15	35394205	35394346	11	141	NA	2	3	4	NA	2

Breast-AdenoCa	47312f61-5ef4-4f25-9320-8fbb4758790e	1	207024300	207027962	12	3662	1	9	2	NA	NA	NA
Breast-AdenoCa	47312f61-5ef4-4f25-9320-8fbb4758790e	1	208206125	208211147	8	5022	1	1	5	NA	NA	1
Breast-AdenoCa	47312f61-5ef4-4f25-9320-8fbb4758790e	1	209503430	209509374	14	5944	2	5	7	NA	NA	NA
Breast-AdenoCa	47312f61-5ef4-4f25-9320-8fbb4758790e	1	210258458	210261378	9	2920	1	3	5	NA	NA	NA
Breast-AdenoCa	47312f61-5ef4-4f25-9320-8fbb4758790e	1	210364684	210374448	17	9764	1	6	10	NA	NA	NA
Breast-AdenoCa	47312f61-5ef4-4f25-9320-8fbb4758790e	1	210475828	210483631	11	7803	1	5	4	NA	NA	1
Breast-AdenoCa	47312f61-5ef4-4f25-9320-8fbb4758790e	1	210895645	210898580	13	2935	4	4	5	NA	NA	NA
Breast-AdenoCa	47312f61-5ef4-4f25-9320-8fbb4758790e	1	211106896	211107515	11	619	NA	1	10	NA	NA	NA
Breast-AdenoCa	47312f61-5ef4-4f25-9320-8fbb4758790e	1	213448768	213457508	10	8740	2	6	2	NA	NA	NA
Breast-AdenoCa	47312f61-5ef4-4f25-9320-8fbb4758790e	1	215629490	215636416	15	6926	1	9	5	NA	NA	NA
Breast-AdenoCa	47312f61-5ef4-4f25-9320-8fbb4758790e	1	216329305	216330306	7	1001	NA	2	5	NA	NA	NA
Breast-AdenoCa	47312f61-5ef4-4f25-9320-8fbb4758790e	1	218043401	218050532	15	7131	2	3	10	NA	NA	NA
Breast-AdenoCa	47312f61-5ef4-4f25-9320-8fbb4758790e	1	219518926	219521414	10	2488	1	2	5	1	1	NA
Breast-AdenoCa	47312f61-5ef4-4f25-9320-8fbb4758790e	1	221599385	221603658	7	4273	1	4	2	NA	NA	NA
Breast-AdenoCa	47312f61-5ef4-4f25-9320-8fbb4758790e	1	223420375	223421643	7	1268	1	2	4	NA	NA	NA
Breast-AdenoCa	47312f61-5ef4-4f25-9320-8fbb4758790e	1	225117821	225122006	8	4185	NA	5	3	NA	NA	NA
Breast-AdenoCa	47312f61-5ef4-4f25-9320-8fbb4758790e	12	27633247	27633381	6	134	1	2	3	NA	NA	NA
Breast-AdenoCa	47312f61-5ef4-4f25-9320-8fbb4758790e	14	60406706	60410766	11	4060	2	1	8	NA	NA	NA
Breast-AdenoCa	47312f61-5ef4-4f25-9320-8fbb4758790e	14	62699888	62702647	11	2759	1	3	7	NA	NA	NA
Breast-AdenoCa	47312f61-5ef4-4f25-9320-8fbb4758790e	14	66197184	66202267	12	5083	NA	4	8	NA	NA	NA
Breast-AdenoCa	47312f61-5ef4-4f25-9320-8fbb4758790e	14	66337946	66341475	12	3529	3	4	5	NA	NA	NA
Breast-AdenoCa	47312f61-5ef4-4f25-9320-8fbb4758790e	22	33889076	33891216	7	2140	1	1	5	NA	NA	NA
Eso-AdenoCa	47874ff4-0b1c-4a31-9192-2a10e93ce9a1	2	156531127	156531617	13	490	NA	NA	NA	13	NA	NA
Eso-AdenoCa	47874ff4-0b1c-4a31-9192-2a10e93ce9a1	3	55542938	55547471	8	4533	1	5	2	NA	NA	NA
Eso-AdenoCa	47874ff4-0b1c-4a31-9192-2a10e93ce9a1	3	194230871	194232949	9	2078	1	NA	8	NA	NA	NA
Eso-AdenoCa	47874ff4-0b1c-4a31-9192-2a10e93ce9a1	5	34077855	34079869	6	2014	1	1	4	NA	NA	NA
Eso-AdenoCa	47874ff4-0b1c-4a31-9192-2a10e93ce9a1	6	10525624	10529304	18	3680	2	2	14	NA	NA	NA
Eso-AdenoCa	47874ff4-0b1c-4a31-9192-2a10e93ce9a1	7	129264340	129265572	10	1232	2	6	2	NA	NA	NA
Eso-AdenoCa	47874ff4-0b1c-4a31-9192-2a10e93ce9a1	11	90453426	90453698	7	272	NA	4	3	NA	NA	NA
Eso-AdenoCa	47874ff4-0b1c-4a31-9192-2a10e93ce9a1	12	64842759	64844167	9	1408	NA	NA	NA	9	NA	NA
Eso-AdenoCa	47874ff4-0b1c-4a31-9192-2a10e93ce9a1	12	125794025	125796713	9	2688	3	4	2	NA	NA	NA
Eso-AdenoCa	47874ff4-0b1c-4a31-9192-2a10e93ce9a1	18	38538574	38539179	6	605	3	1	2	NA	NA	NA
Eso-AdenoCa	47874ff4-0b1c-4a31-9192-2a10e93ce9a1	19	39355228	39359714	9	4486	NA	NA	1	8	NA	NA
Eso-AdenoCa	47874ff4-0b1c-4a31-9192-2a10e93ce9a1	19	45218211	45218965	11	754	4	1	5	NA	NA	1
Eso-AdenoCa	47874ff4-0b1c-4a31-9192-2a10e93ce9a1	19	47987643	47988378	9	735	3	NA	5	NA	NA	1
Eso-AdenoCa	47874ff4-0b1c-4a31-9192-2a10e93ce9a1	19	55848969	55850953	14	1984	2	1	11	NA	NA	NA
Eso-AdenoCa	47874ff4-0b1c-4a31-9192-2a10e93ce9a1	22	37173107	37176002	10	2895	NA	8	2	NA	NA	NA
Eso-AdenoCa	47874ff4-0b1c-4a31-9192-2a10e93ce9a1	X	98854274	98858623	6	4349	NA	4	2	NA	NA	NA
Liver-HCC	47e5ff56-9534-4fcf-a593-33027e877f93	X	67033624	67035995	10	2371	NA	6	4	NA	NA	NA
Bladder-TCC	4838b5a9-968c-4178-bffb-3fafe1f6dc09	6	14657080	14657992	6	912	1	3	2	NA	NA	NA
Bladder-TCC	4838b5a9-968c-4178-bffb-3fafe1f6dc09	8	9555298	9555931	8	633	2	4	2	NA	NA	NA
Bladder-TCC	4838b5a9-968c-4178-bffb-3fafe1f6dc09	11	58608468	58613705	7	5237	1	4	1	NA	NA	1
Bladder-TCC	4838b5a9-968c-4178-bffb-3fafe1f6dc09	12	92130722	92134158	19	3436	2	11	6	NA	NA	NA
Bladder-TCC	4838b5a9-968c-4178-bffb-3fafe1f6dc09	17	41472215	41476562	10	4347	NA	4	6	NA	NA	NA
Bladder-TCC	4838b5a9-968c-4178-bffb-3fafe1f6dc09	19	48692297	48695510	17	3213	2	5	10	NA	NA	NA
Lung-SCC	48f68f73-3ac0-44ac-ba12-b8889fcb7f38	1	76338972	76342730	22	3758	2	7	13	NA	NA	NA
Uterus-AdenoCA	493e7008-551c-4c0e-b567-a0f31868629e	6	57575541	57577745	8	2204	2	5	1	NA	NA	NA
Uterus-AdenoCA	493e7008-551c-4c0e-b567-a0f31868629e	7	69131232	69131537	6	305	1	4	1	NA	NA	NA
Uterus-AdenoCA	493e7008-551c-4c0e-b567-a0f31868629e	11	58427294	58428052	9	758	3	1	5	NA	NA	NA
Uterus-AdenoCA	493e7008-551c-4c0e-b567-a0f31868629e	18	60775123	60775477	8	354	1	5	2	NA	NA	NA
Uterus-AdenoCA	493e7008-551c-4c0e-b567-a0f31868629e	19	10984629	10985178	9	549	3	4	2	NA	NA	NA
Uterus-AdenoCA	493e7008-551c-4c0e-b567-a0f31868629e	19	32132317	32134746	10	2429	5	5	NA	NA	NA	NA
Liver-HCC	49795e42-c623-11e3-bf01-24c6515278c0	8	76610133	76610631	9	498	NA	NA	NA	3	5	1
Liver-HCC	49795e42-c623-11e3-bf01-24c6515278c0	13	69552010	69553002	6	992	1	NA	NA	3	2	NA
Liver-HCC	49795e42-c623-11e3-bf01-24c6515278c0	14	83097689	83100066	7	2377	1	NA	2	2	1	1
CNS-Medullo	49fa7e44-c86f-4b1f-8849-e5fe5b9de734	5	105723921	105724812	7	891	NA	NA	NA	4	1	2
CNS-Medullo	49fa7e44-c86f-4b1f-8849-e5fe5b9de734	X	30036749	30041485	7	4736	NA	NA	1	6	NA	NA
Lymph-BNHL	4a4309b0-2aca-4d6a-ac50-039b28406bba	2	89158082	89160064	38	1982	1	3	7	15	4	8
Lymph-BNHL	4a4309b0-2aca-4d6a-ac50-039b28406bba	3	187462535	187463008	6	473	NA	1	4	1	NA	NA
Lymph-BNHL	4a4309b0-2aca-4d6a-ac50-039b28406bba	14	106210965	106213873	14	2908	1	4	9	NA	NA	NA

Lymph-BNHL	4a4309b0-2aca-4d6a-ac50-039b28406bba	14	106323403	106330170	102	6767	5	16	43	19	8	11
Lymph-BNHL	4a4309b0-2aca-4d6a-ac50-039b28406bba	14	106994233	106994549	12	316	NA	NA	4	7	1	NA
Panc-AdenoCA	4a88981e-e409-440f-9bf0-71ca1bab8b4e	6	68000688	68001507	12	819	NA	NA	12	NA	NA	NA
Panc-AdenoCA	4aa7eabc-0419-4897-b7af-5dcccfd6ac2f	8	129067308	129068394	10	1086	3	2	5	NA	NA	NA
Panc-AdenoCA	4aa7eabc-0419-4897-b7af-5dcccfd6ac2f	8	130898430	130899062	7	632	NA	1	5	NA	NA	1
Panc-AdenoCA	4aa7eabc-0419-4897-b7af-5dcccfd6ac2f	12	115627780	115631204	10	3424	2	2	6	NA	NA	NA
Panc-AdenoCA	4aa7eabc-0419-4897-b7af-5dcccfd6ac2f	12	116552460	116555111	9	2651	3	2	4	NA	NA	NA
Panc-AdenoCA	4aa7eabc-0419-4897-b7af-5dcccfd6ac2f	18	1026359	1026712	6	353	NA	3	3	NA	NA	NA
Lymph-BNHL	4adc846a-42cb-4ebd-a954-e31eb29d7572	1	24699714	24700144	6	430	NA	NA	1	2	NA	3
Lymph-BNHL	4adc846a-42cb-4ebd-a954-e31eb29d7572	2	51356188	51357785	6	1597	NA	NA	NA	2	2	2
Lymph-BNHL	4adc846a-42cb-4ebd-a954-e31eb29d7572	2	69017332	69017554	7	222	NA	1	NA	2	4	NA
Lymph-BNHL	4adc846a-42cb-4ebd-a954-e31eb29d7572	2	82545334	82548976	7	3642	1	1	NA	4	NA	1
Lymph-BNHL	4adc846a-42cb-4ebd-a954-e31eb29d7572	2	89140551	89146089	7	5538	1	NA	1	2	3	NA
Lymph-BNHL	4adc846a-42cb-4ebd-a954-e31eb29d7572	2	89155330	89196264	115	40934	6	16	11	36	20	26
Lymph-BNHL	4adc846a-42cb-4ebd-a954-e31eb29d7572	2	171329939	171330104	10	165	NA	NA	NA	5	4	1
Lymph-BNHL	4adc846a-42cb-4ebd-a954-e31eb29d7572	2	212350143	212359943	13	9800	2	NA	2	3	3	3
Lymph-BNHL	4adc846a-42cb-4ebd-a954-e31eb29d7572	3	16551943	16556008	9	4065	1	NA	NA	6	1	1
Lymph-BNHL	4adc846a-42cb-4ebd-a954-e31eb29d7572	3	163615728	163625306	15	9578	NA	1	2	6	1	5
Lymph-BNHL	4adc846a-42cb-4ebd-a954-e31eb29d7572	3	183272476	183280194	9	7718	NA	NA	1	5	2	1
Lymph-BNHL	4adc846a-42cb-4ebd-a954-e31eb29d7572	3	187461900	187463023	8	1123	NA	3	3	2	NA	NA
Lymph-BNHL	4adc846a-42cb-4ebd-a954-e31eb29d7572	4	45749428	45760109	13	10681	3	NA	1	4	1	4
Lymph-BNHL	4adc846a-42cb-4ebd-a954-e31eb29d7572	4	131248679	131254792	10	6113	2	NA	2	4	NA	2
Lymph-BNHL	4adc846a-42cb-4ebd-a954-e31eb29d7572	4	182117667	182122253	7	4586	1	NA	NA	2	2	2
Lymph-BNHL	4adc846a-42cb-4ebd-a954-e31eb29d7572	4	182630388	182635746	8	5358	NA	NA	NA	4	3	1
Lymph-BNHL	4adc846a-42cb-4ebd-a954-e31eb29d7572	5	117061226	117065582	6	4356	NA	NA	1	2	2	1
Lymph-BNHL	4adc846a-42cb-4ebd-a954-e31eb29d7572	5	165042319	165048724	8	6405	1	NA	2	3	1	1
Lymph-BNHL	4adc846a-42cb-4ebd-a954-e31eb29d7572	6	45918604	45921608	6	3004	1	NA	1	NA	1	3
Lymph-BNHL	4adc846a-42cb-4ebd-a954-e31eb29d7572	6	45935301	45941013	7	5712	NA	2	1	3	NA	1
Lymph-BNHL	4adc846a-42cb-4ebd-a954-e31eb29d7572	6	67127761	67136435	11	8674	1	1	1	6	1	1
Lymph-BNHL	4adc846a-42cb-4ebd-a954-e31eb29d7572	6	84723597	84724785	9	1188	NA	NA	1	4	1	3
Lymph-BNHL	4adc846a-42cb-4ebd-a954-e31eb29d7572	6	120879101	120882173	9	3072	1	2	1	1	1	3
Lymph-BNHL	4adc846a-42cb-4ebd-a954-e31eb29d7572	6	134489432	134497081	22	7649	2	4	13	2	1	NA
Lymph-BNHL	4adc846a-42cb-4ebd-a954-e31eb29d7572	8	1725610	1725694	9	84	NA	NA	NA	5	1	3
Lymph-BNHL	4adc846a-42cb-4ebd-a954-e31eb29d7572	8	8598956	8602936	8	3980	1	2	NA	2	1	2
Lymph-BNHL	4adc846a-42cb-4ebd-a954-e31eb29d7572	8	112724276	112732024	10	7748	1	NA	3	NA	1	5
Lymph-BNHL	4adc846a-42cb-4ebd-a954-e31eb29d7572	9	23235768	23237145	7	1377	1	NA	NA	2	1	3
Lymph-BNHL	4adc846a-42cb-4ebd-a954-e31eb29d7572	10	58302458	58302905	6	447	NA	1	NA	NA	1	4
Lymph-BNHL	4adc846a-42cb-4ebd-a954-e31eb29d7572	11	22295210	22302122	17	6912	1	1	1	5	3	6
Lymph-BNHL	4adc846a-42cb-4ebd-a954-e31eb29d7572	12	90041778	90041891	7	113	NA	NA	NA	3	1	3
Lymph-BNHL	4adc846a-42cb-4ebd-a954-e31eb29d7572	13	64632911	64642059	12	9148	NA	NA	1	3	4	4
Lymph-BNHL	4adc846a-42cb-4ebd-a954-e31eb29d7572	13	69749723	69753577	7	3854	NA	NA	1	3	2	1
Lymph-BNHL	4adc846a-42cb-4ebd-a954-e31eb29d7572	14	96179985	96180327	6	342	NA	NA	1	1	1	3
Lymph-BNHL	4adc846a-42cb-4ebd-a954-e31eb29d7572	14	106237301	106242978	7	5677	NA	2	3	1	NA	1
Lymph-BNHL	4adc846a-42cb-4ebd-a954-e31eb29d7572	14	106326832	106376573	77	49741	8	8	24	20	9	8
Lymph-BNHL	4adc846a-42cb-4ebd-a954-e31eb29d7572	14	107219060	107259302	42	40242	2	9	4	8	15	4
Lymph-BNHL	4adc846a-42cb-4ebd-a954-e31eb29d7572	15	65995413	65995646	8	233	NA	NA	NA	4	NA	4
Lymph-BNHL	4adc846a-42cb-4ebd-a954-e31eb29d7572	17	57915821	57917269	6	1448	NA	1	3	2	NA	NA
Lymph-BNHL	4adc846a-42cb-4ebd-a954-e31eb29d7572	18	1819264	1829590	12	10326	NA	1	1	1	3	6
Lymph-BNHL	4adc846a-42cb-4ebd-a954-e31eb29d7572	22	22785972	22786624	19	652	3	5	3	6	2	NA
Lymph-BNHL	4adc846a-42cb-4ebd-a954-e31eb29d7572	22	23199016	23199562	7	546	NA	NA	NA	2	3	2
Lymph-BNHL	4adc846a-42cb-4ebd-a954-e31eb29d7572	22	23207713	23210198	8	2485	NA	NA	NA	6	1	1
Lymph-BNHL	4adc846a-42cb-4ebd-a954-e31eb29d7572	22	23223168	23246529	42	23361	NA	8	11	17	1	5
Lymph-BNHL	4adc846a-42cb-4ebd-a954-e31eb29d7572	X	12993451	12994235	6	784	1	3	1	NA	NA	1
Lymph-BNHL	4adc846a-42cb-4ebd-a954-e31eb29d7572	X	26451821	26455790	8	3969	1	NA	NA	1	3	3
Lymph-BNHL	4adc846a-42cb-4ebd-a954-e31eb29d7572	X	93224630	93232178	9	7548	NA	NA	NA	3	2	4
Panc-Endocrine	4b5d9d8c-ff95-45f4-9287-2804e8a98ade	X	88458905	88460722	6	1817	1	1	3	NA	1	NA
Liver-HCC	4b8943be-c623-11e3-bf01-24c6515278c0	6	64969074	64971178	9	2104	1	4	3	1	NA	NA
Liver-HCC	4b8943be-c623-11e3-bf01-24c6515278c0	6	73171997	73174458	10	2461	2	4	4	NA	NA	NA
Liver-HCC	4b8943be-c623-11e3-bf01-24c6515278c0	7	83509255	83512108	11	2853	1	5	5	NA	NA	NA
Panc-AdenoCA	4c027164-12de-456f-b933-6bc91f70b46f	7	63707234	63707285	7	51	NA	1	NA	5	1	NA

Panc-AdenoCA	4c027164-12de-456f-b933-6bc91f70b46f	12	4636562	4636834	8	272	2	5	1	NA	NA	NA
Panc-AdenoCA	4c027164-12de-456f-b933-6bc91f70b46f	12	5097035	5097754	18	719	1	4	13	NA	NA	NA
Panc-AdenoCA	4c027164-12de-456f-b933-6bc91f70b46f	12	25832195	25837013	8	4818	NA	3	5	NA	NA	NA
Panc-AdenoCA	4c027164-12de-456f-b933-6bc91f70b46f	12	25929689	25930292	9	603	NA	3	6	NA	NA	NA
Panc-AdenoCA	4c027164-12de-456f-b933-6bc91f70b46f	12	30454498	30455599	9	1101	NA	3	6	NA	NA	NA
Panc-AdenoCA	4c027164-12de-456f-b933-6bc91f70b46f	12	40534195	40534434	8	239	3	2	3	NA	NA	NA
Panc-AdenoCA	4c027164-12de-456f-b933-6bc91f70b46f	12	61099550	61099908	9	358	NA	4	4	1	NA	NA
Panc-AdenoCA	4c027164-12de-456f-b933-6bc91f70b46f	12	74036778	74037284	9	506	3	2	4	NA	NA	NA
Panc-AdenoCA	4c027164-12de-456f-b933-6bc91f70b46f	17	25365973	25368365	10	2392	2	1	7	NA	NA	NA
Panc-AdenoCA	4c027164-12de-456f-b933-6bc91f70b46f	17	53778136	53781952	11	3816	1	5	5	NA	NA	NA
Panc-AdenoCA	4c027164-12de-456f-b933-6bc91f70b46f	17	61548363	61557970	21	9607	2	4	15	NA	NA	NA
Panc-AdenoCA	4c4aa1b1-fda3-4c5b-b588-68aa727500ad	9	34590673	34591394	6	721	2	1	3	NA	NA	NA
Panc-AdenoCA	4c4aa1b1-fda3-4c5b-b588-68aa727500ad	13	74304030	74305019	10	989	NA	3	7	NA	NA	NA
Panc-AdenoCA	4c4aa1b1-fda3-4c5b-b588-68aa727500ad	13	115103418	115105807	22	2389	2	8	12	NA	NA	NA
Panc-AdenoCA	4c4aa1b1-fda3-4c5b-b588-68aa727500ad	18	23752350	23752834	6	484	NA	NA	6	NA	NA	NA
Prost-AdenoCA	4c5228b5-bf31-4abd-a47c-d088e16dba13	3	60759903	60763804	6	3901	NA	5	1	NA	NA	NA
Bone-Leiomyo	4c59fb2d-21b6-4b09-8174-6102de736e4d	1	11213694	11218366	11	4672	4	3	4	NA	NA	NA
Bone-Leiomyo	4c59fb2d-21b6-4b09-8174-6102de736e4d	1	56129453	56130363	9	910	NA	NA	NA	9	NA	NA
Bone-Leiomyo	4c59fb2d-21b6-4b09-8174-6102de736e4d	1	158624312	158635241	19	10929	2	8	9	NA	NA	NA
Bone-Leiomyo	4c59fb2d-21b6-4b09-8174-6102de736e4d	1	160549762	160551276	10	1514	2	4	4	NA	NA	NA
Bone-Leiomyo	4c59fb2d-21b6-4b09-8174-6102de736e4d	1	166539910	166551791	13	11881	2	7	4	NA	NA	NA
Bone-Leiomyo	4c59fb2d-21b6-4b09-8174-6102de736e4d	1	167094167	167099291	7	5124	2	4	1	NA	NA	NA
Bone-Leiomyo	4c59fb2d-21b6-4b09-8174-6102de736e4d	1	169760330	169760597	8	267	1	4	3	NA	NA	NA
Bone-Leiomyo	4c59fb2d-21b6-4b09-8174-6102de736e4d	1	170419621	170423222	15	3601	3	10	2	NA	NA	NA
Bone-Leiomyo	4c59fb2d-21b6-4b09-8174-6102de736e4d	3	76924	80808	10	3884	1	6	3	NA	NA	NA
Bone-Leiomyo	4c59fb2d-21b6-4b09-8174-6102de736e4d	3	28847130	28850139	12	3009	3	5	4	NA	NA	NA
Bone-Leiomyo	4c59fb2d-21b6-4b09-8174-6102de736e4d	5	319893	321352	6	1459	2	3	1	NA	NA	NA
Bone-Leiomyo	4c59fb2d-21b6-4b09-8174-6102de736e4d	5	1496841	1498821	7	1980	1	5	NA	NA	NA	1
Bone-Leiomyo	4c59fb2d-21b6-4b09-8174-6102de736e4d	5	4472912	4477014	8	4102	3	4	1	NA	NA	NA
Bone-Leiomyo	4c59fb2d-21b6-4b09-8174-6102de736e4d	5	4687243	4688486	7	1243	NA	4	3	NA	NA	NA
Bone-Leiomyo	4c59fb2d-21b6-4b09-8174-6102de736e4d	5	22575775	22577777	9	2002	3	2	4	NA	NA	NA
Bone-Leiomyo	4c59fb2d-21b6-4b09-8174-6102de736e4d	5	25740340	25742644	9	2304	1	3	5	NA	NA	NA
Bone-Leiomyo	4c59fb2d-21b6-4b09-8174-6102de736e4d	5	39110268	39111369	8	1101	1	5	2	NA	NA	NA
Bone-Leiomyo	4c59fb2d-21b6-4b09-8174-6102de736e4d	7	122447349	122457300	11	9951	2	5	4	NA	NA	NA
Bone-Leiomyo	4c59fb2d-21b6-4b09-8174-6102de736e4d	7	126472804	126502363	31	29559	3	16	10	1	1	NA
Bone-Leiomyo	4c59fb2d-21b6-4b09-8174-6102de736e4d	7	126707872	126712024	8	4152	3	3	2	NA	NA	NA
Bone-Leiomyo	4c59fb2d-21b6-4b09-8174-6102de736e4d	7	130335065	130337752	7	2687	NA	4	3	NA	NA	NA
Bone-Leiomyo	4c59fb2d-21b6-4b09-8174-6102de736e4d	7	130810133	130812737	10	2604	1	6	3	NA	NA	NA
Bone-Leiomyo	4c59fb2d-21b6-4b09-8174-6102de736e4d	8	24880554	24881840	11	1286	2	2	7	NA	NA	NA
Bone-Leiomyo	4c59fb2d-21b6-4b09-8174-6102de736e4d	9	27631906	27657697	53	25791	NA	NA	52	1	NA	NA
Bone-Leiomyo	4c59fb2d-21b6-4b09-8174-6102de736e4d	12	60999775	61009630	12	9855	2	8	1	NA	1	NA
Bone-Leiomyo	4c59fb2d-21b6-4b09-8174-6102de736e4d	12	61510094	61516053	18	5959	3	8	5	NA	NA	2
Bone-Leiomyo	4c59fb2d-21b6-4b09-8174-6102de736e4d	12	61572748	61575219	7	2471	NA	5	2	NA	NA	NA
Bone-Leiomyo	4c59fb2d-21b6-4b09-8174-6102de736e4d	12	61592826	61596299	9	3473	1	3	5	NA	NA	NA
Bone-Leiomyo	4c59fb2d-21b6-4b09-8174-6102de736e4d	12	62674043	62678116	29	4073	2	20	6	1	NA	NA
Bone-Leiomyo	4c59fb2d-21b6-4b09-8174-6102de736e4d	12	65468286	65474708	11	6422	4	6	1	NA	NA	NA
Bone-Leiomyo	4c59fb2d-21b6-4b09-8174-6102de736e4d	12	65808746	65812444	7	3698	NA	5	1	NA	NA	1
Bone-Leiomyo	4c59fb2d-21b6-4b09-8174-6102de736e4d	12	70984833	70988329	7	3496	1	4	2	NA	NA	NA
Bone-Leiomyo	4c59fb2d-21b6-4b09-8174-6102de736e4d	12	104930540	104940032	11	9492	3	6	1	NA	NA	1
Bone-Leiomyo	4c59fb2d-21b6-4b09-8174-6102de736e4d	13	53934191	53938322	11	4131	2	6	3	NA	NA	NA
Bone-Leiomyo	4c59fb2d-21b6-4b09-8174-6102de736e4d	13	113802308	113804881	10	2573	NA	NA	10	NA	NA	NA
Bone-Leiomyo	4c59fb2d-21b6-4b09-8174-6102de736e4d	14	48677483	48678722	9	1239	1	4	4	NA	NA	NA
Bone-Leiomyo	4c59fb2d-21b6-4b09-8174-6102de736e4d	14	69959463	69964450	14	4987	2	4	8	NA	NA	NA
Bone-Leiomyo	4c59fb2d-21b6-4b09-8174-6102de736e4d	14	73237526	73238893	7	1367	1	4	2	NA	NA	NA
Bone-Leiomyo	4c59fb2d-21b6-4b09-8174-6102de736e4d	17	27256167	27264652	11	8485	3	7	1	NA	NA	NA
Bone-Leiomyo	4c59fb2d-21b6-4b09-8174-6102de736e4d	17	61253689	61255353	9	1664	2	5	2	NA	NA	NA
Bone-Leiomyo	4c59fb2d-21b6-4b09-8174-6102de736e4d	22	34977998	34982773	13	4775	2	8	3	NA	NA	NA
Bone-Leiomyo	4c59fb2d-21b6-4b09-8174-6102de736e4d	22	48579152	48584987	10	5835	NA	6	4	NA	NA	NA
Bone-Leiomyo	4c59fb2d-21b6-4b09-8174-6102de736e4d	X	39137274	39140141	7	2867	1	4	2	NA	NA	NA
Liver-HCC	4c8afa82-c623-11e3-bf01-24c6515278c0	4	47625351	47626167	8	816	1	4	3	NA	NA	NA

Liver-HCC	4c8afa82-c623-11e3-bf01-24c6515278c0	6	151016848	151022144	11	5296	NA	11	NA	NA	NA	NA
Panc-AdenoCA	4cbe411b-b05e-46bd-bea8-126289a0866c	2	200520067	200521886	6	1819	NA	2	4	NA	NA	NA
Panc-AdenoCA	4cbe411b-b05e-46bd-bea8-126289a0866c	17	15315625	15325564	15	9939	3	6	6	NA	NA	NA
Panc-AdenoCA	4cbe411b-b05e-46bd-bea8-126289a0866c	17	16455883	16456837	8	954	NA	6	2	NA	NA	NA
Panc-AdenoCA	4cbe411b-b05e-46bd-bea8-126289a0866c	18	30651910	30653807	7	1897	2	3	2	NA	NA	NA
ColoRect-AdenoCA	4cfb0aee-6661-478a-8694-9d8ba15e46b7	10	90128594	90130955	16	2361	2	4	9	NA	NA	1
Liver-HCC	4cff8590-559e-4204-8635-96e11bfeda68	5	22229486	22231814	6	2328	1	NA	5	NA	NA	NA
Liver-HCC	4cff8590-559e-4204-8635-96e11bfeda68	5	53795385	53800837	15	5452	1	6	8	NA	NA	NA
Liver-HCC	4cff8590-559e-4204-8635-96e11bfeda68	5	151523030	151527348	10	4318	NA	3	6	NA	NA	1
Prost-AdenoCA	4d11d7da-1204-437e-87b1-e8337a67c9a8	8	42776204	42780177	14	3973	1	8	5	NA	NA	NA
Breast-AdenoCa	4d409156-3a36-4c63-9ad6-b6af34f27b06	1	156012093	156013281	9	1188	NA	4	5	NA	NA	NA
Breast-AdenoCa	4d409156-3a36-4c63-9ad6-b6af34f27b06	1	178974917	178975779	11	862	NA	4	7	NA	NA	NA
Breast-AdenoCa	4d409156-3a36-4c63-9ad6-b6af34f27b06	2	11149839	11151909	10	2070	NA	2	8	NA	NA	NA
Breast-AdenoCa	4d409156-3a36-4c63-9ad6-b6af34f27b06	17	37135421	37136828	12	1407	NA	6	6	NA	NA	NA
Breast-AdenoCa	4d409156-3a36-4c63-9ad6-b6af34f27b06	17	57526420	57527045	11	625	NA	5	6	NA	NA	NA
Breast-AdenoCa	4d409156-3a36-4c63-9ad6-b6af34f27b06	17	68231853	68241345	24	9492	8	6	9	NA	1	NA
Bone-Leiomyo	4d4eff82-ede0-47f1-b49a-ada025952114	1	41551039	41553534	16	2495	1	9	6	NA	NA	NA
Bone-Leiomyo	4d4eff82-ede0-47f1-b49a-ada025952114	5	26888554	26897034	24	8480	1	16	7	NA	NA	NA
Bone-Leiomyo	4d4eff82-ede0-47f1-b49a-ada025952114	14	107063043	107063195	7	152	NA	2	5	NA	NA	NA
Bone-Leiomyo	4d4eff82-ede0-47f1-b49a-ada025952114	X	142748866	142749793	6	927	3	3	NA	NA	NA	NA
Lymph-BNHL	4d72d06d-b6af-4294-9eef-6e61e067761f	2	89139603	89142041	6	2438	NA	1	2	1	2	NA
Lymph-BNHL	4d72d06d-b6af-4294-9eef-6e61e067761f	2	89159090	89185697	126	26607	6	13	19	41	24	23
Lymph-BNHL	4d72d06d-b6af-4294-9eef-6e61e067761f	3	4033702	4038220	6	4518	NA	NA	1	5	NA	NA
Lymph-BNHL	4d72d06d-b6af-4294-9eef-6e61e067761f	3	187460839	187463940	15	3101	1	NA	4	5	3	2
Lymph-BNHL	4d72d06d-b6af-4294-9eef-6e61e067761f	4	92284053	92289108	10	5055	2	1	3	1	2	1
Lymph-BNHL	4d72d06d-b6af-4294-9eef-6e61e067761f	5	45172968	45180725	13	7757	1	NA	4	3	3	2
Lymph-BNHL	4d72d06d-b6af-4294-9eef-6e61e067761f	6	23008508	23016315	9	7807	1	NA	2	3	1	2
Lymph-BNHL	4d72d06d-b6af-4294-9eef-6e61e067761f	8	47458041	47476075	20	18034	2	1	3	5	5	4
Lymph-BNHL	4d72d06d-b6af-4294-9eef-6e61e067761f	9	13444281	13448858	8	4577	1	1	1	1	NA	4
Lymph-BNHL	4d72d06d-b6af-4294-9eef-6e61e067761f	10	19842670	19845492	6	2822	2	NA	NA	1	2	1
Lymph-BNHL	4d72d06d-b6af-4294-9eef-6e61e067761f	13	54371766	54375829	6	4063	3	NA	NA	2	NA	1
Lymph-BNHL	4d72d06d-b6af-4294-9eef-6e61e067761f	14	106112507	106114416	11	1909	2	1	6	1	NA	1
Lymph-BNHL	4d72d06d-b6af-4294-9eef-6e61e067761f	14	106325233	106330442	93	5209	8	23	29	14	10	9
Lymph-BNHL	4d72d06d-b6af-4294-9eef-6e61e067761f	14	106725483	106733306	28	7823	1	3	10	4	8	2
Lymph-BNHL	4d72d06d-b6af-4294-9eef-6e61e067761f	18	60827704	60828642	6	938	NA	NA	2	2	1	1
Lymph-BNHL	4d72d06d-b6af-4294-9eef-6e61e067761f	18	60984301	60988353	50	4052	NA	7	13	15	9	6
Lymph-BNHL	4d72d06d-b6af-4294-9eef-6e61e067761f	22	19638017	19638152	6	135	NA	NA	NA	1	1	4
Lymph-BNHL	4d72d06d-b6af-4294-9eef-6e61e067761f	22	23223177	23233030	30	9853	2	2	8	7	4	7
Lymph-BNHL	4d72d06d-b6af-4294-9eef-6e61e067761f	X	9592114	9592321	6	207	NA	1	NA	2	NA	3
Liver-HCC	4dac9498-c623-11e3-bf01-24c6515278c0	Y	8680933	8681285	7	352	NA	6	1	NA	NA	NA
Panc-AdenoCA	4db9e471-0068-4ec6-9bcb-33ef5fdec33e	6	48248584	48249501	7	917	1	3	3	NA	NA	NA
Panc-AdenoCA	4db9e471-0068-4ec6-9bcb-33ef5fdec33e	6	62838912	62840327	9	1415	NA	4	5	NA	NA	NA
Lymph-BNHL	4e7e6e1f-c648-446f-bdf6-0b1fcc6dfa83	1	115601925	115603876	8	1951	NA	2	3	1	1	1
Lymph-BNHL	4e7e6e1f-c648-446f-bdf6-0b1fcc6dfa83	2	89157302	89197349	192	40047	6	30	29	66	32	29
Lymph-BNHL	4e7e6e1f-c648-446f-bdf6-0b1fcc6dfa83	3	187957838	187959559	10	1721	NA	2	1	NA	1	6
Lymph-BNHL	4e7e6e1f-c648-446f-bdf6-0b1fcc6dfa83	4	189462145	189465130	6	2985	NA	NA	NA	3	3	NA
Lymph-BNHL	4e7e6e1f-c648-446f-bdf6-0b1fcc6dfa83	7	40875800	40876453	9	653	NA	NA	NA	7	1	1
Lymph-BNHL	4e7e6e1f-c648-446f-bdf6-0b1fcc6dfa83	14	106067876	106070788	12	2912	2	4	6	NA	NA	NA
Lymph-BNHL	4e7e6e1f-c648-446f-bdf6-0b1fcc6dfa83	14	106112488	106114288	9	1800	1	1	7	NA	NA	NA
Lymph-BNHL	4e7e6e1f-c648-446f-bdf6-0b1fcc6dfa83	14	106322351	106329878	88	7527	10	11	35	18	7	7
Lymph-BNHL	4e7e6e1f-c648-446f-bdf6-0b1fcc6dfa83	14	106994147	106994889	28	742	2	6	4	6	4	6
Lymph-BNHL	4e7e6e1f-c648-446f-bdf6-0b1fcc6dfa83	18	60984127	60988519	32	4392	6	5	8	10	NA	3
Lymph-BNHL	4e7e6e1f-c648-446f-bdf6-0b1fcc6dfa83	22	23198216	23199080	19	864	1	5	6	4	2	1
Lymph-BNHL	4e7e6e1f-c648-446f-bdf6-0b1fcc6dfa83	22	23223095	23232255	22	9160	2	3	3	6	1	7
Skin-Melanoma	4e8396f7-9506-4401-96b6-bb2e89557d59	1	147414220	147414733	6	513	1	NA	5	NA	NA	NA
Skin-Melanoma	4e8396f7-9506-4401-96b6-bb2e89557d59	2	154846620	154847066	6	446	1	NA	5	NA	NA	NA
Skin-Melanoma	4e8396f7-9506-4401-96b6-bb2e89557d59	2	155106032	155110634	13	4602	NA	1	10	2	NA	NA
Skin-Melanoma	4e8396f7-9506-4401-96b6-bb2e89557d59	4	43115717	43118229	6	2512	1	2	2	1	NA	NA
Skin-Melanoma	4e8396f7-9506-4401-96b6-bb2e89557d59	5	103686638	103693322	8	6684	NA	7	1	NA	NA	NA
Skin-Melanoma	4e8396f7-9506-4401-96b6-bb2e89557d59	7	150134669	150134956	8	287	1	3	4	NA	NA	NA

Skin-Melanoma	4e8396f7-9506-4401-96b6-bb2e89557d59	10	5474640	5476868	8	2228	1	2	5	NA	NA	NA
Skin-Melanoma	4e8396f7-9506-4401-96b6-bb2e89557d59	17	51694145	51698687	8	4542	1	2	5	NA	NA	NA
Skin-Melanoma	4e8396f7-9506-4401-96b6-bb2e89557d59	22	49807366	49812904	10	5538	NA	3	7	NA	NA	NA
Breast-AdenoCa	4e84eed6-82a8-4e91-b0fd-61ec6ef69ce9	1	199513764	199514016	8	252	NA	6	2	NA	NA	NA
Breast-AdenoCa	4e84eed6-82a8-4e91-b0fd-61ec6ef69ce9	1	222467318	222471219	11	3901	1	6	4	NA	NA	NA
Breast-AdenoCa	4e84eed6-82a8-4e91-b0fd-61ec6ef69ce9	10	43739015	43740074	6	1059	NA	3	2	1	NA	NA
Breast-AdenoCa	4e84eed6-82a8-4e91-b0fd-61ec6ef69ce9	21	17182758	17183906	6	1148	NA	4	2	NA	NA	NA
Breast-AdenoCa	4e84eed6-82a8-4e91-b0fd-61ec6ef69ce9	21	23684100	23687645	12	3545	NA	6	6	NA	NA	NA
Breast-AdenoCa	4e84eed6-82a8-4e91-b0fd-61ec6ef69ce9	X	93227676	93230769	7	3093	1	2	4	NA	NA	NA
Ovary-AdenoCA	4e913899-eeb8-4630-ba9d-41a81ced8069	1	111504534	111507690	6	3156	NA	1	5	NA	NA	NA
Ovary-AdenoCA	4e913899-eeb8-4630-ba9d-41a81ced8069	11	71791852	71792005	6	153	NA	3	3	NA	NA	NA
Ovary-AdenoCA	4e913899-eeb8-4630-ba9d-41a81ced8069	16	70474319	70480924	8	6605	NA	6	2	NA	NA	NA
Liver-HCC	4ed52e0c-c623-11e3-bf01-24c6515278c0	4	11974145	11977755	7	3610	NA	NA	NA	1	6	NA
Liver-HCC	4ed52e0c-c623-11e3-bf01-24c6515278c0	5	168911103	168911858	7	755	1	4	2	NA	NA	NA
Biliary-AdenoCA	4ef20a13-f107-4654-adca-fc60b8f5be47	2	78095184	78098172	8	2988	1	5	2	NA	NA	NA
Biliary-AdenoCA	4ef20a13-f107-4654-adca-fc60b8f5be47	6	156432745	156440119	10	7374	3	1	5	1	NA	NA
Biliary-AdenoCA	4ef20a13-f107-4654-adca-fc60b8f5be47	14	103376674	103388898	16	12224	1	3	12	NA	NA	NA
Biliary-AdenoCA	4ef20a13-f107-4654-adca-fc60b8f5be47	X	7136176	7137089	6	913	NA	5	1	NA	NA	NA
Liver-HCC	4f1fc78e-733d-4c6e-86b2-dbe1f98c141d	17	10733090	10734736	7	1646	NA	1	NA	4	2	NA
Liver-HCC	4f6f48bc-c622-11e3-bf01-24c6515278c0	2	126650679	126652263	8	1584	NA	NA	NA	4	3	1
Liver-HCC	4f6f48bc-c622-11e3-bf01-24c6515278c0	6	64534566	64537405	17	2839	2	7	8	NA	NA	NA
Ovary-AdenoCA	504cdfc5-6721-43ef-b31e-fdf52f0cd9cd	7	11498086	11498783	10	697	2	2	6	NA	NA	NA
Ovary-AdenoCA	504cdfc5-6721-43ef-b31e-fdf52f0cd9cd	13	63587141	63587848	9	707	1	5	3	NA	NA	NA
Kidney-RCC	50515723-b495-42a9-8750-e3da288bf6a3	3	73101668	73104834	7	3166	2	3	1	NA	1	NA
Kidney-RCC	50a5970d-a0cb-4251-8ab0-74ad0e94be9a	5	100033165	100035520	17	2355	2	NA	15	NA	NA	NA
Kidney-RCC	50a5970d-a0cb-4251-8ab0-74ad0e94be9a	9	125939997	125943397	18	3400	NA	6	12	NA	NA	NA
Biliary-AdenoCA	50e3a421-9741-4cd8-911b-0f47ca518b6a	1	177068911	177075430	28	6519	6	8	14	NA	NA	NA
Biliary-AdenoCA	50e3a421-9741-4cd8-911b-0f47ca518b6a	11	69993601	69994358	18	757	3	6	9	NA	NA	NA
Biliary-AdenoCA	50e3a421-9741-4cd8-911b-0f47ca518b6a	14	40788157	40788644	7	487	NA	NA	NA	NA	4	3
Biliary-AdenoCA	50e3a421-9741-4cd8-911b-0f47ca518b6a	18	71004856	71005188	12	332	NA	NA	12	NA	NA	NA
Liver-HCC	50eb58ba-c623-11e3-bf01-24c6515278c0	12	73819680	73819815	8	135	NA	NA	NA	7	NA	1
Liver-HCC	50eb58ba-c623-11e3-bf01-24c6515278c0	17	66896225	66900719	7	4494	NA	NA	2	3	1	1
Lymph-CLL	513bafce-375c-49b0-ae75-6c607abd05d8	22	22735434	22735666	6	232	2	1	1	NA	2	NA
Panc-AdenoCA	51458c86-0fdd-470e-b059-1ffbffc92a7f	4	189472749	189475192	30	2443	NA	NA	30	NA	NA	NA
Panc-AdenoCA	51458c86-0fdd-470e-b059-1ffbffc92a7f	5	141133524	141134309	10	785	NA	4	6	NA	NA	NA
Panc-AdenoCA	51458c86-0fdd-470e-b059-1ffbffc92a7f	9	74743472	74743660	7	188	NA	NA	7	NA	NA	NA
Panc-AdenoCA	51458c86-0fdd-470e-b059-1ffbffc92a7f	12	66125976	66129404	30	3428	5	5	20	NA	NA	NA
Panc-AdenoCA	51458c86-0fdd-470e-b059-1ffbffc92a7f	12	66158200	66162116	7	3916	NA	3	4	NA	NA	NA
Panc-AdenoCA	51458c86-0fdd-470e-b059-1ffbffc92a7f	15	76702444	76709838	9	7394	NA	3	5	NA	NA	1
Panc-AdenoCA	51458c86-0fdd-470e-b059-1ffbffc92a7f	15	99887831	99889017	15	1186	2	3	10	NA	NA	NA
Panc-AdenoCA	5187e77d-f412-4303-8049-11d1aa1a0235	18	48624691	48626787	13	2096	1	4	8	NA	NA	NA
Panc-AdenoCA	5187e77d-f412-4303-8049-11d1aa1a0235	X	154439357	154443260	8	3903	4	1	3	NA	NA	NA
Prost-AdenoCA	51adf6cf-f422-4d7e-aa46-cc9bf0ef8693	3	22682003	22685156	8	3153	NA	4	4	NA	NA	NA
Prost-AdenoCA	51adf6cf-f422-4d7e-aa46-cc9bf0ef8693	8	16270231	16274568	8	4337	NA	6	2	NA	NA	NA
Prost-AdenoCA	51adf6cf-f422-4d7e-aa46-cc9bf0ef8693	8	103740546	103742965	14	2419	NA	8	6	NA	NA	NA
Prost-AdenoCA	51adf6cf-f422-4d7e-aa46-cc9bf0ef8693	17	49984485	49985160	6	675	NA	3	3	NA	NA	NA
Ovary-AdenoCA	51b25b37-f75c-4380-a0f6-5273e0b7ee33	4	15658258	15658561	6	303	1	1	3	NA	NA	1
Ovary-AdenoCA	51b25b37-f75c-4380-a0f6-5273e0b7ee33	8	70450394	70454758	21	4364	2	3	16	NA	NA	NA
Liver-HCC	520b8ab2-c623-11e3-bf01-24c6515278c0	1	216808912	216812019	14	3107	NA	9	5	NA	NA	NA
Bone-Leiomyo	522dc834-8367-4f49-b93b-fcba91a2de7e	3	79101294	79101359	6	65	NA	NA	6	NA	NA	NA
Skin-Melanoma	524bbd14-1e1f-479b-9a71-d27fce32d8bc	1	245128827	245141572	16	12745	5	4	7	NA	NA	NA
Skin-Melanoma	524bbd14-1e1f-479b-9a71-d27fce32d8bc	3	89184932	89185796	7	864	NA	7	NA	NA	NA	NA
Skin-Melanoma	524bbd14-1e1f-479b-9a71-d27fce32d8bc	5	147595501	147620674	33	25173	3	12	18	NA	NA	NA
Skin-Melanoma	524bbd14-1e1f-479b-9a71-d27fce32d8bc	16	66100715	66104278	7	3563	NA	6	1	NA	NA	NA
Skin-Melanoma	524bbd14-1e1f-479b-9a71-d27fce32d8bc	17	36106618	36108981	9	2363	3	1	5	NA	NA	NA
Skin-Melanoma	524bbd14-1e1f-479b-9a71-d27fce32d8bc	17	43418283	43419014	8	731	1	4	3	NA	NA	NA
Skin-Melanoma	524bbd14-1e1f-479b-9a71-d27fce32d8bc	17	43439609	43446243	31	6634	4	15	12	NA	NA	NA
Skin-Melanoma	524bbd14-1e1f-479b-9a71-d27fce32d8bc	17	47977134	47977372	7	238	2	1	4	NA	NA	NA
Skin-Melanoma	524bbd14-1e1f-479b-9a71-d27fce32d8bc	17	48687636	48735234	66	47598	12	28	24	NA	2	NA
Skin-Melanoma	524bbd14-1e1f-479b-9a71-d27fce32d8bc	17	50167587	50168901	12	1314	NA	3	9	NA	NA	NA

Skin-Melanoma	524bbd14-1e1f-479b-9a71-d27fce32d8bc	17	52845136	52851176	20	6040	2	13	5	NA	NA	NA
Skin-Melanoma	524bbd14-1e1f-479b-9a71-d27fce32d8bc	17	58433544	58437848	10	4304	1	4	5	NA	NA	NA
Skin-Melanoma	524bbd14-1e1f-479b-9a71-d27fce32d8bc	17	58490472	58490673	7	201	NA	NA	7	NA	NA	NA
Skin-Melanoma	524bbd14-1e1f-479b-9a71-d27fce32d8bc	17	59371418	59373885	12	2467	NA	6	6	NA	NA	NA
Skin-Melanoma	524bbd14-1e1f-479b-9a71-d27fce32d8bc	17	61319776	61321749	8	1973	4	1	3	NA	NA	NA
Skin-Melanoma	524bbd14-1e1f-479b-9a71-d27fce32d8bc	17	63624051	63625194	10	1143	2	1	7	NA	NA	NA
Skin-Melanoma	524bbd14-1e1f-479b-9a71-d27fce32d8bc	17	66729983	66730682	10	699	2	4	4	NA	NA	NA
Skin-Melanoma	524bbd14-1e1f-479b-9a71-d27fce32d8bc	17	66893896	66900489	31	6593	4	11	16	NA	NA	NA
Skin-Melanoma	524bbd14-1e1f-479b-9a71-d27fce32d8bc	17	67193774	67202672	19	8898	3	11	5	NA	NA	NA
Skin-Melanoma	524bbd14-1e1f-479b-9a71-d27fce32d8bc	17	67747913	67749585	16	1672	3	10	3	NA	NA	NA
Skin-Melanoma	524bbd14-1e1f-479b-9a71-d27fce32d8bc	17	68724660	68725914	11	1254	3	4	4	NA	NA	NA
Skin-Melanoma	524bbd14-1e1f-479b-9a71-d27fce32d8bc	17	69139515	69157292	35	17777	8	18	9	NA	NA	NA
Skin-Melanoma	524bbd14-1e1f-479b-9a71-d27fce32d8bc	17	74186616	74197194	31	10578	7	12	12	NA	NA	NA
Panc-AdenoCA	5255d645-d3d2-422e-8449-046cd21f4935	3	195322954	195332774	14	9820	7	2	5	NA	NA	NA
Panc-AdenoCA	5255d645-d3d2-422e-8449-046cd21f4935	18	25195363	25199340	9	3977	4	2	2	NA	NA	1
Panc-AdenoCA	5255d645-d3d2-422e-8449-046cd21f4935	19	16816164	16828104	13	11940	5	2	6	NA	NA	NA
Eso-AdenoCa	526b3796-2cbd-4eec-8273-064b41456279	2	237003275	237004737	6	1462	NA	3	3	NA	NA	NA
Eso-AdenoCa	526b3796-2cbd-4eec-8273-064b41456279	4	168488795	168488992	6	197	NA	NA	6	NA	NA	NA
Eso-AdenoCa	526b3796-2cbd-4eec-8273-064b41456279	13	105157491	105158469	7	978	NA	3	3	NA	NA	1
Eso-AdenoCa	526b3796-2cbd-4eec-8273-064b41456279	20	20112095	20117549	10	5454	2	2	6	NA	NA	NA
Lung-AdenoCA	52910a60-bb15-4ba5-9d09-50d8ee6a445b	5	62123396	62124835	10	1439	NA	7	3	NA	NA	NA
Lung-AdenoCA	52910a60-bb15-4ba5-9d09-50d8ee6a445b	8	42369229	42372329	30	3100	NA	10	19	NA	NA	1
Lung-AdenoCA	52910a60-bb15-4ba5-9d09-50d8ee6a445b	8	43686215	43687987	28	1772	3	6	18	NA	NA	1
Panc-AdenoCA	52f43b72-15ea-40a0-a3b4-4bf22bc0bf25	6	69093923	69100353	27	6430	1	11	15	NA	NA	NA
Panc-AdenoCA	52f43b72-15ea-40a0-a3b4-4bf22bc0bf25	17	51856088	51856526	11	438	NA	9	2	NA	NA	NA
Panc-AdenoCA	53534b3c-cd15-4d68-a9b1-6902bb234c45	1	157101639	157102187	7	548	NA	2	5	NA	NA	NA
Panc-AdenoCA	53534b3c-cd15-4d68-a9b1-6902bb234c45	6	129193072	129194610	25	1538	4	17	4	NA	NA	NA
Panc-AdenoCA	53534b3c-cd15-4d68-a9b1-6902bb234c45	8	43626482	43626948	6	466	3	3	NA	NA	NA	NA
Panc-AdenoCA	53534b3c-cd15-4d68-a9b1-6902bb234c45	8	49041231	49041608	10	377	NA	NA	10	NA	NA	NA
Panc-AdenoCA	53534b3c-cd15-4d68-a9b1-6902bb234c45	8	49413948	49414101	8	153	NA	1	7	NA	NA	NA
Panc-AdenoCA	53534b3c-cd15-4d68-a9b1-6902bb234c45	8	68708617	68710090	14	1473	3	3	8	NA	NA	NA
Panc-AdenoCA	53534b3c-cd15-4d68-a9b1-6902bb234c45	8	85533388	85534485	8	1097	1	6	1	NA	NA	NA
Panc-AdenoCA	53534b3c-cd15-4d68-a9b1-6902bb234c45	15	81665932	81668107	10	2175	1	6	3	NA	NA	NA
Panc-AdenoCA	53534b3c-cd15-4d68-a9b1-6902bb234c45	18	33016565	33018120	23	1555	1	12	10	NA	NA	NA
Ovary-AdenoCA	53bcbb68-addd-47fc-9910-cd1d3f38197d	13	113484489	113490433	15	5944	2	3	9	NA	NA	1
Lung-AdenoCA	53d32d8b-5649-42b0-8c52-2c39af2a961b	8	36614508	36618864	7	4356	NA	5	2	NA	NA	NA
CNS-GBM	53dec97d-0464-4ffd-8e2e-95b2b9a03af0	7	55229542	55233043	16	3501	4	8	4	NA	NA	NA
CNS-GBM	53dec97d-0464-4ffd-8e2e-95b2b9a03af0	7	145443694	145445058	9	1364	NA	8	1	NA	NA	NA
CNS-GBM	53dec97d-0464-4ffd-8e2e-95b2b9a03af0	7	145490765	145491638	9	873	NA	5	4	NA	NA	NA
CNS-GBM	53dec97d-0464-4ffd-8e2e-95b2b9a03af0	7	145589491	145593536	22	4045	1	16	5	NA	NA	NA
Liver-HCC	53df19a8-c623-11e3-bf01-24c6515278c0	19	19941598	19945304	6	3706	NA	5	NA	1	NA	NA
Panc-AdenoCA	54195db3-94a9-4538-8bb8-9953d936acd4	8	145077546	145078372	8	826	NA	2	5	1	NA	NA
Panc-AdenoCA	54195db3-94a9-4538-8bb8-9953d936acd4	17	46952302	46952400	6	98	NA	NA	6	NA	NA	NA
Liver-HCC	54cfd4b0-c623-11e3-bf01-24c6515278c0	5	120247005	120249616	6	2611	1	2	1	1	NA	1
Prost-AdenoCA	551d5082-ab6a-4674-af0d-ce408f589949	7	53516632	53516814	6	182	NA	NA	NA	1	5	NA
Prost-AdenoCA	551d5082-ab6a-4674-af0d-ce408f589949	7	107545004	107546012	9	1008	NA	NA	NA	4	5	NA
Ovary-AdenoCA	5533240d-8fed-440d-b781-6092785a77ce	8	55294805	55298756	8	3951	NA	4	4	NA	NA	NA
Ovary-AdenoCA	5533240d-8fed-440d-b781-6092785a77ce	8	55377675	55378971	12	1296	5	5	2	NA	NA	NA
ColoRect-AdenoCA	55345803-83d4-4fdb-b0ee-328753801da1	17	29232696	29233461	13	765	2	3	8	NA	NA	NA
ColoRect-AdenoCA	55345803-83d4-4fdb-b0ee-328753801da1	17	51711603	51713138	14	1535	1	5	8	NA	NA	NA
Uterus-AdenoCA	554b9439-7e11-43ec-8bc3-448c59100b9e	16	32480763	32482117	6	1354	5	1	NA	NA	NA	NA
Uterus-AdenoCA	554b9439-7e11-43ec-8bc3-448c59100b9e	18	4921596	4924061	10	2465	3	4	2	NA	NA	1
Uterus-AdenoCA	554b9439-7e11-43ec-8bc3-448c59100b9e	18	6278413	6284247	29	5834	4	14	11	NA	NA	NA
Uterus-AdenoCA	55ada396-0878-4724-8ebe-00da367eed3d	11	63017266	63017547	7	281	1	2	3	1	NA	NA
Lymph-CLL	55c82198-5be6-4cc5-b5b3-b707af234107	2	89159347	89185520	70	26173	1	11	9	32	10	7
Lymph-CLL	55c82198-5be6-4cc5-b5b3-b707af234107	13	56757557	56760481	7	2924	1	NA	1	1	1	3
Lymph-CLL	55c82198-5be6-4cc5-b5b3-b707af234107	13	85463941	85470090	8	6149	1	NA	NA	1	2	4
Lymph-CLL	55c82198-5be6-4cc5-b5b3-b707af234107	14	106326444	106330146	40	3702	3	10	4	12	5	6
Lymph-CLL	55c82198-5be6-4cc5-b5b3-b707af234107	14	106379279	106382359	18	3080	NA	NA	5	11	1	1
Lymph-CLL	55c82198-5be6-4cc5-b5b3-b707af234107	14	106539205	106539681	12	476	1	NA	3	6	2	NA

Lymph-CLL	55c82198-5be6-4cc5-b5b3-b707af234107	22	23227724	23231205	10	3481	1	1	1	3	2	2
Panc-AdenoCA	5650dc4e-8bce-4716-9199-cd5750d7a888	10	27333171	27336616	49	3445	NA	NA	49	NA	NA	NA
Panc-AdenoCA	5650dc4e-8bce-4716-9199-cd5750d7a888	11	33041447	33044690	8	3243	2	1	5	NA	NA	NA
Panc-AdenoCA	5650dc4e-8bce-4716-9199-cd5750d7a888	11	34587834	34598174	39	10340	1	1	37	NA	NA	NA
Panc-AdenoCA	5650dc4e-8bce-4716-9199-cd5750d7a888	12	30229022	30232458	32	3436	1	8	23	NA	NA	NA
Panc-AdenoCA	5650dc4e-8bce-4716-9199-cd5750d7a888	12	77376586	77378563	17	1977	2	7	6	1	1	NA
Panc-AdenoCA	5650dc4e-8bce-4716-9199-cd5750d7a888	18	18896042	18898157	12	2115	NA	NA	12	NA	NA	NA
Panc-AdenoCA	5650dc4e-8bce-4716-9199-cd5750d7a888	18	22147117	22147510	8	393	NA	NA	8	NA	NA	NA
Biliary-AdenoCA	56cc8740-b132-4cbc-b513-f0b5b2305ca6	1	153237237	153248660	18	11423	NA	9	8	NA	1	NA
Biliary-AdenoCA	56cc8740-b132-4cbc-b513-f0b5b2305ca6	1	160986308	160991954	9	5646	2	6	1	NA	NA	NA
Biliary-AdenoCA	56cc8740-b132-4cbc-b513-f0b5b2305ca6	17	39784903	39788451	26	3548	2	6	18	NA	NA	NA
Biliary-AdenoCA	56cc8740-b132-4cbc-b513-f0b5b2305ca6	18	20031785	20036921	15	5136	4	7	4	NA	NA	NA
Biliary-AdenoCA	56cc8740-b132-4cbc-b513-f0b5b2305ca6	19	9664951	9670594	9	5643	2	6	1	NA	NA	NA
Biliary-AdenoCA	56cc8740-b132-4cbc-b513-f0b5b2305ca6	19	43672953	43673516	8	563	3	2	3	NA	NA	NA
Biliary-AdenoCA	56cc8740-b132-4cbc-b513-f0b5b2305ca6	19	44522567	44524959	11	2392	2	8	1	NA	NA	NA
Skin-Melanoma	56ddcb96-e90b-4fce-aa4c-c677bbfee488	5	3908969	3910423	12	1454	NA	6	6	NA	NA	NA
Skin-Melanoma	56ddcb96-e90b-4fce-aa4c-c677bbfee488	5	15070805	15074697	20	3892	2	10	6	1	1	NA
Skin-Melanoma	56ddcb96-e90b-4fce-aa4c-c677bbfee488	5	20484756	20489012	8	4256	NA	6	2	NA	NA	NA
Skin-Melanoma	56ddcb96-e90b-4fce-aa4c-c677bbfee488	5	35151376	35152002	8	626	2	3	3	NA	NA	NA
Skin-Melanoma	56ddcb96-e90b-4fce-aa4c-c677bbfee488	5	120210904	120212882	13	1978	1	7	4	1	NA	NA
Skin-Melanoma	56ddcb96-e90b-4fce-aa4c-c677bbfee488	7	27824708	27826099	6	1391	NA	4	2	NA	NA	NA
Skin-Melanoma	56ddcb96-e90b-4fce-aa4c-c677bbfee488	7	40679457	40684245	8	4788	NA	6	2	NA	NA	NA
Skin-Melanoma	56ddcb96-e90b-4fce-aa4c-c677bbfee488	7	48179380	48179831	9	451	NA	6	3	NA	NA	NA
Skin-Melanoma	56ddcb96-e90b-4fce-aa4c-c677bbfee488	7	54424642	54432358	11	7716	NA	6	4	NA	NA	1
Skin-Melanoma	56ddcb96-e90b-4fce-aa4c-c677bbfee488	7	81480362	81480785	10	423	1	5	4	NA	NA	NA
Lymph-BNHL	56e57223-264e-489d-bc9a-a07789065c40	4	61254862	61257670	6	2808	NA	2	2	NA	1	1
Lymph-BNHL	56e57223-264e-489d-bc9a-a07789065c40	4	136343505	136360540	24	17035	1	1	5	7	3	7
Lymph-BNHL	56e57223-264e-489d-bc9a-a07789065c40	7	147085956	147086950	6	994	NA	NA	NA	4	1	1
Lymph-BNHL	56e57223-264e-489d-bc9a-a07789065c40	12	122459308	122461118	7	1810	NA	2	4	NA	NA	1
Lymph-BNHL	56e57223-264e-489d-bc9a-a07789065c40	14	106069180	106069963	9	783	1	4	4	NA	NA	NA
Lymph-BNHL	56e57223-264e-489d-bc9a-a07789065c40	14	106112755	106114301	10	1546	NA	2	8	NA	NA	NA
Lymph-BNHL	56e57223-264e-489d-bc9a-a07789065c40	14	106237519	106241249	18	3730	3	4	11	NA	NA	NA
Lymph-BNHL	56e57223-264e-489d-bc9a-a07789065c40	14	106322746	106329550	61	6804	4	9	18	14	3	13
Lymph-BNHL	56e57223-264e-489d-bc9a-a07789065c40	14	106829927	106831081	12	1154	NA	1	2	4	4	1
Lymph-BNHL	56e57223-264e-489d-bc9a-a07789065c40	18	60984007	60988535	41	4528	3	12	16	5	3	2
Lymph-BNHL	56e57223-264e-489d-bc9a-a07789065c40	22	22723412	22724243	12	831	1	2	1	5	2	1
CNS-GBM	56ffaa35-814c-4c0b-b3c6-d4514d34fec2	11	34630518	34639323	14	8805	NA	10	4	NA	NA	NA
CNS-GBM	56ffaa35-814c-4c0b-b3c6-d4514d34fec2	11	44814196	44819019	9	4823	NA	3	6	NA	NA	NA
Stomach-AdenoCA	576a1741-8c65-40da-9f0f-b2ca805d70f3	19	14181133	14181782	6	649	NA	NA	1	5	NA	NA
Stomach-AdenoCA	576a1741-8c65-40da-9f0f-b2ca805d70f3	X	11735241	11735437	10	196	NA	NA	NA	10	NA	NA
Lymph-BNHL	578ba169-1d2f-4187-b174-082bbc6a5bd2	2	89158911	89160367	17	1456	NA	4	3	8	1	1
Lymph-BNHL	578ba169-1d2f-4187-b174-082bbc6a5bd2	2	181485930	181487954	8	2024	3	2	3	NA	NA	NA
Lymph-BNHL	578ba169-1d2f-4187-b174-082bbc6a5bd2	4	183486102	183486876	6	774	NA	NA	NA	3	1	2
Lymph-BNHL	578ba169-1d2f-4187-b174-082bbc6a5bd2	8	128747987	128750554	19	2567	4	2	8	4	NA	1
Lymph-BNHL	578ba169-1d2f-4187-b174-082bbc6a5bd2	14	106325371	106330416	78	5045	6	22	31	12	3	4
Lymph-BNHL	578ba169-1d2f-4187-b174-082bbc6a5bd2	14	107218834	107218986	11	152	NA	1	6	2	NA	2
Lymph-BNHL	578ba169-1d2f-4187-b174-082bbc6a5bd2	18	64277942	64280301	6	2359	NA	NA	NA	4	1	1
Lymph-BNHL	578ba169-1d2f-4187-b174-082bbc6a5bd2	22	23223232	23231597	18	8365	1	3	3	6	2	3
Panc-Endocrine	589e0aea-9057-4b8c-8af1-74c74fe46a38	3	1641727	1649310	17	7583	NA	9	8	NA	NA	NA
Panc-Endocrine	589e0aea-9057-4b8c-8af1-74c74fe46a38	20	13979678	13983885	6	4207	1	4	1	NA	NA	NA
Panc-Endocrine	589e0aea-9057-4b8c-8af1-74c74fe46a38	20	14957408	14958583	8	1175	NA	7	1	NA	NA	NA
Skin-Melanoma	58d3e932-b4a3-4cac-a6ac-73160e354a15	6	64132636	64135852	9	3216	1	8	NA	NA	NA	NA
Skin-Melanoma	58d3e932-b4a3-4cac-a6ac-73160e354a15	11	57998508	58002542	19	4034	1	13	5	NA	NA	NA
Skin-Melanoma	58d3e932-b4a3-4cac-a6ac-73160e354a15	11	69705460	69707527	7	2067	1	NA	6	NA	NA	NA
Skin-Melanoma	58d3e932-b4a3-4cac-a6ac-73160e354a15	11	73142908	73144426	8	1518	NA	4	3	1	NA	NA
Skin-Melanoma	58d3e932-b4a3-4cac-a6ac-73160e354a15	11	75097916	75098266	7	350	NA	6	1	NA	NA	NA
Skin-Melanoma	58d3e932-b4a3-4cac-a6ac-73160e354a15	11	75722851	75727546	15	4695	6	2	6	NA	1	NA
Skin-Melanoma	58d3e932-b4a3-4cac-a6ac-73160e354a15	11	76814200	76818860	7	4660	NA	2	5	NA	NA	NA
Skin-Melanoma	58d3e932-b4a3-4cac-a6ac-73160e354a15	11	78459403	78460242	9	839	1	NA	8	NA	NA	NA
Skin-Melanoma	58d3e932-b4a3-4cac-a6ac-73160e354a15	11	81275618	81281191	8	5573	NA	2	5	NA	NA	1

Skin-Melanoma	58d3e932-b4a3-4cac-a6ac-73160e354a15	11	84848995	84849665	7	670	1	3	3	NA	NA	NA
Skin-Melanoma	58d3e932-b4a3-4cac-a6ac-73160e354a15	11	86204384	86204939	8	555	1	4	3	NA	NA	NA
Skin-Melanoma	58d3e932-b4a3-4cac-a6ac-73160e354a15	11	87771588	87776491	10	4903	3	1	6	NA	NA	NA
Skin-Melanoma	58d3e932-b4a3-4cac-a6ac-73160e354a15	11	88667435	88671400	16	3965	1	1	14	NA	NA	NA
Skin-Melanoma	58d3e932-b4a3-4cac-a6ac-73160e354a15	11	93645589	93646222	8	633	2	NA	6	NA	NA	NA
Skin-Melanoma	58d3e932-b4a3-4cac-a6ac-73160e354a15	11	93735359	93741925	18	6566	1	13	3	1	NA	NA
Skin-Melanoma	58d3e932-b4a3-4cac-a6ac-73160e354a15	11	97421231	97421415	8	184	NA	NA	8	NA	NA	NA
Liver-HCC	59632e7e-c622-11e3-bf01-24c6515278c0	4	97191537	97195384	6	3847	NA	2	4	NA	NA	NA
Biliary-AdenoCA	5975deb5-00d2-4d18-b5c0-34ac9947fe11	17	27095079	27095764	6	685	NA	2	4	NA	NA	NA
Biliary-AdenoCA	5a51a6fc-c623-11e3-bf01-24c6515278c0	7	41110155	41116007	11	5852	NA	NA	NA	8	1	2
ColoRect-AdenoCA	5b180356-cf58-4fad-a3d4-00fc12b43fcc	17	28134258	28134678	9	420	1	4	4	NA	NA	NA
ColoRect-AdenoCA	5b180356-cf58-4fad-a3d4-00fc12b43fcc	17	61330503	61331059	7	556	NA	3	4	NA	NA	NA
ColoRect-AdenoCA	5b180356-cf58-4fad-a3d4-00fc12b43fcc	17	65211878	65212088	9	210	3	2	3	NA	NA	1
ColoRect-AdenoCA	5b180356-cf58-4fad-a3d4-00fc12b43fcc	X	53716263	53749478	40	33215	8	21	9	1	1	NA
Panc-AdenoCA	5b41e033-6df8-411e-a13b-59eeb8afbf9b	3	82780348	82782891	10	2543	3	4	3	NA	NA	NA
Panc-AdenoCA	5b41e033-6df8-411e-a13b-59eeb8afbf9b	4	89018269	89021588	11	3319	1	7	3	NA	NA	NA
Lymph-CLL	5b4b2312-acb5-4329-8d46-7f93213e3daf	2	89159647	89185612	68	25965	5	8	7	24	14	10
Lymph-CLL	5b4b2312-acb5-4329-8d46-7f93213e3daf	14	106323947	106375251	79	51304	2	16	12	29	11	9
Lymph-CLL	5b4b2312-acb5-4329-8d46-7f93213e3daf	14	106725264	106733429	24	8165	1	9	3	8	NA	3
Lymph-CLL	5b4b2312-acb5-4329-8d46-7f93213e3daf	22	23134868	23135439	12	571	1	4	2	4	1	NA
Breast-AdenoCa	5b7d5767-2037-4175-aec9-886776343181	4	61166004	61166603	30	599	NA	NA	30	NA	NA	NA
Breast-AdenoCa	5b7d5767-2037-4175-aec9-886776343181	4	63270587	63277325	24	6738	NA	2	22	NA	NA	NA
Breast-AdenoCa	5b7d5767-2037-4175-aec9-886776343181	6	104702005	104730314	32	28309	NA	13	19	NA	NA	NA
Breast-AdenoCa	5b7d5767-2037-4175-aec9-886776343181	8	51592776	51594392	6	1616	1	5	NA	NA	NA	NA
Breast-AdenoCa	5b7d5767-2037-4175-aec9-886776343181	10	11556790	11558789	14	1999	2	5	7	NA	NA	NA
Breast-AdenoCa	5b7d5767-2037-4175-aec9-886776343181	10	22012362	22013603	11	1241	3	4	4	NA	NA	NA
Breast-AdenoCa	5b7d5767-2037-4175-aec9-886776343181	11	66175383	66180331	7	4948	2	4	1	NA	NA	NA
Breast-AdenoCa	5b7d5767-2037-4175-aec9-886776343181	17	25412016	25412370	6	354	NA	NA	6	NA	NA	NA
Breast-AdenoCa	5b7d5767-2037-4175-aec9-886776343181	17	38230260	38231427	8	1167	NA	5	3	NA	NA	NA
Breast-AdenoCa	5b7d5767-2037-4175-aec9-886776343181	17	38588818	38603781	25	14963	1	9	15	NA	NA	NA
Breast-AdenoCa	5b7d5767-2037-4175-aec9-886776343181	17	47988170	47994069	19	5899	1	14	4	NA	NA	NA
Breast-AdenoCa	5b7d5767-2037-4175-aec9-886776343181	18	30443991	30444098	6	107	NA	2	4	NA	NA	NA
Breast-AdenoCa	5b7d5767-2037-4175-aec9-886776343181	20	30819700	30821297	7	1597	1	1	5	NA	NA	NA
Breast-AdenoCa	5b7d5767-2037-4175-aec9-886776343181	20	30862683	30865396	34	2713	NA	1	33	NA	NA	NA
Breast-AdenoCa	5b7d5767-2037-4175-aec9-886776343181	20	32073006	32073932	13	926	NA	NA	13	NA	NA	NA
Breast-AdenoCa	5b7d5767-2037-4175-aec9-886776343181	20	34175831	34176348	7	517	NA	NA	7	NA	NA	NA
Breast-AdenoCa	5b7d5767-2037-4175-aec9-886776343181	22	24423873	24424354	6	481	1	3	2	NA	NA	NA
Breast-AdenoCa	5b7d5767-2037-4175-aec9-886776343181	22	31056538	31057376	12	838	NA	1	9	1	NA	1
Breast-AdenoCa	5b7d5767-2037-4175-aec9-886776343181	22	43260279	43260986	12	707	NA	2	9	1	NA	NA
Breast-AdenoCa	5b7d5767-2037-4175-aec9-886776343181	22	46647876	46648453	7	577	1	4	1	NA	NA	1
Lymph-BNHL	5bcf473f-417d-47a5-9745-ac7faf830b1f	2	89159466	89181663	66	22197	8	11	11	14	12	10
Lymph-BNHL	5bcf473f-417d-47a5-9745-ac7faf830b1f	14	106112481	106114297	14	1816	3	2	9	NA	NA	NA
Lymph-BNHL	5bcf473f-417d-47a5-9745-ac7faf830b1f	14	106326074	106330160	60	4086	4	9	18	6	11	12
Lymph-BNHL	5bcf473f-417d-47a5-9745-ac7faf830b1f	16	46821863	46821984	6	121	NA	NA	NA	NA	3	3
Lymph-BNHL	5bcf473f-417d-47a5-9745-ac7faf830b1f	18	60985096	60988230	31	3134	3	9	8	5	3	3
Lymph-BNHL	5bcf473f-417d-47a5-9745-ac7faf830b1f	22	23223343	23232008	14	8665	NA	3	7	NA	3	1
Kidney-RCC	5c156f63-6537-4d93-a6c2-4155618cf638	18	4652607	4653250	8	643	1	4	3	NA	NA	NA
Prost-AdenoCA	5c8da06c-7964-4ad1-ac9e-d1b5507105d2	5	136644608	136649064	10	4456	1	5	4	NA	NA	NA
Biliary-AdenoCA	5c9b8a2c-c623-11e3-bf01-24c6515278c0	11	75644634	75645455	8	821	2	3	2	1	NA	NA
Biliary-AdenoCA	5c9b8a2c-c623-11e3-bf01-24c6515278c0	11	77172085	77180866	25	8781	NA	2	23	NA	NA	NA
Ovary-AdenoCA	5cc7c966-499e-49e3-b509-4eeb00ee03d4	10	70308504	70309770	17	1266	2	6	9	NA	NA	NA
Ovary-AdenoCA	5cc7c966-499e-49e3-b509-4eeb00ee03d4	12	41355996	41358061	10	2065	1	9	NA	NA	NA	NA
Liver-HCC	5cf21488-c622-11e3-bf01-24c6515278c0	4	31817011	31819820	8	2809	NA	NA	NA	6	NA	2
Panc-AdenoCA	5d9ff58c-5702-48fc-a66a-d1276327fd49	10	9257957	9258302	7	345	1	2	4	NA	NA	NA
Panc-AdenoCA	5d9ff58c-5702-48fc-a66a-d1276327fd49	10	20244115	20244976	15	861	3	4	8	NA	NA	NA
Panc-AdenoCA	5d9ff58c-5702-48fc-a66a-d1276327fd49	12	100439977	100441866	6	1889	2	3	1	NA	NA	NA
Panc-AdenoCA	5d9ff58c-5702-48fc-a66a-d1276327fd49	15	100637253	100638065	10	812	2	5	3	NA	NA	NA
Breast-AdenoCa	5dbf3203-ce73-41e4-bf9a-32fc856f73f5	17	32346291	32347110	6	819	NA	3	3	NA	NA	NA
Breast-AdenoCa	5dbf3203-ce73-41e4-bf9a-32fc856f73f5	20	3495168	3496890	6	1722	NA	4	2	NA	NA	NA
Breast-AdenoCa	5dbf3203-ce73-41e4-bf9a-32fc856f73f5	21	15831010	15832370	6	1360	NA	5	1	NA	NA	NA

Liver-HCC	5dce221a-c623-11e3-bf01-24c6515278c0	12	99850693	99853226	8	2533	NA	2	6	NA	NA	NA
Liver-HCC	5dce221a-c623-11e3-bf01-24c6515278c0	12	117579537	117581616	11	2079	1	5	5	NA	NA	NA
Liver-HCC	5dce221a-c623-11e3-bf01-24c6515278c0	15	38855709	38862093	13	6384	2	7	4	NA	NA	NA
Liver-HCC	5df489bc-6178-49e4-8a42-506f70300dff	2	160212494	160215815	9	3321	NA	NA	NA	4	5	NA
Liver-HCC	5df489bc-6178-49e4-8a42-506f70300dff	8	43498391	43499116	12	725	NA	NA	NA	4	5	3
Liver-HCC	5df489bc-6178-49e4-8a42-506f70300dff	9	92328350	92328688	6	338	NA	NA	NA	3	2	1
Liver-HCC	5df489bc-6178-49e4-8a42-506f70300dff	9	104936894	104937415	11	521	NA	NA	NA	4	5	2
Liver-HCC	5ead73fe-1c34-48ed-b20d-89fc3c82dbd6	6	65122963	65123394	11	431	NA	NA	11	NA	NA	NA
Kidney-RCC	5ecc88f7-8391-4168-af11-07a6bf9b3652	2	130069176	130070973	8	1797	2	NA	2	2	NA	2
Lymph-BNHL	5f18b334-6616-406f-8f33-0592b56c14e6	2	89159929	89187465	45	27536	1	8	14	13	3	6
Lymph-BNHL	5f18b334-6616-406f-8f33-0592b56c14e6	13	59212502	59213649	6	1147	NA	NA	2	2	2	NA
Lymph-BNHL	5f18b334-6616-406f-8f33-0592b56c14e6	14	106325362	106330061	30	4699	1	9	8	5	3	4
Lymph-BNHL	5f18b334-6616-406f-8f33-0592b56c14e6	18	60984294	60988189	14	3895	NA	NA	6	7	NA	1
Lymph-BNHL	5f18b334-6616-406f-8f33-0592b56c14e6	22	23227689	23231899	16	4210	NA	2	9	4	NA	1
Lymph-BNHL	5f18b334-6616-406f-8f33-0592b56c14e6	X	9018631	9022553	6	3922	1	NA	NA	1	NA	4
Lymph-CLL	5f53bcda-2fd3-4574-9cc2-5efe353372b2	14	106323957	106326713	12	2756	NA	5	7	NA	NA	NA
Eso-AdenoCa	5fd632ea-085e-4e9b-8dcc-ec94e492d3bf	6	7606136	7611563	18	5427	3	1	14	NA	NA	NA
CNS-GBM	5fd77ba9-5015-4d8b-86a0-582e5c76bdd6	9	30495163	30497436	7	2273	1	1	1	3	NA	1
CNS-GBM	5fd77ba9-5015-4d8b-86a0-582e5c76bdd6	12	57971922	57977675	7	5753	1	1	4	NA	NA	1
CNS-GBM	5fd77ba9-5015-4d8b-86a0-582e5c76bdd6	12	58769344	58773864	8	4520	NA	1	7	NA	NA	NA
CNS-GBM	5fd77ba9-5015-4d8b-86a0-582e5c76bdd6	12	58812133	58821294	11	9161	2	1	7	1	NA	NA
CNS-GBM	5fd77ba9-5015-4d8b-86a0-582e5c76bdd6	12	59023707	59036477	14	12770	NA	1	12	1	NA	NA
CNS-GBM	5fd77ba9-5015-4d8b-86a0-582e5c76bdd6	12	59087658	59093152	7	5494	1	NA	4	1	NA	1
CNS-GBM	5fd77ba9-5015-4d8b-86a0-582e5c76bdd6	12	59133161	59145595	14	12434	3	2	6	1	NA	2
CNS-GBM	5fd77ba9-5015-4d8b-86a0-582e5c76bdd6	12	59373127	59382415	14	9288	1	3	5	2	3	NA
CNS-GBM	5fd77ba9-5015-4d8b-86a0-582e5c76bdd6	12	59961779	59967550	7	5771	1	NA	2	1	3	NA
CNS-GBM	5fd77ba9-5015-4d8b-86a0-582e5c76bdd6	X	141260317	141265246	7	4929	NA	NA	NA	6	NA	1
Kidney-RCC	603594e3-e427-494c-b9ee-e872d6629563	2	144138567	144139578	6	1011	2	3	1	NA	NA	NA
Panc-AdenoCA	60aff7a0-4960-4ed1-922b-3233ccbfdba1	8	29083517	29084562	10	1045	2	4	4	NA	NA	NA
Panc-AdenoCA	60aff7a0-4960-4ed1-922b-3233ccbfdba1	8	29578910	29579291	7	381	NA	4	3	NA	NA	NA
Panc-AdenoCA	60aff7a0-4960-4ed1-922b-3233ccbfdba1	8	29780557	29781232	7	675	1	5	1	NA	NA	NA
Panc-AdenoCA	60aff7a0-4960-4ed1-922b-3233ccbfdba1	9	37377766	37379642	11	1876	1	4	6	NA	NA	NA
Panc-AdenoCA	60aff7a0-4960-4ed1-922b-3233ccbfdba1	18	23238486	23238657	7	171	3	3	1	NA	NA	NA
Panc-AdenoCA	60aff7a0-4960-4ed1-922b-3233ccbfdba1	19	39934052	39937395	13	3343	2	4	7	NA	NA	NA
Panc-AdenoCA	60aff7a0-4960-4ed1-922b-3233ccbfdba1	21	18026828	18035592	27	8764	1	8	18	NA	NA	NA
Panc-AdenoCA	60aff7a0-4960-4ed1-922b-3233ccbfdba1	21	18171647	18181167	31	9520	4	10	17	NA	NA	NA
Panc-AdenoCA	60aff7a0-4960-4ed1-922b-3233ccbfdba1	21	39636713	39638344	7	1631	NA	NA	4	1	1	1
Panc-AdenoCA	60aff7a0-4960-4ed1-922b-3233ccbfdba1	22	21026974	21027161	6	187	1	2	3	NA	NA	NA
Panc-AdenoCA	60aff7a0-4960-4ed1-922b-3233ccbfdba1	22	21797074	21800472	17	3398	3	6	8	NA	NA	NA
Panc-AdenoCA	60aff7a0-4960-4ed1-922b-3233ccbfdba1	22	45637689	45639213	9	1524	1	5	3	NA	NA	NA
Panc-AdenoCA	60c33e32-7e19-4e71-b075-a63fcf27e660	1	2353486	2354982	6	1496	NA	3	3	NA	NA	NA
Panc-AdenoCA	60c33e32-7e19-4e71-b075-a63fcf27e660	3	87570029	87572868	8	2839	1	4	3	NA	NA	NA
Panc-AdenoCA	60c33e32-7e19-4e71-b075-a63fcf27e660	12	4538276	4541851	6	3575	NA	2	4	NA	NA	NA
Panc-AdenoCA	60c33e32-7e19-4e71-b075-a63fcf27e660	20	2911859	2913206	7	1347	2	2	3	NA	NA	NA
Panc-AdenoCA	60c33e32-7e19-4e71-b075-a63fcf27e660	21	20137823	20139824	10	2001	NA	3	7	NA	NA	NA
Panc-AdenoCA	60c33e32-7e19-4e71-b075-a63fcf27e660	21	31480476	31481508	12	1032	2	4	6	NA	NA	NA
Panc-AdenoCA	60c33e32-7e19-4e71-b075-a63fcf27e660	X	49543570	49544826	11	1256	1	5	5	NA	NA	NA
Panc-AdenoCA	60e78c80-41a5-456a-9a81-9007d18fd72c	1	181521339	181524196	10	2857	NA	4	6	NA	NA	NA
Panc-AdenoCA	60e78c80-41a5-456a-9a81-9007d18fd72c	1	247457508	247457856	19	348	NA	3	16	NA	NA	NA
Panc-AdenoCA	60e78c80-41a5-456a-9a81-9007d18fd72c	3	20022247	20024629	18	2382	1	7	10	NA	NA	NA
Panc-AdenoCA	60e78c80-41a5-456a-9a81-9007d18fd72c	3	22084108	22085782	14	1674	NA	5	8	NA	1	NA
Panc-AdenoCA	60e78c80-41a5-456a-9a81-9007d18fd72c	7	25731005	25734787	11	3782	NA	5	6	NA	NA	NA
Panc-AdenoCA	60e78c80-41a5-456a-9a81-9007d18fd72c	9	10378225	10378750	8	525	NA	3	5	NA	NA	NA
Panc-AdenoCA	60e78c80-41a5-456a-9a81-9007d18fd72c	11	111742226	111742366	6	140	NA	2	4	NA	NA	NA
Panc-AdenoCA	60e78c80-41a5-456a-9a81-9007d18fd72c	18	33497695	33498679	9	984	2	4	3	NA	NA	NA
Panc-AdenoCA	60e78c80-41a5-456a-9a81-9007d18fd72c	19	12512167	12513503	7	1336	NA	4	3	NA	NA	NA
Panc-AdenoCA	60e78c80-41a5-456a-9a81-9007d18fd72c	19	13261191	13262758	12	1567	3	6	3	NA	NA	NA
Panc-AdenoCA	60e78c80-41a5-456a-9a81-9007d18fd72c	21	18733777	18742346	19	8569	3	4	12	NA	NA	NA
Panc-AdenoCA	60e78c80-41a5-456a-9a81-9007d18fd72c	21	29639348	29647580	11	8232	2	2	7	NA	NA	NA
Panc-AdenoCA	60e78c80-41a5-456a-9a81-9007d18fd72c	21	29654882	29657240	9	2358	3	2	4	NA	NA	NA

Panc-AdenoCA	60e78c80-41a5-456a-9a81-9007d18fd72c	21	30938599	30942977	12	4378	3	5	4	NA	NA	NA
Panc-AdenoCA	60e78c80-41a5-456a-9a81-9007d18fd72c	21	34465571	34475834	31	10263	6	6	19	NA	NA	NA
Panc-AdenoCA	60e78c80-41a5-456a-9a81-9007d18fd72c	21	40217157	40218172	10	1015	3	2	5	NA	NA	NA
Panc-AdenoCA	60e78c80-41a5-456a-9a81-9007d18fd72c	21	42608632	42616287	16	7655	3	4	9	NA	NA	NA
Panc-AdenoCA	60e78c80-41a5-456a-9a81-9007d18fd72c	21	43346020	43348952	7	2932	3	1	3	NA	NA	NA
Biliary-AdenoCA	60f81dba-c623-11e3-bf01-24c6515278c0	1	31085094	31085852	7	758	NA	1	6	NA	NA	NA
Biliary-AdenoCA	60f81dba-c623-11e3-bf01-24c6515278c0	1	31253314	31254464	10	1150	2	7	1	NA	NA	NA
Biliary-AdenoCA	60f81dba-c623-11e3-bf01-24c6515278c0	1	51975350	51978957	13	3607	NA	NA	12	NA	1	NA
Biliary-AdenoCA	60f81dba-c623-11e3-bf01-24c6515278c0	1	117541303	117544142	7	2839	NA	NA	4	NA	2	1
Biliary-AdenoCA	60f81dba-c623-11e3-bf01-24c6515278c0	1	216308265	216313038	18	4773	NA	1	17	NA	NA	NA
Biliary-AdenoCA	60f81dba-c623-11e3-bf01-24c6515278c0	4	68781146	68784477	8	3331	NA	1	7	NA	NA	NA
Biliary-AdenoCA	60f81dba-c623-11e3-bf01-24c6515278c0	6	65300337	65305475	18	5138	NA	NA	18	NA	NA	NA
Biliary-AdenoCA	60f81dba-c623-11e3-bf01-24c6515278c0	6	65328685	65337996	29	9311	2	7	19	1	NA	NA
Biliary-AdenoCA	60f81dba-c623-11e3-bf01-24c6515278c0	6	65383062	65385787	7	2725	NA	NA	7	NA	NA	NA
Biliary-AdenoCA	60f81dba-c623-11e3-bf01-24c6515278c0	13	19616912	19618504	6	1592	NA	2	4	NA	NA	NA
Stomach-AdenoCA	61cad26a-cbc6-4977-83b9-dc61e49f2861	17	61178270	61188571	27	10301	3	2	21	NA	NA	1
Prost-AdenoCA	61d941f1-87df-43af-8a29-b6809c809275	12	28704693	28713750	30	9057	1	13	16	NA	NA	NA
Prost-AdenoCA	61d941f1-87df-43af-8a29-b6809c809275	20	30387479	30389681	7	2202	NA	1	6	NA	NA	NA
Prost-AdenoCA	61d941f1-87df-43af-8a29-b6809c809275	20	34970428	34977909	10	7481	1	5	4	NA	NA	NA
Prost-AdenoCA	6218f366-d5d2-4289-a95e-eeedfae7523a	8	42499928	42502880	6	2952	NA	3	3	NA	NA	NA
Prost-AdenoCA	6218f366-d5d2-4289-a95e-eeedfae7523a	8	42527545	42532781	9	5236	1	5	3	NA	NA	NA
Stomach-AdenoCA	622eaa04-b8f9-471b-98b0-7befd119f14c	18	4467182	4467644	14	462	1	6	7	NA	NA	NA
Stomach-AdenoCA	622eaa04-b8f9-471b-98b0-7befd119f14c	18	19733933	19734631	8	698	2	1	4	1	NA	NA
Lymph-BNHL	626c9788-d70c-449b-b9dc-5e3f6fc00991	2	89127489	89129067	7	1578	NA	4	1	1	1	NA
Lymph-BNHL	626c9788-d70c-449b-b9dc-5e3f6fc00991	2	89155981	89197123	149	41142	7	21	26	49	16	30
Lymph-BNHL	626c9788-d70c-449b-b9dc-5e3f6fc00991	2	89442289	89442651	13	362	NA	2	4	6	1	NA
Lymph-BNHL	626c9788-d70c-449b-b9dc-5e3f6fc00991	4	97640613	97642151	9	1538	NA	NA	NA	2	NA	7
Lymph-BNHL	626c9788-d70c-449b-b9dc-5e3f6fc00991	4	160952648	160956976	8	4328	NA	NA	1	4	1	2
Lymph-BNHL	626c9788-d70c-449b-b9dc-5e3f6fc00991	6	31549062	31549877	6	815	1	3	2	NA	NA	NA
Lymph-BNHL	626c9788-d70c-449b-b9dc-5e3f6fc00991	6	134495396	134496694	10	1298	NA	2	2	1	1	4
Lymph-BNHL	626c9788-d70c-449b-b9dc-5e3f6fc00991	8	59973234	59973526	6	292	NA	NA	NA	3	1	2
Lymph-BNHL	626c9788-d70c-449b-b9dc-5e3f6fc00991	12	113494076	113497946	6	3870	NA	NA	4	NA	1	1
Lymph-BNHL	626c9788-d70c-449b-b9dc-5e3f6fc00991	14	102285457	102287310	6	1853	NA	NA	1	4	NA	1
Lymph-BNHL	626c9788-d70c-449b-b9dc-5e3f6fc00991	14	106055516	106056305	9	789	NA	3	6	NA	NA	NA
Lymph-BNHL	626c9788-d70c-449b-b9dc-5e3f6fc00991	14	106068330	106071318	10	2988	NA	5	4	1	NA	NA
Lymph-BNHL	626c9788-d70c-449b-b9dc-5e3f6fc00991	14	106110995	106114630	28	3635	2	6	19	NA	NA	1
Lymph-BNHL	626c9788-d70c-449b-b9dc-5e3f6fc00991	14	106209666	106214932	21	5266	4	3	10	1	3	NA
Lymph-BNHL	626c9788-d70c-449b-b9dc-5e3f6fc00991	14	106238814	106240670	30	1856	3	7	18	1	NA	1
Lymph-BNHL	626c9788-d70c-449b-b9dc-5e3f6fc00991	14	106324374	106330559	100	6185	8	21	41	13	4	13
Lymph-BNHL	626c9788-d70c-449b-b9dc-5e3f6fc00991	14	106692274	106692552	7	278	NA	1	1	4	NA	1
Lymph-BNHL	626c9788-d70c-449b-b9dc-5e3f6fc00991	16	11462434	11462539	7	105	1	NA	NA	3	1	2
Lymph-BNHL	626c9788-d70c-449b-b9dc-5e3f6fc00991	16	78739234	78745215	7	5981	1	NA	NA	2	3	1
Lymph-BNHL	626c9788-d70c-449b-b9dc-5e3f6fc00991	16	85931783	85933433	8	1650	2	2	1	2	1	NA
Lymph-BNHL	626c9788-d70c-449b-b9dc-5e3f6fc00991	18	60984851	60988523	58	3672	4	6	17	16	6	9
Lymph-BNHL	626c9788-d70c-449b-b9dc-5e3f6fc00991	22	23223125	23300877	137	77752	9	23	35	43	11	16
Panc-AdenoCA	6297aa77-37a0-4f46-987b-32bd8653c0c2	7	23918053	23918220	9	167	3	2	4	NA	NA	NA
Panc-AdenoCA	6297aa77-37a0-4f46-987b-32bd8653c0c2	12	54944214	54945704	9	1490	NA	2	7	NA	NA	NA
Panc-AdenoCA	6297aa77-37a0-4f46-987b-32bd8653c0c2	14	97600219	97601704	12	1485	3	3	6	NA	NA	NA
Panc-AdenoCA	631ab9d0-5400-400c-b0bc-c3e576bcfe06	1	59495407	59498867	11	3460	2	4	4	NA	1	NA
Panc-AdenoCA	631ab9d0-5400-400c-b0bc-c3e576bcfe06	6	49638173	49638584	8	411	NA	3	5	NA	NA	NA
Panc-AdenoCA	631ab9d0-5400-400c-b0bc-c3e576bcfe06	10	37131143	37133497	6	2354	2	2	2	NA	NA	NA
Liver-HCC	632f3eb0-c623-11e3-bf01-24c6515278c0	11	60696356	60697506	9	1150	NA	4	5	NA	NA	NA
Bone-Leiomyo	63db50d6-5ef2-44d0-9906-26eae74ecf44	2	15293960	15295331	7	1371	NA	NA	7	NA	NA	NA
Bone-Leiomyo	63db50d6-5ef2-44d0-9906-26eae74ecf44	2	232280845	232285724	31	4879	NA	NA	31	NA	NA	NA
Bone-Leiomyo	63db50d6-5ef2-44d0-9906-26eae74ecf44	4	1976385	1982551	12	6166	NA	NA	12	NA	NA	NA
Bone-Leiomyo	63db50d6-5ef2-44d0-9906-26eae74ecf44	5	27392618	27392882	9	264	NA	NA	9	NA	NA	NA
Bone-Leiomyo	63db50d6-5ef2-44d0-9906-26eae74ecf44	5	41058917	41061878	8	2961	NA	NA	8	NA	NA	NA
Bone-Leiomyo	63db50d6-5ef2-44d0-9906-26eae74ecf44	5	95142794	95143560	7	766	NA	NA	7	NA	NA	NA
Bone-Leiomyo	63db50d6-5ef2-44d0-9906-26eae74ecf44	5	110134728	110142203	28	7475	1	3	24	NA	NA	NA
Bone-Leiomyo	63db50d6-5ef2-44d0-9906-26eae74ecf44	8	30791253	30796350	7	5097	1	4	2	NA	NA	NA

Bone-Leiomyo	63db50d6-5ef2-44d0-9906-26eae74ecf44	12	48787766	48791490	9	3724	NA	7	2	NA	NA	NA
Bone-Leiomyo	63db50d6-5ef2-44d0-9906-26eae74ecf44	12	52957556	52958033	10	477	2	6	2	NA	NA	NA
Bone-Leiomyo	63db50d6-5ef2-44d0-9906-26eae74ecf44	12	62543060	62550090	11	7030	1	5	5	NA	NA	NA
Bone-Leiomyo	63db50d6-5ef2-44d0-9906-26eae74ecf44	12	66289292	66290720	23	1428	NA	6	17	NA	NA	NA
Bone-Leiomyo	63db50d6-5ef2-44d0-9906-26eae74ecf44	12	69175350	69177906	9	2556	NA	5	4	NA	NA	NA
Bone-Leiomyo	63db50d6-5ef2-44d0-9906-26eae74ecf44	12	73906390	73909071	14	2681	3	5	6	NA	NA	NA
Bone-Leiomyo	63db50d6-5ef2-44d0-9906-26eae74ecf44	12	75391713	75395022	8	3309	1	6	1	NA	NA	NA
Bone-Leiomyo	63db50d6-5ef2-44d0-9906-26eae74ecf44	12	75645966	75652258	11	6292	1	4	6	NA	NA	NA
Bone-Leiomyo	63db50d6-5ef2-44d0-9906-26eae74ecf44	12	75699364	75704801	9	5437	NA	3	6	NA	NA	NA
Bone-Leiomyo	63db50d6-5ef2-44d0-9906-26eae74ecf44	12	75747403	75754483	15	7080	2	3	9	1	NA	NA
Bone-Leiomyo	63db50d6-5ef2-44d0-9906-26eae74ecf44	12	91333135	91336837	9	3702	NA	4	5	NA	NA	NA
Bone-Leiomyo	63db50d6-5ef2-44d0-9906-26eae74ecf44	12	112782959	112790534	16	7575	NA	6	9	NA	1	NA
Bone-Leiomyo	63db50d6-5ef2-44d0-9906-26eae74ecf44	19	6894868	6895354	7	486	NA	NA	6	NA	1	NA
Bone-Leiomyo	63db50d6-5ef2-44d0-9906-26eae74ecf44	Y	13216586	13221267	60	4681	NA	NA	60	NA	NA	NA
Eso-AdenoCa	6495e68e-1434-45d3-a03d-f286447dda79	6	48590028	48601473	13	11445	2	4	5	NA	NA	2
Thy-AdenoCA	64a17d5b-8f40-4ccf-8f2f-e68c5fe6b1ff	1	109559774	109560393	6	619	NA	2	4	NA	NA	NA
Liver-HCC	64b80154-c623-11e3-bf01-24c6515278c0	1	241318799	241322731	12	3932	NA	8	4	NA	NA	NA
Liver-HCC	64b80154-c623-11e3-bf01-24c6515278c0	17	22043194	22046007	10	2813	2	4	4	NA	NA	NA
Lymph-BNHL	650fe009-da01-4717-89df-9c95fafe3d7e	1	188331227	188334133	6	2906	NA	NA	NA	3	1	2
Lymph-BNHL	650fe009-da01-4717-89df-9c95fafe3d7e	2	89158105	89159779	42	1674	4	2	4	20	4	8
Lymph-BNHL	650fe009-da01-4717-89df-9c95fafe3d7e	9	30397045	30400792	7	3747	NA	NA	1	3	2	1
Lymph-BNHL	650fe009-da01-4717-89df-9c95fafe3d7e	14	106326882	106330100	33	3218	NA	6	12	8	1	6
Lymph-BNHL	650fe009-da01-4717-89df-9c95fafe3d7e	14	106725595	106733159	10	7564	2	NA	NA	4	3	1
Lymph-BNHL	650fe009-da01-4717-89df-9c95fafe3d7e	18	60984881	60988278	8	3397	NA	3	3	NA	1	1
Lymph-BNHL	650fe009-da01-4717-89df-9c95fafe3d7e	22	23223200	23231546	15	8346	1	5	2	4	1	2
Lymph-BNHL	650fe009-da01-4717-89df-9c95fafe3d7e	X	142520900	142525780	6	4880	NA	NA	NA	3	1	2
Panc-AdenoCA	654dba9f-cfe5-4121-97c7-868613cdef53	8	77127454	77127800	6	346	NA	5	1	NA	NA	NA
Panc-AdenoCA	654dba9f-cfe5-4121-97c7-868613cdef53	8	117715114	117718768	12	3654	NA	1	NA	1	8	2
Panc-AdenoCA	654dba9f-cfe5-4121-97c7-868613cdef53	11	9281360	9282847	9	1487	NA	5	4	NA	NA	NA
CNS-GBM	65723119-bdfe-46f0-b629-c171023abd71	3	173369004	173369705	9	701	NA	6	3	NA	NA	NA
CNS-GBM	65723119-bdfe-46f0-b629-c171023abd71	7	28280658	28281705	7	1047	1	4	2	NA	NA	NA
CNS-GBM	65723119-bdfe-46f0-b629-c171023abd71	7	28386470	28391920	9	5450	NA	4	4	NA	1	NA
CNS-GBM	65723119-bdfe-46f0-b629-c171023abd71	7	52156698	52158938	11	2240	4	5	2	NA	NA	NA
CNS-GBM	65723119-bdfe-46f0-b629-c171023abd71	7	53906569	53908260	7	1691	2	1	3	NA	1	NA
CNS-GBM	65723119-bdfe-46f0-b629-c171023abd71	7	54650556	54654924	8	4368	5	3	NA	NA	NA	NA
CNS-GBM	65723119-bdfe-46f0-b629-c171023abd71	7	55348023	55349530	7	1507	2	2	3	NA	NA	NA
CNS-GBM	65723119-bdfe-46f0-b629-c171023abd71	7	89717194	89721387	7	4193	NA	1	1	2	3	NA
CNS-GBM	65723119-bdfe-46f0-b629-c171023abd71	X	81207979	81212588	7	4609	1	2	2	2	NA	NA
Stomach-AdenoCA	65be412d-46c3-4cbf-9b46-b35351e53065	5	137918072	137918729	6	657	NA	3	2	NA	NA	1
Stomach-AdenoCA	65be412d-46c3-4cbf-9b46-b35351e53065	19	22086843	22089204	7	2361	NA	6	1	NA	NA	NA
Panc-AdenoCA	65d2dbc3-a163-4696-b246-47a430e66572	2	64610475	64611559	23	1084	2	8	12	NA	NA	1
Panc-AdenoCA	65d2dbc3-a163-4696-b246-47a430e66572	8	65271031	65272159	16	1128	NA	4	10	NA	NA	2
Panc-AdenoCA	65d2dbc3-a163-4696-b246-47a430e66572	8	65400908	65412365	40	11457	4	6	30	NA	NA	NA
Panc-AdenoCA	65d2dbc3-a163-4696-b246-47a430e66572	11	7813379	7813934	6	555	1	2	3	NA	NA	NA
Panc-AdenoCA	65d2dbc3-a163-4696-b246-47a430e66572	11	107556514	107560181	22	3667	2	6	14	NA	NA	NA
Panc-AdenoCA	65d2dbc3-a163-4696-b246-47a430e66572	11	107589258	107590379	9	1121	NA	5	4	NA	NA	NA
Panc-AdenoCA	65d2dbc3-a163-4696-b246-47a430e66572	X	126610295	126612217	11	1922	2	3	6	NA	NA	NA
Uterus-AdenoCA	65f9a820-f026-4a10-8bb7-1fbc3b38321e	19	28029531	28032852	6	3321	NA	2	4	NA	NA	NA
Uterus-AdenoCA	65f9a820-f026-4a10-8bb7-1fbc3b38321e	19	28037649	28039313	22	1664	4	8	10	NA	NA	NA
Panc-AdenoCA	66290cc6-1f1f-4a0b-83d1-423952da4840	17	44171162	44173794	9	2632	NA	4	5	NA	NA	NA
Panc-AdenoCA	66290cc6-1f1f-4a0b-83d1-423952da4840	18	23365345	23366826	6	1481	1	1	4	NA	NA	NA
Ovary-AdenoCA	669f0e01-28f6-4ed8-bdb5-73f84ea28f78	13	75054373	75058192	7	3819	4	3	NA	NA	NA	NA
Ovary-AdenoCA	669f0e01-28f6-4ed8-bdb5-73f84ea28f78	13	78803310	78803700	9	390	1	2	6	NA	NA	NA
Ovary-AdenoCA	669f0e01-28f6-4ed8-bdb5-73f84ea28f78	13	90735523	90736360	7	837	3	3	1	NA	NA	NA
Liver-HCC	670f971f-a525-4ef8-91ba-610cfbaeb914	13	101725537	101728041	8	2504	1	7	NA	NA	NA	NA
Lung-AdenoCA	6726c157-f688-491d-8b56-35628645df89	1	219601499	219603751	7	2252	NA	4	3	NA	NA	NA
Liver-HCC	674819dc-c622-11e3-bf01-24c6515278c0	1	97637216	97637586	6	370	NA	NA	6	NA	NA	NA
Liver-HCC	674819dc-c622-11e3-bf01-24c6515278c0	1	97656565	97657567	8	1002	NA	NA	5	2	1	NA
Liver-HCC	674819dc-c622-11e3-bf01-24c6515278c0	1	114908649	114912194	8	3545	NA	NA	8	NA	NA	NA
Liver-HCC	674819dc-c622-11e3-bf01-24c6515278c0	6	107981879	107986565	6	4686	NA	NA	NA	4	2	NA

Liver-HCC	674819dc-c622-11e3-bf01-24c6515278c0	6	108002598	108004980	15	2382	1	6	8	NA	NA	NA
Breast-AdenoCa	6764e0a2-7a78-45c1-9189-6001c6518ce4	1	1810033	1814673	20	4640	6	4	10	NA	NA	NA
Breast-AdenoCa	6764e0a2-7a78-45c1-9189-6001c6518ce4	1	1867772	1874061	16	6289	2	5	9	NA	NA	NA
Breast-AdenoCa	6764e0a2-7a78-45c1-9189-6001c6518ce4	3	89123766	89125390	9	1624	1	4	4	NA	NA	NA
Breast-AdenoCa	6764e0a2-7a78-45c1-9189-6001c6518ce4	5	760912	763976	6	3064	NA	5	1	NA	NA	NA
Breast-AdenoCa	6764e0a2-7a78-45c1-9189-6001c6518ce4	5	13738309	13745607	16	7298	NA	10	6	NA	NA	NA
Breast-AdenoCa	6764e0a2-7a78-45c1-9189-6001c6518ce4	5	15899735	15905933	9	6198	1	3	5	NA	NA	NA
Breast-AdenoCa	6764e0a2-7a78-45c1-9189-6001c6518ce4	5	21120631	21125300	22	4669	1	12	9	NA	NA	NA
Breast-AdenoCa	6764e0a2-7a78-45c1-9189-6001c6518ce4	5	21732611	21732636	7	25	1	NA	3	NA	NA	3
Breast-AdenoCa	6764e0a2-7a78-45c1-9189-6001c6518ce4	5	24770971	24773270	8	2299	NA	6	2	NA	NA	NA
Breast-AdenoCa	6764e0a2-7a78-45c1-9189-6001c6518ce4	5	25560905	25562255	7	1350	NA	5	2	NA	NA	NA
Breast-AdenoCa	6764e0a2-7a78-45c1-9189-6001c6518ce4	5	34495465	34497511	8	2046	NA	4	4	NA	NA	NA
Breast-AdenoCa	6764e0a2-7a78-45c1-9189-6001c6518ce4	5	36176209	36182622	8	6413	NA	4	4	NA	NA	NA
Breast-AdenoCa	6764e0a2-7a78-45c1-9189-6001c6518ce4	X	133671495	133678289	23	6794	2	15	6	NA	NA	NA
Bone-Leiomyo	67bb70b2-b6f8-4aa9-80ce-48829f9fec56	1	52298961	52301468	8	2507	NA	2	6	NA	NA	NA
Bone-Leiomyo	67bb70b2-b6f8-4aa9-80ce-48829f9fec56	1	61915121	61917371	10	2250	NA	2	8	NA	NA	NA
Bone-Leiomyo	67bb70b2-b6f8-4aa9-80ce-48829f9fec56	5	81491	82091	6	600	1	3	2	NA	NA	NA
Bone-Leiomyo	67bb70b2-b6f8-4aa9-80ce-48829f9fec56	5	173084	173593	9	509	1	3	5	NA	NA	NA
Bone-Leiomyo	67bb70b2-b6f8-4aa9-80ce-48829f9fec56	5	11126533	11126614	7	81	NA	NA	7	NA	NA	NA
Bone-Leiomyo	67bb70b2-b6f8-4aa9-80ce-48829f9fec56	5	18802703	18809366	31	6663	1	15	15	NA	NA	NA
Bone-Leiomyo	67bb70b2-b6f8-4aa9-80ce-48829f9fec56	5	19610765	19611688	9	923	NA	7	2	NA	NA	NA
Bone-Leiomyo	67bb70b2-b6f8-4aa9-80ce-48829f9fec56	5	20184536	20185772	11	1236	1	7	3	NA	NA	NA
Bone-Leiomyo	67bb70b2-b6f8-4aa9-80ce-48829f9fec56	5	26249524	26250586	14	1062	NA	3	11	NA	NA	NA
Bone-Leiomyo	67bb70b2-b6f8-4aa9-80ce-48829f9fec56	5	28776190	28777441	8	1251	1	3	4	NA	NA	NA
Bone-Leiomyo	67bb70b2-b6f8-4aa9-80ce-48829f9fec56	12	54267537	54268323	6	786	NA	2	4	NA	NA	NA
Bone-Leiomyo	67bb70b2-b6f8-4aa9-80ce-48829f9fec56	12	55350275	55355106	30	4831	NA	5	24	NA	NA	1
Bone-Leiomyo	67bb70b2-b6f8-4aa9-80ce-48829f9fec56	12	57902562	57905200	8	2638	NA	2	6	NA	NA	NA
Bone-Leiomyo	67bb70b2-b6f8-4aa9-80ce-48829f9fec56	12	61919609	61923486	9	3877	4	1	4	NA	NA	NA
Bone-Leiomyo	67bb70b2-b6f8-4aa9-80ce-48829f9fec56	12	62557500	62561160	10	3660	NA	3	7	NA	NA	NA
Bone-Leiomyo	67bb70b2-b6f8-4aa9-80ce-48829f9fec56	12	62599160	62603766	7	4606	1	4	2	NA	NA	NA
Bone-Leiomyo	67bb70b2-b6f8-4aa9-80ce-48829f9fec56	12	66475866	66486973	31	11107	1	18	12	NA	NA	NA
Bone-Leiomyo	67bb70b2-b6f8-4aa9-80ce-48829f9fec56	12	67124469	67128456	12	3987	1	8	3	NA	NA	NA
Bone-Leiomyo	67bb70b2-b6f8-4aa9-80ce-48829f9fec56	12	67719446	67719941	7	495	NA	3	4	NA	NA	NA
Bone-Leiomyo	67bb70b2-b6f8-4aa9-80ce-48829f9fec56	12	68385477	68386444	10	967	NA	5	5	NA	NA	NA
Bone-Leiomyo	67bb70b2-b6f8-4aa9-80ce-48829f9fec56	12	68449081	68454060	9	4979	1	1	7	NA	NA	NA
Bone-Leiomyo	67bb70b2-b6f8-4aa9-80ce-48829f9fec56	12	68975949	68977923	9	1974	2	5	2	NA	NA	NA
Bone-Leiomyo	67bb70b2-b6f8-4aa9-80ce-48829f9fec56	12	69201146	69202860	11	1714	NA	3	8	NA	NA	NA
Bone-Leiomyo	67bb70b2-b6f8-4aa9-80ce-48829f9fec56	12	69482161	69484737	7	2576	1	NA	6	NA	NA	NA
Bone-Leiomyo	67bb70b2-b6f8-4aa9-80ce-48829f9fec56	12	69871889	69879280	10	7391	NA	6	4	NA	NA	NA
Bone-Leiomyo	67bb70b2-b6f8-4aa9-80ce-48829f9fec56	12	70262983	70266359	17	3376	1	4	12	NA	NA	NA
Bone-Leiomyo	67bb70b2-b6f8-4aa9-80ce-48829f9fec56	12	72231969	72237957	13	5988	1	7	5	NA	NA	NA
Bone-Leiomyo	67bb70b2-b6f8-4aa9-80ce-48829f9fec56	12	72651189	72651676	7	487	NA	1	6	NA	NA	NA
Bone-Leiomyo	67bb70b2-b6f8-4aa9-80ce-48829f9fec56	12	73859506	73862467	18	2961	4	4	10	NA	NA	NA
Bone-Leiomyo	67bb70b2-b6f8-4aa9-80ce-48829f9fec56	12	74907969	74908478	8	509	1	2	5	NA	NA	NA
Bone-Leiomyo	67bb70b2-b6f8-4aa9-80ce-48829f9fec56	12	86730037	86733004	25	2967	3	5	17	NA	NA	NA
Bone-Leiomyo	67bb70b2-b6f8-4aa9-80ce-48829f9fec56	12	88501729	88505868	9	4139	1	2	6	NA	NA	NA
Bone-Leiomyo	67bb70b2-b6f8-4aa9-80ce-48829f9fec56	12	88532056	88538404	8	6348	1	5	2	NA	NA	NA
Bone-Leiomyo	67bb70b2-b6f8-4aa9-80ce-48829f9fec56	12	94452713	94456504	9	3791	1	5	3	NA	NA	NA
Bone-Leiomyo	67bb70b2-b6f8-4aa9-80ce-48829f9fec56	12	101423981	101427944	16	3963	2	7	7	NA	NA	NA
Bone-Leiomyo	67bb70b2-b6f8-4aa9-80ce-48829f9fec56	12	101589618	101592011	13	2393	1	6	6	NA	NA	NA
Bone-Leiomyo	67bb70b2-b6f8-4aa9-80ce-48829f9fec56	12	101660401	101665487	7	5086	NA	3	4	NA	NA	NA
Bone-Leiomyo	67bb70b2-b6f8-4aa9-80ce-48829f9fec56	12	101951791	101955654	12	3863	NA	4	8	NA	NA	NA
Bone-Leiomyo	67bb70b2-b6f8-4aa9-80ce-48829f9fec56	12	102295379	102296765	7	1386	NA	4	3	NA	NA	NA
Bone-Leiomyo	67bb70b2-b6f8-4aa9-80ce-48829f9fec56	12	105092632	105098950	9	6318	1	1	7	NA	NA	NA
Bone-Leiomyo	67bb70b2-b6f8-4aa9-80ce-48829f9fec56	12	105153294	105154265	9	971	NA	1	8	NA	NA	NA
Bone-Leiomyo	67bb70b2-b6f8-4aa9-80ce-48829f9fec56	12	125712353	125713859	8	1506	NA	3	5	NA	NA	NA
Bone-Leiomyo	67bb70b2-b6f8-4aa9-80ce-48829f9fec56	12	127064130	127065639	9	1509	1	5	3	NA	NA	NA
Bone-Leiomyo	67bb70b2-b6f8-4aa9-80ce-48829f9fec56	12	128128689	128132113	7	3424	1	NA	6	NA	NA	NA
Bone-Leiomyo	67bb70b2-b6f8-4aa9-80ce-48829f9fec56	14	95960828	95961341	8	513	NA	3	5	NA	NA	NA
Bone-Leiomyo	67bb70b2-b6f8-4aa9-80ce-48829f9fec56	14	98386094	98390194	7	4100	1	5	1	NA	NA	NA

Bone-Leiomyo	67bb70b2-b6f8-4aa9-80ce-48829f9fec56	14	98748279	98751461	7	3182	NA	2	5	NA	NA	NA
Bone-Leiomyo	67bb70b2-b6f8-4aa9-80ce-48829f9fec56	15	40688419	40688816	10	397	NA	NA	10	NA	NA	NA
Ovary-AdenoCA	6821446e-8e76-46fe-b0b9-47feb46d2585	19	41330113	41334552	17	4439	NA	13	4	NA	NA	NA
Stomach-AdenoCA	68509ede-3dcf-4a6e-9af0-4a9bb4dfa567	3	88738653	88742533	6	3880	NA	NA	NA	4	2	NA
Stomach-AdenoCA	68509ede-3dcf-4a6e-9af0-4a9bb4dfa567	8	47696313	47699130	6	2817	NA	NA	1	NA	5	NA
Panc-AdenoCA	6867811f-ac89-47da-b5dc-1270033c36e7	1	2353486	2354982	6	1496	NA	3	3	NA	NA	NA
Panc-AdenoCA	6867811f-ac89-47da-b5dc-1270033c36e7	3	87570029	87572868	8	2839	1	4	3	NA	NA	NA
Panc-AdenoCA	6867811f-ac89-47da-b5dc-1270033c36e7	12	4538276	4541851	6	3575	NA	2	4	NA	NA	NA
Panc-AdenoCA	6867811f-ac89-47da-b5dc-1270033c36e7	20	2911859	2913206	7	1347	2	2	3	NA	NA	NA
Panc-AdenoCA	6867811f-ac89-47da-b5dc-1270033c36e7	21	20137823	20139824	10	2001	NA	3	7	NA	NA	NA
Panc-AdenoCA	6867811f-ac89-47da-b5dc-1270033c36e7	21	31480476	31481508	10	1032	1	4	5	NA	NA	NA
Lymph-BNHL	687baf0b-aed9-4ab2-adb6-556a1d177a24	2	89159095	89160139	12	1044	NA	2	3	2	3	2
Lymph-BNHL	687baf0b-aed9-4ab2-adb6-556a1d177a24	11	2290198	2291956	7	1758	NA	NA	6	NA	1	NA
Lymph-BNHL	687baf0b-aed9-4ab2-adb6-556a1d177a24	14	106324853	106330024	71	5171	2	15	24	14	7	9
Lymph-BNHL	687baf0b-aed9-4ab2-adb6-556a1d177a24	14	107034934	107035121	11	187	2	1	6	1	NA	1
Panc-AdenoCA	68ba1105-569a-4209-9b45-0e2d64931806	8	13641415	13642426	6	1011	1	3	2	NA	NA	NA
Lung-AdenoCA	68c2a355-862c-4657-b296-5776ed8447b0	6	13877739	13878776	11	1037	NA	7	4	NA	NA	NA
Lung-AdenoCA	68c2a355-862c-4657-b296-5776ed8447b0	6	28264604	28267292	18	2688	1	7	10	NA	NA	NA
Lung-AdenoCA	68c2a355-862c-4657-b296-5776ed8447b0	17	8998407	8998855	8	448	NA	NA	7	NA	1	NA
Lung-AdenoCA	68c2a355-862c-4657-b296-5776ed8447b0	17	16943712	16944536	12	824	1	5	5	1	NA	NA
Lung-AdenoCA	68c2a355-862c-4657-b296-5776ed8447b0	20	34695699	34696073	6	374	NA	NA	NA	4	2	NA
Liver-HCC	690e481c-c623-11e3-bf01-24c6515278c0	9	25502003	25502320	6	317	4	NA	2	NA	NA	NA
Eso-AdenoCa	69c3c88c-d51e-4e03-b96b-ec4f1a0faa2d	13	34706324	34709898	8	3574	NA	8	NA	NA	NA	NA
Eso-AdenoCa	69c3c88c-d51e-4e03-b96b-ec4f1a0faa2d	13	37510342	37513944	8	3602	4	1	3	NA	NA	NA
Eso-AdenoCa	69c3c88c-d51e-4e03-b96b-ec4f1a0faa2d	13	53707727	53711733	8	4006	4	3	1	NA	NA	NA
Eso-AdenoCa	69c3c88c-d51e-4e03-b96b-ec4f1a0faa2d	13	53716355	53724141	10	7786	3	NA	6	NA	NA	1
Stomach-AdenoCA	6a7cdaf6-211e-4eb8-a70e-1153d69c45e4	3	43475456	43475726	6	270	2	3	1	NA	NA	NA
Stomach-AdenoCA	6a7cdaf6-211e-4eb8-a70e-1153d69c45e4	6	39515142	39516243	8	1101	1	4	3	NA	NA	NA
Stomach-AdenoCA	6a7cdaf6-211e-4eb8-a70e-1153d69c45e4	7	25737300	25739625	6	2325	NA	3	3	NA	NA	NA
Stomach-AdenoCA	6a7cdaf6-211e-4eb8-a70e-1153d69c45e4	10	52008926	52009829	6	903	NA	5	1	NA	NA	NA
Panc-AdenoCA	6ad44218-d34e-4126-bf56-1be2140cd3fb	18	2374464	2375369	7	905	NA	4	3	NA	NA	NA
Panc-AdenoCA	6ad44218-d34e-4126-bf56-1be2140cd3fb	18	9190888	9191912	8	1024	NA	2	6	NA	NA	NA
Panc-AdenoCA	6ad44218-d34e-4126-bf56-1be2140cd3fb	19	28594960	28603853	13	8893	NA	4	9	NA	NA	NA
Panc-AdenoCA	6ad44218-d34e-4126-bf56-1be2140cd3fb	19	33725377	33725889	7	512	NA	2	5	NA	NA	NA
Panc-AdenoCA	6ad44218-d34e-4126-bf56-1be2140cd3fb	19	46741806	46745291	10	3485	1	1	6	1	1	NA
Panc-AdenoCA	6b20f942-7b89-497c-8a2e-ccce74d20ca9	16	80614795	80616255	11	1460	3	2	6	NA	NA	NA
Panc-AdenoCA	6b20f942-7b89-497c-8a2e-ccce74d20ca9	19	40468723	40472628	6	3905	1	3	2	NA	NA	NA
Panc-AdenoCA	6b20f942-7b89-497c-8a2e-ccce74d20ca9	22	18324397	18324532	6	135	NA	NA	6	NA	NA	NA
Panc-AdenoCA	6b20f942-7b89-497c-8a2e-ccce74d20ca9	22	42650910	42652022	24	1112	NA	NA	24	NA	NA	NA
Liver-HCC	6b25a78f-20dc-4ccc-b4c0-f9885d16725d	5	42194892	42196021	13	1129	1	6	6	NA	NA	NA
Liver-HCC	6b50391f-d890-4ca0-b942-beab0f8bf1c9	6	82945666	82945811	6	145	1	1	3	1	NA	NA
Head-SCC	6bbd66dd-efee-4d6e-b02f-f88e98526b82	2	164657660	164658418	9	758	NA	6	3	NA	NA	NA
Head-SCC	6bbd66dd-efee-4d6e-b02f-f88e98526b82	Y	6788147	6790977	8	2830	NA	3	5	NA	NA	NA
Kidney-RCC	6bd4bca3-e568-4d1b-8b42-401fdf13d751	3	70742795	70744353	20	1558	4	6	10	NA	NA	NA
Kidney-RCC	6bd4bca3-e568-4d1b-8b42-401fdf13d751	4	161911302	161915565	6	4263	NA	NA	1	3	NA	2
Kidney-RCC	6bd4bca3-e568-4d1b-8b42-401fdf13d751	8	56812371	56814408	7	2037	2	1	4	NA	NA	NA
Panc-AdenoCA	6bdf00f6-670f-466e-87fb-e853e41f000e	3	146951246	146955724	16	4478	3	4	9	NA	NA	NA
Liver-HCC	6c306cbe-c623-11e3-bf01-24c6515278c0	5	19672442	19675373	8	2931	NA	NA	1	4	NA	3
Ovary-AdenoCA	6c31d730-1734-44ac-92b3-760981528723	4	189284323	189286861	9	2538	NA	4	5	NA	NA	NA
Ovary-AdenoCA	6c31d730-1734-44ac-92b3-760981528723	19	13168850	13170241	12	1391	4	7	1	NA	NA	NA
Ovary-AdenoCA	6c31d730-1734-44ac-92b3-760981528723	19	18053943	18058005	9	4062	NA	7	2	NA	NA	NA
Ovary-AdenoCA	6c31d730-1734-44ac-92b3-760981528723	20	6121292	6137485	45	16193	6	20	17	NA	2	NA
Breast-AdenoCa	6c65cb6d-8c50-4c7c-bc84-eeebe09fbe01	1	62455200	62457340	10	2140	NA	1	2	2	5	NA
Breast-AdenoCa	6c65cb6d-8c50-4c7c-bc84-eeebe09fbe01	1	183237022	183264526	64	27504	NA	1	63	NA	NA	NA
Breast-AdenoCa	6c65cb6d-8c50-4c7c-bc84-eeebe09fbe01	1	186585904	186587263	14	1359	NA	1	13	NA	NA	NA
Breast-AdenoCa	6c65cb6d-8c50-4c7c-bc84-eeebe09fbe01	1	192506935	192521384	19	14449	NA	1	18	NA	NA	NA
Breast-AdenoCa	6c65cb6d-8c50-4c7c-bc84-eeebe09fbe01	1	209719376	209727217	15	7841	NA	NA	15	NA	NA	NA
Breast-AdenoCa	6c8e9197-fd16-4fed-bfda-e349cab26314	1	156408172	156412259	7	4087	1	2	4	NA	NA	NA
Breast-AdenoCa	6c8e9197-fd16-4fed-bfda-e349cab26314	6	84548412	84571054	24	22642	2	1	21	NA	NA	NA
Breast-AdenoCa	6c8e9197-fd16-4fed-bfda-e349cab26314	8	68751775	68751989	9	214	NA	NA	9	NA	NA	NA

Breast-AdenoCa	6c8e9197-fd16-4fed-bfda-e349cab26314	8	108159791	108161672	10	1881	NA	4	6	NA	NA	NA
Breast-AdenoCa	6c8e9197-fd16-4fed-bfda-e349cab26314	8	108324806	108325102	7	296	NA	5	2	NA	NA	NA
Breast-AdenoCa	6c8e9197-fd16-4fed-bfda-e349cab26314	8	113789079	113794596	14	5517	1	2	11	NA	NA	NA
Breast-AdenoCa	6c8e9197-fd16-4fed-bfda-e349cab26314	8	114134944	114138379	13	3435	2	3	8	NA	NA	NA
Breast-AdenoCa	6c8e9197-fd16-4fed-bfda-e349cab26314	8	114943452	114944302	8	850	NA	6	2	NA	NA	NA
Breast-AdenoCa	6c8e9197-fd16-4fed-bfda-e349cab26314	9	28897179	28897185	6	6	1	1	1	1	NA	2
Breast-AdenoCa	6c8e9197-fd16-4fed-bfda-e349cab26314	9	35096976	35101259	7	4283	1	NA	6	NA	NA	NA
Breast-AdenoCa	6c8e9197-fd16-4fed-bfda-e349cab26314	9	35119328	35167522	52	48194	4	6	40	NA	NA	2
Breast-AdenoCa	6c8e9197-fd16-4fed-bfda-e349cab26314	X	33565318	33570174	7	4856	NA	NA	6	NA	NA	1
Ovary-AdenoCA	6ce42746-88aa-485f-a1c4-1759aad95914	10	33024929	33029792	7	4863	2	2	2	NA	NA	1
Ovary-AdenoCA	6ce42746-88aa-485f-a1c4-1759aad95914	11	50734258	50739628	9	5370	4	3	2	NA	NA	NA
Ovary-AdenoCA	6ce42746-88aa-485f-a1c4-1759aad95914	19	40035815	40040483	6	4668	1	NA	5	NA	NA	NA
Lymph-CLL	6cfce053-bfd6-4ca0-b74b-b2e4549e4f1f	2	89159564	89161387	22	1823	1	4	4	9	4	NA
Lymph-CLL	6cfce053-bfd6-4ca0-b74b-b2e4549e4f1f	2	89185049	89185451	12	402	NA	1	3	6	NA	2
Lymph-CLL	6cfce053-bfd6-4ca0-b74b-b2e4549e4f1f	14	106326566	106330421	23	3855	1	4	4	10	2	2
Lymph-CLL	6cfce053-bfd6-4ca0-b74b-b2e4549e4f1f	14	106829866	106830539	9	673	NA	2	NA	4	2	1
Prost-AdenoCA	6d936ef9-b5df-44d3-831f-528bf8ddc131	X	66195531	66197340	6	1809	NA	3	2	NA	1	NA
Prost-AdenoCA	6d936ef9-b5df-44d3-831f-528bf8ddc131	X	83966025	83967682	7	1657	1	NA	3	1	1	1
Stomach-AdenoCA	6dbac113-d4cf-4db5-97c9-50fa400bb47e	7	55481535	55493556	15	12021	1	2	11	NA	1	NA
Stomach-AdenoCA	6dbac113-d4cf-4db5-97c9-50fa400bb47e	13	59407890	59412340	7	4450	NA	NA	NA	1	4	2
Stomach-AdenoCA	6dbac113-d4cf-4db5-97c9-50fa400bb47e	17	33158002	33158678	6	676	1	5	NA	NA	NA	NA
Breast-LobularCa	6dbc5c31-043b-40e9-bf1a-e7b4ec351b4d	3	40426505	40427582	10	1077	1	8	NA	NA	NA	1
Breast-LobularCa	6dbc5c31-043b-40e9-bf1a-e7b4ec351b4d	17	62600071	62608193	14	8122	3	7	4	NA	NA	NA
CNS-GBM	6de41ac1-229b-40b9-a494-5588c284351d	6	115589785	115590362	7	577	NA	5	2	NA	NA	NA
CNS-GBM	6de41ac1-229b-40b9-a494-5588c284351d	19	43933334	43937605	6	4271	1	4	1	NA	NA	NA
Liver-HCC	6e6ad1a2-f1eb-44d1-9852-f5752afcf5eb	19	45143158	45144777	7	1619	NA	NA	7	NA	NA	NA
Liver-HCC	6e6ad1a2-f1eb-44d1-9852-f5752afcf5eb	19	51309747	51312194	8	2447	NA	1	7	NA	NA	NA
Liver-HCC	6e6ad1a2-f1eb-44d1-9852-f5752afcf5eb	19	51318870	51323027	8	4157	1	NA	7	NA	NA	NA
Liver-HCC	6e6ad1a2-f1eb-44d1-9852-f5752afcf5eb	19	58255194	58265306	13	10112	1	6	6	NA	NA	NA
Panc-Endocrine	6e776415-ddf4-4060-953f-08ef22441234	5	58564378	58567573	15	3195	1	8	6	NA	NA	NA
Panc-Endocrine	6e776415-ddf4-4060-953f-08ef22441234	5	58789835	58794762	11	4927	NA	4	4	3	NA	NA
Panc-Endocrine	6e776415-ddf4-4060-953f-08ef22441234	11	61242110	61243672	10	1562	NA	5	5	NA	NA	NA
Panc-Endocrine	6e776415-ddf4-4060-953f-08ef22441234	12	114163035	114163265	6	230	1	2	2	1	NA	NA
Panc-Endocrine	6e776415-ddf4-4060-953f-08ef22441234	18	18902439	18904198	13	1759	2	7	4	NA	NA	NA
Breast-AdenoCa	6e839eaf-1dbb-43f5-8846-c980e05540c7	5	56807677	56812850	18	5173	NA	1	17	NA	NA	NA
Breast-AdenoCa	6e839eaf-1dbb-43f5-8846-c980e05540c7	5	58796205	58797396	13	1191	NA	6	7	NA	NA	NA
Breast-AdenoCa	6e839eaf-1dbb-43f5-8846-c980e05540c7	6	70814337	70815481	7	1144	2	5	NA	NA	NA	NA
Breast-AdenoCa	6e839eaf-1dbb-43f5-8846-c980e05540c7	14	31820204	31821127	8	923	1	3	4	NA	NA	NA
Breast-AdenoCa	6e839eaf-1dbb-43f5-8846-c980e05540c7	15	100523947	100529247	11	5300	NA	9	2	NA	NA	NA
Breast-AdenoCa	6e839eaf-1dbb-43f5-8846-c980e05540c7	15	100538989	100542305	10	3316	NA	7	3	NA	NA	NA
Breast-AdenoCa	6e839eaf-1dbb-43f5-8846-c980e05540c7	17	47591265	47595550	6	4285	1	3	2	NA	NA	NA
Breast-AdenoCa	6e839eaf-1dbb-43f5-8846-c980e05540c7	18	22115516	22118272	7	2756	1	3	3	NA	NA	NA
Liver-HCC	6ec4456e-c622-11e3-bf01-24c6515278c0	21	17026108	17027567	13	1459	3	4	6	NA	NA	NA
Biliary-AdenoCA	6f395380-c623-11e3-bf01-24c6515278c0	9	24263285	24264671	7	1386	NA	4	3	NA	NA	NA
Biliary-AdenoCA	6f395380-c623-11e3-bf01-24c6515278c0	14	24895091	24895140	6	49	NA	NA	NA	2	4	NA
Ovary-AdenoCA	6f981023-4269-4e8e-a4ab-2c92bb27273c	20	62822554	62825445	6	2891	2	3	1	NA	NA	NA
Panc-AdenoCA	6fc0cb07-9713-4355-8543-f85d1c5505c2	4	55403932	55415209	21	11277	2	10	8	NA	1	NA
Panc-AdenoCA	6fc0cb07-9713-4355-8543-f85d1c5505c2	8	83159324	83160458	8	1134	5	NA	3	NA	NA	NA
Panc-AdenoCA	6fc0cb07-9713-4355-8543-f85d1c5505c2	8	87631503	87632337	9	834	1	2	6	NA	NA	NA
Panc-AdenoCA	6fc0cb07-9713-4355-8543-f85d1c5505c2	8	146229307	146230888	9	1581	4	1	4	NA	NA	NA
Panc-AdenoCA	6fc0cb07-9713-4355-8543-f85d1c5505c2	12	22449187	22452762	7	3575	NA	3	4	NA	NA	NA
Panc-AdenoCA	6fc0cb07-9713-4355-8543-f85d1c5505c2	18	329762	335526	19	5764	4	6	9	NA	NA	NA
Panc-AdenoCA	6fc0cb07-9713-4355-8543-f85d1c5505c2	18	10289967	10293219	13	3252	1	4	8	NA	NA	NA
Panc-AdenoCA	6fc0cb07-9713-4355-8543-f85d1c5505c2	18	10982438	10982880	9	442	3	1	5	NA	NA	NA
Liver-HCC	70422e6d-cb1f-4284-8be9-1d4517ffad60	17	12713372	12716443	7	3071	NA	1	4	NA	1	1
Eso-AdenoCa	708e21ac-f529-4461-bc4f-d3a194a92b80	18	18647446	18648375	8	929	NA	5	3	NA	NA	NA
Panc-AdenoCA	70dd8453-11d3-4105-a8d0-6a755c52495d	2	225998943	225999211	10	268	2	1	7	NA	NA	NA
Panc-AdenoCA	70dd8453-11d3-4105-a8d0-6a755c52495d	4	137867005	137870281	13	3276	NA	6	7	NA	NA	NA
Panc-AdenoCA	70dd8453-11d3-4105-a8d0-6a755c52495d	13	76238094	76239830	6	1736	NA	NA	5	NA	NA	1
Panc-AdenoCA	70dd8453-11d3-4105-a8d0-6a755c52495d	X	94138831	94139598	6	767	NA	3	3	NA	NA	NA

Stomach-AdenoCA	7116cc90-f465-4e7e-aa27-48a83ed258e9	17	19771445	19775336	6	3891	1	4	NA	NA	1	NA
Stomach-AdenoCA	7116cc90-f465-4e7e-aa27-48a83ed258e9	21	19115745	19116177	6	432	NA	5	1	NA	NA	NA
Head-SCC	7158c9fd-a633-4fef-aa23-bd285f2a87a8	12	56504766	56506247	6	1481	1	3	2	NA	NA	NA
Lymph-BNHL	71982a1b-52a4-440f-bd5d-62a0e6cc7fd9	6	91004705	91005806	12	1101	2	4	1	2	2	1
Lymph-BNHL	71982a1b-52a4-440f-bd5d-62a0e6cc7fd9	8	128748551	128763492	42	14941	6	15	13	4	3	1
Lymph-BNHL	71982a1b-52a4-440f-bd5d-62a0e6cc7fd9	12	122458647	122462101	12	3454	1	3	4	2	NA	2
Lymph-BNHL	71982a1b-52a4-440f-bd5d-62a0e6cc7fd9	13	75979979	75984337	8	4358	NA	NA	3	3	NA	2
Lymph-BNHL	71982a1b-52a4-440f-bd5d-62a0e6cc7fd9	14	106068001	106070048	6	2047	NA	1	5	NA	NA	NA
Lymph-BNHL	71982a1b-52a4-440f-bd5d-62a0e6cc7fd9	14	106326619	106358414	77	31795	7	17	18	12	6	17
Lymph-BNHL	71982a1b-52a4-440f-bd5d-62a0e6cc7fd9	14	106877741	106878788	18	1047	NA	1	3	7	4	3
Lymph-BNHL	71982a1b-52a4-440f-bd5d-62a0e6cc7fd9	16	11348263	11349626	8	1363	2	3	1	1	1	NA
Lymph-BNHL	71982a1b-52a4-440f-bd5d-62a0e6cc7fd9	22	22378922	22380602	6	1680	1	NA	NA	2	1	2
Lymph-BNHL	71982a1b-52a4-440f-bd5d-62a0e6cc7fd9	22	23263800	23264775	50	975	5	14	14	8	4	5
Panc-AdenoCA	71ef8379-03a0-4b81-92c9-1ba666fe4d7a	2	88492252	88492404	8	152	NA	2	6	NA	NA	NA
Panc-AdenoCA	71ef8379-03a0-4b81-92c9-1ba666fe4d7a	18	3825539	3827924	7	2385	1	2	4	NA	NA	NA
Panc-AdenoCA	71ef8379-03a0-4b81-92c9-1ba666fe4d7a	18	10510871	10514614	7	3743	1	1	5	NA	NA	NA
Panc-AdenoCA	71ef8379-03a0-4b81-92c9-1ba666fe4d7a	18	10789265	10794068	8	4803	3	2	3	NA	NA	NA
Panc-AdenoCA	71ef8379-03a0-4b81-92c9-1ba666fe4d7a	18	20984146	20988001	7	3855	NA	1	6	NA	NA	NA
Panc-AdenoCA	71ef8379-03a0-4b81-92c9-1ba666fe4d7a	18	24744349	24744958	9	609	3	2	4	NA	NA	NA
Panc-AdenoCA	71ef8379-03a0-4b81-92c9-1ba666fe4d7a	19	49941523	49942220	12	697	2	6	4	NA	NA	NA
Panc-AdenoCA	71ef8379-03a0-4b81-92c9-1ba666fe4d7a	19	52346948	52347243	9	295	2	1	6	NA	NA	NA
Panc-AdenoCA	71ef8379-03a0-4b81-92c9-1ba666fe4d7a	19	52365045	52368306	10	3261	2	1	7	NA	NA	NA
Breast-AdenoCa	71fda5a9-7049-406f-9348-2c24f7ddf5f4	1	176545232	176547241	7	2009	3	NA	4	NA	NA	NA
Breast-AdenoCa	71fda5a9-7049-406f-9348-2c24f7ddf5f4	12	86960602	86961914	9	1312	3	2	4	NA	NA	NA
Liver-HCC	7260f57c-c623-11e3-bf01-24c6515278c0	12	109309920	109311154	8	1234	1	3	4	NA	NA	NA
Panc-AdenoCA	7266ec7b-4824-4b88-910e-e9bfb848d55f	18	23036647	23040751	9	4104	NA	1	8	NA	NA	NA
Panc-AdenoCA	72a5e569-99d4-47bc-be1b-6296a5025080	18	20375607	20385726	24	10119	2	16	6	NA	NA	NA
Bone-Leiomyo	72f0a49a-aec8-47e5-846a-956c4da1507c	2	133938970	133940107	6	1137	1	NA	5	NA	NA	NA
Bone-Leiomyo	72f0a49a-aec8-47e5-846a-956c4da1507c	2	242339599	242342388	7	2789	1	1	5	NA	NA	NA
Bone-Leiomyo	72f0a49a-aec8-47e5-846a-956c4da1507c	3	1842807	1843911	14	1104	3	5	4	NA	1	1
Bone-Leiomyo	72f0a49a-aec8-47e5-846a-956c4da1507c	4	187111892	187113423	13	1531	1	1	11	NA	NA	NA
Bone-Leiomyo	72f0a49a-aec8-47e5-846a-956c4da1507c	5	101819840	101820264	6	424	1	3	2	NA	NA	NA
Bone-Leiomyo	72f0a49a-aec8-47e5-846a-956c4da1507c	5	109176037	109177538	11	1501	4	2	5	NA	NA	NA
Bone-Leiomyo	72f0a49a-aec8-47e5-846a-956c4da1507c	5	135041005	135043628	14	2623	2	1	11	NA	NA	NA
Bone-Leiomyo	72f0a49a-aec8-47e5-846a-956c4da1507c	6	161499193	161503349	11	4156	3	3	5	NA	NA	NA
Bone-Leiomyo	72f0a49a-aec8-47e5-846a-956c4da1507c	7	22117307	22117755	9	448	1	2	6	NA	NA	NA
Bone-Leiomyo	72f0a49a-aec8-47e5-846a-956c4da1507c	8	67971695	67972147	8	452	NA	NA	8	NA	NA	NA
Bone-Leiomyo	72f0a49a-aec8-47e5-846a-956c4da1507c	8	100594950	100602460	10	7510	NA	5	5	NA	NA	NA
Bone-Leiomyo	72f0a49a-aec8-47e5-846a-956c4da1507c	9	130666118	130671154	7	5036	2	2	3	NA	NA	NA
Bone-Leiomyo	72f0a49a-aec8-47e5-846a-956c4da1507c	9	130768698	130778931	18	10233	3	5	10	NA	NA	NA
Bone-Leiomyo	72f0a49a-aec8-47e5-846a-956c4da1507c	9	131945073	131950838	15	5765	2	6	7	NA	NA	NA
Bone-Leiomyo	72f0a49a-aec8-47e5-846a-956c4da1507c	9	135941010	135941717	10	707	NA	5	5	NA	NA	NA
Bone-Leiomyo	72f0a49a-aec8-47e5-846a-956c4da1507c	10	21990540	21994022	7	3482	3	1	3	NA	NA	NA
Bone-Leiomyo	72f0a49a-aec8-47e5-846a-956c4da1507c	11	36442260	36442859	7	599	1	4	2	NA	NA	NA
Bone-Leiomyo	72f0a49a-aec8-47e5-846a-956c4da1507c	11	43052852	43054732	10	1880	NA	3	7	NA	NA	NA
Bone-Leiomyo	72f0a49a-aec8-47e5-846a-956c4da1507c	11	90284100	90288531	14	4431	NA	1	13	NA	NA	NA
Bone-Leiomyo	72f0a49a-aec8-47e5-846a-956c4da1507c	11	98137746	98140906	7	3160	NA	NA	6	NA	1	NA
Bone-Leiomyo	72f0a49a-aec8-47e5-846a-956c4da1507c	11	99529253	99531894	9	2641	1	5	3	NA	NA	NA
Bone-Leiomyo	72f0a49a-aec8-47e5-846a-956c4da1507c	11	102546754	102547498	7	744	2	1	3	NA	NA	1
Bone-Leiomyo	72f0a49a-aec8-47e5-846a-956c4da1507c	12	58419782	58421548	6	1766	NA	3	3	NA	NA	NA
Bone-Leiomyo	72f0a49a-aec8-47e5-846a-956c4da1507c	12	66440610	66445359	11	4749	2	2	6	NA	NA	1
Bone-Leiomyo	72f0a49a-aec8-47e5-846a-956c4da1507c	12	68824537	68825309	7	772	1	3	3	NA	NA	NA
Bone-Leiomyo	72f0a49a-aec8-47e5-846a-956c4da1507c	12	69799415	69802586	8	3171	1	2	5	NA	NA	NA
Bone-Leiomyo	72f0a49a-aec8-47e5-846a-956c4da1507c	12	69865382	69869838	7	4456	1	3	3	NA	NA	NA
Bone-Leiomyo	72f0a49a-aec8-47e5-846a-956c4da1507c	12	71768670	71778236	30	9566	NA	2	28	NA	NA	NA
Bone-Leiomyo	72f0a49a-aec8-47e5-846a-956c4da1507c	12	73948981	73953866	21	4885	1	2	18	NA	NA	NA
Bone-Leiomyo	72f0a49a-aec8-47e5-846a-956c4da1507c	12	88489516	88494779	7	5263	1	NA	6	NA	NA	NA
Bone-Leiomyo	72f0a49a-aec8-47e5-846a-956c4da1507c	12	88507950	88509392	17	1442	NA	NA	17	NA	NA	NA
Bone-Leiomyo	72f0a49a-aec8-47e5-846a-956c4da1507c	14	53033663	53035417	7	1754	3	3	1	NA	NA	NA
Bone-Leiomyo	72f0a49a-aec8-47e5-846a-956c4da1507c	17	6159360	6169060	11	9700	NA	4	6	NA	1	NA

Bone-Leiomyo	72f0a49a-aec8-47e5-846a-956c4da1507c	17	7639889	7642475	7	2586	1	2	4	NA	NA	NA
Bone-Leiomyo	72f0a49a-aec8-47e5-846a-956c4da1507c	17	39837174	39840674	7	3500	1	1	5	NA	NA	NA
Bone-Leiomyo	72f0a49a-aec8-47e5-846a-956c4da1507c	17	41112505	41113097	8	592	2	4	2	NA	NA	NA
Bone-Leiomyo	72f0a49a-aec8-47e5-846a-956c4da1507c	17	50949964	50951018	15	1054	NA	NA	15	NA	NA	NA
Bone-Leiomyo	72f0a49a-aec8-47e5-846a-956c4da1507c	17	66897443	66899367	7	1924	4	1	2	NA	NA	NA
Bone-Leiomyo	72f0a49a-aec8-47e5-846a-956c4da1507c	18	4921845	4926510	8	4665	1	3	4	NA	NA	NA
Bone-Leiomyo	72f0a49a-aec8-47e5-846a-956c4da1507c	18	37906866	37907951	8	1085	2	2	4	NA	NA	NA
Bone-Leiomyo	72f0a49a-aec8-47e5-846a-956c4da1507c	20	61079644	61082019	13	2375	1	1	11	NA	NA	NA
Bone-Leiomyo	72f0a49a-aec8-47e5-846a-956c4da1507c	20	61649873	61653112	11	3239	1	3	7	NA	NA	NA
Bone-Leiomyo	72f0a49a-aec8-47e5-846a-956c4da1507c	22	38575734	38580093	6	4359	1	3	2	NA	NA	NA
Bone-Leiomyo	72f0a49a-aec8-47e5-846a-956c4da1507c	22	47382178	47386672	10	4494	4	5	1	NA	NA	NA
Bone-Leiomyo	72f0a49a-aec8-47e5-846a-956c4da1507c	X	20263544	20266006	10	2462	1	2	7	NA	NA	NA
Bone-Leiomyo	72f0a49a-aec8-47e5-846a-956c4da1507c	X	21168456	21170896	9	2440	2	1	5	1	NA	NA
Bone-Leiomyo	72f0a49a-aec8-47e5-846a-956c4da1507c	X	35863047	35867507	7	4460	NA	2	5	NA	NA	NA
Bone-Leiomyo	72f0a49a-aec8-47e5-846a-956c4da1507c	X	73547157	73547915	8	758	1	5	2	NA	NA	NA
Bone-Leiomyo	72f0a49a-aec8-47e5-846a-956c4da1507c	X	73849823	73851681	7	1858	1	NA	6	NA	NA	NA
Panc-AdenoCA	72f82fbd-9838-4082-b605-bc3d80226f16	1	244327402	244328037	9	635	NA	NA	9	NA	NA	NA
Panc-AdenoCA	72f82fbd-9838-4082-b605-bc3d80226f16	3	30643856	30645010	6	1154	1	NA	5	NA	NA	NA
Panc-AdenoCA	72f82fbd-9838-4082-b605-bc3d80226f16	6	48129376	48130276	23	900	NA	1	22	NA	NA	NA
Panc-AdenoCA	73058c80-607e-4b55-b112-8f8f6775d014	16	67705742	67711212	15	5470	NA	NA	13	NA	2	NA
Breast-AdenoCa	73936e8b-c893-4afd-bd1f-be90c06a4869	4	62914338	62914660	6	322	1	4	1	NA	NA	NA
Breast-AdenoCa	73936e8b-c893-4afd-bd1f-be90c06a4869	9	14772098	14772245	6	147	3	1	2	NA	NA	NA
Breast-AdenoCa	73936e8b-c893-4afd-bd1f-be90c06a4869	11	77715378	77716416	8	1038	3	2	2	NA	NA	1
Liver-HCC	73afad06-c623-11e3-bf01-24c6515278c0	8	40369068	40371399	6	2331	NA	4	2	NA	NA	NA
Breast-AdenoCa	74039acd-5aca-4c65-818c-3b577d295be0	1	150447953	150448459	8	506	1	NA	7	NA	NA	NA
Breast-AdenoCa	74039acd-5aca-4c65-818c-3b577d295be0	8	39317806	39318466	8	660	1	4	2	1	NA	NA
Breast-AdenoCa	74039acd-5aca-4c65-818c-3b577d295be0	8	50227012	50228503	7	1491	3	2	1	NA	NA	1
Breast-AdenoCa	74039acd-5aca-4c65-818c-3b577d295be0	8	95240644	95247538	11	6894	4	2	5	NA	NA	NA
Breast-AdenoCa	74039acd-5aca-4c65-818c-3b577d295be0	8	97804963	97808444	7	3481	2	3	2	NA	NA	NA
Breast-AdenoCa	74039acd-5aca-4c65-818c-3b577d295be0	8	115662075	115667854	8	5779	1	1	6	NA	NA	NA
Breast-AdenoCa	74039acd-5aca-4c65-818c-3b577d295be0	8	117804178	117810393	10	6215	4	3	3	NA	NA	NA
Breast-AdenoCa	74039acd-5aca-4c65-818c-3b577d295be0	9	103029001	103029596	6	595	1	3	2	NA	NA	NA
Breast-AdenoCa	74039acd-5aca-4c65-818c-3b577d295be0	21	18035131	18038471	11	3340	5	4	2	NA	NA	NA
Breast-AdenoCa	74039acd-5aca-4c65-818c-3b577d295be0	21	21962443	21977717	21	15274	NA	6	14	NA	1	NA
Panc-AdenoCA	7410c00d-ed0b-48dc-a4bf-eb76ac613980	3	152247349	152248067	17	718	2	7	8	NA	NA	NA
Panc-AdenoCA	7410c00d-ed0b-48dc-a4bf-eb76ac613980	4	5872421	5880394	9	7973	NA	NA	NA	3	5	1
Panc-AdenoCA	7410c00d-ed0b-48dc-a4bf-eb76ac613980	5	97409527	97411813	6	2286	NA	NA	NA	1	5	NA
Panc-AdenoCA	7410c00d-ed0b-48dc-a4bf-eb76ac613980	5	162960153	162965879	18	5726	NA	NA	NA	9	4	5
Panc-AdenoCA	7410c00d-ed0b-48dc-a4bf-eb76ac613980	6	33689693	33694687	6	4994	NA	1	4	NA	NA	1
Panc-AdenoCA	7410c00d-ed0b-48dc-a4bf-eb76ac613980	7	40263192	40271541	11	8349	NA	NA	NA	4	5	2
Panc-AdenoCA	7410c00d-ed0b-48dc-a4bf-eb76ac613980	7	145498573	145508893	15	10320	2	NA	NA	2	4	7
Panc-AdenoCA	7410c00d-ed0b-48dc-a4bf-eb76ac613980	7	145571785	145577450	11	5665	NA	NA	NA	5	2	4
Panc-AdenoCA	7410c00d-ed0b-48dc-a4bf-eb76ac613980	8	91986989	91991537	8	4548	NA	NA	NA	1	7	NA
Panc-AdenoCA	7410c00d-ed0b-48dc-a4bf-eb76ac613980	8	143928268	143929181	7	913	2	5	NA	NA	NA	NA
Panc-AdenoCA	7410c00d-ed0b-48dc-a4bf-eb76ac613980	9	16378513	16393231	31	14718	NA	NA	NA	8	18	5
Panc-AdenoCA	7410c00d-ed0b-48dc-a4bf-eb76ac613980	11	124849639	124851088	6	1449	NA	NA	NA	2	3	1
Panc-AdenoCA	7410c00d-ed0b-48dc-a4bf-eb76ac613980	14	79729206	79735159	14	5953	NA	NA	NA	1	4	9
Panc-AdenoCA	7410c00d-ed0b-48dc-a4bf-eb76ac613980	15	82233247	82244604	23	11357	3	8	11	NA	1	NA
Panc-AdenoCA	7410c00d-ed0b-48dc-a4bf-eb76ac613980	19	35656463	35657780	6	1317	NA	3	3	NA	NA	NA
Panc-AdenoCA	7410c00d-ed0b-48dc-a4bf-eb76ac613980	X	113648417	113654579	8	6162	NA	NA	NA	4	3	1
Kidney-RCC	741c4656-0fd0-416e-86c8-f6533b6c96f4	2	40455081	40458650	6	3569	NA	NA	3	3	NA	NA
Kidney-RCC	741c4656-0fd0-416e-86c8-f6533b6c96f4	9	29224032	29226607	8	2575	1	NA	3	2	1	1
Breast-AdenoCa	7456abd5-303e-4e6f-bf4e-47efefc7310f	12	66661736	66662167	6	431	2	3	1	NA	NA	NA
Breast-AdenoCa	7456abd5-303e-4e6f-bf4e-47efefc7310f	13	73585540	73588081	10	2541	NA	7	3	NA	NA	NA
Breast-AdenoCa	7456abd5-303e-4e6f-bf4e-47efefc7310f	13	89092098	89099615	9	7517	2	7	NA	NA	NA	NA
Breast-AdenoCa	7456abd5-303e-4e6f-bf4e-47efefc7310f	13	89209467	89211235	9	1768	NA	9	NA	NA	NA	NA
Breast-AdenoCa	7456abd5-303e-4e6f-bf4e-47efefc7310f	13	96152548	96156986	8	4438	NA	6	2	NA	NA	NA
Breast-AdenoCa	7456abd5-303e-4e6f-bf4e-47efefc7310f	13	113382413	113387144	7	4731	NA	7	NA	NA	NA	NA
Ovary-AdenoCA	745b8756-0eab-423f-8cde-e0ff1aaa6596	12	33932450	33934053	6	1603	NA	2	4	NA	NA	NA
Panc-AdenoCA	748d3ff3-8699-4519-8e0f-26b6a0581bff	1	35080762	35080898	6	136	NA	2	4	NA	NA	NA

Panc-AdenoCA	748d3ff3-8699-4519-8e0f-26b6a0581bff	17	62333903	62337585	15	3682	NA	6	9	NA	NA	NA
Panc-AdenoCA	748d3ff3-8699-4519-8e0f-26b6a0581bff	17	68576426	68577768	8	1342	1	4	3	NA	NA	NA
Breast-AdenoCa	74b57d2c-a83f-4798-b86b-a533975e4cc0	3	8386950	8388786	6	1836	NA	3	3	NA	NA	NA
Breast-AdenoCa	74b57d2c-a83f-4798-b86b-a533975e4cc0	4	70685768	70690284	11	4516	2	7	2	NA	NA	NA
Breast-AdenoCa	74b57d2c-a83f-4798-b86b-a533975e4cc0	6	92141025	92141279	7	254	NA	2	5	NA	NA	NA
Breast-AdenoCa	74b57d2c-a83f-4798-b86b-a533975e4cc0	6	98166944	98178676	23	11732	7	15	1	NA	NA	NA
Breast-AdenoCa	74b57d2c-a83f-4798-b86b-a533975e4cc0	8	139974771	139977072	6	2301	1	4	1	NA	NA	NA
Breast-AdenoCa	74b57d2c-a83f-4798-b86b-a533975e4cc0	9	19667258	19667389	6	131	NA	5	1	NA	NA	NA
Breast-AdenoCa	74b57d2c-a83f-4798-b86b-a533975e4cc0	10	12678417	12680251	13	1834	3	5	4	NA	NA	1
Breast-AdenoCa	74b57d2c-a83f-4798-b86b-a533975e4cc0	11	62727558	62731426	7	3868	1	5	1	NA	NA	NA
Breast-AdenoCa	74b57d2c-a83f-4798-b86b-a533975e4cc0	13	21959101	21959924	7	823	NA	4	3	NA	NA	NA
Breast-AdenoCa	74b57d2c-a83f-4798-b86b-a533975e4cc0	17	25668825	25669528	10	703	2	1	7	NA	NA	NA
Breast-AdenoCa	74b57d2c-a83f-4798-b86b-a533975e4cc0	19	11886024	11890738	6	4714	NA	4	2	NA	NA	NA
Breast-AdenoCa	74b57d2c-a83f-4798-b86b-a533975e4cc0	20	50917102	50920612	6	3510	NA	1	5	NA	NA	NA
Skin-Melanoma	74e5905f-6350-498a-9454-9ea0593d2f56	7	8412047	8414683	8	2636	NA	2	6	NA	NA	NA
Skin-Melanoma	74e5905f-6350-498a-9454-9ea0593d2f56	8	29999866	30000492	8	626	NA	4	4	NA	NA	NA
Skin-Melanoma	74e5905f-6350-498a-9454-9ea0593d2f56	8	52850264	52856530	25	6266	2	16	7	NA	NA	NA
Skin-Melanoma	74e5905f-6350-498a-9454-9ea0593d2f56	8	54317893	54320206	8	2313	2	NA	5	NA	1	NA
Skin-Melanoma	74e5905f-6350-498a-9454-9ea0593d2f56	8	56561377	56564030	7	2653	NA	2	5	NA	NA	NA
Skin-Melanoma	74e5905f-6350-498a-9454-9ea0593d2f56	8	58281830	58287719	16	5889	4	6	6	NA	NA	NA
Skin-Melanoma	74e5905f-6350-498a-9454-9ea0593d2f56	8	71529901	71538004	12	8103	2	3	7	NA	NA	NA
Skin-Melanoma	74e5905f-6350-498a-9454-9ea0593d2f56	8	72894902	72898123	15	3221	1	7	6	1	NA	NA
Skin-Melanoma	74e5905f-6350-498a-9454-9ea0593d2f56	9	15752760	15753178	7	418	1	1	5	NA	NA	NA
Skin-Melanoma	74e5905f-6350-498a-9454-9ea0593d2f56	9	17242655	17243643	7	988	2	4	1	NA	NA	NA
Skin-Melanoma	74e5905f-6350-498a-9454-9ea0593d2f56	9	17692620	17696551	23	3931	6	9	8	NA	NA	NA
Skin-Melanoma	74e5905f-6350-498a-9454-9ea0593d2f56	9	24419687	24421908	10	2221	1	3	6	NA	NA	NA
Skin-Melanoma	74e5905f-6350-498a-9454-9ea0593d2f56	9	30246363	30248345	8	1982	1	3	4	NA	NA	NA
Skin-Melanoma	74e5905f-6350-498a-9454-9ea0593d2f56	9	36003378	36007156	20	3778	3	7	10	NA	NA	NA
Skin-Melanoma	74e5905f-6350-498a-9454-9ea0593d2f56	12	2866372	2867060	9	688	NA	8	1	NA	NA	NA
Skin-Melanoma	74e5905f-6350-498a-9454-9ea0593d2f56	12	6134211	6135561	9	1350	2	NA	7	NA	NA	NA
Skin-Melanoma	74e5905f-6350-498a-9454-9ea0593d2f56	16	27199361	27204924	16	5563	1	3	10	1	NA	1
Skin-Melanoma	74e5905f-6350-498a-9454-9ea0593d2f56	22	28919423	28921120	6	1697	NA	1	5	NA	NA	NA
Skin-Melanoma	74e5905f-6350-498a-9454-9ea0593d2f56	22	40987135	40994569	13	7434	1	4	8	NA	NA	NA
Skin-Melanoma	74e5905f-6350-498a-9454-9ea0593d2f56	22	44701385	44705424	7	4039	NA	2	5	NA	NA	NA
Skin-Melanoma	74e5905f-6350-498a-9454-9ea0593d2f56	X	154984172	154996113	28	11941	5	18	4	1	NA	NA
Biliary-AdenoCA	7512b38c-c623-11e3-bf01-24c6515278c0	1	207083779	207084646	8	867	NA	4	4	NA	NA	NA
Biliary-AdenoCA	7512b38c-c623-11e3-bf01-24c6515278c0	14	49435506	49436408	18	902	3	7	8	NA	NA	NA
Biliary-AdenoCA	7512b38c-c623-11e3-bf01-24c6515278c0	14	49704411	49706149	14	1738	4	6	4	NA	NA	NA
Biliary-AdenoCA	7512b38c-c623-11e3-bf01-24c6515278c0	17	15221176	15222886	6	1710	1	4	1	NA	NA	NA
ColoRect-AdenoCA	75ad15b9-8f9c-40c1-9ca6-1e8454fbd310	10	54411096	54411446	6	350	1	2	3	NA	NA	NA
ColoRect-AdenoCA	75ad15b9-8f9c-40c1-9ca6-1e8454fbd310	10	82362811	82362979	8	168	3	1	4	NA	NA	NA
Panc-AdenoCA	75cd90ef-e884-4812-bbf3-5a524d95147e	5	63622838	63623326	6	488	NA	NA	6	NA	NA	NA
Panc-AdenoCA	75cd90ef-e884-4812-bbf3-5a524d95147e	8	120705315	120708332	7	3017	1	3	3	NA	NA	NA
Panc-AdenoCA	75fac59c-3346-4c5a-bb9f-fb67fec8d13b	1	73255680	73257130	6	1450	NA	NA	NA	3	3	NA
Panc-AdenoCA	75fac59c-3346-4c5a-bb9f-fb67fec8d13b	1	107260850	107264056	7	3206	NA	NA	NA	3	3	1
Panc-AdenoCA	75fac59c-3346-4c5a-bb9f-fb67fec8d13b	15	95840206	95848982	16	8776	NA	4	11	NA	NA	1
Panc-AdenoCA	75fac59c-3346-4c5a-bb9f-fb67fec8d13b	20	24108822	24111472	13	2650	NA	NA	13	NA	NA	NA
Head-SCC	7625877a-7a2b-466f-944b-9e56689ad47b	8	31792734	31796875	36	4141	5	15	16	NA	NA	NA
Lymph-BNHL	76788f17-257d-47c7-ad85-ac5f959216b5	1	244997704	244997936	6	232	NA	NA	NA	4	1	1
Lymph-BNHL	76788f17-257d-47c7-ad85-ac5f959216b5	8	128748769	128749250	7	481	NA	2	2	2	NA	1
Lymph-BNHL	76788f17-257d-47c7-ad85-ac5f959216b5	10	83199806	83200204	6	398	NA	NA	1	2	NA	3
Lymph-BNHL	76788f17-257d-47c7-ad85-ac5f959216b5	14	106325262	106329998	43	4736	1	12	15	8	3	4
Lymph-BNHL	76788f17-257d-47c7-ad85-ac5f959216b5	14	106829757	106830599	9	842	NA	1	4	2	1	1
Lymph-BNHL	76788f17-257d-47c7-ad85-ac5f959216b5	22	22385525	22385843	18	318	4	2	4	4	1	3
Lymph-BNHL	76788f17-257d-47c7-ad85-ac5f959216b5	22	22759152	22764560	9	5408	2	2	4	1	NA	NA
Lymph-BNHL	76788f17-257d-47c7-ad85-ac5f959216b5	22	23235973	23247562	31	11589	4	6	12	4	1	4
Lung-SCC	7731ef7f-25bd-42f5-9a20-be249306a711	1	84954548	84956337	7	1789	NA	5	2	NA	NA	NA
Lung-SCC	7731ef7f-25bd-42f5-9a20-be249306a711	1	160732164	160735319	7	3155	NA	2	5	NA	NA	NA
Lung-SCC	7731ef7f-25bd-42f5-9a20-be249306a711	2	231758457	231760762	6	2305	1	3	2	NA	NA	NA
Lung-SCC	7731ef7f-25bd-42f5-9a20-be249306a711	3	11818145	11820596	6	2451	NA	4	2	NA	NA	NA

Lung-SCC	7731ef7f-25bd-42f5-9a20-be249306a711	3	172337345	172340133	8	2788	NA	6	2	NA	NA	NA
Lung-SCC	7731ef7f-25bd-42f5-9a20-be249306a711	5	43007591	43010680	6	3089	NA	1	5	NA	NA	NA
Lung-SCC	7731ef7f-25bd-42f5-9a20-be249306a711	6	37659491	37662122	6	2631	1	2	3	NA	NA	NA
Lung-SCC	7731ef7f-25bd-42f5-9a20-be249306a711	6	89539161	89543868	9	4707	NA	4	5	NA	NA	NA
Lung-SCC	7731ef7f-25bd-42f5-9a20-be249306a711	8	1416751	1418805	7	2054	1	3	3	NA	NA	NA
Lung-SCC	7731ef7f-25bd-42f5-9a20-be249306a711	9	34149182	34152068	8	2886	NA	4	3	1	NA	NA
Lung-SCC	7731ef7f-25bd-42f5-9a20-be249306a711	10	63721194	63727840	10	6646	3	2	4	NA	NA	1
Lung-SCC	7731ef7f-25bd-42f5-9a20-be249306a711	10	89533772	89536902	9	3130	NA	6	3	NA	NA	NA
Lung-SCC	7731ef7f-25bd-42f5-9a20-be249306a711	11	47480895	47483196	7	2301	NA	2	5	NA	NA	NA
Lung-SCC	7731ef7f-25bd-42f5-9a20-be249306a711	11	112038634	112042538	11	3904	1	2	8	NA	NA	NA
Lung-SCC	7731ef7f-25bd-42f5-9a20-be249306a711	12	54674799	54677812	6	3013	NA	3	3	NA	NA	NA
Lung-SCC	7731ef7f-25bd-42f5-9a20-be249306a711	14	36156040	36158739	12	2699	6	2	3	NA	NA	1
Lung-SCC	7731ef7f-25bd-42f5-9a20-be249306a711	14	36227677	36233656	11	5979	NA	5	6	NA	NA	NA
Lung-SCC	7731ef7f-25bd-42f5-9a20-be249306a711	14	64262671	64267267	8	4596	NA	4	4	NA	NA	NA
Lung-SCC	7731ef7f-25bd-42f5-9a20-be249306a711	15	63822647	63826107	7	3460	3	4	NA	NA	NA	NA
Lung-SCC	7731ef7f-25bd-42f5-9a20-be249306a711	16	503570	504179	6	609	2	NA	4	NA	NA	NA
Lung-SCC	7731ef7f-25bd-42f5-9a20-be249306a711	16	11270582	11274315	8	3733	NA	4	4	NA	NA	NA
Lung-SCC	7731ef7f-25bd-42f5-9a20-be249306a711	17	28015817	28017875	7	2058	1	3	3	NA	NA	NA
Lung-SCC	7731ef7f-25bd-42f5-9a20-be249306a711	19	11612641	11620617	12	7976	6	3	2	NA	NA	1
Lung-SCC	7731ef7f-25bd-42f5-9a20-be249306a711	19	14497335	14515222	21	17887	1	9	11	NA	NA	NA
Lung-SCC	7731ef7f-25bd-42f5-9a20-be249306a711	20	23039103	23041934	6	2831	NA	2	4	NA	NA	NA
Lung-SCC	7731ef7f-25bd-42f5-9a20-be249306a711	22	19559142	19563905	7	4763	1	6	NA	NA	NA	NA
Biliary-AdenoCA	7789ef18-c623-11e3-bf01-24c6515278c0	1	195781190	195781745	6	555	NA	1	5	NA	NA	NA
Biliary-AdenoCA	7789ef18-c623-11e3-bf01-24c6515278c0	10	14788388	14792387	6	3999	2	2	2	NA	NA	NA
Liver-HCC	77bf9514-88da-473b-9a91-f669ef3cd343	6	35615547	35616115	7	568	NA	NA	NA	4	2	1
Eso-AdenoCa	77c022d3-2f29-49d3-a1df-cc2e9407fd7f	7	56489111	56492857	16	3746	4	12	NA	NA	NA	NA
Eso-AdenoCa	77c022d3-2f29-49d3-a1df-cc2e9407fd7f	13	115003616	115005353	8	1737	2	3	3	NA	NA	NA
Eso-AdenoCa	77c022d3-2f29-49d3-a1df-cc2e9407fd7f	18	21463625	21464215	7	590	3	3	1	NA	NA	NA
Panc-AdenoCA	78100212-65aa-4365-8b64-4b33f77732d5	1	242938606	242942060	12	3454	1	NA	NA	2	6	3
Panc-AdenoCA	78100212-65aa-4365-8b64-4b33f77732d5	2	221600683	221607449	8	6766	NA	NA	NA	2	5	1
Panc-AdenoCA	78100212-65aa-4365-8b64-4b33f77732d5	3	117879725	117883481	12	3756	1	NA	NA	1	5	5
Panc-AdenoCA	78100212-65aa-4365-8b64-4b33f77732d5	4	34255039	34256993	6	1954	NA	NA	NA	3	2	1
Panc-AdenoCA	78100212-65aa-4365-8b64-4b33f77732d5	8	3775973	3780052	6	4079	NA	NA	NA	1	2	3
Panc-AdenoCA	78100212-65aa-4365-8b64-4b33f77732d5	8	41418342	41419871	9	1529	NA	5	4	NA	NA	NA
Panc-AdenoCA	78100212-65aa-4365-8b64-4b33f77732d5	11	89015697	89021518	8	5821	1	NA	NA	NA	5	2
Panc-AdenoCA	78100212-65aa-4365-8b64-4b33f77732d5	11	89038327	89041898	8	3571	1	NA	NA	1	1	5
Panc-AdenoCA	78100212-65aa-4365-8b64-4b33f77732d5	12	33795837	33798716	14	2879	NA	1	NA	1	10	2
Panc-AdenoCA	78100212-65aa-4365-8b64-4b33f77732d5	12	34523965	34524764	8	799	1	1	6	NA	NA	NA
Panc-AdenoCA	78100212-65aa-4365-8b64-4b33f77732d5	13	86656938	86661325	6	4387	NA	NA	NA	1	5	NA
Panc-AdenoCA	78100212-65aa-4365-8b64-4b33f77732d5	X	26406148	26410629	6	4481	NA	NA	2	NA	NA	4
Panc-AdenoCA	78100212-65aa-4365-8b64-4b33f77732d5	X	122844898	122847705	7	2807	1	NA	NA	NA	2	4
Prost-AdenoCA	783579bd-e29d-457c-8236-1a80b557600c	1	193571996	193577913	18	5917	1	16	1	NA	NA	NA
Prost-AdenoCA	783579bd-e29d-457c-8236-1a80b557600c	1	216137993	216140299	15	2306	1	8	6	NA	NA	NA
Prost-AdenoCA	783579bd-e29d-457c-8236-1a80b557600c	4	162797802	162800850	13	3048	NA	9	4	NA	NA	NA
Prost-AdenoCA	783579bd-e29d-457c-8236-1a80b557600c	4	168730821	168733390	9	2569	NA	5	4	NA	NA	NA
Eso-AdenoCa	7866dfb2-46b3-42b4-905b-12f80593d6bd	3	117667495	117668248	6	753	1	3	2	NA	NA	NA
Eso-AdenoCa	7866dfb2-46b3-42b4-905b-12f80593d6bd	3	157310701	157314119	9	3418	3	2	4	NA	NA	NA
Eso-AdenoCa	7866dfb2-46b3-42b4-905b-12f80593d6bd	3	194164273	194166075	9	1802	3	3	3	NA	NA	NA
Eso-AdenoCa	7866dfb2-46b3-42b4-905b-12f80593d6bd	4	32108432	32109856	6	1424	1	2	3	NA	NA	NA
Eso-AdenoCa	7866dfb2-46b3-42b4-905b-12f80593d6bd	4	93510944	93513834	7	2890	NA	2	5	NA	NA	NA
Eso-AdenoCa	7866dfb2-46b3-42b4-905b-12f80593d6bd	7	54401455	54416503	18	15048	1	4	12	NA	1	NA
Eso-AdenoCa	7866dfb2-46b3-42b4-905b-12f80593d6bd	7	86555425	86560695	9	5270	NA	2	7	NA	NA	NA
Eso-AdenoCa	7866dfb2-46b3-42b4-905b-12f80593d6bd	8	78008345	78012827	6	4482	2	2	2	NA	NA	NA
Eso-AdenoCa	7866dfb2-46b3-42b4-905b-12f80593d6bd	8	130629026	130630373	7	1347	2	4	1	NA	NA	NA
Eso-AdenoCa	7866dfb2-46b3-42b4-905b-12f80593d6bd	11	17235950	17236775	7	825	6	1	NA	NA	NA	NA
Eso-AdenoCa	7866dfb2-46b3-42b4-905b-12f80593d6bd	14	33956683	33961631	10	4948	1	NA	9	NA	NA	NA
Eso-AdenoCa	7866dfb2-46b3-42b4-905b-12f80593d6bd	14	38780545	38790438	16	9893	7	4	5	NA	NA	NA
Eso-AdenoCa	7866dfb2-46b3-42b4-905b-12f80593d6bd	14	49141541	49146827	8	5286	2	2	3	NA	NA	1
Eso-AdenoCa	7866dfb2-46b3-42b4-905b-12f80593d6bd	14	59780368	59791977	18	11609	2	2	12	NA	NA	2
ColoRect-AdenoCA	786fc3e4-e2bf-4914-9251-41c800ebb2fa	10	573771	580513	10	6742	1	2	4	1	2	NA

ColoRect-AdenoCA	786fc3e4-e2bf-4914-9251-41c800ebb2fa	10	585580	587180	8	1600	1	2	1	1	3	NA
ColoRect-AdenoCA	786fc3e4-e2bf-4914-9251-41c800ebb2fa	10	615243	617531	11	2288	3	2	2	2	1	1
ColoRect-AdenoCA	786fc3e4-e2bf-4914-9251-41c800ebb2fa	10	647199	652743	9	5544	1	2	2	1	1	2
ColoRect-AdenoCA	786fc3e4-e2bf-4914-9251-41c800ebb2fa	10	663227	668146	9	4919	2	1	4	1	NA	1
Lymph-BNHL	78bd2aa8-e5f6-4b4b-96f0-d406fbdbcee8	2	89157454	89165170	27	7716	NA	3	14	8	1	1
Lymph-BNHL	78bd2aa8-e5f6-4b4b-96f0-d406fbdbcee8	3	120127380	120132551	12	5171	NA	6	6	NA	NA	NA
Lymph-BNHL	78bd2aa8-e5f6-4b4b-96f0-d406fbdbcee8	8	128749174	128753183	11	4009	2	1	8	NA	NA	NA
Lymph-BNHL	78bd2aa8-e5f6-4b4b-96f0-d406fbdbcee8	14	106173255	106178655	11	5400	1	1	9	NA	NA	NA
Lymph-BNHL	78bd2aa8-e5f6-4b4b-96f0-d406fbdbcee8	14	106320634	106330618	23	9984	2	3	17	1	NA	NA
Lymph-BNHL	78bd2aa8-e5f6-4b4b-96f0-d406fbdbcee8	22	23230477	23235602	9	5125	NA	NA	9	NA	NA	NA
Panc-Endocrine	79353875-9d86-4063-a8ea-1d998acb1e81	12	65337864	65341354	12	3490	NA	5	6	NA	1	NA
Biliary-AdenoCA	796a8782-6d7e-47c8-b322-fcfe1dd240c8	4	114075852	114079935	6	4083	2	NA	4	NA	NA	NA
Biliary-AdenoCA	796a8782-6d7e-47c8-b322-fcfe1dd240c8	10	44353402	44354661	10	1259	3	1	6	NA	NA	NA
Biliary-AdenoCA	796a8782-6d7e-47c8-b322-fcfe1dd240c8	10	64094671	64095569	9	898	5	1	3	NA	NA	NA
Biliary-AdenoCA	796a8782-6d7e-47c8-b322-fcfe1dd240c8	20	55923710	55924796	11	1086	6	4	1	NA	NA	NA
Biliary-AdenoCA	796a8782-6d7e-47c8-b322-fcfe1dd240c8	X	8414342	8419860	18	5518	6	8	3	NA	NA	1
Panc-AdenoCA	7981bbf3-dceb-4d89-900c-555967e97921	22	28371318	28371479	8	161	NA	1	6	NA	NA	1
Eso-AdenoCa	7a4f6014-f7a2-459b-bc43-df164e73cbba	18	45498907	45498992	6	85	NA	2	4	NA	NA	NA
Eso-AdenoCa	7a4f6014-f7a2-459b-bc43-df164e73cbba	20	23201491	23202129	9	638	1	3	5	NA	NA	NA
Eso-AdenoCa	7a4f6014-f7a2-459b-bc43-df164e73cbba	20	23214481	23215847	7	1366	NA	2	NA	2	2	1
Bone-Leiomyo	7a5d3b6b-be5a-4e55-9255-32a069b8af3d	4	56700117	56701516	6	1399	1	1	3	NA	NA	1
Bone-Leiomyo	7a5d3b6b-be5a-4e55-9255-32a069b8af3d	9	1318690	1319745	20	1055	2	7	11	NA	NA	NA
Bone-Leiomyo	7a5d3b6b-be5a-4e55-9255-32a069b8af3d	9	2244970	2251619	13	6649	1	2	10	NA	NA	NA
Bone-Leiomyo	7a5d3b6b-be5a-4e55-9255-32a069b8af3d	9	2780524	2783648	12	3124	2	1	9	NA	NA	NA
Bone-Leiomyo	7a5d3b6b-be5a-4e55-9255-32a069b8af3d	9	11342531	11349964	18	7433	NA	NA	17	NA	1	NA
Bone-Leiomyo	7a5d3b6b-be5a-4e55-9255-32a069b8af3d	9	21788095	21788730	7	635	NA	3	4	NA	NA	NA
Bone-Leiomyo	7a5d3b6b-be5a-4e55-9255-32a069b8af3d	9	21943700	21952749	25	9049	1	4	18	NA	2	NA
Bone-Leiomyo	7a5d3b6b-be5a-4e55-9255-32a069b8af3d	16	48010034	48014651	7	4617	NA	NA	7	NA	NA	NA
Liver-HCC	7a6cb147-a576-446d-a605-fa846ea3d1ad	3	56035400	56035877	9	477	NA	8	1	NA	NA	NA
Eso-AdenoCa	7adcea71-1aed-450c-b0ff-b9d9820c6153	4	41386855	41389113	22	2258	2	7	13	NA	NA	NA
Eso-AdenoCa	7adcea71-1aed-450c-b0ff-b9d9820c6153	5	86641698	86643372	8	1674	NA	6	2	NA	NA	NA
Cervix-SCC	7bc3209f-b349-49bf-9b3d-44ef870b586f	10	69730187	69734930	9	4743	2	3	4	NA	NA	NA
Liver-HCC	7c405ca0-c622-11e3-bf01-24c6515278c0	4	99197909	99200051	8	2142	1	7	NA	NA	NA	NA
Panc-AdenoCA	7c43c006-d917-4915-b304-65995b6c0d46	2	33260618	33262215	9	1597	NA	NA	9	NA	NA	NA
Panc-AdenoCA	7c43c006-d917-4915-b304-65995b6c0d46	2	33834601	33835002	9	401	1	5	3	NA	NA	NA
Panc-AdenoCA	7c43c006-d917-4915-b304-65995b6c0d46	18	18921055	18923355	9	2300	1	1	7	NA	NA	NA
Panc-AdenoCA	7c43c006-d917-4915-b304-65995b6c0d46	18	20576935	20578049	11	1114	2	1	8	NA	NA	NA
Panc-AdenoCA	7c43c006-d917-4915-b304-65995b6c0d46	18	71466536	71476590	15	10054	1	6	8	NA	NA	NA
Liver-HCC	7cd7fe38-c623-11e3-bf01-24c6515278c0	15	54347861	54348218	7	357	NA	2	5	NA	NA	NA
Breast-LobularCa	7cdbe0e8-f614-4f54-b864-fd6b39e8ef1c	2	25710894	25721039	13	10145	2	7	4	NA	NA	NA
Breast-LobularCa	7cdbe0e8-f614-4f54-b864-fd6b39e8ef1c	2	195390542	195395202	9	4660	3	6	NA	NA	NA	NA
Breast-LobularCa	7cdbe0e8-f614-4f54-b864-fd6b39e8ef1c	3	48132488	48141748	19	9260	2	9	8	NA	NA	NA
Breast-LobularCa	7cdbe0e8-f614-4f54-b864-fd6b39e8ef1c	3	49262634	49267796	7	5162	3	2	2	NA	NA	NA
Breast-LobularCa	7cdbe0e8-f614-4f54-b864-fd6b39e8ef1c	3	49269664	49275980	11	6316	3	2	5	NA	NA	1
Breast-LobularCa	7cdbe0e8-f614-4f54-b864-fd6b39e8ef1c	5	66255870	66260093	9	4223	NA	7	2	NA	NA	NA
Breast-LobularCa	7cdbe0e8-f614-4f54-b864-fd6b39e8ef1c	7	107566375	107572308	9	5933	NA	3	5	NA	1	NA
Breast-LobularCa	7cdbe0e8-f614-4f54-b864-fd6b39e8ef1c	8	87394042	87396426	6	2384	1	2	3	NA	NA	NA
Breast-LobularCa	7cdbe0e8-f614-4f54-b864-fd6b39e8ef1c	9	108082802	108087047	8	4245	1	2	5	NA	NA	NA
Breast-LobularCa	7cdbe0e8-f614-4f54-b864-fd6b39e8ef1c	10	128998424	129003242	9	4818	2	3	4	NA	NA	NA
Breast-LobularCa	7cdbe0e8-f614-4f54-b864-fd6b39e8ef1c	11	11386068	11392426	8	6358	NA	2	5	NA	NA	1
Breast-LobularCa	7cdbe0e8-f614-4f54-b864-fd6b39e8ef1c	11	119149658	119160891	17	11233	3	9	5	NA	NA	NA
Breast-LobularCa	7cdbe0e8-f614-4f54-b864-fd6b39e8ef1c	12	48281911	48285381	6	3470	NA	3	3	NA	NA	NA
Breast-LobularCa	7cdbe0e8-f614-4f54-b864-fd6b39e8ef1c	13	20575244	20579672	11	4428	NA	6	5	NA	NA	NA
Breast-LobularCa	7cdbe0e8-f614-4f54-b864-fd6b39e8ef1c	13	49306025	49306428	9	403	NA	NA	9	NA	NA	NA
Breast-LobularCa	7cdbe0e8-f614-4f54-b864-fd6b39e8ef1c	19	56095042	56109162	19	14120	3	6	9	NA	1	NA
Breast-LobularCa	7cdbe0e8-f614-4f54-b864-fd6b39e8ef1c	20	4331573	4334890	6	3317	1	NA	4	NA	NA	1
Panc-AdenoCA	7d04f401-3505-410c-8def-0348ecd8aae2	7	4590968	4591770	6	802	1	1	3	NA	1	NA
Panc-AdenoCA	7d04f401-3505-410c-8def-0348ecd8aae2	17	20962886	20963695	11	809	NA	4	7	NA	NA	NA
Panc-AdenoCA	7d04f401-3505-410c-8def-0348ecd8aae2	18	22621824	22622864	8	1040	1	4	3	NA	NA	NA
Panc-AdenoCA	7d04f401-3505-410c-8def-0348ecd8aae2	X	81331773	81333438	13	1665	4	3	6	NA	NA	NA

Panc-AdenoCA	7d04f401-3505-410c-8def-0348ecd8aae2	X	94105822	94112324	11	6502	NA	4	6	1	NA	NA
Bladder-TCC	7d2a22eb-7344-4cba-ad7d-94c3f9ef3d7c	1	26657114	26662929	9	5815	NA	4	5	NA	NA	NA
Bladder-TCC	7d2a22eb-7344-4cba-ad7d-94c3f9ef3d7c	1	225846934	225853268	13	6334	1	7	5	NA	NA	NA
Bladder-TCC	7d2a22eb-7344-4cba-ad7d-94c3f9ef3d7c	2	106082651	106084692	7	2041	NA	6	1	NA	NA	NA
Bladder-TCC	7d2a22eb-7344-4cba-ad7d-94c3f9ef3d7c	2	110436585	110439087	8	2502	NA	4	4	NA	NA	NA
Bladder-TCC	7d2a22eb-7344-4cba-ad7d-94c3f9ef3d7c	7	21549760	21551684	12	1924	3	5	4	NA	NA	NA
Bladder-TCC	7d2a22eb-7344-4cba-ad7d-94c3f9ef3d7c	7	30385675	30386885	7	1210	NA	4	3	NA	NA	NA
Bladder-TCC	7d2a22eb-7344-4cba-ad7d-94c3f9ef3d7c	7	99968925	99971663	12	2738	1	3	8	NA	NA	NA
Bladder-TCC	7d2a22eb-7344-4cba-ad7d-94c3f9ef3d7c	8	25171959	25172763	8	804	1	2	5	NA	NA	NA
Bladder-TCC	7d2a22eb-7344-4cba-ad7d-94c3f9ef3d7c	11	57591762	57592950	7	1188	1	4	2	NA	NA	NA
Bladder-TCC	7d2a22eb-7344-4cba-ad7d-94c3f9ef3d7c	14	24626922	24629201	6	2279	NA	3	3	NA	NA	NA
Bladder-TCC	7d2a22eb-7344-4cba-ad7d-94c3f9ef3d7c	15	51901431	51902607	6	1176	1	2	3	NA	NA	NA
Bladder-TCC	7d2a22eb-7344-4cba-ad7d-94c3f9ef3d7c	15	65972698	65977833	15	5135	NA	5	10	NA	NA	NA
Bladder-TCC	7d2a22eb-7344-4cba-ad7d-94c3f9ef3d7c	19	16532171	16537968	18	5797	1	4	13	NA	NA	NA
Bladder-TCC	7d2a22eb-7344-4cba-ad7d-94c3f9ef3d7c	Y	15361985	15376905	32	14920	2	11	19	NA	NA	NA
Eso-AdenoCa	7d2da7ba-f57c-4083-9572-6fa98008083b	2	10772468	10773423	10	955	1	5	4	NA	NA	NA
Eso-AdenoCa	7d2da7ba-f57c-4083-9572-6fa98008083b	2	11640661	11644111	15	3450	NA	4	10	NA	1	NA
Eso-AdenoCa	7d2da7ba-f57c-4083-9572-6fa98008083b	2	16638648	16645007	11	6359	NA	5	5	NA	1	NA
Eso-AdenoCa	7d2da7ba-f57c-4083-9572-6fa98008083b	2	107357963	107362115	12	4152	1	9	2	NA	NA	NA
Eso-AdenoCa	7d2da7ba-f57c-4083-9572-6fa98008083b	7	46804518	46805398	6	880	NA	1	2	1	2	NA
Eso-AdenoCa	7d2da7ba-f57c-4083-9572-6fa98008083b	7	53979194	53979445	7	251	NA	3	4	NA	NA	NA
Eso-AdenoCa	7d2da7ba-f57c-4083-9572-6fa98008083b	9	78740140	78743114	10	2974	1	3	6	NA	NA	NA
Eso-AdenoCa	7d2da7ba-f57c-4083-9572-6fa98008083b	11	132868460	132875898	14	7438	1	NA	13	NA	NA	NA
Eso-AdenoCa	7d2da7ba-f57c-4083-9572-6fa98008083b	13	107382917	107385880	16	2963	NA	7	9	NA	NA	NA
Eso-AdenoCa	7d2da7ba-f57c-4083-9572-6fa98008083b	16	47615954	47618736	10	2782	5	1	4	NA	NA	NA
Eso-AdenoCa	7d2da7ba-f57c-4083-9572-6fa98008083b	18	20156961	20159454	6	2493	2	1	3	NA	NA	NA
Bone-Leiomyo	7d332cb1-ba25-47e4-8bf8-d25e14f40d59	1	146803001	146806208	10	3207	NA	3	7	NA	NA	NA
Bone-Leiomyo	7d332cb1-ba25-47e4-8bf8-d25e14f40d59	1	168432732	168441477	15	8745	3	1	11	NA	NA	NA
Bone-Leiomyo	7d332cb1-ba25-47e4-8bf8-d25e14f40d59	1	171537273	171538653	14	1380	1	5	8	NA	NA	NA
Bone-Leiomyo	7d332cb1-ba25-47e4-8bf8-d25e14f40d59	1	171707881	171708653	7	772	2	5	NA	NA	NA	NA
Bone-Leiomyo	7d332cb1-ba25-47e4-8bf8-d25e14f40d59	1	174670392	174672627	11	2235	3	4	3	NA	1	NA
Bone-Leiomyo	7d332cb1-ba25-47e4-8bf8-d25e14f40d59	1	179956503	179961245	9	4742	1	4	4	NA	NA	NA
Bone-Leiomyo	7d332cb1-ba25-47e4-8bf8-d25e14f40d59	1	185710490	185714333	8	3843	1	5	2	NA	NA	NA
Bone-Leiomyo	7d332cb1-ba25-47e4-8bf8-d25e14f40d59	1	225121930	225123389	10	1459	1	5	4	NA	NA	NA
Bone-Leiomyo	7d332cb1-ba25-47e4-8bf8-d25e14f40d59	1	225915531	225916320	18	789	NA	NA	18	NA	NA	NA
Bone-Leiomyo	7d332cb1-ba25-47e4-8bf8-d25e14f40d59	2	155719434	155720609	13	1175	NA	4	9	NA	NA	NA
Bone-Leiomyo	7d332cb1-ba25-47e4-8bf8-d25e14f40d59	3	63714150	63714836	6	686	NA	4	2	NA	NA	NA
Bone-Leiomyo	7d332cb1-ba25-47e4-8bf8-d25e14f40d59	4	7187212	7187850	8	638	1	5	2	NA	NA	NA
Bone-Leiomyo	7d332cb1-ba25-47e4-8bf8-d25e14f40d59	5	19273647	19274799	15	1152	1	5	7	NA	NA	2
Bone-Leiomyo	7d332cb1-ba25-47e4-8bf8-d25e14f40d59	5	30940398	30943852	8	3454	1	3	4	NA	NA	NA
Bone-Leiomyo	7d332cb1-ba25-47e4-8bf8-d25e14f40d59	5	170771233	170773771	10	2538	NA	8	2	NA	NA	NA
Bone-Leiomyo	7d332cb1-ba25-47e4-8bf8-d25e14f40d59	5	171966801	171969401	8	2600	NA	1	7	NA	NA	NA
Bone-Leiomyo	7d332cb1-ba25-47e4-8bf8-d25e14f40d59	5	173458781	173463880	15	5099	1	8	6	NA	NA	NA
Bone-Leiomyo	7d332cb1-ba25-47e4-8bf8-d25e14f40d59	6	47479070	47481477	6	2407	NA	2	4	NA	NA	NA
Bone-Leiomyo	7d332cb1-ba25-47e4-8bf8-d25e14f40d59	6	50536586	50539102	17	2516	NA	NA	17	NA	NA	NA
Bone-Leiomyo	7d332cb1-ba25-47e4-8bf8-d25e14f40d59	6	91706741	91711401	10	4660	1	8	1	NA	NA	NA
Bone-Leiomyo	7d332cb1-ba25-47e4-8bf8-d25e14f40d59	7	12054821	12057685	8	2864	1	4	3	NA	NA	NA
Bone-Leiomyo	7d332cb1-ba25-47e4-8bf8-d25e14f40d59	7	26757880	26763758	7	5878	NA	5	2	NA	NA	NA
Bone-Leiomyo	7d332cb1-ba25-47e4-8bf8-d25e14f40d59	7	28214598	28216905	12	2307	NA	1	11	NA	NA	NA
Bone-Leiomyo	7d332cb1-ba25-47e4-8bf8-d25e14f40d59	7	28471952	28477661	14	5709	1	1	12	NA	NA	NA
Bone-Leiomyo	7d332cb1-ba25-47e4-8bf8-d25e14f40d59	7	81675936	81676617	24	681	NA	NA	24	NA	NA	NA
Bone-Leiomyo	7d332cb1-ba25-47e4-8bf8-d25e14f40d59	7	83382267	83385509	11	3242	1	5	4	NA	NA	1
Bone-Leiomyo	7d332cb1-ba25-47e4-8bf8-d25e14f40d59	7	87510775	87521198	18	10423	1	13	4	NA	NA	NA
Bone-Leiomyo	7d332cb1-ba25-47e4-8bf8-d25e14f40d59	7	89999167	90001026	8	1859	3	3	2	NA	NA	NA
Bone-Leiomyo	7d332cb1-ba25-47e4-8bf8-d25e14f40d59	7	119965627	119967180	7	1553	NA	3	4	NA	NA	NA
Bone-Leiomyo	7d332cb1-ba25-47e4-8bf8-d25e14f40d59	9	87513012	87517519	14	4507	1	8	5	NA	NA	NA
Bone-Leiomyo	7d332cb1-ba25-47e4-8bf8-d25e14f40d59	10	8565988	8566814	6	826	NA	NA	6	NA	NA	NA
Bone-Leiomyo	7d332cb1-ba25-47e4-8bf8-d25e14f40d59	10	11482136	11484723	8	2587	1	4	3	NA	NA	NA
Bone-Leiomyo	7d332cb1-ba25-47e4-8bf8-d25e14f40d59	11	13174174	13174870	9	696	NA	NA	9	NA	NA	NA
Bone-Leiomyo	7d332cb1-ba25-47e4-8bf8-d25e14f40d59	11	74741880	74742687	8	807	NA	NA	8	NA	NA	NA

Bone-Leiomyo	7d332cb1-ba25-47e4-8bf8-d25e14f40d59	12	63898015	63901054	6	3039	NA	4	2	NA	NA	NA
Bone-Leiomyo	7d332cb1-ba25-47e4-8bf8-d25e14f40d59	12	64956976	64958276	7	1300	NA	NA	7	NA	NA	NA
Bone-Leiomyo	7d332cb1-ba25-47e4-8bf8-d25e14f40d59	12	65128092	65158724	35	30632	NA	NA	34	1	NA	NA
Bone-Leiomyo	7d332cb1-ba25-47e4-8bf8-d25e14f40d59	12	69403470	69404560	15	1090	1	3	10	NA	1	NA
Bone-Leiomyo	7d332cb1-ba25-47e4-8bf8-d25e14f40d59	12	70330170	70331121	10	951	2	7	1	NA	NA	NA
Bone-Leiomyo	7d332cb1-ba25-47e4-8bf8-d25e14f40d59	12	70551147	70554134	11	2987	NA	NA	11	NA	NA	NA
Bone-Leiomyo	7d332cb1-ba25-47e4-8bf8-d25e14f40d59	12	71712205	71718456	8	6251	1	3	4	NA	NA	NA
Bone-Leiomyo	7d332cb1-ba25-47e4-8bf8-d25e14f40d59	12	77989403	77992574	11	3171	2	5	4	NA	NA	NA
Bone-Leiomyo	7d332cb1-ba25-47e4-8bf8-d25e14f40d59	12	81068145	81068776	9	631	NA	1	8	NA	NA	NA
Bone-Leiomyo	7d332cb1-ba25-47e4-8bf8-d25e14f40d59	12	83153829	83160814	10	6985	NA	6	4	NA	NA	NA
Bone-Leiomyo	7d332cb1-ba25-47e4-8bf8-d25e14f40d59	12	83513501	83515657	9	2156	1	3	5	NA	NA	NA
Bone-Leiomyo	7d332cb1-ba25-47e4-8bf8-d25e14f40d59	12	85289837	85312116	27	22279	NA	1	26	NA	NA	NA
Bone-Leiomyo	7d332cb1-ba25-47e4-8bf8-d25e14f40d59	12	85504739	85506126	7	1387	NA	5	2	NA	NA	NA
Bone-Leiomyo	7d332cb1-ba25-47e4-8bf8-d25e14f40d59	17	77143897	77146689	24	2792	NA	NA	24	NA	NA	NA
Bone-Leiomyo	7d332cb1-ba25-47e4-8bf8-d25e14f40d59	17	77206503	77209509	22	3006	NA	NA	22	NA	NA	NA
Bone-Leiomyo	7d332cb1-ba25-47e4-8bf8-d25e14f40d59	17	77376575	77378426	16	1851	NA	1	15	NA	NA	NA
Bone-Leiomyo	7d332cb1-ba25-47e4-8bf8-d25e14f40d59	19	41205719	41211114	7	5395	1	4	1	NA	1	NA
Bone-Leiomyo	7d332cb1-ba25-47e4-8bf8-d25e14f40d59	X	2685245	2687298	7	2053	1	5	1	NA	NA	NA
Bone-Leiomyo	7d332cb1-ba25-47e4-8bf8-d25e14f40d59	X	149784689	149785291	11	602	NA	NA	11	NA	NA	NA
ColoRect-AdenoCA	7d59205a-65ec-4341-9748-28639d82ed29	3	84136399	84138004	7	1605	1	3	3	NA	NA	NA
Prost-AdenoCA	7d6fb1a7-7742-4b8c-9903-abff706240b5	2	151232954	151234017	8	1063	2	4	2	NA	NA	NA
Prost-AdenoCA	7d6fb1a7-7742-4b8c-9903-abff706240b5	2	152171310	152174009	13	2699	3	4	6	NA	NA	NA
Prost-AdenoCA	7d6fb1a7-7742-4b8c-9903-abff706240b5	13	20993820	20995637	15	1817	2	5	8	NA	NA	NA
Prost-AdenoCA	7d6fb1a7-7742-4b8c-9903-abff706240b5	13	21347875	21351183	12	3308	1	5	6	NA	NA	NA
Prost-AdenoCA	7d6fb1a7-7742-4b8c-9903-abff706240b5	13	103364954	103366196	14	1242	NA	7	7	NA	NA	NA
Prost-AdenoCA	7d6fb1a7-7742-4b8c-9903-abff706240b5	13	103404532	103407690	12	3158	NA	6	6	NA	NA	NA
Prost-AdenoCA	7d6fb1a7-7742-4b8c-9903-abff706240b5	19	22900446	22904614	8	4168	NA	3	5	NA	NA	NA
Prost-AdenoCA	7d6fb1a7-7742-4b8c-9903-abff706240b5	19	57077929	57078575	8	646	1	1	6	NA	NA	NA
Cervix-SCC	7dc3e035-c7ae-4164-a62b-9c2460812b05	6	28951564	28953755	7	2191	NA	3	4	NA	NA	NA
Cervix-SCC	7dc3e035-c7ae-4164-a62b-9c2460812b05	11	68471439	68472687	11	1248	1	3	7	NA	NA	NA
Cervix-SCC	7dc3e035-c7ae-4164-a62b-9c2460812b05	12	56635002	56637832	6	2830	NA	2	4	NA	NA	NA
Cervix-SCC	7dc3e035-c7ae-4164-a62b-9c2460812b05	20	34346101	34349850	6	3749	2	2	2	NA	NA	NA
Prost-AdenoCA	7dd2dc62-0eb4-4d45-86f1-e9e9377181ca	2	78758356	78759122	7	766	NA	NA	NA	4	3	NA
Prost-AdenoCA	7dd2dc62-0eb4-4d45-86f1-e9e9377181ca	5	110839422	110843644	6	4222	NA	NA	NA	2	4	NA
Prost-AdenoCA	7dd2dc62-0eb4-4d45-86f1-e9e9377181ca	10	52816323	52817840	6	1517	1	5	NA	NA	NA	NA
Prost-AdenoCA	7dd2dc62-0eb4-4d45-86f1-e9e9377181ca	13	90335220	90337043	7	1823	NA	7	NA	NA	NA	NA
Stomach-AdenoCA	7e036d40-b669-4929-a1e2-c4736e01a473	12	125346542	125347811	7	1269	2	2	3	NA	NA	NA
Prost-AdenoCA	7e10e325-16d2-42df-b957-df4d3a9477d0	13	21347299	21348118	6	819	NA	2	4	NA	NA	NA
Prost-AdenoCA	7e10e325-16d2-42df-b957-df4d3a9477d0	13	103364954	103366196	14	1242	NA	7	7	NA	NA	NA
Prost-AdenoCA	7e10e325-16d2-42df-b957-df4d3a9477d0	13	103404532	103407690	10	3158	NA	6	4	NA	NA	NA
Prost-AdenoCA	7e10e325-16d2-42df-b957-df4d3a9477d0	19	22900446	22904614	7	4168	NA	2	5	NA	NA	NA
Head-SCC	7e584ff6-0b81-4f85-81da-b8117afee326	6	10843608	10844732	7	1124	NA	NA	NA	6	1	NA
Head-SCC	7e584ff6-0b81-4f85-81da-b8117afee326	10	38113676	38114056	6	380	2	4	NA	NA	NA	NA
Head-SCC	7e584ff6-0b81-4f85-81da-b8117afee326	11	78554777	78556495	17	1718	1	4	12	NA	NA	NA
Head-SCC	7e584ff6-0b81-4f85-81da-b8117afee326	11	88256460	88260888	10	4428	3	1	6	NA	NA	NA
Head-SCC	7e584ff6-0b81-4f85-81da-b8117afee326	11	88484840	88485356	11	516	1	3	7	NA	NA	NA
Liver-HCC	7eac4710-c622-11e3-bf01-24c6515278c0	7	124345868	124346795	8	927	1	4	3	NA	NA	NA
Liver-HCC	7eac4710-c622-11e3-bf01-24c6515278c0	8	128503844	128504679	11	835	NA	6	5	NA	NA	NA
Stomach-AdenoCA	7eb0f792-858f-45d1-a908-125b02ea1506	2	4938982	4945969	9	6987	NA	NA	NA	3	1	5
Stomach-AdenoCA	7eb0f792-858f-45d1-a908-125b02ea1506	2	6432855	6436281	8	3426	NA	NA	NA	4	3	1
Stomach-AdenoCA	7eb0f792-858f-45d1-a908-125b02ea1506	2	18507777	18508981	10	1204	NA	NA	NA	8	2	NA
Stomach-AdenoCA	7eb0f792-858f-45d1-a908-125b02ea1506	2	80027979	80033074	8	5095	NA	NA	NA	5	1	2
Stomach-AdenoCA	7eb0f792-858f-45d1-a908-125b02ea1506	4	32304715	32309415	9	4700	NA	NA	NA	8	NA	1
Stomach-AdenoCA	7eb0f792-858f-45d1-a908-125b02ea1506	4	44479219	44482870	8	3651	NA	NA	NA	3	4	1
Stomach-AdenoCA	7eb0f792-858f-45d1-a908-125b02ea1506	4	66181705	66188495	9	6790	NA	NA	NA	3	3	3
Stomach-AdenoCA	7eb0f792-858f-45d1-a908-125b02ea1506	4	117702302	117708020	10	5718	NA	NA	1	7	NA	2
Stomach-AdenoCA	7eb0f792-858f-45d1-a908-125b02ea1506	5	5201851	5205000	7	3149	NA	NA	NA	3	3	1
Stomach-AdenoCA	7eb0f792-858f-45d1-a908-125b02ea1506	6	57343241	57345800	10	2559	NA	NA	NA	8	NA	2
Stomach-AdenoCA	7eb0f792-858f-45d1-a908-125b02ea1506	7	119483078	119486505	7	3427	NA	NA	NA	2	4	1
Stomach-AdenoCA	7eb0f792-858f-45d1-a908-125b02ea1506	9	121762167	121766037	9	3870	2	NA	NA	4	2	1

Stomach-AdenoCA	7eb0f792-858f-45d1-a908-125b02ea1506	10	37866256	37869442	6	3186	NA	NA	NA	3	3	NA
Stomach-AdenoCA	7eb0f792-858f-45d1-a908-125b02ea1506	13	65554684	65560013	7	5329	NA	NA	NA	3	2	2
Skin-Melanoma	7edc42d3-d08e-4360-a3e1-aeb57cfc6640	2	193836913	193837292	6	379	2	2	2	NA	NA	NA
Skin-Melanoma	7edc42d3-d08e-4360-a3e1-aeb57cfc6640	6	54920457	54920954	6	497	1	4	1	NA	NA	NA
Skin-Melanoma	7edc42d3-d08e-4360-a3e1-aeb57cfc6640	6	63904548	63904865	7	317	NA	4	3	NA	NA	NA
Skin-Melanoma	7edc42d3-d08e-4360-a3e1-aeb57cfc6640	10	73872130	73877316	10	5186	3	7	NA	NA	NA	NA
Skin-Melanoma	7edc42d3-d08e-4360-a3e1-aeb57cfc6640	11	82657112	82660727	7	3615	NA	3	4	NA	NA	NA
Panc-AdenoCA	7f42e118-aab6-438a-9e84-e7451fe12d7e	3	563969	566065	8	2096	NA	NA	NA	3	2	3
Panc-AdenoCA	7f42e118-aab6-438a-9e84-e7451fe12d7e	3	191953874	191959161	7	5287	NA	NA	NA	4	2	1
Panc-AdenoCA	7f42e118-aab6-438a-9e84-e7451fe12d7e	4	13397228	13398310	6	1082	NA	NA	NA	3	3	NA
Panc-AdenoCA	7f42e118-aab6-438a-9e84-e7451fe12d7e	4	180384481	180391147	11	6666	NA	NA	1	4	4	2
Panc-AdenoCA	7f42e118-aab6-438a-9e84-e7451fe12d7e	4	181057843	181060512	7	2669	NA	3	3	NA	NA	1
Panc-AdenoCA	7f42e118-aab6-438a-9e84-e7451fe12d7e	4	189677587	189678602	9	1015	NA	NA	NA	1	6	2
Panc-AdenoCA	7f42e118-aab6-438a-9e84-e7451fe12d7e	8	97747137	97750665	6	3528	NA	NA	NA	3	2	1
Panc-AdenoCA	7f42e118-aab6-438a-9e84-e7451fe12d7e	10	9624695	9627789	6	3094	1	1	3	NA	NA	1
Panc-AdenoCA	7f42e118-aab6-438a-9e84-e7451fe12d7e	10	57465005	57466806	14	1801	NA	NA	NA	4	3	7
Panc-AdenoCA	7f42e118-aab6-438a-9e84-e7451fe12d7e	11	50280840	50284627	7	3787	NA	NA	NA	2	NA	5
Panc-AdenoCA	7f42e118-aab6-438a-9e84-e7451fe12d7e	13	69602193	69607961	11	5768	NA	NA	NA	3	5	3
Panc-AdenoCA	7f42e118-aab6-438a-9e84-e7451fe12d7e	18	21857204	21857524	6	320	NA	4	2	NA	NA	NA
Liver-HCC	7f795952-c623-11e3-bf01-24c6515278c0	1	238677674	238681152	7	3478	NA	3	3	1	NA	NA
Panc-AdenoCA	7fcd8af9-4d73-4c97-95cc-b30b09f21bb2	18	18794649	18794737	7	88	2	NA	5	NA	NA	NA
Ovary-AdenoCA	7fdd07a4-4a27-40c3-af92-a0074e6391f5	5	468806	472722	8	3916	1	5	2	NA	NA	NA
Ovary-AdenoCA	7fdd07a4-4a27-40c3-af92-a0074e6391f5	19	12634668	12639311	30	4643	2	15	12	NA	NA	1
Cervix-SCC	801e67c8-c506-410a-bdf0-856a66e974f4	6	34711319	34714913	8	3594	1	2	5	NA	NA	NA
Cervix-SCC	801e67c8-c506-410a-bdf0-856a66e974f4	8	145621491	145623107	6	1616	1	2	3	NA	NA	NA
Cervix-SCC	801e67c8-c506-410a-bdf0-856a66e974f4	9	85623801	85627423	6	3622	1	2	3	NA	NA	NA
Cervix-SCC	801e67c8-c506-410a-bdf0-856a66e974f4	11	67970260	67972325	7	2065	NA	2	5	NA	NA	NA
Cervix-SCC	801e67c8-c506-410a-bdf0-856a66e974f4	12	55021973	55028395	10	6422	1	6	2	NA	NA	1
Cervix-SCC	801e67c8-c506-410a-bdf0-856a66e974f4	13	102771382	102777779	8	6397	NA	NA	8	NA	NA	NA
Cervix-SCC	801e67c8-c506-410a-bdf0-856a66e974f4	14	103819265	103822916	6	3651	NA	2	4	NA	NA	NA
Cervix-SCC	801e67c8-c506-410a-bdf0-856a66e974f4	22	24503311	24507242	6	3931	NA	2	4	NA	NA	NA
Bladder-TCC	804ffa2e-158b-447d-945c-707684134c87	7	141778613	141780215	9	1602	NA	1	8	NA	NA	NA
Bladder-TCC	804ffa2e-158b-447d-945c-707684134c87	8	63209370	63214907	12	5537	1	1	10	NA	NA	NA
Bladder-TCC	804ffa2e-158b-447d-945c-707684134c87	12	120182179	120190309	11	8130	NA	3	8	NA	NA	NA
Bladder-TCC	804ffa2e-158b-447d-945c-707684134c87	15	39679807	39680616	6	809	NA	3	3	NA	NA	NA
Bladder-TCC	804ffa2e-158b-447d-945c-707684134c87	16	4759550	4765524	7	5974	NA	4	3	NA	NA	NA
Bladder-TCC	804ffa2e-158b-447d-945c-707684134c87	19	11319856	11321351	6	1495	NA	2	4	NA	NA	NA
Bladder-TCC	804ffa2e-158b-447d-945c-707684134c87	20	20993677	20997278	14	3601	2	2	10	NA	NA	NA
Ovary-AdenoCA	8093649a-74d6-4832-9154-8cc0182fa381	11	75049530	75050424	6	894	2	2	2	NA	NA	NA
Ovary-AdenoCA	8093649a-74d6-4832-9154-8cc0182fa381	11	83781423	83782837	10	1414	3	4	3	NA	NA	NA
Ovary-AdenoCA	8093649a-74d6-4832-9154-8cc0182fa381	11	84470514	84471323	7	809	3	2	2	NA	NA	NA
Ovary-AdenoCA	8093649a-74d6-4832-9154-8cc0182fa381	11	97483082	97488741	7	5659	1	NA	6	NA	NA	NA
Ovary-AdenoCA	8093649a-74d6-4832-9154-8cc0182fa381	11	100228042	100230589	7	2547	3	1	3	NA	NA	NA
Ovary-AdenoCA	8093649a-74d6-4832-9154-8cc0182fa381	11	100264372	100270540	8	6168	NA	5	3	NA	NA	NA
Ovary-AdenoCA	8093649a-74d6-4832-9154-8cc0182fa381	11	100690661	100697507	12	6846	2	3	7	NA	NA	NA
Ovary-AdenoCA	8093649a-74d6-4832-9154-8cc0182fa381	11	103615129	103618462	11	3333	2	5	4	NA	NA	NA
Ovary-AdenoCA	8093649a-74d6-4832-9154-8cc0182fa381	11	104087982	104094089	10	6107	2	2	6	NA	NA	NA
Ovary-AdenoCA	8093649a-74d6-4832-9154-8cc0182fa381	11	104407299	104412966	7	5667	1	3	3	NA	NA	NA
Ovary-AdenoCA	8093649a-74d6-4832-9154-8cc0182fa381	11	104429333	104433409	7	4076	1	2	4	NA	NA	NA
Ovary-AdenoCA	8093649a-74d6-4832-9154-8cc0182fa381	11	115111691	115115924	10	4233	6	3	1	NA	NA	NA
Ovary-AdenoCA	8093649a-74d6-4832-9154-8cc0182fa381	11	115882866	115891993	14	9127	6	6	2	NA	NA	NA
Ovary-AdenoCA	8093649a-74d6-4832-9154-8cc0182fa381	11	120942034	120946341	7	4307	1	3	3	NA	NA	NA
Ovary-AdenoCA	8093649a-74d6-4832-9154-8cc0182fa381	11	121547032	121551863	9	4831	5	3	1	NA	NA	NA
Ovary-AdenoCA	8093649a-74d6-4832-9154-8cc0182fa381	11	123403172	123408306	7	5134	3	3	1	NA	NA	NA
Ovary-AdenoCA	8093649a-74d6-4832-9154-8cc0182fa381	11	124316996	124322763	10	5767	1	3	5	NA	NA	1
Ovary-AdenoCA	8093649a-74d6-4832-9154-8cc0182fa381	11	127041291	127046827	7	5536	4	2	1	NA	NA	NA
Ovary-AdenoCA	8093649a-74d6-4832-9154-8cc0182fa381	11	128406178	128411205	7	5027	NA	5	2	NA	NA	NA
Ovary-AdenoCA	8093649a-74d6-4832-9154-8cc0182fa381	11	131095849	131104915	11	9066	NA	6	5	NA	NA	NA
Liver-HCC	80ebcecc-4a7a-40dd-914c-f0648a58095f	8	40173831	40174378	7	547	NA	3	3	NA	NA	1
Liver-HCC	80ebcecc-4a7a-40dd-914c-f0648a58095f	19	19808731	19810348	8	1617	NA	3	5	NA	NA	NA

Head-SCC	81598359-24d5-4a8b-a6e6-7a6f94289119	14	82137912	82138342	6	430	NA	2	4	NA	NA	NA
Liver-HCC	819b4304-c622-11e3-bf01-24c6515278c0	8	4554147	4558315	6	4168	NA	NA	NA	2	2	2
Liver-HCC	81a8b064-e735-455f-b2db-af7ae11daac4	7	114347482	114350548	12	3066	NA	7	5	NA	NA	NA
ColoRect-AdenoCA	81b1e78c-6032-4ff4-b52a-83456b9450ea	6	73998030	73998971	6	941	NA	1	5	NA	NA	NA
Lymph-BNHL	81b4dd02-34ec-4ddc-8818-ed888a60ad88	14	106238664	106241179	17	2515	1	5	10	1	NA	NA
Lymph-BNHL	81b4dd02-34ec-4ddc-8818-ed888a60ad88	14	106324967	106329161	28	4194	1	9	5	8	NA	5
Lymph-BNHL	81b4dd02-34ec-4ddc-8818-ed888a60ad88	18	60984862	60988194	14	3332	2	5	2	3	1	1
Lymph-BNHL	81b4dd02-34ec-4ddc-8818-ed888a60ad88	22	22749427	22749615	6	188	NA	4	1	NA	NA	1
Lymph-BNHL	81b4dd02-34ec-4ddc-8818-ed888a60ad88	22	23227765	23231798	13	4033	2	1	5	3	1	1
Panc-AdenoCA	81bc7f0c-865d-4801-a935-2ab04170df53	5	22873863	22879583	14	5720	1	6	7	NA	NA	NA
Panc-AdenoCA	81bc7f0c-865d-4801-a935-2ab04170df53	10	10811786	10811795	7	9	NA	NA	NA	4	1	2
Lymph-BNHL	81cc0f39-6677-4f2e-9a75-d30152b188f3	2	142732152	142736850	6	4698	NA	NA	NA	2	2	2
Lymph-BNHL	81cc0f39-6677-4f2e-9a75-d30152b188f3	3	187461042	187463289	13	2247	NA	1	7	4	1	NA
Lymph-BNHL	81cc0f39-6677-4f2e-9a75-d30152b188f3	8	116337541	116345567	10	8026	NA	NA	1	1	4	4
Lymph-BNHL	81cc0f39-6677-4f2e-9a75-d30152b188f3	10	58233924	58238432	6	4508	NA	NA	1	3	1	1
Lymph-BNHL	81cc0f39-6677-4f2e-9a75-d30152b188f3	12	112973735	112974028	6	293	NA	NA	6	NA	NA	NA
Lymph-BNHL	81cc0f39-6677-4f2e-9a75-d30152b188f3	12	122458906	122463307	11	4401	2	1	8	NA	NA	NA
Lymph-BNHL	81cc0f39-6677-4f2e-9a75-d30152b188f3	14	106068739	106071254	17	2515	2	5	10	NA	NA	NA
Lymph-BNHL	81cc0f39-6677-4f2e-9a75-d30152b188f3	14	106112651	106114367	18	1716	2	8	8	NA	NA	NA
Lymph-BNHL	81cc0f39-6677-4f2e-9a75-d30152b188f3	14	106321993	106330138	58	8145	6	8	26	8	7	3
Lymph-BNHL	81cc0f39-6677-4f2e-9a75-d30152b188f3	14	106725333	106726169	14	836	1	NA	3	4	3	3
Lymph-BNHL	81cc0f39-6677-4f2e-9a75-d30152b188f3	15	86225855	86226112	8	257	1	4	2	1	NA	NA
Lymph-BNHL	81cc0f39-6677-4f2e-9a75-d30152b188f3	16	10971672	10974173	14	2501	NA	4	10	NA	NA	NA
Lymph-BNHL	81cc0f39-6677-4f2e-9a75-d30152b188f3	18	60804791	60809720	20	4929	NA	1	7	7	4	1
Lymph-BNHL	81cc0f39-6677-4f2e-9a75-d30152b188f3	18	60873259	60875272	8	2013	2	1	NA	3	2	NA
Lymph-BNHL	81cc0f39-6677-4f2e-9a75-d30152b188f3	18	60983837	60988772	76	4935	4	9	32	23	3	5
Lymph-BNHL	81cc0f39-6677-4f2e-9a75-d30152b188f3	22	23028853	23055316	63	26463	3	9	31	12	4	4
Lymph-BNHL	81cc0f39-6677-4f2e-9a75-d30152b188f3	22	23242054	23244238	9	2184	NA	6	1	NA	1	1
Lymph-BNHL	81cc0f39-6677-4f2e-9a75-d30152b188f3	22	23278583	23283127	7	4544	NA	1	3	2	1	NA
Panc-AdenoCA	8282283d-247a-431d-9421-0fcc52f0a897	3	37430664	37431180	10	516	1	8	1	NA	NA	NA
Panc-AdenoCA	8282283d-247a-431d-9421-0fcc52f0a897	10	46074675	46076859	6	2184	1	4	1	NA	NA	NA
Head-SCC	8294a969-59a1-4d3b-895b-f07e96bf9f7a	19	23732298	23733941	8	1643	1	5	1	NA	NA	1
Lymph-BNHL	82b8cda8-fbff-455e-b0db-7ff6528bd6c8	2	89157325	89164756	16	7431	1	2	11	NA	1	1
Lymph-BNHL	82b8cda8-fbff-455e-b0db-7ff6528bd6c8	14	106110529	106114759	9	4230	NA	1	8	NA	NA	NA
Lymph-BNHL	82b8cda8-fbff-455e-b0db-7ff6528bd6c8	14	106322881	106330585	26	7704	NA	NA	22	2	NA	2
Lymph-BNHL	82b8cda8-fbff-455e-b0db-7ff6528bd6c8	22	23230138	23234777	9	4639	NA	1	4	2	NA	2
CNS-GBM	82d7fa2d-19cd-41b7-8ed1-ea9fb9a544c6	9	28619306	28619632	12	326	NA	NA	12	NA	NA	NA
Liver-HCC	83d57c5c-c622-11e3-bf01-24c6515278c0	3	86581565	86582340	6	775	NA	NA	NA	3	3	NA
Head-SCC	83eca7b5-45bb-44c0-8afe-36f8988d0078	3	110983817	110989070	13	5253	4	3	6	NA	NA	NA
Head-SCC	83eca7b5-45bb-44c0-8afe-36f8988d0078	3	193515535	193517494	7	1959	NA	6	1	NA	NA	NA
Head-SCC	83eca7b5-45bb-44c0-8afe-36f8988d0078	4	46230296	46230803	8	507	2	3	3	NA	NA	NA
Head-SCC	83eca7b5-45bb-44c0-8afe-36f8988d0078	5	3859443	3875510	18	16067	1	12	5	NA	NA	NA
Head-SCC	83eca7b5-45bb-44c0-8afe-36f8988d0078	5	93482414	93486383	7	3969	1	2	4	NA	NA	NA
Head-SCC	83eca7b5-45bb-44c0-8afe-36f8988d0078	8	25668439	25675148	10	6709	1	3	6	NA	NA	NA
Head-SCC	83eca7b5-45bb-44c0-8afe-36f8988d0078	14	68663989	68668330	8	4341	2	2	4	NA	NA	NA
Head-SCC	83eca7b5-45bb-44c0-8afe-36f8988d0078	18	6143554	6148113	10	4559	5	1	4	NA	NA	NA
Panc-AdenoCA	8454fe53-869d-41c8-b0c8-a7929d00eec3	6	18938579	18940967	11	2388	1	2	8	NA	NA	NA
Panc-AdenoCA	8454fe53-869d-41c8-b0c8-a7929d00eec3	18	20510481	20512063	9	1582	1	4	4	NA	NA	NA
Panc-AdenoCA	8454fe53-869d-41c8-b0c8-a7929d00eec3	19	13197362	13200045	7	2683	1	4	2	NA	NA	NA
Panc-AdenoCA	84a6ea88-eeb8-4060-a90c-e4abac013ad4	3	185343121	185343626	6	505	NA	3	3	NA	NA	NA
Panc-AdenoCA	84a6ea88-eeb8-4060-a90c-e4abac013ad4	3	185350745	185351477	10	732	2	4	4	NA	NA	NA
Breast-AdenoCa	84c77098-03d0-4b22-afb1-797703e85c6c	1	15530506	15530752	6	246	3	1	2	NA	NA	NA
Breast-AdenoCa	84c77098-03d0-4b22-afb1-797703e85c6c	1	16643565	16643905	7	340	1	2	4	NA	NA	NA
Breast-AdenoCa	84c77098-03d0-4b22-afb1-797703e85c6c	1	76345348	76348969	7	3621	3	1	3	NA	NA	NA
Breast-AdenoCa	84c77098-03d0-4b22-afb1-797703e85c6c	1	107359332	107360496	12	1164	2	5	4	1	NA	NA
Breast-AdenoCa	84c77098-03d0-4b22-afb1-797703e85c6c	2	25903390	25904546	10	1156	1	6	2	NA	NA	1
Breast-AdenoCa	84c77098-03d0-4b22-afb1-797703e85c6c	2	206040099	206047901	26	7802	5	8	13	NA	NA	NA
Breast-AdenoCa	84c77098-03d0-4b22-afb1-797703e85c6c	2	206152841	206163518	15	10677	1	8	6	NA	NA	NA
Breast-AdenoCa	84c77098-03d0-4b22-afb1-797703e85c6c	2	207765929	207769783	26	3854	3	5	18	NA	NA	NA
Breast-AdenoCa	84c77098-03d0-4b22-afb1-797703e85c6c	3	124980879	124983845	11	2966	1	7	3	NA	NA	NA

Breast-AdenoCa	84c77098-03d0-4b22-afb1-797703e85c6c	11	95897979	95900050	6	2071	NA	4	2	NA	NA	NA
Breast-AdenoCa	84c77098-03d0-4b22-afb1-797703e85c6c	12	72247592	72251085	7	3493	NA	3	4	NA	NA	NA
Breast-AdenoCa	84c77098-03d0-4b22-afb1-797703e85c6c	15	68109294	68111678	6	2384	1	4	1	NA	NA	NA
Breast-AdenoCa	84c77098-03d0-4b22-afb1-797703e85c6c	22	19005334	19007398	7	2064	1	4	2	NA	NA	NA
Breast-AdenoCa	84c77098-03d0-4b22-afb1-797703e85c6c	22	25364793	25367930	9	3137	2	2	5	NA	NA	NA
Ovary-AdenoCA	84ca6ab0-9edc-4636-9d27-55cdba334d7d	13	32063380	32076502	20	13122	3	7	10	NA	NA	NA
Ovary-AdenoCA	84ca6ab0-9edc-4636-9d27-55cdba334d7d	19	35823957	35834622	12	10665	2	4	6	NA	NA	NA
Ovary-AdenoCA	84ca6ab0-9edc-4636-9d27-55cdba334d7d	X	36475616	36477925	9	2309	2	2	4	NA	NA	1
Liver-HCC	84fe77b4-f6cc-49bf-a6b9-1621ec9394f8	17	60251125	60251239	7	114	NA	1	2	3	1	NA
Lymph-BNHL	858631eb-4e91-4aad-809c-c3948519313d	2	89136300	89146182	13	9882	NA	1	3	6	1	2
Lymph-BNHL	858631eb-4e91-4aad-809c-c3948519313d	2	89153907	89196860	205	42953	11	31	41	62	29	31
Lymph-BNHL	858631eb-4e91-4aad-809c-c3948519313d	2	89442405	89448022	25	5617	NA	4	6	7	1	7
Lymph-BNHL	858631eb-4e91-4aad-809c-c3948519313d	2	120730255	120730366	7	111	NA	NA	1	1	3	2
Lymph-BNHL	858631eb-4e91-4aad-809c-c3948519313d	2	136874765	136875264	16	499	NA	5	4	3	3	1
Lymph-BNHL	858631eb-4e91-4aad-809c-c3948519313d	3	76935133	76938210	7	3077	NA	NA	1	1	1	4
Lymph-BNHL	858631eb-4e91-4aad-809c-c3948519313d	3	186782908	186784219	11	1311	NA	2	5	1	1	2
Lymph-BNHL	858631eb-4e91-4aad-809c-c3948519313d	3	187457059	187463261	50	6202	4	17	9	10	7	3
Lymph-BNHL	858631eb-4e91-4aad-809c-c3948519313d	4	40195270	40201106	14	5836	NA	2	3	3	3	3
Lymph-BNHL	858631eb-4e91-4aad-809c-c3948519313d	5	124043574	124044543	10	969	NA	1	2	4	1	2
Lymph-BNHL	858631eb-4e91-4aad-809c-c3948519313d	5	124078408	124081153	8	2745	NA	1	NA	5	1	1
Lymph-BNHL	858631eb-4e91-4aad-809c-c3948519313d	6	14118275	14118949	11	674	1	1	8	NA	NA	1
Lymph-BNHL	858631eb-4e91-4aad-809c-c3948519313d	6	134493913	134498122	10	4209	NA	5	4	1	NA	NA
Lymph-BNHL	858631eb-4e91-4aad-809c-c3948519313d	7	83175404	83187996	14	12592	1	NA	NA	5	3	5
Lymph-BNHL	858631eb-4e91-4aad-809c-c3948519313d	9	37032927	37035225	8	2298	NA	2	3	1	2	NA
Lymph-BNHL	858631eb-4e91-4aad-809c-c3948519313d	11	40376717	40382683	8	5966	1	1	1	1	2	2
Lymph-BNHL	858631eb-4e91-4aad-809c-c3948519313d	11	102188485	102189225	9	740	NA	3	5	NA	NA	1
Lymph-BNHL	858631eb-4e91-4aad-809c-c3948519313d	12	122457884	122463053	17	5169	3	2	5	3	1	3
Lymph-BNHL	858631eb-4e91-4aad-809c-c3948519313d	14	69259050	69262445	6	3395	NA	1	4	NA	1	NA
Lymph-BNHL	858631eb-4e91-4aad-809c-c3948519313d	14	106240130	106244437	9	4307	1	1	5	2	NA	NA
Lymph-BNHL	858631eb-4e91-4aad-809c-c3948519313d	14	106325206	106330704	111	5498	5	28	38	12	9	19
Lymph-BNHL	858631eb-4e91-4aad-809c-c3948519313d	14	107049081	107049802	28	721	2	6	6	5	4	5
Lymph-BNHL	858631eb-4e91-4aad-809c-c3948519313d	14	107169791	107179678	19	9887	1	3	5	4	4	2
Lymph-BNHL	858631eb-4e91-4aad-809c-c3948519313d	16	78751251	78756120	7	4869	NA	NA	NA	2	4	1
Lymph-BNHL	858631eb-4e91-4aad-809c-c3948519313d	18	60984043	60988349	56	4306	3	10	13	16	5	9
Lymph-BNHL	858631eb-4e91-4aad-809c-c3948519313d	22	23223297	23281471	127	58174	4	17	45	31	9	21
Lymph-BNHL	858631eb-4e91-4aad-809c-c3948519313d	X	27931593	27936351	11	4758	NA	1	NA	5	1	4
ColoRect-AdenoCA	85e29049-a5e8-49fa-a576-e113c7b2c2e3	11	55400610	55401289	6	679	1	4	1	NA	NA	NA
ColoRect-AdenoCA	85e29049-a5e8-49fa-a576-e113c7b2c2e3	11	123104022	123104895	7	873	NA	4	2	1	NA	NA
Kidney-ChRCC	85ed2434-da94-4333-90ca-6ca4691cbdd3	9	2430302	2435395	7	5093	NA	3	4	NA	NA	NA
Ovary-AdenoCA	8658f4f5-9a50-4195-8ea3-227951977647	3	122831206	122835063	13	3857	6	2	5	NA	NA	NA
Ovary-AdenoCA	8658f4f5-9a50-4195-8ea3-227951977647	11	73695595	73695807	9	212	NA	NA	NA	9	NA	NA
Lymph-BNHL	866ecfe7-caa6-4565-9418-6b9d6c8a3b43	14	106068967	106069949	6	982	NA	NA	6	NA	NA	NA
Lymph-BNHL	866ecfe7-caa6-4565-9418-6b9d6c8a3b43	14	106212768	106214196	24	1428	5	3	13	3	NA	NA
Lymph-BNHL	866ecfe7-caa6-4565-9418-6b9d6c8a3b43	14	106238352	106241524	22	3172	1	4	16	1	NA	NA
Lymph-BNHL	866ecfe7-caa6-4565-9418-6b9d6c8a3b43	14	106323787	106330121	57	6334	4	12	29	4	4	4
Lymph-BNHL	866ecfe7-caa6-4565-9418-6b9d6c8a3b43	14	106994149	106994893	11	744	1	NA	2	4	1	3
Lymph-BNHL	866ecfe7-caa6-4565-9418-6b9d6c8a3b43	18	60984697	60988858	35	4161	1	3	9	9	9	4
Lymph-BNHL	866ecfe7-caa6-4565-9418-6b9d6c8a3b43	22	22676963	22677443	7	480	1	2	3	1	NA	NA
Lymph-BNHL	866ecfe7-caa6-4565-9418-6b9d6c8a3b43	22	22697598	22697757	7	159	2	NA	2	NA	3	NA
Lymph-BNHL	866ecfe7-caa6-4565-9418-6b9d6c8a3b43	22	23260393	23295546	89	35153	4	20	32	19	11	3
Prost-AdenoCA	86874a67-aa86-45f1-be32-3b2722ebedec	5	42294241	42295040	12	799	NA	2	10	NA	NA	NA
Prost-AdenoCA	86874a67-aa86-45f1-be32-3b2722ebedec	5	106855787	106856362	9	575	NA	2	7	NA	NA	NA
Prost-AdenoCA	8689b53c-8883-479c-85f6-d13b86272b5d	4	59515125	59517064	6	1939	NA	2	4	NA	NA	NA
Prost-AdenoCA	8689b53c-8883-479c-85f6-d13b86272b5d	7	48086666	48087227	6	561	NA	NA	6	NA	NA	NA
Breast-AdenoCa	8691c87f-2468-44bb-a670-3dccd6da33c6	3	74461716	74464162	8	2446	2	3	3	NA	NA	NA
Breast-AdenoCa	8691c87f-2468-44bb-a670-3dccd6da33c6	4	191031466	191036698	12	5232	NA	7	5	NA	NA	NA
Breast-AdenoCa	8691c87f-2468-44bb-a670-3dccd6da33c6	5	152027954	152028740	7	786	2	4	1	NA	NA	NA
Breast-AdenoCa	8691c87f-2468-44bb-a670-3dccd6da33c6	6	155418119	155421420	7	3301	NA	1	6	NA	NA	NA
Breast-AdenoCa	8691c87f-2468-44bb-a670-3dccd6da33c6	8	43552478	43567610	29	15132	3	5	21	NA	NA	NA
Breast-AdenoCa	8691c87f-2468-44bb-a670-3dccd6da33c6	10	37645202	37650452	7	5250	1	3	3	NA	NA	NA

Breast-AdenoCa	8691c87f-2468-44bb-a670-3dccd6da33c6	17	36513792	36514825	12	1033	2	6	4	NA	NA	NA
Breast-AdenoCa	8691c87f-2468-44bb-a670-3dccd6da33c6	18	35186849	35192210	8	5361	2	1	5	NA	NA	NA
Breast-AdenoCa	8691c87f-2468-44bb-a670-3dccd6da33c6	19	11463007	11466478	6	3471	NA	2	4	NA	NA	NA
Breast-AdenoCa	8691c87f-2468-44bb-a670-3dccd6da33c6	19	11471456	11476245	7	4789	NA	5	2	NA	NA	NA
Breast-AdenoCa	8691c87f-2468-44bb-a670-3dccd6da33c6	20	25303457	25304165	10	708	1	5	4	NA	NA	NA
Breast-AdenoCa	8691c87f-2468-44bb-a670-3dccd6da33c6	22	23393774	23394463	11	689	2	5	3	NA	NA	1
Ovary-AdenoCA	86f23897-dba0-4e89-8381-d174eaa6fcc1	3	99185009	99187297	8	2288	1	6	1	NA	NA	NA
Ovary-AdenoCA	86f23897-dba0-4e89-8381-d174eaa6fcc1	4	13766385	13780227	20	13842	2	11	7	NA	NA	NA
Ovary-AdenoCA	86f23897-dba0-4e89-8381-d174eaa6fcc1	4	85255984	85260349	11	4365	1	4	6	NA	NA	NA
Ovary-AdenoCA	86f23897-dba0-4e89-8381-d174eaa6fcc1	4	148499091	148512539	17	13448	4	8	5	NA	NA	NA
Ovary-AdenoCA	86f23897-dba0-4e89-8381-d174eaa6fcc1	5	60560930	60571650	20	10720	5	11	4	NA	NA	NA
Ovary-AdenoCA	86f23897-dba0-4e89-8381-d174eaa6fcc1	6	27269039	27276375	13	7336	3	9	1	NA	NA	NA
Ovary-AdenoCA	86f23897-dba0-4e89-8381-d174eaa6fcc1	7	552087	554233	6	2146	1	4	1	NA	NA	NA
Ovary-AdenoCA	86f23897-dba0-4e89-8381-d174eaa6fcc1	8	36956079	36956352	6	273	NA	4	2	NA	NA	NA
Ovary-AdenoCA	86f23897-dba0-4e89-8381-d174eaa6fcc1	16	47977955	47983287	7	5332	3	NA	4	NA	NA	NA
Ovary-AdenoCA	86f23897-dba0-4e89-8381-d174eaa6fcc1	18	23582963	23584291	15	1328	5	8	2	NA	NA	NA
Liver-HCC	87671312-c622-11e3-bf01-24c6515278c0	7	21266797	21268230	14	1433	3	4	7	NA	NA	NA
Head-SCC	878a7fe7-20ff-4651-9587-b4d6fd42e929	8	39368225	39368825	6	600	NA	6	NA	NA	NA	NA
Head-SCC	878a7fe7-20ff-4651-9587-b4d6fd42e929	10	100218324	100221600	37	3276	1	13	23	NA	NA	NA
Head-SCC	878a7fe7-20ff-4651-9587-b4d6fd42e929	18	64339335	64339750	6	415	NA	2	4	NA	NA	NA
Stomach-AdenoCA	8867bdef-3a8d-433e-b10f-d752e2cbb022	10	100085639	100093041	11	7402	3	4	4	NA	NA	NA
Stomach-AdenoCA	8867bdef-3a8d-433e-b10f-d752e2cbb022	13	105854897	105861270	9	6373	1	NA	NA	6	1	1
Stomach-AdenoCA	8867bdef-3a8d-433e-b10f-d752e2cbb022	X	112855891	112862540	9	6649	NA	NA	NA	5	1	3
Prost-AdenoCA	887616c5-06a7-4e83-948c-3546202349fb	3	90420160	90425758	9	5598	1	NA	3	3	2	NA
Prost-AdenoCA	887616c5-06a7-4e83-948c-3546202349fb	4	98339624	98344237	8	4613	NA	NA	2	5	NA	1
Prost-AdenoCA	887616c5-06a7-4e83-948c-3546202349fb	11	56222604	56227532	6	4928	1	1	1	1	2	NA
Prost-AdenoCA	887616c5-06a7-4e83-948c-3546202349fb	X	66195364	66197224	7	1860	2	1	3	NA	1	NA
Ovary-AdenoCA	8888e808-594b-4c76-b2e4-62aa56736f7c	12	90731586	90733935	25	2349	NA	14	11	NA	NA	NA
Panc-AdenoCA	88d5a8b2-daba-45ce-90bf-480fac85bf1d	12	28908883	28909886	13	1003	NA	4	9	NA	NA	NA
Lymph-BNHL	890e840c-1d1d-4874-a8eb-f9d9a2b50a1c	1	163980386	163982076	8	1690	NA	NA	NA	4	2	2
Lymph-BNHL	890e840c-1d1d-4874-a8eb-f9d9a2b50a1c	1	172148405	172153396	25	4991	2	1	3	15	NA	4
Lymph-BNHL	890e840c-1d1d-4874-a8eb-f9d9a2b50a1c	2	58250161	58251919	9	1758	1	1	NA	5	NA	2
Lymph-BNHL	890e840c-1d1d-4874-a8eb-f9d9a2b50a1c	2	89127828	89128862	7	1034	2	2	NA	NA	2	1
Lymph-BNHL	890e840c-1d1d-4874-a8eb-f9d9a2b50a1c	2	89137621	89155570	20	17949	2	1	2	5	8	2
Lymph-BNHL	890e840c-1d1d-4874-a8eb-f9d9a2b50a1c	2	89159103	89196835	142	37732	9	19	17	45	27	25
Lymph-BNHL	890e840c-1d1d-4874-a8eb-f9d9a2b50a1c	2	136875225	136875487	11	262	NA	1	4	3	1	2
Lymph-BNHL	890e840c-1d1d-4874-a8eb-f9d9a2b50a1c	2	205536186	205539621	10	3435	NA	NA	2	4	3	1
Lymph-BNHL	890e840c-1d1d-4874-a8eb-f9d9a2b50a1c	3	117117428	117122359	7	4931	1	1	NA	2	1	2
Lymph-BNHL	890e840c-1d1d-4874-a8eb-f9d9a2b50a1c	3	163660853	163668548	12	7695	1	1	1	5	1	3
Lymph-BNHL	890e840c-1d1d-4874-a8eb-f9d9a2b50a1c	3	187462600	187462946	7	346	NA	1	3	3	NA	NA
Lymph-BNHL	890e840c-1d1d-4874-a8eb-f9d9a2b50a1c	4	62976892	62980326	6	3434	NA	NA	1	1	NA	4
Lymph-BNHL	890e840c-1d1d-4874-a8eb-f9d9a2b50a1c	5	19515294	19517245	7	1951	1	NA	2	1	NA	3
Lymph-BNHL	890e840c-1d1d-4874-a8eb-f9d9a2b50a1c	5	102166564	102171284	8	4720	1	1	2	1	1	2
Lymph-BNHL	890e840c-1d1d-4874-a8eb-f9d9a2b50a1c	6	63012119	63014138	6	2019	NA	NA	NA	3	2	1
Lymph-BNHL	890e840c-1d1d-4874-a8eb-f9d9a2b50a1c	9	113376051	113378471	7	2420	NA	NA	NA	4	1	2
Lymph-BNHL	890e840c-1d1d-4874-a8eb-f9d9a2b50a1c	11	109295946	109299095	8	3149	NA	1	NA	2	3	2
Lymph-BNHL	890e840c-1d1d-4874-a8eb-f9d9a2b50a1c	12	25251244	25259988	24	8744	NA	NA	NA	9	5	10
Lymph-BNHL	890e840c-1d1d-4874-a8eb-f9d9a2b50a1c	12	33757237	33764583	11	7346	3	NA	2	4	NA	2
Lymph-BNHL	890e840c-1d1d-4874-a8eb-f9d9a2b50a1c	12	122458508	122462064	12	3556	NA	5	3	1	2	1
Lymph-BNHL	890e840c-1d1d-4874-a8eb-f9d9a2b50a1c	13	30266200	30270310	7	4110	1	5	1	NA	NA	NA
Lymph-BNHL	890e840c-1d1d-4874-a8eb-f9d9a2b50a1c	14	106039977	106044243	10	4266	NA	3	1	2	3	1
Lymph-BNHL	890e840c-1d1d-4874-a8eb-f9d9a2b50a1c	14	106112255	106113420	7	1165	NA	3	4	NA	NA	NA
Lymph-BNHL	890e840c-1d1d-4874-a8eb-f9d9a2b50a1c	14	106326500	106329842	53	3342	4	15	15	6	7	6
Lymph-BNHL	890e840c-1d1d-4874-a8eb-f9d9a2b50a1c	14	106692133	106692945	15	812	NA	1	7	5	NA	2
Lymph-BNHL	890e840c-1d1d-4874-a8eb-f9d9a2b50a1c	17	29641068	29641197	6	129	NA	NA	NA	4	NA	2
Lymph-BNHL	890e840c-1d1d-4874-a8eb-f9d9a2b50a1c	18	27957535	27964421	8	6886	NA	NA	1	4	NA	3
Lymph-BNHL	890e840c-1d1d-4874-a8eb-f9d9a2b50a1c	18	60794650	60795751	8	1101	NA	2	2	2	NA	2
Lymph-BNHL	890e840c-1d1d-4874-a8eb-f9d9a2b50a1c	18	60984760	60988537	68	3777	3	14	21	17	6	7
Lymph-BNHL	890e840c-1d1d-4874-a8eb-f9d9a2b50a1c	22	22707580	22712196	9	4616	NA	2	3	4	NA	NA
Lymph-BNHL	890e840c-1d1d-4874-a8eb-f9d9a2b50a1c	22	23222368	23293310	75	70942	5	9	25	18	11	7

Lymph-BNHL	890e840c-1d1d-4874-a8eb-f9d9a2b50a1c	22	46182540	46190173	17	7633	NA	NA	2	5	3	7
Lymph-BNHL	890e840c-1d1d-4874-a8eb-f9d9a2b50a1c	X	4322805	4324526	6	1721	NA	1	NA	3	2	NA
Panc-AdenoCA	89195d56-1ef2-41df-b200-9f18e38f574a	1	145033530	145035453	8	1923	NA	NA	8	NA	NA	NA
Panc-AdenoCA	89195d56-1ef2-41df-b200-9f18e38f574a	1	145045076	145046355	11	1279	NA	6	5	NA	NA	NA
Panc-AdenoCA	89195d56-1ef2-41df-b200-9f18e38f574a	1	152695117	152700365	8	5248	NA	1	6	NA	1	NA
Panc-AdenoCA	89195d56-1ef2-41df-b200-9f18e38f574a	1	242822102	242827601	14	5499	NA	3	10	NA	NA	1
Panc-AdenoCA	89195d56-1ef2-41df-b200-9f18e38f574a	1	246082447	246082623	8	176	NA	5	3	NA	NA	NA
Panc-AdenoCA	89195d56-1ef2-41df-b200-9f18e38f574a	2	137643527	137644244	11	717	NA	NA	11	NA	NA	NA
Panc-AdenoCA	89195d56-1ef2-41df-b200-9f18e38f574a	2	137724600	137729558	27	4958	1	4	22	NA	NA	NA
Panc-AdenoCA	89195d56-1ef2-41df-b200-9f18e38f574a	2	138497049	138501494	18	4445	NA	NA	18	NA	NA	NA
Panc-AdenoCA	89195d56-1ef2-41df-b200-9f18e38f574a	2	140332286	140335729	8	3443	1	2	5	NA	NA	NA
Panc-AdenoCA	89195d56-1ef2-41df-b200-9f18e38f574a	2	140680670	140682144	8	1474	NA	NA	8	NA	NA	NA
Panc-AdenoCA	89195d56-1ef2-41df-b200-9f18e38f574a	2	172986453	172989949	8	3496	2	2	4	NA	NA	NA
Panc-AdenoCA	89195d56-1ef2-41df-b200-9f18e38f574a	3	157365137	157367068	8	1931	NA	NA	7	NA	1	NA
Panc-AdenoCA	89195d56-1ef2-41df-b200-9f18e38f574a	3	158614113	158615515	10	1402	NA	NA	10	NA	NA	NA
Panc-AdenoCA	89195d56-1ef2-41df-b200-9f18e38f574a	3	184647489	184650785	16	3296	NA	3	13	NA	NA	NA
Panc-AdenoCA	89195d56-1ef2-41df-b200-9f18e38f574a	4	30903152	30903636	8	484	2	5	1	NA	NA	NA
Panc-AdenoCA	89195d56-1ef2-41df-b200-9f18e38f574a	4	31775839	31782713	30	6874	NA	NA	30	NA	NA	NA
Panc-AdenoCA	89195d56-1ef2-41df-b200-9f18e38f574a	5	1509965	1511503	7	1538	2	3	2	NA	NA	NA
Panc-AdenoCA	89195d56-1ef2-41df-b200-9f18e38f574a	5	44696673	44698860	8	2187	1	4	2	NA	1	NA
Panc-AdenoCA	89195d56-1ef2-41df-b200-9f18e38f574a	6	51403817	51404629	8	812	NA	2	6	NA	NA	NA
Panc-AdenoCA	89195d56-1ef2-41df-b200-9f18e38f574a	6	77223475	77223893	8	418	1	1	6	NA	NA	NA
Panc-AdenoCA	89195d56-1ef2-41df-b200-9f18e38f574a	6	89069564	89070065	9	501	NA	4	5	NA	NA	NA
Panc-AdenoCA	89195d56-1ef2-41df-b200-9f18e38f574a	7	77832131	77832224	6	93	1	NA	4	NA	NA	1
Panc-AdenoCA	89195d56-1ef2-41df-b200-9f18e38f574a	8	31335638	31336354	8	716	NA	2	6	NA	NA	NA
Panc-AdenoCA	89195d56-1ef2-41df-b200-9f18e38f574a	8	100572584	100576151	18	3567	6	3	7	NA	NA	2
Panc-AdenoCA	89195d56-1ef2-41df-b200-9f18e38f574a	8	105502518	105504407	9	1889	NA	NA	9	NA	NA	NA
Panc-AdenoCA	89195d56-1ef2-41df-b200-9f18e38f574a	9	23856196	23857163	6	967	NA	3	3	NA	NA	NA
Panc-AdenoCA	89195d56-1ef2-41df-b200-9f18e38f574a	9	110272956	110273897	7	941	1	3	3	NA	NA	NA
Panc-AdenoCA	89195d56-1ef2-41df-b200-9f18e38f574a	10	57218007	57218357	6	350	2	3	1	NA	NA	NA
Panc-AdenoCA	89195d56-1ef2-41df-b200-9f18e38f574a	12	108467742	108469493	6	1751	2	2	2	NA	NA	NA
Panc-AdenoCA	89195d56-1ef2-41df-b200-9f18e38f574a	14	104427851	104431191	9	3340	5	3	1	NA	NA	NA
Panc-AdenoCA	89195d56-1ef2-41df-b200-9f18e38f574a	15	51187103	51188648	13	1545	4	8	1	NA	NA	NA
Panc-AdenoCA	89195d56-1ef2-41df-b200-9f18e38f574a	15	82063546	82068809	8	5263	1	2	5	NA	NA	NA
Panc-AdenoCA	89195d56-1ef2-41df-b200-9f18e38f574a	16	62699668	62709136	47	9468	2	22	22	NA	1	NA
Panc-AdenoCA	89195d56-1ef2-41df-b200-9f18e38f574a	16	63573871	63576983	9	3112	NA	4	5	NA	NA	NA
Panc-AdenoCA	89195d56-1ef2-41df-b200-9f18e38f574a	16	73374984	73376126	7	1142	NA	4	3	NA	NA	NA
Panc-AdenoCA	89195d56-1ef2-41df-b200-9f18e38f574a	19	54468150	54468468	6	318	1	1	3	NA	NA	1
Ovary-AdenoCA	89dad92e-5b3f-479a-a6da-a94ee7df7f8a	2	240702163	240708324	9	6161	NA	3	6	NA	NA	NA
Ovary-AdenoCA	89dad92e-5b3f-479a-a6da-a94ee7df7f8a	9	16422982	16425537	8	2555	2	3	3	NA	NA	NA
Ovary-AdenoCA	89dad92e-5b3f-479a-a6da-a94ee7df7f8a	9	81280493	81283747	7	3254	NA	3	4	NA	NA	NA
Liver-HCC	8b0c4c91-2d07-4a9b-97a1-d2bda003437e	5	127310141	127310710	6	569	1	1	3	NA	NA	1
Liver-HCC	8b0c4c91-2d07-4a9b-97a1-d2bda003437e	8	27485687	27487973	8	2286	NA	8	NA	NA	NA	NA
Liver-HCC	8b0c4c91-2d07-4a9b-97a1-d2bda003437e	11	68427979	68430273	7	2294	1	4	2	NA	NA	NA
Ovary-AdenoCA	8b28f6d2-4b7d-493b-826e-b119a4fb0cb4	3	179195308	179195433	6	125	3	1	2	NA	NA	NA
Ovary-AdenoCA	8b28f6d2-4b7d-493b-826e-b119a4fb0cb4	3	187075312	187077611	7	2299	3	3	1	NA	NA	NA
Ovary-AdenoCA	8b28f6d2-4b7d-493b-826e-b119a4fb0cb4	3	197702772	197704929	8	2157	5	2	NA	1	NA	NA
Ovary-AdenoCA	8b28f6d2-4b7d-493b-826e-b119a4fb0cb4	7	145387393	145391016	7	3623	2	4	1	NA	NA	NA
Ovary-AdenoCA	8b28f6d2-4b7d-493b-826e-b119a4fb0cb4	17	43459733	43464068	9	4335	4	4	1	NA	NA	NA
Cervix-SCC	8be2b0f5-7bad-4720-b13c-e95b32bdb933	1	8594573	8599935	8	5362	1	2	5	NA	NA	NA
Cervix-SCC	8be2b0f5-7bad-4720-b13c-e95b32bdb933	2	228230150	228234162	6	4012	1	4	1	NA	NA	NA
Cervix-SCC	8be2b0f5-7bad-4720-b13c-e95b32bdb933	3	122485007	122486871	6	1864	2	2	2	NA	NA	NA
Cervix-SCC	8be2b0f5-7bad-4720-b13c-e95b32bdb933	4	187149421	187151849	11	2428	2	8	1	NA	NA	NA
Cervix-SCC	8be2b0f5-7bad-4720-b13c-e95b32bdb933	5	131250529	131254048	8	3519	1	6	1	NA	NA	NA
Cervix-SCC	8be2b0f5-7bad-4720-b13c-e95b32bdb933	6	51309102	51311250	9	2148	2	6	1	NA	NA	NA
Cervix-SCC	8be2b0f5-7bad-4720-b13c-e95b32bdb933	6	53421114	53428872	10	7758	2	5	3	NA	NA	NA
Cervix-SCC	8be2b0f5-7bad-4720-b13c-e95b32bdb933	6	101247803	101253504	7	5701	1	6	NA	NA	NA	NA
Cervix-SCC	8be2b0f5-7bad-4720-b13c-e95b32bdb933	10	75606311	75611865	10	5554	2	4	4	NA	NA	NA
Cervix-SCC	8be2b0f5-7bad-4720-b13c-e95b32bdb933	11	108310513	108312818	6	2305	NA	3	3	NA	NA	NA
Cervix-SCC	8be2b0f5-7bad-4720-b13c-e95b32bdb933	12	26601073	26603283	6	2210	NA	3	3	NA	NA	NA

Cervix-SCC	8be2b0f5-7bad-4720-b13c-e95b32bdb933	12	53331858	53336796	8	4938	3	2	3	NA	NA	NA
Cervix-SCC	8be2b0f5-7bad-4720-b13c-e95b32bdb933	12	54082102	54088500	14	6398	3	5	6	NA	NA	NA
Cervix-SCC	8be2b0f5-7bad-4720-b13c-e95b32bdb933	16	29812898	29815719	6	2821	1	4	1	NA	NA	NA
Cervix-SCC	8be2b0f5-7bad-4720-b13c-e95b32bdb933	18	47511294	47513369	6	2075	1	4	1	NA	NA	NA
Panc-AdenoCA	8be6b14d-286a-471b-a282-ab98bc6050c3	17	54581085	54581342	8	257	NA	2	6	NA	NA	NA
Panc-AdenoCA	8c233a11-3b2e-4273-bbe1-b5a5f5a351d5	6	115383665	115385874	19	2209	2	12	5	NA	NA	NA
Bladder-TCC	8c619cbc-9e91-4716-9711-5236e55d8f46	1	92148777	92152036	6	3259	NA	3	3	NA	NA	NA
Bladder-TCC	8c619cbc-9e91-4716-9711-5236e55d8f46	1	93973591	93975618	7	2027	1	3	3	NA	NA	NA
Bladder-TCC	8c619cbc-9e91-4716-9711-5236e55d8f46	2	9153085	9155750	7	2665	NA	4	3	NA	NA	NA
Bladder-TCC	8c619cbc-9e91-4716-9711-5236e55d8f46	2	10009452	10011898	9	2446	NA	3	6	NA	NA	NA
Bladder-TCC	8c619cbc-9e91-4716-9711-5236e55d8f46	3	48545521	48546724	8	1203	NA	4	4	NA	NA	NA
Bladder-TCC	8c619cbc-9e91-4716-9711-5236e55d8f46	3	49242193	49252292	17	10099	2	4	11	NA	NA	NA
Bladder-TCC	8c619cbc-9e91-4716-9711-5236e55d8f46	3	61976417	61979784	11	3367	NA	2	9	NA	NA	NA
Bladder-TCC	8c619cbc-9e91-4716-9711-5236e55d8f46	3	171163548	171164579	8	1031	1	3	4	NA	NA	NA
Bladder-TCC	8c619cbc-9e91-4716-9711-5236e55d8f46	4	86253152	86253756	6	604	NA	3	3	NA	NA	NA
Bladder-TCC	8c619cbc-9e91-4716-9711-5236e55d8f46	7	3067730	3069540	6	1810	NA	1	5	NA	NA	NA
Bladder-TCC	8c619cbc-9e91-4716-9711-5236e55d8f46	9	93506807	93513193	9	6386	2	6	1	NA	NA	NA
Bladder-TCC	8c619cbc-9e91-4716-9711-5236e55d8f46	10	72116601	72117344	7	743	1	3	3	NA	NA	NA
Bladder-TCC	8c619cbc-9e91-4716-9711-5236e55d8f46	10	94835970	94840921	20	4951	NA	NA	20	NA	NA	NA
Bladder-TCC	8c619cbc-9e91-4716-9711-5236e55d8f46	10	96182250	96182614	7	364	NA	NA	7	NA	NA	NA
Bladder-TCC	8c619cbc-9e91-4716-9711-5236e55d8f46	11	32993900	32997700	9	3800	NA	2	7	NA	NA	NA
Bladder-TCC	8c619cbc-9e91-4716-9711-5236e55d8f46	12	130141763	130143814	7	2051	NA	4	3	NA	NA	NA
Bladder-TCC	8c619cbc-9e91-4716-9711-5236e55d8f46	13	35397263	35398520	8	1257	NA	5	3	NA	NA	NA
Bladder-TCC	8c619cbc-9e91-4716-9711-5236e55d8f46	13	113390419	113392008	7	1589	NA	6	1	NA	NA	NA
Bladder-TCC	8c619cbc-9e91-4716-9711-5236e55d8f46	15	44059518	44062272	6	2754	NA	5	1	NA	NA	NA
Bladder-TCC	8c619cbc-9e91-4716-9711-5236e55d8f46	16	1614648	1616760	6	2112	NA	4	2	NA	NA	NA
Bladder-TCC	8c619cbc-9e91-4716-9711-5236e55d8f46	16	5392921	5394051	7	1130	1	4	2	NA	NA	NA
Bladder-TCC	8c619cbc-9e91-4716-9711-5236e55d8f46	16	69246914	69249649	11	2735	NA	3	8	NA	NA	NA
Bladder-TCC	8c619cbc-9e91-4716-9711-5236e55d8f46	16	89687613	89691059	13	3446	1	2	10	NA	NA	NA
Bladder-TCC	8c619cbc-9e91-4716-9711-5236e55d8f46	17	42295998	42296024	6	26	1	NA	5	NA	NA	NA
Bladder-TCC	8c619cbc-9e91-4716-9711-5236e55d8f46	18	54134932	54137300	7	2368	NA	5	2	NA	NA	NA
Bladder-TCC	8c619cbc-9e91-4716-9711-5236e55d8f46	19	38785101	38791204	12	6103	3	6	3	NA	NA	NA
Bladder-TCC	8c619cbc-9e91-4716-9711-5236e55d8f46	20	61493696	61495438	7	1742	NA	6	1	NA	NA	NA
Bladder-TCC	8c619cbc-9e91-4716-9711-5236e55d8f46	X	44946763	44947175	9	412	2	7	NA	NA	NA	NA
Panc-Endocrine	8cbd1752-7eea-46d3-8623-09e96dfb1fdf	7	150201626	150207303	12	5677	2	4	6	NA	NA	NA
Lymph-CLL	8cce153d-953d-40c5-976b-60f6a143608c	2	89158784	89165307	37	6523	1	5	5	15	5	6
Lymph-CLL	8cce153d-953d-40c5-976b-60f6a143608c	2	89246876	89248722	15	1846	1	1	4	8	1	NA
Lymph-CLL	8cce153d-953d-40c5-976b-60f6a143608c	14	106324868	106363851	54	38983	2	15	13	14	4	6
Lymph-CLL	8cce153d-953d-40c5-976b-60f6a143608c	14	106494165	106494403	9	238	1	1	2	2	2	1
Lymph-CLL	8cce153d-953d-40c5-976b-60f6a143608c	22	23227725	23234934	11	7209	3	3	1	2	2	NA
Lung-SCC	8cf9b32d-3d6f-4898-8c7a-89511b754021	1	78505578	78508841	6	3263	1	1	4	NA	NA	NA
Lung-SCC	8cf9b32d-3d6f-4898-8c7a-89511b754021	2	164457868	164462716	9	4848	2	4	3	NA	NA	NA
Lung-SCC	8cf9b32d-3d6f-4898-8c7a-89511b754021	7	17422394	17424069	6	1675	1	3	2	NA	NA	NA
Lung-SCC	8cf9b32d-3d6f-4898-8c7a-89511b754021	9	20862683	20863628	10	945	3	5	2	NA	NA	NA
Lung-SCC	8cf9b32d-3d6f-4898-8c7a-89511b754021	9	135405266	135407098	10	1832	2	1	7	NA	NA	NA
Lung-SCC	8cf9b32d-3d6f-4898-8c7a-89511b754021	11	109303281	109304559	7	1278	1	6	NA	NA	NA	NA
Lung-SCC	8cf9b32d-3d6f-4898-8c7a-89511b754021	16	27810966	27812369	6	1403	NA	2	4	NA	NA	NA
Lung-SCC	8cf9b32d-3d6f-4898-8c7a-89511b754021	19	33232531	33236962	7	4431	NA	2	5	NA	NA	NA
Lung-SCC	8cf9b32d-3d6f-4898-8c7a-89511b754021	X	132663894	132666194	13	2300	NA	2	10	1	NA	NA
Uterus-AdenoCA	8d4cb709-c95c-4bdc-844b-c0bfa2a3028e	2	154633585	154636491	22	2906	3	7	12	NA	NA	NA
Uterus-AdenoCA	8d4cb709-c95c-4bdc-844b-c0bfa2a3028e	8	61236100	61236389	7	289	3	2	2	NA	NA	NA
Uterus-AdenoCA	8d4cb709-c95c-4bdc-844b-c0bfa2a3028e	8	61262434	61265300	7	2866	3	3	1	NA	NA	NA
Uterus-AdenoCA	8d4cb709-c95c-4bdc-844b-c0bfa2a3028e	8	67323789	67331456	15	7667	2	8	4	NA	NA	1
Skin-Melanoma	8d5ffe62-6dd9-4764-b0b7-5e3010eefe6d	1	119930049	119938291	22	8242	4	7	11	NA	NA	NA
Skin-Melanoma	8d5ffe62-6dd9-4764-b0b7-5e3010eefe6d	1	120292730	120293428	10	698	3	3	4	NA	NA	NA
Skin-Melanoma	8d5ffe62-6dd9-4764-b0b7-5e3010eefe6d	4	55136157	55137636	14	1479	NA	NA	14	NA	NA	NA
Skin-Melanoma	8d5ffe62-6dd9-4764-b0b7-5e3010eefe6d	8	79661058	79662048	10	990	1	4	5	NA	NA	NA
Skin-Melanoma	8d5ffe62-6dd9-4764-b0b7-5e3010eefe6d	8	139347431	139353579	9	6148	NA	NA	9	NA	NA	NA
Skin-Melanoma	8d5ffe62-6dd9-4764-b0b7-5e3010eefe6d	11	30883529	30884741	6	1212	NA	3	3	NA	NA	NA
Panc-AdenoCA	8d67c121-37d0-4fc0-a349-2c9452589d65	3	404972	405356	8	384	NA	2	6	NA	NA	NA

Panc-AdenoCA	8d67c121-37d0-4fc0-a349-2c9452589d65	10	36082303	36083671	10	1368	2	6	2	NA	NA	NA
Panc-AdenoCA	8d67c121-37d0-4fc0-a349-2c9452589d65	12	70747578	70751111	51	3533	2	20	29	NA	NA	NA
Panc-AdenoCA	8d67c121-37d0-4fc0-a349-2c9452589d65	Y	23189954	23191673	10	1719	1	7	2	NA	NA	NA
Liver-HCC	8d7592e2-c622-11e3-bf01-24c6515278c0	13	88646024	88646231	6	207	2	NA	4	NA	NA	NA
ColoRect-AdenoCA	8da641e4-9b12-4163-bd1e-9d5af91e5186	8	6059659	6060278	7	619	NA	6	1	NA	NA	NA
ColoRect-AdenoCA	8da641e4-9b12-4163-bd1e-9d5af91e5186	17	36214364	36215574	14	1210	7	5	2	NA	NA	NA
Uterus-AdenoCA	8dd14f0e-8601-4aa1-864c-3c49e768cdd1	1	119424139	119428190	8	4051	3	4	1	NA	NA	NA
Uterus-AdenoCA	8dd14f0e-8601-4aa1-864c-3c49e768cdd1	19	17542320	17543014	6	694	1	3	2	NA	NA	NA
Uterus-AdenoCA	8dd14f0e-8601-4aa1-864c-3c49e768cdd1	X	55048332	55048738	7	406	1	5	1	NA	NA	NA
Breast-AdenoCa	8e03e773-5557-4e78-889b-4710c515378f	1	188084252	188086188	12	1936	5	7	NA	NA	NA	NA
Breast-AdenoCa	8e03e773-5557-4e78-889b-4710c515378f	1	247258545	247260128	7	1583	4	2	1	NA	NA	NA
Breast-AdenoCa	8e03e773-5557-4e78-889b-4710c515378f	6	66523626	66525100	8	1474	2	5	NA	1	NA	NA
Breast-AdenoCa	8e03e773-5557-4e78-889b-4710c515378f	8	49178936	49190587	14	11651	2	10	2	NA	NA	NA
Breast-AdenoCa	8e03e773-5557-4e78-889b-4710c515378f	X	20389195	20394766	8	5571	1	6	1	NA	NA	NA
Eso-AdenoCa	8e48aa43-6cd1-4aef-83ce-2451b1b86e8e	17	54255080	54255625	7	545	1	2	4	NA	NA	NA
Eso-AdenoCa	8e48aa43-6cd1-4aef-83ce-2451b1b86e8e	17	64455394	64461521	8	6127	2	1	4	1	NA	NA
Eso-AdenoCa	8e48aa43-6cd1-4aef-83ce-2451b1b86e8e	17	74505638	74508306	31	2668	8	12	11	NA	NA	NA
Breast-AdenoCa	8f558713-f32b-403b-aedf-c79efeb41c67	3	96944060	96949943	7	5883	NA	NA	1	1	5	NA
Breast-AdenoCa	8f558713-f32b-403b-aedf-c79efeb41c67	3	118575205	118585055	12	9850	1	6	5	NA	NA	NA
Breast-AdenoCa	8f558713-f32b-403b-aedf-c79efeb41c67	6	57657999	57661393	24	3394	7	15	2	NA	NA	NA
Breast-AdenoCa	8f558713-f32b-403b-aedf-c79efeb41c67	6	82973651	82974750	9	1099	NA	2	4	1	1	1
Breast-AdenoCa	8f558713-f32b-403b-aedf-c79efeb41c67	6	83645195	83651120	9	5925	NA	4	5	NA	NA	NA
Breast-AdenoCa	8f558713-f32b-403b-aedf-c79efeb41c67	6	156452249	156454074	7	1825	5	1	1	NA	NA	NA
Breast-AdenoCa	8f558713-f32b-403b-aedf-c79efeb41c67	17	36006947	36009920	6	2973	NA	1	5	NA	NA	NA
Breast-AdenoCa	8f558713-f32b-403b-aedf-c79efeb41c67	17	36023956	36029356	8	5400	NA	4	2	2	NA	NA
Breast-AdenoCa	8f558713-f32b-403b-aedf-c79efeb41c67	17	36044845	36046632	7	1787	NA	5	2	NA	NA	NA
Breast-AdenoCa	8f558713-f32b-403b-aedf-c79efeb41c67	17	38979535	38981733	10	2198	1	4	5	NA	NA	NA
Breast-AdenoCa	8f558713-f32b-403b-aedf-c79efeb41c67	17	62022565	62023238	8	673	1	4	3	NA	NA	NA
Breast-AdenoCa	8f558713-f32b-403b-aedf-c79efeb41c67	19	47766385	47770745	6	4360	1	NA	5	NA	NA	NA
Kidney-RCC	8fa0484b-5f95-4ec8-b3de-cea2e8f3c5ca	3	89355239	89356471	11	1232	2	7	2	NA	NA	NA
Kidney-RCC	8fa0484b-5f95-4ec8-b3de-cea2e8f3c5ca	13	73247941	73255204	15	7263	2	4	8	NA	NA	1
Lymph-BNHL	8fc11cb6-9d79-41c3-bb45-0dadda890e8d	4	165501893	165503817	6	1924	NA	4	2	NA	NA	NA
Lymph-BNHL	8fc11cb6-9d79-41c3-bb45-0dadda890e8d	6	91005086	91007022	9	1936	NA	1	7	1	NA	NA
Lymph-BNHL	8fc11cb6-9d79-41c3-bb45-0dadda890e8d	9	37024771	37026554	7	1783	NA	3	4	NA	NA	NA
Lymph-BNHL	8fc11cb6-9d79-41c3-bb45-0dadda890e8d	14	106069701	106070132	6	431	1	3	2	NA	NA	NA
Lymph-BNHL	8fc11cb6-9d79-41c3-bb45-0dadda890e8d	14	106326574	106330109	61	3535	2	13	30	8	1	7
Lymph-BNHL	8fc11cb6-9d79-41c3-bb45-0dadda890e8d	14	106994118	106995145	25	1027	1	2	10	5	2	5
Lymph-BNHL	8fc11cb6-9d79-41c3-bb45-0dadda890e8d	18	60983902	60988401	44	4499	1	5	23	7	4	4
Lymph-BNHL	8fc11cb6-9d79-41c3-bb45-0dadda890e8d	22	22379873	22385843	34	5970	1	5	18	5	1	4
Lymph-BNHL	8fc11cb6-9d79-41c3-bb45-0dadda890e8d	22	23203555	23206118	14	2563	NA	1	7	1	1	4
Liver-HCC	8fd5e62c-c622-11e3-bf01-24c6515278c0	1	118697532	118700068	7	2536	1	2	4	NA	NA	NA
Liver-HCC	8fd5e62c-c622-11e3-bf01-24c6515278c0	1	217396849	217397559	10	710	1	4	5	NA	NA	NA
Liver-HCC	8fd5e62c-c622-11e3-bf01-24c6515278c0	1	217716931	217717567	7	636	2	1	4	NA	NA	NA
Panc-AdenoCA	9011cf17-0783-4f9d-b355-4f0fc1a2e78b	1	60804041	60805380	12	1339	5	1	6	NA	NA	NA
Panc-AdenoCA	9011cf17-0783-4f9d-b355-4f0fc1a2e78b	4	66959568	66959839	7	271	NA	NA	7	NA	NA	NA
Panc-AdenoCA	9011cf17-0783-4f9d-b355-4f0fc1a2e78b	8	130097978	130099917	18	1939	2	3	13	NA	NA	NA
Panc-AdenoCA	9011cf17-0783-4f9d-b355-4f0fc1a2e78b	19	21508382	21508758	6	376	2	2	2	NA	NA	NA
Panc-AdenoCA	9011cf17-0783-4f9d-b355-4f0fc1a2e78b	19	38827988	38828576	10	588	2	3	5	NA	NA	NA
Panc-AdenoCA	9011cf17-0783-4f9d-b355-4f0fc1a2e78b	19	56497161	56498567	12	1406	1	4	7	NA	NA	NA
Ovary-AdenoCA	9020b6a5-8325-4b0d-84d9-04371c857910	4	128297966	128302525	7	4559	2	4	1	NA	NA	NA
Ovary-AdenoCA	9020b6a5-8325-4b0d-84d9-04371c857910	6	64443606	64445293	11	1687	1	8	2	NA	NA	NA
Ovary-AdenoCA	9020b6a5-8325-4b0d-84d9-04371c857910	6	68664966	68670835	11	5869	3	5	3	NA	NA	NA
Ovary-AdenoCA	9020b6a5-8325-4b0d-84d9-04371c857910	6	100737406	100737745	7	339	1	2	4	NA	NA	NA
Ovary-AdenoCA	9020b6a5-8325-4b0d-84d9-04371c857910	11	45060616	45061241	6	625	NA	4	2	NA	NA	NA
Ovary-AdenoCA	9020b6a5-8325-4b0d-84d9-04371c857910	19	30050621	30057859	19	7238	3	13	2	NA	NA	1
Ovary-AdenoCA	9020b6a5-8325-4b0d-84d9-04371c857910	19	43623264	43628284	19	5020	2	10	6	NA	NA	1
Breast-AdenoCa	9032b7fe-e38a-4641-a45e-67041668adc4	1	246035567	246039320	8	3753	1	4	2	NA	NA	1
Breast-AdenoCa	9032b7fe-e38a-4641-a45e-67041668adc4	8	82593212	82596137	8	2925	1	6	1	NA	NA	NA
Panc-AdenoCA	9078333d-73d3-496a-9fc3-a94353b7e107	3	99719779	99724832	14	5053	1	6	7	NA	NA	NA
Panc-AdenoCA	9078333d-73d3-496a-9fc3-a94353b7e107	4	187415192	187415871	6	679	2	3	1	NA	NA	NA

Panc-AdenoCA	9078333d-73d3-496a-9fc3-a94353b7e107	13	20141771	20144169	8	2398	3	3	2	NA	NA	NA
Panc-AdenoCA	9078333d-73d3-496a-9fc3-a94353b7e107	18	61348598	61350027	7	1429	1	3	3	NA	NA	NA
Panc-AdenoCA	90b3c237-29a4-48b3-90b3-9f2c0ccb5928	4	155426795	155429785	8	2990	NA	5	3	NA	NA	NA
Panc-AdenoCA	90b3c237-29a4-48b3-90b3-9f2c0ccb5928	6	42968513	42968663	6	150	2	1	3	NA	NA	NA
Panc-AdenoCA	90b3c237-29a4-48b3-90b3-9f2c0ccb5928	8	121679876	121683397	16	3521	3	5	7	NA	NA	1
CNS-GBM	914421d2-898e-433e-bb66-b1710eaae2c7	2	225954505	225956388	6	1883	1	5	NA	NA	NA	NA
Lymph-CLL	915cbb43-9e00-433d-818f-531011bea57e	3	187461951	187463096	8	1145	NA	1	2	3	NA	2
Lymph-CLL	915cbb43-9e00-433d-818f-531011bea57e	14	106324858	106329814	97	4956	5	26	21	22	11	12
Lymph-CLL	915cbb43-9e00-433d-818f-531011bea57e	14	106815249	106816606	11	1357	NA	NA	4	2	2	3
Lymph-CLL	915cbb43-9e00-433d-818f-531011bea57e	22	22516384	22517024	13	640	1	2	6	2	1	1
Lymph-CLL	915cbb43-9e00-433d-818f-531011bea57e	22	23028166	23029460	10	1294	2	1	3	3	1	NA
Lymph-CLL	915cbb43-9e00-433d-818f-531011bea57e	22	23242159	23248364	9	6205	1	4	2	NA	1	1
Bladder-TCC	91f458e6-64b7-454d-a542-b0aa23638fd8	1	55043466	55044255	6	789	NA	3	3	NA	NA	NA
Bladder-TCC	91f458e6-64b7-454d-a542-b0aa23638fd8	4	74848987	74852625	15	3638	1	8	6	NA	NA	NA
Bladder-TCC	91f458e6-64b7-454d-a542-b0aa23638fd8	5	62286417	62287079	10	662	NA	8	2	NA	NA	NA
Bladder-TCC	91f458e6-64b7-454d-a542-b0aa23638fd8	7	8260975	8264024	14	3049	5	3	6	NA	NA	NA
Bladder-TCC	91f458e6-64b7-454d-a542-b0aa23638fd8	8	72028945	72029613	10	668	NA	1	9	NA	NA	NA
Bladder-TCC	91f458e6-64b7-454d-a542-b0aa23638fd8	8	100439696	100445288	15	5592	3	5	6	NA	1	NA
Bladder-TCC	91f458e6-64b7-454d-a542-b0aa23638fd8	10	114836938	114838608	7	1670	NA	6	1	NA	NA	NA
Bladder-TCC	91f458e6-64b7-454d-a542-b0aa23638fd8	10	134017912	134021280	13	3368	4	6	3	NA	NA	NA
Bladder-TCC	91f458e6-64b7-454d-a542-b0aa23638fd8	12	72674197	72685999	15	11802	3	4	7	NA	NA	1
Bladder-TCC	91f458e6-64b7-454d-a542-b0aa23638fd8	12	96007084	96009136	8	2052	NA	3	5	NA	NA	NA
Bladder-TCC	91f458e6-64b7-454d-a542-b0aa23638fd8	12	109471226	109471701	9	475	NA	NA	9	NA	NA	NA
Bladder-TCC	91f458e6-64b7-454d-a542-b0aa23638fd8	13	27326020	27328057	8	2037	1	5	2	NA	NA	NA
Bladder-TCC	91f458e6-64b7-454d-a542-b0aa23638fd8	19	29830589	29835374	7	4785	NA	6	1	NA	NA	NA
Bladder-TCC	91f458e6-64b7-454d-a542-b0aa23638fd8	19	39877398	39877732	7	334	NA	2	5	NA	NA	NA
Eso-AdenoCa	9258860c-a336-4075-996d-2ee0c18b281c	7	38479266	38482647	6	3381	NA	NA	NA	2	4	NA
Lymph-BNHL	929f2216-6ab3-414f-a814-49b69d4b49d9	2	89159309	89159918	19	609	NA	5	3	6	2	3
Lymph-BNHL	929f2216-6ab3-414f-a814-49b69d4b49d9	2	90196442	90198141	10	1699	NA	1	6	2	1	NA
Lymph-BNHL	929f2216-6ab3-414f-a814-49b69d4b49d9	4	76816994	76820719	17	3725	NA	8	9	NA	NA	NA
Lymph-BNHL	929f2216-6ab3-414f-a814-49b69d4b49d9	4	161772811	161779015	9	6204	4	3	2	NA	NA	NA
Lymph-BNHL	929f2216-6ab3-414f-a814-49b69d4b49d9	6	37138248	37141276	13	3028	1	2	10	NA	NA	NA
Lymph-BNHL	929f2216-6ab3-414f-a814-49b69d4b49d9	8	2508071	2508261	6	190	NA	NA	NA	3	1	2
Lymph-BNHL	929f2216-6ab3-414f-a814-49b69d4b49d9	14	106109956	106114481	29	4525	1	7	21	NA	NA	NA
Lymph-BNHL	929f2216-6ab3-414f-a814-49b69d4b49d9	14	106176244	106177345	8	1101	NA	3	5	NA	NA	NA
Lymph-BNHL	929f2216-6ab3-414f-a814-49b69d4b49d9	14	106208351	106214160	14	5809	1	1	12	NA	NA	NA
Lymph-BNHL	929f2216-6ab3-414f-a814-49b69d4b49d9	14	106236883	106241546	50	4663	3	14	33	NA	NA	NA
Lymph-BNHL	929f2216-6ab3-414f-a814-49b69d4b49d9	14	106322585	106357997	153	35412	13	23	55	36	5	21
Lymph-BNHL	929f2216-6ab3-414f-a814-49b69d4b49d9	14	106829834	106830575	26	741	3	3	8	5	3	4
Lymph-BNHL	929f2216-6ab3-414f-a814-49b69d4b49d9	22	22758730	22764542	16	5812	NA	5	6	2	1	2
Lymph-BNHL	929f2216-6ab3-414f-a814-49b69d4b49d9	22	23036792	23040701	11	3909	NA	2	8	NA	1	NA
Lymph-BNHL	929f2216-6ab3-414f-a814-49b69d4b49d9	22	23236216	23248761	25	12545	2	8	9	4	1	1
Panc-AdenoCA	92dc0e0c-842f-40de-9c39-486b491ea80a	11	94529638	94537904	23	8266	2	10	11	NA	NA	NA
Panc-AdenoCA	92dc0e0c-842f-40de-9c39-486b491ea80a	11	109515187	109522107	18	6920	5	7	6	NA	NA	NA
Panc-AdenoCA	92dc0e0c-842f-40de-9c39-486b491ea80a	16	33876795	33879145	15	2350	4	6	5	NA	NA	NA
Liver-HCC	9321341c-c622-11e3-bf01-24c6515278c0	3	173042037	173046800	7	4763	1	2	3	1	NA	NA
Liver-HCC	93a49462-4bd3-4efa-87a5-69006abba5a0	4	80119500	80124647	19	5147	2	7	8	2	NA	NA
Liver-HCC	93a49462-4bd3-4efa-87a5-69006abba5a0	11	57657349	57658917	11	1568	NA	5	5	NA	1	NA
Liver-HCC	93a49462-4bd3-4efa-87a5-69006abba5a0	17	28751007	28753729	12	2722	NA	3	8	NA	1	NA
Ovary-AdenoCA	941fcb56-e059-403d-aab1-0692a3ecc45e	19	19214480	19214804	6	324	3	2	1	NA	NA	NA
Ovary-AdenoCA	941fcb56-e059-403d-aab1-0692a3ecc45e	19	23569742	23570440	9	698	NA	3	6	NA	NA	NA
Ovary-AdenoCA	941fcb56-e059-403d-aab1-0692a3ecc45e	19	30014038	30015680	10	1642	NA	4	6	NA	NA	NA
Ovary-AdenoCA	941fcb56-e059-403d-aab1-0692a3ecc45e	19	42723424	42724059	7	635	NA	4	3	NA	NA	NA
Ovary-AdenoCA	941fcb56-e059-403d-aab1-0692a3ecc45e	19	45627649	45628300	11	651	NA	5	6	NA	NA	NA
Ovary-AdenoCA	941fcb56-e059-403d-aab1-0692a3ecc45e	22	22715761	22715935	9	174	NA	NA	NA	9	NA	NA
Liver-HCC	94431626-c622-11e3-bf01-24c6515278c0	6	57170514	57171592	6	1078	NA	NA	NA	NA	3	3
Liver-HCC	94431626-c622-11e3-bf01-24c6515278c0	17	18618148	18619217	6	1069	NA	NA	NA	1	2	3
Panc-AdenoCA	94652d14-2e4d-4f4a-a4f7-8df77df788c0	9	104807703	104811103	26	3400	5	7	13	1	NA	NA
Panc-AdenoCA	94652d14-2e4d-4f4a-a4f7-8df77df788c0	9	122827360	122827711	11	351	NA	4	7	NA	NA	NA
Panc-AdenoCA	94652d14-2e4d-4f4a-a4f7-8df77df788c0	19	38951860	38954475	9	2615	1	4	4	NA	NA	NA

Panc-AdenoCA	94652d14-2e4d-4f4a-a4f7-8df77df788c0	19	39967546	39973129	12	5583	3	1	7	1	NA	NA	
Panc-AdenoCA	94652d14-2e4d-4f4a-a4f7-8df77df788c0	19	49846181	49851860	27	5679	1	NA	26	NA	NA	NA	
Panc-AdenoCA	94652d14-2e4d-4f4a-a4f7-8df77df788c0	X	85412699	85417111	23	4412	NA	9	14	NA	NA	NA	
Stomach-AdenoCA	9536f736-63bc-4099-bd54-740f5910f4a8	11	111523798	111528006	7	4208	3	1	2	NA	NA	1	
Stomach-AdenoCA	9536f736-63bc-4099-bd54-740f5910f4a8	17	31068484	31068664	8	180	1	4	3	NA	NA	NA	
Panc-AdenoCA	95cc9620-7686-4805-a96c-cf8e659847c3	3	95033030	95038627	15	5597	NA	NA	NA	3	5	7	
Panc-AdenoCA	95cc9620-7686-4805-a96c-cf8e659847c3	5	45605635	45618938	26	13303	NA	NA	NA	12	9	5	
Ovary-AdenoCA	95fc38ac-2b36-4c46-abbf-8d2d52ff9626	12	21750946	21752450	8	1504	1	3	4	NA	NA	NA	
Liver-HCC	96abdbc8-c622-11e3-bf01-24c6515278c0	1	222004239	222011215	32	6976	2	15	14	1	NA	NA	
Liver-HCC	96abdbc8-c622-11e3-bf01-24c6515278c0	15	24764608	24768535	16	3927	3	10	3	NA	NA	NA	
Panc-AdenoCA	96ba50eb-3c12-41ad-ac7b-d23b2001fe44	6	62600669	62601591	8	922	1	2	4	NA	NA	1	
Panc-AdenoCA	96ba50eb-3c12-41ad-ac7b-d23b2001fe44	X	5423923	5424435	6	512	1	3	2	NA	NA	NA	
Panc-AdenoCA	96cca60e-17dc-44c3-90f7-1b57b0e08e5f	11	24820193	24822898	8	2705	1	1	5	NA	NA	1	
Panc-AdenoCA	96cca60e-17dc-44c3-90f7-1b57b0e08e5f	11	29852632	29854752	15	2120	2	7	6	NA	NA	NA	
CNS-GBM	96e3db14-2bb1-4f68-aed6-5e794750c96e	3	3155326	3164352	15	9026	NA	7	8	NA	NA	NA	
CNS-GBM	96e3db14-2bb1-4f68-aed6-5e794750c96e	6	163163836	163168151	10	4315	2	4	3	1	NA	NA	
Uterus-AdenoCA	96f337d2-cec4-4484-87cd-ab2109d2bf50	6	31778403	31778448	7	45	3	NA	2	NA	2	NA	
Breast-AdenoCa	97064332-1d4f-4571-93ff-354ffe6d7df9	8	127655363	127657916	14	2553	3	8	3	NA	NA	NA	
Breast-AdenoCa	97064332-1d4f-4571-93ff-354ffe6d7df9	8	146008861	146010751	12	1890	2	8	2	NA	NA	NA	
Bladder-TCC	973d0577-8ca4-44a1-817f-1d3c1bada151	2	56234009	56237379	6	3370	NA	2	4	NA	NA	NA	
Bladder-TCC	973d0577-8ca4-44a1-817f-1d3c1bada151	8	89361516	89364498	6	2982	2	1	3	NA	NA	NA	
Bladder-TCC	973d0577-8ca4-44a1-817f-1d3c1bada151	8	89844698	89850348	10	5650	1	7	2	NA	NA	NA	
Bladder-TCC	973d0577-8ca4-44a1-817f-1d3c1bada151	9	101141617	101144027	6	2410	1	2	3	NA	NA	NA	
Bladder-TCC	973d0577-8ca4-44a1-817f-1d3c1bada151	14	52007601	52011136	6	3535	1	3	2	NA	NA	NA	
Eso-AdenoCa	9749a9a5-cb7f-4038-a5f9-5e2d6b97c689	1	242731435	242731724	6	289	2	2	2	NA	NA	NA	
Eso-AdenoCa	9749a9a5-cb7f-4038-a5f9-5e2d6b97c689	13	39689708	39690320	6	612	1	4	1	NA	NA	NA	
Eso-AdenoCa	9749a9a5-cb7f-4038-a5f9-5e2d6b97c689	13	41891318	41893891	8	2573	1	2	5	NA	NA	NA	
Panc-AdenoCA	97c46ede-b280-4344-8dbb-e860c6472239	4	175811784	175812915	7	1131	NA	2	5	NA	NA	NA	
Panc-AdenoCA	97c46ede-b280-4344-8dbb-e860c6472239	7	63446892	63450352	6	3460	NA	3	3	NA	NA	NA	
Panc-AdenoCA	97c46ede-b280-4344-8dbb-e860c6472239	10	1864019	1865152	13	1133	1	6	5	1	NA	NA	
Panc-AdenoCA	97c46ede-b280-4344-8dbb-e860c6472239	12	44616160	44616510	6	350	NA	NA	6	NA	NA	NA	
Panc-AdenoCA	97c46ede-b280-4344-8dbb-e860c6472239	16	60983336	60993272	15	9936	1	5	9	NA	NA	NA	
Panc-AdenoCA	97c46ede-b280-4344-8dbb-e860c6472239	17	34235624	34236865	10	1241	2	2	6	NA	NA	NA	
Prost-AdenoCA	982b58c4-02b3-4e7d-8af0-4b133d0c7e50	X	126161935	126162497	6	562	NA	NA	NA	1	5	NA	
Biliary-AdenoCA	983c14b8-f35b-497b-9fd4-5e5f5f0a7aa7	1	236868065	236869688	16	1623	NA	2	14	NA	NA	NA	
Biliary-AdenoCA	983c14b8-f35b-497b-9fd4-5e5f5f0a7aa7	12	26225868	26228551	6	2683	1	NA	4	NA	NA	1	
Biliary-AdenoCA	983c14b8-f35b-497b-9fd4-5e5f5f0a7aa7	12	26265278	26265855	9	577	1	5	3	NA	NA	NA	
Biliary-AdenoCA	983c14b8-f35b-497b-9fd4-5e5f5f0a7aa7	12	27453955	27461841	9	7886	NA	2	6	NA	NA	1	
Biliary-AdenoCA	983c14b8-f35b-497b-9fd4-5e5f5f0a7aa7	22	39184231	39187555	15	3324	3	6	6	NA	NA	NA	
Biliary-AdenoCA	983c14b8-f35b-497b-9fd4-5e5f5f0a7aa7	22	48620867	48628560	15	7693	3	6	6	NA	NA	NA	
Breast-AdenoCa	987528ac-437a-4eb8-a335-4f2076d5c006	2	208990076	208995539	23	5463	8	9	6	NA	NA	NA	
Breast-AdenoCa	987528ac-437a-4eb8-a335-4f2076d5c006	2	209563998	209566722	30	2724	10	14	6	NA	NA	NA	
Breast-AdenoCa	987528ac-437a-4eb8-a335-4f2076d5c006	6	111220040	111220539	6	499	2	4	NA	NA	NA	NA	
Breast-AdenoCa	987528ac-437a-4eb8-a335-4f2076d5c006	6	112140598	112141817	15	1219	3	8	3	1	NA	NA	
Breast-AdenoCa	987528ac-437a-4eb8-a335-4f2076d5c006	6	161661239	161661759	7	520	3	2	2	NA	NA	NA	
Breast-AdenoCa	987528ac-437a-4eb8-a335-4f2076d5c006	7	82117417	82117425	6	8	2	NA	1	3	NA	NA	
Breast-AdenoCa	987528ac-437a-4eb8-a335-4f2076d5c006	7	111252492	111254587	9	2095	2	3	4	NA	NA	NA	
Breast-AdenoCa	987528ac-437a-4eb8-a335-4f2076d5c006	9	71515957	71519082	11	3125	2	1	7	NA	NA	1	
Breast-AdenoCa	987528ac-437a-4eb8-a335-4f2076d5c006	18	866634	867125	19	491	2	4	13	NA	NA	NA	
Breast-AdenoCa	987d41ee-6bab-490c-b0f2-bf28c0089de9	4	175955478	175955569	6	91	NA	NA	6	NA	NA	NA	
Breast-AdenoCa	987d41ee-6bab-490c-b0f2-bf28c0089de9	4	179915732	179920840	13	5108	1	1	11	NA	NA	NA	
Breast-AdenoCa	987d41ee-6bab-490c-b0f2-bf28c0089de9	4	180012741	180029254	19	16513	NA	4	15	NA	NA	NA	
Breast-AdenoCa	987d41ee-6bab-490c-b0f2-bf28c0089de9	4	183125765	183129415	7	3650	NA	NA	6	NA	NA	1	
Breast-AdenoCa	987d41ee-6bab-490c-b0f2-bf28c0089de9	4	188900711	188904784	12	4073	NA	3	9	NA	NA	NA	
Breast-AdenoCa	987d41ee-6bab-490c-b0f2-bf28c0089de9	4	190659858	190661714	9	1856	1	2	6	NA	NA	NA	
Breast-AdenoCa	987d41ee-6bab-490c-b0f2-bf28c0089de9	6	87468665	87469061	7	396	NA	5	2	NA	NA	NA	
Breast-AdenoCa	987d41ee-6bab-490c-b0f2-bf28c0089de9	11	20397483	20401441	7	3958	NA	2	4	NA	NA	1	
Breast-AdenoCa	987d41ee-6bab-490c-b0f2-bf28c0089de9	11	102403912	102408617	7	4705	NA	NA	2	1	4	NA	
Breast-AdenoCa	987d41ee-6bab-490c-b0f2-bf28c0089de9	17	33456560	33461354	6	4794	NA	NA	5	NA	1	NA	
Breast-AdenoCa	987d41ee-6bab-490c-b0f2-bf28c0089de9	17	47575827	47576908	7	1081	NA	NA	7	NA	NA	NA	

Breast-AdenoCa	987d41ee-6bab-490c-b0f2-bf28c0089de9	17	50080974	50096345	17	15371	3	9	2	1	2	NA
Breast-AdenoCa	987d41ee-6bab-490c-b0f2-bf28c0089de9	17	55685086	55686253	8	1167	NA	NA	8	NA	NA	NA
Breast-AdenoCa	987d41ee-6bab-490c-b0f2-bf28c0089de9	X	53560335	53562391	6	2056	NA	4	2	NA	NA	NA
CNS-GBM	9880c3c9-5685-42a7-8fe9-7585ea1a1d37	7	55347445	55351873	13	4428	1	9	3	NA	NA	NA
CNS-GBM	9880c3c9-5685-42a7-8fe9-7585ea1a1d37	9	21134273	21134882	7	609	NA	6	1	NA	NA	NA
CNS-GBM	9880c3c9-5685-42a7-8fe9-7585ea1a1d37	9	22370441	22373180	8	2739	3	4	1	NA	NA	NA
Liver-HCC	992d9566-b1da-421a-bf36-d23382b115fa	2	117001767	117002675	8	908	1	6	1	NA	NA	NA
Liver-HCC	992d9566-b1da-421a-bf36-d23382b115fa	5	121720588	121721068	7	480	NA	NA	NA	1	5	1
Lymph-BNHL	995a1ad2-faca-4a37-a59d-e62455985afb	1	167598375	167601569	23	3194	1	1	20	1	NA	NA
Lymph-BNHL	995a1ad2-faca-4a37-a59d-e62455985afb	1	198608734	198610282	9	1548	NA	NA	5	1	2	1
Lymph-BNHL	995a1ad2-faca-4a37-a59d-e62455985afb	1	203273976	203277147	69	3171	9	12	36	3	3	6
Lymph-BNHL	995a1ad2-faca-4a37-a59d-e62455985afb	1	226924001	226926383	14	2382	1	2	10	1	NA	NA
Lymph-BNHL	995a1ad2-faca-4a37-a59d-e62455985afb	2	89154339	89292013	203	1E+05	12	23	73	34	28	33
Lymph-BNHL	995a1ad2-faca-4a37-a59d-e62455985afb	2	96809448	96810938	9	1490	2	NA	7	NA	NA	NA
Lymph-BNHL	995a1ad2-faca-4a37-a59d-e62455985afb	2	100757259	100758558	9	1299	NA	2	6	NA	NA	1
Lymph-BNHL	995a1ad2-faca-4a37-a59d-e62455985afb	2	136874448	136875082	8	634	1	2	4	1	NA	NA
Lymph-BNHL	995a1ad2-faca-4a37-a59d-e62455985afb	3	16552195	16556188	9	3993	1	2	5	1	NA	NA
Lymph-BNHL	995a1ad2-faca-4a37-a59d-e62455985afb	3	186739852	186742823	11	2971	NA	2	8	NA	NA	1
Lymph-BNHL	995a1ad2-faca-4a37-a59d-e62455985afb	3	187456599	187468656	50	12057	4	10	26	4	3	3
Lymph-BNHL	995a1ad2-faca-4a37-a59d-e62455985afb	4	50412	59830	12	9418	NA	5	5	NA	1	1
Lymph-BNHL	995a1ad2-faca-4a37-a59d-e62455985afb	4	25861946	25865972	7	4026	NA	2	5	NA	NA	NA
Lymph-BNHL	995a1ad2-faca-4a37-a59d-e62455985afb	4	40194144	40256378	74	62234	5	15	43	2	4	5
Lymph-BNHL	995a1ad2-faca-4a37-a59d-e62455985afb	4	74483816	74485567	9	1751	NA	3	4	1	1	NA
Lymph-BNHL	995a1ad2-faca-4a37-a59d-e62455985afb	5	131824809	131826141	18	1332	NA	3	15	NA	NA	NA
Lymph-BNHL	995a1ad2-faca-4a37-a59d-e62455985afb	5	149785911	149792128	20	6217	1	5	10	1	2	1
Lymph-BNHL	995a1ad2-faca-4a37-a59d-e62455985afb	5	158524101	158529446	31	5345	1	2	25	1	NA	2
Lymph-BNHL	995a1ad2-faca-4a37-a59d-e62455985afb	6	26123678	26129062	8	5384	NA	1	7	NA	NA	NA
Lymph-BNHL	995a1ad2-faca-4a37-a59d-e62455985afb	6	26156807	26158768	8	1961	NA	2	5	NA	1	NA
Lymph-BNHL	995a1ad2-faca-4a37-a59d-e62455985afb	6	37138279	37140056	15	1777	3	3	9	NA	NA	NA
Lymph-BNHL	995a1ad2-faca-4a37-a59d-e62455985afb	6	134489585	134497632	20	8047	1	6	11	1	1	NA
Lymph-BNHL	995a1ad2-faca-4a37-a59d-e62455985afb	6	150954522	150956939	8	2417	NA	1	7	NA	NA	NA
Lymph-BNHL	995a1ad2-faca-4a37-a59d-e62455985afb	6	159464876	159465556	8	680	3	NA	NA	2	1	2
Lymph-BNHL	995a1ad2-faca-4a37-a59d-e62455985afb	9	37024170	37073126	50	48956	4	11	32	1	2	NA
Lymph-BNHL	995a1ad2-faca-4a37-a59d-e62455985afb	9	37381027	37399812	23	18785	2	4	15	1	1	NA
Lymph-BNHL	995a1ad2-faca-4a37-a59d-e62455985afb	9	85767460	85772818	7	5358	1	NA	2	1	1	2
Lymph-BNHL	995a1ad2-faca-4a37-a59d-e62455985afb	11	65190367	65191293	8	926	1	2	5	NA	NA	NA
Lymph-BNHL	995a1ad2-faca-4a37-a59d-e62455985afb	11	102188540	102201588	16	13048	1	2	5	2	2	4
Lymph-BNHL	995a1ad2-faca-4a37-a59d-e62455985afb	11	111249111	111249675	8	564	NA	1	7	NA	NA	NA
Lymph-BNHL	995a1ad2-faca-4a37-a59d-e62455985afb	11	128386509	128399300	32	12791	NA	8	21	NA	2	1
Lymph-BNHL	995a1ad2-faca-4a37-a59d-e62455985afb	12	8762169	8764545	10	2376	2	1	5	1	1	NA
Lymph-BNHL	995a1ad2-faca-4a37-a59d-e62455985afb	12	92537402	92539572	22	2170	NA	4	14	1	2	1
Lymph-BNHL	995a1ad2-faca-4a37-a59d-e62455985afb	12	113493230	113536315	77	43085	8	7	50	7	2	3
Lymph-BNHL	995a1ad2-faca-4a37-a59d-e62455985afb	12	122459181	122463599	22	4418	2	5	14	NA	1	NA
Lymph-BNHL	995a1ad2-faca-4a37-a59d-e62455985afb	13	46958832	46962778	7	3946	1	2	2	1	NA	1
Lymph-BNHL	995a1ad2-faca-4a37-a59d-e62455985afb	14	69256739	69262278	21	5539	4	7	10	NA	NA	NA
Lymph-BNHL	995a1ad2-faca-4a37-a59d-e62455985afb	14	106110093	106115124	12	5031	2	3	7	NA	NA	NA
Lymph-BNHL	995a1ad2-faca-4a37-a59d-e62455985afb	14	106208758	106215087	20	6329	2	4	10	NA	2	2
Lymph-BNHL	995a1ad2-faca-4a37-a59d-e62455985afb	14	106237000	106242534	23	5534	3	2	17	1	NA	NA
Lymph-BNHL	995a1ad2-faca-4a37-a59d-e62455985afb	14	106321039	106370993	108	49954	8	20	58	7	10	5
Lymph-BNHL	995a1ad2-faca-4a37-a59d-e62455985afb	14	106668134	106677533	16	9399	NA	NA	8	4	NA	4
Lymph-BNHL	995a1ad2-faca-4a37-a59d-e62455985afb	14	107176258	107179039	12	2781	NA	2	9	NA	1	NA
Lymph-BNHL	995a1ad2-faca-4a37-a59d-e62455985afb	14	107253677	107259526	7	5849	NA	2	4	NA	NA	1
Lymph-BNHL	995a1ad2-faca-4a37-a59d-e62455985afb	15	80256783	80270906	44	14123	3	11	22	1	7	NA
Lymph-BNHL	995a1ad2-faca-4a37-a59d-e62455985afb	15	80352303	80355725	30	3422	1	12	12	NA	3	2
Lymph-BNHL	995a1ad2-faca-4a37-a59d-e62455985afb	16	10971287	10974559	57	3272	4	13	39	NA	NA	1
Lymph-BNHL	995a1ad2-faca-4a37-a59d-e62455985afb	16	11347154	11349771	27	2617	4	6	15	NA	1	1
Lymph-BNHL	995a1ad2-faca-4a37-a59d-e62455985afb	17	56408450	56409665	14	1215	NA	3	9	NA	2	NA
Lymph-BNHL	995a1ad2-faca-4a37-a59d-e62455985afb	17	57915636	57918417	7	2781	NA	1	6	NA	NA	NA
Lymph-BNHL	995a1ad2-faca-4a37-a59d-e62455985afb	17	62006374	62009479	17	3105	NA	2	13	1	1	NA
Lymph-BNHL	995a1ad2-faca-4a37-a59d-e62455985afb	22	23223221	23294726	181	71505	7	31	##	16	8	18

Lymph-BNHL	995a1ad2-faca-4a37-a59d-e62455985afb	22	29195059	29196300	11	1241	1	4	4	NA	1	1
Lymph-BNHL	995a1ad2-faca-4a37-a59d-e62455985afb	X	12993422	12994206	6	784	NA	1	3	1	NA	1
Uterus-AdenoCA	99696731-c88f-427b-975b-1d90013e63ff	2	231189295	231189381	7	86	NA	1	NA	6	NA	NA
Uterus-AdenoCA	99696731-c88f-427b-975b-1d90013e63ff	2	233827697	233828502	7	805	2	2	3	NA	NA	NA
Liver-HCC	99bdc3da-c622-11e3-bf01-24c6515278c0	1	161786133	161787393	8	1260	1	3	4	NA	NA	NA
Liver-HCC	99bdc3da-c622-11e3-bf01-24c6515278c0	1	162714525	162716000	13	1475	1	5	7	NA	NA	NA
Liver-HCC	99bdc3da-c622-11e3-bf01-24c6515278c0	6	31432980	31435103	11	2123	1	3	7	NA	NA	NA
Liver-HCC	99bdc3da-c622-11e3-bf01-24c6515278c0	8	73975581	73976092	8	511	1	4	3	NA	NA	NA
Lymph-CLL	99e7016a-c3c1-431c-8838-beb78a3cc017	14	106211000	106213014	15	2014	1	1	13	NA	NA	NA
Lymph-CLL	99e7016a-c3c1-431c-8838-beb78a3cc017	14	106324932	106330024	27	5092	3	4	13	5	1	1
Lymph-CLL	99e7016a-c3c1-431c-8838-beb78a3cc017	14	107012993	107013849	25	856	NA	3	6	11	3	2
Lymph-CLL	99e7016a-c3c1-431c-8838-beb78a3cc017	22	23055068	23055596	7	528	NA	3	2	1	NA	1
Lymph-CLL	99e7016a-c3c1-431c-8838-beb78a3cc017	22	23235998	23242043	10	6045	NA	6	NA	2	NA	2
Ovary-AdenoCA	9a5bb831-8cb8-4de0-b94b-088cb38def1a	3	80594246	80596093	11	1847	4	4	3	NA	NA	NA
Ovary-AdenoCA	9a5bb831-8cb8-4de0-b94b-088cb38def1a	3	95640042	95642503	12	2461	NA	9	3	NA	NA	NA
Ovary-AdenoCA	9a5bb831-8cb8-4de0-b94b-088cb38def1a	3	102742299	102749133	9	6834	3	1	5	NA	NA	NA
Ovary-AdenoCA	9a5bb831-8cb8-4de0-b94b-088cb38def1a	3	129301933	129308374	37	6441	12	7	18	NA	NA	NA
Ovary-AdenoCA	9a5bb831-8cb8-4de0-b94b-088cb38def1a	3	133060655	133060980	7	325	1	1	5	NA	NA	NA
Ovary-AdenoCA	9a5bb831-8cb8-4de0-b94b-088cb38def1a	3	133289679	133291036	10	1357	2	2	6	NA	NA	NA
Ovary-AdenoCA	9a5bb831-8cb8-4de0-b94b-088cb38def1a	6	62932459	62934377	9	1918	NA	4	5	NA	NA	NA
Ovary-AdenoCA	9a5bb831-8cb8-4de0-b94b-088cb38def1a	7	141794857	141795277	8	420	1	3	4	NA	NA	NA
Liver-HCC	9aac83e4-c622-11e3-bf01-24c6515278c0	19	6606281	6609761	16	3480	NA	4	12	NA	NA	NA
Liver-HCC	9aac83e4-c622-11e3-bf01-24c6515278c0	19	15682351	15683757	38	1406	NA	NA	38	NA	NA	NA
Liver-HCC	9aac83e4-c622-11e3-bf01-24c6515278c0	19	16084976	16086827	15	1851	NA	1	14	NA	NA	NA
Liver-HCC	9aac83e4-c622-11e3-bf01-24c6515278c0	19	16352163	16353056	8	893	NA	NA	8	NA	NA	NA
Kidney-RCC	9ae0744a-9bc1-4cd7-b7cf-c6569ed9e4aa	8	106192950	106195484	6	2534	NA	NA	2	3	NA	1
Kidney-RCC	9ae0744a-9bc1-4cd7-b7cf-c6569ed9e4aa	11	20447637	20451749	7	4112	1	1	1	4	NA	NA
Kidney-RCC	9ae0744a-9bc1-4cd7-b7cf-c6569ed9e4aa	X	125776946	125778117	8	1171	1	4	3	NA	NA	NA
Eso-AdenoCa	9ae33a63-6b1a-43bf-9205-463c867bcbc4	10	113962107	113964102	6	1995	1	4	1	NA	NA	NA
Lymph-BNHL	9b3e7a03-cf8c-4da8-bc36-20129d7783ba	1	191181498	191184361	7	2863	1	1	1	3	NA	1
Lymph-BNHL	9b3e7a03-cf8c-4da8-bc36-20129d7783ba	2	12806180	12807014	6	834	NA	1	1	3	NA	1
Lymph-BNHL	9b3e7a03-cf8c-4da8-bc36-20129d7783ba	2	12829287	12832847	7	3560	2	NA	1	2	1	1
Lymph-BNHL	9b3e7a03-cf8c-4da8-bc36-20129d7783ba	2	89156968	89160851	97	3883	3	15	20	30	18	11
Lymph-BNHL	9b3e7a03-cf8c-4da8-bc36-20129d7783ba	3	171801583	171801730	7	147	NA	1	NA	1	2	3
Lymph-BNHL	9b3e7a03-cf8c-4da8-bc36-20129d7783ba	3	187460676	187463220	35	2544	2	9	8	9	6	1
Lymph-BNHL	9b3e7a03-cf8c-4da8-bc36-20129d7783ba	4	35001091	35002561	6	1470	NA	NA	NA	2	1	3
Lymph-BNHL	9b3e7a03-cf8c-4da8-bc36-20129d7783ba	4	180612447	180618765	9	6318	1	2	NA	5	1	NA
Lymph-BNHL	9b3e7a03-cf8c-4da8-bc36-20129d7783ba	6	14118169	14119087	10	918	NA	1	3	3	2	1
Lymph-BNHL	9b3e7a03-cf8c-4da8-bc36-20129d7783ba	6	102863784	102870184	8	6400	1	NA	1	5	1	NA
Lymph-BNHL	9b3e7a03-cf8c-4da8-bc36-20129d7783ba	6	134492247	134495835	8	3588	1	3	2	2	NA	NA
Lymph-BNHL	9b3e7a03-cf8c-4da8-bc36-20129d7783ba	7	12853689	12858915	7	5226	NA	NA	NA	2	2	3
Lymph-BNHL	9b3e7a03-cf8c-4da8-bc36-20129d7783ba	7	80258700	80263516	7	4816	NA	1	NA	3	1	2
Lymph-BNHL	9b3e7a03-cf8c-4da8-bc36-20129d7783ba	7	110663348	110675279	14	11931	NA	NA	3	NA	4	7
Lymph-BNHL	9b3e7a03-cf8c-4da8-bc36-20129d7783ba	7	110678267	110683761	7	5494	NA	NA	NA	2	2	3
Lymph-BNHL	9b3e7a03-cf8c-4da8-bc36-20129d7783ba	7	110716795	110725254	11	8459	NA	1	NA	6	2	2
Lymph-BNHL	9b3e7a03-cf8c-4da8-bc36-20129d7783ba	7	110765764	110774151	10	8387	1	NA	NA	2	1	6
Lymph-BNHL	9b3e7a03-cf8c-4da8-bc36-20129d7783ba	7	110804963	110809971	7	5008	1	1	2	NA	1	2
Lymph-BNHL	9b3e7a03-cf8c-4da8-bc36-20129d7783ba	7	110816285	110819541	8	3256	NA	NA	1	3	3	1
Lymph-BNHL	9b3e7a03-cf8c-4da8-bc36-20129d7783ba	7	110834882	110840008	7	5126	NA	NA	NA	3	2	2
Lymph-BNHL	9b3e7a03-cf8c-4da8-bc36-20129d7783ba	9	37025333	37026843	6	1510	2	1	3	NA	NA	NA
Lymph-BNHL	9b3e7a03-cf8c-4da8-bc36-20129d7783ba	10	58473504	58477558	8	4054	1	NA	NA	5	1	1
Lymph-BNHL	9b3e7a03-cf8c-4da8-bc36-20129d7783ba	11	40843087	40846984	7	3897	NA	NA	NA	3	NA	4
Lymph-BNHL	9b3e7a03-cf8c-4da8-bc36-20129d7783ba	12	122459241	122463184	18	3943	NA	3	12	3	NA	NA
Lymph-BNHL	9b3e7a03-cf8c-4da8-bc36-20129d7783ba	13	75983767	75991235	15	7468	NA	2	4	4	4	1
Lymph-BNHL	9b3e7a03-cf8c-4da8-bc36-20129d7783ba	14	106066575	106096399	38	29824	5	12	19	1	NA	1
Lymph-BNHL	9b3e7a03-cf8c-4da8-bc36-20129d7783ba	14	106209802	106213645	7	3843	2	4	NA	NA	1	NA
Lymph-BNHL	9b3e7a03-cf8c-4da8-bc36-20129d7783ba	14	106238463	106241463	12	3000	1	3	6	NA	2	NA
Lymph-BNHL	9b3e7a03-cf8c-4da8-bc36-20129d7783ba	14	106320057	106330136	79	10079	8	20	23	16	8	4
Lymph-BNHL	9b3e7a03-cf8c-4da8-bc36-20129d7783ba	14	106573644	106575949	15	2305	NA	2	4	2	5	2
Lymph-BNHL	9b3e7a03-cf8c-4da8-bc36-20129d7783ba	16	10971563	10974364	13	2801	1	3	9	NA	NA	NA

Lymph-BNHL	9b3e7a03-cf8c-4da8-bc36-20129d7783ba	18	60983923	60988087	35	4164	2	3	17	7	3	3
Lymph-BNHL	9b3e7a03-cf8c-4da8-bc36-20129d7783ba	22	23195456	23199232	6	3776	2	NA	1	2	1	NA
Lymph-BNHL	9b3e7a03-cf8c-4da8-bc36-20129d7783ba	22	23222116	23320018	179	97902	4	41	44	56	18	16
Lymph-BNHL	9b3e7a03-cf8c-4da8-bc36-20129d7783ba	X	32824563	32836343	22	11780	1	1	NA	13	4	3
Eso-AdenoCa	9ba6be6d-2032-444b-ab3c-dbee8023759d	8	29053445	29057481	10	4036	NA	3	7	NA	NA	NA
Eso-AdenoCa	9c00828e-e9ae-4b9c-959e-34837ee2b230	3	161042706	161046613	7	3907	NA	NA	NA	1	4	2
Eso-AdenoCa	9c00828e-e9ae-4b9c-959e-34837ee2b230	12	70621089	70621949	7	860	2	5	NA	NA	NA	NA
Eso-AdenoCa	9c00828e-e9ae-4b9c-959e-34837ee2b230	13	38565054	38569444	11	4390	NA	9	2	NA	NA	NA
Lymph-BNHL	9c27fedd-b1b3-4af0-9e9b-20271854db08	2	89157089	89160111	26	3022	7	11	6	1	1	NA
Lymph-BNHL	9c27fedd-b1b3-4af0-9e9b-20271854db08	2	111877993	111900426	26	22433	4	15	4	1	2	NA
Lymph-BNHL	9c27fedd-b1b3-4af0-9e9b-20271854db08	3	187456241	187463989	28	7748	3	14	9	NA	2	NA
Lymph-BNHL	9c27fedd-b1b3-4af0-9e9b-20271854db08	3	187957456	187963484	10	6028	1	4	2	NA	1	2
Lymph-BNHL	9c27fedd-b1b3-4af0-9e9b-20271854db08	4	40196491	40202683	9	6192	2	4	2	NA	1	NA
Lymph-BNHL	9c27fedd-b1b3-4af0-9e9b-20271854db08	5	36435192	36436825	6	1633	2	1	2	NA	NA	1
Lymph-BNHL	9c27fedd-b1b3-4af0-9e9b-20271854db08	5	165554546	165554914	10	368	2	2	6	NA	NA	NA
Lymph-BNHL	9c27fedd-b1b3-4af0-9e9b-20271854db08	6	14118301	14119735	24	1434	4	9	5	3	3	NA
Lymph-BNHL	9c27fedd-b1b3-4af0-9e9b-20271854db08	6	134492472	134495359	7	2887	NA	2	5	NA	NA	NA
Lymph-BNHL	9c27fedd-b1b3-4af0-9e9b-20271854db08	9	37383896	37385303	11	1407	NA	4	6	NA	1	NA
Lymph-BNHL	9c27fedd-b1b3-4af0-9e9b-20271854db08	11	65190684	65191497	6	813	2	3	1	NA	NA	NA
Lymph-BNHL	9c27fedd-b1b3-4af0-9e9b-20271854db08	12	8762578	8781460	24	18882	2	12	8	NA	2	NA
Lymph-BNHL	9c27fedd-b1b3-4af0-9e9b-20271854db08	12	25205990	25211070	9	5080	1	7	1	NA	NA	NA
Lymph-BNHL	9c27fedd-b1b3-4af0-9e9b-20271854db08	14	106327599	106348949	35	21350	NA	13	9	3	2	8
Lymph-BNHL	9c27fedd-b1b3-4af0-9e9b-20271854db08	16	10971520	10974293	18	2773	2	8	8	NA	NA	NA
Lymph-BNHL	9c27fedd-b1b3-4af0-9e9b-20271854db08	16	11348395	11349545	8	1150	NA	6	1	NA	NA	1
Lymph-BNHL	9c27fedd-b1b3-4af0-9e9b-20271854db08	16	85932950	85943016	12	10066	NA	7	3	1	1	NA
Lymph-BNHL	9c27fedd-b1b3-4af0-9e9b-20271854db08	20	7745637	7750741	8	5104	NA	2	2	NA	NA	4
Lymph-BNHL	9c27fedd-b1b3-4af0-9e9b-20271854db08	20	49127316	49131185	9	3869	1	4	3	NA	1	NA
Lymph-BNHL	9c27fedd-b1b3-4af0-9e9b-20271854db08	22	23223168	23236545	62	13377	4	32	14	NA	7	5
Lymph-BNHL	9c27fedd-b1b3-4af0-9e9b-20271854db08	X	33145671	33156617	15	10946	1	7	7	NA	NA	NA
Lymph-BNHL	9c27fedd-b1b3-4af0-9e9b-20271854db08	X	64069631	64071596	9	1965	1	4	3	1	NA	NA
Panc-AdenoCA	9c399826-087f-44b9-98c0-bd881646b0f4	5	8472584	8473485	11	901	4	2	5	NA	NA	NA
Panc-AdenoCA	9c399826-087f-44b9-98c0-bd881646b0f4	5	14613574	14613867	8	293	2	NA	6	NA	NA	NA
Panc-AdenoCA	9c399826-087f-44b9-98c0-bd881646b0f4	11	20532344	20534677	17	2333	2	6	9	NA	NA	NA
Panc-AdenoCA	9c399826-087f-44b9-98c0-bd881646b0f4	12	25413042	25413150	6	108	NA	3	3	NA	NA	NA
Breast-AdenoCa	9c70688d-6e43-4520-9262-eaae4e4d597d	11	73448166	73449366	6	1200	1	3	1	NA	1	NA
Breast-AdenoCa	9c70688d-6e43-4520-9262-eaae4e4d597d	11	79359322	79359728	9	406	NA	5	4	NA	NA	NA
Breast-AdenoCa	9c70688d-6e43-4520-9262-eaae4e4d597d	17	29684279	29685030	7	751	2	4	1	NA	NA	NA
Breast-AdenoCa	9c70688d-6e43-4520-9262-eaae4e4d597d	17	60390917	60392981	7	2064	1	2	4	NA	NA	NA
Panc-AdenoCA	9c857452-c81d-4b9e-87a8-5b41f4394f7c	12	44108022	44108539	6	517	NA	2	4	NA	NA	NA
Panc-AdenoCA	9c857452-c81d-4b9e-87a8-5b41f4394f7c	19	9450162	9452759	10	2597	NA	5	5	NA	NA	NA
Liver-HCC	9cb80280-c622-11e3-bf01-24c6515278c0	10	57743410	57747333	9	3923	3	3	3	NA	NA	NA
Panc-Endocrine	9cfa4e8f-ea4d-433b-8c2c-54f66ecb7e43	6	796335	796655	6	320	NA	NA	6	NA	NA	NA
Bone-Leiomyo	9d691fa3-5c32-4b98-85c4-f6e0fd4c37e0	1	26433165	26433605	10	440	NA	NA	10	NA	NA	NA
Bone-Leiomyo	9d691fa3-5c32-4b98-85c4-f6e0fd4c37e0	4	13054932	13056691	7	1759	2	3	2	NA	NA	NA
Bone-Leiomyo	9d691fa3-5c32-4b98-85c4-f6e0fd4c37e0	4	13565836	13567447	7	1611	3	NA	4	NA	NA	NA
Bone-Leiomyo	9d691fa3-5c32-4b98-85c4-f6e0fd4c37e0	4	35709016	35709758	7	742	NA	2	5	NA	NA	NA
Bone-Leiomyo	9d691fa3-5c32-4b98-85c4-f6e0fd4c37e0	12	34519401	34523523	9	4122	1	3	5	NA	NA	NA
Bone-Leiomyo	9d691fa3-5c32-4b98-85c4-f6e0fd4c37e0	12	58632294	58636509	7	4215	2	1	4	NA	NA	NA
Bone-Leiomyo	9d691fa3-5c32-4b98-85c4-f6e0fd4c37e0	12	63473412	63477190	7	3778	NA	2	5	NA	NA	NA
Bone-Leiomyo	9d691fa3-5c32-4b98-85c4-f6e0fd4c37e0	12	63481588	63497544	33	15956	2	19	11	1	NA	NA
Bone-Leiomyo	9d691fa3-5c32-4b98-85c4-f6e0fd4c37e0	12	65006354	65007279	9	925	1	5	3	NA	NA	NA
Bone-Leiomyo	9d691fa3-5c32-4b98-85c4-f6e0fd4c37e0	12	68096405	68098407	7	2002	NA	1	6	NA	NA	NA
Bone-Leiomyo	9d691fa3-5c32-4b98-85c4-f6e0fd4c37e0	12	68248123	68248805	7	682	2	3	2	NA	NA	NA
Bone-Leiomyo	9d691fa3-5c32-4b98-85c4-f6e0fd4c37e0	12	130509426	130511010	7	1584	NA	NA	7	NA	NA	NA
Bone-Leiomyo	9d691fa3-5c32-4b98-85c4-f6e0fd4c37e0	15	101454503	101457766	6	3263	2	2	2	NA	NA	NA
Bone-Leiomyo	9d691fa3-5c32-4b98-85c4-f6e0fd4c37e0	17	28232263	28235390	6	3127	1	4	1	NA	NA	NA
Breast-AdenoCa	9ddf2119-a222-4fa5-a9f3-0bec7eeea36b	1	62680446	62694376	39	13930	4	8	24	NA	NA	3
Breast-AdenoCa	9ddf2119-a222-4fa5-a9f3-0bec7eeea36b	2	160852766	160860373	14	7607	1	6	7	NA	NA	NA
Breast-AdenoCa	9ddf2119-a222-4fa5-a9f3-0bec7eeea36b	7	144216627	144220888	6	4261	2	2	2	NA	NA	NA
Breast-AdenoCa	9ddf2119-a222-4fa5-a9f3-0bec7eeea36b	8	47927915	47928115	6	200	2	1	3	NA	NA	NA

Breast-AdenoCa	9ddf2119-a222-4fa5-a9f3-0bec7eeea36b	10	31569406	31572401	9	2995	2	3	3	NA	1	NA
Breast-AdenoCa	9ddf2119-a222-4fa5-a9f3-0bec7eeea36b	19	5414034	5431526	54	17492	12	15	26	NA	NA	1
Breast-AdenoCa	9ddf2119-a222-4fa5-a9f3-0bec7eeea36b	19	44487697	44498460	19	10763	6	3	10	NA	NA	NA
Breast-AdenoCa	9ddf2119-a222-4fa5-a9f3-0bec7eeea36b	20	46702344	46705793	6	3449	2	2	2	NA	NA	NA
Eso-AdenoCa	9de495d1-55b2-4535-9b0a-a999df35977c	1	175888573	175889808	11	1235	NA	3	8	NA	NA	NA
Panc-AdenoCA	9df57388-7de1-4c90-910e-ef63a246dff1	12	23603791	23612227	15	8436	2	8	5	NA	NA	NA
Panc-AdenoCA	9df57388-7de1-4c90-910e-ef63a246dff1	12	24159176	24161019	10	1843	3	2	5	NA	NA	NA
Panc-AdenoCA	9df57388-7de1-4c90-910e-ef63a246dff1	22	41218640	41219436	6	796	1	4	1	NA	NA	NA
Panc-AdenoCA	9df57388-7de1-4c90-910e-ef63a246dff1	22	50095126	50096484	11	1358	NA	8	3	NA	NA	NA
Skin-Melanoma	9e0009d1-c993-4247-9706-88ee84591dec	4	41148422	41154543	9	6121	4	1	4	NA	NA	NA
Skin-Melanoma	9e0009d1-c993-4247-9706-88ee84591dec	4	54329195	54330937	7	1742	1	5	1	NA	NA	NA
Skin-Melanoma	9e0009d1-c993-4247-9706-88ee84591dec	4	54350086	54351151	10	1065	NA	6	3	1	NA	NA
Skin-Melanoma	9e0009d1-c993-4247-9706-88ee84591dec	4	55030253	55031247	7	994	1	3	3	NA	NA	NA
Skin-Melanoma	9e0009d1-c993-4247-9706-88ee84591dec	4	56938873	56940473	8	1600	NA	7	1	NA	NA	NA
Skin-Melanoma	9e0009d1-c993-4247-9706-88ee84591dec	4	97489966	97491558	7	1592	NA	4	3	NA	NA	NA
Skin-Melanoma	9e0009d1-c993-4247-9706-88ee84591dec	4	105146877	105149029	13	2152	NA	5	8	NA	NA	NA
Skin-Melanoma	9e0009d1-c993-4247-9706-88ee84591dec	4	106962968	106964980	20	2012	2	12	6	NA	NA	NA
Skin-Melanoma	9e0009d1-c993-4247-9706-88ee84591dec	5	3219157	3229200	34	10043	11	6	16	NA	NA	1
Skin-Melanoma	9e0009d1-c993-4247-9706-88ee84591dec	5	29220862	29226189	8	5327	3	2	3	NA	NA	NA
Skin-Melanoma	9e0009d1-c993-4247-9706-88ee84591dec	5	30519960	30526313	13	6353	NA	4	8	1	NA	NA
Skin-Melanoma	9e0009d1-c993-4247-9706-88ee84591dec	5	46074168	46077330	14	3162	2	4	7	1	NA	NA
Skin-Melanoma	9e0009d1-c993-4247-9706-88ee84591dec	8	53637052	53639828	6	2776	1	4	1	NA	NA	NA
Skin-Melanoma	9e0009d1-c993-4247-9706-88ee84591dec	8	57568076	57573927	24	5851	3	14	7	NA	NA	NA
Skin-Melanoma	9e0009d1-c993-4247-9706-88ee84591dec	8	62071019	62074580	10	3561	NA	7	3	NA	NA	NA
Skin-Melanoma	9e0009d1-c993-4247-9706-88ee84591dec	8	69714053	69722549	29	8496	3	3	23	NA	NA	NA
Skin-Melanoma	9e0009d1-c993-4247-9706-88ee84591dec	11	23801386	23804328	7	2942	1	2	4	NA	NA	NA
Skin-Melanoma	9e0009d1-c993-4247-9706-88ee84591dec	11	23869802	23871906	15	2104	1	12	2	NA	NA	NA
Skin-Melanoma	9e0009d1-c993-4247-9706-88ee84591dec	11	27507092	27512059	13	4967	1	10	2	NA	NA	NA
Skin-Melanoma	9e0009d1-c993-4247-9706-88ee84591dec	11	37505316	37508807	8	3491	NA	3	5	NA	NA	NA
Skin-Melanoma	9e0009d1-c993-4247-9706-88ee84591dec	11	37838680	37840436	7	1756	1	6	NA	NA	NA	NA
Skin-Melanoma	9e0009d1-c993-4247-9706-88ee84591dec	11	68321664	68325332	11	3668	4	2	5	NA	NA	NA
Skin-Melanoma	9e0009d1-c993-4247-9706-88ee84591dec	11	69591289	69595736	16	4447	5	2	8	NA	1	NA
Skin-Melanoma	9e0009d1-c993-4247-9706-88ee84591dec	11	69692133	69698082	10	5949	NA	1	9	NA	NA	NA
Skin-Melanoma	9e0009d1-c993-4247-9706-88ee84591dec	11	69959793	69964171	9	4378	2	3	4	NA	NA	NA
Skin-Melanoma	9e0009d1-c993-4247-9706-88ee84591dec	11	72280491	72285084	8	4593	1	2	5	NA	NA	NA
Skin-Melanoma	9e0009d1-c993-4247-9706-88ee84591dec	11	72435936	72438636	18	2700	4	3	10	NA	NA	1
Skin-Melanoma	9e0009d1-c993-4247-9706-88ee84591dec	11	74338314	74344517	8	6203	1	3	4	NA	NA	NA
Skin-Melanoma	9e0009d1-c993-4247-9706-88ee84591dec	11	76110637	76113656	7	3019	NA	3	4	NA	NA	NA
Skin-Melanoma	9e0009d1-c993-4247-9706-88ee84591dec	11	76519886	76527531	10	7645	NA	5	5	NA	NA	NA
Skin-Melanoma	9e0009d1-c993-4247-9706-88ee84591dec	11	76529897	76534298	7	4401	NA	3	4	NA	NA	NA
Skin-Melanoma	9e0009d1-c993-4247-9706-88ee84591dec	11	81782986	81785482	7	2496	NA	7	NA	NA	NA	NA
Skin-Melanoma	9e0009d1-c993-4247-9706-88ee84591dec	11	82162771	82169042	13	6271	3	5	5	NA	NA	NA
Skin-Melanoma	9e0009d1-c993-4247-9706-88ee84591dec	11	84725993	84730948	7	4955	NA	2	5	NA	NA	NA
Skin-Melanoma	9e0009d1-c993-4247-9706-88ee84591dec	12	67952864	67953983	9	1119	NA	7	2	NA	NA	NA
Skin-Melanoma	9e0009d1-c993-4247-9706-88ee84591dec	12	71222785	71224572	27	1787	3	3	21	NA	NA	NA
Skin-Melanoma	9e0009d1-c993-4247-9706-88ee84591dec	13	49992609	49996841	11	4232	1	7	3	NA	NA	NA
Skin-Melanoma	9e0009d1-c993-4247-9706-88ee84591dec	15	26324368	26327957	11	3589	1	3	7	NA	NA	NA
Skin-Melanoma	9e0009d1-c993-4247-9706-88ee84591dec	15	55824044	55827531	8	3487	NA	2	6	NA	NA	NA
Skin-Melanoma	9e0009d1-c993-4247-9706-88ee84591dec	16	65117615	65122465	15	4850	2	1	12	NA	NA	NA
Skin-Melanoma	9e0009d1-c993-4247-9706-88ee84591dec	17	17800937	17807974	9	7037	NA	5	4	NA	NA	NA
Skin-Melanoma	9e0009d1-c993-4247-9706-88ee84591dec	17	30991100	30994226	11	3126	3	2	6	NA	NA	NA
Skin-Melanoma	9e0009d1-c993-4247-9706-88ee84591dec	17	35040681	35051324	16	10643	NA	11	4	NA	1	NA
Skin-Melanoma	9e0009d1-c993-4247-9706-88ee84591dec	19	33182209	33182541	9	332	2	3	4	NA	NA	NA
Skin-Melanoma	9e0009d1-c993-4247-9706-88ee84591dec	21	16127282	16131114	7	3832	NA	5	2	NA	NA	NA
Skin-Melanoma	9e0009d1-c993-4247-9706-88ee84591dec	21	17425198	17429635	14	4437	NA	10	4	NA	NA	NA
Prost-AdenoCA	9e272bea-9193-4c10-a802-f7e18b2937a3	5	96649159	96650210	10	1051	1	3	6	NA	NA	NA
Prost-AdenoCA	9e272bea-9193-4c10-a802-f7e18b2937a3	5	97257903	97267328	17	9425	NA	8	9	NA	NA	NA
Prost-AdenoCA	9e272bea-9193-4c10-a802-f7e18b2937a3	5	98187488	98193540	41	6052	2	15	24	NA	NA	NA
Prost-AdenoCA	9e272bea-9193-4c10-a802-f7e18b2937a3	5	101260492	101265502	19	5010	NA	10	9	NA	NA	NA
Prost-AdenoCA	9e272bea-9193-4c10-a802-f7e18b2937a3	7	15853026	15854251	16	1225	2	6	8	NA	NA	NA

Prost-AdenoCA	9e272bea-9193-4c10-a802-f7e18b2937a3	7	123518632	123524010	7	5378	1	5	1	NA	NA	NA
Prost-AdenoCA	9e272bea-9193-4c10-a802-f7e18b2937a3	8	43515218	43517230	15	2012	2	10	3	NA	NA	NA
Prost-AdenoCA	9e272bea-9193-4c10-a802-f7e18b2937a3	12	16014045	16014562	8	517	NA	NA	NA	5	3	NA
Prost-AdenoCA	9e272bea-9193-4c10-a802-f7e18b2937a3	12	16946870	16947568	10	698	NA	1	NA	1	8	NA
ColoRect-AdenoCA	9e5f0a23-e184-4611-b42b-e882dcf23acd	1	157602620	157604177	9	1557	NA	NA	1	8	NA	NA
ColoRect-AdenoCA	9e5f0a23-e184-4611-b42b-e882dcf23acd	4	178456464	178461622	7	5158	2	2	2	1	NA	NA
ColoRect-AdenoCA	9e5f0a23-e184-4611-b42b-e882dcf23acd	8	32229287	32233402	11	4115	3	4	4	NA	NA	NA
Bone-Leiomyo	9e7b51fb-2249-451a-bf23-bb2a3d935928	1	6001660	6001859	9	199	3	2	4	NA	NA	NA
Bone-Leiomyo	9e7b51fb-2249-451a-bf23-bb2a3d935928	1	6161362	6163009	13	1647	2	6	4	NA	NA	1
Bone-Leiomyo	9e7b51fb-2249-451a-bf23-bb2a3d935928	1	188815515	188816200	9	685	1	3	5	NA	NA	NA
Bone-Leiomyo	9e7b51fb-2249-451a-bf23-bb2a3d935928	2	15409	18532	13	3123	NA	12	1	NA	NA	NA
Bone-Leiomyo	9e7b51fb-2249-451a-bf23-bb2a3d935928	2	103216514	103217939	9	1425	2	3	4	NA	NA	NA
Bone-Leiomyo	9e7b51fb-2249-451a-bf23-bb2a3d935928	2	124757829	124770188	22	12359	4	12	6	NA	NA	NA
Bone-Leiomyo	9e7b51fb-2249-451a-bf23-bb2a3d935928	2	142227225	142230297	12	3072	2	6	4	NA	NA	NA
Bone-Leiomyo	9e7b51fb-2249-451a-bf23-bb2a3d935928	3	23383276	23386016	8	2740	NA	3	5	NA	NA	NA
Bone-Leiomyo	9e7b51fb-2249-451a-bf23-bb2a3d935928	4	39293519	39294865	6	1346	NA	2	4	NA	NA	NA
Bone-Leiomyo	9e7b51fb-2249-451a-bf23-bb2a3d935928	4	70666609	70667970	13	1361	3	6	4	NA	NA	NA
Bone-Leiomyo	9e7b51fb-2249-451a-bf23-bb2a3d935928	11	30731134	30733919	11	2785	1	4	6	NA	NA	NA
Bone-Leiomyo	9e7b51fb-2249-451a-bf23-bb2a3d935928	11	33671187	33676187	15	5000	3	10	2	NA	NA	NA
Bone-Leiomyo	9e7b51fb-2249-451a-bf23-bb2a3d935928	14	32102169	32103005	9	836	2	5	2	NA	NA	NA
Bone-Leiomyo	9e7b51fb-2249-451a-bf23-bb2a3d935928	15	57323633	57339400	24	15767	4	16	4	NA	NA	NA
Bone-Leiomyo	9e7b51fb-2249-451a-bf23-bb2a3d935928	15	87173593	87177998	14	4405	5	6	3	NA	NA	NA
Bone-Leiomyo	9e7b51fb-2249-451a-bf23-bb2a3d935928	18	255061	261741	11	6680	4	4	3	NA	NA	NA
Bone-Leiomyo	9e7b51fb-2249-451a-bf23-bb2a3d935928	18	355938	371676	24	15738	6	10	8	NA	NA	NA
Bone-Leiomyo	9e7b51fb-2249-451a-bf23-bb2a3d935928	18	10965461	10977186	35	11725	4	20	10	1	NA	NA
Bone-Leiomyo	9e7b51fb-2249-451a-bf23-bb2a3d935928	18	40653952	40656091	13	2139	2	5	6	NA	NA	NA
Bone-Leiomyo	9e7b51fb-2249-451a-bf23-bb2a3d935928	21	23191665	23193141	7	1476	NA	1	6	NA	NA	NA
Bone-Leiomyo	9e7b51fb-2249-451a-bf23-bb2a3d935928	Y	23317077	23317855	7	778	NA	5	1	NA	NA	1
Lymph-BNHL	9e842227-2bc6-4185-bedb-abeb03836b51	1	239876976	239881296	6	4320	NA	NA	2	3	NA	1
Lymph-BNHL	9e842227-2bc6-4185-bedb-abeb03836b51	2	89155721	89160403	89	4682	3	9	24	32	9	12
Lymph-BNHL	9e842227-2bc6-4185-bedb-abeb03836b51	2	136874837	136875306	8	469	NA	1	3	2	2	NA
Lymph-BNHL	9e842227-2bc6-4185-bedb-abeb03836b51	3	187461700	187468084	11	6384	NA	3	7	1	NA	NA
Lymph-BNHL	9e842227-2bc6-4185-bedb-abeb03836b51	12	86683411	86700952	25	17541	2	9	4	4	2	4
Lymph-BNHL	9e842227-2bc6-4185-bedb-abeb03836b51	13	38927142	38943935	23	16793	NA	NA	2	9	5	7
Lymph-BNHL	9e842227-2bc6-4185-bedb-abeb03836b51	14	106069127	106071055	9	1928	1	2	5	NA	NA	1
Lymph-BNHL	9e842227-2bc6-4185-bedb-abeb03836b51	14	106151157	106153334	8	2177	NA	NA	6	1	NA	1
Lymph-BNHL	9e842227-2bc6-4185-bedb-abeb03836b51	14	106212568	106213827	12	1259	1	2	8	1	NA	NA
Lymph-BNHL	9e842227-2bc6-4185-bedb-abeb03836b51	14	106323858	106329230	85	5372	2	16	31	17	8	11
Lymph-BNHL	9e842227-2bc6-4185-bedb-abeb03836b51	14	106815652	106816789	8	1137	NA	1	5	NA	2	NA
Lymph-BNHL	9e842227-2bc6-4185-bedb-abeb03836b51	18	60983828	60988357	51	4529	3	6	29	4	5	4
Lymph-BNHL	9e842227-2bc6-4185-bedb-abeb03836b51	22	23203674	23206325	6	2651	NA	1	4	NA	1	NA
Lymph-BNHL	9e842227-2bc6-4185-bedb-abeb03836b51	22	23223439	23283439	62	60000	5	12	29	8	6	2
Panc-AdenoCA	9ebac79d-8b38-4469-837e-b834725fe6d5	19	32349427	32349700	6	273	1	2	3	NA	NA	NA
Liver-HCC	9ec31146-c622-11e3-bf01-24c6515278c0	8	40141513	40142661	7	1148	NA	NA	NA	2	4	1
Head-SCC	9fb6b7be-0084-48f7-a256-6d170e72e97f	2	175733063	175738985	14	5922	NA	6	8	NA	NA	NA
Cervix-SCC	9ff21093-58d7-4b69-aade-c242a383ea56	3	113901416	113902321	7	905	NA	1	6	NA	NA	NA
Cervix-SCC	9ff21093-58d7-4b69-aade-c242a383ea56	3	169358728	169360052	7	1324	4	2	1	NA	NA	NA
Cervix-SCC	9ff21093-58d7-4b69-aade-c242a383ea56	21	17800146	17806964	8	6818	1	2	5	NA	NA	NA
Cervix-SCC	9ff21093-58d7-4b69-aade-c242a383ea56	X	58556014	58557351	9	1337	1	1	2	NA	3	2
Panc-AdenoCA	9ffe694e-b488-489e-bdbe-0800e505eec4	8	8550396	8550562	6	166	NA	2	4	NA	NA	NA
Skin-Melanoma	a0583407-712d-46f3-8c4e-4eeaa0149087	1	4116886	4117117	6	231	NA	NA	6	NA	NA	NA
Skin-Melanoma	a0583407-712d-46f3-8c4e-4eeaa0149087	8	107850011	107850799	6	788	NA	NA	1	1	2	2
Skin-Melanoma	a0583407-712d-46f3-8c4e-4eeaa0149087	12	40561199	40563234	7	2035	2	2	3	NA	NA	NA
Skin-Melanoma	a0583407-712d-46f3-8c4e-4eeaa0149087	12	45981834	45986149	9	4315	NA	4	4	1	NA	NA
Skin-Melanoma	a0583407-712d-46f3-8c4e-4eeaa0149087	12	51342308	51352497	13	10189	1	5	7	NA	NA	NA
Skin-Melanoma	a0583407-712d-46f3-8c4e-4eeaa0149087	12	59480612	59481380	14	768	5	4	5	NA	NA	NA
Prost-AdenoCA	a08ec059-7592-4698-bb45-25a9c3680c23	5	27678518	27681438	6	2920	NA	NA	1	5	NA	NA
Ovary-AdenoCA	a0bbb3b1-e774-4c75-9301-ba43fb803f20	8	95726439	95732540	18	6101	NA	3	15	NA	NA	NA
Ovary-AdenoCA	a0bbb3b1-e774-4c75-9301-ba43fb803f20	8	123021363	123028645	12	7282	1	6	3	NA	NA	2
ColoRect-AdenoCA	a0f94565-9169-49ee-8e6a-21bdc43d9de6	20	6281555	6281564	7	9	NA	2	1	1	2	1

Head-SCC	a0fe80e0-e3ac-485b-8a1a-4c80c101b874	3	106400424	106404717	23	4293	2	16	5	NA	NA	NA
Head-SCC	a0fe80e0-e3ac-485b-8a1a-4c80c101b874	11	69992450	69998235	15	5785	3	4	8	NA	NA	NA
Head-SCC	a0fe80e0-e3ac-485b-8a1a-4c80c101b874	11	122036813	122037172	7	359	6	NA	1	NA	NA	NA
Head-SCC	a0fe80e0-e3ac-485b-8a1a-4c80c101b874	19	5906225	5907497	16	1272	2	4	10	NA	NA	NA
Head-SCC	a0fe80e0-e3ac-485b-8a1a-4c80c101b874	19	11883603	11885948	13	2345	4	7	2	NA	NA	NA
Liver-HCC	a1a75f66-c622-11e3-bf01-24c6515278c0	21	23820680	23822444	10	1764	2	6	2	NA	NA	NA
Prost-AdenoCA	a1af7c42-47af-435c-bb51-b932a3cd7b91	9	37833566	37834635	7	1069	1	4	2	NA	NA	NA
Prost-AdenoCA	a1af7c42-47af-435c-bb51-b932a3cd7b91	18	36338875	36342117	11	3242	NA	4	7	NA	NA	NA
Eso-AdenoCa	a1e3dc5b-b81f-4890-870c-ed3b8ac36dec	1	107885964	107888374	16	2410	4	8	4	NA	NA	NA
Eso-AdenoCa	a1e3dc5b-b81f-4890-870c-ed3b8ac36dec	11	56100569	56104067	10	3498	1	4	5	NA	NA	NA
Eso-AdenoCa	a1e3dc5b-b81f-4890-870c-ed3b8ac36dec	12	68823536	68825659	10	2123	NA	NA	10	NA	NA	NA
Ovary-AdenoCA	a1fc7064-abf5-477d-983e-576a784e1d40	19	40844762	40845712	10	950	3	6	NA	NA	1	NA
Ovary-AdenoCA	a1fc7064-abf5-477d-983e-576a784e1d40	X	567289	568719	8	1430	2	4	2	NA	NA	NA
Panc-AdenoCA	a2238b5f-0513-487a-9b1b-7bef85ebf336	13	41424259	41427287	9	3028	NA	8	1	NA	NA	NA
Panc-AdenoCA	a2238b5f-0513-487a-9b1b-7bef85ebf336	13	41524609	41525410	8	801	NA	4	4	NA	NA	NA
Panc-AdenoCA	a2238b5f-0513-487a-9b1b-7bef85ebf336	17	13267652	13269661	8	2009	1	6	1	NA	NA	NA
Panc-AdenoCA	a2238b5f-0513-487a-9b1b-7bef85ebf336	X	77766392	77766877	6	485	NA	5	1	NA	NA	NA
Lymph-BNHL	a29278af-7ecf-403e-b6a9-623ea7879d05	1	105865667	105869719	7	4052	NA	NA	2	2	1	2
Lymph-BNHL	a29278af-7ecf-403e-b6a9-623ea7879d05	1	203274945	203276256	12	1311	1	7	3	NA	1	NA
Lymph-BNHL	a29278af-7ecf-403e-b6a9-623ea7879d05	2	89127809	89134634	8	6825	NA	NA	1	3	NA	4
Lymph-BNHL	a29278af-7ecf-403e-b6a9-623ea7879d05	2	89137355	89246976	246	1E+05	6	34	43	84	35	44
Lymph-BNHL	a29278af-7ecf-403e-b6a9-623ea7879d05	2	146445889	146446480	7	591	2	NA	NA	3	2	NA
Lymph-BNHL	a29278af-7ecf-403e-b6a9-623ea7879d05	3	187460510	187470856	50	10346	5	14	11	12	4	4
Lymph-BNHL	a29278af-7ecf-403e-b6a9-623ea7879d05	3	187957814	187959747	7	1933	NA	1	3	2	1	NA
Lymph-BNHL	a29278af-7ecf-403e-b6a9-623ea7879d05	3	188468341	188472190	7	3849	NA	1	NA	2	1	3
Lymph-BNHL	a29278af-7ecf-403e-b6a9-623ea7879d05	4	47207	51222	9	4015	NA	1	3	1	2	2
Lymph-BNHL	a29278af-7ecf-403e-b6a9-623ea7879d05	4	59045342	59050831	7	5489	NA	NA	2	2	1	2
Lymph-BNHL	a29278af-7ecf-403e-b6a9-623ea7879d05	4	59067868	59069233	7	1365	NA	NA	1	2	3	1
Lymph-BNHL	a29278af-7ecf-403e-b6a9-623ea7879d05	6	91005484	91006032	6	548	NA	1	3	2	NA	NA
Lymph-BNHL	a29278af-7ecf-403e-b6a9-623ea7879d05	6	134494230	134501286	12	7056	2	3	4	2	NA	1
Lymph-BNHL	a29278af-7ecf-403e-b6a9-623ea7879d05	7	40846316	40849242	8	2926	NA	2	NA	2	4	NA
Lymph-BNHL	a29278af-7ecf-403e-b6a9-623ea7879d05	8	73230461	73232267	6	1806	NA	NA	NA	4	1	1
Lymph-BNHL	a29278af-7ecf-403e-b6a9-623ea7879d05	13	86308931	86311815	7	2884	NA	NA	1	2	2	2
Lymph-BNHL	a29278af-7ecf-403e-b6a9-623ea7879d05	14	106055794	106057796	12	2002	NA	4	8	NA	NA	NA
Lymph-BNHL	a29278af-7ecf-403e-b6a9-623ea7879d05	14	106173564	106175884	20	2320	5	2	8	2	2	1
Lymph-BNHL	a29278af-7ecf-403e-b6a9-623ea7879d05	14	106326944	106366865	126	39921	7	16	50	21	15	17
Lymph-BNHL	a29278af-7ecf-403e-b6a9-623ea7879d05	16	64355625	64356183	6	558	NA	1	1	4	NA	NA
Lymph-BNHL	a29278af-7ecf-403e-b6a9-623ea7879d05	19	10340746	10341618	6	872	1	2	2	NA	NA	1
Lymph-BNHL	a29278af-7ecf-403e-b6a9-623ea7879d05	22	23227173	23282947	186	55774	13	27	57	47	23	19
Stomach-AdenoCA	a2e67393-50d7-4bcc-9bbd-c695086a936a	12	67282174	67283291	10	1117	1	7	2	NA	NA	NA
Stomach-AdenoCA	a2e67393-50d7-4bcc-9bbd-c695086a936a	21	17205874	17207963	8	2089	1	6	1	NA	NA	NA
Bone-Leiomyo	a2f02ff7-8433-4cb5-9324-34f13edeaca1	3	168114450	168115338	15	888	NA	7	8	NA	NA	NA
Bone-Leiomyo	a2f02ff7-8433-4cb5-9324-34f13edeaca1	3	174069760	174075403	11	5643	NA	5	6	NA	NA	NA
Bone-Leiomyo	a2f02ff7-8433-4cb5-9324-34f13edeaca1	6	44145221	44146189	9	968	NA	NA	9	NA	NA	NA
Bone-Leiomyo	a2f02ff7-8433-4cb5-9324-34f13edeaca1	11	20475262	20476120	6	858	NA	2	4	NA	NA	NA
Bone-Leiomyo	a2f02ff7-8433-4cb5-9324-34f13edeaca1	12	69311201	69323968	21	12767	2	1	17	NA	1	NA
Bone-Leiomyo	a2f02ff7-8433-4cb5-9324-34f13edeaca1	12	71200803	71208696	9	7893	1	5	3	NA	NA	NA
Bone-Leiomyo	a2f02ff7-8433-4cb5-9324-34f13edeaca1	12	87168281	87168731	7	450	NA	3	4	NA	NA	NA
Bone-Leiomyo	a2f02ff7-8433-4cb5-9324-34f13edeaca1	12	89158535	89166088	16	7553	NA	2	14	NA	NA	NA
Bone-Leiomyo	a2f02ff7-8433-4cb5-9324-34f13edeaca1	19	28935523	28937178	7	1655	NA	3	4	NA	NA	NA
Bone-Leiomyo	a2f02ff7-8433-4cb5-9324-34f13edeaca1	19	29475664	29480055	7	4391	NA	NA	7	NA	NA	NA
Bone-Leiomyo	a2f02ff7-8433-4cb5-9324-34f13edeaca1	21	35309632	35315408	8	5776	2	4	2	NA	NA	NA
Liver-HCC	a2f143d6-ba07-4ff0-960d-b29c3c716665	13	58683345	58683352	6	7	NA	NA	NA	3	NA	3
Ovary-AdenoCA	a3135834-3af0-4e98-bc6f-ad8ddf33db80	4	167631328	167632363	7	1035	4	NA	1	NA	NA	2
Ovary-AdenoCA	a3135834-3af0-4e98-bc6f-ad8ddf33db80	7	77889768	77891757	17	1989	5	3	9	NA	NA	NA
Head-SCC	a315b798-f037-48d5-91ed-e733e3bbd6ee	3	174756200	174760536	11	4336	2	3	5	NA	NA	1
Head-SCC	a315b798-f037-48d5-91ed-e733e3bbd6ee	4	43371936	43376446	8	4510	2	5	1	NA	NA	NA
Head-SCC	a315b798-f037-48d5-91ed-e733e3bbd6ee	6	64711707	64715017	11	3310	1	3	7	NA	NA	NA
Head-SCC	a315b798-f037-48d5-91ed-e733e3bbd6ee	10	85413288	85414782	11	1494	2	7	2	NA	NA	NA
Head-SCC	a315b798-f037-48d5-91ed-e733e3bbd6ee	10	85429420	85432181	23	2761	2	7	14	NA	NA	NA

Head-SCC	a315b798-f037-48d5-91ed-e733e3bbd6ee	10	94769556	94772000	15	2444	NA	2	13	NA	NA	NA
Head-SCC	a315b798-f037-48d5-91ed-e733e3bbd6ee	11	99730979	99732396	7	1417	NA	6	1	NA	NA	NA
Panc-AdenoCA	a3210fd0-344c-468e-8ff2-2d0869a2fb75	1	95997307	95997571	8	264	1	2	5	NA	NA	NA
Panc-AdenoCA	a3210fd0-344c-468e-8ff2-2d0869a2fb75	3	177436140	177441481	9	5341	1	4	4	NA	NA	NA
Panc-AdenoCA	a3210fd0-344c-468e-8ff2-2d0869a2fb75	6	21237202	21241478	12	4276	2	5	5	NA	NA	NA
Panc-AdenoCA	a3210fd0-344c-468e-8ff2-2d0869a2fb75	7	103037073	103038333	7	1260	2	3	2	NA	NA	NA
Panc-AdenoCA	a3210fd0-344c-468e-8ff2-2d0869a2fb75	8	98407092	98407934	6	842	NA	1	5	NA	NA	NA
Panc-AdenoCA	a3210fd0-344c-468e-8ff2-2d0869a2fb75	14	21762341	21766586	16	4245	1	7	8	NA	NA	NA
Panc-AdenoCA	a3210fd0-344c-468e-8ff2-2d0869a2fb75	15	76910509	76913066	8	2557	NA	5	3	NA	NA	NA
Panc-AdenoCA	a3210fd0-344c-468e-8ff2-2d0869a2fb75	19	45382426	45383974	6	1548	1	2	3	NA	NA	NA
Panc-AdenoCA	a3210fd0-344c-468e-8ff2-2d0869a2fb75	20	62339489	62344376	11	4887	2	3	6	NA	NA	NA
Panc-AdenoCA	a3210fd0-344c-468e-8ff2-2d0869a2fb75	21	33289535	33292378	8	2843	1	3	4	NA	NA	NA
Panc-AdenoCA	a3210fd0-344c-468e-8ff2-2d0869a2fb75	X	46009889	46013605	7	3716	NA	2	4	NA	NA	1
Ovary-AdenoCA	a330a96e-9897-4605-b5f1-5b5ef45cd365	2	24916752	24920583	10	3831	NA	7	3	NA	NA	NA
Ovary-AdenoCA	a330a96e-9897-4605-b5f1-5b5ef45cd365	3	162488623	162493554	26	4931	2	9	15	NA	NA	NA
Eso-AdenoCa	a37a825c-d74e-46cc-92ef-b65016857b4a	5	122105022	122107205	6	2183	1	NA	5	NA	NA	NA
Eso-AdenoCa	a37a825c-d74e-46cc-92ef-b65016857b4a	8	140987660	140987735	7	75	NA	1	4	NA	2	NA
Eso-AdenoCa	a37a825c-d74e-46cc-92ef-b65016857b4a	9	117818471	117820342	6	1871	3	2	1	NA	NA	NA
Liver-HCC	a3914a6c-c622-11e3-bf01-24c6515278c0	8	87047066	87047946	7	880	NA	4	2	NA	1	NA
Liver-HCC	a3914a6c-c622-11e3-bf01-24c6515278c0	8	97402528	97406707	7	4179	1	1	5	NA	NA	NA
Biliary-AdenoCA	a47c2012-c13d-48ac-88b6-e09bfd50122b	2	61886283	61889361	7	3078	NA	6	1	NA	NA	NA
Biliary-AdenoCA	a47c2012-c13d-48ac-88b6-e09bfd50122b	6	165419491	165425537	24	6046	2	10	12	NA	NA	NA
Biliary-AdenoCA	a47c2012-c13d-48ac-88b6-e09bfd50122b	10	93870517	93874072	8	3555	1	1	6	NA	NA	NA
Biliary-AdenoCA	a47c2012-c13d-48ac-88b6-e09bfd50122b	16	69491385	69496319	7	4934	NA	4	3	NA	NA	NA
Panc-AdenoCA	a492a32b-bf2f-4f8c-b06e-794a4baa2fe7	15	58664349	58664440	6	91	NA	1	5	NA	NA	NA
Stomach-AdenoCA	a4c838df-6ac4-455a-8096-8ec680064374	22	29069107	29069310	10	203	NA	NA	NA	10	NA	NA
Kidney-RCC	a4f8305f-4409-4776-b34d-4878d18c8693	12	56305678	56306451	7	773	NA	4	3	NA	NA	NA
Panc-AdenoCA	a5346247-3f30-470a-b4ab-732180786291	17	38088668	38090512	8	1844	1	2	5	NA	NA	NA
Kidney-RCC	a56cd282-f6c5-4373-b3d8-676562894598	3	80369864	80373797	7	3933	NA	NA	7	NA	NA	NA
Kidney-RCC	a56cd282-f6c5-4373-b3d8-676562894598	3	80967652	80975679	10	8027	NA	NA	10	NA	NA	NA
Panc-AdenoCA	a5d5c238-0447-47fa-9a81-3b823cbdcea6	12	68339613	68341904	12	2291	2	3	7	NA	NA	NA
Panc-AdenoCA	a5d5c238-0447-47fa-9a81-3b823cbdcea6	15	87003901	87005330	7	1429	NA	1	6	NA	NA	NA
Panc-AdenoCA	a5d5c238-0447-47fa-9a81-3b823cbdcea6	17	79212339	79218135	16	5796	3	5	8	NA	NA	NA
Panc-AdenoCA	a5d5c238-0447-47fa-9a81-3b823cbdcea6	18	20753606	20753927	6	321	1	1	4	NA	NA	NA
Panc-AdenoCA	a5d5c238-0447-47fa-9a81-3b823cbdcea6	18	22276753	22277994	17	1241	3	4	10	NA	NA	NA
Panc-AdenoCA	a5d5c238-0447-47fa-9a81-3b823cbdcea6	18	30379442	30383607	10	4165	NA	1	9	NA	NA	NA
Liver-HCC	a612b654-c622-11e3-bf01-24c6515278c0	3	120155135	120157750	20	2615	2	5	9	2	2	NA
Liver-HCC	a612b654-c622-11e3-bf01-24c6515278c0	3	120710874	120714974	9	4100	1	5	3	NA	NA	NA
ColoRect-AdenoCA	a64000df-16e5-4b88-865f-5c680adf60eb	8	38945419	38946963	6	1544	NA	5	1	NA	NA	NA
Cervix-SCC	a67f4531-99ef-43df-82f5-f6abc4b11826	2	27270683	27286981	32	16298	4	6	22	NA	NA	NA
Cervix-SCC	a67f4531-99ef-43df-82f5-f6abc4b11826	14	81715039	81719749	8	4710	NA	3	5	NA	NA	NA
Cervix-SCC	a67f4531-99ef-43df-82f5-f6abc4b11826	15	68008961	68014065	9	5104	2	1	6	NA	NA	NA
Cervix-SCC	a67f4531-99ef-43df-82f5-f6abc4b11826	19	45258763	45265712	15	6949	2	6	7	NA	NA	NA
CNS-GBM	a6957d15-453c-4f3b-ab8c-c38054dfe548	6	133960332	133961606	8	1274	1	5	1	NA	1	NA
CNS-GBM	a6957d15-453c-4f3b-ab8c-c38054dfe548	12	57894776	57896232	6	1456	1	1	4	NA	NA	NA
CNS-GBM	a6957d15-453c-4f3b-ab8c-c38054dfe548	12	61754273	61756480	8	2207	1	6	1	NA	NA	NA
CNS-GBM	a6957d15-453c-4f3b-ab8c-c38054dfe548	12	62078944	62086082	9	7138	3	5	1	NA	NA	NA
CNS-GBM	a6957d15-453c-4f3b-ab8c-c38054dfe548	12	71802824	71809012	8	6188	2	5	1	NA	NA	NA
CNS-GBM	a6957d15-453c-4f3b-ab8c-c38054dfe548	12	85556044	85557661	10	1617	2	5	1	1	1	NA
Breast-AdenoCa	a6c14ebc-81a2-44f7-af87-2b7ec85e5696	1	178600775	178601074	6	299	NA	3	3	NA	NA	NA
Breast-AdenoCa	a6c14ebc-81a2-44f7-af87-2b7ec85e5696	1	182605716	182609364	12	3648	NA	5	7	NA	NA	NA
Breast-AdenoCa	a6c14ebc-81a2-44f7-af87-2b7ec85e5696	1	206691314	206691962	8	648	2	1	5	NA	NA	NA
Breast-AdenoCa	a6c14ebc-81a2-44f7-af87-2b7ec85e5696	5	176919445	176921246	11	1801	2	5	4	NA	NA	NA
Breast-AdenoCa	a6c14ebc-81a2-44f7-af87-2b7ec85e5696	10	36805549	36807291	7	1742	NA	2	5	NA	NA	NA
Breast-AdenoCa	a6c14ebc-81a2-44f7-af87-2b7ec85e5696	11	76701555	76702213	7	658	NA	4	3	NA	NA	NA
Breast-AdenoCa	a6c14ebc-81a2-44f7-af87-2b7ec85e5696	11	84749382	84753756	10	4374	NA	5	5	NA	NA	NA
Breast-AdenoCa	a6c14ebc-81a2-44f7-af87-2b7ec85e5696	13	24123033	24124530	7	1497	NA	NA	7	NA	NA	NA
Breast-AdenoCa	a6c14ebc-81a2-44f7-af87-2b7ec85e5696	17	35347865	35358168	15	10303	NA	1	13	1	NA	NA
Skin-Melanoma	a6e24c2a-26b9-409c-bbcd-a8fcdd1e437c	2	39401088	39408601	10	7513	NA	6	4	NA	NA	NA
Skin-Melanoma	a6e24c2a-26b9-409c-bbcd-a8fcdd1e437c	2	85108641	85110401	7	1760	2	4	1	NA	NA	NA

Skin-Melanoma	a6e24c2a-26b9-409c-bbcd-a8fcdd1e437c	2	86999677	87000499	7	822	NA	1	5	1	NA	NA
Skin-Melanoma	a6e24c2a-26b9-409c-bbcd-a8fcdd1e437c	3	110189152	110190468	8	1316	NA	7	1	NA	NA	NA
Skin-Melanoma	a6e24c2a-26b9-409c-bbcd-a8fcdd1e437c	10	89396866	89397782	9	916	NA	NA	9	NA	NA	NA
Skin-Melanoma	a6e24c2a-26b9-409c-bbcd-a8fcdd1e437c	20	8313053	8314844	31	1791	2	1	28	NA	NA	NA
Panc-AdenoCA	a6ebe0c0-8aab-4b9f-8328-4b795895a77d	5	103305234	103305247	8	13	1	NA	NA	NA	1	6
Panc-AdenoCA	a6ebe0c0-8aab-4b9f-8328-4b795895a77d	6	154569256	154569734	6	478	NA	3	3	NA	NA	NA
Panc-AdenoCA	a6ebe0c0-8aab-4b9f-8328-4b795895a77d	6	156557950	156558259	12	309	1	7	4	NA	NA	NA
Panc-AdenoCA	a6ebe0c0-8aab-4b9f-8328-4b795895a77d	6	159186028	159191746	7	5718	2	2	3	NA	NA	NA
Panc-AdenoCA	a6ebe0c0-8aab-4b9f-8328-4b795895a77d	16	33578945	33587252	14	8307	3	1	1	3	3	3
Panc-AdenoCA	a6ebe0c0-8aab-4b9f-8328-4b795895a77d	19	28684597	28685682	6	1085	1	3	2	NA	NA	NA
CNS-Oligo	a78544d7-65c6-4778-af62-ceec24c14056	12	43661071	43661699	6	628	NA	6	NA	NA	NA	NA
Panc-AdenoCA	a7a56d67-1bd7-4d69-9237-34c2d261261b	3	134897227	134897250	12	23	2	3	2	2	2	1
Panc-AdenoCA	a7a56d67-1bd7-4d69-9237-34c2d261261b	8	140122513	140122531	6	18	1	1	NA	2	NA	2
Head-SCC	a8041e86-071e-46cd-8fb5-7ecdc1ab0553	2	26198025	26206647	13	8622	1	3	9	NA	NA	NA
Head-SCC	a8041e86-071e-46cd-8fb5-7ecdc1ab0553	8	37920656	37924070	8	3414	1	2	5	NA	NA	NA
Head-SCC	a8041e86-071e-46cd-8fb5-7ecdc1ab0553	8	37952481	37979859	29	27378	2	10	17	NA	NA	NA
Head-SCC	a8041e86-071e-46cd-8fb5-7ecdc1ab0553	8	38020443	38031036	12	10593	1	8	3	NA	NA	NA
Head-SCC	a8041e86-071e-46cd-8fb5-7ecdc1ab0553	8	38035073	38041751	8	6678	NA	6	2	NA	NA	NA
Head-SCC	a8041e86-071e-46cd-8fb5-7ecdc1ab0553	8	38049916	38055500	7	5584	NA	2	5	NA	NA	NA
Head-SCC	a8041e86-071e-46cd-8fb5-7ecdc1ab0553	8	38060471	38080774	39	20303	2	10	27	NA	NA	NA
Head-SCC	a8041e86-071e-46cd-8fb5-7ecdc1ab0553	10	123072756	123076113	9	3357	1	6	2	NA	NA	NA
Liver-HCC	a846f5e8-c622-11e3-bf01-24c6515278c0	4	31816701	31819906	9	3205	NA	NA	NA	4	NA	5
Liver-HCC	a846f5e8-c622-11e3-bf01-24c6515278c0	12	33761699	33763383	11	1684	NA	7	4	NA	NA	NA
Breast-AdenoCa	a85cf239-ff51-46e7-9b88-4c2cb49c66b9	6	138015769	138016952	6	1183	1	2	3	NA	NA	NA
Breast-AdenoCa	a85cf239-ff51-46e7-9b88-4c2cb49c66b9	10	4057985	4059424	7	1439	2	4	1	NA	NA	NA
Breast-AdenoCa	a85cf239-ff51-46e7-9b88-4c2cb49c66b9	X	86887944	86887951	6	7	NA	1	3	NA	NA	2
Breast-AdenoCa	a876398c-5b1d-444f-a360-5fe2db697480	11	96115608	96117312	9	1704	3	2	4	NA	NA	NA
Breast-AdenoCa	a876398c-5b1d-444f-a360-5fe2db697480	22	37756376	37758810	7	2434	2	1	2	NA	2	NA
Breast-AdenoCa	a876398c-5b1d-444f-a360-5fe2db697480	X	66889699	66890350	6	651	NA	NA	1	1	NA	4
Kidney-RCC	a9dbd55c-5dcc-48db-8785-6baef3fdd7db	16	34272356	34274389	6	2033	NA	3	2	NA	1	NA
Kidney-RCC	a9dbd55c-5dcc-48db-8785-6baef3fdd7db	16	64151091	64152307	12	1216	1	7	4	NA	NA	NA
ColoRect-AdenoCA	aa06ae46-2336-4666-a3af-fc790daf8da4	12	4186508	4186946	6	438	2	2	2	NA	NA	NA
ColoRect-AdenoCA	aa06ae46-2336-4666-a3af-fc790daf8da4	12	4663005	4668575	9	5570	2	3	4	NA	NA	NA
ColoRect-AdenoCA	aa06ae46-2336-4666-a3af-fc790daf8da4	12	6429094	6435420	12	6326	6	2	3	1	NA	NA
ColoRect-AdenoCA	aa06ae46-2336-4666-a3af-fc790daf8da4	12	6537666	6540831	9	3165	1	1	7	NA	NA	NA
ColoRect-AdenoCA	aa06ae46-2336-4666-a3af-fc790daf8da4	12	32724616	32725472	7	856	2	3	2	NA	NA	NA
ColoRect-AdenoCA	aa06ae46-2336-4666-a3af-fc790daf8da4	12	32919175	32923670	8	4495	4	2	2	NA	NA	NA
ColoRect-AdenoCA	aa06ae46-2336-4666-a3af-fc790daf8da4	12	49794728	49798716	8	3988	1	2	5	NA	NA	NA
ColoRect-AdenoCA	aa06ae46-2336-4666-a3af-fc790daf8da4	12	50684666	50685479	8	813	2	1	5	NA	NA	NA
ColoRect-AdenoCA	aa06ae46-2336-4666-a3af-fc790daf8da4	12	51168236	51174865	12	6629	3	7	2	NA	NA	NA
ColoRect-AdenoCA	aa06ae46-2336-4666-a3af-fc790daf8da4	12	57581720	57590395	13	8675	6	6	1	NA	NA	NA
ColoRect-AdenoCA	aa06ae46-2336-4666-a3af-fc790daf8da4	12	58082203	58083255	12	1052	3	3	6	NA	NA	NA
ColoRect-AdenoCA	aa06ae46-2336-4666-a3af-fc790daf8da4	12	93402174	93407253	8	5079	NA	2	6	NA	NA	NA
ColoRect-AdenoCA	aa06ae46-2336-4666-a3af-fc790daf8da4	12	112191771	112196101	8	4330	6	NA	2	NA	NA	NA
ColoRect-AdenoCA	aa06ae46-2336-4666-a3af-fc790daf8da4	12	112744619	112750486	11	5867	1	2	7	NA	NA	1
ColoRect-AdenoCA	aa06ae46-2336-4666-a3af-fc790daf8da4	12	122232575	122236979	7	4404	1	2	4	NA	NA	NA
ColoRect-AdenoCA	aa06ae46-2336-4666-a3af-fc790daf8da4	12	123637930	123643786	9	5856	3	3	1	NA	1	1
ColoRect-AdenoCA	aa06ae46-2336-4666-a3af-fc790daf8da4	12	132223505	132231095	11	7590	2	2	7	NA	NA	NA
ColoRect-AdenoCA	aa06ae46-2336-4666-a3af-fc790daf8da4	21	30521595	30522928	9	1333	2	5	2	NA	NA	NA
Panc-AdenoCA	aa4a868a-df23-4eef-a618-e945aa2ce98a	4	131265569	131267713	6	2144	NA	NA	6	NA	NA	NA
Panc-AdenoCA	aa4a868a-df23-4eef-a618-e945aa2ce98a	4	136152042	136152273	9	231	2	3	4	NA	NA	NA
Panc-AdenoCA	aa4a868a-df23-4eef-a618-e945aa2ce98a	5	36058599	36058657	6	58	NA	NA	5	1	NA	NA
Panc-AdenoCA	aa4a868a-df23-4eef-a618-e945aa2ce98a	8	13572152	13575800	7	3648	NA	NA	NA	4	1	2
Panc-AdenoCA	aa4a868a-df23-4eef-a618-e945aa2ce98a	9	28204202	28214187	13	9985	1	10	2	NA	NA	NA
Panc-AdenoCA	aa4a868a-df23-4eef-a618-e945aa2ce98a	13	60407537	60409806	6	2269	NA	NA	NA	3	2	1
Lymph-BNHL	aa847c3d-d3a7-46aa-a81b-db5099a458fb	8	128749217	128752907	27	3690	NA	6	11	6	1	3
Lymph-BNHL	aa847c3d-d3a7-46aa-a81b-db5099a458fb	8	128807530	128809016	8	1486	NA	NA	3	5	NA	NA
Lymph-BNHL	aa847c3d-d3a7-46aa-a81b-db5099a458fb	14	106237883	106250359	32	12476	2	9	13	3	2	3
Lymph-BNHL	aa847c3d-d3a7-46aa-a81b-db5099a458fb	14	106318497	106356712	158	38215	7	48	47	27	10	19
Lymph-BNHL	aa847c3d-d3a7-46aa-a81b-db5099a458fb	14	107048788	107049618	7	830	NA	2	NA	2	1	2

Lymph-BNHL	aa847c3d-d3a7-46aa-a81b-db5099a458fb	22	23040686	23041566	10	880	1	2	5	2	NA	NA	
Lymph-BNHL	aa847c3d-d3a7-46aa-a81b-db5099a458fb	22	23055169	23055622	12	453	1	3	5	3	NA	NA	
Lymph-BNHL	aa847c3d-d3a7-46aa-a81b-db5099a458fb	22	23100821	23115032	45	14211	6	8	14	10	2	5	
Lymph-BNHL	aa847c3d-d3a7-46aa-a81b-db5099a458fb	22	23235975	23247836	46	11861	4	12	15	11	2	2	
Liver-HCC	aabddb20-c622-11e3-bf01-24c6515278c0	12	68457777	68459682	6	1905	NA	NA	6	NA	NA	NA	
Breast-AdenoCa	aacaba97-ecde-440b-aae0-0e9c608c4b24	14	74292169	74295440	8	3271	1	1	5	1	NA	NA	
Breast-AdenoCa	aacaba97-ecde-440b-aae0-0e9c608c4b24	17	56831141	56831621	6	480	NA	NA	6	NA	NA	NA	
Prost-AdenoCA	ab8a55ed-ff47-4cad-ad91-52b9dc25aca7	1	106486375	106489406	11	3031	1	2	1	4	2	1	
Prost-AdenoCA	ab8a55ed-ff47-4cad-ad91-52b9dc25aca7	20	35286184	35290481	8	4297	NA	4	4	NA	NA	NA	
Prost-AdenoCA	ab8a55ed-ff47-4cad-ad91-52b9dc25aca7	X	83966025	83967824	8	1799	1	NA	4	2	NA	1	
Head-SCC	ab923db7-54e8-4a50-b7fd-c2b4b300041e	2	919790	923529	6	3739	1	1	4	NA	NA	NA	
Head-SCC	ab923db7-54e8-4a50-b7fd-c2b4b300041e	3	8766336	8767953	7	1617	NA	3	4	NA	NA	NA	
Head-SCC	ab923db7-54e8-4a50-b7fd-c2b4b300041e	7	46868269	46872152	9	3883	1	2	5	NA	NA	1	
Liver-HCC	abbbe65c-c622-11e3-bf01-24c6515278c0	4	36143554	36145719	7	2165	NA	1	NA	NA	6	NA	
Bladder-TCC	abd2d959-d5ed-4eb3-9759-67eb1aa23325	1	27207323	27212425	9	5102	1	6	2	NA	NA	NA	
Bladder-TCC	abd2d959-d5ed-4eb3-9759-67eb1aa23325	2	7476145	7479320	19	3175	NA	NA	19	NA	NA	NA	
Bladder-TCC	abd2d959-d5ed-4eb3-9759-67eb1aa23325	2	219420916	219423573	7	2657	NA	5	2	NA	NA	NA	
Bladder-TCC	abd2d959-d5ed-4eb3-9759-67eb1aa23325	2	238576742	238578740	7	1998	1	2	4	NA	NA	NA	
Bladder-TCC	abd2d959-d5ed-4eb3-9759-67eb1aa23325	3	14716772	14718119	8	1347	NA	NA	8	NA	NA	NA	
Bladder-TCC	abd2d959-d5ed-4eb3-9759-67eb1aa23325	3	47603386	47611750	24	8364	NA	5	19	NA	NA	NA	
Bladder-TCC	abd2d959-d5ed-4eb3-9759-67eb1aa23325	3	156945385	156948725	11	3340	2	6	3	NA	NA	NA	
Bladder-TCC	abd2d959-d5ed-4eb3-9759-67eb1aa23325	4	153284854	153288499	6	3645	1	3	2	NA	NA	NA	
Bladder-TCC	abd2d959-d5ed-4eb3-9759-67eb1aa23325	5	108147645	108152559	6	4914	1	1	4	NA	NA	NA	
Bladder-TCC	abd2d959-d5ed-4eb3-9759-67eb1aa23325	6	1304050	1310883	8	6833	NA	1	7	NA	NA	NA	
Bladder-TCC	abd2d959-d5ed-4eb3-9759-67eb1aa23325	6	107694944	107696104	10	1160	1	5	4	NA	NA	NA	
Bladder-TCC	abd2d959-d5ed-4eb3-9759-67eb1aa23325	6	107709990	107712434	20	2444	2	9	8	1	NA	NA	
Bladder-TCC	abd2d959-d5ed-4eb3-9759-67eb1aa23325	6	108895743	108906705	15	10962	1	4	9	1	NA	NA	
Bladder-TCC	abd2d959-d5ed-4eb3-9759-67eb1aa23325	8	110752357	110757379	11	5022	2	4	5	NA	NA	NA	
Bladder-TCC	abd2d959-d5ed-4eb3-9759-67eb1aa23325	8	123372735	123373965	17	1230	2	7	8	NA	NA	NA	
Bladder-TCC	abd2d959-d5ed-4eb3-9759-67eb1aa23325	8	143564091	143569453	10	5362	1	3	6	NA	NA	NA	
Bladder-TCC	abd2d959-d5ed-4eb3-9759-67eb1aa23325	9	123441696	123443072	6	1376	NA	3	3	NA	NA	NA	
Bladder-TCC	abd2d959-d5ed-4eb3-9759-67eb1aa23325	10	105507558	105509279	11	1721	NA	8	3	NA	NA	NA	
Bladder-TCC	abd2d959-d5ed-4eb3-9759-67eb1aa23325	11	12719978	12722113	7	2135	NA	5	2	NA	NA	NA	
Bladder-TCC	abd2d959-d5ed-4eb3-9759-67eb1aa23325	11	87878907	87880843	10	1936	1	3	6	NA	NA	NA	
Bladder-TCC	abd2d959-d5ed-4eb3-9759-67eb1aa23325	12	5412796	5413743	7	947	1	2	4	NA	NA	NA	
Bladder-TCC	abd2d959-d5ed-4eb3-9759-67eb1aa23325	12	47637961	47639970	7	2009	1	4	2	NA	NA	NA	
Bladder-TCC	abd2d959-d5ed-4eb3-9759-67eb1aa23325	16	30723437	30726984	7	3547	NA	5	2	NA	NA	NA	
Bladder-TCC	abd2d959-d5ed-4eb3-9759-67eb1aa23325	19	13265860	13267745	6	1885	2	1	3	NA	NA	NA	
Bladder-TCC	abd2d959-d5ed-4eb3-9759-67eb1aa23325	19	42759517	42762209	9	2692	1	3	5	NA	NA	NA	
Bladder-TCC	abd2d959-d5ed-4eb3-9759-67eb1aa23325	21	45760929	45763035	6	2106	NA	4	2	NA	NA	NA	
Bladder-TCC	abd2d959-d5ed-4eb3-9759-67eb1aa23325	22	39853982	39861472	14	7490	NA	4	8	NA	1	1	
Lymph-BNHL	abedd46c-47b8-4242-adb6-12835400cb3f	1	11081297	11086147	6	4850	NA	NA	1	2	1	2	
Lymph-BNHL	abedd46c-47b8-4242-adb6-12835400cb3f	1	111250237	111253194	7	2957	NA	NA	1	3	NA	3	
Lymph-BNHL	abedd46c-47b8-4242-adb6-12835400cb3f	2	17582772	17585521	6	2749	1	NA	NA	1	1	3	
Lymph-BNHL	abedd46c-47b8-4242-adb6-12835400cb3f	2	89127478	89292176	274	2E+05	13	25	26	89	57	64	
Lymph-BNHL	abedd46c-47b8-4242-adb6-12835400cb3f	2	126106541	126119357	15	12816	NA	NA	1	7	5	2	
Lymph-BNHL	abedd46c-47b8-4242-adb6-12835400cb3f	2	136875077	136875507	8	430	1	1	NA	NA	2	4	
Lymph-BNHL	abedd46c-47b8-4242-adb6-12835400cb3f	2	146878398	146878677	7	279	NA	NA	NA	4	3	NA	
Lymph-BNHL	abedd46c-47b8-4242-adb6-12835400cb3f	3	16494858	16495899	6	1041	NA	1	NA	2	1	2	
Lymph-BNHL	abedd46c-47b8-4242-adb6-12835400cb3f	3	110336166	110336961	7	795	NA	NA	NA	5	1	1	
Lymph-BNHL	abedd46c-47b8-4242-adb6-12835400cb3f	3	166293088	166302561	15	9473	1	1	NA	6	3	4	
Lymph-BNHL	abedd46c-47b8-4242-adb6-12835400cb3f	3	187460864	187481654	60	20790	4	13	7	21	8	7	
Lymph-BNHL	abedd46c-47b8-4242-adb6-12835400cb3f	3	187657482	187664650	11	7168	NA	2	NA	5	2	2	
Lymph-BNHL	abedd46c-47b8-4242-adb6-12835400cb3f	3	187957842	187960053	22	2211	NA	1	1	12	3	5	
Lymph-BNHL	abedd46c-47b8-4242-adb6-12835400cb3f	3	188465846	188472265	20	6419	1	1	1	13	3	1	
Lymph-BNHL	abedd46c-47b8-4242-adb6-12835400cb3f	4	40199118	40202119	17	3001	1	1	2	5	5	3	
Lymph-BNHL	abedd46c-47b8-4242-adb6-12835400cb3f	4	63934404	63944346	15	9942	NA	1	NA	7	3	4	
Lymph-BNHL	abedd46c-47b8-4242-adb6-12835400cb3f	5	162518136	162522000	6	3864	NA	NA	NA	2	2	2	
Lymph-BNHL	abedd46c-47b8-4242-adb6-12835400cb3f	6	19570260	19574867	7	4607	NA	1	NA	5	NA	1	
Lymph-BNHL	abedd46c-47b8-4242-adb6-12835400cb3f	6	31549590	31550010	15	420	NA	5	5	2	3	NA	

Lymph-BNHL	abedd46c-47b8-4242-adb6-12835400cb3f	6	91004993	91007444	13	2451	NA	4	2	4	2	1
Lymph-BNHL	abedd46c-47b8-4242-adb6-12835400cb3f	6	134491760	134557456	125	65696	5	27	9	32	25	27
Lymph-BNHL	abedd46c-47b8-4242-adb6-12835400cb3f	7	27870895	27873652	6	2757	NA	NA	NA	4	NA	2
Lymph-BNHL	abedd46c-47b8-4242-adb6-12835400cb3f	9	1922073	1926817	7	4744	1	NA	1	2	1	2
Lymph-BNHL	abedd46c-47b8-4242-adb6-12835400cb3f	9	37406657	37407433	8	776	NA	1	3	2	2	NA
Lymph-BNHL	abedd46c-47b8-4242-adb6-12835400cb3f	10	67838251	67850886	16	12635	NA	3	3	NA	3	7
Lymph-BNHL	abedd46c-47b8-4242-adb6-12835400cb3f	10	67865141	67872128	11	6987	2	1	1	1	5	1
Lymph-BNHL	abedd46c-47b8-4242-adb6-12835400cb3f	10	67876352	67881978	7	5626	NA	NA	NA	4	1	2
Lymph-BNHL	abedd46c-47b8-4242-adb6-12835400cb3f	10	67900422	67904793	7	4371	2	NA	NA	3	1	1
Lymph-BNHL	abedd46c-47b8-4242-adb6-12835400cb3f	12	69030164	69032629	7	2465	1	NA	NA	4	2	NA
Lymph-BNHL	abedd46c-47b8-4242-adb6-12835400cb3f	12	99085196	99085379	8	183	NA	NA	NA	3	3	2
Lymph-BNHL	abedd46c-47b8-4242-adb6-12835400cb3f	12	122459110	122466702	19	7592	NA	3	5	9	1	1
Lymph-BNHL	abedd46c-47b8-4242-adb6-12835400cb3f	13	41155848	41156364	8	516	NA	NA	1	5	1	1
Lymph-BNHL	abedd46c-47b8-4242-adb6-12835400cb3f	13	48542521	48542619	7	98	NA	NA	NA	3	2	2
Lymph-BNHL	abedd46c-47b8-4242-adb6-12835400cb3f	14	69259033	69262125	7	3092	1	1	2	NA	3	NA
Lymph-BNHL	abedd46c-47b8-4242-adb6-12835400cb3f	14	83712091	83714313	8	2222	NA	NA	NA	3	2	3
Lymph-BNHL	abedd46c-47b8-4242-adb6-12835400cb3f	14	106051870	106072076	25	20206	NA	4	12	1	5	3
Lymph-BNHL	abedd46c-47b8-4242-adb6-12835400cb3f	14	106211015	106213328	8	2313	NA	1	7	NA	NA	NA
Lymph-BNHL	abedd46c-47b8-4242-adb6-12835400cb3f	14	106235800	106238667	8	2867	1	1	2	1	1	2
Lymph-BNHL	abedd46c-47b8-4242-adb6-12835400cb3f	14	106327144	106390817	85	63673	5	16	19	22	14	9
Lymph-BNHL	abedd46c-47b8-4242-adb6-12835400cb3f	14	106816305	106817783	12	1478	1	NA	3	4	3	1
Lymph-BNHL	abedd46c-47b8-4242-adb6-12835400cb3f	15	86233751	86235781	6	2030	NA	NA	NA	4	NA	2
Lymph-BNHL	abedd46c-47b8-4242-adb6-12835400cb3f	16	30321517	30321823	6	306	NA	NA	NA	2	2	2
Lymph-BNHL	abedd46c-47b8-4242-adb6-12835400cb3f	17	56409049	56409673	6	624	NA	2	4	NA	NA	NA
Lymph-BNHL	abedd46c-47b8-4242-adb6-12835400cb3f	20	37435442	37437190	6	1748	1	1	4	NA	NA	NA
Lymph-BNHL	abedd46c-47b8-4242-adb6-12835400cb3f	21	21635903	21636419	7	516	NA	NA	1	4	1	1
Lymph-BNHL	abedd46c-47b8-4242-adb6-12835400cb3f	22	23199038	23199373	8	335	2	NA	1	2	2	1
Lymph-BNHL	abedd46c-47b8-4242-adb6-12835400cb3f	22	23222936	23284845	147	61909	12	27	27	31	28	22
Lymph-BNHL	abedd46c-47b8-4242-adb6-12835400cb3f	X	12992939	12994393	12	1454	NA	5	3	2	1	1
Lymph-BNHL	abedd46c-47b8-4242-adb6-12835400cb3f	X	42802152	42802689	11	537	NA	2	3	2	3	1
Lymph-BNHL	abedd46c-47b8-4242-adb6-12835400cb3f	X	138167598	138168785	13	1187	NA	1	2	4	1	5
Panc-AdenoCA	ac02c38e-5fca-4995-b0cc-39b6189976a3	6	66543925	66559860	26	15935	3	7	16	NA	NA	NA
Panc-AdenoCA	ac02c38e-5fca-4995-b0cc-39b6189976a3	7	76221381	76228415	13	7034	3	5	5	NA	NA	NA
Panc-AdenoCA	ac02c38e-5fca-4995-b0cc-39b6189976a3	7	151911292	151913639	12	2347	4	3	4	NA	NA	1
Panc-AdenoCA	ac02c38e-5fca-4995-b0cc-39b6189976a3	8	5379660	5383950	40	4290	6	13	21	NA	NA	NA
Panc-AdenoCA	ac02c38e-5fca-4995-b0cc-39b6189976a3	12	4548017	4551867	13	3850	3	2	8	NA	NA	NA
Panc-AdenoCA	ac02c38e-5fca-4995-b0cc-39b6189976a3	18	51044499	51045418	6	919	NA	2	4	NA	NA	NA
Panc-AdenoCA	ac02c38e-5fca-4995-b0cc-39b6189976a3	21	41180449	41187730	15	7281	1	6	8	NA	NA	NA
Lymph-BNHL	ac2c8928-33a2-4aa0-8bc1-cdfa1ca0b56a	2	89159737	89165523	44	5786	NA	5	7	16	5	11
Lymph-BNHL	ac2c8928-33a2-4aa0-8bc1-cdfa1ca0b56a	3	60602988	60608867	8	5879	3	NA	1	4	NA	NA
Lymph-BNHL	ac2c8928-33a2-4aa0-8bc1-cdfa1ca0b56a	14	106325473	106328981	12	3508	4	1	1	3	NA	3
Lymph-BNHL	ac2c8928-33a2-4aa0-8bc1-cdfa1ca0b56a	14	106370719	106374303	8	3584	NA	1	1	2	1	3
Lymph-BNHL	ac2c8928-33a2-4aa0-8bc1-cdfa1ca0b56a	14	106830023	106831496	11	1473	1	NA	2	4	3	1
Lymph-BNHL	ac2c8928-33a2-4aa0-8bc1-cdfa1ca0b56a	22	23223402	23235278	39	11876	1	6	13	15	2	2
Bladder-TCC	acc629cb-ad03-4cec-9b21-922e4932ef3e	1	1611896	1639076	29	27180	9	6	13	1	NA	NA
Bladder-TCC	acc629cb-ad03-4cec-9b21-922e4932ef3e	1	202770864	202777707	21	6843	2	9	10	NA	NA	NA
Bladder-TCC	acc629cb-ad03-4cec-9b21-922e4932ef3e	2	161022891	161025127	6	2236	1	4	1	NA	NA	NA
Bladder-TCC	acc629cb-ad03-4cec-9b21-922e4932ef3e	4	128221887	128224267	8	2380	1	1	6	NA	NA	NA
Bladder-TCC	acc629cb-ad03-4cec-9b21-922e4932ef3e	4	154119552	154120370	7	818	1	3	3	NA	NA	NA
Bladder-TCC	acc629cb-ad03-4cec-9b21-922e4932ef3e	5	176933076	176934269	6	1193	NA	2	4	NA	NA	NA
Bladder-TCC	acc629cb-ad03-4cec-9b21-922e4932ef3e	6	40232939	40235659	9	2720	NA	5	4	NA	NA	NA
Bladder-TCC	acc629cb-ad03-4cec-9b21-922e4932ef3e	6	53398610	53403822	9	5212	NA	6	3	NA	NA	NA
Bladder-TCC	acc629cb-ad03-4cec-9b21-922e4932ef3e	6	74957793	74958262	7	469	1	3	3	NA	NA	NA
Bladder-TCC	acc629cb-ad03-4cec-9b21-922e4932ef3e	7	47778006	47780585	18	2579	NA	3	15	NA	NA	NA
Bladder-TCC	acc629cb-ad03-4cec-9b21-922e4932ef3e	7	120159380	120160593	7	1213	NA	5	2	NA	NA	NA
Bladder-TCC	acc629cb-ad03-4cec-9b21-922e4932ef3e	7	123493716	123496613	12	2897	3	4	5	NA	NA	NA
Bladder-TCC	acc629cb-ad03-4cec-9b21-922e4932ef3e	7	128542280	128544957	8	2677	2	3	3	NA	NA	NA
Bladder-TCC	acc629cb-ad03-4cec-9b21-922e4932ef3e	8	92983201	92984996	7	1795	2	4	1	NA	NA	NA
Bladder-TCC	acc629cb-ad03-4cec-9b21-922e4932ef3e	9	4076994	4080515	6	3521	NA	NA	6	NA	NA	NA
Bladder-TCC	acc629cb-ad03-4cec-9b21-922e4932ef3e	9	15842385	15844511	9	2126	NA	NA	9	NA	NA	NA

Bladder-TCC	acc629cb-ad03-4cec-9b21-922e4932ef3e	9	15857279	15859522	7	2243	1	2	4	NA	NA	NA
Bladder-TCC	acc629cb-ad03-4cec-9b21-922e4932ef3e	11	66518750	66519575	6	825	NA	4	2	NA	NA	NA
Bladder-TCC	acc629cb-ad03-4cec-9b21-922e4932ef3e	12	47978327	47979844	6	1517	1	4	1	NA	NA	NA
Bladder-TCC	acc629cb-ad03-4cec-9b21-922e4932ef3e	14	35477819	35481094	6	3275	NA	3	3	NA	NA	NA
Bladder-TCC	acc629cb-ad03-4cec-9b21-922e4932ef3e	14	64789379	64793108	8	3729	NA	6	2	NA	NA	NA
Bladder-TCC	acc629cb-ad03-4cec-9b21-922e4932ef3e	15	59137257	59140730	9	3473	NA	6	3	NA	NA	NA
Bladder-TCC	acc629cb-ad03-4cec-9b21-922e4932ef3e	16	3024681	3027773	11	3092	1	4	5	NA	NA	1
Bladder-TCC	acc629cb-ad03-4cec-9b21-922e4932ef3e	16	58262763	58267958	19	5195	1	10	8	NA	NA	NA
Bladder-TCC	acc629cb-ad03-4cec-9b21-922e4932ef3e	17	37929975	37932335	7	2360	NA	4	3	NA	NA	NA
Bladder-TCC	acc629cb-ad03-4cec-9b21-922e4932ef3e	17	60586037	60590028	7	3991	NA	3	4	NA	NA	NA
Bladder-TCC	acc629cb-ad03-4cec-9b21-922e4932ef3e	17	60595691	60599440	11	3749	3	5	3	NA	NA	NA
Bladder-TCC	acc629cb-ad03-4cec-9b21-922e4932ef3e	19	21984145	21985520	9	1375	2	3	4	NA	NA	NA
Bladder-TCC	acc629cb-ad03-4cec-9b21-922e4932ef3e	20	47706040	47709677	7	3637	1	NA	5	NA	1	NA
Bladder-TCC	acc629cb-ad03-4cec-9b21-922e4932ef3e	X	96111869	96113474	6	1605	NA	4	2	NA	NA	NA
Ovary-AdenoCA	acd510de-b732-4a1a-8b72-6d22e763540b	7	118961636	118962829	10	1193	4	6	NA	NA	NA	NA
Lung-SCC	ad41f1f3-e38e-4418-9c16-52bca5719f39	2	199229909	199235091	9	5182	1	3	5	NA	NA	NA
Lung-SCC	ad41f1f3-e38e-4418-9c16-52bca5719f39	2	214139848	214146290	8	6442	NA	7	1	NA	NA	NA
Lung-SCC	ad41f1f3-e38e-4418-9c16-52bca5719f39	2	227715908	227721767	8	5859	1	6	1	NA	NA	NA
Lung-SCC	ad41f1f3-e38e-4418-9c16-52bca5719f39	5	178840522	178841904	16	1382	1	7	8	NA	NA	NA
Lung-SCC	ad41f1f3-e38e-4418-9c16-52bca5719f39	8	35972202	35976247	9	4045	1	6	2	NA	NA	NA
Eso-AdenoCa	ad4aa4dc-bea4-4f4a-8b1b-8f361112f84f	7	124883040	124883686	7	646	1	2	4	NA	NA	NA
Eso-AdenoCa	ad4aa4dc-bea4-4f4a-8b1b-8f361112f84f	7	127581181	127582863	8	1682	NA	2	6	NA	NA	NA
Lymph-CLL	ad92f17f-11e6-49bd-9835-6b2f47053485	2	89159407	89165368	18	5961	NA	5	NA	6	3	4
Lymph-CLL	ad92f17f-11e6-49bd-9835-6b2f47053485	14	106326619	106329454	27	2835	1	1	8	9	3	5
Kidney-RCC	ad9455e9-7147-489e-9b1f-3540c457c260	16	80973714	80973721	6	7	NA	NA	3	1	NA	2
Liver-HCC	adfbba82-c622-11e3-bf01-24c6515278c0	14	56677852	56680459	8	2607	2	4	1	1	NA	NA
Liver-HCC	adfbba82-c622-11e3-bf01-24c6515278c0	17	16003025	16004446	6	1421	NA	4	2	NA	NA	NA
Ovary-AdenoCA	ae82fead-2671-4335-a342-67bb69935ad9	2	236585390	236588311	19	2921	2	9	7	1	NA	NA
Ovary-AdenoCA	ae82fead-2671-4335-a342-67bb69935ad9	2	239344041	239345263	7	1222	3	4	NA	NA	NA	NA
Ovary-AdenoCA	ae82fead-2671-4335-a342-67bb69935ad9	8	129650599	129667818	25	17219	NA	16	9	NA	NA	NA
Ovary-AdenoCA	ae82fead-2671-4335-a342-67bb69935ad9	11	86593064	86594124	12	1060	3	4	5	NA	NA	NA
Ovary-AdenoCA	ae82fead-2671-4335-a342-67bb69935ad9	13	92246911	92251366	13	4455	3	5	5	NA	NA	NA
Ovary-AdenoCA	ae82fead-2671-4335-a342-67bb69935ad9	17	30987004	30987918	7	914	2	4	1	NA	NA	NA
Liver-HCC	af2cbdb6-c622-11e3-bf01-24c6515278c0	1	243034576	243039521	8	4945	NA	3	2	1	2	NA
Panc-AdenoCA	af9cfac8-7fbe-49cb-aeca-8d68aea57994	1	113937836	113943591	11	5755	NA	6	5	NA	NA	NA
Panc-AdenoCA	af9cfac8-7fbe-49cb-aeca-8d68aea57994	14	32442485	32444186	6	1701	1	NA	5	NA	NA	NA
Panc-AdenoCA	af9cfac8-7fbe-49cb-aeca-8d68aea57994	17	59987467	59988147	8	680	NA	5	3	NA	NA	NA
Panc-AdenoCA	af9cfac8-7fbe-49cb-aeca-8d68aea57994	19	28068666	28069573	6	907	NA	3	3	NA	NA	NA
Panc-AdenoCA	af9cfac8-7fbe-49cb-aeca-8d68aea57994	19	28123934	28128376	19	4442	1	5	13	NA	NA	NA
Panc-AdenoCA	af9cfac8-7fbe-49cb-aeca-8d68aea57994	19	29968906	29978247	13	9341	2	4	7	NA	NA	NA
Panc-AdenoCA	af9cfac8-7fbe-49cb-aeca-8d68aea57994	19	30033876	30034595	17	719	6	3	8	NA	NA	NA
Panc-AdenoCA	af9cfac8-7fbe-49cb-aeca-8d68aea57994	19	30695591	30696773	16	1182	6	7	2	1	NA	NA
Panc-AdenoCA	af9cfac8-7fbe-49cb-aeca-8d68aea57994	19	31758564	31764335	8	5771	3	1	1	1	NA	2
Panc-AdenoCA	af9cfac8-7fbe-49cb-aeca-8d68aea57994	19	33109863	33119848	16	9985	6	1	8	NA	NA	1
Bone-Leiomyo	afee5b10-3dff-4e50-9575-bc9fe20c5dea	2	26698171	26698649	7	478	1	NA	6	NA	NA	NA
Bone-Leiomyo	afee5b10-3dff-4e50-9575-bc9fe20c5dea	2	26724562	26726293	8	1731	NA	NA	8	NA	NA	NA
Bone-Leiomyo	afee5b10-3dff-4e50-9575-bc9fe20c5dea	2	41245061	41246938	8	1877	NA	6	2	NA	NA	NA
Bone-Leiomyo	afee5b10-3dff-4e50-9575-bc9fe20c5dea	2	60743436	60747890	9	4454	1	2	6	NA	NA	NA
Bone-Leiomyo	afee5b10-3dff-4e50-9575-bc9fe20c5dea	2	180863924	180873362	11	9438	1	6	4	NA	NA	NA
Bone-Leiomyo	afee5b10-3dff-4e50-9575-bc9fe20c5dea	4	55760548	55765327	7	4779	NA	NA	7	NA	NA	NA
Bone-Leiomyo	afee5b10-3dff-4e50-9575-bc9fe20c5dea	6	103914468	103919444	6	4976	1	NA	1	2	NA	2
Bone-Leiomyo	afee5b10-3dff-4e50-9575-bc9fe20c5dea	12	4751350	4752528	14	1178	2	3	9	NA	NA	NA
Bone-Leiomyo	afee5b10-3dff-4e50-9575-bc9fe20c5dea	12	107146101	107156387	13	10286	1	NA	12	NA	NA	NA
Bone-Leiomyo	afee5b10-3dff-4e50-9575-bc9fe20c5dea	12	118249829	118254940	9	5111	1	2	5	1	NA	NA
Bone-Leiomyo	afee5b10-3dff-4e50-9575-bc9fe20c5dea	12	121510417	121512030	9	1613	1	3	5	NA	NA	NA
Bone-Leiomyo	afee5b10-3dff-4e50-9575-bc9fe20c5dea	12	121632875	121633391	8	516	NA	NA	5	1	NA	2
Bone-Leiomyo	afee5b10-3dff-4e50-9575-bc9fe20c5dea	12	129438950	129441544	7	2594	1	1	5	NA	NA	NA
Bone-Leiomyo	afee5b10-3dff-4e50-9575-bc9fe20c5dea	12	131906156	131915567	15	9411	5	2	8	NA	NA	NA
Bone-Leiomyo	afee5b10-3dff-4e50-9575-bc9fe20c5dea	14	25998263	26002683	13	4420	2	5	6	NA	NA	NA
Bone-Leiomyo	afee5b10-3dff-4e50-9575-bc9fe20c5dea	15	91954296	91955063	8	767	NA	1	7	NA	NA	NA

Skin-Melanoma	affa4ef4-02ec-40f1-b6a2-739f8bf2afa9	8	34836999	34838322	11	1323	2	7	2	NA	NA	NA
Skin-Melanoma	affa4ef4-02ec-40f1-b6a2-739f8bf2afa9	8	138208867	138211027	12	2160	NA	7	4	NA	1	NA
Skin-Melanoma	affa4ef4-02ec-40f1-b6a2-739f8bf2afa9	8	141016919	141018079	10	1160	1	6	3	NA	NA	NA
Eso-AdenoCa	b02b4bba-6e66-44fb-a48f-38c309aaaac5	2	23366685	23367899	6	1214	NA	1	5	NA	NA	NA
Eso-AdenoCa	b02b4bba-6e66-44fb-a48f-38c309aaaac5	2	157002233	157004707	7	2474	1	3	3	NA	NA	NA
Eso-AdenoCa	b02b4bba-6e66-44fb-a48f-38c309aaaac5	2	186106999	186111741	7	4742	2	1	3	NA	1	NA
Eso-AdenoCa	b02b4bba-6e66-44fb-a48f-38c309aaaac5	2	205951183	205955608	7	4425	1	4	2	NA	NA	NA
Eso-AdenoCa	b02b4bba-6e66-44fb-a48f-38c309aaaac5	5	32856957	32860205	7	3248	NA	5	2	NA	NA	NA
Eso-AdenoCa	b02b4bba-6e66-44fb-a48f-38c309aaaac5	18	24956115	24960311	17	4196	NA	NA	16	1	NA	NA
Liver-HCC	b070af2a-c622-11e3-bf01-24c6515278c0	4	46136817	46138368	36	1551	2	14	19	1	NA	NA
Liver-HCC	b070af2a-c622-11e3-bf01-24c6515278c0	4	58842633	58843631	14	998	NA	4	10	NA	NA	NA
Liver-HCC	b070af2a-c622-11e3-bf01-24c6515278c0	5	64503620	64506440	10	2820	1	4	5	NA	NA	NA
Liver-HCC	b070af2a-c622-11e3-bf01-24c6515278c0	5	95076741	95078175	18	1434	2	11	5	NA	NA	NA
Liver-HCC	b070af2a-c622-11e3-bf01-24c6515278c0	8	127506667	127506825	6	158	1	4	1	NA	NA	NA
Liver-HCC	b070af2a-c622-11e3-bf01-24c6515278c0	10	53554687	53563393	12	8706	1	3	8	NA	NA	NA
Liver-HCC	b070af2a-c622-11e3-bf01-24c6515278c0	11	65101927	65103505	7	1578	1	3	3	NA	NA	NA
Liver-HCC	b070af2a-c622-11e3-bf01-24c6515278c0	15	63642548	63642929	11	381	NA	4	7	NA	NA	NA
Liver-HCC	b070af2a-c622-11e3-bf01-24c6515278c0	15	74824636	74828507	14	3871	1	4	9	NA	NA	NA
Liver-HCC	b070af2a-c622-11e3-bf01-24c6515278c0	X	63794799	63796137	6	1338	NA	NA	NA	1	5	NA
Liver-HCC	b070af2a-c622-11e3-bf01-24c6515278c0	X	71366767	71373574	8	6807	NA	1	1	3	3	NA
Lymph-BNHL	b2190e26-1809-4aba-9f45-07c2edb3b4a4	2	89158896	89160400	22	1504	1	3	2	8	5	3
Lymph-BNHL	b2190e26-1809-4aba-9f45-07c2edb3b4a4	4	137504019	137507007	6	2988	NA	NA	2	2	NA	2
Lymph-BNHL	b2190e26-1809-4aba-9f45-07c2edb3b4a4	5	24131230	24136205	6	4975	NA	NA	1	1	NA	4
Lymph-BNHL	b2190e26-1809-4aba-9f45-07c2edb3b4a4	6	71895633	71898635	7	3002	NA	NA	1	3	1	2
Lymph-BNHL	b2190e26-1809-4aba-9f45-07c2edb3b4a4	8	15944820	15953463	11	8643	NA	NA	2	4	4	1
Lymph-BNHL	b2190e26-1809-4aba-9f45-07c2edb3b4a4	8	128748945	128752791	41	3846	5	11	15	4	1	5
Lymph-BNHL	b2190e26-1809-4aba-9f45-07c2edb3b4a4	14	48109792	48113500	7	3708	1	1	1	1	2	1
Lymph-BNHL	b2190e26-1809-4aba-9f45-07c2edb3b4a4	14	106325025	106330380	60	5355	3	13	11	9	10	14
Panc-Endocrine	b27b569a-2c79-4453-8a2d-41fd0b4dd28d	8	16537228	16537468	7	240	NA	NA	7	NA	NA	NA
Panc-Endocrine	b27b569a-2c79-4453-8a2d-41fd0b4dd28d	8	110047239	110048108	8	869	NA	NA	8	NA	NA	NA
Panc-Endocrine	b27b569a-2c79-4453-8a2d-41fd0b4dd28d	8	110066664	110071408	27	4744	NA	NA	27	NA	NA	NA
Panc-Endocrine	b27b569a-2c79-4453-8a2d-41fd0b4dd28d	8	111699021	111700080	14	1059	NA	4	10	NA	NA	NA
Panc-Endocrine	b27b569a-2c79-4453-8a2d-41fd0b4dd28d	8	112146997	112156723	28	9726	1	11	13	NA	1	2
Panc-Endocrine	b27b569a-2c79-4453-8a2d-41fd0b4dd28d	8	118123598	118138770	18	15172	NA	NA	18	NA	NA	NA
Panc-Endocrine	b27b569a-2c79-4453-8a2d-41fd0b4dd28d	8	128597929	128600871	41	2942	NA	7	34	NA	NA	NA
Breast-AdenoCa	b27d75ba-5989-4200-bfe9-f1b7d7cf8008	6	132134922	132139876	13	4954	3	3	6	NA	NA	1
Breast-AdenoCa	b27d75ba-5989-4200-bfe9-f1b7d7cf8008	8	128381194	128386381	7	5187	NA	NA	7	NA	NA	NA
CNS-GBM	b2d17671-d2e1-4c97-8b01-a976d5abe1d6	2	350936	355688	6	4752	1	1	2	1	1	NA
CNS-GBM	b2d17671-d2e1-4c97-8b01-a976d5abe1d6	13	92988926	92990115	7	1189	NA	4	3	NA	NA	NA
Kidney-RCC	b30dfb8b-8288-4e5a-afc2-3d5bd7bfa26c	4	162519872	162523881	6	4009	1	1	1	2	NA	1
Liver-HCC	b32449c0-c622-11e3-bf01-24c6515278c0	X	46555018	46556089	6	1071	2	NA	3	1	NA	NA
Prost-AdenoCA	b33978c6-a855-4f9d-a0b0-d79453b9de41	1	74431927	74433565	7	1638	1	NA	1	3	2	NA
Prost-AdenoCA	b33978c6-a855-4f9d-a0b0-d79453b9de41	1	170073426	170076840	7	3414	NA	NA	3	2	1	1
Prost-AdenoCA	b33978c6-a855-4f9d-a0b0-d79453b9de41	1	170729442	170732170	7	2728	NA	3	NA	3	NA	1
Prost-AdenoCA	b33978c6-a855-4f9d-a0b0-d79453b9de41	1	191026759	191032671	9	5912	2	2	3	1	NA	1
Prost-AdenoCA	b33978c6-a855-4f9d-a0b0-d79453b9de41	1	195637953	195641002	7	3049	1	NA	2	NA	1	3
Prost-AdenoCA	b33978c6-a855-4f9d-a0b0-d79453b9de41	2	18344086	18346529	7	2443	NA	NA	2	2	2	1
Prost-AdenoCA	b33978c6-a855-4f9d-a0b0-d79453b9de41	2	147402870	147405108	7	2238	NA	2	1	4	NA	NA
Prost-AdenoCA	b33978c6-a855-4f9d-a0b0-d79453b9de41	2	157926243	157928519	8	2276	NA	NA	1	3	1	3
Prost-AdenoCA	b33978c6-a855-4f9d-a0b0-d79453b9de41	3	39780707	39783325	7	2618	1	NA	2	2	NA	2
Prost-AdenoCA	b33978c6-a855-4f9d-a0b0-d79453b9de41	3	89125576	89126968	7	1392	1	1	2	1	2	NA
Prost-AdenoCA	b33978c6-a855-4f9d-a0b0-d79453b9de41	3	105027215	105030518	8	3303	1	NA	1	1	4	1
Prost-AdenoCA	b33978c6-a855-4f9d-a0b0-d79453b9de41	4	18497855	18501177	8	3322	NA	1	4	1	2	NA
Prost-AdenoCA	b33978c6-a855-4f9d-a0b0-d79453b9de41	4	68134600	68137448	7	2848	NA	NA	2	4	NA	1
Prost-AdenoCA	b33978c6-a855-4f9d-a0b0-d79453b9de41	4	83074948	83078982	8	4034	1	1	3	NA	2	1
Prost-AdenoCA	b33978c6-a855-4f9d-a0b0-d79453b9de41	4	104767127	104772594	7	5467	4	NA	2	1	NA	NA
Prost-AdenoCA	b33978c6-a855-4f9d-a0b0-d79453b9de41	5	122665708	122668177	6	2469	NA	1	1	3	NA	1
Prost-AdenoCA	b33978c6-a855-4f9d-a0b0-d79453b9de41	5	143875334	143878868	8	3534	1	1	1	2	NA	3
Prost-AdenoCA	b33978c6-a855-4f9d-a0b0-d79453b9de41	6	66576395	66577357	6	962	NA	NA	3	3	NA	NA
Prost-AdenoCA	b33978c6-a855-4f9d-a0b0-d79453b9de41	6	124560938	124565266	8	4328	1	NA	2	3	1	1

Prost-AdenoCA	b33978c6-a855-4f9d-a0b0-d79453b9de41	6	126930284	126932794	9	2510	1	1	2	3	1	1
Prost-AdenoCA	b33978c6-a855-4f9d-a0b0-d79453b9de41	7	34499319	34502408	7	3089	NA	1	3	1	1	1
Prost-AdenoCA	b33978c6-a855-4f9d-a0b0-d79453b9de41	7	93117566	93120747	9	3181	NA	NA	2	7	NA	NA
Prost-AdenoCA	b33978c6-a855-4f9d-a0b0-d79453b9de41	8	49266137	49269881	7	3744	1	NA	NA	4	1	1
Prost-AdenoCA	b33978c6-a855-4f9d-a0b0-d79453b9de41	8	100602636	100605277	7	2641	1	1	NA	3	1	1
Prost-AdenoCA	b33978c6-a855-4f9d-a0b0-d79453b9de41	8	128056825	128058214	12	1389	NA	NA	1	8	1	2
Prost-AdenoCA	b33978c6-a855-4f9d-a0b0-d79453b9de41	9	12865371	12868085	6	2714	NA	NA	2	2	2	NA
Prost-AdenoCA	b33978c6-a855-4f9d-a0b0-d79453b9de41	10	59477740	59480830	7	3090	NA	1	2	2	NA	2
Prost-AdenoCA	b33978c6-a855-4f9d-a0b0-d79453b9de41	11	29686757	29689799	6	3042	NA	NA	1	3	NA	2
Prost-AdenoCA	b33978c6-a855-4f9d-a0b0-d79453b9de41	11	50427681	50434083	8	6402	NA	1	2	3	1	1
Prost-AdenoCA	b33978c6-a855-4f9d-a0b0-d79453b9de41	12	45178656	45179832	6	1176	NA	1	1	3	1	NA
Prost-AdenoCA	b33978c6-a855-4f9d-a0b0-d79453b9de41	12	88833396	88835936	7	2540	NA	1	2	2	1	1
Prost-AdenoCA	b33978c6-a855-4f9d-a0b0-d79453b9de41	13	57341784	57343002	6	1218	NA	2	2	2	NA	NA
Prost-AdenoCA	b33978c6-a855-4f9d-a0b0-d79453b9de41	13	92486185	92487194	7	1009	1	NA	1	3	NA	2
Prost-AdenoCA	b33978c6-a855-4f9d-a0b0-d79453b9de41	13	104835756	104837392	7	1636	NA	1	NA	4	1	1
Prost-AdenoCA	b33978c6-a855-4f9d-a0b0-d79453b9de41	14	60132913	60135718	7	2805	1	1	2	NA	1	2
Prost-AdenoCA	b33978c6-a855-4f9d-a0b0-d79453b9de41	14	86368856	86370853	7	1997	NA	NA	3	1	2	1
Prost-AdenoCA	b33978c6-a855-4f9d-a0b0-d79453b9de41	18	30551209	30552528	6	1319	1	NA	2	1	NA	2
Prost-AdenoCA	b33978c6-a855-4f9d-a0b0-d79453b9de41	20	10800601	10802623	7	2022	NA	1	1	4	NA	1
Prost-AdenoCA	b33978c6-a855-4f9d-a0b0-d79453b9de41	21	18527833	18529713	6	1880	NA	NA	1	4	1	NA
Prost-AdenoCA	b33978c6-a855-4f9d-a0b0-d79453b9de41	X	21233703	21238304	6	4601	1	NA	NA	3	1	1
Prost-AdenoCA	b33978c6-a855-4f9d-a0b0-d79453b9de41	X	66195647	66197804	7	2157	1	2	1	2	1	NA
Prost-AdenoCA	b33978c6-a855-4f9d-a0b0-d79453b9de41	Y	21955355	21958277	7	2922	NA	NA	1	4	2	NA
Prost-AdenoCA	b33978c6-a855-4f9d-a0b0-d79453b9de41	Y	23047962	23051209	8	3247	1	1	2	2	1	1
Prost-AdenoCA	b33b7c8f-0b0d-4009-88a7-48e9d9cae6cb	X	66193142	66197372	7	4230	NA	3	1	1	2	NA
Skin-Melanoma	b35d9a68-29f4-49ab-b83e-b5151679e3af	1	77978536	77978893	20	357	NA	NA	20	NA	NA	NA
Skin-Melanoma	b35d9a68-29f4-49ab-b83e-b5151679e3af	3	155050285	155051352	6	1067	2	1	3	NA	NA	NA
Skin-Melanoma	b35d9a68-29f4-49ab-b83e-b5151679e3af	4	44243313	44245915	25	2602	2	6	17	NA	NA	NA
Skin-Melanoma	b35d9a68-29f4-49ab-b83e-b5151679e3af	4	45108797	45113286	11	4489	NA	4	7	NA	NA	NA
Skin-Melanoma	b35d9a68-29f4-49ab-b83e-b5151679e3af	5	925093	927398	6	2305	1	2	3	NA	NA	NA
Skin-Melanoma	b35d9a68-29f4-49ab-b83e-b5151679e3af	5	3328950	3332304	11	3354	1	3	7	NA	NA	NA
Skin-Melanoma	b35d9a68-29f4-49ab-b83e-b5151679e3af	5	42033141	42035543	12	2402	1	2	9	NA	NA	NA
Skin-Melanoma	b35d9a68-29f4-49ab-b83e-b5151679e3af	5	42748130	42751006	8	2876	NA	NA	8	NA	NA	NA
Skin-Melanoma	b35d9a68-29f4-49ab-b83e-b5151679e3af	5	43999210	44028149	32	28939	3	5	23	1	NA	NA
Skin-Melanoma	b35d9a68-29f4-49ab-b83e-b5151679e3af	5	115424056	115433842	27	9786	NA	NA	27	NA	NA	NA
Skin-Melanoma	b35d9a68-29f4-49ab-b83e-b5151679e3af	5	130415298	130422478	12	7180	1	NA	11	NA	NA	NA
Skin-Melanoma	b35d9a68-29f4-49ab-b83e-b5151679e3af	7	8742496	8742978	8	482	NA	2	6	NA	NA	NA
Skin-Melanoma	b35d9a68-29f4-49ab-b83e-b5151679e3af	7	9657331	9658701	8	1370	3	NA	3	2	NA	NA
Skin-Melanoma	b35d9a68-29f4-49ab-b83e-b5151679e3af	7	14415798	14421288	8	5490	1	1	3	NA	NA	3
Skin-Melanoma	b35d9a68-29f4-49ab-b83e-b5151679e3af	7	24052949	24057469	11	4520	3	4	4	NA	NA	NA
Skin-Melanoma	b35d9a68-29f4-49ab-b83e-b5151679e3af	7	80286094	80289590	12	3496	NA	7	5	NA	NA	NA
Skin-Melanoma	b35d9a68-29f4-49ab-b83e-b5151679e3af	7	86964719	86965738	9	1019	NA	1	8	NA	NA	NA
Skin-Melanoma	b35d9a68-29f4-49ab-b83e-b5151679e3af	7	157175300	157181264	11	5964	NA	NA	11	NA	NA	NA
Skin-Melanoma	b35d9a68-29f4-49ab-b83e-b5151679e3af	8	58025536	58030639	7	5103	NA	1	1	2	2	1
Skin-Melanoma	b35d9a68-29f4-49ab-b83e-b5151679e3af	11	58017709	58018652	8	943	NA	4	4	NA	NA	NA
Skin-Melanoma	b35d9a68-29f4-49ab-b83e-b5151679e3af	11	77868429	77875064	28	6635	NA	NA	28	NA	NA	NA
Skin-Melanoma	b35d9a68-29f4-49ab-b83e-b5151679e3af	11	93510076	93510641	9	565	NA	5	3	NA	NA	1
Skin-Melanoma	b35d9a68-29f4-49ab-b83e-b5151679e3af	11	96171320	96172582	10	1262	NA	2	8	NA	NA	NA
Skin-Melanoma	b35d9a68-29f4-49ab-b83e-b5151679e3af	11	106005098	106006192	9	1094	2	2	5	NA	NA	NA
Skin-Melanoma	b35d9a68-29f4-49ab-b83e-b5151679e3af	18	14749799	14752302	6	2503	1	5	NA	NA	NA	NA
Skin-Melanoma	b35d9a68-29f4-49ab-b83e-b5151679e3af	21	23764026	23767991	8	3965	1	3	3	1	NA	NA
Skin-Melanoma	b35d9a68-29f4-49ab-b83e-b5151679e3af	22	17488105	17494096	8	5991	2	2	4	NA	NA	NA
Skin-Melanoma	b35d9a68-29f4-49ab-b83e-b5151679e3af	22	31555447	31559035	7	3588	NA	2	5	NA	NA	NA
Uterus-AdenoCA	b38d0777-4901-48b8-9cdc-33b7f13a424f	16	77470503	77470864	10	361	1	4	5	NA	NA	NA
Skin-Melanoma	b3befa40-8f44-4eb6-ada0-ec395f460656	1	1472966	1473876	6	910	1	NA	3	1	NA	1
Skin-Melanoma	b3befa40-8f44-4eb6-ada0-ec395f460656	6	66480957	66488827	25	7870	5	7	13	NA	NA	NA
Skin-Melanoma	b3befa40-8f44-4eb6-ada0-ec395f460656	9	22550279	22556052	17	5773	NA	3	13	NA	NA	1
Skin-Melanoma	b3befa40-8f44-4eb6-ada0-ec395f460656	12	34479696	34484098	16	4402	2	5	9	NA	NA	NA
Skin-Melanoma	b3befa40-8f44-4eb6-ada0-ec395f460656	16	89699483	89703867	8	4384	NA	NA	8	NA	NA	NA
Skin-Melanoma	b3befa40-8f44-4eb6-ada0-ec395f460656	17	32172270	32174017	9	1747	1	2	6	NA	NA	NA

Skin-Melanoma	b3befa40-8f44-4eb6-ada0-ec395f460656	17	49036388	49042618	10	6230	NA	NA	NA	5	2	3
Prost-AdenoCA	b41c63e8-bb85-4f83-9bc6-e611fd9ef075	11	13756565	13759454	20	2889	1	13	5	1	NA	NA
Panc-AdenoCA	b47aa163-eec9-4225-940b-4373e78152e2	18	22734765	22736107	7	1342	1	2	4	NA	NA	NA
Stomach-AdenoCA	b49d5310-3cc5-4386-9444-cf0d61b52376	4	154986626	154988852	6	2226	1	NA	4	NA	1	NA
Panc-AdenoCA	b54b9433-ec10-4cb5-a860-4555da64917b	1	149284779	149286418	9	1639	NA	NA	9	NA	NA	NA
Panc-AdenoCA	b54b9433-ec10-4cb5-a860-4555da64917b	1	230623757	230626584	7	2827	1	NA	6	NA	NA	NA
Panc-AdenoCA	b54b9433-ec10-4cb5-a860-4555da64917b	4	180755747	180758023	6	2276	1	3	2	NA	NA	NA
Panc-AdenoCA	b54b9433-ec10-4cb5-a860-4555da64917b	5	158298043	158301193	7	3150	NA	1	6	NA	NA	NA
Panc-AdenoCA	b54b9433-ec10-4cb5-a860-4555da64917b	7	20820315	20821401	6	1086	2	3	1	NA	NA	NA
Panc-AdenoCA	b54b9433-ec10-4cb5-a860-4555da64917b	7	114869052	114875250	8	6198	5	1	2	NA	NA	NA
Panc-AdenoCA	b54b9433-ec10-4cb5-a860-4555da64917b	10	36429955	36432440	11	2485	1	5	5	NA	NA	NA
Panc-AdenoCA	b54b9433-ec10-4cb5-a860-4555da64917b	10	93786359	93787608	10	1249	2	4	4	NA	NA	NA
Panc-AdenoCA	b54b9433-ec10-4cb5-a860-4555da64917b	12	80601171	80603316	12	2145	NA	2	10	NA	NA	NA
Panc-AdenoCA	b54b9433-ec10-4cb5-a860-4555da64917b	13	108094773	108101562	27	6789	NA	9	15	NA	1	2
Panc-AdenoCA	b54b9433-ec10-4cb5-a860-4555da64917b	14	50019301	50019453	6	152	NA	3	3	NA	NA	NA
Panc-AdenoCA	b54b9433-ec10-4cb5-a860-4555da64917b	16	60656254	60660448	6	4194	1	3	2	NA	NA	NA
Panc-AdenoCA	b54b9433-ec10-4cb5-a860-4555da64917b	17	19869009	19870896	17	1887	2	3	12	NA	NA	NA
Panc-AdenoCA	b54b9433-ec10-4cb5-a860-4555da64917b	18	39105381	39106296	13	915	1	2	10	NA	NA	NA
Panc-AdenoCA	b54b9433-ec10-4cb5-a860-4555da64917b	19	46859671	46861101	6	1430	1	2	3	NA	NA	NA
Panc-AdenoCA	b54b9433-ec10-4cb5-a860-4555da64917b	X	93525408	93526179	7	771	1	4	2	NA	NA	NA
Panc-AdenoCA	b54b9433-ec10-4cb5-a860-4555da64917b	X	117165731	117166180	9	449	3	1	5	NA	NA	NA
Liver-HCC	b55751c4-c622-11e3-bf01-24c6515278c0	11	97933319	97933770	14	451	1	7	4	NA	NA	2
Panc-AdenoCA	b5cabba2-30a4-458e-897c-00ec3fefa6d2	18	25726769	25728739	6	1970	1	4	1	NA	NA	NA
Liver-HCC	b67208c4-c622-11e3-bf01-24c6515278c0	4	58778438	58784653	11	6215	NA	6	5	NA	NA	NA
Liver-HCC	b67208c4-c622-11e3-bf01-24c6515278c0	10	87381627	87389039	12	7412	1	2	9	NA	NA	NA
Panc-AdenoCA	b7008a98-5aa0-4cc9-9bee-af2a422266db	6	79096030	79096412	7	382	1	3	3	NA	NA	NA
Panc-AdenoCA	b7008a98-5aa0-4cc9-9bee-af2a422266db	7	86097935	86098199	8	264	3	3	1	NA	NA	1
Panc-AdenoCA	b7008a98-5aa0-4cc9-9bee-af2a422266db	12	24323382	24325513	8	2131	NA	1	7	NA	NA	NA
Panc-AdenoCA	b7008a98-5aa0-4cc9-9bee-af2a422266db	17	11419030	11429138	54	10108	NA	2	52	NA	NA	NA
Panc-AdenoCA	b7008a98-5aa0-4cc9-9bee-af2a422266db	18	28837319	28839065	7	1746	1	2	4	NA	NA	NA
Breast-AdenoCa	b752b444-f033-4be4-9d24-e5e80b4181af	18	19309751	19314747	8	4996	4	1	3	NA	NA	NA
Breast-AdenoCa	b752b444-f033-4be4-9d24-e5e80b4181af	20	23032937	23034704	14	1767	3	5	6	NA	NA	NA
Ovary-AdenoCA	b75b2663-dcc6-411c-bfcc-574aa33cf388	16	48168914	48171307	6	2393	NA	4	2	NA	NA	NA
Uterus-AdenoCA	b77084ab-8148-49e0-b3f9-90f7d9ea1862	11	57654886	57658112	6	3226	NA	2	4	NA	NA	NA
Uterus-AdenoCA	b77084ab-8148-49e0-b3f9-90f7d9ea1862	11	58285738	58290800	26	5062	1	9	14	NA	NA	2
Uterus-AdenoCA	b77084ab-8148-49e0-b3f9-90f7d9ea1862	11	58352455	58375343	26	22888	5	8	13	NA	NA	NA
Uterus-AdenoCA	b77084ab-8148-49e0-b3f9-90f7d9ea1862	11	59756244	59759595	17	3351	2	3	11	NA	NA	1
Uterus-AdenoCA	b77084ab-8148-49e0-b3f9-90f7d9ea1862	17	37715599	37718602	18	3003	6	3	9	NA	NA	NA
Uterus-AdenoCA	b77084ab-8148-49e0-b3f9-90f7d9ea1862	17	80773032	80775263	18	2231	3	6	9	NA	NA	NA
Lung-SCC	b7a7d93b-38a7-4fc3-a433-3bb0a8cb7c42	1	114648076	114649842	11	1766	1	2	8	NA	NA	NA
Lung-SCC	b7a7d93b-38a7-4fc3-a433-3bb0a8cb7c42	1	246156435	246161847	21	5412	2	8	11	NA	NA	NA
Lung-SCC	b7a7d93b-38a7-4fc3-a433-3bb0a8cb7c42	2	72959781	72960718	16	937	NA	NA	16	NA	NA	NA
Head-SCC	b86e88e7-0d5f-4b32-a35f-dc97251ab990	2	106108517	106115117	13	6600	1	6	5	NA	1	NA
Head-SCC	b86e88e7-0d5f-4b32-a35f-dc97251ab990	2	154606925	154610965	8	4040	NA	2	6	NA	NA	NA
Head-SCC	b86e88e7-0d5f-4b32-a35f-dc97251ab990	3	50047028	50050292	6	3264	NA	3	3	NA	NA	NA
Head-SCC	b86e88e7-0d5f-4b32-a35f-dc97251ab990	6	37716298	37723906	13	7608	2	4	7	NA	NA	NA
Head-SCC	b86e88e7-0d5f-4b32-a35f-dc97251ab990	8	25229120	25232544	7	3424	2	3	2	NA	NA	NA
Head-SCC	b86e88e7-0d5f-4b32-a35f-dc97251ab990	10	109353297	109359066	8	5769	NA	3	5	NA	NA	NA
Head-SCC	b86e88e7-0d5f-4b32-a35f-dc97251ab990	12	124082940	124085180	6	2240	2	3	1	NA	NA	NA
Eso-AdenoCa	b8f3137e-5e92-4a56-90d4-884a4ed2ef9c	3	136406695	136410757	7	4062	1	3	3	NA	NA	NA
Eso-AdenoCa	b8f3137e-5e92-4a56-90d4-884a4ed2ef9c	5	73055270	73058820	6	3550	NA	4	1	1	NA	NA
Eso-AdenoCa	b8f3137e-5e92-4a56-90d4-884a4ed2ef9c	7	50194250	50194770	12	520	NA	NA	NA	12	NA	NA
Eso-AdenoCa	b8f3137e-5e92-4a56-90d4-884a4ed2ef9c	13	45284020	45285720	9	1700	NA	NA	NA	9	NA	NA
Eso-AdenoCa	b8f3137e-5e92-4a56-90d4-884a4ed2ef9c	19	46660314	46660973	6	659	NA	5	1	NA	NA	NA
Eso-AdenoCa	b8f3137e-5e92-4a56-90d4-884a4ed2ef9c	X	99930125	99931358	6	1233	1	4	1	NA	NA	NA
Liver-HCC	b994762c-c622-11e3-bf01-24c6515278c0	6	7048752	7051424	15	2672	5	6	4	NA	NA	NA
Eso-AdenoCa	b9bcba3a-c9be-4305-b5f4-c861282921e0	1	83003403	83004764	11	1361	NA	NA	NA	5	4	2
Eso-AdenoCa	b9bcba3a-c9be-4305-b5f4-c861282921e0	2	81659686	81663739	6	4053	NA	1	1	1	2	1
Eso-AdenoCa	b9bcba3a-c9be-4305-b5f4-c861282921e0	2	215482904	215489960	9	7056	NA	NA	2	3	2	2
Eso-AdenoCa	b9bcba3a-c9be-4305-b5f4-c861282921e0	3	163720257	163722838	6	2581	1	NA	1	1	NA	3

Eso-AdenoCa	b9bcba3a-c9be-4305-b5f4-c861282921e0	4	158009929	158012047	6	2118	1	NA	NA	1	3	1
Eso-AdenoCa	b9bcba3a-c9be-4305-b5f4-c861282921e0	5	163873748	163877165	10	3417	NA	NA	1	3	4	2
Eso-AdenoCa	b9bcba3a-c9be-4305-b5f4-c861282921e0	9	28206043	28207651	6	1608	NA	NA	NA	NA	5	1
Eso-AdenoCa	b9bcba3a-c9be-4305-b5f4-c861282921e0	12	84641835	84644391	6	2556	NA	NA	NA	3	NA	3
Eso-AdenoCa	b9bcba3a-c9be-4305-b5f4-c861282921e0	13	56213358	56217681	6	4323	NA	NA	NA	1	1	4
Eso-AdenoCa	b9bcba3a-c9be-4305-b5f4-c861282921e0	13	65433242	65438826	7	5584	NA	NA	NA	3	2	2
Eso-AdenoCa	b9bcba3a-c9be-4305-b5f4-c861282921e0	13	67829724	67835559	8	5835	NA	NA	NA	3	2	3
Eso-AdenoCa	b9bcba3a-c9be-4305-b5f4-c861282921e0	13	90676813	90683652	9	6839	NA	NA	NA	4	3	2
Eso-AdenoCa	b9bcba3a-c9be-4305-b5f4-c861282921e0	13	92619691	92620443	7	752	NA	NA	NA	5	1	1
Eso-AdenoCa	b9bcba3a-c9be-4305-b5f4-c861282921e0	18	40272205	40276347	6	4142	2	NA	1	1	2	NA
Eso-AdenoCa	b9bcba3a-c9be-4305-b5f4-c861282921e0	X	140943625	140946713	8	3088	2	NA	NA	4	1	1
Cervix-AdenoCA	b9d1a64e-d445-4174-a5b4-76dd6ea69419	10	134189077	134192981	9	3904	NA	2	7	NA	NA	NA
Lymph-BNHL	b9dbc78e-44ce-427c-bbeb-de8d097bde2c	2	136873464	136875888	15	2424	NA	5	5	4	NA	1
Lymph-BNHL	b9dbc78e-44ce-427c-bbeb-de8d097bde2c	3	32021891	32024016	20	2125	2	6	9	1	1	1
Lymph-BNHL	b9dbc78e-44ce-427c-bbeb-de8d097bde2c	4	49620	51904	7	2284	1	2	NA	2	NA	2
Lymph-BNHL	b9dbc78e-44ce-427c-bbeb-de8d097bde2c	4	40199023	40201830	9	2807	1	NA	4	2	2	NA
Lymph-BNHL	b9dbc78e-44ce-427c-bbeb-de8d097bde2c	9	37025361	37026776	7	1415	NA	2	4	NA	1	NA
Lymph-BNHL	b9dbc78e-44ce-427c-bbeb-de8d097bde2c	11	65266612	65267472	6	860	NA	NA	1	2	2	1
Lymph-BNHL	b9dbc78e-44ce-427c-bbeb-de8d097bde2c	12	25206818	25213059	13	6241	1	5	2	1	2	2
Lymph-BNHL	b9dbc78e-44ce-427c-bbeb-de8d097bde2c	12	92538069	92539302	15	1233	3	3	4	2	2	1
Lymph-BNHL	b9dbc78e-44ce-427c-bbeb-de8d097bde2c	12	113494725	113496475	10	1750	2	1	5	NA	2	NA
Lymph-BNHL	b9dbc78e-44ce-427c-bbeb-de8d097bde2c	13	46946467	46948706	10	2239	NA	1	8	1	NA	NA
Lymph-BNHL	b9dbc78e-44ce-427c-bbeb-de8d097bde2c	13	46959249	46961141	11	1892	3	1	6	NA	1	NA
Lymph-BNHL	b9dbc78e-44ce-427c-bbeb-de8d097bde2c	14	69257956	69259538	8	1582	1	3	1	NA	1	2
Lymph-BNHL	b9dbc78e-44ce-427c-bbeb-de8d097bde2c	14	96178902	96180184	10	1282	2	4	1	1	1	1
Lymph-BNHL	b9dbc78e-44ce-427c-bbeb-de8d097bde2c	14	106209410	106213368	12	3958	1	2	9	NA	NA	NA
Lymph-BNHL	b9dbc78e-44ce-427c-bbeb-de8d097bde2c	14	106320319	106329069	42	8750	6	8	20	2	3	3
Lymph-BNHL	b9dbc78e-44ce-427c-bbeb-de8d097bde2c	14	106387128	106391285	10	4157	NA	1	9	NA	NA	NA
Lymph-BNHL	b9dbc78e-44ce-427c-bbeb-de8d097bde2c	17	75446089	75450404	9	4315	1	3	5	NA	NA	NA
Lymph-BNHL	b9dbc78e-44ce-427c-bbeb-de8d097bde2c	22	22712125	22712521	9	396	NA	2	3	NA	1	3
Lymph-BNHL	b9dbc78e-44ce-427c-bbeb-de8d097bde2c	22	23230078	23249096	65	19018	4	14	30	8	4	5
Eso-AdenoCa	ba096d4f-5a6c-4c31-ae03-e7483cf58c38	11	100039832	100043233	6	3401	2	1	2	1	NA	NA
Eso-AdenoCa	ba096d4f-5a6c-4c31-ae03-e7483cf58c38	12	50206533	50207595	11	1062	1	7	3	NA	NA	NA
Lymph-BNHL	baa02e74-9cae-4996-858e-17d9f52d924b	1	222857128	222857366	6	238	NA	NA	NA	2	1	3
Lymph-BNHL	baa02e74-9cae-4996-858e-17d9f52d924b	2	65590827	65593668	6	2841	2	1	NA	2	1	NA
Lymph-BNHL	baa02e74-9cae-4996-858e-17d9f52d924b	2	89140636	89145911	7	5275	NA	NA	NA	NA	5	2
Lymph-BNHL	baa02e74-9cae-4996-858e-17d9f52d924b	2	89154304	89160450	65	6146	2	7	9	27	4	16
Lymph-BNHL	baa02e74-9cae-4996-858e-17d9f52d924b	3	187460824	187463283	26	2459	NA	6	6	4	3	7
Lymph-BNHL	baa02e74-9cae-4996-858e-17d9f52d924b	3	187658409	187661267	10	2858	1	4	2	1	2	NA
Lymph-BNHL	baa02e74-9cae-4996-858e-17d9f52d924b	3	187957504	187962068	10	4564	NA	2	NA	3	1	4
Lymph-BNHL	baa02e74-9cae-4996-858e-17d9f52d924b	3	188471627	188472033	7	406	NA	1	NA	4	1	1
Lymph-BNHL	baa02e74-9cae-4996-858e-17d9f52d924b	4	25863537	25865750	7	2213	NA	1	2	4	NA	NA
Lymph-BNHL	baa02e74-9cae-4996-858e-17d9f52d924b	4	40195502	40200350	11	4848	NA	NA	3	6	1	1
Lymph-BNHL	baa02e74-9cae-4996-858e-17d9f52d924b	5	15551119	15555096	6	3977	NA	NA	NA	4	2	NA
Lymph-BNHL	baa02e74-9cae-4996-858e-17d9f52d924b	6	91000864	91006663	16	5799	1	2	1	5	5	2
Lymph-BNHL	baa02e74-9cae-4996-858e-17d9f52d924b	7	105730255	105730387	7	132	NA	NA	NA	5	2	NA
Lymph-BNHL	baa02e74-9cae-4996-858e-17d9f52d924b	7	110663313	110667705	7	4392	NA	NA	1	1	1	4
Lymph-BNHL	baa02e74-9cae-4996-858e-17d9f52d924b	7	110719406	110724916	7	5510	1	NA	1	1	2	2
Lymph-BNHL	baa02e74-9cae-4996-858e-17d9f52d924b	7	110736169	110742749	8	6580	NA	NA	1	2	2	3
Lymph-BNHL	baa02e74-9cae-4996-858e-17d9f52d924b	7	110765186	110774548	13	9362	NA	NA	NA	6	2	5
Lymph-BNHL	baa02e74-9cae-4996-858e-17d9f52d924b	12	113495518	113496639	6	1121	2	NA	1	2	1	NA
Lymph-BNHL	baa02e74-9cae-4996-858e-17d9f52d924b	12	122458040	122463800	26	5760	2	2	6	8	4	4
Lymph-BNHL	baa02e74-9cae-4996-858e-17d9f52d924b	14	106067590	106072065	13	4475	2	5	4	2	NA	NA
Lymph-BNHL	baa02e74-9cae-4996-858e-17d9f52d924b	14	106240367	106241976	8	1609	1	NA	7	NA	NA	NA
Lymph-BNHL	baa02e74-9cae-4996-858e-17d9f52d924b	14	106323083	106328864	85	5781	5	16	33	14	8	9
Lymph-BNHL	baa02e74-9cae-4996-858e-17d9f52d924b	14	106493788	106494363	14	575	1	1	3	5	2	2
Lymph-BNHL	baa02e74-9cae-4996-858e-17d9f52d924b	16	10972330	10973520	7	1190	NA	2	5	NA	NA	NA
Lymph-BNHL	baa02e74-9cae-4996-858e-17d9f52d924b	16	62013900	62016875	7	2975	NA	1	NA	2	2	2
Lymph-BNHL	baa02e74-9cae-4996-858e-17d9f52d924b	16	85933048	85934446	17	1398	1	2	3	2	7	2
Lymph-BNHL	baa02e74-9cae-4996-858e-17d9f52d924b	18	60805688	60809499	8	3811	NA	2	1	2	1	2

Lymph-BNHL	baa02e74-9cae-4996-858e-17d9f52d924b	18	60873246	60876259	7	3013	NA	1	1	1	3	1
Lymph-BNHL	baa02e74-9cae-4996-858e-17d9f52d924b	18	60984826	60988444	33	3618	NA	7	9	9	4	4
Lymph-BNHL	baa02e74-9cae-4996-858e-17d9f52d924b	19	10340147	10341506	11	1359	3	6	NA	2	NA	NA
Lymph-BNHL	baa02e74-9cae-4996-858e-17d9f52d924b	22	23198470	23199408	14	938	NA	2	2	5	2	3
Lymph-BNHL	baa02e74-9cae-4996-858e-17d9f52d924b	22	23223024	23288986	91	65962	4	14	23	26	11	13
Lymph-BNHL	baa02e74-9cae-4996-858e-17d9f52d924b	22	29195775	29196242	7	467	NA	2	2	2	1	NA
Lymph-BNHL	baa02e74-9cae-4996-858e-17d9f52d924b	X	33144963	33146418	8	1455	NA	NA	1	2	NA	5
Eso-AdenoCa	bb5bedd3-d8fc-4739-8e6b-9e37223f8be2	5	25253317	25257350	6	4033	NA	NA	NA	2	3	1
Eso-AdenoCa	bb5bedd3-d8fc-4739-8e6b-9e37223f8be2	6	154761490	154761946	9	456	2	2	5	NA	NA	NA
ColoRect-AdenoCA	bb6150f7-23e4-40f2-b466-6b6edd19c502	2	45071056	45071951	11	895	2	2	7	NA	NA	NA
Biliary-AdenoCA	bb65d670-0411-4fc5-a6cb-019caec1ab36	1	62039296	62041385	8	2089	NA	NA	6	2	NA	NA
Biliary-AdenoCA	bb65d670-0411-4fc5-a6cb-019caec1ab36	2	62503099	62503952	9	853	1	3	5	NA	NA	NA
Biliary-AdenoCA	bb65d670-0411-4fc5-a6cb-019caec1ab36	2	164158117	164158486	9	369	NA	1	8	NA	NA	NA
Biliary-AdenoCA	bb65d670-0411-4fc5-a6cb-019caec1ab36	2	240523919	240529419	7	5500	NA	NA	7	NA	NA	NA
Biliary-AdenoCA	bb65d670-0411-4fc5-a6cb-019caec1ab36	3	135923867	135924905	7	1038	1	NA	6	NA	NA	NA
Biliary-AdenoCA	bb65d670-0411-4fc5-a6cb-019caec1ab36	5	1739796	1741338	12	1542	1	2	7	NA	1	1
Biliary-AdenoCA	bb65d670-0411-4fc5-a6cb-019caec1ab36	5	2416797	2417998	11	1201	1	3	7	NA	NA	NA
Biliary-AdenoCA	bb65d670-0411-4fc5-a6cb-019caec1ab36	5	84304368	84320993	43	16625	5	12	25	1	NA	NA
Biliary-AdenoCA	bb65d670-0411-4fc5-a6cb-019caec1ab36	5	85181445	85182439	7	994	1	2	4	NA	NA	NA
Biliary-AdenoCA	bb65d670-0411-4fc5-a6cb-019caec1ab36	5	86811498	86812491	12	993	2	1	9	NA	NA	NA
Biliary-AdenoCA	bb65d670-0411-4fc5-a6cb-019caec1ab36	6	57287951	57293276	20	5325	5	8	7	NA	NA	NA
Biliary-AdenoCA	bb65d670-0411-4fc5-a6cb-019caec1ab36	6	69516031	69516494	7	463	1	2	4	NA	NA	NA
Biliary-AdenoCA	bb65d670-0411-4fc5-a6cb-019caec1ab36	6	91062487	91063395	8	908	1	3	4	NA	NA	NA
Biliary-AdenoCA	bb65d670-0411-4fc5-a6cb-019caec1ab36	6	101243413	101243757	8	344	1	1	5	NA	NA	1
Biliary-AdenoCA	bb65d670-0411-4fc5-a6cb-019caec1ab36	6	119130604	119133020	13	2416	1	3	9	NA	NA	NA
Biliary-AdenoCA	bb65d670-0411-4fc5-a6cb-019caec1ab36	9	578848	580587	8	1739	NA	NA	8	NA	NA	NA
Biliary-AdenoCA	bb65d670-0411-4fc5-a6cb-019caec1ab36	12	29979652	29985439	25	5787	4	8	12	NA	NA	1
Biliary-AdenoCA	bb65d670-0411-4fc5-a6cb-019caec1ab36	14	26485931	26488781	14	2850	NA	NA	14	NA	NA	NA
Biliary-AdenoCA	bb65d670-0411-4fc5-a6cb-019caec1ab36	14	34344113	34344309	11	196	2	1	8	NA	NA	NA
Biliary-AdenoCA	bb65d670-0411-4fc5-a6cb-019caec1ab36	20	44515003	44517309	10	2306	NA	2	7	1	NA	NA
Biliary-AdenoCA	bb65d670-0411-4fc5-a6cb-019caec1ab36	20	45274358	45278806	8	4448	NA	5	3	NA	NA	NA
Biliary-AdenoCA	bb65d670-0411-4fc5-a6cb-019caec1ab36	X	68798924	68813113	24	14189	3	12	9	NA	NA	NA
Panc-AdenoCA	bb8176a7-ea7c-42d7-a85a-cec1ca386f2e	17	31111881	31118882	16	7001	1	6	9	NA	NA	NA
Liver-HCC	bba106ce-c622-11e3-bf01-24c6515278c0	18	1962174	1966025	13	3851	1	7	5	NA	NA	NA
Liver-HCC	bba106ce-c622-11e3-bf01-24c6515278c0	19	15451315	15454182	21	2867	NA	8	13	NA	NA	NA
Ovary-AdenoCA	bbb2cf2f-8f32-43d4-846c-d1020e6329ec	12	19761930	19764568	6	2638	1	2	2	1	NA	NA
Ovary-AdenoCA	bbb2cf2f-8f32-43d4-846c-d1020e6329ec	12	21803858	21808619	7	4761	2	NA	5	NA	NA	NA
Panc-AdenoCA	bbdd7393-024b-4073-b5bc-ef7a68c15b8a	X	139185890	139185900	9	10	1	NA	2	4	NA	2
Panc-AdenoCA	bbff7954-95ec-455c-b0bc-92f67a09ee77	1	158808887	158809464	6	577	1	NA	5	NA	NA	NA
Panc-AdenoCA	bbff7954-95ec-455c-b0bc-92f67a09ee77	15	61460779	61462309	6	1530	NA	3	3	NA	NA	NA
Panc-AdenoCA	bbff7954-95ec-455c-b0bc-92f67a09ee77	15	66835290	66836926	7	1636	NA	2	5	NA	NA	NA
Panc-AdenoCA	bbff7954-95ec-455c-b0bc-92f67a09ee77	15	98464380	98464881	8	501	2	1	5	NA	NA	NA
Panc-AdenoCA	bbff7954-95ec-455c-b0bc-92f67a09ee77	15	102086175	102086915	7	740	NA	1	6	NA	NA	NA
Panc-AdenoCA	bbff7954-95ec-455c-b0bc-92f67a09ee77	15	102107425	102117495	18	10070	1	2	15	NA	NA	NA
Panc-AdenoCA	bbff7954-95ec-455c-b0bc-92f67a09ee77	18	25196705	25198831	6	2126	1	1	4	NA	NA	NA
Panc-AdenoCA	bbff7954-95ec-455c-b0bc-92f67a09ee77	20	4919069	4919892	9	823	NA	3	6	NA	NA	NA
Panc-AdenoCA	bbff7954-95ec-455c-b0bc-92f67a09ee77	20	6729174	6729489	7	315	2	3	2	NA	NA	NA
Stomach-AdenoCA	bc0dee07-de20-44d6-be65-05af7e63ac96	4	73042895	73043409	6	514	1	NA	4	1	NA	NA
Stomach-AdenoCA	bc0dee07-de20-44d6-be65-05af7e63ac96	22	43977562	43993502	22	15940	5	6	11	NA	NA	NA
Stomach-AdenoCA	bc0dee07-de20-44d6-be65-05af7e63ac96	22	44015722	44019690	8	3968	2	NA	6	NA	NA	NA
Stomach-AdenoCA	bc0dee07-de20-44d6-be65-05af7e63ac96	X	85288607	85299720	17	11113	2	5	10	NA	NA	NA
Lymph-BNHL	bc1d5327-2e76-4e0e-b749-72a559469d0d	8	88769256	88772566	6	3310	1	NA	1	4	NA	NA
Lymph-BNHL	bc1d5327-2e76-4e0e-b749-72a559469d0d	8	88777000	88796913	26	19913	2	2	3	9	1	9
Lymph-BNHL	bc1d5327-2e76-4e0e-b749-72a559469d0d	14	106210543	106213791	7	3248	1	2	4	NA	NA	NA
Lymph-BNHL	bc1d5327-2e76-4e0e-b749-72a559469d0d	14	106326843	106351024	33	24181	5	9	11	6	1	1
Panc-AdenoCA	bcef0b6c-6584-4090-9d28-ef784a7e5fbb	10	134436743	134449208	16	12465	5	6	5	NA	NA	NA
Panc-AdenoCA	bcef0b6c-6584-4090-9d28-ef784a7e5fbb	16	30838738	30851260	24	12522	NA	6	17	NA	NA	1
Skin-Melanoma	bcf76f1a-7109-422d-94c9-5e8364895fa0	1	4519055	4523471	6	4416	2	3	1	NA	NA	NA
Skin-Melanoma	bcf76f1a-7109-422d-94c9-5e8364895fa0	2	84673377	84676992	10	3615	NA	5	5	NA	NA	NA
Skin-Melanoma	bcf76f1a-7109-422d-94c9-5e8364895fa0	2	184758037	184759608	7	1571	3	4	NA	NA	NA	NA

Skin-Melanoma	bcf76f1a-7109-422d-94c9-5e8364895fa0	2	188601839	188604099	13	2260	4	4	5	NA	NA	NA
Skin-Melanoma	bcf76f1a-7109-422d-94c9-5e8364895fa0	2	189946260	189949111	10	2851	2	2	6	NA	NA	NA
Skin-Melanoma	bcf76f1a-7109-422d-94c9-5e8364895fa0	2	191025410	191027673	8	2263	1	6	1	NA	NA	NA
Skin-Melanoma	bcf76f1a-7109-422d-94c9-5e8364895fa0	3	89597042	89599751	10	2709	2	5	3	NA	NA	NA
Skin-Melanoma	bcf76f1a-7109-422d-94c9-5e8364895fa0	4	176023227	176026270	11	3043	2	3	6	NA	NA	NA
Skin-Melanoma	bcf76f1a-7109-422d-94c9-5e8364895fa0	8	3789083	3789331	6	248	NA	NA	6	NA	NA	NA
Skin-Melanoma	bcf76f1a-7109-422d-94c9-5e8364895fa0	8	136974660	136980734	11	6074	2	2	7	NA	NA	NA
Skin-Melanoma	bcf76f1a-7109-422d-94c9-5e8364895fa0	11	17127551	17131089	6	3538	NA	2	4	NA	NA	NA
Skin-Melanoma	bcf76f1a-7109-422d-94c9-5e8364895fa0	17	9225405	9228584	8	3179	1	6	NA	NA	NA	1
Skin-Melanoma	bcf76f1a-7109-422d-94c9-5e8364895fa0	X	5590798	5591669	9	871	NA	7	2	NA	NA	NA
Skin-Melanoma	bcf76f1a-7109-422d-94c9-5e8364895fa0	X	137906058	137908684	9	2626	2	5	2	NA	NA	NA
Skin-Melanoma	bcf76f1a-7109-422d-94c9-5e8364895fa0	Y	18926563	18940532	34	13969	3	13	17	NA	NA	1
Stomach-AdenoCA	bd2f8133-3e71-4c5e-998f-0c1a9fdfb75f	11	3715686	3717504	6	1818	NA	5	1	NA	NA	NA
Lymph-BNHL	bd403458-5154-488b-931a-a7e737a6bf8c	1	203275229	203275856	7	627	NA	1	2	1	1	2
Lymph-BNHL	bd403458-5154-488b-931a-a7e737a6bf8c	2	89127404	89128775	10	1371	1	2	NA	2	3	2
Lymph-BNHL	bd403458-5154-488b-931a-a7e737a6bf8c	2	89137538	89142675	12	5137	1	1	1	5	3	1
Lymph-BNHL	bd403458-5154-488b-931a-a7e737a6bf8c	2	89154806	89327165	202	2E+05	10	19	21	64	32	56
Lymph-BNHL	bd403458-5154-488b-931a-a7e737a6bf8c	2	136874767	136875527	13	760	1	2	5	3	NA	2
Lymph-BNHL	bd403458-5154-488b-931a-a7e737a6bf8c	2	204400600	204407893	14	7293	1	NA	3	5	NA	5
Lymph-BNHL	bd403458-5154-488b-931a-a7e737a6bf8c	3	2133637	2138000	7	4363	1	NA	NA	3	1	2
Lymph-BNHL	bd403458-5154-488b-931a-a7e737a6bf8c	3	187462250	187463783	18	1533	1	7	2	3	3	2
Lymph-BNHL	bd403458-5154-488b-931a-a7e737a6bf8c	3	187957308	187959934	21	2626	NA	1	3	9	6	2
Lymph-BNHL	bd403458-5154-488b-931a-a7e737a6bf8c	3	188471260	188473993	7	2733	NA	2	1	1	3	NA
Lymph-BNHL	bd403458-5154-488b-931a-a7e737a6bf8c	4	40193834	40202724	18	8890	NA	1	6	2	4	5
Lymph-BNHL	bd403458-5154-488b-931a-a7e737a6bf8c	5	90028105	90032972	12	4867	3	1	NA	5	1	2
Lymph-BNHL	bd403458-5154-488b-931a-a7e737a6bf8c	6	31540653	31549999	26	9346	4	4	6	4	5	3
Lymph-BNHL	bd403458-5154-488b-931a-a7e737a6bf8c	6	91005225	91007018	8	1793	NA	1	3	2	2	NA
Lymph-BNHL	bd403458-5154-488b-931a-a7e737a6bf8c	6	134493478	134536616	73	43138	2	10	17	15	14	15
Lymph-BNHL	bd403458-5154-488b-931a-a7e737a6bf8c	7	14333031	14338665	7	5634	NA	NA	2	1	1	3
Lymph-BNHL	bd403458-5154-488b-931a-a7e737a6bf8c	7	14341994	14344576	7	2582	NA	NA	NA	2	1	4
Lymph-BNHL	bd403458-5154-488b-931a-a7e737a6bf8c	11	102188510	102189479	7	969	NA	1	3	1	NA	2
Lymph-BNHL	bd403458-5154-488b-931a-a7e737a6bf8c	12	122459042	122466517	16	7475	1	1	4	5	4	1
Lymph-BNHL	bd403458-5154-488b-931a-a7e737a6bf8c	14	43108069	43109843	6	1774	NA	NA	1	2	2	1
Lymph-BNHL	bd403458-5154-488b-931a-a7e737a6bf8c	14	69258223	69259538	25	1315	3	6	2	7	3	4
Lymph-BNHL	bd403458-5154-488b-931a-a7e737a6bf8c	14	96107071	96107789	8	718	1	1	NA	2	4	NA
Lymph-BNHL	bd403458-5154-488b-931a-a7e737a6bf8c	14	96178241	96180232	19	1991	1	2	4	4	6	2
Lymph-BNHL	bd403458-5154-488b-931a-a7e737a6bf8c	14	106113089	106114751	8	1662	2	2	2	2	NA	NA
Lymph-BNHL	bd403458-5154-488b-931a-a7e737a6bf8c	14	106208446	106239851	47	31405	5	11	22	2	3	4
Lymph-BNHL	bd403458-5154-488b-931a-a7e737a6bf8c	14	106326629	106363883	124	37254	8	19	24	40	16	17
Lymph-BNHL	bd403458-5154-488b-931a-a7e737a6bf8c	14	107131429	107132693	30	1264	3	7	7	7	1	5
Lymph-BNHL	bd403458-5154-488b-931a-a7e737a6bf8c	14	107169986	107179917	33	9931	1	6	9	9	4	4
Lymph-BNHL	bd403458-5154-488b-931a-a7e737a6bf8c	15	22532231	22535590	6	3359	3	2	NA	NA	1	NA
Lymph-BNHL	bd403458-5154-488b-931a-a7e737a6bf8c	17	56408971	56409696	7	725	NA	2	5	NA	NA	NA
Lymph-BNHL	bd403458-5154-488b-931a-a7e737a6bf8c	22	23198193	23199495	10	1302	2	3	3	1	NA	1
Lymph-BNHL	bd403458-5154-488b-931a-a7e737a6bf8c	22	23220067	23282817	135	62750	7	16	34	32	23	23
Lymph-BNHL	bd403458-5154-488b-931a-a7e737a6bf8c	X	12993351	12994912	9	1561	1	2	4	1	1	NA
Lymph-BNHL	bd6bd940-ef06-433a-80d9-dca5683e9cd2	3	187461782	187462696	6	914	NA	1	3	2	NA	NA
Lymph-BNHL	bd6bd940-ef06-433a-80d9-dca5683e9cd2	14	106069050	106071165	11	2115	NA	1	10	NA	NA	NA
Lymph-BNHL	bd6bd940-ef06-433a-80d9-dca5683e9cd2	14	106324498	106330196	87	5698	6	22	40	6	6	7
Lymph-BNHL	bd6bd940-ef06-433a-80d9-dca5683e9cd2	14	106994193	106994885	7	692	NA	2	2	1	1	1
Lymph-BNHL	bd6bd940-ef06-433a-80d9-dca5683e9cd2	18	60984901	60988726	30	3825	3	3	14	6	3	1
Lymph-BNHL	bd6bd940-ef06-433a-80d9-dca5683e9cd2	22	22380726	22385647	7	4921	NA	NA	5	2	NA	NA
Lymph-BNHL	bdbcffb0-c7da-4e7d-8002-9feffe48533c	14	106325793	106330101	35	4308	2	6	8	4	12	3
Lymph-BNHL	bdbcffb0-c7da-4e7d-8002-9feffe48533c	14	106691995	106692268	9	273	1	NA	1	4	2	1
Lymph-BNHL	bdbcffb0-c7da-4e7d-8002-9feffe48533c	18	60984884	60988229	6	3345	NA	3	2	NA	NA	1
Liver-HCC	bdeeb1a6-c622-11e3-bf01-24c6515278c0	16	75176306	75176770	8	464	NA	NA	1	2	4	1
Prost-AdenoCA	beafb7c6-ef39-478e-aa47-0f1ad39f95a3	2	196727991	196728625	6	634	1	1	4	NA	NA	NA
Lung-AdenoCA	bf7462a2-394f-4838-bcb6-4d0126fa48b1	1	240092967	240099169	23	6202	6	5	11	NA	NA	1
Lung-AdenoCA	bf7462a2-394f-4838-bcb6-4d0126fa48b1	6	19611418	19613354	6	1936	NA	1	2	1	2	NA
Lung-AdenoCA	bf7462a2-394f-4838-bcb6-4d0126fa48b1	9	6447422	6450993	10	3571	NA	3	7	NA	NA	NA

Lung-AdenoCA	bf7462a2-394f-4838-bcb6-4d0126fa48b1	13	30412723	30413097	11	374	NA	5	6	NA	NA	NA
Lung-AdenoCA	bf7462a2-394f-4838-bcb6-4d0126fa48b1	16	90062956	90068484	7	5528	1	3	3	NA	NA	NA
Lung-AdenoCA	bf7462a2-394f-4838-bcb6-4d0126fa48b1	19	34267710	34268090	16	380	NA	4	11	1	NA	NA
Lung-AdenoCA	bf7462a2-394f-4838-bcb6-4d0126fa48b1	19	35977576	35978071	8	495	NA	NA	8	NA	NA	NA
Ovary-AdenoCA	bff518fb-6da7-4dfc-ae4c-bd3f641028e2	9	122221085	122223012	10	1927	NA	2	8	NA	NA	NA
Liver-HCC	c016d025-3c92-41c5-b846-493b1fcce79e	4	35004389	35005191	8	802	2	4	1	NA	NA	1
Liver-HCC	c016d025-3c92-41c5-b846-493b1fcce79e	5	30944949	30948645	14	3696	NA	3	2	4	5	NA
Liver-HCC	c016d025-3c92-41c5-b846-493b1fcce79e	5	37188369	37192197	13	3828	NA	8	4	NA	1	NA
Liver-HCC	c016d025-3c92-41c5-b846-493b1fcce79e	14	34361337	34362833	11	1496	3	4	3	NA	1	NA
Liver-HCC	c016d025-3c92-41c5-b846-493b1fcce79e	19	10139467	10143101	7	3634	NA	3	3	1	NA	NA
Panc-AdenoCA	c0523251-3ac2-4292-bb00-9ae9ea9009f6	12	72467650	72468626	9	976	NA	5	4	NA	NA	NA
Panc-AdenoCA	c0523251-3ac2-4292-bb00-9ae9ea9009f6	13	46041043	46042909	11	1866	2	2	7	NA	NA	NA
Panc-AdenoCA	c0523251-3ac2-4292-bb00-9ae9ea9009f6	16	87319905	87327398	9	7493	NA	3	6	NA	NA	NA
Liver-HCC	c05adc19-2e01-4712-a35a-69eee4d40998	1	98337528	98337736	8	208	NA	8	NA	NA	NA	NA
Liver-HCC	c05adc19-2e01-4712-a35a-69eee4d40998	1	190122704	190126969	9	4265	1	NA	7	NA	NA	1
Liver-HCC	c05adc19-2e01-4712-a35a-69eee4d40998	5	16237724	16239146	8	1422	NA	NA	7	1	NA	NA
Liver-HCC	c05adc19-2e01-4712-a35a-69eee4d40998	7	109018279	109019101	6	822	NA	1	NA	2	NA	3
Liver-HCC	c05adc19-2e01-4712-a35a-69eee4d40998	8	78436335	78438334	6	1999	1	5	NA	NA	NA	NA
Liver-HCC	c05adc19-2e01-4712-a35a-69eee4d40998	8	90866372	90871629	25	5257	3	14	8	NA	NA	NA
Liver-HCC	c05adc19-2e01-4712-a35a-69eee4d40998	11	70670058	70670950	9	892	NA	NA	9	NA	NA	NA
Liver-HCC	c05adc19-2e01-4712-a35a-69eee4d40998	11	75764506	75770271	9	5765	NA	NA	NA	2	6	1
Liver-HCC	c05adc19-2e01-4712-a35a-69eee4d40998	12	34767667	34770522	11	2855	NA	NA	11	NA	NA	NA
Liver-HCC	c05adc19-2e01-4712-a35a-69eee4d40998	13	40807311	40808413	7	1102	1	NA	6	NA	NA	NA
Liver-HCC	c05adc19-2e01-4712-a35a-69eee4d40998	17	53248273	53249023	7	750	1	3	3	NA	NA	NA
Liver-HCC	c05adc19-2e01-4712-a35a-69eee4d40998	17	53273189	53278253	7	5064	NA	3	4	NA	NA	NA
Liver-HCC	c05adc19-2e01-4712-a35a-69eee4d40998	X	86711786	86713190	8	1404	NA	1	4	2	NA	1
CNS-GBM	c065761d-f775-457f-bda0-4c7c257a701e	1	50531169	50534859	6	3690	NA	5	1	NA	NA	NA
CNS-GBM	c065761d-f775-457f-bda0-4c7c257a701e	4	55500043	55503801	9	3758	2	3	4	NA	NA	NA
CNS-GBM	c065761d-f775-457f-bda0-4c7c257a701e	4	62900282	62907163	11	6881	1	7	2	1	NA	NA
CNS-GBM	c065761d-f775-457f-bda0-4c7c257a701e	4	116938129	116940920	8	2791	1	6	NA	1	NA	NA
CNS-GBM	c065761d-f775-457f-bda0-4c7c257a701e	4	116974093	116979589	7	5496	3	2	2	NA	NA	NA
CNS-GBM	c065761d-f775-457f-bda0-4c7c257a701e	4	131507359	131508878	14	1519	1	7	6	NA	NA	NA
CNS-GBM	c065761d-f775-457f-bda0-4c7c257a701e	4	139003871	139005066	7	1195	NA	5	2	NA	NA	NA
CNS-GBM	c065761d-f775-457f-bda0-4c7c257a701e	13	37720849	37730547	13	9698	1	5	5	NA	1	1
CNS-GBM	c065761d-f775-457f-bda0-4c7c257a701e	13	81307869	81309779	7	1910	1	3	3	NA	NA	NA
CNS-GBM	c065761d-f775-457f-bda0-4c7c257a701e	13	91408493	91411509	7	3016	1	1	5	NA	NA	NA
Lymph-CLL	c13d7ee5-2bf0-43e4-9cd3-9326ed9355cf	2	89159470	89165491	32	6021	NA	8	10	10	NA	4
Lymph-CLL	c13d7ee5-2bf0-43e4-9cd3-9326ed9355cf	14	106324978	106330413	49	5435	2	8	14	15	6	4
Lymph-CLL	c13d7ee5-2bf0-43e4-9cd3-9326ed9355cf	19	38133349	38134102	8	753	NA	1	NA	6	NA	1
Panc-AdenoCA	c13fb736-614c-4d5f-83bf-2d7586f4fb53	2	115302759	115306169	20	3410	NA	5	15	NA	NA	NA
Panc-AdenoCA	c13fb736-614c-4d5f-83bf-2d7586f4fb53	6	105936682	105937793	9	1111	NA	5	4	NA	NA	NA
Panc-AdenoCA	c13fb736-614c-4d5f-83bf-2d7586f4fb53	6	152259258	152263926	63	4668	16	19	28	NA	NA	NA
Panc-AdenoCA	c13fb736-614c-4d5f-83bf-2d7586f4fb53	9	21302510	21302574	6	64	1	NA	5	NA	NA	NA
Panc-AdenoCA	c13fb736-614c-4d5f-83bf-2d7586f4fb53	11	29337320	29338239	13	919	2	3	8	NA	NA	NA
Panc-AdenoCA	c13fb736-614c-4d5f-83bf-2d7586f4fb53	12	125051135	125053198	9	2063	NA	NA	9	NA	NA	NA
Panc-AdenoCA	c13fb736-614c-4d5f-83bf-2d7586f4fb53	18	48552898	48553481	6	583	1	1	4	NA	NA	NA
Panc-AdenoCA	c13fb736-614c-4d5f-83bf-2d7586f4fb53	22	41292851	41293289	8	438	NA	5	3	NA	NA	NA
Panc-AdenoCA	c13fb736-614c-4d5f-83bf-2d7586f4fb53	X	14240746	14241141	6	395	2	1	3	NA	NA	NA
Panc-AdenoCA	c15a51cc-4fdc-4343-a410-13ddc238dac8	18	18646424	18650124	6	3700	4	NA	2	NA	NA	NA
Panc-AdenoCA	c15a51cc-4fdc-4343-a410-13ddc238dac8	18	39017012	39023877	34	6865	6	14	14	NA	NA	NA
Panc-AdenoCA	c15a51cc-4fdc-4343-a410-13ddc238dac8	18	73853841	73855017	11	1176	2	2	6	NA	NA	1
Panc-AdenoCA	c15a51cc-4fdc-4343-a410-13ddc238dac8	18	76082951	76128002	63	45051	6	26	30	NA	1	NA
Stomach-AdenoCA	c1b44966-0f72-4c4f-8783-ab3ffe7f17b2	1	246417294	246417935	9	641	2	6	1	NA	NA	NA
Stomach-AdenoCA	c1b44966-0f72-4c4f-8783-ab3ffe7f17b2	3	55163890	55168471	30	4581	3	16	9	1	NA	1
Stomach-AdenoCA	c1b44966-0f72-4c4f-8783-ab3ffe7f17b2	12	69823869	69824012	6	143	NA	2	4	NA	NA	NA
Stomach-AdenoCA	c1b44966-0f72-4c4f-8783-ab3ffe7f17b2	12	69908904	69914756	7	5852	NA	1	5	NA	1	NA
Liver-HCC	c1d9ed06-7498-4c6c-a0de-dbf28e868109	2	41195720	41196345	8	625	NA	NA	8	NA	NA	NA
Liver-HCC	c1d9ed06-7498-4c6c-a0de-dbf28e868109	18	20840757	20841054	6	297	NA	NA	1	1	4	NA
Head-SCC	c2124bb2-30e1-4093-9a8a-3a188a6dc0a9	10	42948770	42953429	7	4659	1	2	4	NA	NA	NA
Head-SCC	c2124bb2-30e1-4093-9a8a-3a188a6dc0a9	10	53176610	53177552	16	942	NA	6	10	NA	NA	NA

Head-SCC	c2124bb2-30e1-4093-9a8a-3a188a6dc0a9	11	70342675	70344454	9	1779	2	2	5	NA	NA	NA
Head-SCC	c2124bb2-30e1-4093-9a8a-3a188a6dc0a9	12	38713025	38715339	6	2314	NA	3	3	NA	NA	NA
Head-SCC	c2124bb2-30e1-4093-9a8a-3a188a6dc0a9	12	45645067	45656941	26	11874	NA	11	15	NA	NA	NA
CNS-GBM	c27290e4-6835-448a-abdc-df8ddd5f4630	1	198130207	198134489	6	4282	NA	NA	1	2	2	1
CNS-GBM	c27290e4-6835-448a-abdc-df8ddd5f4630	7	50465120	50468575	6	3455	1	1	4	NA	NA	NA
CNS-GBM	c27290e4-6835-448a-abdc-df8ddd5f4630	7	80531022	80532602	10	1580	2	5	3	NA	NA	NA
Kidney-RCC	c2aafb9c-230a-43df-9a43-b34aa145a7bd	3	120797276	120798925	6	1649	3	2	1	NA	NA	NA
Panc-AdenoCA	c3679262-63d7-463e-9501-6ab3f177aee8	1	46139441	46140103	7	662	NA	5	2	NA	NA	NA
Panc-AdenoCA	c3679262-63d7-463e-9501-6ab3f177aee8	4	132465235	132465960	9	725	1	4	4	NA	NA	NA
Panc-AdenoCA	c3679262-63d7-463e-9501-6ab3f177aee8	8	125506406	125507257	12	851	5	3	4	NA	NA	NA
Panc-AdenoCA	c3679262-63d7-463e-9501-6ab3f177aee8	8	130720538	130731530	15	10992	2	6	7	NA	NA	NA
Lung-AdenoCA	c437535b-2acc-4236-beae-94e2c1336830	1	37302464	37305863	13	3399	1	6	6	NA	NA	NA
Lung-AdenoCA	c437535b-2acc-4236-beae-94e2c1336830	2	222218135	222225163	11	7028	1	2	8	NA	NA	NA
Lung-AdenoCA	c437535b-2acc-4236-beae-94e2c1336830	4	181833079	181836861	6	3782	1	4	1	NA	NA	NA
Lung-AdenoCA	c437535b-2acc-4236-beae-94e2c1336830	7	129071105	129072176	19	1071	2	11	6	NA	NA	NA
Lung-AdenoCA	c437535b-2acc-4236-beae-94e2c1336830	7	153459593	153462285	11	2692	NA	NA	4	3	3	1
Lung-AdenoCA	c437535b-2acc-4236-beae-94e2c1336830	8	33510024	33511773	7	1749	2	3	2	NA	NA	NA
Lung-AdenoCA	c437535b-2acc-4236-beae-94e2c1336830	11	57986786	57989929	6	3143	1	1	4	NA	NA	NA
Lung-AdenoCA	c437535b-2acc-4236-beae-94e2c1336830	12	71478526	71478669	6	143	NA	NA	6	NA	NA	NA
Lung-AdenoCA	c437535b-2acc-4236-beae-94e2c1336830	14	72614197	72618492	8	4295	1	4	3	NA	NA	NA
Lung-AdenoCA	c437535b-2acc-4236-beae-94e2c1336830	18	1309248	1312289	7	3041	NA	4	1	1	1	NA
Lung-AdenoCA	c437535b-2acc-4236-beae-94e2c1336830	19	29261816	29262261	6	445	2	1	3	NA	NA	NA
Lung-AdenoCA	c437535b-2acc-4236-beae-94e2c1336830	19	29614890	29615851	8	961	NA	7	1	NA	NA	NA
Bone-Leiomyo	c556f81b-8a6c-4bbb-876f-2e2ce570c185	1	156995591	156996408	6	817	3	1	2	NA	NA	NA
Bone-Leiomyo	c556f81b-8a6c-4bbb-876f-2e2ce570c185	1	164598648	164604465	9	5817	NA	NA	9	NA	NA	NA
Bone-Leiomyo	c556f81b-8a6c-4bbb-876f-2e2ce570c185	1	195844800	195849020	11	4220	2	5	4	NA	NA	NA
Bone-Leiomyo	c556f81b-8a6c-4bbb-876f-2e2ce570c185	12	62153537	62154978	7	1441	NA	NA	7	NA	NA	NA
Bone-Leiomyo	c556f81b-8a6c-4bbb-876f-2e2ce570c185	12	65723981	65728088	10	4107	NA	7	3	NA	NA	NA
Bone-Leiomyo	c556f81b-8a6c-4bbb-876f-2e2ce570c185	12	66306049	66306816	13	767	3	6	4	NA	NA	NA
Bone-Leiomyo	c556f81b-8a6c-4bbb-876f-2e2ce570c185	12	66666448	66667015	7	567	1	2	4	NA	NA	NA
Bone-Leiomyo	c556f81b-8a6c-4bbb-876f-2e2ce570c185	12	73550601	73552462	7	1861	1	3	3	NA	NA	NA
Bone-Leiomyo	c556f81b-8a6c-4bbb-876f-2e2ce570c185	21	43275772	43277501	8	1729	1	2	5	NA	NA	NA
Stomach-AdenoCA	c612570a-881d-421f-98b9-0e4563133744	4	146415510	146418351	13	2841	3	6	4	NA	NA	NA
Lymph-BNHL	c66926a8-474d-482c-bfb7-043029c8b737	2	89157513	89165241	28	7728	4	3	14	3	NA	4
Lymph-BNHL	c66926a8-474d-482c-bfb7-043029c8b737	5	85163388	85165284	8	1896	NA	NA	NA	3	4	1
Lymph-BNHL	c66926a8-474d-482c-bfb7-043029c8b737	9	112811640	112815174	6	3534	1	2	3	NA	NA	NA
Lymph-BNHL	c66926a8-474d-482c-bfb7-043029c8b737	14	106066364	106071897	14	5533	2	3	9	NA	NA	NA
Lymph-BNHL	c66926a8-474d-482c-bfb7-043029c8b737	14	106326703	106329236	23	2533	1	2	13	4	2	1
Lymph-BNHL	c66926a8-474d-482c-bfb7-043029c8b737	14	106815753	106816486	7	733	1	2	2	1	NA	1
Lymph-BNHL	c66926a8-474d-482c-bfb7-043029c8b737	16	10972311	10974883	19	2572	7	7	5	NA	NA	NA
Breast-AdenoCa	c67409b2-ac25-42a0-8543-4636ef132fe4	6	96363827	96368017	8	4190	NA	8	NA	NA	NA	NA
Breast-AdenoCa	c67409b2-ac25-42a0-8543-4636ef132fe4	8	41038734	41044850	8	6116	1	5	2	NA	NA	NA
Prost-AdenoCA	c6a28615-1b92-4085-956e-eeedb2573c03	5	128806040	128810352	6	4312	NA	3	3	NA	NA	NA
Prost-AdenoCA	c6a28615-1b92-4085-956e-eeedb2573c03	5	128813103	128819440	11	6337	1	6	4	NA	NA	NA
Panc-AdenoCA	c741fb12-8160-43a7-bc70-2c545ea0031d	2	185702265	185702810	7	545	2	3	2	NA	NA	NA
Panc-AdenoCA	c741fb12-8160-43a7-bc70-2c545ea0031d	18	8447033	8447291	9	258	2	4	3	NA	NA	NA
Panc-AdenoCA	c741fb12-8160-43a7-bc70-2c545ea0031d	18	19404183	19404454	8	271	NA	3	5	NA	NA	NA
Panc-AdenoCA	c741fb12-8160-43a7-bc70-2c545ea0031d	18	23060055	23060451	7	396	1	1	5	NA	NA	NA
Panc-AdenoCA	c741fb12-8160-43a7-bc70-2c545ea0031d	X	49060561	49064321	14	3760	5	8	1	NA	NA	NA
Panc-AdenoCA	c741fb12-8160-43a7-bc70-2c545ea0031d	X	52096267	52097498	15	1231	3	5	7	NA	NA	NA
Panc-AdenoCA	c741fb12-8160-43a7-bc70-2c545ea0031d	X	86951229	86952833	22	1604	2	9	11	NA	NA	NA
Panc-AdenoCA	c741fb12-8160-43a7-bc70-2c545ea0031d	X	87065861	87066693	8	832	NA	4	4	NA	NA	NA
Panc-AdenoCA	c741fb12-8160-43a7-bc70-2c545ea0031d	X	87271327	87272420	18	1093	2	7	8	1	NA	NA
Panc-AdenoCA	c741fb12-8160-43a7-bc70-2c545ea0031d	X	87314050	87316227	23	2177	5	7	11	NA	NA	NA
Uterus-AdenoCA	c75cc75a-7496-420f-b526-ea63c77e9839	19	57605973	57611150	10	5177	1	6	3	NA	NA	NA
Ovary-AdenoCA	c767254e-b289-4904-a80f-050cf01ff8ba	2	103032646	103036798	11	4152	1	8	2	NA	NA	NA
Ovary-AdenoCA	c767254e-b289-4904-a80f-050cf01ff8ba	7	119460457	119462539	6	2082	2	NA	1	NA	2	1
Head-SCC	c76f7fcc-2748-4b6d-805e-d21652dd5e3a	2	172976016	172977031	9	1015	1	2	5	NA	NA	1
Head-SCC	c76f7fcc-2748-4b6d-805e-d21652dd5e3a	7	55053607	55054275	7	668	3	2	2	NA	NA	NA
Head-SCC	c76f7fcc-2748-4b6d-805e-d21652dd5e3a	7	55218998	55219865	8	867	1	3	4	NA	NA	NA

Head-SCC	c76f7fcc-2748-4b6d-805e-d21652dd5e3a	11	28441346	28443964	14	2618	NA	9	4	NA	NA	1
Head-SCC	c76f7fcc-2748-4b6d-805e-d21652dd5e3a	15	87161539	87162027	10	488	3	3	4	NA	NA	NA
Ovary-AdenoCA	c9959f68-c385-4c1f-9188-8203844d288e	2	119235455	119248334	25	12879	3	7	15	NA	NA	NA
Ovary-AdenoCA	c9959f68-c385-4c1f-9188-8203844d288e	21	46788946	46794133	9	5187	NA	5	4	NA	NA	NA
Panc-AdenoCA	c9e7c629-7b57-4ede-b315-0cea8c97c48e	2	9288153	9290968	14	2815	NA	7	7	NA	NA	NA
Panc-AdenoCA	c9e7c629-7b57-4ede-b315-0cea8c97c48e	2	9491207	9491996	10	789	NA	6	4	NA	NA	NA
Panc-AdenoCA	c9e7c629-7b57-4ede-b315-0cea8c97c48e	3	167852968	167858393	21	5425	1	6	14	NA	NA	NA
Panc-AdenoCA	c9e7c629-7b57-4ede-b315-0cea8c97c48e	21	27815396	27818283	13	2887	2	4	7	NA	NA	NA
Skin-Melanoma	ca004926-f3ac-4824-8ed5-d84e62efdfae	2	86255084	86257767	19	2683	2	8	9	NA	NA	NA
Skin-Melanoma	ca004926-f3ac-4824-8ed5-d84e62efdfae	2	175192321	175196099	12	3778	1	6	5	NA	NA	NA
Skin-Melanoma	ca004926-f3ac-4824-8ed5-d84e62efdfae	3	131594499	131597328	13	2829	5	3	5	NA	NA	NA
Skin-Melanoma	ca004926-f3ac-4824-8ed5-d84e62efdfae	3	180741298	180741882	7	584	1	2	4	NA	NA	NA
Skin-Melanoma	ca004926-f3ac-4824-8ed5-d84e62efdfae	6	90039657	90054884	19	15227	2	12	5	NA	NA	NA
Skin-Melanoma	ca004926-f3ac-4824-8ed5-d84e62efdfae	7	128986	132018	6	3032	1	3	2	NA	NA	NA
Skin-Melanoma	ca004926-f3ac-4824-8ed5-d84e62efdfae	7	69739988	69742041	18	2053	2	9	6	NA	1	NA
Skin-Melanoma	ca004926-f3ac-4824-8ed5-d84e62efdfae	7	69934052	69934602	9	550	NA	8	1	NA	NA	NA
Skin-Melanoma	ca004926-f3ac-4824-8ed5-d84e62efdfae	7	70217318	70217934	7	616	1	4	2	NA	NA	NA
Skin-Melanoma	ca004926-f3ac-4824-8ed5-d84e62efdfae	7	87962925	87969605	14	6680	NA	7	7	NA	NA	NA
Skin-Melanoma	ca004926-f3ac-4824-8ed5-d84e62efdfae	9	31654323	31654649	8	326	2	4	2	NA	NA	NA
Skin-Melanoma	ca004926-f3ac-4824-8ed5-d84e62efdfae	10	61723474	61725674	9	2200	NA	7	2	NA	NA	NA
Skin-Melanoma	ca004926-f3ac-4824-8ed5-d84e62efdfae	11	60674306	60686872	21	12566	1	8	12	NA	NA	NA
Skin-Melanoma	ca004926-f3ac-4824-8ed5-d84e62efdfae	11	68565639	68566988	10	1349	2	1	7	NA	NA	NA
Skin-Melanoma	ca004926-f3ac-4824-8ed5-d84e62efdfae	11	71131417	71135711	8	4294	1	1	6	NA	NA	NA
Skin-Melanoma	ca004926-f3ac-4824-8ed5-d84e62efdfae	11	71181953	71182211	7	258	1	2	4	NA	NA	NA
Skin-Melanoma	ca004926-f3ac-4824-8ed5-d84e62efdfae	11	71265725	71269336	10	3611	NA	NA	10	NA	NA	NA
Skin-Melanoma	ca004926-f3ac-4824-8ed5-d84e62efdfae	11	76186682	76187378	7	696	NA	NA	7	NA	NA	NA
Skin-Melanoma	ca004926-f3ac-4824-8ed5-d84e62efdfae	11	76407395	76411643	8	4248	NA	1	7	NA	NA	NA
Skin-Melanoma	ca004926-f3ac-4824-8ed5-d84e62efdfae	12	50801143	50805262	8	4119	1	3	3	NA	NA	1
Skin-Melanoma	ca004926-f3ac-4824-8ed5-d84e62efdfae	12	67112682	67116322	12	3640	NA	NA	12	NA	NA	NA
Skin-Melanoma	ca004926-f3ac-4824-8ed5-d84e62efdfae	12	77853722	77857180	8	3458	1	4	3	NA	NA	NA
Skin-Melanoma	ca004926-f3ac-4824-8ed5-d84e62efdfae	12	78347277	78349351	10	2074	NA	NA	10	NA	NA	NA
Skin-Melanoma	ca004926-f3ac-4824-8ed5-d84e62efdfae	12	89614125	89622391	16	8266	1	7	7	NA	NA	1
Skin-Melanoma	ca004926-f3ac-4824-8ed5-d84e62efdfae	13	77492229	77495373	8	3144	NA	1	7	NA	NA	NA
Skin-Melanoma	ca004926-f3ac-4824-8ed5-d84e62efdfae	17	59477512	59477921	6	409	2	2	2	NA	NA	NA
Skin-Melanoma	ca004926-f3ac-4824-8ed5-d84e62efdfae	17	59495338	59504714	26	9376	3	11	12	NA	NA	NA
Skin-Melanoma	ca004926-f3ac-4824-8ed5-d84e62efdfae	21	14613651	14616455	10	2804	NA	5	5	NA	NA	NA
Skin-Melanoma	ca004926-f3ac-4824-8ed5-d84e62efdfae	X	95247292	95252786	11	5494	1	4	6	NA	NA	NA
Skin-Melanoma	ca004926-f3ac-4824-8ed5-d84e62efdfae	X	124110285	124112330	8	2045	NA	2	6	NA	NA	NA
Panc-AdenoCA	ca284012-bd06-47a3-8a9d-c6b185d87d85	6	52390387	52391335	7	948	1	1	5	NA	NA	NA
Panc-AdenoCA	ca284012-bd06-47a3-8a9d-c6b185d87d85	6	120365372	120366297	10	925	1	4	5	NA	NA	NA
Panc-AdenoCA	cafe9603-a804-48fb-9217-e2d1c3c346c5	1	194278806	194281057	6	2251	3	NA	3	NA	NA	NA
Panc-AdenoCA	cafe9603-a804-48fb-9217-e2d1c3c346c5	5	63952385	63953743	6	1358	1	NA	5	NA	NA	NA
Panc-AdenoCA	cafe9603-a804-48fb-9217-e2d1c3c346c5	5	81218700	81220543	8	1843	NA	NA	8	NA	NA	NA
Panc-AdenoCA	cafe9603-a804-48fb-9217-e2d1c3c346c5	8	39409370	39411084	10	1714	1	NA	9	NA	NA	NA
Panc-AdenoCA	cafe9603-a804-48fb-9217-e2d1c3c346c5	8	133174116	133178410	11	4294	3	1	7	NA	NA	NA
Panc-AdenoCA	cafe9603-a804-48fb-9217-e2d1c3c346c5	9	10481857	10490307	13	8450	2	NA	11	NA	NA	NA
Panc-AdenoCA	cafe9603-a804-48fb-9217-e2d1c3c346c5	9	12590407	12593381	7	2974	1	NA	6	NA	NA	NA
Panc-AdenoCA	cafe9603-a804-48fb-9217-e2d1c3c346c5	13	22546263	22554219	11	7956	NA	NA	11	NA	NA	NA
Panc-AdenoCA	cafe9603-a804-48fb-9217-e2d1c3c346c5	13	38174856	38180702	7	5846	NA	NA	7	NA	NA	NA
Panc-AdenoCA	cafe9603-a804-48fb-9217-e2d1c3c346c5	13	39619585	39623569	9	3984	NA	NA	8	NA	1	NA
Panc-AdenoCA	cafe9603-a804-48fb-9217-e2d1c3c346c5	13	62546270	62554610	12	8340	NA	NA	12	NA	NA	NA
Panc-AdenoCA	cafe9603-a804-48fb-9217-e2d1c3c346c5	13	69509946	69515155	13	5209	NA	NA	13	NA	NA	NA
Panc-AdenoCA	cafe9603-a804-48fb-9217-e2d1c3c346c5	13	85115712	85119239	9	3527	NA	NA	9	NA	NA	NA
Panc-AdenoCA	cafe9603-a804-48fb-9217-e2d1c3c346c5	13	96239271	96240436	10	1165	3	2	5	NA	NA	NA
Panc-AdenoCA	cafe9603-a804-48fb-9217-e2d1c3c346c5	19	21564230	21565718	11	1488	NA	6	4	1	NA	NA
Panc-AdenoCA	cafe9603-a804-48fb-9217-e2d1c3c346c5	19	22039311	22040090	9	779	NA	1	8	NA	NA	NA
Panc-AdenoCA	cafe9603-a804-48fb-9217-e2d1c3c346c5	19	22548886	22549583	13	697	2	1	10	NA	NA	NA
Panc-AdenoCA	cafe9603-a804-48fb-9217-e2d1c3c346c5	22	47860444	47863247	9	2803	2	2	5	NA	NA	NA
Eso-AdenoCa	cb381d49-4546-400c-af02-f877083caa0a	5	58817363	58820249	15	2886	2	7	4	NA	1	1
Eso-AdenoCa	cb381d49-4546-400c-af02-f877083caa0a	16	68147646	68152692	17	5046	2	12	3	NA	NA	NA

Eso-AdenoCa	cb381d49-4546-400c-af02-f877083caa0a	17	46994260	46994745	7	485	4	1	2	NA	NA	NA
Panc-AdenoCA	cb4608a7-6aec-4cba-b20f-489e214c9dbd	2	225634947	225635236	8	289	NA	3	5	NA	NA	NA
Panc-AdenoCA	cb4608a7-6aec-4cba-b20f-489e214c9dbd	5	37408932	37411425	19	2493	1	10	8	NA	NA	NA
Panc-AdenoCA	cb4608a7-6aec-4cba-b20f-489e214c9dbd	9	37286703	37292945	28	6242	4	6	18	NA	NA	NA
Panc-AdenoCA	cb4608a7-6aec-4cba-b20f-489e214c9dbd	9	37368451	37369597	13	1146	NA	7	6	NA	NA	NA
Bone-Leiomyo	cb5e1546-cda6-4991-911c-f3dd9f1a475a	1	195030687	195048268	19	17581	1	10	7	NA	1	NA
Bone-Leiomyo	cb5e1546-cda6-4991-911c-f3dd9f1a475a	2	136577686	136588598	16	10912	6	3	5	NA	NA	2
Bone-Leiomyo	cb5e1546-cda6-4991-911c-f3dd9f1a475a	9	17953000	17954628	7	1628	1	4	2	NA	NA	NA
Bone-Leiomyo	cb5e1546-cda6-4991-911c-f3dd9f1a475a	9	21678222	21681200	12	2978	1	4	7	NA	NA	NA
Bone-Leiomyo	cb5e1546-cda6-4991-911c-f3dd9f1a475a	9	71669987	71676376	15	6389	4	5	6	NA	NA	NA
Bone-Leiomyo	cb5e1546-cda6-4991-911c-f3dd9f1a475a	9	71973853	71976388	10	2535	NA	8	2	NA	NA	NA
Bone-Leiomyo	cb5e1546-cda6-4991-911c-f3dd9f1a475a	9	72769245	72774789	9	5544	2	1	6	NA	NA	NA
Bone-Leiomyo	cb5e1546-cda6-4991-911c-f3dd9f1a475a	9	79435348	79438704	7	3356	3	1	3	NA	NA	NA
Bone-Leiomyo	cb5e1546-cda6-4991-911c-f3dd9f1a475a	9	81293870	81297676	10	3806	3	2	5	NA	NA	NA
Bone-Leiomyo	cb5e1546-cda6-4991-911c-f3dd9f1a475a	9	86245189	86247598	7	2409	NA	2	5	NA	NA	NA
Bone-Leiomyo	cb5e1546-cda6-4991-911c-f3dd9f1a475a	9	87739173	87740984	8	1811	3	NA	5	NA	NA	NA
Bone-Leiomyo	cb5e1546-cda6-4991-911c-f3dd9f1a475a	10	49478028	49478404	7	376	NA	4	2	1	NA	NA
Bone-Leiomyo	cb5e1546-cda6-4991-911c-f3dd9f1a475a	10	49922616	49925695	10	3079	3	NA	7	NA	NA	NA
Bone-Leiomyo	cb5e1546-cda6-4991-911c-f3dd9f1a475a	10	49951200	49959678	16	8478	1	1	13	NA	NA	1
Bone-Leiomyo	cb5e1546-cda6-4991-911c-f3dd9f1a475a	11	43819512	43822657	11	3145	1	4	5	1	NA	NA
Bone-Leiomyo	cb5e1546-cda6-4991-911c-f3dd9f1a475a	15	43043217	43045961	7	2744	1	2	4	NA	NA	NA
Bone-Leiomyo	cb5e1546-cda6-4991-911c-f3dd9f1a475a	17	63040061	63044765	21	4704	1	NA	20	NA	NA	NA
Bone-Leiomyo	cb5e1546-cda6-4991-911c-f3dd9f1a475a	20	8644783	8645093	6	310	1	2	3	NA	NA	NA
Bone-Leiomyo	cb5e1546-cda6-4991-911c-f3dd9f1a475a	20	8791512	8791874	20	362	NA	NA	20	NA	NA	NA
Bone-Leiomyo	cb5e1546-cda6-4991-911c-f3dd9f1a475a	20	14721674	14724477	9	2803	NA	2	7	NA	NA	NA
Eso-AdenoCa	cb753e6f-5ea3-4b58-9db3-7a62d15c8b2e	1	185593236	185593456	8	220	1	5	2	NA	NA	NA
Eso-AdenoCa	cb753e6f-5ea3-4b58-9db3-7a62d15c8b2e	7	82517649	82518186	10	537	NA	4	6	NA	NA	NA
Eso-AdenoCa	cb753e6f-5ea3-4b58-9db3-7a62d15c8b2e	7	87646693	87648270	11	1577	2	7	2	NA	NA	NA
Eso-AdenoCa	cb753e6f-5ea3-4b58-9db3-7a62d15c8b2e	18	20131421	20132805	11	1384	1	8	2	NA	NA	NA
Eso-AdenoCa	cb753e6f-5ea3-4b58-9db3-7a62d15c8b2e	20	44690169	44691347	9	1178	NA	4	5	NA	NA	NA
Eso-AdenoCa	cb753e6f-5ea3-4b58-9db3-7a62d15c8b2e	X	58100777	58105560	8	4783	1	3	3	1	NA	NA
Eso-AdenoCa	cb753e6f-5ea3-4b58-9db3-7a62d15c8b2e	X	124735975	124736389	7	414	1	NA	6	NA	NA	NA
Bone-Leiomyo	cb783f8b-d092-4104-b809-3f90b19df7df	1	205220366	205221029	8	663	1	1	6	NA	NA	NA
Bone-Leiomyo	cb783f8b-d092-4104-b809-3f90b19df7df	1	237768880	237771769	8	2889	2	4	2	NA	NA	NA
Bone-Leiomyo	cb783f8b-d092-4104-b809-3f90b19df7df	3	191900244	191903446	9	3202	NA	NA	2	2	4	1
Bone-Leiomyo	cb783f8b-d092-4104-b809-3f90b19df7df	9	72778591	72780638	11	2047	NA	3	8	NA	NA	NA
Bone-Leiomyo	cb783f8b-d092-4104-b809-3f90b19df7df	12	58429329	58433095	8	3766	1	3	4	NA	NA	NA
Bone-Leiomyo	cb783f8b-d092-4104-b809-3f90b19df7df	12	67220782	67224977	9	4195	NA	NA	9	NA	NA	NA
Bone-Leiomyo	cb783f8b-d092-4104-b809-3f90b19df7df	12	68838758	68842747	9	3989	1	NA	8	NA	NA	NA
Bone-Leiomyo	cb783f8b-d092-4104-b809-3f90b19df7df	12	69705514	69710973	7	5459	NA	3	4	NA	NA	NA
Bone-Leiomyo	cb783f8b-d092-4104-b809-3f90b19df7df	12	69785582	69791055	8	5473	1	1	2	1	2	1
Bone-Leiomyo	cb783f8b-d092-4104-b809-3f90b19df7df	12	70540528	70543375	7	2847	NA	3	4	NA	NA	NA
Bone-Leiomyo	cb783f8b-d092-4104-b809-3f90b19df7df	12	70575177	70575887	8	710	NA	4	4	NA	NA	NA
Bone-Leiomyo	cb783f8b-d092-4104-b809-3f90b19df7df	12	84028335	84032584	7	4249	1	5	1	NA	NA	NA
Bone-Leiomyo	cb783f8b-d092-4104-b809-3f90b19df7df	12	96439498	96450753	20	11255	2	10	8	NA	NA	NA
Bone-Leiomyo	cb783f8b-d092-4104-b809-3f90b19df7df	12	99158093	99165600	10	7507	2	1	7	NA	NA	NA
Bone-Leiomyo	cb783f8b-d092-4104-b809-3f90b19df7df	12	102768315	102771732	7	3417	1	4	2	NA	NA	NA
Bone-Leiomyo	cb783f8b-d092-4104-b809-3f90b19df7df	12	103475670	103476665	9	995	1	8	NA	NA	NA	NA
Bone-Leiomyo	cb783f8b-d092-4104-b809-3f90b19df7df	21	21250050	21251753	8	1703	2	3	3	NA	NA	NA
Kidney-RCC	cbb788dd-964b-4cfd-80e1-979c7b5b3c52	21	37029465	37032833	7	3368	NA	5	2	NA	NA	NA
Uterus-AdenoCA	cc19a480-9c66-4ee8-a0c0-921acac02689	1	31555925	31563919	10	7994	1	3	6	NA	NA	NA
Uterus-AdenoCA	cc19a480-9c66-4ee8-a0c0-921acac02689	3	127151706	127151862	6	156	1	NA	5	NA	NA	NA
Uterus-AdenoCA	cc19a480-9c66-4ee8-a0c0-921acac02689	8	64141640	64142117	8	477	1	4	3	NA	NA	NA
Uterus-AdenoCA	cc19a480-9c66-4ee8-a0c0-921acac02689	8	80665122	80665779	7	657	1	5	1	NA	NA	NA
Uterus-AdenoCA	cc19a480-9c66-4ee8-a0c0-921acac02689	10	78891067	78895553	6	4486	1	2	3	NA	NA	NA
Uterus-AdenoCA	cc19a480-9c66-4ee8-a0c0-921acac02689	18	26052400	26061162	35	8762	3	12	20	NA	NA	NA
Uterus-AdenoCA	cc19a480-9c66-4ee8-a0c0-921acac02689	19	13337462	13345331	12	7869	2	2	8	NA	NA	NA
Uterus-AdenoCA	cc19a480-9c66-4ee8-a0c0-921acac02689	19	29487825	29495891	21	8066	NA	8	13	NA	NA	NA
Breast-AdenoCa	cc4f9175-a8c8-424a-99d7-8bd11cae0e52	2	9826712	9831890	7	5178	NA	2	5	NA	NA	NA
Breast-AdenoCa	cc4f9175-a8c8-424a-99d7-8bd11cae0e52	3	160859397	160863445	6	4048	1	NA	5	NA	NA	NA

Breast-AdenoCa	cc4f9175-a8c8-424a-99d7-8bd11cae0e52	5	68419204	68423729	10	4525	1	4	5	NA	NA	NA
Breast-AdenoCa	cc4f9175-a8c8-424a-99d7-8bd11cae0e52	6	79100333	79106474	9	6141	2	6	1	NA	NA	NA
Breast-AdenoCa	cc4f9175-a8c8-424a-99d7-8bd11cae0e52	7	65894471	65896813	6	2342	2	2	2	NA	NA	NA
Breast-AdenoCa	cc4f9175-a8c8-424a-99d7-8bd11cae0e52	8	94265988	94268377	8	2389	1	3	3	1	NA	NA
Breast-AdenoCa	cc4f9175-a8c8-424a-99d7-8bd11cae0e52	8	96644318	96647661	7	3343	NA	4	3	NA	NA	NA
Breast-AdenoCa	cc4f9175-a8c8-424a-99d7-8bd11cae0e52	16	2582361	2587311	7	4950	1	2	4	NA	NA	NA
Breast-AdenoCa	cc4f9175-a8c8-424a-99d7-8bd11cae0e52	16	4451757	4456508	8	4751	1	1	6	NA	NA	NA
Breast-AdenoCa	cc4f9175-a8c8-424a-99d7-8bd11cae0e52	16	50833343	50833549	8	206	3	3	2	NA	NA	NA
Breast-AdenoCa	cc4f9175-a8c8-424a-99d7-8bd11cae0e52	18	68320366	68323115	7	2749	1	3	3	NA	NA	NA
Breast-AdenoCa	cc4f9175-a8c8-424a-99d7-8bd11cae0e52	19	13454045	13455780	8	1735	1	5	1	NA	1	NA
Liver-HCC	cc792058-c622-11e3-bf01-24c6515278c0	4	58740689	58745200	18	4511	2	10	6	NA	NA	NA
Liver-HCC	cc792058-c622-11e3-bf01-24c6515278c0	12	78750756	78751111	6	355	1	2	1	NA	2	NA
Panc-AdenoCA	cc7af465-22ed-485d-a78c-282b840bf7c9	2	14647694	14649259	8	1565	NA	5	3	NA	NA	NA
Panc-AdenoCA	cc7af465-22ed-485d-a78c-282b840bf7c9	17	32956050	32956856	14	806	NA	NA	14	NA	NA	NA
Panc-AdenoCA	cc7af465-22ed-485d-a78c-282b840bf7c9	17	33902576	33904556	33	1980	NA	NA	26	2	5	NA
Panc-AdenoCA	cc7af465-22ed-485d-a78c-282b840bf7c9	17	49531037	49531854	11	817	NA	4	7	NA	NA	NA
Panc-AdenoCA	cc7af465-22ed-485d-a78c-282b840bf7c9	17	52838716	52843097	9	4381	1	3	5	NA	NA	NA
Lymph-BNHL	cc7bba1d-8990-4557-a565-9cff1085f023	14	106324877	106330263	40	5386	2	5	13	10	3	7
Lymph-BNHL	cc7bba1d-8990-4557-a565-9cff1085f023	22	23039946	23040849	6	903	NA	1	3	2	NA	NA
Lung-AdenoCA	cd0aeed5-93a1-4287-8a88-fe6b7b5e3983	5	1603470	1606799	8	3329	NA	NA	1	3	3	1
Lung-AdenoCA	cd0aeed5-93a1-4287-8a88-fe6b7b5e3983	5	2558730	2560581	8	1851	NA	4	4	NA	NA	NA
Lung-AdenoCA	cd0aeed5-93a1-4287-8a88-fe6b7b5e3983	5	4210446	4211399	9	953	NA	2	7	NA	NA	NA
Lung-AdenoCA	cd0aeed5-93a1-4287-8a88-fe6b7b5e3983	5	18619744	18621651	22	1907	NA	1	21	NA	NA	NA
Lung-AdenoCA	cd0aeed5-93a1-4287-8a88-fe6b7b5e3983	5	19655787	19658381	10	2594	NA	3	7	NA	NA	NA
Lung-AdenoCA	cd0aeed5-93a1-4287-8a88-fe6b7b5e3983	5	25970956	25972932	7	1976	NA	2	4	NA	1	NA
Lung-AdenoCA	cd0aeed5-93a1-4287-8a88-fe6b7b5e3983	7	4504423	4505388	11	965	3	3	5	NA	NA	NA
Lung-AdenoCA	cd0aeed5-93a1-4287-8a88-fe6b7b5e3983	7	19611605	19638011	31	26406	NA	13	16	1	NA	1
Lung-AdenoCA	cd0aeed5-93a1-4287-8a88-fe6b7b5e3983	8	2824721	2827434	8	2713	NA	2	6	NA	NA	NA
Lung-AdenoCA	cd0aeed5-93a1-4287-8a88-fe6b7b5e3983	8	2891210	2896018	15	4808	NA	7	8	NA	NA	NA
Lung-AdenoCA	cd0aeed5-93a1-4287-8a88-fe6b7b5e3983	8	2958252	2958532	7	280	NA	3	4	NA	NA	NA
Lung-AdenoCA	cd0aeed5-93a1-4287-8a88-fe6b7b5e3983	9	90407783	90417722	25	9939	NA	6	17	NA	2	NA
Lung-AdenoCA	cd0aeed5-93a1-4287-8a88-fe6b7b5e3983	9	101217678	101218640	17	962	NA	8	9	NA	NA	NA
Lung-AdenoCA	cd0aeed5-93a1-4287-8a88-fe6b7b5e3983	9	129294708	129297109	8	2401	NA	NA	1	2	5	NA
Lung-AdenoCA	cd0aeed5-93a1-4287-8a88-fe6b7b5e3983	9	130041490	130047221	12	5731	NA	4	6	2	NA	NA
Lung-AdenoCA	cd0aeed5-93a1-4287-8a88-fe6b7b5e3983	12	64227020	64227361	6	341	NA	3	3	NA	NA	NA
Lung-AdenoCA	cd0aeed5-93a1-4287-8a88-fe6b7b5e3983	12	68292048	68292742	8	694	1	6	1	NA	NA	NA
Lung-AdenoCA	cd0aeed5-93a1-4287-8a88-fe6b7b5e3983	12	68731599	68731948	8	349	1	3	4	NA	NA	NA
Lung-AdenoCA	cd0aeed5-93a1-4287-8a88-fe6b7b5e3983	12	69624905	69628863	9	3958	NA	1	5	1	2	NA
Lung-AdenoCA	cd0aeed5-93a1-4287-8a88-fe6b7b5e3983	12	69639856	69642623	12	2767	NA	1	10	1	NA	NA
Lung-AdenoCA	cd0aeed5-93a1-4287-8a88-fe6b7b5e3983	12	69809193	69812070	8	2877	NA	3	2	3	NA	NA
Lung-AdenoCA	cd0aeed5-93a1-4287-8a88-fe6b7b5e3983	12	70125113	70129963	14	4850	NA	5	9	NA	NA	NA
Lung-AdenoCA	cd0aeed5-93a1-4287-8a88-fe6b7b5e3983	12	72630469	72632392	16	1923	NA	6	9	NA	NA	1
Lung-AdenoCA	cd0aeed5-93a1-4287-8a88-fe6b7b5e3983	12	77768826	77774616	37	5790	NA	11	21	1	3	1
Lung-AdenoCA	cd0aeed5-93a1-4287-8a88-fe6b7b5e3983	12	82494871	82502318	29	7447	NA	NA	29	NA	NA	NA
Lung-AdenoCA	cd0aeed5-93a1-4287-8a88-fe6b7b5e3983	15	24273594	24277001	7	3407	NA	1	2	1	3	NA
Lung-AdenoCA	cd0aeed5-93a1-4287-8a88-fe6b7b5e3983	19	19773643	19773847	8	204	1	1	5	NA	NA	1
Lung-AdenoCA	cd0aeed5-93a1-4287-8a88-fe6b7b5e3983	X	2477660	2479834	6	2174	NA	NA	6	NA	NA	NA
Lung-AdenoCA	cd0aeed5-93a1-4287-8a88-fe6b7b5e3983	X	22992369	22997320	7	4951	NA	2	2	1	2	NA
Lung-AdenoCA	cd0aeed5-93a1-4287-8a88-fe6b7b5e3983	X	28091993	28095279	30	3286	NA	2	28	NA	NA	NA
Lung-AdenoCA	cd0aeed5-93a1-4287-8a88-fe6b7b5e3983	X	31162553	31165255	15	2702	NA	NA	14	1	NA	NA
Lung-AdenoCA	cd0aeed5-93a1-4287-8a88-fe6b7b5e3983	X	35468685	35469914	8	1229	NA	NA	2	1	5	NA
Lung-AdenoCA	cd0aeed5-93a1-4287-8a88-fe6b7b5e3983	X	35894749	35899785	17	5036	NA	1	16	NA	NA	NA
ColoRect-AdenoCA	cd3d7559-b583-4474-81df-4bf9232de3c2	20	18545378	18549013	13	3635	5	3	5	NA	NA	NA
Ovary-AdenoCA	cd9efdef-a7fb-49e5-9515-63606ae8bbfc	1	106731111	106738028	9	6917	2	4	3	NA	NA	NA
Bone-Leiomyo	cdbbd701-9c05-4f9e-923d-06039dd8a04d	7	153317315	153323094	10	5779	1	NA	9	NA	NA	NA
Bone-Leiomyo	cdbbd701-9c05-4f9e-923d-06039dd8a04d	9	31844445	31845314	8	869	NA	4	4	NA	NA	NA
Bone-Leiomyo	cdbbd701-9c05-4f9e-923d-06039dd8a04d	X	19772412	19779033	11	6621	1	10	NA	NA	NA	NA
Eso-AdenoCa	ce799e7b-30e7-44a5-a185-3e50d5e059ef	19	14506966	14507270	9	304	3	NA	6	NA	NA	NA
Eso-AdenoCa	ce799e7b-30e7-44a5-a185-3e50d5e059ef	19	28502092	28502478	10	386	1	4	5	NA	NA	NA
Eso-AdenoCa	ce799e7b-30e7-44a5-a185-3e50d5e059ef	20	14728497	14729718	10	1221	3	NA	7	NA	NA	NA

Lymph-BNHL	ce85ccf3-6621-4976-b187-2f28d45dd4d9	2	89127134	89132087	9	4953	NA	NA	2	2	4	1
Lymph-BNHL	ce85ccf3-6621-4976-b187-2f28d45dd4d9	2	89157280	89160877	63	3597	3	10	7	16	11	16
Lymph-BNHL	ce85ccf3-6621-4976-b187-2f28d45dd4d9	2	96810052	96811012	8	960	NA	4	4	NA	NA	NA
Lymph-BNHL	ce85ccf3-6621-4976-b187-2f28d45dd4d9	2	111875520	111881634	24	6114	1	7	16	NA	NA	NA
Lymph-BNHL	ce85ccf3-6621-4976-b187-2f28d45dd4d9	3	187461901	187463132	7	1231	1	1	2	2	NA	1
Lymph-BNHL	ce85ccf3-6621-4976-b187-2f28d45dd4d9	6	102451448	102456223	6	4775	NA	1	1	3	NA	1
Lymph-BNHL	ce85ccf3-6621-4976-b187-2f28d45dd4d9	14	106067210	106071010	16	3800	2	6	8	NA	NA	NA
Lymph-BNHL	ce85ccf3-6621-4976-b187-2f28d45dd4d9	14	106326837	106376198	105	49361	3	28	31	16	12	15
Lymph-BNHL	ce85ccf3-6621-4976-b187-2f28d45dd4d9	14	107176263	107180608	13	4345	1	2	NA	5	3	2
Lymph-BNHL	ce85ccf3-6621-4976-b187-2f28d45dd4d9	16	10971483	10974349	8	2866	NA	4	4	NA	NA	NA
Lymph-BNHL	ce85ccf3-6621-4976-b187-2f28d45dd4d9	19	10339621	10345081	10	5460	1	2	7	NA	NA	NA
Lymph-BNHL	ce85ccf3-6621-4976-b187-2f28d45dd4d9	22	23223275	23231589	25	8314	NA	5	3	8	4	5
Panc-AdenoCA	cf072bfe-3191-4a71-9e0b-decaa28365e1	4	117154706	117173282	50	18576	2	28	20	NA	NA	NA
Panc-AdenoCA	cf072bfe-3191-4a71-9e0b-decaa28365e1	5	135871264	135871465	7	201	1	3	3	NA	NA	NA
Panc-AdenoCA	cf072bfe-3191-4a71-9e0b-decaa28365e1	11	94970318	94973162	13	2844	3	4	6	NA	NA	NA
Panc-AdenoCA	cf072bfe-3191-4a71-9e0b-decaa28365e1	14	82711979	82719680	20	7701	2	10	7	NA	NA	1
Liver-HCC	cf2d34c4-c622-11e3-bf01-24c6515278c0	1	79085999	79086068	8	69	NA	NA	NA	2	5	1
Liver-HCC	cf2d34c4-c622-11e3-bf01-24c6515278c0	3	23509906	23509960	7	54	NA	NA	NA	1	5	1
Liver-HCC	cf2d34c4-c622-11e3-bf01-24c6515278c0	3	43746387	43746430	7	43	NA	NA	NA	2	2	3
Liver-HCC	cf2d34c4-c622-11e3-bf01-24c6515278c0	6	56986524	56986753	7	229	NA	6	1	NA	NA	NA
Liver-HCC	cf2d34c4-c622-11e3-bf01-24c6515278c0	7	67906786	67906822	6	36	NA	NA	NA	NA	6	NA
Liver-HCC	cf2d34c4-c622-11e3-bf01-24c6515278c0	9	98664881	98665882	7	1001	NA	NA	1	1	5	NA
Liver-HCC	cf2d34c4-c622-11e3-bf01-24c6515278c0	12	24690208	24690250	7	42	NA	NA	NA	NA	5	2
Liver-HCC	cf2d34c4-c622-11e3-bf01-24c6515278c0	14	24895090	24895119	6	29	NA	NA	NA	2	3	1
Liver-HCC	cf2d34c4-c622-11e3-bf01-24c6515278c0	17	67482567	67482596	6	29	NA	NA	NA	2	3	1
Liver-HCC	cf2d34c4-c622-11e3-bf01-24c6515278c0	X	150517579	150518524	10	945	3	4	2	NA	1	NA
Panc-AdenoCA	cfe0bf13-2ff4-4164-9b6a-cd3615bf3ddc	6	93039619	93043234	7	3615	NA	NA	NA	3	4	NA
Liver-HCC	d02597f8-3ac7-4165-a65f-0e134e5d215b	3	100017285	100023413	9	6128	2	4	3	NA	NA	NA
Liver-HCC	d02597f8-3ac7-4165-a65f-0e134e5d215b	3	102271869	102276935	17	5066	2	11	4	NA	NA	NA
Liver-HCC	d02597f8-3ac7-4165-a65f-0e134e5d215b	11	67454960	67459338	12	4378	1	5	6	NA	NA	NA
Panc-AdenoCA	d05ea63c-86a3-463a-a790-2edaa74b4da7	3	81961808	81963559	9	1751	NA	5	4	NA	NA	NA
Panc-AdenoCA	d05ea63c-86a3-463a-a790-2edaa74b4da7	3	166453949	166455497	9	1548	3	5	1	NA	NA	NA
Panc-AdenoCA	d05ea63c-86a3-463a-a790-2edaa74b4da7	3	176356883	176358747	10	1864	2	3	5	NA	NA	NA
Panc-AdenoCA	d05ea63c-86a3-463a-a790-2edaa74b4da7	3	189819277	189821473	13	2196	3	5	5	NA	NA	NA
Panc-AdenoCA	d05ea63c-86a3-463a-a790-2edaa74b4da7	3	190678875	190680161	8	1286	4	2	2	NA	NA	NA
Panc-AdenoCA	d05ea63c-86a3-463a-a790-2edaa74b4da7	3	192338464	192338824	8	360	2	NA	6	NA	NA	NA
Panc-AdenoCA	d05ea63c-86a3-463a-a790-2edaa74b4da7	4	190845596	190846460	6	864	NA	4	2	NA	NA	NA
Panc-AdenoCA	d05ea63c-86a3-463a-a790-2edaa74b4da7	8	33789336	33790842	6	1506	NA	6	NA	NA	NA	NA
Panc-AdenoCA	d05ea63c-86a3-463a-a790-2edaa74b4da7	8	34617000	34618006	7	1006	1	1	5	NA	NA	NA
Panc-AdenoCA	d05ea63c-86a3-463a-a790-2edaa74b4da7	10	110106969	110108019	17	1050	4	5	8	NA	NA	NA
Panc-AdenoCA	d05ea63c-86a3-463a-a790-2edaa74b4da7	12	61590535	61590951	6	416	1	4	1	NA	NA	NA
Panc-AdenoCA	d05ea63c-86a3-463a-a790-2edaa74b4da7	17	14821736	14822375	6	639	1	4	1	NA	NA	NA
Panc-AdenoCA	d05ea63c-86a3-463a-a790-2edaa74b4da7	18	19258912	19262450	11	3538	NA	2	9	NA	NA	NA
Panc-AdenoCA	d05ea63c-86a3-463a-a790-2edaa74b4da7	X	33449746	33451908	8	2162	2	4	2	NA	NA	NA
ColoRect-AdenoCA	d0a0d845-32e2-4590-b909-29bd27b92900	20	36465161	36468009	14	2848	NA	7	7	NA	NA	NA
CNS-GBM	d1132127-1250-43af-9c16-425798a3d1a7	4	40890219	40891191	8	972	2	NA	1	1	2	2
CNS-GBM	d1132127-1250-43af-9c16-425798a3d1a7	12	86523042	86524353	9	1311	1	8	NA	NA	NA	NA
Panc-AdenoCA	d1804679-e728-4597-ac69-49554c087b9e	1	195669114	195672232	7	3118	1	3	3	NA	NA	NA
Panc-AdenoCA	d1804679-e728-4597-ac69-49554c087b9e	4	133903007	133903993	6	986	1	3	2	NA	NA	NA
Panc-AdenoCA	d1804679-e728-4597-ac69-49554c087b9e	6	78536785	78538093	10	1308	1	3	6	NA	NA	NA
Panc-AdenoCA	d1804679-e728-4597-ac69-49554c087b9e	7	94299378	94299891	6	513	NA	6	NA	NA	NA	NA
Panc-AdenoCA	d1804679-e728-4597-ac69-49554c087b9e	10	110509385	110511738	19	2353	NA	11	8	NA	NA	NA
Panc-AdenoCA	d1804679-e728-4597-ac69-49554c087b9e	14	79960772	79961640	9	868	2	2	5	NA	NA	NA
Panc-AdenoCA	d1804679-e728-4597-ac69-49554c087b9e	X	104331923	104332981	8	1058	NA	5	3	NA	NA	NA
Liver-HCC	d182b67c-c622-11e3-bf01-24c6515278c0	8	49546428	49553204	17	6776	3	8	6	NA	NA	NA
Liver-HCC	d252f328-4583-4e97-9a71-bb2885f06f73	8	69091877	69097750	9	5873	3	2	4	NA	NA	NA
Head-SCC	d25a4c65-9cb4-4611-909e-e68f93408d84	11	101423173	101437286	17	14113	2	4	10	NA	NA	1
Head-SCC	d25a4c65-9cb4-4611-909e-e68f93408d84	11	102059650	102061823	8	2173	NA	4	4	NA	NA	NA
Ovary-AdenoCA	d2e4dc23-992d-4e44-add4-2972718083cb	7	70148570	70152944	7	4374	NA	4	3	NA	NA	NA
Ovary-AdenoCA	d2e4dc23-992d-4e44-add4-2972718083cb	13	26268405	26268694	8	289	3	1	4	NA	NA	NA

Ovary-AdenoCA	d2e4dc23-992d-4e44-add4-2972718083cb	17	38529051	38531790	6	2739	1	NA	NA	NA	1	4
Ovary-AdenoCA	d2e4dc23-992d-4e44-add4-2972718083cb	18	26100206	26105030	7	4824	NA	5	2	NA	NA	NA
Lymph-BNHL	d31cce9d-1b38-44fd-9378-0d128e12a011	2	84637471	84637928	6	457	NA	NA	NA	5	1	NA
Lymph-BNHL	d31cce9d-1b38-44fd-9378-0d128e12a011	2	158780056	158785316	8	5260	NA	NA	1	6	1	NA
Lymph-BNHL	d31cce9d-1b38-44fd-9378-0d128e12a011	3	187460835	187463059	14	2224	2	1	6	3	NA	2
Lymph-BNHL	d31cce9d-1b38-44fd-9378-0d128e12a011	4	30860154	30866907	8	6753	NA	NA	1	5	NA	2
Lymph-BNHL	d31cce9d-1b38-44fd-9378-0d128e12a011	13	107266735	107268548	7	1813	NA	NA	1	5	NA	1
Lymph-BNHL	d31cce9d-1b38-44fd-9378-0d128e12a011	14	106238754	106241366	19	2612	4	4	9	2	NA	NA
Lymph-BNHL	d31cce9d-1b38-44fd-9378-0d128e12a011	14	106322040	106350602	148	28562	8	21	37	34	15	33
Lymph-BNHL	d31cce9d-1b38-44fd-9378-0d128e12a011	14	106829741	106830860	22	1119	1	3	5	4	4	5
Lymph-BNHL	d31cce9d-1b38-44fd-9378-0d128e12a011	22	22735488	22736088	7	600	NA	2	1	2	2	NA
Panc-AdenoCA	d333b55b-8bac-4a99-9d23-3cc0c25057bf	1	113520343	113520935	6	592	NA	NA	6	NA	NA	NA
Panc-AdenoCA	d333b55b-8bac-4a99-9d23-3cc0c25057bf	2	96963807	96965979	24	2172	5	8	11	NA	NA	NA
Panc-AdenoCA	d333b55b-8bac-4a99-9d23-3cc0c25057bf	8	117758611	117759176	7	565	NA	4	1	NA	1	1
Head-SCC	d3709eea-ca2a-49ac-b79c-871a5473d450	1	14797048	14798307	7	1259	NA	4	3	NA	NA	NA
Head-SCC	d3709eea-ca2a-49ac-b79c-871a5473d450	2	37441393	37444092	7	2699	NA	5	2	NA	NA	NA
Head-SCC	d3709eea-ca2a-49ac-b79c-871a5473d450	3	12666692	12671202	6	4510	NA	3	3	NA	NA	NA
Head-SCC	d3709eea-ca2a-49ac-b79c-871a5473d450	4	160240539	160244139	6	3600	2	NA	4	NA	NA	NA
Head-SCC	d3709eea-ca2a-49ac-b79c-871a5473d450	6	117383310	117384807	6	1497	NA	2	3	NA	1	NA
Head-SCC	d3709eea-ca2a-49ac-b79c-871a5473d450	7	92123604	92125429	6	1825	NA	6	NA	NA	NA	NA
Head-SCC	d3709eea-ca2a-49ac-b79c-871a5473d450	8	66894015	66897581	6	3566	NA	5	1	NA	NA	NA
Head-SCC	d3709eea-ca2a-49ac-b79c-871a5473d450	9	114819831	114821524	6	1693	NA	4	2	NA	NA	NA
Head-SCC	d3709eea-ca2a-49ac-b79c-871a5473d450	10	52634526	52638325	8	3799	NA	3	5	NA	NA	NA
Head-SCC	d3709eea-ca2a-49ac-b79c-871a5473d450	11	68715145	68716970	6	1825	NA	4	2	NA	NA	NA
Head-SCC	d3709eea-ca2a-49ac-b79c-871a5473d450	12	50507730	50512026	7	4296	NA	3	4	NA	NA	NA
Head-SCC	d3709eea-ca2a-49ac-b79c-871a5473d450	14	50597372	50598935	6	1563	NA	2	3	1	NA	NA
Head-SCC	d3709eea-ca2a-49ac-b79c-871a5473d450	17	6721271	6730357	14	9086	NA	11	3	NA	NA	NA
Head-SCC	d3709eea-ca2a-49ac-b79c-871a5473d450	17	7192917	7194167	7	1250	NA	3	4	NA	NA	NA
Head-SCC	d3709eea-ca2a-49ac-b79c-871a5473d450	19	10104041	10106133	6	2092	NA	5	1	NA	NA	NA
Head-SCC	d3709eea-ca2a-49ac-b79c-871a5473d450	21	17027722	17030720	6	2998	1	3	2	NA	NA	NA
Ovary-AdenoCA	d392ded3-afc8-4c79-b278-40245f18f2f8	19	14980305	14987167	9	6862	NA	2	7	NA	NA	NA
Ovary-AdenoCA	d392ded3-afc8-4c79-b278-40245f18f2f8	19	15012113	15013105	13	992	1	2	10	NA	NA	NA
Ovary-AdenoCA	d392ded3-afc8-4c79-b278-40245f18f2f8	19	23938498	23944207	21	5709	2	7	12	NA	NA	NA
Ovary-AdenoCA	d392ded3-afc8-4c79-b278-40245f18f2f8	19	34640266	34643945	9	3679	NA	1	8	NA	NA	NA
Ovary-AdenoCA	d392ded3-afc8-4c79-b278-40245f18f2f8	19	43871843	43873028	7	1185	1	1	5	NA	NA	NA
Ovary-AdenoCA	d392ded3-afc8-4c79-b278-40245f18f2f8	19	43910761	43915247	15	4486	NA	4	11	NA	NA	NA
Ovary-AdenoCA	d392ded3-afc8-4c79-b278-40245f18f2f8	19	43985069	43989054	7	3985	1	NA	6	NA	NA	NA
Ovary-AdenoCA	d392ded3-afc8-4c79-b278-40245f18f2f8	21	23643798	23646051	7	2253	2	NA	5	NA	NA	NA
Ovary-AdenoCA	d392ded3-afc8-4c79-b278-40245f18f2f8	21	33003089	33013682	23	10593	4	9	10	NA	NA	NA
Ovary-AdenoCA	d392ded3-afc8-4c79-b278-40245f18f2f8	21	43885751	43889057	8	3306	3	1	3	NA	NA	1
Ovary-AdenoCA	d392ded3-afc8-4c79-b278-40245f18f2f8	21	44346540	44348898	12	2358	2	4	5	NA	NA	1
Liver-HCC	d3ad5b5a-c622-11e3-bf01-24c6515278c0	X	68920373	68920800	6	427	NA	4	2	NA	NA	NA
ColoRect-AdenoCA	d3aff5d3-23c0-43ae-9c01-8ddd776b530b	20	10612336	10616709	6	4373	NA	3	3	NA	NA	NA
ColoRect-AdenoCA	d3aff5d3-23c0-43ae-9c01-8ddd776b530b	20	10618957	10625083	9	6126	3	5	NA	1	NA	NA
ColoRect-AdenoCA	d3aff5d3-23c0-43ae-9c01-8ddd776b530b	20	19329498	19331890	8	2392	1	5	2	NA	NA	NA
ColoRect-AdenoCA	d3aff5d3-23c0-43ae-9c01-8ddd776b530b	20	37462741	37467813	20	5072	4	12	3	NA	NA	1
ColoRect-AdenoCA	d3aff5d3-23c0-43ae-9c01-8ddd776b530b	20	48324683	48326232	8	1549	4	1	3	NA	NA	NA
ColoRect-AdenoCA	d3aff5d3-23c0-43ae-9c01-8ddd776b530b	20	51538689	51544680	9	5991	3	3	3	NA	NA	NA
ColoRect-AdenoCA	d3d65db3-36f9-41c7-8e5e-1683ce94dfcb	11	10179443	10184966	21	5523	2	12	5	1	NA	1
ColoRect-AdenoCA	d3d65db3-36f9-41c7-8e5e-1683ce94dfcb	11	13925635	13929780	27	4145	5	18	3	NA	1	NA
ColoRect-AdenoCA	d3d65db3-36f9-41c7-8e5e-1683ce94dfcb	11	36633776	36634260	8	484	NA	4	4	NA	NA	NA
ColoRect-AdenoCA	d3d65db3-36f9-41c7-8e5e-1683ce94dfcb	11	80024036	80031938	24	7902	4	5	15	NA	NA	NA
ColoRect-AdenoCA	d3d65db3-36f9-41c7-8e5e-1683ce94dfcb	17	7115002	7116011	9	1009	3	4	2	NA	NA	NA
ColoRect-AdenoCA	d3d65db3-36f9-41c7-8e5e-1683ce94dfcb	17	7141122	7142473	17	1351	3	10	4	NA	NA	NA
ColoRect-AdenoCA	d3d65db3-36f9-41c7-8e5e-1683ce94dfcb	17	27040695	27042600	7	1905	3	4	NA	NA	NA	NA
ColoRect-AdenoCA	d3d65db3-36f9-41c7-8e5e-1683ce94dfcb	21	18327755	18330640	25	2885	7	8	9	NA	NA	1
ColoRect-AdenoCA	d3d65db3-36f9-41c7-8e5e-1683ce94dfcb	21	35119914	35121021	8	1107	1	5	2	NA	NA	NA
Kidney-RCC	d429c27a-5963-42fe-b3a0-7252c20669d8	20	22155499	22163341	12	7842	NA	1	NA	5	NA	6
Skin-Melanoma	d432e99a-67fb-4609-b90f-99438eee7cae	1	118593338	118595444	9	2106	NA	4	5	NA	NA	NA
Skin-Melanoma	d432e99a-67fb-4609-b90f-99438eee7cae	17	52954966	52956940	7	1974	NA	NA	7	NA	NA	NA

Skin-Melanoma	d432e99a-67fb-4609-b90f-99438eee7cae	17	57012516	57012727	8	211	1	4	3	NA	NA	NA
Skin-Melanoma	d432e99a-67fb-4609-b90f-99438eee7cae	17	67905321	67907413	8	2092	1	2	5	NA	NA	NA
ColoRect-AdenoCA	d452293a-6148-4fcf-9fbc-446815016751	8	93905941	93906853	8	912	NA	4	4	NA	NA	NA
ColoRect-AdenoCA	d452293a-6148-4fcf-9fbc-446815016751	14	26097462	26099196	8	1734	4	3	1	NA	NA	NA
Bladder-TCC	d4615ca0-b5c7-4a5c-8593-bd50034a78ae	7	112419407	112422085	6	2678	1	4	1	NA	NA	NA
Bladder-TCC	d4615ca0-b5c7-4a5c-8593-bd50034a78ae	8	109026248	109026265	6	17	1	NA	1	3	1	NA
Panc-AdenoCA	d4907a1b-8b06-47c5-8bca-c781d9cddaf8	1	118725049	118725938	14	889	1	4	8	NA	NA	1
Panc-AdenoCA	d4907a1b-8b06-47c5-8bca-c781d9cddaf8	8	13849267	13849713	6	446	1	3	2	NA	NA	NA
Panc-AdenoCA	d4907a1b-8b06-47c5-8bca-c781d9cddaf8	8	37565972	37570669	12	4697	4	4	4	NA	NA	NA
Panc-AdenoCA	d4907a1b-8b06-47c5-8bca-c781d9cddaf8	12	133214553	133219196	6	4643	2	1	3	NA	NA	NA
Panc-AdenoCA	d4907a1b-8b06-47c5-8bca-c781d9cddaf8	22	17782041	17786616	18	4575	7	3	8	NA	NA	NA
Head-SCC	d4b80307-abbd-48bb-b52c-6dcd409699a2	2	45467400	45467986	10	586	2	1	7	NA	NA	NA
Ovary-AdenoCA	d4bf6034-aeae-48a6-907b-10e2cc904015	6	50752609	50754334	7	1725	2	2	3	NA	NA	NA
Ovary-AdenoCA	d4bf6034-aeae-48a6-907b-10e2cc904015	10	50793398	50793902	6	504	2	2	2	NA	NA	NA
Ovary-AdenoCA	d4bf6034-aeae-48a6-907b-10e2cc904015	10	66562518	66567567	7	5049	1	5	1	NA	NA	NA
Ovary-AdenoCA	d4bf6034-aeae-48a6-907b-10e2cc904015	20	17655859	17656979	7	1120	NA	2	5	NA	NA	NA
Kidney-RCC	d4c6061b-5019-4564-806d-4e75910a4690	3	84510947	84511465	7	518	1	5	1	NA	NA	NA
Eso-AdenoCa	d4cf11be-3bd7-403b-9722-91d8e8761e4d	6	32704754	32706496	11	1742	3	2	6	NA	NA	NA
Eso-AdenoCa	d4cf11be-3bd7-403b-9722-91d8e8761e4d	10	109514768	109519718	6	4950	1	NA	NA	2	1	2
Eso-AdenoCa	d4cf11be-3bd7-403b-9722-91d8e8761e4d	17	50395442	50395703	6	261	2	1	3	NA	NA	NA
Eso-AdenoCa	d4cf11be-3bd7-403b-9722-91d8e8761e4d	17	51157620	51159718	8	2098	1	3	4	NA	NA	NA
Eso-AdenoCa	d4cf11be-3bd7-403b-9722-91d8e8761e4d	18	15209808	15211226	6	1418	NA	1	5	NA	NA	NA
Eso-AdenoCa	d4cf11be-3bd7-403b-9722-91d8e8761e4d	X	55417228	55419340	6	2112	NA	3	3	NA	NA	NA
Breast-AdenoCa	d5372745-95cf-4572-a6e7-e7f5b796911a	1	94528272	94548410	23	20138	6	13	3	NA	1	NA
Breast-AdenoCa	d5372745-95cf-4572-a6e7-e7f5b796911a	1	119707129	119713262	16	6133	NA	7	9	NA	NA	NA
Breast-AdenoCa	d5372745-95cf-4572-a6e7-e7f5b796911a	2	80717656	80718810	6	1154	1	3	NA	NA	1	1
Breast-AdenoCa	d5372745-95cf-4572-a6e7-e7f5b796911a	3	133079005	133081351	10	2346	4	4	2	NA	NA	NA
Breast-AdenoCa	d5372745-95cf-4572-a6e7-e7f5b796911a	3	154164000	154169311	13	5311	1	6	6	NA	NA	NA
Breast-AdenoCa	d5372745-95cf-4572-a6e7-e7f5b796911a	4	41265150	41267977	6	2827	1	4	1	NA	NA	NA
Breast-AdenoCa	d5372745-95cf-4572-a6e7-e7f5b796911a	4	41416483	41417556	7	1073	6	1	NA	NA	NA	NA
Breast-AdenoCa	d5372745-95cf-4572-a6e7-e7f5b796911a	8	40695656	40696161	10	505	1	NA	9	NA	NA	NA
Breast-AdenoCa	d5372745-95cf-4572-a6e7-e7f5b796911a	8	61741025	61741753	12	728	2	3	7	NA	NA	NA
Breast-AdenoCa	d5372745-95cf-4572-a6e7-e7f5b796911a	10	67748657	67751138	6	2481	1	5	NA	NA	NA	NA
Breast-AdenoCa	d5372745-95cf-4572-a6e7-e7f5b796911a	11	67088354	67090711	7	2357	1	5	1	NA	NA	NA
Breast-AdenoCa	d5372745-95cf-4572-a6e7-e7f5b796911a	19	2366255	2366882	12	627	1	6	5	NA	NA	NA
Breast-AdenoCa	d5372745-95cf-4572-a6e7-e7f5b796911a	19	5654893	5659213	11	4320	NA	4	7	NA	NA	NA
Breast-AdenoCa	d5372745-95cf-4572-a6e7-e7f5b796911a	21	47522717	47527957	10	5240	1	9	NA	NA	NA	NA
Bone-Leiomyo	d53c2680-89ac-4f87-a7ba-f4c669546b76	6	168741255	168741885	6	630	NA	3	3	NA	NA	NA
Bone-Leiomyo	d53c2680-89ac-4f87-a7ba-f4c669546b76	10	379157	379806	8	649	NA	2	6	NA	NA	NA
Bone-Leiomyo	d53c2680-89ac-4f87-a7ba-f4c669546b76	10	395996	396383	7	387	NA	3	4	NA	NA	NA
Bone-Leiomyo	d53c2680-89ac-4f87-a7ba-f4c669546b76	10	25527113	25529185	8	2072	2	2	3	NA	1	NA
Bone-Leiomyo	d53c2680-89ac-4f87-a7ba-f4c669546b76	10	25669829	25671190	23	1361	1	7	15	NA	NA	NA
Bone-Leiomyo	d53c2680-89ac-4f87-a7ba-f4c669546b76	10	25843811	25845977	13	2166	1	8	4	NA	NA	NA
Bone-Leiomyo	d53c2680-89ac-4f87-a7ba-f4c669546b76	10	28918430	28919606	8	1176	2	4	2	NA	NA	NA
Bone-Leiomyo	d53c2680-89ac-4f87-a7ba-f4c669546b76	10	29539745	29542230	7	2485	NA	5	2	NA	NA	NA
Bone-Leiomyo	d53c2680-89ac-4f87-a7ba-f4c669546b76	10	37866001	37867150	7	1149	1	5	1	NA	NA	NA
Lung-SCC	d54ca81c-1bcc-41ea-b17a-9f7249ebe9c1	1	40995878	40998103	20	2225	NA	5	15	NA	NA	NA
Lung-SCC	d54ca81c-1bcc-41ea-b17a-9f7249ebe9c1	1	63433773	63434509	7	736	1	NA	6	NA	NA	NA
Lung-SCC	d54ca81c-1bcc-41ea-b17a-9f7249ebe9c1	2	153388930	153389238	8	308	1	1	6	NA	NA	NA
Lung-SCC	d54ca81c-1bcc-41ea-b17a-9f7249ebe9c1	3	175599692	175602282	20	2590	1	8	11	NA	NA	NA
Lung-SCC	d54ca81c-1bcc-41ea-b17a-9f7249ebe9c1	3	190181568	190182054	10	486	1	2	7	NA	NA	NA
Lung-SCC	d54ca81c-1bcc-41ea-b17a-9f7249ebe9c1	4	107852703	107854143	7	1440	NA	4	3	NA	NA	NA
Lung-SCC	d54ca81c-1bcc-41ea-b17a-9f7249ebe9c1	9	4909994	4911282	6	1288	2	2	2	NA	NA	NA
Lung-SCC	d54ca81c-1bcc-41ea-b17a-9f7249ebe9c1	X	73630015	73631238	8	1223	NA	4	4	NA	NA	NA
CNS-GBM	d60f54f5-b154-42c4-99fb-cea4e7a33dc7	1	209997351	210001493	10	4142	3	5	2	NA	NA	NA
CNS-GBM	d60f54f5-b154-42c4-99fb-cea4e7a33dc7	1	213044107	213044501	8	394	NA	NA	8	NA	NA	NA
CNS-GBM	d60f54f5-b154-42c4-99fb-cea4e7a33dc7	1	216903533	216908443	7	4910	NA	3	1	2	1	NA
CNS-GBM	d60f54f5-b154-42c4-99fb-cea4e7a33dc7	1	235668692	235688653	26	19961	4	10	12	NA	NA	NA
CNS-GBM	d60f54f5-b154-42c4-99fb-cea4e7a33dc7	1	235836765	235839625	10	2860	1	6	2	NA	1	NA
CNS-GBM	d60f54f5-b154-42c4-99fb-cea4e7a33dc7	1	240083436	240086385	8	2949	1	4	3	NA	NA	NA

CNS-GBM	d60f54f5-b154-42c4-99fb-cea4e7a33dc7	6	75382415	75386809	6	4394	2	2	2	NA	NA	NA
CNS-GBM	d60f54f5-b154-42c4-99fb-cea4e7a33dc7	9	16046158	16046370	8	212	NA	NA	8	NA	NA	NA
Liver-HCC	d60f880a-c622-11e3-bf01-24c6515278c0	14	34812966	34813723	7	757	NA	NA	7	NA	NA	NA
Stomach-AdenoCA	d6738c83-7b52-4224-846a-67bd085aaab8	3	924837	930054	7	5217	2	3	2	NA	NA	NA
Ovary-AdenoCA	d67cad13-e849-48b0-926c-10b6046ba0b9	1	6646809	6652980	18	6171	4	6	8	NA	NA	NA
Ovary-AdenoCA	d67cad13-e849-48b0-926c-10b6046ba0b9	1	12345109	12348095	16	2986	3	6	7	NA	NA	NA
Ovary-AdenoCA	d67cad13-e849-48b0-926c-10b6046ba0b9	2	63548384	63549332	10	948	2	4	3	NA	NA	1
Ovary-AdenoCA	d67cad13-e849-48b0-926c-10b6046ba0b9	2	177542418	177545464	10	3046	3	2	5	NA	NA	NA
Ovary-AdenoCA	d67cad13-e849-48b0-926c-10b6046ba0b9	2	238310573	238312770	10	2197	2	5	3	NA	NA	NA
Ovary-AdenoCA	d67cad13-e849-48b0-926c-10b6046ba0b9	5	39556420	39556969	12	549	3	2	7	NA	NA	NA
Ovary-AdenoCA	d67cad13-e849-48b0-926c-10b6046ba0b9	6	7028222	7028733	6	511	1	2	3	NA	NA	NA
Ovary-AdenoCA	d67cad13-e849-48b0-926c-10b6046ba0b9	12	24544160	24547490	6	3330	1	5	NA	NA	NA	NA
Lymph-BNHL	d6ee68e5-ceb3-4a63-b3bd-e64fe5398102	2	89157407	89165550	49	8143	2	4	6	16	10	11
Lymph-BNHL	d6ee68e5-ceb3-4a63-b3bd-e64fe5398102	2	89326738	89327032	8	294	NA	2	4	1	1	NA
Lymph-BNHL	d6ee68e5-ceb3-4a63-b3bd-e64fe5398102	14	106210933	106213373	13	2440	1	2	7	NA	2	1
Lymph-BNHL	d6ee68e5-ceb3-4a63-b3bd-e64fe5398102	14	106324398	106329187	52	4789	1	9	18	13	4	7
Lymph-BNHL	d6ee68e5-ceb3-4a63-b3bd-e64fe5398102	18	60985015	60988291	9	3276	NA	1	4	3	NA	1
Lymph-BNHL	d6ee68e5-ceb3-4a63-b3bd-e64fe5398102	22	23227613	23231640	12	4027	NA	2	6	3	NA	1
Eso-AdenoCa	d707940e-0f9d-4da3-9380-90a422e44efd	3	90410527	90411018	7	491	NA	3	1	2	1	NA
Lymph-BNHL	d733cf1a-4c42-4def-b6cb-5ef2c6a27fcd	1	70016088	70021303	7	5215	NA	NA	2	2	1	2
Lymph-BNHL	d733cf1a-4c42-4def-b6cb-5ef2c6a27fcd	1	195265034	195276856	14	11822	NA	1	2	4	2	5
Lymph-BNHL	d733cf1a-4c42-4def-b6cb-5ef2c6a27fcd	1	203274837	203275766	19	929	1	2	9	3	3	1
Lymph-BNHL	d733cf1a-4c42-4def-b6cb-5ef2c6a27fcd	2	136874316	136875714	8	1398	NA	1	7	NA	NA	NA
Lymph-BNHL	d733cf1a-4c42-4def-b6cb-5ef2c6a27fcd	2	139651834	139657022	8	5188	NA	NA	2	4	2	NA
Lymph-BNHL	d733cf1a-4c42-4def-b6cb-5ef2c6a27fcd	3	60777962	60782541	6	4579	1	NA	1	3	NA	1
Lymph-BNHL	d733cf1a-4c42-4def-b6cb-5ef2c6a27fcd	3	60784259	60790946	8	6687	1	NA	3	3	NA	1
Lymph-BNHL	d733cf1a-4c42-4def-b6cb-5ef2c6a27fcd	3	68011836	68016803	7	4967	1	NA	NA	3	NA	3
Lymph-BNHL	d733cf1a-4c42-4def-b6cb-5ef2c6a27fcd	3	110044714	110048363	9	3649	NA	NA	2	2	2	3
Lymph-BNHL	d733cf1a-4c42-4def-b6cb-5ef2c6a27fcd	3	144682728	144688346	9	5618	1	NA	1	4	1	2
Lymph-BNHL	d733cf1a-4c42-4def-b6cb-5ef2c6a27fcd	3	186711709	186716196	8	4487	NA	NA	4	3	NA	1
Lymph-BNHL	d733cf1a-4c42-4def-b6cb-5ef2c6a27fcd	3	186783994	186784212	7	218	1	NA	NA	1	2	3
Lymph-BNHL	d733cf1a-4c42-4def-b6cb-5ef2c6a27fcd	3	187458826	187476237	56	17411	1	7	15	14	11	8
Lymph-BNHL	d733cf1a-4c42-4def-b6cb-5ef2c6a27fcd	3	187660877	187665570	11	4693	NA	1	2	5	1	2
Lymph-BNHL	d733cf1a-4c42-4def-b6cb-5ef2c6a27fcd	3	187688046	187693859	8	5813	NA	1	3	3	1	NA
Lymph-BNHL	d733cf1a-4c42-4def-b6cb-5ef2c6a27fcd	3	188471432	188471873	7	441	NA	NA	5	NA	1	1
Lymph-BNHL	d733cf1a-4c42-4def-b6cb-5ef2c6a27fcd	4	37130	41259	7	4129	NA	NA	NA	6	1	NA
Lymph-BNHL	d733cf1a-4c42-4def-b6cb-5ef2c6a27fcd	4	25862561	25864134	9	1573	NA	NA	3	3	2	1
Lymph-BNHL	d733cf1a-4c42-4def-b6cb-5ef2c6a27fcd	4	40194761	40204378	28	9617	2	1	19	3	2	1
Lymph-BNHL	d733cf1a-4c42-4def-b6cb-5ef2c6a27fcd	4	64989623	64995303	12	5680	NA	NA	1	6	2	3
Lymph-BNHL	d733cf1a-4c42-4def-b6cb-5ef2c6a27fcd	4	121657783	121660806	7	3023	NA	1	NA	5	1	NA
Lymph-BNHL	d733cf1a-4c42-4def-b6cb-5ef2c6a27fcd	4	132560742	132561480	7	738	NA	1	NA	3	2	1
Lymph-BNHL	d733cf1a-4c42-4def-b6cb-5ef2c6a27fcd	4	161473631	161475971	8	2340	NA	NA	1	4	NA	3
Lymph-BNHL	d733cf1a-4c42-4def-b6cb-5ef2c6a27fcd	4	161489656	161490807	7	1151	NA	NA	4	NA	1	2
Lymph-BNHL	d733cf1a-4c42-4def-b6cb-5ef2c6a27fcd	4	165087940	165091696	9	3756	1	NA	1	4	NA	3
Lymph-BNHL	d733cf1a-4c42-4def-b6cb-5ef2c6a27fcd	4	171846586	171851143	7	4557	1	NA	1	3	1	1
Lymph-BNHL	d733cf1a-4c42-4def-b6cb-5ef2c6a27fcd	4	174258592	174261606	7	3014	NA	1	1	4	NA	1
Lymph-BNHL	d733cf1a-4c42-4def-b6cb-5ef2c6a27fcd	5	85207674	85209449	9	1775	NA	3	NA	2	1	3
Lymph-BNHL	d733cf1a-4c42-4def-b6cb-5ef2c6a27fcd	5	88324736	88342032	20	17296	NA	NA	6	4	2	8
Lymph-BNHL	d733cf1a-4c42-4def-b6cb-5ef2c6a27fcd	5	88360404	88364391	8	3987	NA	NA	3	2	1	2
Lymph-BNHL	d733cf1a-4c42-4def-b6cb-5ef2c6a27fcd	5	105091980	105093250	7	1270	1	NA	NA	3	NA	3
Lymph-BNHL	d733cf1a-4c42-4def-b6cb-5ef2c6a27fcd	5	109822805	109828814	9	6009	NA	NA	1	7	NA	1
Lymph-BNHL	d733cf1a-4c42-4def-b6cb-5ef2c6a27fcd	6	20981509	20984803	7	3294	NA	NA	2	4	NA	1
Lymph-BNHL	d733cf1a-4c42-4def-b6cb-5ef2c6a27fcd	6	37138937	37139756	9	819	1	1	5	2	NA	NA
Lymph-BNHL	d733cf1a-4c42-4def-b6cb-5ef2c6a27fcd	6	134491940	134496682	10	4742	NA	2	8	NA	NA	NA
Lymph-BNHL	d733cf1a-4c42-4def-b6cb-5ef2c6a27fcd	7	82915500	82920128	9	4628	1	NA	NA	3	2	3
Lymph-BNHL	d733cf1a-4c42-4def-b6cb-5ef2c6a27fcd	7	110612962	110617829	7	4867	NA	NA	1	3	NA	3
Lymph-BNHL	d733cf1a-4c42-4def-b6cb-5ef2c6a27fcd	7	110630395	110636675	8	6280	NA	NA	NA	1	4	3
Lymph-BNHL	d733cf1a-4c42-4def-b6cb-5ef2c6a27fcd	7	110652899	110667882	17	14983	1	1	2	8	2	3
Lymph-BNHL	d733cf1a-4c42-4def-b6cb-5ef2c6a27fcd	7	110670824	110801114	133	1E+05	10	2	15	61	12	33
Lymph-BNHL	d733cf1a-4c42-4def-b6cb-5ef2c6a27fcd	7	122751701	122754825	8	3124	1	1	1	3	1	1

Lymph-BNHL	d733cf1a-4c42-4def-b6cb-5ef2c6a27fcd	8	66296120	66299941	10	3821	2	NA	NA	4	3	1
Lymph-BNHL	d733cf1a-4c42-4def-b6cb-5ef2c6a27fcd	9	37024963	37033505	18	8542	NA	5	12	NA	1	NA
Lymph-BNHL	d733cf1a-4c42-4def-b6cb-5ef2c6a27fcd	9	73648712	73651617	7	2905	2	NA	1	NA	NA	4
Lymph-BNHL	d733cf1a-4c42-4def-b6cb-5ef2c6a27fcd	10	19516486	19518667	7	2181	NA	NA	1	3	2	1
Lymph-BNHL	d733cf1a-4c42-4def-b6cb-5ef2c6a27fcd	11	92742766	92745991	8	3225	NA	NA	2	3	NA	3
Lymph-BNHL	d733cf1a-4c42-4def-b6cb-5ef2c6a27fcd	11	128389554	128392005	7	2451	NA	NA	7	NA	NA	NA
Lymph-BNHL	d733cf1a-4c42-4def-b6cb-5ef2c6a27fcd	12	21244725	21248348	7	3623	NA	NA	1	1	3	2
Lymph-BNHL	d733cf1a-4c42-4def-b6cb-5ef2c6a27fcd	12	71403179	71408122	9	4943	NA	1	1	3	2	2
Lymph-BNHL	d733cf1a-4c42-4def-b6cb-5ef2c6a27fcd	12	73156772	73164099	9	7327	2	NA	4	2	NA	1
Lymph-BNHL	d733cf1a-4c42-4def-b6cb-5ef2c6a27fcd	12	73179987	73192021	14	12034	2	4	1	3	NA	4
Lymph-BNHL	d733cf1a-4c42-4def-b6cb-5ef2c6a27fcd	12	84553358	84557532	7	4174	NA	1	1	2	1	2
Lymph-BNHL	d733cf1a-4c42-4def-b6cb-5ef2c6a27fcd	12	113494167	113497201	29	3034	NA	3	15	6	4	1
Lymph-BNHL	d733cf1a-4c42-4def-b6cb-5ef2c6a27fcd	12	122457468	122464647	21	7179	1	5	9	2	4	NA
Lymph-BNHL	d733cf1a-4c42-4def-b6cb-5ef2c6a27fcd	13	46958261	46963142	12	4881	NA	NA	8	2	2	NA
Lymph-BNHL	d733cf1a-4c42-4def-b6cb-5ef2c6a27fcd	13	56404507	56409619	8	5112	NA	1	1	2	1	3
Lymph-BNHL	d733cf1a-4c42-4def-b6cb-5ef2c6a27fcd	13	87862111	87884681	36	22570	NA	1	9	10	8	8
Lymph-BNHL	d733cf1a-4c42-4def-b6cb-5ef2c6a27fcd	14	62164125	62165493	6	1368	1	NA	5	NA	NA	NA
Lymph-BNHL	d733cf1a-4c42-4def-b6cb-5ef2c6a27fcd	14	106110083	106112755	13	2672	NA	5	7	NA	NA	1
Lymph-BNHL	d733cf1a-4c42-4def-b6cb-5ef2c6a27fcd	14	106207570	106241387	71	33817	4	12	44	7	1	3
Lymph-BNHL	d733cf1a-4c42-4def-b6cb-5ef2c6a27fcd	14	106322630	106330797	121	8167	8	15	43	28	12	15
Lymph-BNHL	d733cf1a-4c42-4def-b6cb-5ef2c6a27fcd	14	106518847	106524394	45	5547	NA	2	8	14	7	14
Lymph-BNHL	d733cf1a-4c42-4def-b6cb-5ef2c6a27fcd	16	10971688	10973829	7	2141	NA	1	6	NA	NA	NA
Lymph-BNHL	d733cf1a-4c42-4def-b6cb-5ef2c6a27fcd	17	56409012	56409674	7	662	NA	NA	7	NA	NA	NA
Lymph-BNHL	d733cf1a-4c42-4def-b6cb-5ef2c6a27fcd	18	26608967	26616157	14	7190	NA	NA	1	2	5	6
Lymph-BNHL	d733cf1a-4c42-4def-b6cb-5ef2c6a27fcd	18	27536991	27543949	8	6958	NA	NA	1	3	1	3
Lymph-BNHL	d733cf1a-4c42-4def-b6cb-5ef2c6a27fcd	18	27548266	27568112	21	19846	NA	NA	6	3	3	9
Lymph-BNHL	d733cf1a-4c42-4def-b6cb-5ef2c6a27fcd	18	27570144	27576950	10	6806	1	NA	3	3	1	2
Lymph-BNHL	d733cf1a-4c42-4def-b6cb-5ef2c6a27fcd	21	43252191	43255384	6	3193	NA	NA	1	2	2	1
Lymph-BNHL	d733cf1a-4c42-4def-b6cb-5ef2c6a27fcd	22	22724089	22764325	79	40236	2	5	31	22	6	13
Lymph-BNHL	d733cf1a-4c42-4def-b6cb-5ef2c6a27fcd	22	23223214	23287287	95	64073	4	13	34	20	11	13
Lymph-BNHL	d733cf1a-4c42-4def-b6cb-5ef2c6a27fcd	X	112825880	112828446	9	2566	1	NA	NA	4	2	2
Lymph-BNHL	d733cf1a-4c42-4def-b6cb-5ef2c6a27fcd	X	138416366	138423475	10	7109	2	1	2	1	2	2
Ovary-AdenoCA	d8c2b4b2-e12b-43d2-bafc-87b29f027797	5	52298726	52309543	19	10817	NA	13	5	NA	NA	1
Ovary-AdenoCA	d8c2b4b2-e12b-43d2-bafc-87b29f027797	9	140863039	140864210	6	1171	4	2	NA	NA	NA	NA
Ovary-AdenoCA	d8c2b4b2-e12b-43d2-bafc-87b29f027797	20	31728142	31738585	12	10443	3	6	2	NA	NA	1
Ovary-AdenoCA	d8c2b4b2-e12b-43d2-bafc-87b29f027797	20	41923992	41924944	7	952	1	4	1	NA	NA	1
Breast-AdenoCa	d8c6d4b8-f279-4edc-aaa3-a1cc266aec4d	1	147407369	147408617	6	1248	NA	NA	6	NA	NA	NA
Breast-AdenoCa	d8c6d4b8-f279-4edc-aaa3-a1cc266aec4d	1	164192963	164193446	8	483	NA	6	2	NA	NA	NA
Breast-AdenoCa	d8c6d4b8-f279-4edc-aaa3-a1cc266aec4d	1	165091767	165092244	9	477	NA	NA	9	NA	NA	NA
Breast-AdenoCa	d8c6d4b8-f279-4edc-aaa3-a1cc266aec4d	2	50535431	50536066	16	635	NA	5	11	NA	NA	NA
Breast-AdenoCa	d8c6d4b8-f279-4edc-aaa3-a1cc266aec4d	3	108194229	108228076	41	33847	1	1	39	NA	NA	NA
Breast-AdenoCa	d8c6d4b8-f279-4edc-aaa3-a1cc266aec4d	4	33244968	33245804	8	836	3	4	NA	NA	1	NA
Breast-AdenoCa	d8c6d4b8-f279-4edc-aaa3-a1cc266aec4d	6	107379536	107380567	6	1031	NA	5	1	NA	NA	NA
Breast-AdenoCa	d8c6d4b8-f279-4edc-aaa3-a1cc266aec4d	10	60069632	60070143	8	511	NA	NA	8	NA	NA	NA
Breast-AdenoCa	d8c6d4b8-f279-4edc-aaa3-a1cc266aec4d	10	89437921	89438992	9	1071	NA	6	3	NA	NA	NA
Breast-AdenoCa	d8c6d4b8-f279-4edc-aaa3-a1cc266aec4d	11	10724416	10727012	7	2596	NA	NA	7	NA	NA	NA
Breast-AdenoCa	d8c6d4b8-f279-4edc-aaa3-a1cc266aec4d	11	51481069	51488288	21	7219	NA	11	9	1	NA	NA
Breast-AdenoCa	d8c6d4b8-f279-4edc-aaa3-a1cc266aec4d	11	72170914	72171252	7	338	1	3	3	NA	NA	NA
Breast-AdenoCa	d8c6d4b8-f279-4edc-aaa3-a1cc266aec4d	11	79969918	79970471	7	553	1	1	5	NA	NA	NA
Breast-AdenoCa	d8c6d4b8-f279-4edc-aaa3-a1cc266aec4d	11	84518408	84519567	15	1159	NA	11	4	NA	NA	NA
Breast-AdenoCa	d8c6d4b8-f279-4edc-aaa3-a1cc266aec4d	15	62247879	62254755	8	6876	1	1	6	NA	NA	NA
Breast-AdenoCa	d8c6d4b8-f279-4edc-aaa3-a1cc266aec4d	17	45734803	45735049	13	246	NA	NA	13	NA	NA	NA
Breast-AdenoCa	d8c6d4b8-f279-4edc-aaa3-a1cc266aec4d	18	20021557	20022702	13	1145	NA	8	4	NA	NA	1
Breast-AdenoCa	d8c6d4b8-f279-4edc-aaa3-a1cc266aec4d	19	16138100	16139193	11	1093	1	5	5	NA	NA	NA
Breast-AdenoCa	d8c6d4b8-f279-4edc-aaa3-a1cc266aec4d	19	21111686	21120241	15	8555	3	2	9	NA	NA	1
Breast-AdenoCa	d8c6d4b8-f279-4edc-aaa3-a1cc266aec4d	20	8163616	8173069	17	9453	1	12	4	NA	NA	NA
Breast-AdenoCa	d8c6d4b8-f279-4edc-aaa3-a1cc266aec4d	22	37994955	38019460	31	24505	6	3	21	NA	NA	1
Breast-AdenoCa	d8c6d4b8-f279-4edc-aaa3-a1cc266aec4d	X	83051583	83052094	7	511	1	3	3	NA	NA	NA
Stomach-AdenoCA	d8d5585d-32cd-4ac4-b410-a4122a17a558	15	95552021	95556799	6	4778	1	4	1	NA	NA	NA
Stomach-AdenoCA	d8d5585d-32cd-4ac4-b410-a4122a17a558	16	56671846	56671923	6	77	1	1	1	1	1	1

Bone-Leiomyo	d8f0becd-fda8-41f4-a424-e082f9eae22c	2	97165463	97178008	18	12545	8	5	5	NA	NA	NA
Bone-Leiomyo	d8f0becd-fda8-41f4-a424-e082f9eae22c	2	211790960	211794965	16	4005	2	4	9	NA	1	NA
Bone-Leiomyo	d8f0becd-fda8-41f4-a424-e082f9eae22c	6	15706755	15711238	12	4483	3	2	7	NA	NA	NA
Bone-Leiomyo	d8f0becd-fda8-41f4-a424-e082f9eae22c	6	37181612	37190606	16	8994	2	7	7	NA	NA	NA
Bone-Leiomyo	d8f0becd-fda8-41f4-a424-e082f9eae22c	6	57014919	57017129	19	2210	1	7	11	NA	NA	NA
Bone-Leiomyo	d8f0becd-fda8-41f4-a424-e082f9eae22c	7	26822667	26827095	7	4428	NA	2	5	NA	NA	NA
Bone-Leiomyo	d8f0becd-fda8-41f4-a424-e082f9eae22c	7	140697472	140700443	7	2971	NA	6	1	NA	NA	NA
Bone-Leiomyo	d8f0becd-fda8-41f4-a424-e082f9eae22c	10	37783344	37783484	9	140	NA	NA	9	NA	NA	NA
Bone-Leiomyo	d8f0becd-fda8-41f4-a424-e082f9eae22c	11	88895993	88900347	7	4354	NA	3	4	NA	NA	NA
Bone-Leiomyo	d8f0becd-fda8-41f4-a424-e082f9eae22c	11	105359303	105361175	7	1872	1	3	3	NA	NA	NA
Bone-Leiomyo	d8f0becd-fda8-41f4-a424-e082f9eae22c	15	76034451	76039910	10	5459	1	3	6	NA	NA	NA
Bone-Leiomyo	d8f0becd-fda8-41f4-a424-e082f9eae22c	15	80430522	80432550	16	2028	1	5	10	NA	NA	NA
Bone-Leiomyo	d8f0becd-fda8-41f4-a424-e082f9eae22c	22	24219372	24220632	8	1260	1	2	5	NA	NA	NA
Bone-Leiomyo	d8f0becd-fda8-41f4-a424-e082f9eae22c	22	28334139	28340679	11	6540	1	7	2	1	NA	NA
Bone-Leiomyo	d8f0becd-fda8-41f4-a424-e082f9eae22c	22	30577646	30581317	17	3671	3	8	6	NA	NA	NA
Bone-Leiomyo	d8f0becd-fda8-41f4-a424-e082f9eae22c	X	101873368	101877317	11	3949	3	3	5	NA	NA	NA
Panc-AdenoCA	d91f487e-0895-44ef-aeb1-a62bd576f8b0	1	95219996	95221847	9	1851	NA	1	8	NA	NA	NA
Panc-AdenoCA	d91f487e-0895-44ef-aeb1-a62bd576f8b0	4	31862914	31866115	22	3201	4	7	10	NA	NA	1
Panc-AdenoCA	d91f487e-0895-44ef-aeb1-a62bd576f8b0	12	24030614	24033176	14	2562	3	5	6	NA	NA	NA
Panc-AdenoCA	d91f487e-0895-44ef-aeb1-a62bd576f8b0	12	28069320	28071326	7	2006	NA	1	6	NA	NA	NA
Panc-AdenoCA	d91f487e-0895-44ef-aeb1-a62bd576f8b0	17	45289122	45289357	6	235	NA	1	5	NA	NA	NA
Panc-AdenoCA	d91f487e-0895-44ef-aeb1-a62bd576f8b0	17	57904334	57910667	8	6333	NA	2	4	NA	1	1
Panc-AdenoCA	d91f487e-0895-44ef-aeb1-a62bd576f8b0	17	59395684	59397651	8	1967	NA	6	2	NA	NA	NA
Panc-AdenoCA	d91f487e-0895-44ef-aeb1-a62bd576f8b0	17	59576938	59579471	12	2533	1	6	5	NA	NA	NA
Panc-AdenoCA	d91f487e-0895-44ef-aeb1-a62bd576f8b0	17	60117876	60118465	10	589	2	4	4	NA	NA	NA
Panc-AdenoCA	d91f487e-0895-44ef-aeb1-a62bd576f8b0	17	64728655	64728755	7	100	NA	3	4	NA	NA	NA
Kidney-RCC	d926a39f-d057-4e78-8907-b74f52157d99	3	76044655	76045749	8	1094	2	1	5	NA	NA	NA
Kidney-RCC	d926a39f-d057-4e78-8907-b74f52157d99	5	109500472	109500973	11	501	5	5	1	NA	NA	NA
Kidney-RCC	d926a39f-d057-4e78-8907-b74f52157d99	5	112083468	112086754	8	3286	NA	3	5	NA	NA	NA
ColoRect-AdenoCA	d9690a27-bb02-498e-b15c-ee76e31d16ee	1	242204380	242208453	6	4073	2	3	1	NA	NA	NA
Ovary-AdenoCA	d9e66fc5-9018-4568-b388-c5eb756f7823	10	135008734	135009587	8	853	2	2	4	NA	NA	NA
Ovary-AdenoCA	d9e66fc5-9018-4568-b388-c5eb756f7823	17	77649189	77651728	17	2539	5	9	3	NA	NA	NA
Ovary-AdenoCA	d9e66fc5-9018-4568-b388-c5eb756f7823	20	36623980	36628911	6	4931	2	NA	4	NA	NA	NA
Ovary-AdenoCA	d9e66fc5-9018-4568-b388-c5eb756f7823	20	43584637	43585956	7	1319	4	2	1	NA	NA	NA
Ovary-AdenoCA	d9e66fc5-9018-4568-b388-c5eb756f7823	20	43787440	43789242	9	1802	NA	NA	2	2	3	2
Ovary-AdenoCA	d9e66fc5-9018-4568-b388-c5eb756f7823	20	45128130	45128627	10	497	NA	7	3	NA	NA	NA
Ovary-AdenoCA	d9e66fc5-9018-4568-b388-c5eb756f7823	20	45943996	45946768	9	2772	2	NA	2	1	4	NA
Panc-AdenoCA	da1ed4b9-f428-48aa-834d-1d9673999044	6	9932072	9933839	7	1767	NA	5	2	NA	NA	NA
Panc-AdenoCA	da1ed4b9-f428-48aa-834d-1d9673999044	7	141736847	141737822	7	975	1	6	NA	NA	NA	NA
Panc-AdenoCA	da1ed4b9-f428-48aa-834d-1d9673999044	8	38046566	38050463	23	3897	2	10	11	NA	NA	NA
Panc-AdenoCA	da1ed4b9-f428-48aa-834d-1d9673999044	12	13629184	13631854	9	2670	3	5	1	NA	NA	NA
Panc-AdenoCA	da1ed4b9-f428-48aa-834d-1d9673999044	12	25849568	25852187	11	2619	NA	6	5	NA	NA	NA
Panc-AdenoCA	da1ed4b9-f428-48aa-834d-1d9673999044	13	74667739	74670378	9	2639	1	3	5	NA	NA	NA
Panc-AdenoCA	da1ed4b9-f428-48aa-834d-1d9673999044	15	83354682	83355039	7	357	3	2	2	NA	NA	NA
Panc-AdenoCA	da1ed4b9-f428-48aa-834d-1d9673999044	18	1716618	1717150	6	532	NA	NA	NA	1	5	NA
Panc-AdenoCA	da1ed4b9-f428-48aa-834d-1d9673999044	22	26393598	26394199	6	601	1	1	4	NA	NA	NA
Ovary-AdenoCA	da43386c-47f8-4e03-b6ca-8b94e13792e7	2	131834724	131834731	7	7	NA	2	2	1	2	NA
Breast-AdenoCa	dabb5a94-9706-4429-8db0-c328e2004340	8	30282009	30286153	16	4144	2	10	4	NA	NA	NA
Breast-AdenoCa	dabb5a94-9706-4429-8db0-c328e2004340	20	49190914	49192259	6	1345	3	2	NA	NA	1	NA
Panc-AdenoCA	db2a1df8-487d-4dad-a347-5c5ed539d5ad	15	43430792	43434686	24	3894	4	6	13	NA	NA	1
Breast-AdenoCa	db45d81f-695c-4f99-beea-6dbec1ff47fc	2	199794919	199795444	10	525	1	6	3	NA	NA	NA
Breast-AdenoCa	db45d81f-695c-4f99-beea-6dbec1ff47fc	3	66999658	67001426	6	1768	NA	2	4	NA	NA	NA
Breast-AdenoCa	db45d81f-695c-4f99-beea-6dbec1ff47fc	3	183615700	183620290	7	4590	1	1	5	NA	NA	NA
Breast-AdenoCa	db45d81f-695c-4f99-beea-6dbec1ff47fc	4	168879057	168881535	6	2478	NA	2	4	NA	NA	NA
Breast-AdenoCa	db45d81f-695c-4f99-beea-6dbec1ff47fc	5	65390289	65391385	6	1096	1	NA	5	NA	NA	NA
Breast-AdenoCa	db45d81f-695c-4f99-beea-6dbec1ff47fc	6	129617922	129621283	6	3361	1	4	1	NA	NA	NA
Breast-AdenoCa	db45d81f-695c-4f99-beea-6dbec1ff47fc	10	74701461	74707758	8	6297	NA	4	4	NA	NA	NA
Breast-AdenoCa	db45d81f-695c-4f99-beea-6dbec1ff47fc	11	60728515	60732595	9	4080	1	5	3	NA	NA	NA
Breast-AdenoCa	db45d81f-695c-4f99-beea-6dbec1ff47fc	11	66651827	66654845	8	3018	NA	2	6	NA	NA	NA
Breast-AdenoCa	db45d81f-695c-4f99-beea-6dbec1ff47fc	11	70226358	70228031	11	1673	1	7	3	NA	NA	NA

Breast-AdenoCa	db45d81f-695c-4f99-beea-6dbec1ff47fc	11	72021503	72025477	11	3974	1	3	7	NA	NA	NA
Breast-AdenoCa	db45d81f-695c-4f99-beea-6dbec1ff47fc	14	64494021	64511407	31	17386	3	1	27	NA	NA	NA
Breast-AdenoCa	db45d81f-695c-4f99-beea-6dbec1ff47fc	15	77087744	77093228	8	5484	NA	3	5	NA	NA	NA
Breast-AdenoCa	db45d81f-695c-4f99-beea-6dbec1ff47fc	17	37092296	37096376	6	4080	NA	4	1	NA	NA	1
Breast-AdenoCa	db45d81f-695c-4f99-beea-6dbec1ff47fc	21	36757985	36758419	6	434	NA	3	3	NA	NA	NA
Breast-AdenoCa	db45d81f-695c-4f99-beea-6dbec1ff47fc	X	29593924	29608394	19	14470	NA	3	16	NA	NA	NA
Breast-AdenoCa	db45d81f-695c-4f99-beea-6dbec1ff47fc	X	30036007	30039222	11	3215	1	1	3	4	NA	2
Breast-AdenoCa	db45d81f-695c-4f99-beea-6dbec1ff47fc	X	34997660	34998855	8	1195	2	2	4	NA	NA	NA
Breast-AdenoCa	db45d81f-695c-4f99-beea-6dbec1ff47fc	X	153253193	153254234	7	1041	NA	1	6	NA	NA	NA
Kidney-RCC	db4d1d04-1189-4c0e-99a7-904667af07b5	1	189115812	189117911	9	2099	7	1	1	NA	NA	NA
Lymph-BNHL	db9ce6c6-529c-4da6-92c4-f4f3fc3fe109	2	89159034	89160056	25	1022	NA	4	1	12	4	4
Lymph-BNHL	db9ce6c6-529c-4da6-92c4-f4f3fc3fe109	5	24066705	24066822	6	117	NA	NA	NA	2	3	1
Lymph-BNHL	db9ce6c6-529c-4da6-92c4-f4f3fc3fe109	14	106327029	106330546	47	3517	1	5	17	15	4	5
Lymph-BNHL	db9ce6c6-529c-4da6-92c4-f4f3fc3fe109	22	23230138	23233933	21	3795	1	4	4	6	4	2
Liver-HCC	dbfa4e8a-c622-11e3-bf01-24c6515278c0	2	89164015	89165315	6	1300	NA	1	NA	4	1	NA
Panc-Endocrine	dc4ba4bc-6333-4fe9-8805-e058cc9e6e18	1	8016292	8017269	6	977	2	2	2	NA	NA	NA
Panc-Endocrine	dc4ba4bc-6333-4fe9-8805-e058cc9e6e18	6	74507361	74508472	10	1111	2	3	5	NA	NA	NA
Panc-Endocrine	dc4ba4bc-6333-4fe9-8805-e058cc9e6e18	13	78820537	78822122	10	1585	3	2	5	NA	NA	NA
Lymph-BNHL	dc4bc4c4-2cc1-4a2e-a9f2-613088af17bf	1	216748538	216752063	12	3525	NA	NA	NA	6	2	4
Lymph-BNHL	dc4bc4c4-2cc1-4a2e-a9f2-613088af17bf	6	392081	393251	32	1170	2	6	20	1	2	1
Lymph-BNHL	dc4bc4c4-2cc1-4a2e-a9f2-613088af17bf	14	106211986	106213807	15	1821	1	2	11	NA	NA	1
Lymph-BNHL	dc4bc4c4-2cc1-4a2e-a9f2-613088af17bf	14	106323324	106329424	58	6100	3	8	17	20	4	6
Lymph-BNHL	dc4bc4c4-2cc1-4a2e-a9f2-613088af17bf	14	106452705	106452966	8	261	2	2	3	NA	NA	1
Lymph-BNHL	dc4bc4c4-2cc1-4a2e-a9f2-613088af17bf	22	23100514	23101564	9	1050	NA	1	6	1	NA	1
Prost-AdenoCA	dc85552c-2488-48d9-9da1-67a380945b85	21	35611208	35616649	7	5441	1	1	5	NA	NA	NA
Prost-AdenoCA	dc85552c-2488-48d9-9da1-67a380945b85	21	42854523	42855608	9	1085	1	NA	8	NA	NA	NA
Panc-AdenoCA	dc856038-f5f7-4dfc-a0a4-3e3a6b8d160f	1	207349688	207351562	9	1874	NA	3	6	NA	NA	NA
Panc-AdenoCA	dc856038-f5f7-4dfc-a0a4-3e3a6b8d160f	8	39134026	39136567	18	2541	1	3	13	NA	NA	1
Ovary-AdenoCA	dce54d09-9827-4fe2-abe1-c5b7d528ba7f	6	74850091	74854703	6	4612	1	2	3	NA	NA	NA
Ovary-AdenoCA	dce54d09-9827-4fe2-abe1-c5b7d528ba7f	7	91475023	91479050	9	4027	3	6	NA	NA	NA	NA
Head-SCC	dd67dec6-35dd-4efe-b913-ed4884855365	15	24169960	24170830	6	870	2	1	3	NA	NA	NA
Eso-AdenoCa	dd7d623b-b9af-4147-9aa6-e09793691f10	13	24029863	24031078	6	1215	NA	NA	NA	1	4	1
Lymph-BNHL	dd8f3e1c-6ed6-41c2-957d-6f679a3cb3ca	2	165414889	165418419	6	3530	2	NA	NA	1	2	1
Lymph-BNHL	dd8f3e1c-6ed6-41c2-957d-6f679a3cb3ca	11	38578100	38580039	6	1939	1	2	1	1	NA	1
Lymph-BNHL	dd8f3e1c-6ed6-41c2-957d-6f679a3cb3ca	11	89023440	89025310	7	1870	NA	NA	NA	3	1	3
Lymph-BNHL	dd8f3e1c-6ed6-41c2-957d-6f679a3cb3ca	14	106326300	106330424	9	4124	1	3	5	NA	NA	NA
Lymph-BNHL	dd8f3e1c-6ed6-41c2-957d-6f679a3cb3ca	18	28549278	28551930	7	2652	NA	NA	1	2	4	NA
Lymph-BNHL	dd8f3e1c-6ed6-41c2-957d-6f679a3cb3ca	22	23236220	23237129	8	909	2	4	2	NA	NA	NA
Eso-AdenoCa	de581588-be15-4bf4-95d1-aa5478b53ab6	5	36225340	36225944	10	604	NA	7	3	NA	NA	NA
Eso-AdenoCa	de581588-be15-4bf4-95d1-aa5478b53ab6	9	33275137	33276817	7	1680	NA	5	2	NA	NA	NA
Eso-AdenoCa	de581588-be15-4bf4-95d1-aa5478b53ab6	17	39276723	39278586	8	1863	2	2	4	NA	NA	NA
Head-SCC	de8ef60b-4dbe-4aa8-adef-6f58cdfada29	11	57691385	57693716	7	2331	1	6	NA	NA	NA	NA
Lymph-CLL	de99a4de-e916-4572-ac9e-73e341827229	2	89159475	89215113	80	55638	3	9	7	30	11	20
Lymph-CLL	de99a4de-e916-4572-ac9e-73e341827229	14	106238431	106240967	6	2536	NA	1	4	NA	1	NA
Lymph-CLL	de99a4de-e916-4572-ac9e-73e341827229	14	106324584	106351481	72	26897	4	12	15	21	7	13
Lymph-CLL	de99a4de-e916-4572-ac9e-73e341827229	14	106829709	106830576	16	867	1	2	NA	9	1	3
Lymph-CLL	de99a4de-e916-4572-ac9e-73e341827229	22	23229693	23231690	7	1997	NA	2	2	2	1	NA
Uterus-AdenoCA	dec775c5-7d9a-4dc5-b399-dc4b7ba49d73	5	52537097	52540528	14	3431	2	2	10	NA	NA	NA
Uterus-AdenoCA	dec775c5-7d9a-4dc5-b399-dc4b7ba49d73	11	60838474	60840532	7	2058	1	3	3	NA	NA	NA
Uterus-AdenoCA	dec775c5-7d9a-4dc5-b399-dc4b7ba49d73	19	29157084	29163797	9	6713	1	4	4	NA	NA	NA
Panc-AdenoCA	ded2689b-8e37-480c-b37c-acc538e54ddf	1	107238869	107244830	11	5961	1	6	4	NA	NA	NA
Panc-AdenoCA	ded2689b-8e37-480c-b37c-acc538e54ddf	3	89745540	89746063	7	523	NA	3	4	NA	NA	NA
Panc-AdenoCA	ded2689b-8e37-480c-b37c-acc538e54ddf	5	63817536	63818214	6	678	1	1	4	NA	NA	NA
Panc-AdenoCA	ded2689b-8e37-480c-b37c-acc538e54ddf	5	112401238	112403759	10	2521	2	4	4	NA	NA	NA
Panc-AdenoCA	ded2689b-8e37-480c-b37c-acc538e54ddf	9	21879704	21880397	6	693	1	4	1	NA	NA	NA
Panc-AdenoCA	ded2689b-8e37-480c-b37c-acc538e54ddf	9	30586131	30588625	14	2494	2	9	3	NA	NA	NA
Panc-AdenoCA	ded2689b-8e37-480c-b37c-acc538e54ddf	16	50625599	50628058	9	2459	NA	5	4	NA	NA	NA
Panc-AdenoCA	ded2689b-8e37-480c-b37c-acc538e54ddf	18	48591213	48592384	13	1171	NA	1	12	NA	NA	NA
Eso-AdenoCa	ded8b673-53df-4038-a375-7cd0fd2a1b9f	17	67891537	67901805	16	10268	2	1	13	NA	NA	NA
Breast-AdenoCa	df291849-4c35-44e1-b013-8f6b7ee36113	1	43103936	43106024	7	2088	4	3	NA	NA	NA	NA

Breast-AdenoCa	df291849-4c35-44e1-b013-8f6b7ee36113	1	162152440	162153436	9	996	9	NA	NA	NA	NA	NA
Breast-AdenoCa	df291849-4c35-44e1-b013-8f6b7ee36113	1	171494253	171495905	11	1652	2	6	2	NA	NA	1
Breast-AdenoCa	df291849-4c35-44e1-b013-8f6b7ee36113	1	174410344	174411066	7	722	1	4	2	NA	NA	NA
Breast-AdenoCa	df291849-4c35-44e1-b013-8f6b7ee36113	2	174755088	174756170	9	1082	NA	6	3	NA	NA	NA
Breast-AdenoCa	df291849-4c35-44e1-b013-8f6b7ee36113	4	61480398	61481196	6	798	2	1	3	NA	NA	NA
Breast-AdenoCa	df291849-4c35-44e1-b013-8f6b7ee36113	8	37066507	37068171	11	1664	1	7	3	NA	NA	NA
Breast-AdenoCa	df291849-4c35-44e1-b013-8f6b7ee36113	8	65572941	65574191	15	1250	8	6	1	NA	NA	NA
Breast-AdenoCa	df291849-4c35-44e1-b013-8f6b7ee36113	8	81346585	81346889	9	304	3	4	2	NA	NA	NA
Breast-AdenoCa	df291849-4c35-44e1-b013-8f6b7ee36113	12	76310843	76315410	6	4567	1	1	4	NA	NA	NA
Breast-AdenoCa	df291849-4c35-44e1-b013-8f6b7ee36113	12	84239829	84245419	11	5590	2	3	6	NA	NA	NA
Breast-AdenoCa	df291849-4c35-44e1-b013-8f6b7ee36113	14	26121286	26129617	12	8331	2	4	5	NA	1	NA
Breast-AdenoCa	df291849-4c35-44e1-b013-8f6b7ee36113	16	14165808	14167197	12	1389	3	5	2	1	NA	1
Breast-AdenoCa	df291849-4c35-44e1-b013-8f6b7ee36113	19	32357142	32359223	10	2081	5	4	1	NA	NA	NA
Breast-AdenoCa	df291849-4c35-44e1-b013-8f6b7ee36113	19	33789211	33791509	13	2298	4	8	1	NA	NA	NA
Breast-AdenoCa	df291849-4c35-44e1-b013-8f6b7ee36113	20	33716320	33718617	9	2297	3	6	NA	NA	NA	NA
Breast-AdenoCa	df291849-4c35-44e1-b013-8f6b7ee36113	22	21944218	21949705	9	5487	NA	5	4	NA	NA	NA
Bone-Leiomyo	df4366c4-170f-4233-b577-a8ea277b069c	1	36316207	36316860	6	653	3	1	2	NA	NA	NA
Bone-Leiomyo	df4366c4-170f-4233-b577-a8ea277b069c	1	182016875	182017066	7	191	2	2	3	NA	NA	NA
Bone-Leiomyo	df4366c4-170f-4233-b577-a8ea277b069c	1	221543766	221547390	11	3624	3	2	6	NA	NA	NA
Bone-Leiomyo	df4366c4-170f-4233-b577-a8ea277b069c	1	248445629	248450707	20	5078	2	9	9	NA	NA	NA
Bone-Leiomyo	df4366c4-170f-4233-b577-a8ea277b069c	2	13636774	13638928	6	2154	3	1	2	NA	NA	NA
Bone-Leiomyo	df4366c4-170f-4233-b577-a8ea277b069c	2	24854779	24855006	7	227	2	2	3	NA	NA	NA
Bone-Leiomyo	df4366c4-170f-4233-b577-a8ea277b069c	2	27598864	27599243	12	379	NA	NA	12	NA	NA	NA
Bone-Leiomyo	df4366c4-170f-4233-b577-a8ea277b069c	2	35184758	35185861	7	1103	NA	3	4	NA	NA	NA
Bone-Leiomyo	df4366c4-170f-4233-b577-a8ea277b069c	2	177562247	177563572	10	1325	1	4	4	NA	NA	1
Bone-Leiomyo	df4366c4-170f-4233-b577-a8ea277b069c	2	233265034	233267849	8	2815	NA	1	7	NA	NA	NA
Bone-Leiomyo	df4366c4-170f-4233-b577-a8ea277b069c	4	37012025	37015013	11	2988	2	4	5	NA	NA	NA
Bone-Leiomyo	df4366c4-170f-4233-b577-a8ea277b069c	5	6799029	6801564	7	2535	2	1	4	NA	NA	NA
Bone-Leiomyo	df4366c4-170f-4233-b577-a8ea277b069c	5	126053219	126059936	12	6717	2	5	5	NA	NA	NA
Bone-Leiomyo	df4366c4-170f-4233-b577-a8ea277b069c	5	135422875	135427377	10	4502	2	8	NA	NA	NA	NA
Bone-Leiomyo	df4366c4-170f-4233-b577-a8ea277b069c	6	99011008	99013932	9	2924	2	2	5	NA	NA	NA
Bone-Leiomyo	df4366c4-170f-4233-b577-a8ea277b069c	7	123018018	123018145	11	127	NA	NA	11	NA	NA	NA
Bone-Leiomyo	df4366c4-170f-4233-b577-a8ea277b069c	7	132065118	132065300	13	182	NA	1	12	NA	NA	NA
Bone-Leiomyo	df4366c4-170f-4233-b577-a8ea277b069c	7	133277491	133278647	10	1156	1	6	3	NA	NA	NA
Bone-Leiomyo	df4366c4-170f-4233-b577-a8ea277b069c	8	626393	626632	6	239	NA	NA	6	NA	NA	NA
Bone-Leiomyo	df4366c4-170f-4233-b577-a8ea277b069c	8	1392189	1399037	9	6848	1	NA	8	NA	NA	NA
Bone-Leiomyo	df4366c4-170f-4233-b577-a8ea277b069c	9	78086388	78089063	11	2675	1	6	4	NA	NA	NA
Bone-Leiomyo	df4366c4-170f-4233-b577-a8ea277b069c	11	9179719	9182942	6	3223	1	2	3	NA	NA	NA
Bone-Leiomyo	df4366c4-170f-4233-b577-a8ea277b069c	12	55847774	55848510	6	736	NA	1	5	NA	NA	NA
Bone-Leiomyo	df4366c4-170f-4233-b577-a8ea277b069c	12	56281308	56288225	11	6917	3	2	6	NA	NA	NA
Bone-Leiomyo	df4366c4-170f-4233-b577-a8ea277b069c	12	58536337	58538507	8	2170	NA	5	3	NA	NA	NA
Bone-Leiomyo	df4366c4-170f-4233-b577-a8ea277b069c	12	65296408	65298897	12	2489	1	5	6	NA	NA	NA
Bone-Leiomyo	df4366c4-170f-4233-b577-a8ea277b069c	12	68580608	68583065	16	2457	NA	3	13	NA	NA	NA
Bone-Leiomyo	df4366c4-170f-4233-b577-a8ea277b069c	12	76843800	76845982	15	2182	2	5	8	NA	NA	NA
Bone-Leiomyo	df4366c4-170f-4233-b577-a8ea277b069c	12	126131666	126133894	10	2228	1	6	2	1	NA	NA
Bone-Leiomyo	df4366c4-170f-4233-b577-a8ea277b069c	18	62528659	62529002	6	343	1	2	3	NA	NA	NA
Bone-Leiomyo	df4366c4-170f-4233-b577-a8ea277b069c	20	8351119	8355051	9	3932	2	1	6	NA	NA	NA
Bone-Leiomyo	df4366c4-170f-4233-b577-a8ea277b069c	X	103189312	103190336	6	1024	NA	NA	6	NA	NA	NA
Bone-Leiomyo	df4366c4-170f-4233-b577-a8ea277b069c	X	103527888	103530230	9	2342	1	NA	8	NA	NA	NA
Bone-Leiomyo	df4366c4-170f-4233-b577-a8ea277b069c	X	154205155	154211627	10	6472	NA	NA	10	NA	NA	NA
Lymph-BNHL	df814571-57f1-4e55-bc1a-c892c568c4ba	2	89158123	89161238	31	3115	1	6	10	10	1	3
Lymph-BNHL	df814571-57f1-4e55-bc1a-c892c568c4ba	8	128748945	128752635	12	3690	NA	2	5	3	1	1
Lymph-BNHL	df814571-57f1-4e55-bc1a-c892c568c4ba	14	106325124	106383201	60	58077	3	19	21	13	3	1
Lymph-BNHL	df814571-57f1-4e55-bc1a-c892c568c4ba	22	23204163	23204717	8	554	NA	NA	1	3	2	2
Lymph-BNHL	df814571-57f1-4e55-bc1a-c892c568c4ba	22	23230196	23234668	25	4472	1	6	12	5	NA	1
Kidney-RCC	e053d377-e4f2-4aee-af7d-e61605e0f4f5	X	147029527	147029751	8	224	4	3	1	NA	NA	NA
Cervix-SCC	e07991a4-0901-4ed1-8704-aa376565d862	1	16014621	16018210	7	3589	NA	4	3	NA	NA	NA
Cervix-SCC	e07991a4-0901-4ed1-8704-aa376565d862	6	128980834	128983924	7	3090	NA	NA	7	NA	NA	NA
Cervix-SCC	e07991a4-0901-4ed1-8704-aa376565d862	7	101455328	101457369	6	2041	1	4	1	NA	NA	NA
Cervix-SCC	e07991a4-0901-4ed1-8704-aa376565d862	11	122594817	122596475	11	1658	3	1	7	NA	NA	NA

Cervix-SCC	e07991a4-0901-4ed1-8704-aa376565d862	19	51359309	51361307	6	1998	NA	3	3	NA	NA	NA
Panc-AdenoCA	e0aa815f-668a-4de9-bb32-3f215bb7f843	2	96917502	96919245	6	1743	1	2	3	NA	NA	NA
Panc-AdenoCA	e0aa815f-668a-4de9-bb32-3f215bb7f843	3	14504412	14509431	7	5019	NA	3	4	NA	NA	NA
Panc-AdenoCA	e0aa815f-668a-4de9-bb32-3f215bb7f843	4	48516435	48519934	9	3499	2	3	4	NA	NA	NA
Panc-AdenoCA	e0aa815f-668a-4de9-bb32-3f215bb7f843	9	21717629	21718993	9	1364	1	6	2	NA	NA	NA
Panc-AdenoCA	e1df938f-1f23-4291-98ef-13db6debb796	6	22910842	22917736	13	6894	1	7	5	NA	NA	NA
Lymph-BNHL	e2b09705-c5c8-48ee-a90e-19648a7bf2cb	2	89157325	89197289	72	39964	2	12	21	19	7	11
Lymph-BNHL	e2b09705-c5c8-48ee-a90e-19648a7bf2cb	3	18099578	18104124	6	4546	1	NA	NA	1	NA	4
Lymph-BNHL	e2b09705-c5c8-48ee-a90e-19648a7bf2cb	3	20322165	20333127	13	10962	1	NA	1	8	NA	3
Lymph-BNHL	e2b09705-c5c8-48ee-a90e-19648a7bf2cb	3	164226698	164234652	10	7954	NA	1	2	5	2	NA
Lymph-BNHL	e2b09705-c5c8-48ee-a90e-19648a7bf2cb	4	104912043	104922811	14	10768	NA	NA	7	3	2	2
Lymph-BNHL	e2b09705-c5c8-48ee-a90e-19648a7bf2cb	5	103671914	103687531	23	15617	NA	NA	NA	12	2	9
Lymph-BNHL	e2b09705-c5c8-48ee-a90e-19648a7bf2cb	5	168747129	168752302	9	5173	NA	NA	2	3	1	3
Lymph-BNHL	e2b09705-c5c8-48ee-a90e-19648a7bf2cb	6	40120611	40125282	6	4671	1	1	1	2	1	NA
Lymph-BNHL	e2b09705-c5c8-48ee-a90e-19648a7bf2cb	6	79294330	79295056	7	726	NA	NA	NA	6	1	NA
Lymph-BNHL	e2b09705-c5c8-48ee-a90e-19648a7bf2cb	11	24745863	24758814	19	12951	4	2	4	NA	2	7
Lymph-BNHL	e2b09705-c5c8-48ee-a90e-19648a7bf2cb	12	87944114	87947805	8	3691	NA	2	1	3	NA	2
Lymph-BNHL	e2b09705-c5c8-48ee-a90e-19648a7bf2cb	13	81745171	81749234	7	4063	NA	NA	NA	2	2	3
Lymph-BNHL	e2b09705-c5c8-48ee-a90e-19648a7bf2cb	14	84613409	84620975	11	7566	NA	1	1	5	NA	4
Lymph-BNHL	e2b09705-c5c8-48ee-a90e-19648a7bf2cb	14	106211512	106212487	12	975	1	4	7	NA	NA	NA
Lymph-BNHL	e2b09705-c5c8-48ee-a90e-19648a7bf2cb	14	106323842	106330012	53	6170	5	12	21	7	1	7
Lymph-BNHL	e2b09705-c5c8-48ee-a90e-19648a7bf2cb	14	106573618	106579740	33	6122	NA	3	12	11	3	4
Lymph-BNHL	e2b09705-c5c8-48ee-a90e-19648a7bf2cb	18	60983695	60988231	31	4536	2	6	7	5	7	4
Lymph-BNHL	e2b09705-c5c8-48ee-a90e-19648a7bf2cb	22	23028899	23035337	10	6438	NA	2	4	NA	1	3
Lymph-BNHL	e2b09705-c5c8-48ee-a90e-19648a7bf2cb	22	23039920	23041379	9	1459	1	2	3	2	NA	1
Lymph-BNHL	e2b09705-c5c8-48ee-a90e-19648a7bf2cb	X	32312421	32315936	13	3515	3	2	3	4	NA	1
Lymph-BNHL	e2b09705-c5c8-48ee-a90e-19648a7bf2cb	X	100580958	100584966	8	4008	NA	NA	2	4	NA	2
Head-SCC	e2e6a409-b64d-463c-ac3b-e7987a3124f0	2	202105725	202111350	33	5625	4	12	17	NA	NA	NA
Head-SCC	e2e6a409-b64d-463c-ac3b-e7987a3124f0	9	114625015	114627186	6	2171	NA	3	3	NA	NA	NA
Head-SCC	e2e6a409-b64d-463c-ac3b-e7987a3124f0	11	18084825	18087068	6	2243	1	1	4	NA	NA	NA
Lymph-BNHL	e2fa7251-507e-4d76-95a3-a228adc3885a	2	35540005	35542867	6	2862	1	NA	NA	2	1	2
Lymph-BNHL	e2fa7251-507e-4d76-95a3-a228adc3885a	2	89155476	89247653	95	92177	1	17	5	37	20	15
Lymph-BNHL	e2fa7251-507e-4d76-95a3-a228adc3885a	14	40972401	40976409	8	4008	NA	NA	1	1	1	5
Lymph-BNHL	e2fa7251-507e-4d76-95a3-a228adc3885a	14	106094322	106095489	7	1167	1	2	2	1	NA	1
Lymph-BNHL	e2fa7251-507e-4d76-95a3-a228adc3885a	14	106112702	106114310	12	1608	1	4	6	NA	NA	1
Lymph-BNHL	e2fa7251-507e-4d76-95a3-a228adc3885a	14	106210665	106231597	29	20932	1	13	12	3	NA	NA
Lymph-BNHL	e2fa7251-507e-4d76-95a3-a228adc3885a	14	106323353	106369717	117	46364	3	21	31	29	14	19
Lymph-BNHL	e2fa7251-507e-4d76-95a3-a228adc3885a	14	106573618	106579577	22	5959	NA	5	2	13	1	1
Lymph-BNHL	e2fa7251-507e-4d76-95a3-a228adc3885a	22	23204552	23210178	11	5626	1	NA	2	2	4	2
Lymph-BNHL	e2fa7251-507e-4d76-95a3-a228adc3885a	22	23223389	23232996	53	9607	1	8	7	21	10	6
Ovary-AdenoCA	e45f3391-2e74-4767-817a-280cebac7c57	10	79537698	79540853	25	3155	1	15	9	NA	NA	NA
Ovary-AdenoCA	e45f3391-2e74-4767-817a-280cebac7c57	12	60906674	60908008	6	1334	1	NA	NA	3	NA	2
Ovary-AdenoCA	e45f3391-2e74-4767-817a-280cebac7c57	16	27450741	27457701	9	6960	2	1	6	NA	NA	NA
Ovary-AdenoCA	e45f3391-2e74-4767-817a-280cebac7c57	16	27461091	27467814	8	6723	4	3	1	NA	NA	NA
Ovary-AdenoCA	e4aaca83-3ae9-47f6-a975-c144767ad705	1	37876563	37876977	7	414	1	2	4	NA	NA	NA
Ovary-AdenoCA	e4aaca83-3ae9-47f6-a975-c144767ad705	1	43862028	43863678	10	1650	2	3	5	NA	NA	NA
Ovary-AdenoCA	e4aaca83-3ae9-47f6-a975-c144767ad705	1	230780814	230782332	8	1518	1	5	2	NA	NA	NA
Ovary-AdenoCA	e4aaca83-3ae9-47f6-a975-c144767ad705	8	142567772	142572383	7	4611	1	4	2	NA	NA	NA
Ovary-AdenoCA	e4aaca83-3ae9-47f6-a975-c144767ad705	10	55560205	55561963	8	1758	3	2	2	NA	1	NA
Ovary-AdenoCA	e4aaca83-3ae9-47f6-a975-c144767ad705	11	121585407	121590173	15	4766	1	10	4	NA	NA	NA
Ovary-AdenoCA	e4aaca83-3ae9-47f6-a975-c144767ad705	12	20328616	20328850	7	234	NA	4	3	NA	NA	NA
Ovary-AdenoCA	e4aaca83-3ae9-47f6-a975-c144767ad705	17	50809490	50813714	10	4224	1	7	2	NA	NA	NA
Ovary-AdenoCA	e4aaca83-3ae9-47f6-a975-c144767ad705	17	50876161	50877277	7	1116	NA	2	5	NA	NA	NA
Ovary-AdenoCA	e4aaca83-3ae9-47f6-a975-c144767ad705	19	33465216	33472828	10	7612	3	2	5	NA	NA	NA
Ovary-AdenoCA	e4aaca83-3ae9-47f6-a975-c144767ad705	19	37302105	37302443	7	338	3	2	2	NA	NA	NA
Panc-AdenoCA	e4c8c7f0-5bac-4d59-91c4-c98207150548	12	113346709	113346740	7	31	2	NA	3	NA	2	NA
Biliary-AdenoCA	e4fd1b3e-c622-11e3-bf01-24c6515278c0	5	51677634	51678061	7	427	NA	1	6	NA	NA	NA
Panc-AdenoCA	e5193d7e-e8b7-4098-bf98-8b3a62781d13	2	77777923	77782490	6	4567	NA	NA	NA	2	1	3
Panc-AdenoCA	e5193d7e-e8b7-4098-bf98-8b3a62781d13	11	118674742	118676443	8	1701	2	2	4	NA	NA	NA
Panc-AdenoCA	e5193d7e-e8b7-4098-bf98-8b3a62781d13	X	98825231	98826574	9	1343	NA	NA	8	NA	NA	1

Breast-AdenoCa	e5593865-5f8e-4a4c-b36f-73fbe64d66da	6	157975878	157977622	16	1744	NA	NA	16	NA	NA	NA
Breast-AdenoCa	e5593865-5f8e-4a4c-b36f-73fbe64d66da	7	8365317	8365720	8	403	NA	NA	8	NA	NA	NA
Breast-AdenoCa	e5593865-5f8e-4a4c-b36f-73fbe64d66da	7	10064919	10067371	11	2452	3	6	2	NA	NA	NA
Breast-AdenoCa	e5593865-5f8e-4a4c-b36f-73fbe64d66da	7	21850956	21851755	14	799	NA	NA	14	NA	NA	NA
Breast-AdenoCa	e5593865-5f8e-4a4c-b36f-73fbe64d66da	8	37658687	37659860	10	1173	NA	NA	10	NA	NA	NA
Breast-AdenoCa	e5593865-5f8e-4a4c-b36f-73fbe64d66da	8	37699688	37700919	19	1231	NA	NA	19	NA	NA	NA
Breast-AdenoCa	e5593865-5f8e-4a4c-b36f-73fbe64d66da	8	56070103	56071512	21	1409	NA	NA	21	NA	NA	NA
Breast-AdenoCa	e5593865-5f8e-4a4c-b36f-73fbe64d66da	8	58414800	58444071	52	29271	NA	NA	52	NA	NA	NA
Breast-AdenoCa	e5593865-5f8e-4a4c-b36f-73fbe64d66da	8	58483757	58484545	17	788	NA	1	16	NA	NA	NA
Breast-AdenoCa	e5593865-5f8e-4a4c-b36f-73fbe64d66da	8	70938658	70939101	13	443	NA	NA	13	NA	NA	NA
Breast-AdenoCa	e5593865-5f8e-4a4c-b36f-73fbe64d66da	8	71149010	71171319	71	22309	NA	NA	71	NA	NA	NA
Breast-AdenoCa	e5593865-5f8e-4a4c-b36f-73fbe64d66da	8	72928230	72931142	11	2912	NA	NA	11	NA	NA	NA
Breast-AdenoCa	e5593865-5f8e-4a4c-b36f-73fbe64d66da	16	51784340	51805279	27	20939	2	6	18	1	NA	NA
Breast-AdenoCa	e5593865-5f8e-4a4c-b36f-73fbe64d66da	17	27892449	27892905	6	456	NA	NA	6	NA	NA	NA
Breast-AdenoCa	e5593865-5f8e-4a4c-b36f-73fbe64d66da	17	35205858	35207054	7	1196	2	3	2	NA	NA	NA
Panc-AdenoCA	e56b0990-ff67-47c1-b9ad-87ef1d4aa2ae	2	28098419	28098984	6	565	NA	1	5	NA	NA	NA
Panc-AdenoCA	e56b0990-ff67-47c1-b9ad-87ef1d4aa2ae	4	111731332	111732841	8	1509	NA	2	6	NA	NA	NA
Panc-AdenoCA	e56b0990-ff67-47c1-b9ad-87ef1d4aa2ae	4	147990352	147996249	22	5897	2	9	11	NA	NA	NA
Panc-AdenoCA	e56b0990-ff67-47c1-b9ad-87ef1d4aa2ae	5	103864390	103870347	12	5957	1	NA	NA	NA	2	9
Panc-AdenoCA	e56b0990-ff67-47c1-b9ad-87ef1d4aa2ae	8	86466320	86467667	6	1347	NA	NA	NA	3	2	1
Panc-AdenoCA	e56b0990-ff67-47c1-b9ad-87ef1d4aa2ae	14	97298183	97302515	7	4332	NA	NA	NA	3	4	NA
Panc-AdenoCA	e56b0990-ff67-47c1-b9ad-87ef1d4aa2ae	21	16626404	16627179	6	775	NA	4	2	NA	NA	NA
Panc-AdenoCA	e56b0990-ff67-47c1-b9ad-87ef1d4aa2ae	22	22422599	22423361	13	762	5	3	5	NA	NA	NA
Panc-AdenoCA	e56b0990-ff67-47c1-b9ad-87ef1d4aa2ae	22	27022166	27023243	10	1077	1	1	8	NA	NA	NA
Lymph-BNHL	e6168db0-d12f-4218-8ab9-bd704201cb2b	1	203274775	203276001	30	1226	5	7	12	3	1	2
Lymph-BNHL	e6168db0-d12f-4218-8ab9-bd704201cb2b	1	226921342	226926196	11	4854	NA	3	4	2	1	1
Lymph-BNHL	e6168db0-d12f-4218-8ab9-bd704201cb2b	2	36097584	36102516	6	4932	NA	NA	2	2	NA	2
Lymph-BNHL	e6168db0-d12f-4218-8ab9-bd704201cb2b	2	81744955	81749802	9	4847	NA	NA	NA	6	2	1
Lymph-BNHL	e6168db0-d12f-4218-8ab9-bd704201cb2b	2	89127688	89134144	9	6456	NA	3	2	2	NA	2
Lymph-BNHL	e6168db0-d12f-4218-8ab9-bd704201cb2b	2	89157390	89159876	68	2486	3	2	10	28	13	12
Lymph-BNHL	e6168db0-d12f-4218-8ab9-bd704201cb2b	2	89247182	89248026	30	844	NA	1	7	12	6	4
Lymph-BNHL	e6168db0-d12f-4218-8ab9-bd704201cb2b	2	189538431	189558722	23	20291	2	7	4	7	2	1
Lymph-BNHL	e6168db0-d12f-4218-8ab9-bd704201cb2b	3	77617391	77621021	16	3630	2	4	1	6	1	2
Lymph-BNHL	e6168db0-d12f-4218-8ab9-bd704201cb2b	3	84059681	84065411	7	5730	NA	NA	NA	4	2	1
Lymph-BNHL	e6168db0-d12f-4218-8ab9-bd704201cb2b	3	187461912	187463782	28	1870	2	6	5	8	4	3
Lymph-BNHL	e6168db0-d12f-4218-8ab9-bd704201cb2b	4	11072386	11072618	6	232	1	NA	5	NA	NA	NA
Lymph-BNHL	e6168db0-d12f-4218-8ab9-bd704201cb2b	4	40195908	40200747	17	4839	NA	3	2	6	3	3
Lymph-BNHL	e6168db0-d12f-4218-8ab9-bd704201cb2b	4	131845470	131851399	7	5929	1	NA	NA	1	1	4
Lymph-BNHL	e6168db0-d12f-4218-8ab9-bd704201cb2b	5	28275561	28280702	7	5141	NA	NA	NA	3	1	3
Lymph-BNHL	e6168db0-d12f-4218-8ab9-bd704201cb2b	5	149791471	149796420	8	4949	NA	NA	3	1	2	2
Lymph-BNHL	e6168db0-d12f-4218-8ab9-bd704201cb2b	6	14118181	14119035	6	854	1	4	NA	1	NA	NA
Lymph-BNHL	e6168db0-d12f-4218-8ab9-bd704201cb2b	7	71609679	71614668	6	4989	NA	4	NA	NA	2	NA
Lymph-BNHL	e6168db0-d12f-4218-8ab9-bd704201cb2b	7	82431072	82432804	7	1732	NA	NA	NA	4	NA	3
Lymph-BNHL	e6168db0-d12f-4218-8ab9-bd704201cb2b	7	83580974	83583725	7	2751	NA	NA	2	5	NA	NA
Lymph-BNHL	e6168db0-d12f-4218-8ab9-bd704201cb2b	8	2859862	2862750	8	2888	1	NA	2	5	NA	NA
Lymph-BNHL	e6168db0-d12f-4218-8ab9-bd704201cb2b	8	34713591	34721483	11	7892	NA	2	4	2	1	2
Lymph-BNHL	e6168db0-d12f-4218-8ab9-bd704201cb2b	8	109995341	109997352	9	2011	NA	5	4	NA	NA	NA
Lymph-BNHL	e6168db0-d12f-4218-8ab9-bd704201cb2b	9	28494099	28499664	7	5565	NA	1	NA	3	NA	3
Lymph-BNHL	e6168db0-d12f-4218-8ab9-bd704201cb2b	9	37383943	37386962	10	3019	NA	4	2	2	1	1
Lymph-BNHL	e6168db0-d12f-4218-8ab9-bd704201cb2b	11	39928113	39941211	20	13098	1	1	5	4	7	2
Lymph-BNHL	e6168db0-d12f-4218-8ab9-bd704201cb2b	11	90519810	90525471	7	5661	2	1	NA	NA	1	3
Lymph-BNHL	e6168db0-d12f-4218-8ab9-bd704201cb2b	11	102188536	102189572	9	1036	NA	1	1	1	4	2
Lymph-BNHL	e6168db0-d12f-4218-8ab9-bd704201cb2b	12	113493864	113511662	36	17798	1	7	14	10	1	3
Lymph-BNHL	e6168db0-d12f-4218-8ab9-bd704201cb2b	12	122458512	122463215	20	4703	1	5	5	1	6	2
Lymph-BNHL	e6168db0-d12f-4218-8ab9-bd704201cb2b	13	23715932	23718556	7	2624	NA	2	5	NA	NA	NA
Lymph-BNHL	e6168db0-d12f-4218-8ab9-bd704201cb2b	13	70123286	70128939	11	5653	NA	NA	2	5	1	3
Lymph-BNHL	e6168db0-d12f-4218-8ab9-bd704201cb2b	14	51260555	51261448	7	893	NA	NA	2	NA	4	1
Lymph-BNHL	e6168db0-d12f-4218-8ab9-bd704201cb2b	14	51749051	51760104	14	11053	2	6	NA	4	1	1
Lymph-BNHL	e6168db0-d12f-4218-8ab9-bd704201cb2b	14	64192974	64193544	8	570	1	NA	1	3	1	2
Lymph-BNHL	e6168db0-d12f-4218-8ab9-bd704201cb2b	14	69258266	69259492	10	1226	NA	1	8	1	NA	NA

Lymph-BNHL	e6168db0-d12f-4218-8ab9-bd704201cb2b	14	106212568	106285376	91	72808	11	20	48	7	4	1
Lymph-BNHL	e6168db0-d12f-4218-8ab9-bd704201cb2b	14	106322791	106339117	169	16326	8	27	47	41	16	30
Lymph-BNHL	e6168db0-d12f-4218-8ab9-bd704201cb2b	14	107112925	107114114	15	1189	2	1	3	4	3	2
Lymph-BNHL	e6168db0-d12f-4218-8ab9-bd704201cb2b	15	27650276	27655147	10	4871	1	3	5	1	NA	NA
Lymph-BNHL	e6168db0-d12f-4218-8ab9-bd704201cb2b	15	41819073	41822802	13	3729	NA	NA	13	NA	NA	NA
Lymph-BNHL	e6168db0-d12f-4218-8ab9-bd704201cb2b	18	19099573	19103661	18	4088	3	7	7	NA	NA	1
Lymph-BNHL	e6168db0-d12f-4218-8ab9-bd704201cb2b	19	10340860	10341565	11	705	2	NA	6	2	NA	1
Lymph-BNHL	e6168db0-d12f-4218-8ab9-bd704201cb2b	20	3532716	3532984	6	268	NA	NA	NA	4	1	1
Lymph-BNHL	e6168db0-d12f-4218-8ab9-bd704201cb2b	21	21877568	21884155	8	6587	NA	NA	2	3	NA	3
Lymph-BNHL	e6168db0-d12f-4218-8ab9-bd704201cb2b	21	21886965	21891958	8	4993	1	1	1	4	NA	1
Lymph-BNHL	e6168db0-d12f-4218-8ab9-bd704201cb2b	21	21907763	21913500	7	5737	NA	NA	NA	2	2	3
Lymph-BNHL	e6168db0-d12f-4218-8ab9-bd704201cb2b	22	23198865	23199575	9	710	NA	NA	7	2	NA	NA
Lymph-BNHL	e6168db0-d12f-4218-8ab9-bd704201cb2b	22	23223132	23235628	100	12496	4	22	33	22	6	13
Lymph-BNHL	e6168db0-d12f-4218-8ab9-bd704201cb2b	X	64071688	64072158	6	470	NA	1	NA	2	1	2
Bladder-TCC	e6c78a98-f45b-482b-a551-4f11b8c1ff8b	1	88887238	88890134	11	2896	1	8	2	NA	NA	NA
Bladder-TCC	e6c78a98-f45b-482b-a551-4f11b8c1ff8b	1	150606414	150612322	11	5908	1	4	6	NA	NA	NA
Bladder-TCC	e6c78a98-f45b-482b-a551-4f11b8c1ff8b	2	19160708	19164445	6	3737	NA	5	1	NA	NA	NA
Bladder-TCC	e6c78a98-f45b-482b-a551-4f11b8c1ff8b	2	136229468	136230808	7	1340	NA	1	6	NA	NA	NA
Bladder-TCC	e6c78a98-f45b-482b-a551-4f11b8c1ff8b	3	52629794	52632341	7	2547	NA	4	3	NA	NA	NA
Bladder-TCC	e6c78a98-f45b-482b-a551-4f11b8c1ff8b	3	80055744	80057627	11	1883	NA	6	5	NA	NA	NA
Bladder-TCC	e6c78a98-f45b-482b-a551-4f11b8c1ff8b	3	128748321	128753404	10	5083	NA	10	NA	NA	NA	NA
Bladder-TCC	e6c78a98-f45b-482b-a551-4f11b8c1ff8b	4	134115225	134122094	11	6869	1	6	4	NA	NA	NA
Bladder-TCC	e6c78a98-f45b-482b-a551-4f11b8c1ff8b	4	148703741	148710235	11	6494	NA	3	8	NA	NA	NA
Bladder-TCC	e6c78a98-f45b-482b-a551-4f11b8c1ff8b	5	39809849	39811380	6	1531	1	3	1	NA	NA	1
Bladder-TCC	e6c78a98-f45b-482b-a551-4f11b8c1ff8b	6	157027028	157029571	6	2543	1	1	4	NA	NA	NA
Bladder-TCC	e6c78a98-f45b-482b-a551-4f11b8c1ff8b	7	146583822	146584031	7	209	1	2	4	NA	NA	NA
Bladder-TCC	e6c78a98-f45b-482b-a551-4f11b8c1ff8b	8	24794705	24795792	8	1087	1	4	3	NA	NA	NA
Bladder-TCC	e6c78a98-f45b-482b-a551-4f11b8c1ff8b	9	19248504	19252776	7	4272	1	4	2	NA	NA	NA
Bladder-TCC	e6c78a98-f45b-482b-a551-4f11b8c1ff8b	9	133497184	133498981	11	1797	1	4	6	NA	NA	NA
Bladder-TCC	e6c78a98-f45b-482b-a551-4f11b8c1ff8b	10	9154398	9156581	7	2183	NA	6	1	NA	NA	NA
Bladder-TCC	e6c78a98-f45b-482b-a551-4f11b8c1ff8b	12	110409411	110412264	7	2853	NA	6	1	NA	NA	NA
Bladder-TCC	e6c78a98-f45b-482b-a551-4f11b8c1ff8b	13	41717532	41722433	8	4901	1	4	3	NA	NA	NA
Bladder-TCC	e6c78a98-f45b-482b-a551-4f11b8c1ff8b	13	99082799	99086442	9	3643	2	5	2	NA	NA	NA
Bladder-TCC	e6c78a98-f45b-482b-a551-4f11b8c1ff8b	14	40356576	40360572	8	3996	1	5	2	NA	NA	NA
Bladder-TCC	e6c78a98-f45b-482b-a551-4f11b8c1ff8b	14	90165460	90167183	7	1723	1	4	2	NA	NA	NA
Bladder-TCC	e6c78a98-f45b-482b-a551-4f11b8c1ff8b	16	4268218	4269744	7	1526	4	2	1	NA	NA	NA
Bladder-TCC	e6c78a98-f45b-482b-a551-4f11b8c1ff8b	17	18080550	18082089	6	1539	1	2	3	NA	NA	NA
Bladder-TCC	e6c78a98-f45b-482b-a551-4f11b8c1ff8b	17	45193169	45196685	12	3516	1	6	5	NA	NA	NA
Bladder-TCC	e6c78a98-f45b-482b-a551-4f11b8c1ff8b	19	20765343	20767583	9	2240	NA	7	2	NA	NA	NA
Bladder-TCC	e6c78a98-f45b-482b-a551-4f11b8c1ff8b	20	32149100	32152723	6	3623	NA	6	NA	NA	NA	NA
Bladder-TCC	e6c78a98-f45b-482b-a551-4f11b8c1ff8b	20	62279973	62281611	8	1638	NA	7	1	NA	NA	NA
Bladder-TCC	e6c78a98-f45b-482b-a551-4f11b8c1ff8b	22	20797832	20803905	13	6073	NA	4	9	NA	NA	NA
Panc-AdenoCA	e6eda5db-4d4f-418e-b0d4-ed9b3e5259d3	6	66954160	66955710	7	1550	NA	5	2	NA	NA	NA
Ovary-AdenoCA	e6f7344b-951f-4f3f-b140-7bf53164c462	1	96312253	96316807	6	4554	2	3	1	NA	NA	NA
Ovary-AdenoCA	e6f7344b-951f-4f3f-b140-7bf53164c462	3	104940013	104944672	6	4659	NA	4	2	NA	NA	NA
Ovary-AdenoCA	e6f7344b-951f-4f3f-b140-7bf53164c462	8	48444836	48447626	9	2790	4	4	1	NA	NA	NA
Ovary-AdenoCA	e6f7344b-951f-4f3f-b140-7bf53164c462	10	114357875	114376148	33	18273	NA	19	14	NA	NA	NA
Ovary-AdenoCA	e6f7344b-951f-4f3f-b140-7bf53164c462	11	105825256	105825610	10	354	NA	1	9	NA	NA	NA
Ovary-AdenoCA	e6f7344b-951f-4f3f-b140-7bf53164c462	12	98691669	98694695	14	3026	NA	11	3	NA	NA	NA
Ovary-AdenoCA	e6f7344b-951f-4f3f-b140-7bf53164c462	18	20093420	20093910	6	490	1	3	2	NA	NA	NA
Panc-AdenoCA	e7048a93-a1ed-4cdc-a1ab-fa507408ca77	8	66113440	66113679	13	239	3	2	8	NA	NA	NA
Panc-AdenoCA	e7048a93-a1ed-4cdc-a1ab-fa507408ca77	9	28697591	28700902	6	3311	NA	2	4	NA	NA	NA
Panc-AdenoCA	e7048a93-a1ed-4cdc-a1ab-fa507408ca77	18	21743804	21744037	6	233	NA	3	3	NA	NA	NA
Panc-AdenoCA	e7048a93-a1ed-4cdc-a1ab-fa507408ca77	18	22155717	22156967	13	1250	2	3	8	NA	NA	NA
Liver-HCC	e75fbf08-c622-11e3-bf01-24c6515278c0	4	22778483	22781527	9	3044	NA	5	2	NA	2	NA
Liver-HCC	e75fbf08-c622-11e3-bf01-24c6515278c0	20	50106928	50107711	7	783	NA	2	5	NA	NA	NA
Panc-AdenoCA	e7603fc8-9b73-4c17-86f9-46ca1bceeb1d	7	19738199	19739629	13	1430	NA	5	8	NA	NA	NA
Panc-AdenoCA	e7603fc8-9b73-4c17-86f9-46ca1bceeb1d	9	20516934	20517535	6	601	1	1	4	NA	NA	NA
Panc-AdenoCA	e7603fc8-9b73-4c17-86f9-46ca1bceeb1d	9	20909251	20911813	20	2562	1	7	12	NA	NA	NA
Panc-AdenoCA	e7603fc8-9b73-4c17-86f9-46ca1bceeb1d	9	21009625	21013049	15	3424	1	6	8	NA	NA	NA

Eso-AdenoCa	e7a0889c-dd8c-4230-9c63-384f1f6ee577	2	160733902	160734982	6	1080	NA	4	2	NA	NA	NA
Biliary-AdenoCA	e7c90af0-4b03-453e-b44f-e07248415088	4	114697943	114698687	6	744	3	1	2	NA	NA	NA
Biliary-AdenoCA	e7c90af0-4b03-453e-b44f-e07248415088	7	54890507	54905892	34	15385	2	12	20	NA	NA	NA
Biliary-AdenoCA	e7c90af0-4b03-453e-b44f-e07248415088	10	92452406	92453290	6	884	1	2	3	NA	NA	NA
Biliary-AdenoCA	e7c90af0-4b03-453e-b44f-e07248415088	11	41126983	41128399	7	1416	NA	1	3	NA	2	1
Biliary-AdenoCA	e7c90af0-4b03-453e-b44f-e07248415088	17	37010928	37012524	12	1596	2	6	4	NA	NA	NA
Biliary-AdenoCA	e7c90af0-4b03-453e-b44f-e07248415088	19	51566711	51566952	7	241	NA	3	4	NA	NA	NA
Biliary-AdenoCA	e7c90af0-4b03-453e-b44f-e07248415088	19	56564955	56565806	9	851	1	5	3	NA	NA	NA
Uterus-AdenoCA	e7d74d34-3255-4c20-90fd-b105e6e229c8	1	153480595	153482899	6	2304	3	1	2	NA	NA	NA
Uterus-AdenoCA	e7d74d34-3255-4c20-90fd-b105e6e229c8	1	201300707	201308222	22	7515	6	8	8	NA	NA	NA
Uterus-AdenoCA	e7d74d34-3255-4c20-90fd-b105e6e229c8	1	201720869	201725514	7	4645	3	1	3	NA	NA	NA
Uterus-AdenoCA	e7d74d34-3255-4c20-90fd-b105e6e229c8	3	169055726	169059328	6	3602	NA	3	3	NA	NA	NA
Uterus-AdenoCA	e7d74d34-3255-4c20-90fd-b105e6e229c8	5	17278993	17305771	54	26778	10	4	37	NA	NA	3
Uterus-AdenoCA	e7d74d34-3255-4c20-90fd-b105e6e229c8	7	55702346	55705229	7	2883	NA	1	6	NA	NA	NA
Lung-SCC	e7ebc6fb-0926-4c8a-a67b-0c6b9c1ffaba	1	157007338	157009562	6	2224	NA	NA	6	NA	NA	NA
Lung-SCC	e7ebc6fb-0926-4c8a-a67b-0c6b9c1ffaba	2	241189471	241194764	12	5293	NA	6	6	NA	NA	NA
Lung-SCC	e7ebc6fb-0926-4c8a-a67b-0c6b9c1ffaba	3	175801773	175805046	32	3273	NA	3	29	NA	NA	NA
Lung-SCC	e7ebc6fb-0926-4c8a-a67b-0c6b9c1ffaba	3	177231674	177233402	8	1728	NA	3	5	NA	NA	NA
Lung-SCC	e7ebc6fb-0926-4c8a-a67b-0c6b9c1ffaba	3	179537218	179538998	12	1780	NA	NA	12	NA	NA	NA
Lung-SCC	e7ebc6fb-0926-4c8a-a67b-0c6b9c1ffaba	3	189642434	189655229	17	12795	NA	8	9	NA	NA	NA
Lung-SCC	e7ebc6fb-0926-4c8a-a67b-0c6b9c1ffaba	6	33604802	33606464	7	1662	NA	2	5	NA	NA	NA
Lung-SCC	e7ebc6fb-0926-4c8a-a67b-0c6b9c1ffaba	6	56337156	56343289	31	6133	4	13	14	NA	NA	NA
Lung-SCC	e7ebc6fb-0926-4c8a-a67b-0c6b9c1ffaba	7	2616002	2616777	6	775	NA	2	4	NA	NA	NA
Lung-SCC	e7ebc6fb-0926-4c8a-a67b-0c6b9c1ffaba	8	102058019	102062159	9	4140	NA	1	8	NA	NA	NA
Lung-SCC	e7ebc6fb-0926-4c8a-a67b-0c6b9c1ffaba	9	774910	775762	7	852	NA	2	5	NA	NA	NA
Lung-SCC	e7ebc6fb-0926-4c8a-a67b-0c6b9c1ffaba	9	35757500	35760150	7	2650	2	4	1	NA	NA	NA
Lung-SCC	e7ebc6fb-0926-4c8a-a67b-0c6b9c1ffaba	9	36685404	36700130	18	14726	2	6	10	NA	NA	NA
Lung-SCC	e7ebc6fb-0926-4c8a-a67b-0c6b9c1ffaba	10	77887483	77888012	6	529	1	3	2	NA	NA	NA
Lung-SCC	e7ebc6fb-0926-4c8a-a67b-0c6b9c1ffaba	11	124218513	124231169	29	12656	NA	15	14	NA	NA	NA
Lung-SCC	e7ebc6fb-0926-4c8a-a67b-0c6b9c1ffaba	11	125259128	125260724	14	1596	4	7	3	NA	NA	NA
Lung-SCC	e7ebc6fb-0926-4c8a-a67b-0c6b9c1ffaba	12	123512249	123512954	6	705	2	1	3	NA	NA	NA
Lung-SCC	e7ebc6fb-0926-4c8a-a67b-0c6b9c1ffaba	13	25463867	25466876	6	3009	NA	5	1	NA	NA	NA
Lung-SCC	e7ebc6fb-0926-4c8a-a67b-0c6b9c1ffaba	13	48554045	48555210	8	1165	1	3	4	NA	NA	NA
Lung-SCC	e7ebc6fb-0926-4c8a-a67b-0c6b9c1ffaba	15	31861766	31863148	6	1382	NA	3	3	NA	NA	NA
Lung-SCC	e7ebc6fb-0926-4c8a-a67b-0c6b9c1ffaba	16	8962896	8965924	7	3028	2	1	4	NA	NA	NA
Lung-SCC	e7ebc6fb-0926-4c8a-a67b-0c6b9c1ffaba	16	30935746	30937306	10	1560	1	5	4	NA	NA	NA
Lung-SCC	e7ebc6fb-0926-4c8a-a67b-0c6b9c1ffaba	17	71107062	71108510	6	1448	3	NA	3	NA	NA	NA
Lung-SCC	e7ebc6fb-0926-4c8a-a67b-0c6b9c1ffaba	19	16820237	16824957	11	4720	1	3	7	NA	NA	NA
Lung-SCC	e7ebc6fb-0926-4c8a-a67b-0c6b9c1ffaba	20	47834479	47836128	6	1649	2	1	3	NA	NA	NA
Breast-AdenoCa	e8392f20-c9e3-4649-b64f-5b266c393a76	11	93274690	93280525	20	5835	2	10	7	1	NA	NA
Lymph-BNHL	e84e0649-a2e8-4873-9cb6-1aa65601ae3a	1	82007298	82014471	9	7173	3	NA	1	4	NA	1
Lymph-BNHL	e84e0649-a2e8-4873-9cb6-1aa65601ae3a	1	106270366	106275602	12	5236	1	NA	2	3	2	4
Lymph-BNHL	e84e0649-a2e8-4873-9cb6-1aa65601ae3a	1	248088813	248091888	7	3075	2	NA	2	1	NA	2
Lymph-BNHL	e84e0649-a2e8-4873-9cb6-1aa65601ae3a	4	23029754	23030441	7	687	NA	NA	1	3	1	2
Lymph-BNHL	e84e0649-a2e8-4873-9cb6-1aa65601ae3a	4	58974876	58981567	8	6691	NA	1	2	1	2	2
Lymph-BNHL	e84e0649-a2e8-4873-9cb6-1aa65601ae3a	4	64169582	64170842	7	1260	2	NA	NA	1	1	3
Lymph-BNHL	e84e0649-a2e8-4873-9cb6-1aa65601ae3a	4	96854051	96866570	17	12519	1	3	3	3	1	6
Lymph-BNHL	e84e0649-a2e8-4873-9cb6-1aa65601ae3a	4	127371754	127378740	12	6986	3	1	2	2	NA	4
Lymph-BNHL	e84e0649-a2e8-4873-9cb6-1aa65601ae3a	4	143065875	143076046	15	10171	NA	NA	4	4	1	6
Lymph-BNHL	e84e0649-a2e8-4873-9cb6-1aa65601ae3a	4	173037103	173040783	7	3680	1	1	1	NA	2	2
Lymph-BNHL	e84e0649-a2e8-4873-9cb6-1aa65601ae3a	5	21400750	21405095	9	4345	2	1	1	3	NA	2
Lymph-BNHL	e84e0649-a2e8-4873-9cb6-1aa65601ae3a	5	23455449	23461035	7	5586	NA	NA	1	2	1	3
Lymph-BNHL	e84e0649-a2e8-4873-9cb6-1aa65601ae3a	6	391851	398809	81	6958	7	29	23	10	8	4
Lymph-BNHL	e84e0649-a2e8-4873-9cb6-1aa65601ae3a	6	49255411	49261263	8	5852	1	NA	2	2	NA	3
Lymph-BNHL	e84e0649-a2e8-4873-9cb6-1aa65601ae3a	6	102864572	102873001	13	8429	1	4	NA	1	2	5
Lymph-BNHL	e84e0649-a2e8-4873-9cb6-1aa65601ae3a	7	88729895	88731537	6	1642	NA	NA	NA	NA	4	2
Lymph-BNHL	e84e0649-a2e8-4873-9cb6-1aa65601ae3a	8	84739972	84743402	6	3430	2	NA	NA	2	1	1
Lymph-BNHL	e84e0649-a2e8-4873-9cb6-1aa65601ae3a	11	79259979	79261777	6	1798	NA	NA	NA	1	NA	5
Lymph-BNHL	e84e0649-a2e8-4873-9cb6-1aa65601ae3a	11	89234086	89236328	7	2242	1	NA	NA	5	NA	1
Lymph-BNHL	e84e0649-a2e8-4873-9cb6-1aa65601ae3a	13	65864841	65868941	6	4100	2	NA	1	1	1	1

Lymph-BNHL	e84e0649-a2e8-4873-9cb6-1aa65601ae3a	13	67446441	67448583	7	2142	2	NA	1	NA	2	2
Lymph-BNHL	e84e0649-a2e8-4873-9cb6-1aa65601ae3a	13	85640138	85645780	7	5642	3	NA	1	1	2	NA
Lymph-BNHL	e84e0649-a2e8-4873-9cb6-1aa65601ae3a	13	89576150	89582983	11	6833	1	1	NA	2	4	3
Lymph-BNHL	e84e0649-a2e8-4873-9cb6-1aa65601ae3a	14	43099774	43103340	6	3566	2	NA	NA	1	1	2
Lymph-BNHL	e84e0649-a2e8-4873-9cb6-1aa65601ae3a	14	106068880	106072009	24	3129	4	6	7	2	3	2
Lymph-BNHL	e84e0649-a2e8-4873-9cb6-1aa65601ae3a	14	106112882	106137538	26	24656	3	7	9	4	1	2
Lymph-BNHL	e84e0649-a2e8-4873-9cb6-1aa65601ae3a	14	106173741	106176911	30	3170	3	5	6	7	4	5
Lymph-BNHL	e84e0649-a2e8-4873-9cb6-1aa65601ae3a	14	106358187	106367498	18	9311	NA	3	5	4	5	1
Lymph-BNHL	e84e0649-a2e8-4873-9cb6-1aa65601ae3a	14	106641827	106642769	31	942	3	5	4	5	6	8
Lymph-BNHL	e84e0649-a2e8-4873-9cb6-1aa65601ae3a	14	107178916	107179052	7	136	1	1	4	NA	1	NA
Lymph-BNHL	e84e0649-a2e8-4873-9cb6-1aa65601ae3a	17	36042994	36043107	6	113	NA	NA	NA	1	2	3
Lymph-BNHL	e84e0649-a2e8-4873-9cb6-1aa65601ae3a	20	12478955	12481700	6	2745	NA	NA	3	NA	1	2
Lymph-BNHL	e84e0649-a2e8-4873-9cb6-1aa65601ae3a	22	23198076	23265021	90	66945	13	16	35	6	6	14
Lymph-BNHL	e84e0649-a2e8-4873-9cb6-1aa65601ae3a	X	28488571	28490989	6	2418	2	NA	1	NA	NA	3
Lymph-BNHL	e84e0649-a2e8-4873-9cb6-1aa65601ae3a	X	82163571	82164959	7	1388	1	NA	1	1	1	3
Lymph-BNHL	e84e0649-a2e8-4873-9cb6-1aa65601ae3a	X	86377490	86386243	16	8753	3	NA	1	3	2	7
Lymph-BNHL	e84e0649-a2e8-4873-9cb6-1aa65601ae3a	X	86740083	86745202	9	5119	2	2	NA	1	3	1
Lymph-BNHL	e84e0649-a2e8-4873-9cb6-1aa65601ae3a	X	87906423	87913774	9	7351	1	2	1	1	1	3
Lymph-BNHL	e84e0649-a2e8-4873-9cb6-1aa65601ae3a	X	87988342	87994316	8	5974	2	NA	1	1	1	3
Lymph-BNHL	e84e0649-a2e8-4873-9cb6-1aa65601ae3a	X	88011957	88016754	8	4797	4	NA	2	NA	NA	2
Lymph-BNHL	e84e0649-a2e8-4873-9cb6-1aa65601ae3a	X	94944652	94949158	7	4506	1	3	NA	2	NA	1
Lymph-BNHL	e89e9c69-ffcd-4a4c-818d-1dee43ddc76a	2	89156354	89161381	59	5027	3	7	30	11	5	3
Lymph-BNHL	e89e9c69-ffcd-4a4c-818d-1dee43ddc76a	2	89246834	89247574	8	740	1	1	4	2	NA	NA
Lymph-BNHL	e89e9c69-ffcd-4a4c-818d-1dee43ddc76a	9	37025236	37027190	13	1954	NA	4	9	NA	NA	NA
Lymph-BNHL	e89e9c69-ffcd-4a4c-818d-1dee43ddc76a	14	106240221	106241546	29	1325	2	1	26	NA	NA	NA
Lymph-BNHL	e89e9c69-ffcd-4a4c-818d-1dee43ddc76a	14	106321869	106389316	83	67447	6	8	64	4	1	NA
Lymph-BNHL	e89e9c69-ffcd-4a4c-818d-1dee43ddc76a	14	106518459	106518792	10	333	NA	1	8	NA	1	NA
Lymph-BNHL	e89e9c69-ffcd-4a4c-818d-1dee43ddc76a	16	19554812	19558789	15	3977	NA	9	4	NA	1	1
Lymph-BNHL	e89e9c69-ffcd-4a4c-818d-1dee43ddc76a	16	30566887	30570221	8	3334	1	1	2	3	NA	1
Lymph-BNHL	e89e9c69-ffcd-4a4c-818d-1dee43ddc76a	22	23230463	23236231	30	5768	2	4	20	3	1	NA
Kidney-ChRCC	e9032df3-8692-4146-a867-b9b64c9b310a	3	28206436	28207497	11	1061	4	NA	7	NA	NA	NA
Kidney-ChRCC	e9032df3-8692-4146-a867-b9b64c9b310a	3	29181860	29185502	13	3642	NA	4	9	NA	NA	NA
Kidney-ChRCC	e9032df3-8692-4146-a867-b9b64c9b310a	3	29993713	29995075	11	1362	1	2	8	NA	NA	NA
Kidney-ChRCC	e9032df3-8692-4146-a867-b9b64c9b310a	3	30121291	30121796	7	505	NA	2	5	NA	NA	NA
Kidney-ChRCC	e9032df3-8692-4146-a867-b9b64c9b310a	3	30697074	30698798	11	1724	1	2	8	NA	NA	NA
Kidney-ChRCC	e9032df3-8692-4146-a867-b9b64c9b310a	3	30865059	30875422	53	10363	2	16	35	NA	NA	NA
Kidney-ChRCC	e9032df3-8692-4146-a867-b9b64c9b310a	3	32131442	32136470	15	5028	NA	2	13	NA	NA	NA
Kidney-ChRCC	e9032df3-8692-4146-a867-b9b64c9b310a	3	47990583	47994846	8	4263	NA	2	6	NA	NA	NA
Kidney-ChRCC	e9032df3-8692-4146-a867-b9b64c9b310a	8	50987912	50988477	11	565	NA	5	6	NA	NA	NA
Kidney-ChRCC	e9032df3-8692-4146-a867-b9b64c9b310a	8	70154323	70161867	24	7544	1	7	16	NA	NA	NA
Kidney-ChRCC	e9032df3-8692-4146-a867-b9b64c9b310a	8	102950772	102951228	9	456	1	3	5	NA	NA	NA
Kidney-ChRCC	e9032df3-8692-4146-a867-b9b64c9b310a	8	105510296	105512480	10	2184	NA	4	6	NA	NA	NA
Kidney-ChRCC	e9032df3-8692-4146-a867-b9b64c9b310a	12	27259716	27266028	15	6312	NA	NA	15	NA	NA	NA
Kidney-ChRCC	e9032df3-8692-4146-a867-b9b64c9b310a	15	43495595	43497091	10	1496	NA	1	9	NA	NA	NA
Kidney-ChRCC	e9032df3-8692-4146-a867-b9b64c9b310a	15	49178004	49183790	7	5786	NA	NA	7	NA	NA	NA
Kidney-ChRCC	e9032df3-8692-4146-a867-b9b64c9b310a	15	55068113	55068456	7	343	NA	4	3	NA	NA	NA
Kidney-ChRCC	e9032df3-8692-4146-a867-b9b64c9b310a	15	55122413	55125268	8	2855	NA	NA	8	NA	NA	NA
Kidney-ChRCC	e9032df3-8692-4146-a867-b9b64c9b310a	15	56639233	56639440	7	207	NA	NA	7	NA	NA	NA
Kidney-ChRCC	e9032df3-8692-4146-a867-b9b64c9b310a	15	66532915	66533522	10	607	NA	2	8	NA	NA	NA
Kidney-ChRCC	e9032df3-8692-4146-a867-b9b64c9b310a	15	95527438	95530480	20	3042	1	11	8	NA	NA	NA
Eso-AdenoCa	e93b0979-65ef-4883-9b6e-39eb17966e66	2	31675481	31678717	8	3236	3	3	2	NA	NA	NA
Eso-AdenoCa	e93b0979-65ef-4883-9b6e-39eb17966e66	8	127422442	127423933	7	1491	2	3	2	NA	NA	NA
Panc-AdenoCA	e97ea3d7-9fcd-4df1-9ea8-587cb3be75ec	9	21927618	21928036	6	418	NA	4	2	NA	NA	NA
Panc-AdenoCA	e97ea3d7-9fcd-4df1-9ea8-587cb3be75ec	10	86758533	86761163	17	2630	1	13	3	NA	NA	NA
Lymph-BNHL	e98e4e23-a139-4fff-b017-35f3f976eddd	2	89160666	89161299	12	633	NA	NA	3	6	1	2
Lymph-BNHL	e98e4e23-a139-4fff-b017-35f3f976eddd	4	181846842	181851186	9	4344	NA	NA	1	7	NA	1
Lymph-BNHL	e98e4e23-a139-4fff-b017-35f3f976eddd	5	99887494	99890026	7	2532	NA	1	NA	5	1	NA
Lymph-BNHL	e98e4e23-a139-4fff-b017-35f3f976eddd	7	81154638	81160378	7	5740	1	NA	2	3	NA	1
Lymph-BNHL	e98e4e23-a139-4fff-b017-35f3f976eddd	13	64112729	64119943	9	7214	NA	NA	1	5	1	2
Lymph-BNHL	e98e4e23-a139-4fff-b017-35f3f976eddd	14	106208869	106214726	15	5857	1	1	10	2	1	NA

Lymph-BNHL	e98e4e23-a139-4fff-b017-35f3f976eddd	14	106326543	106330267	57	3724	2	13	19	13	4	6
Lymph-BNHL	e98e4e23-a139-4fff-b017-35f3f976eddd	14	106518678	106519610	9	932	1	1	5	NA	NA	2
Lymph-BNHL	e98e4e23-a139-4fff-b017-35f3f976eddd	18	60984962	60988779	15	3817	NA	4	3	2	4	2
Lymph-BNHL	e98e4e23-a139-4fff-b017-35f3f976eddd	22	23192289	23192792	9	503	NA	4	1	2	1	1
Lymph-BNHL	e98e4e23-a139-4fff-b017-35f3f976eddd	22	23230583	23231848	11	1265	NA	1	4	4	NA	2
Ovary-AdenoCA	e9d98643-01ee-40c3-a617-e004559625cd	12	70133917	70134999	6	1082	1	3	2	NA	NA	NA
Ovary-AdenoCA	e9d98643-01ee-40c3-a617-e004559625cd	18	4613979	4615636	13	1657	1	7	5	NA	NA	NA
Stomach-AdenoCA	ea2055d1-7b8b-4aaf-9a70-8802f4deef9b	21	20430349	20431046	7	697	4	NA	3	NA	NA	NA
Breast-AdenoCa	ea43434b-197e-48ac-ae2e-46bc7f3776de	8	43297017	43299202	8	2185	2	3	3	NA	NA	NA
Breast-AdenoCa	ea43434b-197e-48ac-ae2e-46bc7f3776de	8	124694612	124695949	7	1337	2	2	3	NA	NA	NA
Uterus-AdenoCA	ea6efcd1-11de-45f0-8bde-f1c06ad27e79	5	10247487	10247754	6	267	1	1	4	NA	NA	NA
Uterus-AdenoCA	ea6efcd1-11de-45f0-8bde-f1c06ad27e79	7	94548806	94552795	8	3989	2	5	1	NA	NA	NA
Uterus-AdenoCA	ea6efcd1-11de-45f0-8bde-f1c06ad27e79	8	43493284	43497749	12	4465	2	7	NA	3	NA	NA
Uterus-AdenoCA	ea6efcd1-11de-45f0-8bde-f1c06ad27e79	9	3606219	3607105	9	886	2	5	2	NA	NA	NA
Uterus-AdenoCA	ea6efcd1-11de-45f0-8bde-f1c06ad27e79	15	47782302	47783113	7	811	2	2	3	NA	NA	NA
Uterus-AdenoCA	ea6efcd1-11de-45f0-8bde-f1c06ad27e79	15	47799142	47801050	8	1908	NA	4	4	NA	NA	NA
CNS-GBM	eab71331-53d3-4cf5-96c9-5121a3962c27	X	148801613	148803789	9	2176	2	5	2	NA	NA	NA
CNS-GBM	eab71331-53d3-4cf5-96c9-5121a3962c27	X	148978519	148981726	8	3207	6	NA	2	NA	NA	NA
Skin-Melanoma	ead1bc2e-42c3-46d9-8f6f-8cb16ba9d2c3	3	26692009	26696090	17	4081	NA	4	13	NA	NA	NA
Skin-Melanoma	ead1bc2e-42c3-46d9-8f6f-8cb16ba9d2c3	5	15057302	15064251	9	6949	1	5	1	NA	1	1
Skin-Melanoma	ead1bc2e-42c3-46d9-8f6f-8cb16ba9d2c3	5	17843354	17844846	21	1492	1	3	17	NA	NA	NA
Skin-Melanoma	ead1bc2e-42c3-46d9-8f6f-8cb16ba9d2c3	5	37308115	37309735	8	1620	1	NA	7	NA	NA	NA
Skin-Melanoma	ead1bc2e-42c3-46d9-8f6f-8cb16ba9d2c3	7	125734166	125738797	20	4631	5	2	13	NA	NA	NA
Skin-Melanoma	ead1bc2e-42c3-46d9-8f6f-8cb16ba9d2c3	7	149508540	149510134	10	1594	2	1	7	NA	NA	NA
Skin-Melanoma	ead1bc2e-42c3-46d9-8f6f-8cb16ba9d2c3	11	59767868	59768338	6	470	1	1	4	NA	NA	NA
Skin-Melanoma	ead1bc2e-42c3-46d9-8f6f-8cb16ba9d2c3	11	81236149	81236640	8	491	NA	NA	8	NA	NA	NA
Skin-Melanoma	ead1bc2e-42c3-46d9-8f6f-8cb16ba9d2c3	14	91353033	91353589	6	556	NA	1	5	NA	NA	NA
Eso-AdenoCa	eb1531b0-8af1-4b2b-9192-644cb47101fc	7	54664721	54670197	9	5476	2	1	6	NA	NA	NA
Eso-AdenoCa	eb1531b0-8af1-4b2b-9192-644cb47101fc	9	127611107	127614492	6	3385	NA	3	3	NA	NA	NA
Eso-AdenoCa	eb1531b0-8af1-4b2b-9192-644cb47101fc	10	68161583	68164404	8	2821	2	NA	NA	4	2	NA
Eso-AdenoCa	eb1531b0-8af1-4b2b-9192-644cb47101fc	11	118357419	118357813	7	394	1	NA	6	NA	NA	NA
Eso-AdenoCa	eb1531b0-8af1-4b2b-9192-644cb47101fc	17	75357536	75361200	7	3664	1	1	4	1	NA	NA
Liver-HCC	eb1792ee-6946-4e0d-bdd1-c924328dba5d	1	111020321	111020676	6	355	2	3	1	NA	NA	NA
Liver-HCC	eb1792ee-6946-4e0d-bdd1-c924328dba5d	8	47539451	47541045	7	1594	NA	NA	2	NA	4	1
Stomach-AdenoCA	eb18e71d-2a27-4eae-aa27-4b2c0716d2ba	2	186446057	186449053	13	2996	2	6	5	NA	NA	NA
Stomach-AdenoCA	eb59b79f-bd36-4bf4-83e3-2af73bd1d30a	1	26791328	26792907	6	1579	NA	4	2	NA	NA	NA
Stomach-AdenoCA	eb59b79f-bd36-4bf4-83e3-2af73bd1d30a	17	37841283	37843579	7	2296	2	4	1	NA	NA	NA
Skin-Melanoma	eb9a8f12-6451-43cc-95b3-2e86cf704c96	1	149923924	149926457	6	2533	1	5	NA	NA	NA	NA
Skin-Melanoma	eb9a8f12-6451-43cc-95b3-2e86cf704c96	2	122542471	122548444	15	5973	2	6	6	NA	NA	1
Skin-Melanoma	eb9a8f12-6451-43cc-95b3-2e86cf704c96	2	122706615	122712593	20	5978	2	11	7	NA	NA	NA
Skin-Melanoma	eb9a8f12-6451-43cc-95b3-2e86cf704c96	2	122748494	122750787	8	2293	NA	5	3	NA	NA	NA
Skin-Melanoma	eb9a8f12-6451-43cc-95b3-2e86cf704c96	3	18217041	18217517	10	476	NA	2	8	NA	NA	NA
Skin-Melanoma	eb9a8f12-6451-43cc-95b3-2e86cf704c96	4	173063751	173068212	9	4461	NA	1	8	NA	NA	NA
Lymph-CLL	ebc1a26b-9582-4756-acd5-b02d1152319d	2	89159924	89185561	46	25637	2	11	7	12	9	5
Lymph-CLL	ebc1a26b-9582-4756-acd5-b02d1152319d	5	27140248	27144288	6	4040	NA	1	1	NA	3	1
Lymph-CLL	ebc1a26b-9582-4756-acd5-b02d1152319d	14	106325473	106330138	20	4665	1	8	6	4	1	NA
Lymph-CLL	ebc1a26b-9582-4756-acd5-b02d1152319d	14	106452863	106454271	7	1408	NA	NA	3	2	2	NA
Lymph-CLL	ebc1a26b-9582-4756-acd5-b02d1152319d	22	23223211	23227946	7	4735	1	NA	2	4	NA	NA
Lymph-BNHL	ebe0ed67-2d3f-45cd-8f9b-4912595b16a0	1	12442645	12442744	6	99	NA	NA	NA	3	2	1
Lymph-BNHL	ebe0ed67-2d3f-45cd-8f9b-4912595b16a0	2	82567704	82569071	6	1367	NA	NA	2	NA	1	3
Lymph-BNHL	ebe0ed67-2d3f-45cd-8f9b-4912595b16a0	2	89156697	89159429	12	2732	NA	1	1	3	1	6
Lymph-BNHL	ebe0ed67-2d3f-45cd-8f9b-4912595b16a0	3	187461318	187464138	26	2820	2	5	6	7	1	5
Lymph-BNHL	ebe0ed67-2d3f-45cd-8f9b-4912595b16a0	4	161383537	161386979	6	3442	NA	NA	1	NA	2	3
Lymph-BNHL	ebe0ed67-2d3f-45cd-8f9b-4912595b16a0	6	103492515	103496355	8	3840	2	NA	1	4	NA	1
Lymph-BNHL	ebe0ed67-2d3f-45cd-8f9b-4912595b16a0	8	103243232	103243560	6	328	NA	NA	NA	3	1	2
Lymph-BNHL	ebe0ed67-2d3f-45cd-8f9b-4912595b16a0	14	69256875	69259383	7	2508	1	1	3	NA	1	1
Lymph-BNHL	ebe0ed67-2d3f-45cd-8f9b-4912595b16a0	14	106176452	106178061	10	1609	NA	5	3	1	1	NA
Lymph-BNHL	ebe0ed67-2d3f-45cd-8f9b-4912595b16a0	14	106211933	106213914	11	1981	NA	3	6	1	1	NA
Lymph-BNHL	ebe0ed67-2d3f-45cd-8f9b-4912595b16a0	14	106323783	106330231	112	6448	8	29	37	17	11	10
Lymph-BNHL	ebe0ed67-2d3f-45cd-8f9b-4912595b16a0	14	106725391	106726055	12	664	NA	2	NA	3	4	3

Lymph-BNHL	ebe0ed67-2d3f-45cd-8f9b-4912595b16a0	18	60984920	60988825	25	3905	1	3	10	3	6	2
Lymph-BNHL	ebe0ed67-2d3f-45cd-8f9b-4912595b16a0	22	23229556	23231171	7	1615	NA	NA	7	NA	NA	NA
Prost-AdenoCA	ec16d1f5-f8a9-4c19-8cce-92f6ea9eb3f7	13	62777788	62778164	7	376	NA	1	6	NA	NA	NA
Prost-AdenoCA	ec16d1f5-f8a9-4c19-8cce-92f6ea9eb3f7	18	33528954	33533129	21	4175	NA	15	5	NA	NA	1
Biliary-AdenoCA	ec399861-7a56-4ffd-8619-700b6e3a4367	1	15188852	15192832	6	3980	NA	1	5	NA	NA	NA
Biliary-AdenoCA	ec399861-7a56-4ffd-8619-700b6e3a4367	1	115761856	115762831	7	975	NA	4	3	NA	NA	NA
Biliary-AdenoCA	ec399861-7a56-4ffd-8619-700b6e3a4367	2	210372844	210376393	7	3549	NA	5	2	NA	NA	NA
Biliary-AdenoCA	ec399861-7a56-4ffd-8619-700b6e3a4367	2	241099216	241100664	9	1448	NA	8	1	NA	NA	NA
Biliary-AdenoCA	ec399861-7a56-4ffd-8619-700b6e3a4367	5	142286556	142292431	7	5875	NA	1	6	NA	NA	NA
Biliary-AdenoCA	ec399861-7a56-4ffd-8619-700b6e3a4367	12	21303203	21305943	6	2740	2	1	3	NA	NA	NA
Biliary-AdenoCA	ec399861-7a56-4ffd-8619-700b6e3a4367	14	30932727	30937135	8	4408	NA	5	3	NA	NA	NA
Biliary-AdenoCA	ec399861-7a56-4ffd-8619-700b6e3a4367	14	31265115	31265370	9	255	NA	5	3	NA	NA	1
Biliary-AdenoCA	ec399861-7a56-4ffd-8619-700b6e3a4367	14	36925572	36927921	7	2349	3	NA	4	NA	NA	NA
Biliary-AdenoCA	ec399861-7a56-4ffd-8619-700b6e3a4367	19	41652781	41655014	9	2233	NA	2	7	NA	NA	NA
Biliary-AdenoCA	ec399861-7a56-4ffd-8619-700b6e3a4367	20	3239675	3243780	7	4105	2	4	1	NA	NA	NA
Eso-AdenoCa	ec474dfa-527c-44f3-9224-bf1c858cabfc	2	144920490	144925023	7	4533	NA	NA	NA	1	6	NA
Eso-AdenoCa	ec474dfa-527c-44f3-9224-bf1c858cabfc	10	57881095	57885104	6	4009	NA	NA	NA	2	2	2
Liver-HCC	ec5e2990-c622-11e3-bf01-24c6515278c0	17	20025956	20032993	12	7037	5	5	1	1	NA	NA
Panc-AdenoCA	ec77847e-48fd-4ba5-bc3e-3cd1b149b552	8	140122513	140122531	6	18	1	1	NA	2	NA	2
Panc-AdenoCA	ec77847e-48fd-4ba5-bc3e-3cd1b149b552	18	20279874	20281276	10	1402	3	4	3	NA	NA	NA
Panc-AdenoCA	ec77847e-48fd-4ba5-bc3e-3cd1b149b552	19	50375093	50375574	6	481	NA	NA	6	NA	NA	NA
Panc-AdenoCA	ecfe388a-99f5-44b9-9226-a6ae6d892538	6	67402363	67404637	9	2274	NA	6	2	1	NA	NA
Panc-AdenoCA	ecfe388a-99f5-44b9-9226-a6ae6d892538	6	67416823	67419191	8	2368	1	3	4	NA	NA	NA
Panc-AdenoCA	ecfe388a-99f5-44b9-9226-a6ae6d892538	10	43238011	43239031	11	1020	3	2	6	NA	NA	NA
Panc-AdenoCA	ecfe388a-99f5-44b9-9226-a6ae6d892538	10	43257321	43259699	29	2378	3	13	13	NA	NA	NA
Panc-AdenoCA	ecfe388a-99f5-44b9-9226-a6ae6d892538	14	31079534	31085851	17	6317	3	5	8	1	NA	NA
Panc-AdenoCA	ecfe388a-99f5-44b9-9226-a6ae6d892538	17	66248882	66250314	7	1432	2	2	3	NA	NA	NA
Panc-AdenoCA	ecfe388a-99f5-44b9-9226-a6ae6d892538	17	66298895	66300028	12	1133	1	2	9	NA	NA	NA
Panc-AdenoCA	ecfe388a-99f5-44b9-9226-a6ae6d892538	18	48566948	48569895	16	2947	3	9	4	NA	NA	NA
Uterus-AdenoCA	ed32c725-08ae-48eb-8fa2-719b9aeb7550	8	37994836	38007660	17	12824	7	4	5	1	NA	NA
Uterus-AdenoCA	ed32c725-08ae-48eb-8fa2-719b9aeb7550	8	68300244	68303583	9	3339	6	1	2	NA	NA	NA
Uterus-AdenoCA	ed32c725-08ae-48eb-8fa2-719b9aeb7550	12	46027758	46031520	6	3762	NA	6	NA	NA	NA	NA
Uterus-AdenoCA	ed32c725-08ae-48eb-8fa2-719b9aeb7550	15	37416600	37417397	9	797	NA	NA	NA	2	5	2
Uterus-AdenoCA	ed32c725-08ae-48eb-8fa2-719b9aeb7550	18	52364560	52369344	19	4784	5	12	2	NA	NA	NA
Uterus-AdenoCA	ed32c725-08ae-48eb-8fa2-719b9aeb7550	X	54085916	54091782	13	5866	NA	2	11	NA	NA	NA
Stomach-AdenoCA	eda1d8d9-e8f5-46f2-bb16-15d0b10c2cf9	2	124668479	124680040	13	11561	NA	NA	1	8	3	1
Stomach-AdenoCA	eda1d8d9-e8f5-46f2-bb16-15d0b10c2cf9	X	125451630	125458429	8	6799	NA	NA	2	5	NA	1
Panc-AdenoCA	edac1323-2497-45e6-9148-e9c955292ba2	4	134861812	134866105	11	4293	NA	7	4	NA	NA	NA
Panc-AdenoCA	edac1323-2497-45e6-9148-e9c955292ba2	5	115901037	115904022	24	2985	5	10	9	NA	NA	NA
Panc-AdenoCA	edac1323-2497-45e6-9148-e9c955292ba2	7	132251703	132252357	6	654	1	1	4	NA	NA	NA
Panc-AdenoCA	edac1323-2497-45e6-9148-e9c955292ba2	7	134335263	134340682	25	5419	4	4	17	NA	NA	NA
Panc-AdenoCA	edac1323-2497-45e6-9148-e9c955292ba2	8	11842651	11843902	16	1251	1	4	11	NA	NA	NA
Panc-AdenoCA	edac1323-2497-45e6-9148-e9c955292ba2	8	138855989	138868899	17	12910	2	4	9	1	1	NA
Panc-AdenoCA	edac1323-2497-45e6-9148-e9c955292ba2	13	70446410	70447274	6	864	NA	1	5	NA	NA	NA
Panc-AdenoCA	edac1323-2497-45e6-9148-e9c955292ba2	16	4289257	4291140	6	1883	1	1	4	NA	NA	NA
Panc-AdenoCA	edac1323-2497-45e6-9148-e9c955292ba2	16	5390889	5393496	15	2607	7	3	5	NA	NA	NA
Panc-AdenoCA	edac1323-2497-45e6-9148-e9c955292ba2	16	22840288	22847055	9	6767	2	2	5	NA	NA	NA
Panc-AdenoCA	edac1323-2497-45e6-9148-e9c955292ba2	16	23118483	23123639	10	5156	2	3	5	NA	NA	NA
Panc-AdenoCA	edac1323-2497-45e6-9148-e9c955292ba2	17	76762823	76766126	8	3303	NA	7	1	NA	NA	NA
Panc-AdenoCA	edac1323-2497-45e6-9148-e9c955292ba2	X	99980380	99981133	12	753	2	5	3	1	NA	1
Panc-AdenoCA	edac1323-2497-45e6-9148-e9c955292ba2	X	100138254	100138730	13	476	NA	7	6	NA	NA	NA
Stomach-AdenoCA	edc8839f-fa6b-41e0-ad49-f688073730bd	8	94594327	94599235	6	4908	NA	4	2	NA	NA	NA
Stomach-AdenoCA	edc8839f-fa6b-41e0-ad49-f688073730bd	21	25286400	25293725	10	7325	NA	NA	NA	2	2	6
Breast-AdenoCa	eddfbfb9-3ae8-449b-b870-46062a2571c1	3	127114478	127115577	12	1099	2	6	4	NA	NA	NA
Breast-AdenoCa	eddfbfb9-3ae8-449b-b870-46062a2571c1	5	85485558	85486865	10	1307	NA	5	5	NA	NA	NA
Breast-AdenoCa	eddfbfb9-3ae8-449b-b870-46062a2571c1	8	122174246	122179147	17	4901	3	10	3	NA	1	NA
Panc-AdenoCA	ee5d5e7d-78cf-4a29-a9ee-56aa3da877dd	1	203685453	203685883	6	430	1	2	3	NA	NA	NA
Panc-AdenoCA	ee5d5e7d-78cf-4a29-a9ee-56aa3da877dd	18	13550876	13552442	12	1566	2	3	7	NA	NA	NA
Lung-SCC	ee6cc68e-8d2a-41ee-82c6-0fecdf7e6259	11	41455057	41463408	11	8351	NA	NA	1	3	6	1
Lung-SCC	ee6cc68e-8d2a-41ee-82c6-0fecdf7e6259	X	63092958	63097221	9	4263	NA	8	1	NA	NA	NA

Lung-SCC	ee6cc68e-8d2a-41ee-82c6-0fecdf7e6259	X	102948803	102952158	7	3355	NA	NA	1	1	3	2
Panc-AdenoCA	ee8ba991-8c96-476e-b096-49f3aaaffc2f	1	209387236	209387671	6	435	NA	3	3	NA	NA	NA
Panc-AdenoCA	ee8ba991-8c96-476e-b096-49f3aaaffc2f	9	101465919	101466482	7	563	NA	NA	7	NA	NA	NA
Panc-AdenoCA	ee8ba991-8c96-476e-b096-49f3aaaffc2f	15	80216434	80218444	16	2010	4	7	5	NA	NA	NA
Panc-AdenoCA	ee8ba991-8c96-476e-b096-49f3aaaffc2f	18	1056884	1059361	7	2477	NA	3	4	NA	NA	NA
Head-SCC	ee963667-56b1-49bd-8896-e07b536d3014	7	87044172	87049160	11	4988	3	4	4	NA	NA	NA
Skin-Melanoma	eeddf701-93f9-4f10-85cb-9dce1760bae8	1	93296401	93299991	7	3590	NA	5	2	NA	NA	NA
Skin-Melanoma	eeddf701-93f9-4f10-85cb-9dce1760bae8	2	205474149	205482322	11	8173	1	7	3	NA	NA	NA
Skin-Melanoma	eeddf701-93f9-4f10-85cb-9dce1760bae8	6	142708141	142713115	8	4974	NA	5	3	NA	NA	NA
Skin-Melanoma	eeddf701-93f9-4f10-85cb-9dce1760bae8	9	37078226	37079541	15	1315	1	9	5	NA	NA	NA
Skin-Melanoma	eeddf701-93f9-4f10-85cb-9dce1760bae8	14	70953708	70955632	8	1924	NA	7	1	NA	NA	NA
Panc-AdenoCA	ef002c3d-358f-4fc0-98f3-2f8b89de4374	4	80346736	80348000	13	1264	2	3	8	NA	NA	NA
Panc-AdenoCA	ef002c3d-358f-4fc0-98f3-2f8b89de4374	4	80431381	80437676	29	6295	3	11	15	NA	NA	NA
Panc-AdenoCA	ef002c3d-358f-4fc0-98f3-2f8b89de4374	4	125188951	125189914	9	963	NA	5	4	NA	NA	NA
Panc-AdenoCA	ef002c3d-358f-4fc0-98f3-2f8b89de4374	10	109600831	109601442	6	611	NA	3	3	NA	NA	NA
Panc-AdenoCA	ef002c3d-358f-4fc0-98f3-2f8b89de4374	11	131945550	131949033	17	3483	1	10	6	NA	NA	NA
Panc-AdenoCA	ef002c3d-358f-4fc0-98f3-2f8b89de4374	19	54035840	54038672	7	2832	NA	4	3	NA	NA	NA
Panc-AdenoCA	ef002c3d-358f-4fc0-98f3-2f8b89de4374	21	11148714	11149862	6	1148	2	1	3	NA	NA	NA
Panc-AdenoCA	ef3a0ccd-85bc-4e5f-a8c6-5a2e2d9e6717	3	118868187	118870169	15	1982	NA	2	12	1	NA	NA
Panc-AdenoCA	ef3a0ccd-85bc-4e5f-a8c6-5a2e2d9e6717	13	78700158	78700398	7	240	1	2	4	NA	NA	NA
Panc-AdenoCA	ef3a0ccd-85bc-4e5f-a8c6-5a2e2d9e6717	18	30220054	30222976	17	2922	1	6	10	NA	NA	NA
Panc-AdenoCA	ef3a0ccd-85bc-4e5f-a8c6-5a2e2d9e6717	22	25330684	25331137	8	453	NA	6	2	NA	NA	NA
Head-SCC	ef673d3d-2031-4036-ba25-4bc7ef04075b	1	17203361	17203816	6	455	NA	1	5	NA	NA	NA
Head-SCC	ef673d3d-2031-4036-ba25-4bc7ef04075b	1	17543007	17545298	8	2291	NA	1	7	NA	NA	NA
Head-SCC	ef673d3d-2031-4036-ba25-4bc7ef04075b	1	19923509	19926215	8	2706	NA	6	2	NA	NA	NA
Head-SCC	ef673d3d-2031-4036-ba25-4bc7ef04075b	1	38036175	38039572	8	3397	NA	1	5	1	NA	1
Head-SCC	ef673d3d-2031-4036-ba25-4bc7ef04075b	1	232036121	232038334	8	2213	NA	5	3	NA	NA	NA
Head-SCC	ef673d3d-2031-4036-ba25-4bc7ef04075b	2	25529688	25530960	7	1272	NA	1	6	NA	NA	NA
Head-SCC	ef673d3d-2031-4036-ba25-4bc7ef04075b	3	176869213	176872782	8	3569	1	3	4	NA	NA	NA
Head-SCC	ef673d3d-2031-4036-ba25-4bc7ef04075b	3	176877869	176882770	11	4901	1	6	4	NA	NA	NA
Head-SCC	ef673d3d-2031-4036-ba25-4bc7ef04075b	3	183044613	183046729	7	2116	1	NA	6	NA	NA	NA
Head-SCC	ef673d3d-2031-4036-ba25-4bc7ef04075b	4	56197451	56202043	7	4592	1	2	3	NA	NA	1
Head-SCC	ef673d3d-2031-4036-ba25-4bc7ef04075b	6	2500652	2502230	6	1578	NA	3	3	NA	NA	NA
Head-SCC	ef673d3d-2031-4036-ba25-4bc7ef04075b	7	139098016	139099717	7	1701	3	1	3	NA	NA	NA
Head-SCC	ef673d3d-2031-4036-ba25-4bc7ef04075b	7	157137780	157140606	7	2826	NA	2	5	NA	NA	NA
Head-SCC	ef673d3d-2031-4036-ba25-4bc7ef04075b	8	126380721	126382723	8	2002	NA	2	6	NA	NA	NA
Head-SCC	ef673d3d-2031-4036-ba25-4bc7ef04075b	9	116036584	116039899	7	3315	1	3	3	NA	NA	NA
Head-SCC	ef673d3d-2031-4036-ba25-4bc7ef04075b	10	4697086	4703332	9	6246	NA	4	5	NA	NA	NA
Head-SCC	ef673d3d-2031-4036-ba25-4bc7ef04075b	10	61743500	61747730	11	4230	NA	4	7	NA	NA	NA
Head-SCC	ef673d3d-2031-4036-ba25-4bc7ef04075b	10	61828403	61831953	8	3550	NA	3	5	NA	NA	NA
Head-SCC	ef673d3d-2031-4036-ba25-4bc7ef04075b	11	64020606	64028194	18	7588	4	8	6	NA	NA	NA
Head-SCC	ef673d3d-2031-4036-ba25-4bc7ef04075b	12	51143230	51146342	9	3112	NA	2	7	NA	NA	NA
Head-SCC	ef673d3d-2031-4036-ba25-4bc7ef04075b	12	110918116	110920432	7	2316	1	2	3	NA	NA	1
Head-SCC	ef673d3d-2031-4036-ba25-4bc7ef04075b	13	22726938	22731293	7	4355	1	NA	5	1	NA	NA
Head-SCC	ef673d3d-2031-4036-ba25-4bc7ef04075b	13	42340405	42342192	7	1787	NA	2	5	NA	NA	NA
Head-SCC	ef673d3d-2031-4036-ba25-4bc7ef04075b	14	24810128	24816249	8	6121	NA	2	6	NA	NA	NA
Head-SCC	ef673d3d-2031-4036-ba25-4bc7ef04075b	15	78973841	78976080	8	2239	NA	NA	8	NA	NA	NA
Head-SCC	ef673d3d-2031-4036-ba25-4bc7ef04075b	16	28518068	28522431	6	4363	NA	3	3	NA	NA	NA
Head-SCC	ef673d3d-2031-4036-ba25-4bc7ef04075b	16	67384849	67386225	9	1376	1	3	5	NA	NA	NA
Head-SCC	ef673d3d-2031-4036-ba25-4bc7ef04075b	17	19232736	19238859	10	6123	1	4	5	NA	NA	NA
Head-SCC	ef673d3d-2031-4036-ba25-4bc7ef04075b	17	27942016	27943262	7	1246	NA	3	4	NA	NA	NA
Head-SCC	ef673d3d-2031-4036-ba25-4bc7ef04075b	17	74625693	74628446	8	2753	NA	2	6	NA	NA	NA
Head-SCC	ef673d3d-2031-4036-ba25-4bc7ef04075b	20	39115971	39120215	10	4244	2	4	4	NA	NA	NA
Head-SCC	ef673d3d-2031-4036-ba25-4bc7ef04075b	22	38005636	38009849	8	4213	NA	3	5	NA	NA	NA
Head-SCC	ef673d3d-2031-4036-ba25-4bc7ef04075b	22	43629444	43633924	15	4480	2	5	8	NA	NA	NA
Panc-AdenoCA	efacd7d6-56cf-4f6a-87f0-1a7ab6b1a643	18	28166197	28167899	10	1702	1	4	5	NA	NA	NA
Ovary-AdenoCA	efbec43c-0c16-4006-abe8-c3ec2ec42c05	2	138238823	138240563	8	1740	1	4	3	NA	NA	NA
Ovary-AdenoCA	efec3225-de07-4559-9a90-95223495cc61	12	121328003	121332444	13	4441	6	3	4	NA	NA	NA
Lymph-BNHL	f047cb85-8f88-45d5-83c3-0416c9f3fff7	2	89158944	89160084	27	1140	3	NA	6	9	6	3
Lymph-BNHL	f047cb85-8f88-45d5-83c3-0416c9f3fff7	2	89326774	89327050	10	276	NA	4	4	1	NA	1

Lymph-BNHL	f047cb85-8f88-45d5-83c3-0416c9f3fff7	12	122458999	122461729	7	2730	NA	2	4	NA	NA	1
Lymph-BNHL	f047cb85-8f88-45d5-83c3-0416c9f3fff7	14	106112507	106113957	12	1450	1	NA	11	NA	NA	NA
Lymph-BNHL	f047cb85-8f88-45d5-83c3-0416c9f3fff7	14	106209043	106213885	12	4842	1	1	8	2	NA	NA
Lymph-BNHL	f047cb85-8f88-45d5-83c3-0416c9f3fff7	14	106322219	106330289	58	8070	6	8	17	13	9	5
Lymph-BNHL	f047cb85-8f88-45d5-83c3-0416c9f3fff7	14	106725266	106733439	31	8173	1	3	13	8	4	2
Lymph-BNHL	f047cb85-8f88-45d5-83c3-0416c9f3fff7	18	60984100	60988475	33	4375	1	4	22	1	NA	5
Lymph-BNHL	f047cb85-8f88-45d5-83c3-0416c9f3fff7	22	23223147	23232313	30	9166	3	4	15	4	2	2
Lymph-BNHL	f04aecf0-eb12-4ab9-928e-7bcf201b0b40	2	89157759	89161381	104	3622	5	14	20	37	17	11
Lymph-BNHL	f04aecf0-eb12-4ab9-928e-7bcf201b0b40	14	106208649	106213275	9	4626	1	1	7	NA	NA	NA
Lymph-BNHL	f04aecf0-eb12-4ab9-928e-7bcf201b0b40	14	106240665	106241919	7	1254	NA	2	5	NA	NA	NA
Lymph-BNHL	f04aecf0-eb12-4ab9-928e-7bcf201b0b40	14	106322630	106329345	76	6715	8	9	29	17	3	10
Lymph-BNHL	f04aecf0-eb12-4ab9-928e-7bcf201b0b40	14	106994137	106994603	11	466	1	1	4	1	2	2
Lymph-BNHL	f04aecf0-eb12-4ab9-928e-7bcf201b0b40	18	60984967	60988121	7	3154	NA	3	2	1	NA	1
Lymph-BNHL	f04aecf0-eb12-4ab9-928e-7bcf201b0b40	22	23229878	23232007	22	2129	NA	4	4	6	4	4
Lymph-NOS	f075dcdb-4b6a-4186-bfc5-c1787c4a14ac	1	103449309	103451554	7	2245	NA	NA	1	5	1	NA
Lymph-NOS	f075dcdb-4b6a-4186-bfc5-c1787c4a14ac	2	89158480	89197265	105	38785	NA	12	9	50	12	22
Lymph-NOS	f075dcdb-4b6a-4186-bfc5-c1787c4a14ac	3	187461927	187468087	10	6160	NA	5	1	2	1	1
Lymph-NOS	f075dcdb-4b6a-4186-bfc5-c1787c4a14ac	7	14969597	14974889	9	5292	1	NA	NA	3	3	2
Lymph-NOS	f075dcdb-4b6a-4186-bfc5-c1787c4a14ac	7	78917147	78921548	9	4401	NA	NA	NA	5	NA	4
Lymph-NOS	f075dcdb-4b6a-4186-bfc5-c1787c4a14ac	12	122459043	122463596	6	4553	NA	2	NA	2	1	1
Lymph-NOS	f075dcdb-4b6a-4186-bfc5-c1787c4a14ac	14	106326519	106330719	78	4200	2	23	18	23	6	6
Lymph-NOS	f075dcdb-4b6a-4186-bfc5-c1787c4a14ac	14	106494176	106496230	27	2054	NA	2	6	12	4	3
Lymph-NOS	f075dcdb-4b6a-4186-bfc5-c1787c4a14ac	15	84343165	84346933	6	3768	NA	1	3	1	NA	1
Lymph-NOS	f075dcdb-4b6a-4186-bfc5-c1787c4a14ac	22	23223276	23227767	6	4491	1	1	2	NA	1	1
Lymph-NOS	f075dcdb-4b6a-4186-bfc5-c1787c4a14ac	22	23230106	23231737	15	1631	NA	1	6	5	2	1
Lymph-NOS	f075dcdb-4b6a-4186-bfc5-c1787c4a14ac	X	57257387	57257576	7	189	NA	NA	NA	3	NA	4
Biliary-AdenoCA	f0e0d978-3e32-4444-a07a-26049b2594e6	6	89052087	89055835	9	3748	1	3	5	NA	NA	NA
Biliary-AdenoCA	f0e0d978-3e32-4444-a07a-26049b2594e6	18	23428197	23429787	10	1590	1	1	8	NA	NA	NA
Biliary-AdenoCA	f0e0d978-3e32-4444-a07a-26049b2594e6	18	30624589	30626236	20	1647	2	5	13	NA	NA	NA
Biliary-AdenoCA	f0e0d978-3e32-4444-a07a-26049b2594e6	18	33597474	33598063	10	589	1	5	4	NA	NA	NA
Ovary-AdenoCA	f1504811-8363-41e6-b43c-62452b1262d3	18	24214996	24221423	14	6427	4	4	5	NA	NA	1
Ovary-AdenoCA	f1504811-8363-41e6-b43c-62452b1262d3	18	31751845	31757738	7	5893	NA	2	5	NA	NA	NA
Ovary-AdenoCA	f1504811-8363-41e6-b43c-62452b1262d3	18	72717860	72721501	12	3641	4	6	2	NA	NA	NA
Prost-AdenoCA	f1534b2a-5350-442c-bb9d-8c7142668d31	12	10905540	10913933	19	8393	2	9	8	NA	NA	NA
Prost-AdenoCA	f1534b2a-5350-442c-bb9d-8c7142668d31	12	12771316	12772177	7	861	NA	3	4	NA	NA	NA
Prost-AdenoCA	f1534b2a-5350-442c-bb9d-8c7142668d31	12	45490197	45493593	28	3396	1	6	21	NA	NA	NA
Liver-HCC	f181848a-c622-11e3-bf01-24c6515278c0	4	39498301	39498967	6	666	1	2	3	NA	NA	NA
Liver-HCC	f1a4f60a-5228-47cd-883f-ff8d2dfe1633	20	20960273	20961226	11	953	1	2	8	NA	NA	NA
Liver-HCC	f1a4f60a-5228-47cd-883f-ff8d2dfe1633	20	22568057	22569501	13	1444	1	6	6	NA	NA	NA
Liver-HCC	f1a4f60a-5228-47cd-883f-ff8d2dfe1633	20	61934893	61937407	11	2514	3	5	3	NA	NA	NA
Panc-AdenoCA	f1d9124e-dfa2-415e-b8b8-dd872fd3e2cb	4	117943569	117947606	8	4037	NA	NA	NA	3	5	NA
Panc-AdenoCA	f1d9124e-dfa2-415e-b8b8-dd872fd3e2cb	11	55366000	55372733	10	6733	NA	NA	NA	2	5	3
Panc-AdenoCA	f1d9124e-dfa2-415e-b8b8-dd872fd3e2cb	15	78652468	78652886	9	418	NA	2	7	NA	NA	NA
Bone-Osteosarc	f221c897-6ad0-0df9-e040-11ac0c4813ef	3	80455117	80457760	16	2643	2	4	10	NA	NA	NA
Bone-Osteosarc	f221c897-6ad0-0df9-e040-11ac0c4813ef	8	75471704	75473591	6	1887	NA	NA	1	5	NA	NA
Bone-Osteosarc	f221c897-6ad0-0df9-e040-11ac0c4813ef	18	21450894	21453307	9	2413	1	1	7	NA	NA	NA
Breast-AdenoCa	f221cbb5-eefa-187f-e040-11ac0c481708	6	126233148	126394625	191	2E+05	1	4	##	NA	NA	NA
Breast-AdenoCa	f221cbb5-eefa-187f-e040-11ac0c481708	6	126430813	126437625	178	6812	2	2	##	NA	NA	NA
Breast-AdenoCa	f221cbb5-eefa-187f-e040-11ac0c481708	6	130419337	130489254	160	69917	9	13	##	1	NA	NA
Breast-AdenoCa	f221cbb5-eefa-187f-e040-11ac0c481708	6	131788326	131792696	7	4370	NA	1	6	NA	NA	NA
Breast-AdenoCa	f221cbb5-eefa-187f-e040-11ac0c481708	6	131810126	131818990	31	8864	1	NA	29	1	NA	NA
Breast-AdenoCa	f221cbb5-eefa-187f-e040-11ac0c481708	6	132396811	132410166	17	13355	1	1	15	NA	NA	NA
Breast-AdenoCa	f221cbb5-eefa-187f-e040-11ac0c481708	6	132544956	132552483	12	7527	1	1	10	NA	NA	NA
Breast-AdenoCa	f221cbb5-eefa-187f-e040-11ac0c481708	6	132599455	132603528	50	4073	2	7	41	NA	NA	NA
Breast-AdenoCa	f221cbb5-eefa-187f-e040-11ac0c481708	6	133707397	133716520	18	9123	NA	NA	18	NA	NA	NA
Breast-AdenoCa	f221cbb5-eefa-187f-e040-11ac0c481708	6	134015015	134021775	8	6760	1	2	5	NA	NA	NA
Breast-AdenoCa	f221cbb5-eefa-187f-e040-11ac0c481708	6	134024091	134025308	11	1217	1	1	9	NA	NA	NA
Breast-AdenoCa	f221cbb5-eefa-187f-e040-11ac0c481708	6	134115823	134118849	8	3026	1	1	5	NA	NA	1
Breast-AdenoCa	f221cbb5-eefa-187f-e040-11ac0c481708	6	135256447	135262041	9	5594	1	3	5	NA	NA	NA
Breast-AdenoCa	f221cbb5-eefa-187f-e040-11ac0c481708	6	137979704	137982971	7	3267	1	3	3	NA	NA	NA

Breast-AdenoCa	f221cbb5-eefa-187f-e040-11ac0c481708	6	138010047	138018379	12	8332	NA	1	10	1	NA	NA
Breast-AdenoCa	f221cbb5-eefa-187f-e040-11ac0c481708	12	10504996	10508274	14	3278	2	NA	12	NA	NA	NA
Ovary-AdenoCA	f26b1f44-12de-43ba-85bb-bc61741a5a88	1	42274794	42276427	16	1633	4	1	10	NA	NA	1
Ovary-AdenoCA	f26b1f44-12de-43ba-85bb-bc61741a5a88	1	43081573	43082739	10	1166	1	2	7	NA	NA	NA
Ovary-AdenoCA	f26b1f44-12de-43ba-85bb-bc61741a5a88	2	157464881	157466066	7	1185	2	4	1	NA	NA	NA
Ovary-AdenoCA	f26b1f44-12de-43ba-85bb-bc61741a5a88	4	17754548	17758952	8	4404	2	4	2	NA	NA	NA
Ovary-AdenoCA	f26b1f44-12de-43ba-85bb-bc61741a5a88	6	55444084	55444695	6	611	1	3	2	NA	NA	NA
Ovary-AdenoCA	f26b1f44-12de-43ba-85bb-bc61741a5a88	7	21567659	21569090	14	1431	2	9	3	NA	NA	NA
Panc-AdenoCA	f283ed80-8302-4f26-99ed-ea20d101289d	1	4929732	4933773	22	4041	2	8	12	NA	NA	NA
Biliary-AdenoCA	f2b2111c-c622-11e3-bf01-24c6515278c0	10	60787479	60787913	6	434	NA	NA	NA	2	4	NA
Lymph-BNHL	f2c08ac1-4755-4c36-a11d-338d8df9fa0d	2	89159862	89166508	53	6646	2	10	12	13	6	10
Lymph-BNHL	f2c08ac1-4755-4c36-a11d-338d8df9fa0d	2	89246833	89247480	8	647	NA	2	2	2	1	1
Lymph-BNHL	f2c08ac1-4755-4c36-a11d-338d8df9fa0d	6	143772810	143775680	16	2870	NA	7	9	NA	NA	NA
Lymph-BNHL	f2c08ac1-4755-4c36-a11d-338d8df9fa0d	14	106112657	106113989	10	1332	2	2	6	NA	NA	NA
Lymph-BNHL	f2c08ac1-4755-4c36-a11d-338d8df9fa0d	14	106211760	106212587	8	827	1	3	4	NA	NA	NA
Lymph-BNHL	f2c08ac1-4755-4c36-a11d-338d8df9fa0d	14	106324798	106330339	56	5541	9	9	23	6	3	6
Lymph-BNHL	f2c08ac1-4755-4c36-a11d-338d8df9fa0d	18	60985900	60988291	16	2391	1	3	7	2	2	1
Eso-AdenoCa	f2e639cc-c30c-459f-8afc-7ace7d0209c5	3	177718662	177723125	13	4463	1	8	4	NA	NA	NA
Eso-AdenoCa	f2e639cc-c30c-459f-8afc-7ace7d0209c5	6	64890956	64894273	7	3317	2	3	2	NA	NA	NA
Eso-AdenoCa	f2e639cc-c30c-459f-8afc-7ace7d0209c5	10	28910009	28911127	26	1118	6	7	13	NA	NA	NA
Eso-AdenoCa	f2e639cc-c30c-459f-8afc-7ace7d0209c5	13	94746753	94747656	17	903	3	3	11	NA	NA	NA
Eso-AdenoCa	f2e639cc-c30c-459f-8afc-7ace7d0209c5	17	42629326	42629773	9	447	NA	1	8	NA	NA	NA
Eso-AdenoCa	f2e639cc-c30c-459f-8afc-7ace7d0209c5	18	9881792	9882629	6	837	1	2	3	NA	NA	NA
Head-SCC	f35f7712-d5c6-47f6-98ed-704edbbc1e19	6	70441272	70446489	20	5217	2	6	12	NA	NA	NA
Head-SCC	f35f7712-d5c6-47f6-98ed-704edbbc1e19	8	208414	223318	21	14904	NA	3	17	1	NA	NA
Head-SCC	f35f7712-d5c6-47f6-98ed-704edbbc1e19	8	36066524	36067812	12	1288	NA	1	11	NA	NA	NA
Head-SCC	f35f7712-d5c6-47f6-98ed-704edbbc1e19	10	70578351	70581895	8	3544	NA	2	6	NA	NA	NA
Head-SCC	f35f7712-d5c6-47f6-98ed-704edbbc1e19	13	33123519	33125057	6	1538	NA	NA	6	NA	NA	NA
Head-SCC	f35f7712-d5c6-47f6-98ed-704edbbc1e19	15	81969233	81971033	7	1800	NA	1	6	NA	NA	NA
Head-SCC	f35f7712-d5c6-47f6-98ed-704edbbc1e19	18	59355713	59358746	6	3033	1	4	1	NA	NA	NA
Head-SCC	f35f7712-d5c6-47f6-98ed-704edbbc1e19	21	40132878	40134510	6	1632	1	3	2	NA	NA	NA
Head-SCC	f35f7712-d5c6-47f6-98ed-704edbbc1e19	X	70429705	70436558	8	6853	NA	5	3	NA	NA	NA
Bladder-TCC	f389176f-d8f3-45c2-aae4-7378a3d6fc7f	2	67771988	67774398	6	2410	NA	3	3	NA	NA	NA
Bladder-TCC	f389176f-d8f3-45c2-aae4-7378a3d6fc7f	2	158989085	158992390	7	3305	1	3	3	NA	NA	NA
Bladder-TCC	f389176f-d8f3-45c2-aae4-7378a3d6fc7f	3	1588129	1592679	10	4550	3	5	NA	1	NA	1
Bladder-TCC	f389176f-d8f3-45c2-aae4-7378a3d6fc7f	8	25097988	25100151	6	2163	2	4	NA	NA	NA	NA
Bladder-TCC	f389176f-d8f3-45c2-aae4-7378a3d6fc7f	14	37505257	37518272	20	13015	8	5	7	NA	NA	NA
Bladder-TCC	f389176f-d8f3-45c2-aae4-7378a3d6fc7f	14	37552189	37558814	11	6625	3	3	5	NA	NA	NA
Bladder-TCC	f389176f-d8f3-45c2-aae4-7378a3d6fc7f	14	37605199	37607047	14	1848	9	3	2	NA	NA	NA
Bladder-TCC	f389176f-d8f3-45c2-aae4-7378a3d6fc7f	14	37619257	37631023	20	11766	9	11	NA	NA	NA	NA
Bladder-TCC	f389176f-d8f3-45c2-aae4-7378a3d6fc7f	15	29262627	29267475	32	4848	4	14	14	NA	NA	NA
Bladder-TCC	f389176f-d8f3-45c2-aae4-7378a3d6fc7f	17	45723299	45723527	8	228	2	3	3	NA	NA	NA
Bladder-TCC	f389176f-d8f3-45c2-aae4-7378a3d6fc7f	18	19020669	19020772	8	103	NA	4	4	NA	NA	NA
Bladder-TCC	f389176f-d8f3-45c2-aae4-7378a3d6fc7f	20	33304937	33312373	20	7436	2	5	12	1	NA	NA
ColoRect-AdenoCA	f38b5d2e-5cab-45c7-bb0a-38b2efc5c156	1	176842114	176844318	10	2204	NA	5	5	NA	NA	NA
ColoRect-AdenoCA	f38b5d2e-5cab-45c7-bb0a-38b2efc5c156	5	180116712	180117546	7	834	NA	5	2	NA	NA	NA
ColoRect-AdenoCA	f38b5d2e-5cab-45c7-bb0a-38b2efc5c156	7	53012987	53016607	11	3620	5	3	2	1	NA	NA
ColoRect-AdenoCA	f38b5d2e-5cab-45c7-bb0a-38b2efc5c156	10	6821686	6830945	11	9259	2	6	2	1	NA	NA
ColoRect-AdenoCA	f38b5d2e-5cab-45c7-bb0a-38b2efc5c156	10	27904295	27909739	9	5444	3	6	NA	NA	NA	NA
ColoRect-AdenoCA	f38b5d2e-5cab-45c7-bb0a-38b2efc5c156	12	2556012	2558744	8	2732	1	3	4	NA	NA	NA
ColoRect-AdenoCA	f38b5d2e-5cab-45c7-bb0a-38b2efc5c156	19	22942175	22943757	6	1582	1	3	2	NA	NA	NA
Breast-AdenoCa	f393bafd-1baa-e5f4-e040-11ac0d48450b	5	173624840	173626444	15	1604	1	9	5	NA	NA	NA
Breast-AdenoCa	f393bafd-1baa-e5f4-e040-11ac0d48450b	6	16654698	16654723	6	25	NA	NA	4	2	NA	NA
Breast-AdenoCa	f393bafd-1baa-e5f4-e040-11ac0d48450b	8	37611250	37614750	8	3500	NA	6	2	NA	NA	NA
Breast-AdenoCa	f393bafd-1baa-e5f4-e040-11ac0d48450b	8	62786214	62786966	7	752	NA	3	4	NA	NA	NA
Breast-AdenoCa	f393bafd-1baa-e5f4-e040-11ac0d48450b	11	20542416	20546086	24	3670	1	11	11	NA	NA	1
Breast-AdenoCa	f393bafd-1baa-e5f4-e040-11ac0d48450b	15	25671387	25674669	6	3282	1	3	2	NA	NA	NA
Breast-AdenoCa	f393bafe-7503-5c45-e040-11ac0d484511	2	34262625	34284709	34	22084	14	16	4	NA	NA	NA
Breast-AdenoCa	f393bafe-7503-5c45-e040-11ac0d484511	15	97406152	97409080	6	2928	2	NA	1	NA	NA	3
Breast-AdenoCa	f393bb00-888d-710f-e040-11ac0d484518	4	56246905	56249256	7	2351	3	3	1	NA	NA	NA

Breast-AdenoCa	f393bb00-888d-710f-e040-11ac0d484518	4	56254598	56255693	7	1095	1	NA	6	NA	NA	NA
Breast-AdenoCa	f393bb01-6ed7-9533-e040-11ac0d484521	7	121441036	121445905	6	4869	NA	4	1	1	NA	NA
Breast-AdenoCa	f393bb01-6ed7-9533-e040-11ac0d484521	9	91359485	91363713	7	4228	1	4	2	NA	NA	NA
Breast-AdenoCa	f393bb01-6ed7-9533-e040-11ac0d484521	10	37885462	37888921	19	3459	8	8	2	NA	NA	1
Breast-AdenoCa	f393bb01-6ed7-9533-e040-11ac0d484521	10	38201372	38203035	30	1663	9	7	13	NA	NA	1
Breast-AdenoCa	f393bb01-6ed7-9533-e040-11ac0d484521	10	121309080	121322097	16	13017	1	5	10	NA	NA	NA
Breast-AdenoCa	f393bb05-53c2-f80a-e040-11ac0d484528	2	67735650	67744177	16	8527	2	7	6	NA	NA	1
Breast-AdenoCa	f393bb05-ec1c-17be-e040-11ac0d48452f	2	132363019	132364134	6	1115	1	3	2	NA	NA	NA
Breast-AdenoCa	f393bb05-ec1c-17be-e040-11ac0d48452f	3	73649074	73660947	15	11873	1	1	13	NA	NA	NA
Breast-AdenoCa	f393bb05-ec1c-17be-e040-11ac0d48452f	3	73664621	73666636	7	2015	NA	1	6	NA	NA	NA
Breast-AdenoCa	f393bb05-ec1c-17be-e040-11ac0d48452f	3	74093063	74108819	19	15756	NA	11	8	NA	NA	NA
Breast-AdenoCa	f393bb05-ec1c-17be-e040-11ac0d48452f	3	77898746	77903154	8	4408	NA	2	6	NA	NA	NA
Breast-AdenoCa	f393bb05-ec1c-17be-e040-11ac0d48452f	3	96463847	96465970	7	2123	2	4	1	NA	NA	NA
Breast-AdenoCa	f393bb05-ec1c-17be-e040-11ac0d48452f	4	54328647	54330646	6	1999	NA	NA	6	NA	NA	NA
Breast-AdenoCa	f393bb05-ec1c-17be-e040-11ac0d48452f	4	56434812	56436213	8	1401	1	NA	7	NA	NA	NA
Breast-AdenoCa	f393bb05-ec1c-17be-e040-11ac0d48452f	4	74625193	74626287	17	1094	NA	NA	17	NA	NA	NA
Breast-AdenoCa	f393bb05-ec1c-17be-e040-11ac0d48452f	4	75558707	75560994	8	2287	NA	8	NA	NA	NA	NA
Breast-AdenoCa	f393bb05-ec1c-17be-e040-11ac0d48452f	4	76987429	76990089	10	2660	NA	3	7	NA	NA	NA
Breast-AdenoCa	f393bb05-ec1c-17be-e040-11ac0d48452f	4	88912564	88924250	24	11686	2	13	9	NA	NA	NA
Breast-AdenoCa	f393bb05-ec1c-17be-e040-11ac0d48452f	4	126024088	126027474	14	3386	NA	8	6	NA	NA	NA
Breast-AdenoCa	f393bb05-ec1c-17be-e040-11ac0d48452f	4	126198161	126201366	10	3205	NA	NA	10	NA	NA	NA
Breast-AdenoCa	f393bb05-ec1c-17be-e040-11ac0d48452f	4	157227818	157229680	9	1862	NA	6	3	NA	NA	NA
Breast-AdenoCa	f393bb05-ec1c-17be-e040-11ac0d48452f	4	164420639	164428829	16	8190	3	4	9	NA	NA	NA
Breast-AdenoCa	f393bb05-ec1c-17be-e040-11ac0d48452f	6	158151640	158157605	31	5965	2	15	14	NA	NA	NA
Breast-AdenoCa	f393bb05-ec1c-17be-e040-11ac0d48452f	8	43101096	43105420	8	4324	NA	5	3	NA	NA	NA
Breast-AdenoCa	f393bb05-ec1c-17be-e040-11ac0d48452f	8	88697677	88699927	8	2250	NA	6	2	NA	NA	NA
Breast-AdenoCa	f393bb05-ec1c-17be-e040-11ac0d48452f	8	91702622	91704432	9	1810	1	4	4	NA	NA	NA
Breast-AdenoCa	f393bb05-ec1c-17be-e040-11ac0d48452f	8	91902732	91904826	9	2094	NA	3	6	NA	NA	NA
Breast-AdenoCa	f393bb05-ec1c-17be-e040-11ac0d48452f	10	17287099	17287982	6	883	2	1	3	NA	NA	NA
Breast-AdenoCa	f393bb05-ec1c-17be-e040-11ac0d48452f	10	24506055	24507243	18	1188	1	2	15	NA	NA	NA
Breast-AdenoCa	f393bb05-ec1c-17be-e040-11ac0d48452f	10	24724120	24726795	15	2675	1	9	5	NA	NA	NA
Breast-AdenoCa	f393bb05-ec1c-17be-e040-11ac0d48452f	10	25405061	25405857	7	796	2	2	3	NA	NA	NA
Breast-AdenoCa	f393bb05-ec1c-17be-e040-11ac0d48452f	10	55289338	55294185	8	4847	1	4	2	NA	1	NA
Breast-AdenoCa	f393bb05-ec1c-17be-e040-11ac0d48452f	10	57287133	57289397	10	2264	1	5	4	NA	NA	NA
Breast-AdenoCa	f393bb05-ec1c-17be-e040-11ac0d48452f	10	57323991	57329065	27	5074	7	14	5	NA	NA	1
Breast-AdenoCa	f393bb05-ec1c-17be-e040-11ac0d48452f	11	66514354	66517276	12	2922	5	3	4	NA	NA	NA
Breast-AdenoCa	f393bb05-ec1c-17be-e040-11ac0d48452f	11	79897069	79897422	8	353	NA	3	5	NA	NA	NA
Breast-AdenoCa	f393bb05-ec1c-17be-e040-11ac0d48452f	11	80439985	80440147	7	162	NA	NA	7	NA	NA	NA
Breast-AdenoCa	f393bb05-ec1c-17be-e040-11ac0d48452f	12	26476587	26478884	8	2297	1	6	1	NA	NA	NA
Breast-AdenoCa	f393bb05-ec1c-17be-e040-11ac0d48452f	12	66725767	66731227	10	5460	1	5	3	1	NA	NA
Breast-AdenoCa	f393bb05-ec1c-17be-e040-11ac0d48452f	12	66844953	66856770	19	11817	2	12	4	NA	NA	1
Breast-AdenoCa	f393bb05-ec1c-17be-e040-11ac0d48452f	12	67313572	67315554	7	1982	NA	4	3	NA	NA	NA
Breast-AdenoCa	f393bb05-ec1c-17be-e040-11ac0d48452f	12	67633021	67634276	7	1255	1	6	NA	NA	NA	NA
Breast-AdenoCa	f393bb05-ec1c-17be-e040-11ac0d48452f	12	68453716	68455076	11	1360	1	3	6	NA	NA	1
Breast-AdenoCa	f393bb05-ec1c-17be-e040-11ac0d48452f	12	70985062	70994342	19	9280	1	7	11	NA	NA	NA
Breast-AdenoCa	f393bb05-ec1c-17be-e040-11ac0d48452f	12	71418699	71422455	9	3756	NA	4	5	NA	NA	NA
Breast-AdenoCa	f393bb05-ec1c-17be-e040-11ac0d48452f	12	71722726	71729176	9	6450	NA	3	6	NA	NA	NA
Breast-AdenoCa	f393bb05-ec1c-17be-e040-11ac0d48452f	12	71850536	71852522	11	1986	NA	5	6	NA	NA	NA
Breast-AdenoCa	f393bb05-ec1c-17be-e040-11ac0d48452f	12	71862650	71867824	11	5174	2	5	4	NA	NA	NA
Breast-AdenoCa	f393bb05-ec1c-17be-e040-11ac0d48452f	12	73437579	73441470	14	3891	1	6	7	NA	NA	NA
Breast-AdenoCa	f393bb05-ec1c-17be-e040-11ac0d48452f	12	73453894	73455816	9	1922	NA	NA	9	NA	NA	NA
Breast-AdenoCa	f393bb05-ec1c-17be-e040-11ac0d48452f	12	73480480	73482074	10	1594	2	2	6	NA	NA	NA
Breast-AdenoCa	f393bb05-ec1c-17be-e040-11ac0d48452f	13	49401070	49402728	6	1658	NA	NA	6	NA	NA	NA
Breast-AdenoCa	f393bb05-ec1c-17be-e040-11ac0d48452f	13	49412727	49414195	11	1468	1	2	8	NA	NA	NA
Breast-AdenoCa	f393bb05-ec1c-17be-e040-11ac0d48452f	16	65595712	65596793	11	1081	NA	NA	11	NA	NA	NA
Breast-AdenoCa	f393bb05-ec1c-17be-e040-11ac0d48452f	17	7492600	7497727	20	5127	2	12	6	NA	NA	NA
Breast-AdenoCa	f393bb05-ec1c-17be-e040-11ac0d48452f	17	8929005	8929566	7	561	NA	NA	7	NA	NA	NA
Breast-AdenoCa	f393bb05-ec1c-17be-e040-11ac0d48452f	19	37797662	37798516	9	854	NA	1	8	NA	NA	NA
Breast-AdenoCa	f393bb05-ec1c-17be-e040-11ac0d48452f	20	31472480	31478185	24	5705	3	15	6	NA	NA	NA
Breast-AdenoCa	f393bb05-ec1c-17be-e040-11ac0d48452f	21	18355830	18357993	6	2163	NA	4	1	NA	NA	1

Breast-AdenoCa	f393bb05-ec1c-17be-e040-11ac0d48452f	X	32123861	32124505	9	644	NA	5	3	NA	NA	1
Breast-AdenoCa	f393bb05-ec1c-17be-e040-11ac0d48452f	X	32345326	32348028	12	2702	1	4	7	NA	NA	NA
Breast-AdenoCa	f393bb05-ec1c-17be-e040-11ac0d48452f	X	32743161	32744197	8	1036	NA	7	1	NA	NA	NA
Breast-AdenoCa	f393bb07-270c-2c93-e040-11ac0d484533	11	68962929	68963994	6	1065	NA	4	2	NA	NA	NA
Breast-AdenoCa	f393bb07-270c-2c93-e040-11ac0d484533	11	78824054	78825767	8	1713	NA	4	4	NA	NA	NA
Breast-AdenoCa	f393bb08-5b50-e009-e040-11ac0d484537	5	55991444	55996280	13	4836	3	8	1	NA	1	NA
Breast-AdenoCa	f393bb08-5b50-e009-e040-11ac0d484537	10	115268236	115272259	8	4023	1	NA	1	3	NA	3
Breast-AdenoCa	f393bb08-5b50-e009-e040-11ac0d484537	10	117593152	117594233	9	1081	NA	6	3	NA	NA	NA
Breast-AdenoCa	f393bb08-5b50-e009-e040-11ac0d484537	12	48661295	48662234	8	939	1	4	3	NA	NA	NA
Breast-AdenoCa	f393bb08-5b50-e009-e040-11ac0d484537	16	23595884	23597189	7	1305	1	2	4	NA	NA	NA
Breast-AdenoCa	f393bb08-5b50-e009-e040-11ac0d484537	17	21217055	21220257	10	3202	NA	NA	10	NA	NA	NA
Breast-AdenoCa	f393bb08-5b50-e009-e040-11ac0d484537	18	28267552	28270158	14	2606	2	9	3	NA	NA	NA
Breast-AdenoCa	f393bb08-5b50-e009-e040-11ac0d484537	19	24160140	24160598	6	458	1	3	1	NA	1	NA
Breast-AdenoCa	f393bb08-5b50-e009-e040-11ac0d484537	22	44489193	44494957	10	5764	1	7	2	NA	NA	NA
Breast-AdenoCa	f393bb0a-9b20-a0e5-e040-11ac0d48454e	1	40804052	40804517	9	465	NA	8	1	NA	NA	NA
Breast-AdenoCa	f393bb0a-9b20-a0e5-e040-11ac0d48454e	8	39131841	39135563	16	3722	NA	5	11	NA	NA	NA
Breast-AdenoCa	f393bb0a-9b20-a0e5-e040-11ac0d48454e	14	34483513	34487399	11	3886	3	2	6	NA	NA	NA
Breast-AdenoCa	f393bb0a-9b20-a0e5-e040-11ac0d48454e	17	37497596	37504367	14	6771	1	4	9	NA	NA	NA
Breast-AdenoCa	f393bb0a-df7d-645c-e040-11ac0d484550	1	52686607	52687615	7	1008	NA	3	4	NA	NA	NA
Breast-AdenoCa	f393bb0a-df7d-645c-e040-11ac0d484550	1	58209671	58216379	23	6708	2	6	15	NA	NA	NA
Breast-AdenoCa	f393bb0a-df7d-645c-e040-11ac0d484550	1	58811855	58812442	8	587	NA	8	NA	NA	NA	NA
Breast-AdenoCa	f393bb0a-df7d-645c-e040-11ac0d484550	2	45284339	45285531	6	1192	NA	3	3	NA	NA	NA
Breast-AdenoCa	f393bb0a-df7d-645c-e040-11ac0d484550	2	145050921	145056596	7	5675	NA	1	6	NA	NA	NA
Breast-AdenoCa	f393bb0a-df7d-645c-e040-11ac0d484550	2	203675091	203682856	16	7765	3	5	8	NA	NA	NA
Breast-AdenoCa	f393bb0a-df7d-645c-e040-11ac0d484550	3	72919362	72925993	14	6631	1	8	4	NA	NA	1
Breast-AdenoCa	f393bb0a-df7d-645c-e040-11ac0d484550	5	11772687	11774767	7	2080	NA	2	5	NA	NA	NA
Breast-AdenoCa	f393bb0a-df7d-645c-e040-11ac0d484550	5	67020081	67022763	27	2682	NA	4	23	NA	NA	NA
Breast-AdenoCa	f393bb0a-df7d-645c-e040-11ac0d484550	6	109632327	109641082	10	8755	1	2	7	NA	NA	NA
Breast-AdenoCa	f393bb0a-df7d-645c-e040-11ac0d484550	6	109642711	109649666	9	6955	2	NA	7	NA	NA	NA
Breast-AdenoCa	f393bb0a-df7d-645c-e040-11ac0d484550	8	108218755	108219861	14	1106	5	5	4	NA	NA	NA
Breast-AdenoCa	f393bb0a-df7d-645c-e040-11ac0d484550	8	128408127	128433414	33	25287	6	10	17	NA	NA	NA
Breast-AdenoCa	f393bb0a-df7d-645c-e040-11ac0d484550	9	117390817	117394646	6	3829	2	1	3	NA	NA	NA
Breast-AdenoCa	f393bb0a-df7d-645c-e040-11ac0d484550	11	70769665	70775331	7	5666	1	1	5	NA	NA	NA
Breast-AdenoCa	f393bb0a-df7d-645c-e040-11ac0d484550	11	70776699	70784309	9	7610	NA	2	7	NA	NA	NA
Breast-AdenoCa	f393bb0a-df7d-645c-e040-11ac0d484550	12	56497424	56500493	6	3069	1	NA	5	NA	NA	NA
Breast-AdenoCa	f393bb0a-df7d-645c-e040-11ac0d484550	12	65421768	65427555	9	5787	2	3	4	NA	NA	NA
Breast-AdenoCa	f393bb0a-df7d-645c-e040-11ac0d484550	12	113223785	113228606	10	4821	2	3	5	NA	NA	NA
Breast-AdenoCa	f393bb0a-df7d-645c-e040-11ac0d484550	12	115315473	115318646	9	3173	1	3	5	NA	NA	NA
Breast-AdenoCa	f393bb0a-df7d-645c-e040-11ac0d484550	15	26266291	26271068	8	4777	1	3	4	NA	NA	NA
Breast-AdenoCa	f393bb0a-df7d-645c-e040-11ac0d484550	15	61374295	61382676	13	8381	1	3	9	NA	NA	NA
Breast-AdenoCa	f393bb0a-df7d-645c-e040-11ac0d484550	16	4963730	4965780	6	2050	NA	3	3	NA	NA	NA
Breast-AdenoCa	f393bb0a-df7d-645c-e040-11ac0d484550	17	37371264	37375915	6	4651	1	NA	4	NA	NA	1
Breast-AdenoCa	f393bb0a-df7d-645c-e040-11ac0d484550	17	37949858	37956355	17	6497	NA	7	10	NA	NA	NA
Breast-AdenoCa	f393bb0a-df7d-645c-e040-11ac0d484550	19	42849576	42871045	30	21469	2	5	23	NA	NA	NA
Breast-AdenoCa	f393bb0a-df7d-645c-e040-11ac0d484550	19	47527623	47529863	11	2240	NA	4	7	NA	NA	NA
Breast-AdenoCa	f393bb0b-08ed-3335-e040-11ac0d484554	3	188844053	188851859	21	7806	6	5	9	NA	NA	1
Breast-AdenoCa	f393bb0b-08ed-3335-e040-11ac0d484554	8	17688977	17689461	12	484	6	5	NA	NA	NA	1
Breast-AdenoCa	f393bb0b-08ed-3335-e040-11ac0d484554	8	43703674	43704447	7	773	4	3	NA	NA	NA	NA
Breast-AdenoCa	f393bb0b-08ed-3335-e040-11ac0d484554	8	59929821	59931043	8	1222	2	3	3	NA	NA	NA
Breast-AdenoCa	f393bb0b-08ed-3335-e040-11ac0d484554	11	4233607	4234939	12	1332	2	4	6	NA	NA	NA
Breast-AdenoCa	f393bb0b-08ed-3335-e040-11ac0d484554	11	90521038	90524583	7	3545	1	3	3	NA	NA	NA
Breast-AdenoCa	f393bb0b-08ed-3335-e040-11ac0d484554	14	103258683	103265053	13	6370	1	4	8	NA	NA	NA
Breast-AdenoCa	f393bb0b-08ed-3335-e040-11ac0d484554	19	13389122	13389561	7	439	1	2	4	NA	NA	NA
Breast-AdenoCa	f393bb0b-08ed-3335-e040-11ac0d484554	19	15711458	15715746	10	4288	2	7	NA	NA	NA	1
Breast-AdenoCa	f393bb0b-08ed-3335-e040-11ac0d484554	19	33310259	33314350	10	4091	3	3	4	NA	NA	NA
Liver-HCC	f410b432-c622-11e3-bf01-24c6515278c0	5	105723383	105724404	6	1021	NA	NA	NA	3	2	1
Eso-AdenoCa	f48c3c82-bebe-4b8e-909e-e1a51a7142ec	1	114912629	114916521	8	3892	2	4	2	NA	NA	NA
Eso-AdenoCa	f48c3c82-bebe-4b8e-909e-e1a51a7142ec	10	17571104	17579832	11	8728	3	5	3	NA	NA	NA
Eso-AdenoCa	f48c3c82-bebe-4b8e-909e-e1a51a7142ec	11	98004688	98005829	10	1141	4	2	4	NA	NA	NA
Eso-AdenoCa	f48c3c82-bebe-4b8e-909e-e1a51a7142ec	13	58648587	58649298	11	711	5	5	1	NA	NA	NA

Eso-AdenoCa	f48c3c82-bebe-4b8e-909e-e1a51a7142ec	18	22821208	22822228	7	1020	NA	4	2	NA	NA	1
Panc-AdenoCA	f4e673a9-5b85-46e7-bcac-2ce6e1026896	2	123805969	123811553	7	5584	NA	NA	1	5	1	NA
Panc-AdenoCA	f4e673a9-5b85-46e7-bcac-2ce6e1026896	4	59990298	60000618	15	10320	1	NA	NA	4	6	4
Panc-AdenoCA	f4e673a9-5b85-46e7-bcac-2ce6e1026896	4	131205817	131214845	12	9028	NA	NA	NA	7	4	1
Panc-AdenoCA	f4e926fd-006e-4c85-b71c-1433c325acb0	1	68836812	68838858	19	2046	NA	9	10	NA	NA	NA
Panc-AdenoCA	f4e926fd-006e-4c85-b71c-1433c325acb0	6	35157723	35160814	10	3091	2	4	3	NA	NA	1
Panc-AdenoCA	f4e926fd-006e-4c85-b71c-1433c325acb0	8	15524505	15528291	6	3786	NA	4	2	NA	NA	NA
Panc-AdenoCA	f4e926fd-006e-4c85-b71c-1433c325acb0	9	97386817	97393717	31	6900	1	14	15	NA	1	NA
Panc-AdenoCA	f4e926fd-006e-4c85-b71c-1433c325acb0	18	48478784	48479322	6	538	NA	NA	6	NA	NA	NA
Prost-AdenoCA	f5378545-17d4-4a64-a57e-f6c91ef4cb3a	1	239013752	239017948	6	4196	1	NA	2	1	1	1
Prost-AdenoCA	f5378545-17d4-4a64-a57e-f6c91ef4cb3a	2	222267845	222268260	6	415	1	NA	NA	1	4	NA
Prost-AdenoCA	f5378545-17d4-4a64-a57e-f6c91ef4cb3a	4	116573893	116575274	6	1381	NA	1	1	2	2	NA
Prost-AdenoCA	f5378545-17d4-4a64-a57e-f6c91ef4cb3a	4	145167256	145169638	7	2382	NA	NA	2	1	3	1
Prost-AdenoCA	f5378545-17d4-4a64-a57e-f6c91ef4cb3a	7	62130930	62133010	6	2080	1	NA	2	1	NA	2
Prost-AdenoCA	f5378545-17d4-4a64-a57e-f6c91ef4cb3a	8	75298534	75300752	6	2218	NA	NA	2	3	1	NA
Prost-AdenoCA	f5378545-17d4-4a64-a57e-f6c91ef4cb3a	8	134624891	134633533	16	8642	2	8	6	NA	NA	NA
Prost-AdenoCA	f5378545-17d4-4a64-a57e-f6c91ef4cb3a	13	93445064	93447502	7	2438	NA	2	2	1	2	NA
Prost-AdenoCA	f5378545-17d4-4a64-a57e-f6c91ef4cb3a	14	63573583	63575765	6	2182	NA	NA	2	2	NA	2
Prost-AdenoCA	f5378545-17d4-4a64-a57e-f6c91ef4cb3a	15	97822907	97823788	7	881	NA	NA	2	4	NA	1
Prost-AdenoCA	f5378545-17d4-4a64-a57e-f6c91ef4cb3a	18	68000850	68003154	7	2304	NA	2	5	NA	NA	NA
Prost-AdenoCA	f5378545-17d4-4a64-a57e-f6c91ef4cb3a	X	21230556	21236966	9	6410	NA	NA	4	1	3	1
Prost-AdenoCA	f5378545-17d4-4a64-a57e-f6c91ef4cb3a	Y	9914315	9918843	6	4528	NA	1	3	NA	1	1
Panc-AdenoCA	f5bb7ae4-4bb4-4e87-b83c-bc8620584989	17	73492855	73494951	6	2096	1	NA	5	NA	NA	NA
Panc-AdenoCA	f5bb7ae4-4bb4-4e87-b83c-bc8620584989	18	64752876	64754803	12	1927	1	5	5	NA	1	NA
Lymph-BNHL	f60007b3-0603-4ad1-8b47-d2d4c4c621e4	14	106112552	106113782	6	1230	NA	2	4	NA	NA	NA
Lymph-BNHL	f60007b3-0603-4ad1-8b47-d2d4c4c621e4	14	106325787	106329165	60	3378	2	11	14	13	11	9
Lymph-BNHL	f60007b3-0603-4ad1-8b47-d2d4c4c621e4	14	106994205	106995254	13	1049	NA	NA	3	2	3	5
Lymph-BNHL	f60007b3-0603-4ad1-8b47-d2d4c4c621e4	18	60983996	60988261	34	4265	6	7	6	6	4	5
Lymph-BNHL	f60007b3-0603-4ad1-8b47-d2d4c4c621e4	22	22380014	22380927	10	913	2	NA	5	1	1	1
Lymph-BNHL	f60007b3-0603-4ad1-8b47-d2d4c4c621e4	22	23152566	23154755	11	2189	NA	2	5	1	2	1
Prost-AdenoCA	f601cf2f-081f-484d-ab0e-21a8ec8d3770	7	39270292	39275304	17	5012	NA	6	6	1	3	1
Prost-AdenoCA	f601cf2f-081f-484d-ab0e-21a8ec8d3770	11	107031811	107033714	7	1903	NA	3	4	NA	NA	NA
Breast-AdenoCa	f6114c69-71a1-47d5-9b28-b0227b1872f7	1	212748071	212748481	6	410	NA	NA	6	NA	NA	NA
Breast-AdenoCa	f6114c69-71a1-47d5-9b28-b0227b1872f7	4	57448763	57450734	10	1971	NA	5	5	NA	NA	NA
Breast-AdenoCa	f6114c69-71a1-47d5-9b28-b0227b1872f7	8	92661802	92664431	7	2629	NA	NA	7	NA	NA	NA
Breast-AdenoCa	f6114c69-71a1-47d5-9b28-b0227b1872f7	8	98129348	98134678	48	5330	2	14	29	1	1	1
Breast-AdenoCa	f6114c69-71a1-47d5-9b28-b0227b1872f7	8	98396372	98404149	41	7777	3	4	34	NA	NA	NA
Breast-AdenoCa	f6114c69-71a1-47d5-9b28-b0227b1872f7	8	98452224	98456466	14	4242	NA	2	12	NA	NA	NA
Breast-AdenoCa	f6114c69-71a1-47d5-9b28-b0227b1872f7	8	101276441	101278410	13	1969	1	7	5	NA	NA	NA
Breast-AdenoCa	f6114c69-71a1-47d5-9b28-b0227b1872f7	8	115707309	115709120	10	1811	1	4	5	NA	NA	NA
Breast-AdenoCa	f6114c69-71a1-47d5-9b28-b0227b1872f7	8	117609045	117612253	7	3208	NA	6	1	NA	NA	NA
Breast-AdenoCa	f6114c69-71a1-47d5-9b28-b0227b1872f7	8	124090049	124096890	168	6841	1	6	##	NA	NA	NA
Breast-AdenoCa	f6114c69-71a1-47d5-9b28-b0227b1872f7	8	127632028	127633662	8	1634	2	4	2	NA	NA	NA
Breast-AdenoCa	f6114c69-71a1-47d5-9b28-b0227b1872f7	8	127645915	127647287	7	1372	NA	NA	7	NA	NA	NA
Breast-AdenoCa	f6114c69-71a1-47d5-9b28-b0227b1872f7	8	128103107	128107090	8	3983	1	5	2	NA	NA	NA
Breast-AdenoCa	f6114c69-71a1-47d5-9b28-b0227b1872f7	8	136085710	136088036	12	2326	2	7	3	NA	NA	NA
Breast-AdenoCa	f6114c69-71a1-47d5-9b28-b0227b1872f7	12	92172250	92178657	10	6407	1	1	8	NA	NA	NA
Breast-AdenoCa	f6114c69-71a1-47d5-9b28-b0227b1872f7	12	97436055	97451470	22	15415	3	9	9	NA	1	NA
Breast-AdenoCa	f6114c69-71a1-47d5-9b28-b0227b1872f7	17	29331757	29339786	16	8029	NA	7	9	NA	NA	NA
Breast-AdenoCa	f6114c69-71a1-47d5-9b28-b0227b1872f7	17	38093940	38095131	11	1191	NA	3	8	NA	NA	NA
Ovary-AdenoCA	f6189828-eeaa-4d21-b163-53bf3d47a640	6	93310111	93310403	6	292	1	1	4	NA	NA	NA
Ovary-AdenoCA	f6189828-eeaa-4d21-b163-53bf3d47a640	9	38719270	38722659	14	3389	4	6	4	NA	NA	NA
Prost-AdenoCA	f640d377-98e9-41d3-8761-61eb33072c65	1	70274483	70277149	7	2666	1	1	4	NA	1	NA
Prost-AdenoCA	f640d377-98e9-41d3-8761-61eb33072c65	8	112216113	112218875	6	2762	1	NA	NA	3	2	NA
Prost-AdenoCA	f640d377-98e9-41d3-8761-61eb33072c65	8	128056636	128057546	7	910	NA	2	3	2	NA	NA
Prost-AdenoCA	f640d377-98e9-41d3-8761-61eb33072c65	X	66195485	66196776	6	1291	1	2	3	NA	NA	NA
Cervix-SCC	f658c350-fb89-4268-8a59-a07e365f4221	1	28277777	28281891	8	4114	1	4	3	NA	NA	NA
Cervix-SCC	f658c350-fb89-4268-8a59-a07e365f4221	2	54552214	54556331	6	4117	NA	2	4	NA	NA	NA
Cervix-SCC	f658c350-fb89-4268-8a59-a07e365f4221	3	171148905	171152658	11	3753	NA	3	8	NA	NA	NA
Cervix-SCC	f658c350-fb89-4268-8a59-a07e365f4221	5	76266394	76269908	7	3514	2	3	2	NA	NA	NA

Cervix-SCC	f658c350-fb89-4268-8a59-a07e365f4221	5	180146253	180150998	7	4745	NA	2	5	NA	NA	NA
Cervix-SCC	f658c350-fb89-4268-8a59-a07e365f4221	8	41784279	41787966	7	3687	1	3	3	NA	NA	NA
Cervix-SCC	f658c350-fb89-4268-8a59-a07e365f4221	10	79523772	79528061	6	4289	1	2	3	NA	NA	NA
Cervix-SCC	f658c350-fb89-4268-8a59-a07e365f4221	12	59836283	59840135	6	3852	NA	2	4	NA	NA	NA
Cervix-SCC	f658c350-fb89-4268-8a59-a07e365f4221	15	36821599	36827147	7	5548	1	1	5	NA	NA	NA
Cervix-SCC	f658c350-fb89-4268-8a59-a07e365f4221	16	1701126	1704077	8	2951	2	4	2	NA	NA	NA
Breast-AdenoCa	f69e5057-eda6-4391-ba9a-aedbf6b572da	4	60430381	60436313	7	5932	1	2	4	NA	NA	NA
Breast-AdenoCa	f69e5057-eda6-4391-ba9a-aedbf6b572da	4	61348219	61351021	12	2802	5	6	1	NA	NA	NA
Ovary-AdenoCA	f6c811ff-f22e-490b-9b23-b527d20e6e6d	1	162397578	162400702	15	3124	1	11	2	NA	NA	1
Panc-AdenoCA	f7702c0c-9636-4700-a8ae-7a58afd9fa71	8	42101919	42107076	15	5157	3	4	8	NA	NA	NA
Panc-AdenoCA	f7702c0c-9636-4700-a8ae-7a58afd9fa71	8	129674205	129679788	23	5583	NA	1	18	NA	4	NA
Panc-AdenoCA	f7702c0c-9636-4700-a8ae-7a58afd9fa71	8	130838441	130841843	12	3402	NA	NA	12	NA	NA	NA
Panc-AdenoCA	f7702c0c-9636-4700-a8ae-7a58afd9fa71	8	142077077	142080260	22	3183	1	9	12	NA	NA	NA
Panc-AdenoCA	f7702c0c-9636-4700-a8ae-7a58afd9fa71	9	22079720	22080371	6	651	1	3	2	NA	NA	NA
Panc-AdenoCA	f7702c0c-9636-4700-a8ae-7a58afd9fa71	15	50080278	50083561	6	3283	NA	1	5	NA	NA	NA
Panc-AdenoCA	f7702c0c-9636-4700-a8ae-7a58afd9fa71	19	15986833	15988724	7	1891	2	5	NA	NA	NA	NA
Panc-AdenoCA	f7702c0c-9636-4700-a8ae-7a58afd9fa71	19	17024027	17024522	14	495	NA	NA	14	NA	NA	NA
Panc-AdenoCA	f7702c0c-9636-4700-a8ae-7a58afd9fa71	22	45024769	45026625	10	1856	NA	5	5	NA	NA	NA
Breast-AdenoCa	f7b84bac-f161-9eee-e040-11ac0c4847ed	1	20093544	20097027	10	3483	1	6	3	NA	NA	NA
Breast-AdenoCa	f7b84bac-f161-9eee-e040-11ac0c4847ed	6	122483441	122487520	6	4079	3	1	2	NA	NA	NA
Breast-AdenoCa	f7b84bac-f161-9eee-e040-11ac0c4847ed	19	9900806	9901894	7	1088	1	3	3	NA	NA	NA
Prost-AdenoCA	f7cc504a-37c9-45f5-b50d-599c54dca157	10	124020193	124021661	7	1468	NA	NA	1	4	NA	2
Breast-AdenoCa	f7d70fac-c76f-399e-e040-11ac0c482f9c	8	57846193	57848375	11	2182	1	3	7	NA	NA	NA
Breast-AdenoCa	f7d70fac-c76f-399e-e040-11ac0c482f9c	9	87963650	87964617	8	967	1	3	4	NA	NA	NA
Breast-AdenoCa	f7d70fac-c76f-399e-e040-11ac0c482f9c	15	60206865	60211015	20	4150	NA	9	11	NA	NA	NA
Breast-AdenoCa	f7d70fac-c76f-399e-e040-11ac0c482f9c	16	51685554	51689804	8	4250	NA	4	4	NA	NA	NA
Breast-AdenoCa	f7e4a2a8-093d-9e36-e040-11ac0d480c7d	11	15557395	15560328	18	2933	2	13	3	NA	NA	NA
Breast-AdenoCa	f7e7b258-b4b2-e6c5-e040-11ac0d486773	3	35877013	35877243	7	230	1	6	NA	NA	NA	NA
Breast-AdenoCa	f7e7b258-b4b2-e6c5-e040-11ac0d486773	11	79617218	79618513	7	1295	2	3	2	NA	NA	NA
Breast-AdenoCa	f7e7b258-b4b2-e6c5-e040-11ac0d486773	11	91257219	91258146	7	927	1	2	4	NA	NA	NA
Breast-AdenoCa	f7e7b258-b4b2-e6c5-e040-11ac0d486773	12	132235547	132236239	6	692	1	5	NA	NA	NA	NA
Breast-AdenoCa	f7f3e156-0dde-72b9-e040-11ac0d48542c	1	39620780	39621785	7	1005	1	1	5	NA	NA	NA
Breast-AdenoCa	f7f3e156-0dde-72b9-e040-11ac0d48542c	2	2116463	2121393	9	4930	1	3	5	NA	NA	NA
Breast-AdenoCa	f7f3e156-0dde-72b9-e040-11ac0d48542c	2	178232582	178236350	14	3768	2	6	5	1	NA	NA
Breast-AdenoCa	f7f3e156-0dde-72b9-e040-11ac0d48542c	11	17222967	17227917	6	4950	NA	NA	5	NA	1	NA
Breast-AdenoCa	f7f3e156-0dde-72b9-e040-11ac0d48542c	11	17267823	17274519	8	6696	1	3	4	NA	NA	NA
Breast-AdenoCa	f7f3e156-0dde-72b9-e040-11ac0d48542c	17	26764516	26765427	7	911	NA	5	2	NA	NA	NA
Breast-AdenoCa	f7f3e156-0dde-72b9-e040-11ac0d48542c	17	28242378	28248945	12	6567	4	4	4	NA	NA	NA
Breast-AdenoCa	f7f3e156-0dde-72b9-e040-11ac0d48542c	17	62994469	62999876	10	5407	1	5	4	NA	NA	NA
Breast-AdenoCa	f7f3e156-0dde-72b9-e040-11ac0d48542c	20	29505150	29508805	7	3655	1	3	3	NA	NA	NA
Bone-Osteosarc	f82d213f-9ba5-7b6b-e040-11ac0c486882	1	87353605	87354667	6	1062	1	1	4	NA	NA	NA
Bone-Osteosarc	f82d213f-9ba5-7b6b-e040-11ac0c486882	8	2380811	2382466	6	1655	NA	2	4	NA	NA	NA
Bone-Osteosarc	f82d213f-9ba5-7b6b-e040-11ac0c486882	8	140402566	140412464	28	9898	2	6	19	1	NA	NA
Bone-Osteosarc	f82d213f-9ba5-7b6b-e040-11ac0c486882	8	142344020	142344326	8	306	1	1	6	NA	NA	NA
Bone-Osteosarc	f82d213f-9ba5-7b6b-e040-11ac0c486882	8	143115817	143116997	9	1180	1	3	5	NA	NA	NA
Bone-Osteosarc	f82d213f-9ba5-7b6b-e040-11ac0c486882	12	10618283	10618648	10	365	NA	1	9	NA	NA	NA
Bone-Osteosarc	f82d213f-bc99-5b1d-e040-11ac0c486880	2	4468919	4470238	8	1319	NA	1	7	NA	NA	NA
Bone-Osteosarc	f82d213f-bc99-5b1d-e040-11ac0c486880	5	30533893	30539172	7	5279	1	1	4	NA	1	NA
Bone-Osteosarc	f82d213f-bc99-5b1d-e040-11ac0c486880	5	39336862	39338032	8	1170	1	3	4	NA	NA	NA
Bone-Osteosarc	f82d213f-bc99-5b1d-e040-11ac0c486880	8	37886556	37887469	8	913	1	5	2	NA	NA	NA
Bone-Osteosarc	f82d213f-bc99-5b1d-e040-11ac0c486880	20	60504247	60506834	6	2587	NA	1	5	NA	NA	NA
Bone-Osteosarc	f82d213f-bc99-5b1d-e040-11ac0c486880	21	26262766	26263483	11	717	2	2	7	NA	NA	NA
Bone-Osteosarc	f82d213f-caa7-fd59-e040-11ac0d483e46	5	3427092	3431978	6	4886	2	1	3	NA	NA	NA
Bone-Osteosarc	f82d213f-caa7-fd59-e040-11ac0d483e46	5	7806116	7809129	10	3013	NA	2	8	NA	NA	NA
Bone-Osteosarc	f82d213f-caa7-fd59-e040-11ac0d483e46	5	8613034	8614207	12	1173	1	1	10	NA	NA	NA
Bone-Osteosarc	f82d213f-caa7-fd59-e040-11ac0d483e46	5	11021360	11026780	7	5420	1	1	5	NA	NA	NA
Bone-Osteosarc	f82d213f-caa7-fd59-e040-11ac0d483e46	5	15259834	15265692	7	5858	2	1	4	NA	NA	NA
Bone-Osteosarc	f82d213f-caa7-fd59-e040-11ac0d483e46	5	15809787	15810084	8	297	NA	2	6	NA	NA	NA
Bone-Osteosarc	f82d213f-caa7-fd59-e040-11ac0d483e46	5	20553927	20556713	11	2786	2	5	4	NA	NA	NA
Bone-Osteosarc	f82d213f-caa7-fd59-e040-11ac0d483e46	5	28436749	28437437	7	688	1	2	3	NA	NA	1

Bone-Osteosarc	f82d213f-caa7-fd59-e040-11ac0d483e46	5	39165251	39170698	13	5447	4	2	7	NA	NA	NA
Bone-Osteosarc	f82d213f-caa7-fd59-e040-11ac0d483e46	5	39439147	39445932	9	6785	1	NA	8	NA	NA	NA
Bone-Osteosarc	f82d213f-caa7-fd59-e040-11ac0d483e46	5	42500058	42500569	8	511	NA	2	6	NA	NA	NA
Bone-Osteosarc	f82d213f-caa7-fd59-e040-11ac0d483e46	5	42527081	42527463	7	382	1	1	5	NA	NA	NA
Bone-Osteosarc	f82d213f-caa7-fd59-e040-11ac0d483e46	5	45845318	45850642	17	5324	1	2	14	NA	NA	NA
Bone-Osteosarc	f82d213f-caa7-fd59-e040-11ac0d483e46	11	64163730	64165898	13	2168	4	2	7	NA	NA	NA
Bone-Osteosarc	f82d213f-caa7-fd59-e040-11ac0d483e46	12	6931335	6937383	9	6048	1	2	6	NA	NA	NA
Bone-Osteosarc	f82d213f-caa7-fd59-e040-11ac0d483e46	12	11839219	11843357	11	4138	1	4	6	NA	NA	NA
Bone-Osteosarc	f82d213f-caa7-fd59-e040-11ac0d483e46	12	58238340	58240751	14	2411	3	2	9	NA	NA	NA
Bone-Osteosarc	f82d213f-caa7-fd59-e040-11ac0d483e46	12	69398938	69401309	14	2371	NA	1	13	NA	NA	NA
Bone-Osteosarc	f82d213f-caa7-fd59-e040-11ac0d483e46	12	71115054	71127387	17	12333	1	6	10	NA	NA	NA
Bone-Osteosarc	f82d213f-caa7-fd59-e040-11ac0d483e46	12	71837517	71842732	11	5215	2	8	NA	NA	NA	1
Bone-Osteosarc	f82d213f-caa7-fd59-e040-11ac0d483e46	12	79646495	79650771	11	4276	NA	NA	11	NA	NA	NA
Bone-Osteosarc	f82d213f-caa7-fd59-e040-11ac0d483e46	12	90078646	90084818	16	6172	NA	6	10	NA	NA	NA
Bone-Osteosarc	f82d213f-caa7-fd59-e040-11ac0d483e46	12	90632523	90639084	9	6561	NA	3	6	NA	NA	NA
Bone-Osteosarc	f82d213f-caa7-fd59-e040-11ac0d483e46	12	127800979	127802755	12	1776	1	5	6	NA	NA	NA
Bone-Osteosarc	f82d213f-caa7-fd59-e040-11ac0d483e46	12	128349412	128349853	7	441	1	4	1	NA	NA	1
Bone-Osteosarc	f82d213f-caa7-fd59-e040-11ac0d483e46	14	106963358	106978586	17	15228	3	NA	14	NA	NA	NA
Bone-Osteosarc	f82d213f-caa7-fd59-e040-11ac0d483e46	16	62562574	62564109	7	1535	NA	3	4	NA	NA	NA
Bone-Osteosarc	f82d213f-caa7-fd59-e040-11ac0d483e46	16	87191531	87191958	10	427	NA	NA	10	NA	NA	NA
Bone-Osteosarc	f82d213f-caa7-fd59-e040-11ac0d483e46	18	2340396	2341092	6	696	NA	4	2	NA	NA	NA
Bone-Osteosarc	f82d213f-caa7-fd59-e040-11ac0d483e46	18	13012351	13015067	7	2716	1	2	4	NA	NA	NA
Bone-Osteosarc	f82d213f-caa7-fd59-e040-11ac0d483e46	18	22805797	22809918	7	4121	NA	1	6	NA	NA	NA
Bone-Osteosarc	f82d213f-caa7-fd59-e040-11ac0d483e46	X	18824554	18827936	10	3382	NA	3	7	NA	NA	NA
Bone-Osteosarc	f82d213f-caa7-fd59-e040-11ac0d483e46	X	21270589	21271270	12	681	NA	7	4	NA	NA	1
Bone-Osteosarc	f82d213f-caa7-fd59-e040-11ac0d483e46	X	41651480	41654616	12	3136	NA	7	5	NA	NA	NA
Bone-Osteosarc	f82d213f-caa7-fd59-e040-11ac0d483e46	X	48350882	48356368	14	5486	2	3	9	NA	NA	NA
Bone-Osteosarc	f82d2146-70f5-8114-e040-11ac0d483e50	1	71432634	71433625	15	991	2	2	11	NA	NA	NA
Bone-Osteosarc	f82d2146-70f5-8114-e040-11ac0d483e50	2	119631030	119632106	8	1076	1	2	5	NA	NA	NA
Bone-Osteosarc	f82d2146-70f5-8114-e040-11ac0d483e50	3	32995741	32998634	13	2893	NA	2	11	NA	NA	NA
Bone-Osteosarc	f82d2146-70f5-8114-e040-11ac0d483e50	3	158205371	158213628	30	8257	7	6	17	NA	NA	NA
Bone-Osteosarc	f82d2146-70f5-8114-e040-11ac0d483e50	4	10068358	10069139	6	781	NA	2	4	NA	NA	NA
Bone-Osteosarc	f82d2146-70f5-8114-e040-11ac0d483e50	4	32846276	32860842	21	14566	2	2	17	NA	NA	NA
Bone-Osteosarc	f82d2146-70f5-8114-e040-11ac0d483e50	4	37984810	37985247	8	437	NA	NA	8	NA	NA	NA
Bone-Osteosarc	f82d2146-70f5-8114-e040-11ac0d483e50	4	139030382	139032961	8	2579	2	1	5	NA	NA	NA
Bone-Osteosarc	f82d2146-70f5-8114-e040-11ac0d483e50	5	118171082	118171608	6	526	1	1	4	NA	NA	NA
Bone-Osteosarc	f82d2146-70f5-8114-e040-11ac0d483e50	6	11379321	11381741	8	2420	1	2	5	NA	NA	NA
Bone-Osteosarc	f82d2146-70f5-8114-e040-11ac0d483e50	6	16762217	16770488	20	8271	3	5	12	NA	NA	NA
Bone-Osteosarc	f82d2146-70f5-8114-e040-11ac0d483e50	6	18381873	18383467	22	1594	3	4	15	NA	NA	NA
Bone-Osteosarc	f82d2146-70f5-8114-e040-11ac0d483e50	8	19714076	19718638	14	4562	1	1	12	NA	NA	NA
Bone-Osteosarc	f82d2146-70f5-8114-e040-11ac0d483e50	10	1048308	1049365	7	1057	1	3	3	NA	NA	NA
Bone-Osteosarc	f82d2146-70f5-8114-e040-11ac0d483e50	12	23504733	23510018	13	5285	NA	5	7	NA	NA	1
Bone-Osteosarc	f82d2146-70f5-8114-e040-11ac0d483e50	16	76686314	76688047	10	1733	1	6	3	NA	NA	NA
Bone-Osteosarc	f82d2146-70f5-8114-e040-11ac0d483e50	19	8927963	8928495	13	532	3	4	6	NA	NA	NA
Bone-Osteosarc	f82d2146-70f5-8114-e040-11ac0d483e50	22	22912671	22922089	33	9418	2	3	28	NA	NA	NA
Bone-Osteosarc	f82d2146-70f5-8114-e040-11ac0d483e50	X	69938373	69939232	6	859	1	1	4	NA	NA	NA
Bone-Osteosarc	f82d2146-70f5-8114-e040-11ac0d483e50	X	75758206	75760071	13	1865	1	5	7	NA	NA	NA
Bone-Osteosarc	f82d2146-70f5-8114-e040-11ac0d483e50	X	77678620	77679041	11	421	2	5	4	NA	NA	NA
Bone-Osteosarc	f82d2146-70f5-8114-e040-11ac0d483e50	X	83276204	83281064	25	4860	1	6	18	NA	NA	NA
Bone-Osteosarc	f82d2146-70f5-8114-e040-11ac0d483e50	X	83682065	83682547	8	482	NA	5	3	NA	NA	NA
Bone-Osteosarc	f82d2146-70f5-8114-e040-11ac0d483e50	X	83755093	83756128	8	1035	1	5	2	NA	NA	NA
Bone-Osteosarc	f82d2146-70f5-8114-e040-11ac0d483e50	X	93721531	93723266	24	1735	3	4	17	NA	NA	NA
Bone-Osteosarc	f82d2146-70f5-8114-e040-11ac0d483e50	X	95382701	95389911	12	7210	1	4	7	NA	NA	NA
Bone-Osteosarc	f82d2146-70f5-8114-e040-11ac0d483e50	X	95417405	95418293	7	888	NA	4	3	NA	NA	NA
Bone-Osteosarc	f82d2146-70f5-8114-e040-11ac0d483e50	X	101296465	101297554	14	1089	3	3	8	NA	NA	NA
Bone-Osteosarc	f82d2146-70f5-8114-e040-11ac0d483e50	X	130102735	130104738	12	2003	3	4	5	NA	NA	NA
Bone-Osteosarc	f82d2146-70f5-8114-e040-11ac0d483e50	X	131199260	131201084	15	1824	2	1	12	NA	NA	NA
Bone-Osteosarc	f82d2146-70f5-8114-e040-11ac0d483e50	X	131256283	131268574	26	12291	6	4	15	NA	NA	1
Bone-Osteosarc	f82d2146-726a-d0e0-e040-11ac0c486888	5	1797574	1802116	7	4542	2	4	1	NA	NA	NA
Bone-Osteosarc	f82d2146-726a-d0e0-e040-11ac0c486888	11	102146448	102152388	7	5940	1	6	NA	NA	NA	NA

Bone-Osteosarc	f82d2146-726a-d0e0-e040-11ac0c486888	11	104349772	104361661	20	11889	2	8	10	NA	NA	NA
Bone-Osteosarc	f82d2146-726a-d0e0-e040-11ac0c486888	19	31628701	31638508	13	9807	4	5	4	NA	NA	NA
Bone-Osteosarc	f83f1eb8-b2c8-c3d6-e040-11ac0c48261e	2	78631589	78636751	7	5162	2	NA	1	2	NA	2
Bone-Osteosarc	f83fc777-5416-c3e9-e040-11ac0d482c8e	1	50597636	50600723	18	3087	NA	7	10	1	NA	NA
Bone-Osteosarc	f83fc777-5416-c3e9-e040-11ac0d482c8e	1	52219782	52223714	13	3932	NA	7	6	NA	NA	NA
Bone-Osteosarc	f83fc777-5416-c3e9-e040-11ac0d482c8e	1	96225482	96231426	7	5944	NA	3	3	1	NA	NA
Bone-Osteosarc	f83fc777-5416-c3e9-e040-11ac0d482c8e	2	175571946	175575156	6	3210	1	3	2	NA	NA	NA
Bone-Osteosarc	f83fc777-5416-c3e9-e040-11ac0d482c8e	4	88019121	88020704	10	1583	NA	4	6	NA	NA	NA
Bone-Osteosarc	f83fc777-5416-c3e9-e040-11ac0d482c8e	5	25730652	25731270	7	618	3	NA	4	NA	NA	NA
Bone-Osteosarc	f83fc777-5416-c3e9-e040-11ac0d482c8e	15	52378051	52380501	8	2450	NA	2	6	NA	NA	NA
Bone-Osteosarc	f83fc777-5416-c3e9-e040-11ac0d482c8e	22	32919151	32920805	6	1654	NA	4	2	NA	NA	NA
Bone-Osteosarc	f843ed5c-0336-3d33-e040-11ac0d48478c	11	134929255	134931642	8	2387	NA	7	1	NA	NA	NA
Bone-Osteosarc	f843ed5c-0336-3d33-e040-11ac0d48478c	12	70786425	70791113	7	4688	2	2	2	1	NA	NA
Bone-Osteosarc	f843ed5c-0336-3d33-e040-11ac0d48478c	19	9852796	9856182	9	3386	NA	4	5	NA	NA	NA
Bone-Osteosarc	f845ebba-8c56-b559-e040-11ac0c4863d5	1	114169226	114170119	6	893	NA	4	2	NA	NA	NA
Bone-Osteosarc	f845ebba-8c56-b559-e040-11ac0c4863d5	20	21202865	21206002	6	3137	NA	4	2	NA	NA	NA
Breast-AdenoCa	f848b66f-bd9e-4fba-afd4-eb58848d1ef4	1	41823068	41825511	6	2443	2	2	1	NA	NA	1
Bone-Osteosarc	f85397dc-e6e9-4f54-e040-11ac0d48706c	1	176399832	176403261	9	3429	NA	NA	9	NA	NA	NA
Bone-Osteosarc	f85397dc-e6e9-4f54-e040-11ac0d48706c	5	119711837	119715236	6	3399	2	3	1	NA	NA	NA
Bone-Osteosarc	f85397dc-e6e9-4f54-e040-11ac0d48706c	6	66519423	66520937	16	1514	NA	7	9	NA	NA	NA
Bone-Osteosarc	f85397dc-e6e9-4f54-e040-11ac0d48706c	6	73783089	73784046	7	957	NA	2	5	NA	NA	NA
Bone-Osteosarc	f85397dc-e6e9-4f54-e040-11ac0d48706c	7	106153256	106160425	17	7169	NA	NA	17	NA	NA	NA
Bone-Osteosarc	f85397dc-e6e9-4f54-e040-11ac0d48706c	10	72202008	72202603	8	595	1	NA	7	NA	NA	NA
Bone-Osteosarc	f85397dc-e6e9-4f54-e040-11ac0d48706c	13	25221743	25222102	12	359	2	3	7	NA	NA	NA
Bone-Osteosarc	f85397dc-e6e9-4f54-e040-11ac0d48706c	16	12642136	12646513	7	4377	1	1	5	NA	NA	NA
Bone-Osteosarc	f85397dc-e6e9-4f54-e040-11ac0d48706c	18	41615785	41615841	6	56	1	NA	5	NA	NA	NA
Bone-Osteosarc	f85397dc-e6e9-4f54-e040-11ac0d48706c	19	823755	824090	6	335	NA	1	5	NA	NA	NA
Bone-Osteosarc	f85397dc-e6e9-4f54-e040-11ac0d48706c	X	74476768	74482185	13	5417	1	7	5	NA	NA	NA
Bone-Osteosarc	f85397dc-e6e9-4f54-e040-11ac0d48706c	X	138503904	138512208	31	8304	1	6	24	NA	NA	NA
Bone-Osteosarc	f856fa85-fdb8-c0b0-e040-11ac0d480b4e	2	7070967	7074397	6	3430	1	3	2	NA	NA	NA
Bone-Osteosarc	f856fa85-fdb8-c0b0-e040-11ac0d480b4e	3	85450022	85459308	26	9286	3	9	13	NA	NA	1
Bone-Osteosarc	f856fa85-fdb8-c0b0-e040-11ac0d480b4e	7	1565068	1572273	9	7205	1	2	6	NA	NA	NA
Bone-Osteosarc	f856fa85-fdb8-c0b0-e040-11ac0d480b4e	7	21147464	21151348	27	3884	9	9	9	NA	NA	NA
Bone-Osteosarc	f856fa85-fdb8-c0b0-e040-11ac0d480b4e	8	611479	613294	7	1815	NA	5	2	NA	NA	NA
Bone-Osteosarc	f856fa85-fdb8-c0b0-e040-11ac0d480b4e	8	50701120	50706116	8	4996	2	NA	6	NA	NA	NA
Bone-Osteosarc	f856fa85-fdb8-c0b0-e040-11ac0d480b4e	11	11202744	11205556	7	2812	NA	1	6	NA	NA	NA
Bone-Osteosarc	f856fa85-fdb8-c0b0-e040-11ac0d480b4e	11	18702421	18703527	7	1106	NA	1	6	NA	NA	NA
Bone-Osteosarc	f856fa85-fdb8-c0b0-e040-11ac0d480b4e	14	23018911	23021075	8	2164	1	5	2	NA	NA	NA
Bone-Osteosarc	f856fa85-fdb8-c0b0-e040-11ac0d480b4e	16	65335656	65337654	16	1998	2	8	6	NA	NA	NA
Bone-Osteosarc	f856fa85-fdb8-c0b0-e040-11ac0d480b4e	19	15982868	15987260	6	4392	NA	3	3	NA	NA	NA
Bone-Osteosarc	f856fa85-fdb8-c0b0-e040-11ac0d480b4e	19	16007752	16009840	7	2088	NA	1	6	NA	NA	NA
Bone-Osteosarc	f856fa85-fdb8-c0b0-e040-11ac0d480b4e	19	17674541	17678083	7	3542	NA	2	5	NA	NA	NA
Bone-Osteosarc	f856fa85-fdb8-c0b0-e040-11ac0d480b4e	19	22843114	22843970	9	856	NA	5	4	NA	NA	NA
Bone-Osteosarc	f856fa85-fdb8-c0b0-e040-11ac0d480b4e	19	42040063	42042197	7	2134	2	5	NA	NA	NA	NA
Bone-Osteosarc	f856fa85-fdb8-c0b0-e040-11ac0d480b4e	22	28839469	28841468	11	1999	2	3	6	NA	NA	NA
Bone-Osteosarc	f856fa85-fdb8-c0b0-e040-11ac0d480b4e	22	37302967	37309955	18	6988	2	4	12	NA	NA	NA
Bone-Osteosarc	f856fa85-fdb8-c0b0-e040-11ac0d480b4e	Y	2893321	2893957	7	636	NA	2	5	NA	NA	NA
Bone-Osteosarc	f856fa85-fdb8-c0b0-e040-11ac0d480b4e	Y	14323293	14326240	7	2947	1	2	4	NA	NA	NA
Bone-Osteosarc	f856fa85-fdb8-c0b0-e040-11ac0d480b4e	Y	22745878	22749096	16	3218	3	6	7	NA	NA	NA
Ovary-AdenoCA	f858d813-f3c5-4ad9-8c20-9f231d6624d8	1	35060978	35063030	8	2052	NA	4	4	NA	NA	NA
Ovary-AdenoCA	f858d813-f3c5-4ad9-8c20-9f231d6624d8	1	62317902	62319741	10	1839	1	1	8	NA	NA	NA
Ovary-AdenoCA	f858d813-f3c5-4ad9-8c20-9f231d6624d8	1	68914849	68916576	12	1727	1	8	3	NA	NA	NA
Ovary-AdenoCA	f858d813-f3c5-4ad9-8c20-9f231d6624d8	3	118470655	118474837	6	4182	1	1	4	NA	NA	NA
Ovary-AdenoCA	f858d813-f3c5-4ad9-8c20-9f231d6624d8	6	63244256	63244558	9	302	3	3	3	NA	NA	NA
Ovary-AdenoCA	f858d813-f3c5-4ad9-8c20-9f231d6624d8	6	67920392	67923245	8	2853	1	5	2	NA	NA	NA
Ovary-AdenoCA	f858d813-f3c5-4ad9-8c20-9f231d6624d8	6	72625156	72626972	7	1816	NA	5	2	NA	NA	NA
Ovary-AdenoCA	f858d813-f3c5-4ad9-8c20-9f231d6624d8	7	144471618	144475454	6	3836	1	4	1	NA	NA	NA
Ovary-AdenoCA	f858d813-f3c5-4ad9-8c20-9f231d6624d8	8	35023162	35024435	9	1273	1	2	6	NA	NA	NA
Ovary-AdenoCA	f858d813-f3c5-4ad9-8c20-9f231d6624d8	8	35522081	35522494	7	413	4	1	1	NA	1	NA
Ovary-AdenoCA	f858d813-f3c5-4ad9-8c20-9f231d6624d8	8	62875075	62876205	7	1130	NA	7	NA	NA	NA	NA

Ovary-AdenoCA	f858d813-f3c5-4ad9-8c20-9f231d6624d8	10	4625148	4629567	6	4419	NA	2	3	1	NA	NA
Ovary-AdenoCA	f858d813-f3c5-4ad9-8c20-9f231d6624d8	10	6525006	6532330	23	7324	10	8	5	NA	NA	NA
Ovary-AdenoCA	f858d813-f3c5-4ad9-8c20-9f231d6624d8	10	15322840	15327911	20	5071	3	9	8	NA	NA	NA
Ovary-AdenoCA	f858d813-f3c5-4ad9-8c20-9f231d6624d8	10	15511858	15515430	13	3572	NA	7	6	NA	NA	NA
Ovary-AdenoCA	f858d813-f3c5-4ad9-8c20-9f231d6624d8	10	16254175	16254847	12	672	4	5	3	NA	NA	NA
Ovary-AdenoCA	f858d813-f3c5-4ad9-8c20-9f231d6624d8	10	21318368	21323384	10	5016	2	4	4	NA	NA	NA
Ovary-AdenoCA	f858d813-f3c5-4ad9-8c20-9f231d6624d8	10	31046052	31052655	11	6603	NA	6	4	1	NA	NA
Ovary-AdenoCA	f858d813-f3c5-4ad9-8c20-9f231d6624d8	11	63708679	63711823	10	3144	1	4	5	NA	NA	NA
Ovary-AdenoCA	f858d813-f3c5-4ad9-8c20-9f231d6624d8	11	134267119	134269235	9	2116	2	2	5	NA	NA	NA
Ovary-AdenoCA	f858d813-f3c5-4ad9-8c20-9f231d6624d8	12	9410791	9411151	6	360	NA	NA	6	NA	NA	NA
Ovary-AdenoCA	f858d813-f3c5-4ad9-8c20-9f231d6624d8	12	32766103	32774019	12	7916	5	7	NA	NA	NA	NA
Ovary-AdenoCA	f858d813-f3c5-4ad9-8c20-9f231d6624d8	12	39445520	39451858	17	6338	1	11	5	NA	NA	NA
Ovary-AdenoCA	f858d813-f3c5-4ad9-8c20-9f231d6624d8	12	43068736	43070728	11	1992	1	7	3	NA	NA	NA
Ovary-AdenoCA	f858d813-f3c5-4ad9-8c20-9f231d6624d8	20	33959042	33961930	13	2888	2	7	4	NA	NA	NA
Bone-Osteosarc	f8593ac0-9480-22a0-e040-11ac0d48697a	4	45332631	45333007	6	376	NA	3	1	1	NA	1
Bone-Osteosarc	f8593ac0-9480-22a0-e040-11ac0d48697a	21	16613291	16614971	7	1680	NA	4	3	NA	NA	NA
Bone-Osteosarc	f85add3d-e2e6-31f8-e040-11ac0d48479c	2	212884193	212887230	11	3037	1	6	4	NA	NA	NA
Bone-Osteosarc	f85add3d-e2e6-31f8-e040-11ac0d48479c	6	23755453	23757756	10	2303	1	8	1	NA	NA	NA
Bone-Osteosarc	f85add3d-e2e6-31f8-e040-11ac0d48479c	6	106610356	106611051	10	695	1	5	4	NA	NA	NA
Bone-Osteosarc	f85add3d-e2e6-31f8-e040-11ac0d48479c	10	932411	936895	8	4484	NA	1	6	1	NA	NA
Bone-Osteosarc	f85add3d-e2e6-31f8-e040-11ac0d48479c	10	946944	948355	16	1411	4	6	6	NA	NA	NA
Bone-Osteosarc	f85add3d-e2e6-31f8-e040-11ac0d48479c	13	111549956	111551055	6	1099	1	3	1	NA	NA	1
Bone-Osteosarc	f85add3d-e2e6-31f8-e040-11ac0d48479c	16	26915136	26920271	12	5135	2	4	5	NA	NA	1
Bone-Osteosarc	f85add3d-e2e6-31f8-e040-11ac0d48479c	20	22291327	22292396	11	1069	1	7	3	NA	NA	NA
Bone-Osteosarc	f85add3d-e2e6-31f8-e040-11ac0d48479c	20	24896332	24897246	13	914	2	3	8	NA	NA	NA
Bone-Osteosarc	f85add3d-e2e6-31f8-e040-11ac0d48479c	20	49378074	49379872	8	1798	2	3	3	NA	NA	NA
Bone-Osteosarc	f85add3d-e2e6-31f8-e040-11ac0d48479c	X	28191168	28192174	13	1006	4	4	5	NA	NA	NA
Bone-Osteosarc	f86975a2-78a4-ef8c-e040-11ac0c4809da	1	49638282	49643539	13	5257	NA	8	4	NA	NA	1
Bone-Osteosarc	f86975a2-78a4-ef8c-e040-11ac0c4809da	2	99109764	99122543	14	12779	2	2	10	NA	NA	NA
Bone-Osteosarc	f86975a2-78a4-ef8c-e040-11ac0c4809da	3	116610904	116612166	6	1262	1	1	4	NA	NA	NA
Bone-Osteosarc	f86975a2-78a4-ef8c-e040-11ac0c4809da	5	98771637	98774057	12	2420	5	3	4	NA	NA	NA
Bone-Osteosarc	f86975a2-78a4-ef8c-e040-11ac0c4809da	6	55687110	55687792	8	682	NA	3	5	NA	NA	NA
Bone-Osteosarc	f86975a2-78a4-ef8c-e040-11ac0c4809da	13	23987748	23994570	16	6822	2	4	10	NA	NA	NA
Bone-Osteosarc	f86975a2-78a4-ef8c-e040-11ac0c4809da	13	44467348	44467871	7	523	NA	1	6	NA	NA	NA
Bone-Osteosarc	f86975a2-78a4-ef8c-e040-11ac0c4809da	13	47193530	47194261	8	731	NA	3	5	NA	NA	NA
Bone-Osteosarc	f86975a2-78a4-ef8c-e040-11ac0c4809da	16	8917485	8921452	13	3967	3	6	3	1	NA	NA
Bone-Osteosarc	f86975a2-78a4-ef8c-e040-11ac0c4809da	16	26114215	26115764	12	1549	2	4	6	NA	NA	NA
Bone-Osteosarc	f86975a2-78a4-ef8c-e040-11ac0c4809da	17	21356966	21360788	9	3822	1	2	6	NA	NA	NA
Bone-Osteosarc	f86975a2-78a4-ef8c-e040-11ac0c4809da	18	19723534	19728355	7	4821	NA	3	4	NA	NA	NA
Bone-Osteosarc	f86975a2-78a4-ef8c-e040-11ac0c4809da	X	72675521	72677189	15	1668	6	3	6	NA	NA	NA
Bone-Osteosarc	f86975a2-78a4-ef8c-e040-11ac0c4809da	X	96327135	96327937	10	802	1	3	6	NA	NA	NA
Bone-Osteosarc	f86975a2-78a4-ef8c-e040-11ac0c4809da	X	142112030	142117057	20	5027	6	6	8	NA	NA	NA
Bone-Osteosarc	f86ae246-2492-9785-e040-11ac0d4813b0	2	55069650	55071072	17	1422	NA	NA	17	NA	NA	NA
Bone-Osteosarc	f86ae246-2492-9785-e040-11ac0d4813b0	5	10017008	10019203	9	2195	NA	NA	9	NA	NA	NA
Bone-Osteosarc	f86ae246-2492-9785-e040-11ac0d4813b0	5	10063830	10065956	8	2126	NA	NA	8	NA	NA	NA
Bone-Osteosarc	f86ae246-2492-9785-e040-11ac0d4813b0	7	114955368	114959478	6	4110	2	NA	4	NA	NA	NA
Bone-Osteosarc	f86ae246-2492-9785-e040-11ac0d4813b0	7	148085092	148089847	12	4755	1	7	4	NA	NA	NA
Bone-Osteosarc	f86ae246-2492-9785-e040-11ac0d4813b0	8	53974488	53975115	6	627	NA	NA	6	NA	NA	NA
Bone-Osteosarc	f86ae246-2492-9785-e040-11ac0d4813b0	8	130951354	130951578	7	224	NA	NA	7	NA	NA	NA
Bone-Osteosarc	f86ae246-2492-9785-e040-11ac0d4813b0	8	134748144	134752247	7	4103	NA	1	6	NA	NA	NA
Bone-Osteosarc	f86ae246-2492-9785-e040-11ac0d4813b0	8	141183441	141188558	11	5117	NA	4	7	NA	NA	NA
Bone-Osteosarc	f86ae246-2492-9785-e040-11ac0d4813b0	8	142250077	142255678	8	5601	1	4	3	NA	NA	NA
Bone-Osteosarc	f86ae246-2492-9785-e040-11ac0d4813b0	11	98367115	98368946	7	1831	1	2	4	NA	NA	NA
Bone-Osteosarc	f86ae246-2492-9785-e040-11ac0d4813b0	13	27166909	27167770	10	861	2	NA	8	NA	NA	NA
Bone-Osteosarc	f86ae246-2492-9785-e040-11ac0d4813b0	13	30511689	30512272	16	583	NA	NA	16	NA	NA	NA
Bone-Osteosarc	f86ae246-2492-9785-e040-11ac0d4813b0	13	52295777	52297227	16	1450	NA	NA	16	NA	NA	NA
Bone-Osteosarc	f86ae246-2492-9785-e040-11ac0d4813b0	13	75252056	75252819	7	763	NA	2	5	NA	NA	NA
Bone-Osteosarc	f86ae246-2492-9785-e040-11ac0d4813b0	17	20446577	20451492	26	4915	1	NA	25	NA	NA	NA
Bone-Osteosarc	f86ae246-2492-9785-e040-11ac0d4813b0	17	21294918	21299228	23	4310	NA	NA	23	NA	NA	NA
Bone-Osteosarc	f86ae246-2492-9785-e040-11ac0d4813b0	17	73617590	73620709	8	3119	1	2	5	NA	NA	NA

Bone-Osteosarc	f86ae246-2492-9785-e040-11ac0d4813b0	X	55761372	55763135	9	1763	2	5	2	NA	NA	NA
Bone-Osteosarc	f86ae246-2492-9785-e040-11ac0d4813b0	Y	17330883	17332138	6	1255	NA	5	1	NA	NA	NA
Bone-Osteosarc	f86b7e84-7040-c751-e040-11ac0c485675	2	102609608	102611737	8	2129	3	4	1	NA	NA	NA
Bone-Osteosarc	f86b7e84-7040-c751-e040-11ac0c485675	3	179880377	179882372	7	1995	1	2	4	NA	NA	NA
Bone-Osteosarc	f86b7e84-7040-c751-e040-11ac0c485675	4	133829268	133834194	9	4926	1	1	7	NA	NA	NA
Bone-Osteosarc	f86b7e84-7040-c751-e040-11ac0c485675	6	1391862	1396706	6	4844	NA	NA	6	NA	NA	NA
Bone-Osteosarc	f86b7e84-7040-c751-e040-11ac0c485675	6	1397971	1402341	16	4370	NA	1	15	NA	NA	NA
Bone-Osteosarc	f86b7e84-7040-c751-e040-11ac0c485675	6	2545118	2553296	23	8178	NA	NA	23	NA	NA	NA
Bone-Osteosarc	f86b7e84-7040-c751-e040-11ac0c485675	6	22690749	22691427	14	678	NA	1	13	NA	NA	NA
Bone-Osteosarc	f86b7e84-7040-c751-e040-11ac0c485675	6	28647875	28652711	22	4836	NA	NA	22	NA	NA	NA
Bone-Osteosarc	f86b7e84-7040-c751-e040-11ac0c485675	6	29177251	29181334	32	4083	NA	NA	32	NA	NA	NA
Bone-Osteosarc	f86b7e84-7040-c751-e040-11ac0c485675	6	29226563	29230711	23	4148	NA	NA	23	NA	NA	NA
Bone-Osteosarc	f86b7e84-7040-c751-e040-11ac0c485675	6	51564493	51565971	11	1478	2	6	2	NA	1	NA
Bone-Osteosarc	f86b7e84-7040-c751-e040-11ac0c485675	12	23113545	23114850	7	1305	NA	3	3	NA	NA	1
Bone-Osteosarc	f86b7e84-7040-c751-e040-11ac0c485675	16	504443	507161	7	2718	2	1	4	NA	NA	NA
Bone-Osteosarc	f86b7e84-7040-c751-e040-11ac0c485675	16	29223488	29224997	7	1509	1	3	3	NA	NA	NA
Bone-Osteosarc	f86b7e84-7040-c751-e040-11ac0c485675	18	3027329	3028894	8	1565	NA	3	5	NA	NA	NA
Bone-Osteosarc	f86b7e84-7040-c751-e040-11ac0c485675	18	9729421	9730141	7	720	NA	5	2	NA	NA	NA
Bone-Osteosarc	f86b7e84-7040-c751-e040-11ac0c485675	18	19407744	19414898	18	7154	NA	9	8	1	NA	NA
Bone-Osteosarc	f86b7e84-7040-c751-e040-11ac0c485675	19	52353213	52353681	6	468	NA	2	4	NA	NA	NA
Bone-Osteosarc	f86b7e84-7040-c751-e040-11ac0c485675	22	28979575	28981738	16	2163	NA	NA	16	NA	NA	NA
Bone-Osteosarc	f86e2d80-911b-7a19-e040-11ac0d486900	3	89576840	89580222	13	3382	1	5	7	NA	NA	NA
Bone-Osteosarc	f86e2d80-911b-7a19-e040-11ac0d486900	3	89893322	89897221	8	3899	NA	6	1	NA	NA	1
Bone-Osteosarc	f86e2d80-911b-7a19-e040-11ac0d486900	4	184718036	184718704	6	668	NA	NA	6	NA	NA	NA
Bone-Osteosarc	f86e2d80-911b-7a19-e040-11ac0d486900	12	40155800	40156623	7	823	NA	4	3	NA	NA	NA
Bone-Osteosarc	f86e2d80-911b-7a19-e040-11ac0d486900	17	20559116	20561936	6	2820	NA	2	3	1	NA	NA
Bone-Osteosarc	f86e2d80-911b-7a19-e040-11ac0d486900	Y	16777706	16779218	6	1512	1	1	4	NA	NA	NA
Liver-HCC	f8733b80-c622-11e3-bf01-24c6515278c0	1	174809156	174809484	8	328	1	2	5	NA	NA	NA
Liver-HCC	f8733b80-c622-11e3-bf01-24c6515278c0	1	199151679	199153395	7	1716	3	1	3	NA	NA	NA
Liver-HCC	f8733b80-c622-11e3-bf01-24c6515278c0	4	63880003	63881347	6	1344	2	3	1	NA	NA	NA
Liver-HCC	f8733b80-c622-11e3-bf01-24c6515278c0	5	100746002	100746986	8	984	1	NA	NA	2	1	4
Liver-HCC	f8733b80-c622-11e3-bf01-24c6515278c0	12	31162028	31162787	13	759	NA	4	9	NA	NA	NA
Liver-HCC	f8733b80-c622-11e3-bf01-24c6515278c0	13	104165339	104167369	7	2030	NA	NA	3	3	1	NA
Bone-Osteosarc	f87348df-7186-4c6b-e040-11ac0c482ba9	1	202824830	202832676	13	7846	2	4	6	NA	1	NA
Bone-Osteosarc	f87348df-7186-4c6b-e040-11ac0c482ba9	1	203106446	203111449	26	5003	12	8	5	NA	NA	1
Bone-Osteosarc	f87348df-7186-4c6b-e040-11ac0c482ba9	2	41294569	41300120	7	5551	1	NA	6	NA	NA	NA
Bone-Osteosarc	f87348df-7186-4c6b-e040-11ac0c482ba9	2	41308215	41313098	9	4883	NA	2	7	NA	NA	NA
Bone-Osteosarc	f87348df-7186-4c6b-e040-11ac0c482ba9	2	168989671	168995607	11	5936	1	4	6	NA	NA	NA
Bone-Osteosarc	f87348df-7186-4c6b-e040-11ac0c482ba9	3	42482473	42486916	11	4443	6	2	2	NA	NA	1
Bone-Osteosarc	f87348df-7186-4c6b-e040-11ac0c482ba9	6	91404918	91409462	7	4544	1	6	NA	NA	NA	NA
Bone-Osteosarc	f87348df-7186-4c6b-e040-11ac0c482ba9	10	67274177	67278087	10	3910	5	NA	5	NA	NA	NA
Bone-Osteosarc	f87348df-7186-4c6b-e040-11ac0c482ba9	20	47908116	47908707	8	591	NA	6	2	NA	NA	NA
Bone-Osteosarc	f87d7c27-eeef-920e-e040-11ac0d48388d	3	80399037	80402062	9	3025	NA	5	4	NA	NA	NA
Bone-Osteosarc	f87d7c27-eeef-920e-e040-11ac0d48388d	3	80603449	80603975	7	526	NA	4	3	NA	NA	NA
Bone-Osteosarc	f87d7c27-eeef-920e-e040-11ac0d48388d	3	82710613	82714104	9	3491	1	3	5	NA	NA	NA
Bone-Osteosarc	f87d7c27-eeef-920e-e040-11ac0d48388d	3	82730035	82732401	8	2366	NA	3	5	NA	NA	NA
Bone-Osteosarc	f87d7c27-eeef-920e-e040-11ac0d48388d	4	65953167	65953474	6	307	1	4	1	NA	NA	NA
Bone-Osteosarc	f87d7c27-eeef-920e-e040-11ac0d48388d	6	64814951	64820737	16	5786	1	7	8	NA	NA	NA
Bone-Osteosarc	f87d7c27-eeef-920e-e040-11ac0d48388d	6	65637613	65638775	7	1162	NA	4	3	NA	NA	NA
Bone-Osteosarc	f87d7c27-eeef-920e-e040-11ac0d48388d	7	35717941	35719264	10	1323	1	5	4	NA	NA	NA
Bone-Osteosarc	f87d7c27-eeef-920e-e040-11ac0d48388d	8	105614540	105619398	11	4858	3	3	5	NA	NA	NA
Bone-Osteosarc	f87d7c27-eeef-920e-e040-11ac0d48388d	8	127773344	127774076	7	732	1	4	2	NA	NA	NA
Bone-Osteosarc	f87d7c27-eeef-920e-e040-11ac0d48388d	8	132817604	132818901	10	1297	NA	7	3	NA	NA	NA
Bone-Osteosarc	f87d7c27-eeef-920e-e040-11ac0d48388d	11	17739450	17741086	6	1636	NA	3	3	NA	NA	NA
Bone-Osteosarc	f87d7c27-eeef-920e-e040-11ac0d48388d	11	35901735	35904753	16	3018	3	5	8	NA	NA	NA
Bone-Osteosarc	f87d7c27-eeef-920e-e040-11ac0d48388d	12	38831594	38832073	7	479	1	NA	6	NA	NA	NA
Bone-Osteosarc	f87d7c27-eeef-920e-e040-11ac0d48388d	12	65810827	65811365	9	538	2	2	5	NA	NA	NA
Bone-Osteosarc	f87d7c27-eeef-920e-e040-11ac0d48388d	12	66146964	66152243	8	5279	NA	3	5	NA	NA	NA
Bone-Osteosarc	f87d7c27-eeef-920e-e040-11ac0d48388d	12	66744322	66745977	26	1655	5	5	16	NA	NA	NA
Bone-Osteosarc	f87d7c27-eeef-920e-e040-11ac0d48388d	12	66807026	66808595	7	1569	NA	5	2	NA	NA	NA

Bone-Osteosarc	f87d7c27-eeef-920e-e040-11ac0d48388d	12	80973050	80974000	7	950	NA	1	6	NA	NA	NA
Bone-Osteosarc	f87d7c27-eeef-920e-e040-11ac0d48388d	12	83029719	83031125	10	1406	NA	5	5	NA	NA	NA
Bone-Osteosarc	f87d7c27-eeef-920e-e040-11ac0d48388d	12	86372801	86388330	20	15529	NA	3	16	NA	NA	1
Bone-Osteosarc	f87d7c27-eeef-920e-e040-11ac0d48388d	12	86632868	86633367	7	499	2	1	4	NA	NA	NA
Bone-Osteosarc	f87d7c27-eeef-920e-e040-11ac0d48388d	12	87122427	87125269	11	2842	2	6	3	NA	NA	NA
Bone-Osteosarc	f87d7c27-eeef-920e-e040-11ac0d48388d	12	87685269	87724661	47	39392	6	13	27	NA	1	NA
Bone-Osteosarc	f87d7c27-eeef-920e-e040-11ac0d48388d	12	88445843	88446503	15	660	2	6	7	NA	NA	NA
Bone-Osteosarc	f87d7c27-eeef-920e-e040-11ac0d48388d	12	89953667	89954316	11	649	2	2	6	1	NA	NA
Bone-Osteosarc	f87d7c27-eeef-920e-e040-11ac0d48388d	12	91275563	91276522	8	959	NA	4	4	NA	NA	NA
Bone-Osteosarc	f87d7c27-eeef-920e-e040-11ac0d48388d	12	91931438	91938083	8	6645	NA	6	2	NA	NA	NA
Bone-Osteosarc	f87d7c27-eeef-920e-e040-11ac0d48388d	12	92138809	92140221	24	1412	4	4	16	NA	NA	NA
Bone-Osteosarc	f87d7c27-eeef-920e-e040-11ac0d48388d	12	92865355	92870293	29	4938	5	15	7	1	NA	1
Bone-Osteosarc	f87d7c27-eeef-920e-e040-11ac0d48388d	12	97657765	97659001	9	1236	2	1	6	NA	NA	NA
Bone-Osteosarc	f87d7c27-eeef-920e-e040-11ac0d48388d	12	97670981	97672818	8	1837	NA	NA	8	NA	NA	NA
Bone-Osteosarc	f87d7c27-eeef-920e-e040-11ac0d48388d	12	98245121	98245922	8	801	2	4	2	NA	NA	NA
Bone-Osteosarc	f87d7c27-eeef-920e-e040-11ac0d48388d	12	98331766	98334385	13	2619	1	3	9	NA	NA	NA
Bone-Osteosarc	f87d7c27-eeef-920e-e040-11ac0d48388d	12	109248563	109259277	19	10714	3	3	13	NA	NA	NA
Bone-Osteosarc	f87d7c27-eeef-920e-e040-11ac0d48388d	12	113727030	113727698	9	668	1	NA	8	NA	NA	NA
Bone-Osteosarc	f87d7c27-eeef-920e-e040-11ac0d48388d	15	58314866	58316107	9	1241	NA	1	8	NA	NA	NA
Bone-Osteosarc	f87d7c27-eeef-920e-e040-11ac0d48388d	15	87722088	87726329	26	4241	2	15	9	NA	NA	NA
Bone-Osteosarc	f87d7c27-eeef-920e-e040-11ac0d48388d	17	8703682	8704908	6	1226	NA	4	2	NA	NA	NA
Bone-Osteosarc	f87d7c27-eeef-920e-e040-11ac0d48388d	17	10738939	10740344	10	1405	1	4	5	NA	NA	NA
Bone-Osteosarc	f87d7c27-eeef-920e-e040-11ac0d48388d	17	12768551	12772569	15	4018	2	9	4	NA	NA	NA
Bone-Osteosarc	f87d7c27-eeef-920e-e040-11ac0d48388d	17	19309457	19311005	11	1548	1	1	9	NA	NA	NA
Bone-Osteosarc	f87d7c27-eeef-920e-e040-11ac0d48388d	19	20357459	20358111	6	652	NA	1	5	NA	NA	NA
Bone-Osteosarc	f87d7c27-eeef-920e-e040-11ac0d48388d	19	23671055	23673121	7	2066	1	4	2	NA	NA	NA
Bone-Osteosarc	f87d7c27-eeef-920e-e040-11ac0d48388d	X	65770115	65771890	8	1775	NA	NA	8	NA	NA	NA
Bone-Osteosarc	f87eb1b5-1712-ca1f-e040-11ac0c483848	4	4596189	4597430	14	1241	3	5	6	NA	NA	NA
Bone-Osteosarc	f87eb1b5-1712-ca1f-e040-11ac0c483848	9	21936556	21943574	9	7018	1	5	3	NA	NA	NA
Bone-Osteosarc	f87eb1b5-1712-ca1f-e040-11ac0c483848	16	65043650	65054454	17	10804	NA	9	6	NA	NA	2
Myeloid-MPN	f8e61a02-8c9e-aaee-e040-11ac0d481b6a	12	66350891	66353363	6	2472	NA	NA	NA	5	1	NA
Breast-AdenoCa	f8f749b7-547d-49fa-9da2-44eed962b6fd	1	23564882	23571837	10	6955	1	3	6	NA	NA	NA
Breast-AdenoCa	f8f749b7-547d-49fa-9da2-44eed962b6fd	1	51478692	51483573	10	4881	NA	3	7	NA	NA	NA
Breast-AdenoCa	f8f749b7-547d-49fa-9da2-44eed962b6fd	1	115398986	115404939	7	5953	1	2	4	NA	NA	NA
Breast-AdenoCa	f8f749b7-547d-49fa-9da2-44eed962b6fd	3	18950093	18954849	7	4756	NA	3	3	NA	NA	1
Breast-AdenoCa	f8f749b7-547d-49fa-9da2-44eed962b6fd	4	38935148	38941206	11	6058	NA	4	7	NA	NA	NA
Breast-AdenoCa	f8f749b7-547d-49fa-9da2-44eed962b6fd	4	97794543	97797525	9	2982	2	3	4	NA	NA	NA
Breast-AdenoCa	f8f749b7-547d-49fa-9da2-44eed962b6fd	4	175492644	175496121	8	3477	1	3	4	NA	NA	NA
Breast-AdenoCa	f8f749b7-547d-49fa-9da2-44eed962b6fd	6	24864651	24870295	9	5644	NA	5	4	NA	NA	NA
Breast-AdenoCa	f8f749b7-547d-49fa-9da2-44eed962b6fd	6	65847891	65848132	7	241	NA	3	4	NA	NA	NA
Breast-AdenoCa	f8f749b7-547d-49fa-9da2-44eed962b6fd	9	97658180	97663004	6	4824	2	1	3	NA	NA	NA
Breast-AdenoCa	f8f749b7-547d-49fa-9da2-44eed962b6fd	11	69884007	69887293	6	3286	NA	2	4	NA	NA	NA
Breast-AdenoCa	f8f749b7-547d-49fa-9da2-44eed962b6fd	12	111726668	111729770	8	3102	NA	5	3	NA	NA	NA
Breast-AdenoCa	f8f749b7-547d-49fa-9da2-44eed962b6fd	14	39360336	39371162	24	10826	4	5	15	NA	NA	NA
Breast-AdenoCa	f8f749b7-547d-49fa-9da2-44eed962b6fd	14	96669938	96682225	16	12287	6	6	4	NA	NA	NA
Breast-AdenoCa	f8f749b7-547d-49fa-9da2-44eed962b6fd	19	49408958	49413690	6	4732	2	1	3	NA	NA	NA
Panc-AdenoCA	f94c4f69-8119-4eaf-97c1-5106890c14d4	X	33713622	33717880	10	4258	NA	6	4	NA	NA	NA
Lymph-BNHL	f9837a56-7244-4846-a63d-266e6a92f168	2	89158166	89161339	16	3173	NA	5	7	2	2	NA
Lymph-BNHL	f9837a56-7244-4846-a63d-266e6a92f168	14	106211818	106213665	18	1847	1	2	15	NA	NA	NA
Lymph-BNHL	f9837a56-7244-4846-a63d-266e6a92f168	14	106236367	106241367	34	5000	3	5	26	NA	NA	NA
Lymph-BNHL	f9837a56-7244-4846-a63d-266e6a92f168	14	106323657	106330788	64	7131	4	13	39	6	NA	2
Kidney-RCC	f9a81200-5381-496a-8062-099f9e793618	3	83857223	83859946	11	2723	3	5	3	NA	NA	NA
Prost-AdenoCA	f9c0a0d9-5af6-4476-e040-11ac0d4830d3	1	189561007	189561575	6	568	NA	2	4	NA	NA	NA
Prost-AdenoCA	f9c0a0d9-5af6-4476-e040-11ac0d4830d3	2	155274579	155279418	7	4839	NA	1	6	NA	NA	NA
Prost-AdenoCA	f9c26646-d2b0-cf30-e040-11ac0d483918	4	127490449	127490890	8	441	NA	7	1	NA	NA	NA
Prost-AdenoCA	f9c26646-d2b0-cf30-e040-11ac0d483918	4	130242836	130243275	7	439	1	2	4	NA	NA	NA
Prost-AdenoCA	f9c26646-d2b0-cf30-e040-11ac0d483918	7	123703707	123706891	6	3184	NA	3	3	NA	NA	NA
Prost-AdenoCA	f9c26646-d2b0-cf30-e040-11ac0d483918	8	50604195	50608498	7	4303	1	1	5	NA	NA	NA
Prost-AdenoCA	f9c26646-d2b0-cf30-e040-11ac0d483918	12	5202907	5203861	8	954	NA	3	4	1	NA	NA
Prost-AdenoCA	f9c26646-d2b0-cf30-e040-11ac0d483918	18	882778	893427	14	10649	1	8	5	NA	NA	NA

Prost-AdenoCA	f9c3bc8e-dbc4-1ed0-e040-11ac0d4803a9	17	50054465	50057108	26	2643	NA	18	8	NA	NA	NA
Prost-AdenoCA	f9c3eaad-a0d9-8bf8-e040-11ac0d481d8e	1	75400303	75405829	20	5526	1	12	6	1	NA	NA
Prost-AdenoCA	f9c3eaad-a0d9-8bf8-e040-11ac0d481d8e	2	53173378	53175548	8	2170	NA	5	3	NA	NA	NA
Prost-AdenoCA	f9c3eaad-a0d9-8bf8-e040-11ac0d481d8e	4	180672607	180675115	8	2508	NA	5	2	NA	1	NA
Prost-AdenoCA	f9c650e7-9053-78eb-e040-11ac0d4874bb	1	238321024	238324485	15	3461	3	9	3	NA	NA	NA
Prost-AdenoCA	f9c650e7-9053-78eb-e040-11ac0d4874bb	1	245861126	245864167	9	3041	2	7	NA	NA	NA	NA
Prost-AdenoCA	f9c70e38-dd99-3fe2-e040-11ac0d4862f2	1	205335684	205336083	7	399	NA	3	4	NA	NA	NA
Lung-AdenoCA	fa2c3620-7e82-4b12-b135-87986f0f2890	8	39394220	39395208	16	988	3	6	7	NA	NA	NA
Lung-AdenoCA	fa2c3620-7e82-4b12-b135-87986f0f2890	8	77591255	77596640	7	5385	NA	3	4	NA	NA	NA
Lung-AdenoCA	fa2c3620-7e82-4b12-b135-87986f0f2890	8	111856302	111856556	7	254	NA	1	5	NA	1	NA
Lung-AdenoCA	fa2c3620-7e82-4b12-b135-87986f0f2890	8	113298890	113299812	12	922	2	2	8	NA	NA	NA
Lung-AdenoCA	fa2c3620-7e82-4b12-b135-87986f0f2890	8	115139359	115139820	9	461	3	3	3	NA	NA	NA
Lung-AdenoCA	fa2c3620-7e82-4b12-b135-87986f0f2890	8	137992634	137993269	10	635	1	3	6	NA	NA	NA
Lung-AdenoCA	fa2c3620-7e82-4b12-b135-87986f0f2890	8	138026013	138026397	9	384	NA	3	6	NA	NA	NA
Lung-AdenoCA	fa2c3620-7e82-4b12-b135-87986f0f2890	20	249404	253629	21	4225	2	10	9	NA	NA	NA
Lymph-BNHL	fa676301-902f-473f-8313-5bff34ae549a	2	89155725	89185693	187	29968	8	23	53	38	35	30
Lymph-BNHL	fa676301-902f-473f-8313-5bff34ae549a	3	187461833	187464034	16	2201	3	3	3	5	1	1
Lymph-BNHL	fa676301-902f-473f-8313-5bff34ae549a	4	34971102	34976641	8	5539	NA	NA	1	3	NA	4
Lymph-BNHL	fa676301-902f-473f-8313-5bff34ae549a	4	150358745	150366952	10	8207	1	NA	NA	6	1	2
Lymph-BNHL	fa676301-902f-473f-8313-5bff34ae549a	5	146612119	146612265	6	146	NA	NA	NA	NA	4	2
Lymph-BNHL	fa676301-902f-473f-8313-5bff34ae549a	6	19492824	19493062	6	238	1	2	1	1	NA	1
Lymph-BNHL	fa676301-902f-473f-8313-5bff34ae549a	6	101103386	101109972	11	6586	NA	NA	2	3	2	4
Lymph-BNHL	fa676301-902f-473f-8313-5bff34ae549a	7	110658512	110665976	10	7464	1	1	1	4	2	1
Lymph-BNHL	fa676301-902f-473f-8313-5bff34ae549a	9	37025361	37026786	7	1425	NA	2	5	NA	NA	NA
Lymph-BNHL	fa676301-902f-473f-8313-5bff34ae549a	9	112811392	112813240	7	1848	1	2	4	NA	NA	NA
Lymph-BNHL	fa676301-902f-473f-8313-5bff34ae549a	10	63809033	63813066	6	4033	1	2	3	NA	NA	NA
Lymph-BNHL	fa676301-902f-473f-8313-5bff34ae549a	11	21148767	21151927	6	3160	NA	NA	NA	2	2	2
Lymph-BNHL	fa676301-902f-473f-8313-5bff34ae549a	14	30046443	30055542	11	9099	3	NA	5	1	NA	2
Lymph-BNHL	fa676301-902f-473f-8313-5bff34ae549a	14	106067826	106144789	94	76963	24	10	56	1	1	2
Lymph-BNHL	fa676301-902f-473f-8313-5bff34ae549a	14	106238334	106239095	7	761	3	1	3	NA	NA	NA
Lymph-BNHL	fa676301-902f-473f-8313-5bff34ae549a	14	106327255	106378032	83	50777	10	16	28	11	8	10
Lymph-BNHL	fa676301-902f-473f-8313-5bff34ae549a	14	107170375	107178515	23	8140	NA	4	6	7	4	2
Lymph-BNHL	fa676301-902f-473f-8313-5bff34ae549a	16	10971514	10974102	31	2588	5	4	22	NA	NA	NA
Lymph-BNHL	fa676301-902f-473f-8313-5bff34ae549a	16	11348059	11349536	9	1477	1	2	6	NA	NA	NA
Lymph-BNHL	fa676301-902f-473f-8313-5bff34ae549a	16	27325860	27327276	13	1416	2	2	9	NA	NA	NA
Lymph-BNHL	fa676301-902f-473f-8313-5bff34ae549a	22	23222988	23231148	42	8160	6	7	13	6	5	5
Lymph-BNHL	fa676301-902f-473f-8313-5bff34ae549a	X	33145287	33146531	17	1244	3	3	6	1	4	NA
Lung-AdenoCA	fa6a60f5-8949-4e01-9435-d3117601627f	10	23984617	23988262	11	3645	1	4	6	NA	NA	NA
Lung-AdenoCA	fa6a60f5-8949-4e01-9435-d3117601627f	12	24456151	24457019	11	868	NA	3	8	NA	NA	NA
Lung-AdenoCA	fa6a60f5-8949-4e01-9435-d3117601627f	15	91642705	91643787	7	1082	NA	1	6	NA	NA	NA
Lung-AdenoCA	fa8844f8-c4b6-487a-8187-e30c12a7a453	6	56989167	56990258	7	1091	3	3	1	NA	NA	NA
Lung-AdenoCA	fa8844f8-c4b6-487a-8187-e30c12a7a453	10	53709357	53710053	8	696	NA	3	5	NA	NA	NA
Lung-AdenoCA	fa8844f8-c4b6-487a-8187-e30c12a7a453	17	11977560	11983011	9	5451	NA	NA	NA	3	5	1
Eso-AdenoCa	fab0be4b-c84a-45cd-a76f-44d9a8bf1846	1	246309700	246310330	10	630	1	3	6	NA	NA	NA
Eso-AdenoCa	fab0be4b-c84a-45cd-a76f-44d9a8bf1846	3	9436070	9440371	11	4301	1	4	6	NA	NA	NA
Eso-AdenoCa	fab0be4b-c84a-45cd-a76f-44d9a8bf1846	7	154752831	154754529	7	1698	1	1	5	NA	NA	NA
Eso-AdenoCa	fab0be4b-c84a-45cd-a76f-44d9a8bf1846	11	102557285	102559308	26	2023	3	6	17	NA	NA	NA
Eso-AdenoCa	fab0be4b-c84a-45cd-a76f-44d9a8bf1846	13	113262796	113268747	8	5951	1	1	4	NA	NA	2
Eso-AdenoCa	fab0be4b-c84a-45cd-a76f-44d9a8bf1846	14	19041865	19044106	6	2241	1	1	4	NA	NA	NA
Eso-AdenoCa	fab0be4b-c84a-45cd-a76f-44d9a8bf1846	17	18887896	18892890	7	4994	2	3	2	NA	NA	NA
Eso-AdenoCa	fab0be4b-c84a-45cd-a76f-44d9a8bf1846	17	32010567	32015898	8	5331	NA	NA	7	NA	NA	1
Eso-AdenoCa	fab0be4b-c84a-45cd-a76f-44d9a8bf1846	X	22665308	22667201	7	1893	2	NA	5	NA	NA	NA
Lung-SCC	fab3d6f4-de1c-4d6c-bdea-ac39a7884657	3	142560499	142567170	31	6671	2	9	20	NA	NA	NA
Lung-SCC	fab3d6f4-de1c-4d6c-bdea-ac39a7884657	15	71592137	71599099	15	6962	4	2	9	NA	NA	NA
Liver-HCC	fabf1f6c-c622-11e3-bf01-24c6515278c0	4	168057693	168059295	6	1602	2	3	1	NA	NA	NA
ColoRect-AdenoCA	faff4626-615b-416a-b7a6-9d177dcc94a9	3	69677837	69679310	10	1473	1	8	1	NA	NA	NA
ColoRect-AdenoCA	faff4626-615b-416a-b7a6-9d177dcc94a9	20	54271516	54271909	7	393	1	6	NA	NA	NA	NA
ColoRect-AdenoCA	faff4626-615b-416a-b7a6-9d177dcc94a9	20	57738289	57740123	7	1834	1	3	2	NA	1	NA
Lymph-CLL	fb74cca4-ac9c-4be0-80e3-e71592f96aae	14	106324760	106329920	30	5160	1	6	16	3	3	1
Lymph-CLL	fb74cca4-ac9c-4be0-80e3-e71592f96aae	22	23161926	23162221	7	295	1	1	3	1	NA	1

Head-SCC	fb8b5ff5-4164-4276-b75b-b0ee6bda7329	1	28414749	28415790	6	1041	NA	4	2	NA	NA	NA
Head-SCC	fb8b5ff5-4164-4276-b75b-b0ee6bda7329	2	28828906	28832563	6	3657	NA	4	2	NA	NA	NA
Head-SCC	fb8b5ff5-4164-4276-b75b-b0ee6bda7329	4	151429560	151431901	7	2341	1	3	3	NA	NA	NA
Head-SCC	fb8b5ff5-4164-4276-b75b-b0ee6bda7329	7	43667013	43673622	8	6609	NA	5	3	NA	NA	NA
Head-SCC	fb8b5ff5-4164-4276-b75b-b0ee6bda7329	10	73639056	73642521	8	3465	NA	6	2	NA	NA	NA
Head-SCC	fb8b5ff5-4164-4276-b75b-b0ee6bda7329	12	122004552	122008348	12	3796	1	NA	10	NA	NA	1
Head-SCC	fb8b5ff5-4164-4276-b75b-b0ee6bda7329	18	9964297	9967712	8	3415	NA	2	6	NA	NA	NA
Head-SCC	fb8b5ff5-4164-4276-b75b-b0ee6bda7329	18	34248985	34251181	7	2196	1	1	5	NA	NA	NA
Head-SCC	fb8b5ff5-4164-4276-b75b-b0ee6bda7329	19	1095918	1099032	6	3114	1	1	4	NA	NA	NA
Head-SCC	fb8b5ff5-4164-4276-b75b-b0ee6bda7329	19	29348140	29352629	11	4489	5	3	3	NA	NA	NA
Head-SCC	fb8b5ff5-4164-4276-b75b-b0ee6bda7329	20	23130235	23131223	6	988	NA	3	3	NA	NA	NA
Breast-AdenoCa	fb9135d9-2acf-41dd-8552-359e6b8c9470	3	50156620	50157633	6	1013	2	2	2	NA	NA	NA
Breast-AdenoCa	fb9135d9-2acf-41dd-8552-359e6b8c9470	3	54930479	54932577	7	2098	2	3	2	NA	NA	NA
Breast-AdenoCa	fb9135d9-2acf-41dd-8552-359e6b8c9470	3	55485610	55488586	8	2976	1	4	3	NA	NA	NA
Breast-AdenoCa	fb9135d9-2acf-41dd-8552-359e6b8c9470	3	55930800	55936487	7	5687	2	3	1	1	NA	NA
Breast-AdenoCa	fb9135d9-2acf-41dd-8552-359e6b8c9470	3	56214523	56214746	8	223	NA	4	4	NA	NA	NA
Breast-AdenoCa	fb9135d9-2acf-41dd-8552-359e6b8c9470	7	19886374	19887764	12	1390	NA	8	4	NA	NA	NA
Breast-AdenoCa	fb9135d9-2acf-41dd-8552-359e6b8c9470	7	52434749	52436584	11	1835	1	10	NA	NA	NA	NA
Breast-AdenoCa	fb9135d9-2acf-41dd-8552-359e6b8c9470	8	13645783	13647685	10	1902	3	5	2	NA	NA	NA
Breast-AdenoCa	fb9135d9-2acf-41dd-8552-359e6b8c9470	8	38535779	38538096	7	2317	NA	6	1	NA	NA	NA
Breast-AdenoCa	fb9135d9-2acf-41dd-8552-359e6b8c9470	8	123209429	123209871	8	442	NA	NA	8	NA	NA	NA
Breast-AdenoCa	fb9135d9-2acf-41dd-8552-359e6b8c9470	8	134462679	134463392	8	713	NA	NA	8	NA	NA	NA
Breast-AdenoCa	fb9135d9-2acf-41dd-8552-359e6b8c9470	9	31036650	31037507	10	857	3	5	2	NA	NA	NA
Breast-AdenoCa	fb9135d9-2acf-41dd-8552-359e6b8c9470	20	30040142	30042096	7	1954	NA	4	3	NA	NA	NA
Breast-AdenoCa	fc447d4f-2532-c8ea-e040-11ac0c48469f	3	59037107	59041247	6	4140	1	1	4	NA	NA	NA
Breast-AdenoCa	fc447d4f-2532-c8ea-e040-11ac0c48469f	12	102824575	102831460	10	6885	1	1	8	NA	NA	NA
Breast-AdenoCa	fc447d4f-2532-c8ea-e040-11ac0c48469f	14	56368969	56371757	6	2788	2	3	1	NA	NA	NA
Breast-AdenoCa	fc447d4f-2532-c8ea-e040-11ac0c48469f	15	77632867	77642237	11	9370	2	3	6	NA	NA	NA
Breast-AdenoCa	fc447d51-cdc2-a180-e040-11ac0c4846a4	8	61466084	61467188	13	1104	NA	6	7	NA	NA	NA
Breast-AdenoCa	fc447d51-cdc2-a180-e040-11ac0c4846a4	8	117771009	117773136	16	2127	1	7	8	NA	NA	NA
Breast-AdenoCa	fc447d51-cdc2-a180-e040-11ac0c4846a4	X	110457854	110458470	10	616	3	1	6	NA	NA	NA
Breast-AdenoCa	fc447d51-cdc2-a180-e040-11ac0c4846a4	X	112447826	112448169	9	343	3	1	5	NA	NA	NA
Breast-AdenoCa	fc447d53-24d1-c83d-e040-11ac0c4846a6	6	52985598	52987056	10	1458	NA	4	6	NA	NA	NA
Breast-AdenoCa	fc447d53-24d1-c83d-e040-11ac0c4846a6	7	65881651	65885841	9	4190	1	3	5	NA	NA	NA
Breast-AdenoCa	fc447d53-24d1-c83d-e040-11ac0c4846a6	9	138805884	138806496	9	612	1	4	4	NA	NA	NA
Breast-AdenoCa	fc447d53-24d1-c83d-e040-11ac0c4846a6	11	77270125	77275519	11	5394	3	2	6	NA	NA	NA
Breast-AdenoCa	fc447d53-24d1-c83d-e040-11ac0c4846a6	12	32784973	32792158	23	7185	NA	1	20	NA	2	NA
Breast-AdenoCa	fc447d53-24d1-c83d-e040-11ac0c4846a6	17	35351093	35352856	9	1763	NA	7	2	NA	NA	NA
Breast-AdenoCa	fc447d55-95d8-0b34-e040-11ac0d483afa	2	99741807	99742245	7	438	2	1	4	NA	NA	NA
Breast-AdenoCa	fc447d56-0d53-e0c3-e040-11ac0c4846a8	1	119828198	119835398	9	7200	1	4	4	NA	NA	NA
Breast-AdenoCa	fc447d56-0d53-e0c3-e040-11ac0c4846a8	19	36480241	36480770	7	529	2	3	2	NA	NA	NA
Breast-AdenoCa	fc447d56-0d53-e0c3-e040-11ac0c4846a8	20	44416621	44423386	8	6765	1	2	4	1	NA	NA
ColoRect-AdenoCA	fc453f67-6623-4e8c-b519-0bd79e4d4651	2	195974742	195975361	6	619	NA	NA	NA	4	1	1
ColoRect-AdenoCA	fc453f67-6623-4e8c-b519-0bd79e4d4651	2	212830962	212837922	9	6960	NA	NA	3	2	2	2
ColoRect-AdenoCA	fc453f67-6623-4e8c-b519-0bd79e4d4651	5	29864889	29866283	6	1394	NA	NA	NA	3	2	1
ColoRect-AdenoCA	fc453f67-6623-4e8c-b519-0bd79e4d4651	13	34240049	34243906	7	3857	NA	NA	1	1	5	NA
ColoRect-AdenoCA	fc453f67-6623-4e8c-b519-0bd79e4d4651	16	64382161	64384178	8	2017	NA	NA	NA	3	1	4
ColoRect-AdenoCA	fc453f67-6623-4e8c-b519-0bd79e4d4651	X	86000280	86001883	7	1603	NA	NA	NA	2	3	2
Breast-AdenoCa	fc5dc6d8-62d2-76d8-e040-11ac0d4863c3	1	69373282	69377104	6	3822	2	4	NA	NA	NA	NA
Breast-AdenoCa	fc5dc6d8-62d2-76d8-e040-11ac0d4863c3	11	40329638	40331154	12	1516	1	5	5	NA	NA	1
Breast-AdenoCa	fc5dc6d8-62d2-76d8-e040-11ac0d4863c3	13	61072718	61076094	6	3376	3	1	2	NA	NA	NA
Breast-AdenoCa	fc5dc6d8-62d2-76d8-e040-11ac0d4863c3	19	5374908	5379753	11	4845	3	5	2	NA	1	NA
Breast-AdenoCa	fc5dc6d8-62d2-76d8-e040-11ac0d4863c3	19	12135450	12141565	10	6115	1	1	7	1	NA	NA
Breast-AdenoCa	fc5dc6d8-62d2-76d8-e040-11ac0d4863c3	19	38652025	38654003	7	1978	2	2	NA	2	1	NA
Breast-AdenoCa	fc639186-8302-798e-e040-11ac0c484cec	12	6501813	6503793	10	1980	4	6	NA	NA	NA	NA
Breast-AdenoCa	fc63cbab-d27a-5ebb-e040-11ac0c48724f	16	59450103	59451279	6	1176	NA	3	3	NA	NA	NA
Breast-AdenoCa	fc68e599-6a40-61dc-e040-11ac0d48700d	1	115552046	115552442	6	396	3	1	2	NA	NA	NA
Breast-AdenoCa	fc68e599-6a40-61dc-e040-11ac0d48700d	6	279142	317535	67	38393	2	NA	65	NA	NA	NA
Breast-AdenoCa	fc68e599-6a40-61dc-e040-11ac0d48700d	6	15872294	15876218	9	3924	NA	1	8	NA	NA	NA
Breast-AdenoCa	fc68e599-6a40-61dc-e040-11ac0d48700d	7	66611691	66613242	7	1551	NA	4	3	NA	NA	NA

Breast-AdenoCa	fc68e599-6a40-61dc-e040-11ac0d48700d	11	67458180	67458921	7	741	2	3	2	NA	NA	NA
Breast-AdenoCa	fc68e599-6a40-61dc-e040-11ac0d48700d	11	90274794	90277739	8	2945	NA	6	2	NA	NA	NA
Breast-AdenoCa	fc68e599-6a40-61dc-e040-11ac0d48700d	11	96614073	96615719	7	1646	NA	4	3	NA	NA	NA
Breast-AdenoCa	fc68e599-6a40-61dc-e040-11ac0d48700d	17	12247302	12248871	16	1569	NA	NA	16	NA	NA	NA
Breast-AdenoCa	fc68e599-6a40-61dc-e040-11ac0d48700d	X	16603461	16606630	12	3169	2	4	6	NA	NA	NA
Breast-AdenoCa	fc68e599-6a40-61dc-e040-11ac0d48700d	X	102563913	102568929	14	5016	NA	8	6	NA	NA	NA
Breast-AdenoCa	fc6d77a9-121b-48ab-a899-713c3d1319a2	13	100715087	100717232	6	2145	NA	6	NA	NA	NA	NA
Breast-AdenoCa	fc7eb420-5c13-20bd-e040-11ac0d484574	10	49723642	49726078	6	2436	1	3	2	NA	NA	NA
Breast-AdenoCa	fc7f8eeb-9c40-123e-e040-11ac0c484061	6	62984471	62985327	7	856	1	1	5	NA	NA	NA
Breast-AdenoCa	fc7f8eeb-9c40-123e-e040-11ac0c484061	7	12026202	12026933	6	731	2	1	3	NA	NA	NA
Breast-AdenoCa	fc806c50-2f6e-418d-e040-11ac0d485f01	8	35379694	35380852	6	1158	NA	NA	5	NA	1	NA
Breast-AdenoCa	fc8130df-1bc2-c537-e040-11ac0d485de6	11	66482402	66484900	10	2498	3	2	5	NA	NA	NA
Breast-AdenoCa	fc8130df-1bc2-c537-e040-11ac0d485de6	11	73226344	73230035	9	3691	NA	6	3	NA	NA	NA
Breast-AdenoCa	fc8130df-1bc2-c537-e040-11ac0d485de6	11	75501954	75504286	19	2332	1	6	12	NA	NA	NA
Breast-AdenoCa	fc8130df-1bc2-c537-e040-11ac0d485de6	19	22273325	22274500	13	1175	1	7	5	NA	NA	NA
Breast-AdenoCa	fc8130df-2d5f-3b53-e040-11ac0d485dee	17	27307347	27308452	11	1105	NA	NA	11	NA	NA	NA
Breast-AdenoCa	fc8130df-2d5f-3b53-e040-11ac0d485dee	19	51914452	51914673	6	221	NA	2	4	NA	NA	NA
Breast-AdenoCa	fc8130df-30a5-3e6a-e040-11ac0d485de2	2	5232965	5241713	13	8748	1	4	8	NA	NA	NA
Breast-AdenoCa	fc8130df-3225-3f96-e040-11ac0d485dfe	1	28029973	28043254	28	13281	3	16	7	NA	2	NA
Breast-AdenoCa	fc8130df-3225-3f96-e040-11ac0d485dfe	1	154162223	154174092	17	11869	3	8	6	NA	NA	NA
Breast-AdenoCa	fc8130df-3225-3f96-e040-11ac0d485dfe	2	138924748	138926181	6	1433	1	1	4	NA	NA	NA
Breast-AdenoCa	fc8130df-3225-3f96-e040-11ac0d485dfe	3	49899588	49901488	6	1900	1	4	1	NA	NA	NA
Breast-AdenoCa	fc8130df-3225-3f96-e040-11ac0d485dfe	3	54478271	54483102	9	4831	NA	4	4	NA	1	NA
Breast-AdenoCa	fc8130df-3225-3f96-e040-11ac0d485dfe	4	82583763	82589567	7	5804	NA	1	6	NA	NA	NA
Breast-AdenoCa	fc8130df-3225-3f96-e040-11ac0d485dfe	14	72123727	72128032	7	4305	NA	3	4	NA	NA	NA
Breast-AdenoCa	fc8130df-3225-3f96-e040-11ac0d485dfe	16	52447958	52452741	6	4783	1	2	3	NA	NA	NA
Breast-AdenoCa	fc8130df-3225-3f96-e040-11ac0d485dfe	17	59535349	59537310	7	1961	NA	4	3	NA	NA	NA
Breast-AdenoCa	fc8130df-3225-3f96-e040-11ac0d485dfe	17	61921784	61927088	8	5304	NA	4	3	1	NA	NA
Breast-AdenoCa	fc8130df-3225-3f96-e040-11ac0d485dfe	22	32737315	32742043	7	4728	NA	4	3	NA	NA	NA
Breast-AdenoCa	fc8130df-35ac-2304-e040-11ac0d485dda	3	144043313	144043943	6	630	NA	4	2	NA	NA	NA
Breast-AdenoCa	fc8130df-35ac-2304-e040-11ac0d485dda	3	144225944	144227182	8	1238	NA	5	3	NA	NA	NA
Breast-AdenoCa	fc8130df-35ac-2304-e040-11ac0d485dda	8	80222130	80226319	7	4189	NA	2	5	NA	NA	NA
Breast-AdenoCa	fc8130df-35ac-2304-e040-11ac0d485dda	15	90285095	90285146	6	51	NA	NA	3	2	1	NA
Breast-AdenoCa	fc8130df-35ac-2304-e040-11ac0d485dda	18	77764737	77770050	11	5313	2	7	2	NA	NA	NA
Breast-AdenoCa	fc8130df-6bec-7627-e040-11ac0d485e04	7	6341227	6344692	6	3465	1	1	3	NA	1	NA
Breast-AdenoCa	fc8130df-8ec8-5b1e-e040-11ac0d485e06	1	26473976	26475840	6	1864	NA	3	3	NA	NA	NA
Breast-AdenoCa	fc8130df-8ec8-5b1e-e040-11ac0d485e06	1	153580076	153586488	8	6412	1	2	5	NA	NA	NA
Breast-AdenoCa	fc8130df-8ec8-5b1e-e040-11ac0d485e06	2	23988755	23993335	6	4580	NA	NA	5	NA	NA	1
Breast-AdenoCa	fc8130df-8ec8-5b1e-e040-11ac0d485e06	6	139488671	139492443	6	3772	NA	1	5	NA	NA	NA
Breast-AdenoCa	fc8130df-8ec8-5b1e-e040-11ac0d485e06	6	139500518	139508939	10	8421	2	1	6	NA	1	NA
Breast-AdenoCa	fc8130df-8ec8-5b1e-e040-11ac0d485e06	8	97489786	97495339	8	5553	1	2	5	NA	NA	NA
Breast-AdenoCa	fc8130df-8ec8-5b1e-e040-11ac0d485e06	12	101968216	101972936	6	4720	NA	NA	6	NA	NA	NA
Breast-AdenoCa	fc8130df-8ec8-5b1e-e040-11ac0d485e06	12	101999666	102021900	25	22234	1	6	17	NA	1	NA
Breast-AdenoCa	fc8130df-8ec8-5b1e-e040-11ac0d485e06	13	21111936	21132876	22	20940	4	5	12	NA	1	NA
Breast-AdenoCa	fc8130df-8ec8-5b1e-e040-11ac0d485e06	13	21140767	21149619	20	8852	6	8	6	NA	NA	NA
Breast-AdenoCa	fc8130df-8ec8-5b1e-e040-11ac0d485e06	13	21196370	21242759	71	46389	14	19	38	NA	NA	NA
Breast-AdenoCa	fc8130df-8ec8-5b1e-e040-11ac0d485e06	14	95753049	95755512	8	2463	1	2	5	NA	NA	NA
Breast-AdenoCa	fc8130df-8ec8-5b1e-e040-11ac0d485e06	16	1398352	1402690	6	4338	NA	NA	6	NA	NA	NA
Breast-AdenoCa	fc8130df-8ec8-5b1e-e040-11ac0d485e06	20	35670550	35674156	6	3606	NA	1	4	NA	1	NA
Breast-AdenoCa	fc8130df-90ba-5d94-e040-11ac0d485e0c	1	62202020	62202979	6	959	NA	1	5	NA	NA	NA
Breast-AdenoCa	fc8130df-90ba-5d94-e040-11ac0d485e0c	6	163931561	163931872	6	311	NA	3	3	NA	NA	NA
Breast-AdenoCa	fc8130df-90ba-5d94-e040-11ac0d485e0c	18	56574936	56577055	7	2119	3	2	2	NA	NA	NA
Breast-AdenoCa	fc8130df-e399-e34d-e040-11ac0c483279	1	8976832	8982950	9	6118	1	1	7	NA	NA	NA
Breast-AdenoCa	fc8130df-e399-e34d-e040-11ac0c483279	1	117637594	117641846	10	4252	1	7	2	NA	NA	NA
Breast-AdenoCa	fc8130df-e399-e34d-e040-11ac0c483279	1	152037084	152042446	7	5362	2	1	4	NA	NA	NA
Breast-AdenoCa	fc8130df-e399-e34d-e040-11ac0c483279	1	225418014	225421412	7	3398	1	1	4	NA	NA	1
Breast-AdenoCa	fc8130df-e399-e34d-e040-11ac0c483279	2	25726008	25730707	7	4699	NA	4	3	NA	NA	NA
Breast-AdenoCa	fc8130df-e399-e34d-e040-11ac0c483279	3	125983733	126002800	24	19067	2	8	13	NA	1	NA
Breast-AdenoCa	fc8130df-e399-e34d-e040-11ac0c483279	4	65781986	65783222	6	1236	1	3	2	NA	NA	NA
Breast-AdenoCa	fc8130df-e399-e34d-e040-11ac0c483279	9	19568373	19574775	9	6402	2	1	6	NA	NA	NA

Breast-AdenoCa	fc8130df-e399-e34d-e040-11ac0c483279	11	19487614	19489216	7	1602	NA	2	5	NA	NA	NA
Breast-AdenoCa	fc8130df-e399-e34d-e040-11ac0c483279	12	133451099	133455615	6	4516	NA	3	3	NA	NA	NA
Breast-AdenoCa	fc8130df-e399-e34d-e040-11ac0c483279	16	75026764	75030117	7	3353	1	1	5	NA	NA	NA
Breast-AdenoCa	fc8130df-e399-e34d-e040-11ac0c483279	X	67700861	67703750	6	2889	2	1	3	NA	NA	NA
Breast-AdenoCa	fc8130e0-08dd-b996-e040-11ac0c48327b	1	168586139	168588546	19	2407	1	7	9	NA	2	NA
Breast-AdenoCa	fc8130e0-08dd-b996-e040-11ac0c48327b	6	145760350	145764106	9	3756	1	5	3	NA	NA	NA
Breast-AdenoCa	fc8130e0-08dd-b996-e040-11ac0c48327b	6	162597509	162606158	22	8649	6	8	7	NA	NA	1
Breast-AdenoCa	fc8130e0-08dd-b996-e040-11ac0c48327b	16	86088144	86089331	11	1187	3	4	4	NA	NA	NA
Breast-AdenoCa	fc8130e0-08dd-b996-e040-11ac0c48327b	17	51294154	51298344	19	4190	NA	15	4	NA	NA	NA
Breast-AdenoCa	fc8130e0-08dd-b996-e040-11ac0c48327b	20	52956709	52958338	11	1629	NA	5	6	NA	NA	NA
Breast-AdenoCa	fc8130e0-09f1-b964-e040-11ac0c48326e	12	57310568	57314199	10	3631	NA	NA	10	NA	NA	NA
Breast-AdenoCa	fc8130e0-09f1-b964-e040-11ac0c48326e	20	48834365	48835273	9	908	NA	3	6	NA	NA	NA
Breast-AdenoCa	fc8130e0-09f1-b964-e040-11ac0c48326e	20	50292063	50294965	7	2902	NA	4	2	NA	NA	1
Breast-AdenoCa	fc8130e0-0e1f-b54d-e040-11ac0c48326c	2	240858422	240861138	11	2716	1	1	9	NA	NA	NA
Breast-AdenoCa	fc8130e0-0e1f-b54d-e040-11ac0c48326c	6	134604111	134605190	6	1079	NA	1	5	NA	NA	NA
Breast-AdenoCa	fc8130e0-0e1f-b54d-e040-11ac0c48326c	7	149253034	149254820	13	1786	2	NA	11	NA	NA	NA
Breast-AdenoCa	fc8130e0-0e1f-b54d-e040-11ac0c48326c	8	10599726	10601443	10	1717	1	2	7	NA	NA	NA
Breast-AdenoCa	fc8130e0-0e1f-b54d-e040-11ac0c48326c	8	14642164	14644423	8	2259	NA	2	6	NA	NA	NA
Breast-AdenoCa	fc8130e0-0e1f-b54d-e040-11ac0c48326c	8	22027312	22034639	18	7327	1	8	9	NA	NA	NA
Breast-AdenoCa	fc8130e0-0e1f-b54d-e040-11ac0c48326c	9	17470223	17470995	9	772	NA	4	5	NA	NA	NA
Breast-AdenoCa	fc8130e0-0e1f-b54d-e040-11ac0c48326c	9	33375442	33377199	9	1757	NA	2	7	NA	NA	NA
Breast-AdenoCa	fc8130e0-0e1f-b54d-e040-11ac0c48326c	10	81071857	81076836	11	4979	NA	7	4	NA	NA	NA
Breast-AdenoCa	fc8130e0-0e1f-b54d-e040-11ac0c48326c	10	96617662	96621508	18	3846	2	5	11	NA	NA	NA
Breast-AdenoCa	fc8130e0-0e1f-b54d-e040-11ac0c48326c	11	80807745	80808511	7	766	NA	4	3	NA	NA	NA
Breast-AdenoCa	fc8130e0-0e1f-b54d-e040-11ac0c48326c	12	20184145	20186040	17	1895	2	9	6	NA	NA	NA
Breast-AdenoCa	fc8130e0-0e1f-b54d-e040-11ac0c48326c	12	68289801	68292462	20	2661	NA	11	9	NA	NA	NA
Breast-AdenoCa	fc8130e0-0e1f-b54d-e040-11ac0c48326c	13	111127909	111132309	7	4400	NA	NA	7	NA	NA	NA
Breast-AdenoCa	fc8130e0-0e1f-b54d-e040-11ac0c48326c	20	55911779	55914197	11	2418	NA	3	8	NA	NA	NA
Breast-AdenoCa	fc8130e0-0e1f-b54d-e040-11ac0c48326c	20	57691678	57693528	10	1850	NA	NA	10	NA	NA	NA
Breast-AdenoCa	fc8130e0-0e1f-b54d-e040-11ac0c48326c	21	25677207	25677727	8	520	1	6	1	NA	NA	NA
Breast-AdenoCa	fc8130e0-0e1f-b54d-e040-11ac0c48326c	21	25755740	25756298	7	558	1	NA	6	NA	NA	NA
Breast-AdenoCa	fc8130e0-0e1f-b54d-e040-11ac0c48326c	22	19812919	19813376	8	457	2	2	4	NA	NA	NA
Breast-AdenoCa	fc8130e0-0f1a-b6eb-e040-11ac0c48328f	4	75977965	75978697	6	732	NA	4	2	NA	NA	NA
Breast-AdenoCa	fc8130e0-0f1a-b6eb-e040-11ac0c48328f	8	118272055	118273856	10	1801	1	6	2	NA	1	NA
Breast-AdenoCa	fc8130e0-0f1a-b6eb-e040-11ac0c48328f	10	52005185	52005762	10	577	NA	5	5	NA	NA	NA
Breast-AdenoCa	fc8130e0-0f1a-b6eb-e040-11ac0c48328f	10	56400787	56401253	7	466	NA	2	5	NA	NA	NA
Breast-AdenoCa	fc8130e0-0f1a-b6eb-e040-11ac0c48328f	10	59368185	59381661	23	13476	NA	8	15	NA	NA	NA
Breast-AdenoCa	fc8130e0-0f1a-b6eb-e040-11ac0c48328f	10	69623882	69624850	10	968	4	3	3	NA	NA	NA
Breast-AdenoCa	fc8130e0-0f1a-b6eb-e040-11ac0c48328f	10	69845843	69847269	15	1426	2	5	8	NA	NA	NA
Breast-AdenoCa	fc8130e0-0f1a-b6eb-e040-11ac0c48328f	10	69868069	69870965	10	2896	1	5	4	NA	NA	NA
Breast-AdenoCa	fc8130e0-0f1a-b6eb-e040-11ac0c48328f	10	71745484	71748161	10	2677	1	5	4	NA	NA	NA
Breast-AdenoCa	fc8130e0-0f1a-b6eb-e040-11ac0c48328f	19	53019556	53020211	6	655	1	4	1	NA	NA	NA
Breast-LobularCa	fc8130e0-a38a-23b9-e040-11ac0c48327f	4	61800476	61802159	9	1683	NA	8	1	NA	NA	NA
Breast-LobularCa	fc8130e0-a38a-23b9-e040-11ac0c48327f	4	81641422	81642358	9	936	NA	4	5	NA	NA	NA
Breast-LobularCa	fc8130e0-a38a-23b9-e040-11ac0c48327f	5	76703988	76704931	7	943	2	2	3	NA	NA	NA
Breast-LobularCa	fc8130e0-a38a-23b9-e040-11ac0c48327f	7	107756198	107758593	8	2395	1	NA	7	NA	NA	NA
Breast-LobularCa	fc8130e0-a38a-23b9-e040-11ac0c48327f	7	109420968	109421906	12	938	1	3	8	NA	NA	NA
Breast-LobularCa	fc8130e0-a38a-23b9-e040-11ac0c48327f	7	110046807	110049625	7	2818	NA	2	5	NA	NA	NA
Breast-LobularCa	fc8130e0-a38a-23b9-e040-11ac0c48327f	7	110937485	110938555	9	1070	NA	2	7	NA	NA	NA
Breast-LobularCa	fc8130e0-a38a-23b9-e040-11ac0c48327f	7	111286090	111287121	9	1031	NA	2	7	NA	NA	NA
Breast-LobularCa	fc8130e0-a38a-23b9-e040-11ac0c48327f	14	43574779	43575139	6	360	NA	1	5	NA	NA	NA
Breast-LobularCa	fc8130e0-a38a-23b9-e040-11ac0c48327f	17	11661461	11661814	11	353	1	2	8	NA	NA	NA
Breast-LobularCa	fc8130e0-a38a-23b9-e040-11ac0c48327f	17	22105667	22107071	19	1404	1	5	13	NA	NA	NA
Breast-AdenoCa	fc8130e0-a538-dda3-e040-11ac0c483264	8	43623549	43624586	7	1037	NA	4	3	NA	NA	NA
Breast-AdenoCa	fc8130e0-a538-dda3-e040-11ac0c483264	8	68095267	68097779	7	2512	NA	3	4	NA	NA	NA
Breast-AdenoCa	fc8130e0-a538-dda3-e040-11ac0c483264	10	42392062	42392888	6	826	1	3	2	NA	NA	NA
Breast-AdenoCa	fc8130e0-a538-dda3-e040-11ac0c483264	10	79958416	79959543	8	1127	1	2	5	NA	NA	NA
Breast-AdenoCa	fc8130e0-a538-dda3-e040-11ac0c483264	15	97351994	97354064	7	2070	2	5	NA	NA	NA	NA
Breast-AdenoCa	fc8130e0-a538-dda3-e040-11ac0c483264	16	2391347	2395932	6	4585	2	1	3	NA	NA	NA
Breast-AdenoCa	fc8130e0-a538-dda3-e040-11ac0c483264	17	51817235	51822158	7	4923	NA	2	5	NA	NA	NA

Breast-AdenoCa	fc8130e0-a538-dda3-e040-11ac0c483264	17	70628569	70629755	8	1186	NA	NA	8	NA	NA	NA
Breast-AdenoCa	fc8130e0-a538-dda3-e040-11ac0c483264	19	20004722	20005837	6	1115	NA	5	1	NA	NA	NA
Breast-AdenoCa	fc8130e0-a538-dda3-e040-11ac0c483264	20	20689480	20690643	10	1163	NA	NA	10	NA	NA	NA
Breast-AdenoCa	fc8130e0-a539-dda8-e040-11ac0c48326a	6	38861121	38861490	7	369	2	1	4	NA	NA	NA
Breast-AdenoCa	fc8130e0-a539-dda8-e040-11ac0c48326a	6	39168198	39179275	26	11077	2	8	16	NA	NA	NA
Breast-AdenoCa	fc8130e0-a539-dda8-e040-11ac0c48326a	6	42230460	42231908	9	1448	1	5	3	NA	NA	NA
Breast-AdenoCa	fc8130e0-a539-dda8-e040-11ac0c48326a	6	48141010	48141952	13	942	1	7	5	NA	NA	NA
Breast-AdenoCa	fc8130e0-a539-dda8-e040-11ac0c48326a	6	82339266	82339897	12	631	NA	4	8	NA	NA	NA
Breast-AdenoCa	fc8130e0-a539-dda8-e040-11ac0c48326a	17	11012730	11014409	7	1679	1	2	4	NA	NA	NA
Breast-AdenoCa	fc8130e0-a539-dda8-e040-11ac0c48326a	18	23059817	23060880	8	1063	NA	6	2	NA	NA	NA
Breast-AdenoCa	fc8130e0-a774-d834-e040-11ac0c483268	2	4097248	4100387	10	3139	2	4	4	NA	NA	NA
Breast-AdenoCa	fc8130e0-a774-d834-e040-11ac0c483268	2	40135202	40146569	22	11367	3	12	7	NA	NA	NA
Breast-AdenoCa	fc8130e0-a774-d834-e040-11ac0c483268	2	41107355	41111378	14	4023	NA	9	5	NA	NA	NA
Breast-AdenoCa	fc8130e0-a774-d834-e040-11ac0c483268	2	125375005	125380840	7	5835	NA	1	6	NA	NA	NA
Breast-AdenoCa	fc8130e0-a774-d834-e040-11ac0c483268	2	193107981	193113644	7	5663	2	2	3	NA	NA	NA
Breast-AdenoCa	fc8130e0-a774-d834-e040-11ac0c483268	6	3658937	3659704	10	767	3	2	5	NA	NA	NA
Breast-AdenoCa	fc8130e0-a774-d834-e040-11ac0c483268	7	94764266	94766353	8	2087	3	NA	5	NA	NA	NA
Breast-AdenoCa	fc8130e0-a774-d834-e040-11ac0c483268	7	111119021	111123520	7	4499	1	4	2	NA	NA	NA
Breast-AdenoCa	fc8130e0-a774-d834-e040-11ac0c483268	7	136524240	136531639	13	7399	NA	7	6	NA	NA	NA
Breast-AdenoCa	fc8130e0-a774-d834-e040-11ac0c483268	8	93026675	93030030	6	3355	1	3	2	NA	NA	NA
Breast-AdenoCa	fc8130e0-a774-d834-e040-11ac0c483268	8	98347475	98353562	13	6087	2	4	7	NA	NA	NA
Breast-AdenoCa	fc8130e0-a774-d834-e040-11ac0c483268	8	115601754	115605908	8	4154	NA	3	5	NA	NA	NA
Breast-AdenoCa	fc8130e0-a774-d834-e040-11ac0c483268	8	115909875	115914624	7	4749	NA	3	4	NA	NA	NA
Breast-AdenoCa	fc8130e0-a774-d834-e040-11ac0c483268	12	91500576	91505463	6	4887	1	4	1	NA	NA	NA
Breast-AdenoCa	fc8130e0-a774-d834-e040-11ac0c483268	12	130111239	130116671	7	5432	NA	2	5	NA	NA	NA
Breast-AdenoCa	fc8130e0-a774-d834-e040-11ac0c483268	19	14097476	14100953	6	3477	NA	4	2	NA	NA	NA
Breast-AdenoCa	fc8130e0-a774-d834-e040-11ac0c483268	20	1650616	1655962	14	5346	4	3	6	NA	1	NA
Breast-AdenoCa	fc8130e0-a774-d834-e040-11ac0c483268	20	1860917	1862402	14	1485	NA	3	10	1	NA	NA
Breast-AdenoCa	fc8130e0-a774-d834-e040-11ac0c483268	X	135167072	135169163	7	2091	NA	NA	NA	2	4	1
Breast-AdenoCa	fc8130e0-a8b4-d80d-e040-11ac0c483272	5	133800291	133806092	7	5801	NA	NA	7	NA	NA	NA
Breast-AdenoCa	fc8130e0-a8b4-d80d-e040-11ac0c483272	10	111155735	111157014	6	1279	NA	3	3	NA	NA	NA
Breast-AdenoCa	fc8130e0-a8b4-d80d-e040-11ac0c483272	12	113096233	113102167	7	5934	1	3	3	NA	NA	NA
Breast-AdenoCa	fc8130e3-01ba-c740-e040-11ac0c483291	3	1012517	1018799	8	6282	1	4	2	1	NA	NA
Breast-AdenoCa	fc8130e3-01ba-c740-e040-11ac0c483291	4	12237926	12244702	17	6776	2	10	4	1	NA	NA
Breast-AdenoCa	fc8130e3-01ba-c740-e040-11ac0c483291	4	38023322	38027293	8	3971	1	2	2	NA	1	2
Breast-AdenoCa	fc8130e3-01ba-c740-e040-11ac0c483291	5	172138916	172139192	8	276	NA	NA	8	NA	NA	NA
Breast-AdenoCa	fc8130e3-01ba-c740-e040-11ac0c483291	12	46156581	46156753	6	172	NA	1	5	NA	NA	NA
Breast-AdenoCa	fc8130e3-01ba-c740-e040-11ac0c483291	21	32481734	32483793	7	2059	3	NA	2	2	NA	NA
Breast-AdenoCa	fc8130e3-023d-c7d4-e040-11ac0c483295	3	5316304	5317222	7	918	1	5	1	NA	NA	NA
Breast-AdenoCa	fc8130e3-023d-c7d4-e040-11ac0c483295	7	7736506	7737356	7	850	NA	5	2	NA	NA	NA
Breast-AdenoCa	fc8130e3-023d-c7d4-e040-11ac0c483295	8	42788457	42793471	15	5014	NA	5	10	NA	NA	NA
Breast-AdenoCa	fc8130e3-023d-c7d4-e040-11ac0c483295	12	90323961	90325342	9	1381	NA	5	4	NA	NA	NA
Breast-AdenoCa	fc8130e3-023d-c7d4-e040-11ac0c483295	14	26771886	26777788	15	5902	NA	12	3	NA	NA	NA
Breast-AdenoCa	fc8130e3-023d-c7d4-e040-11ac0c483295	19	4823237	4824044	10	807	NA	1	9	NA	NA	NA
Breast-AdenoCa	fc8130e3-023d-c7d4-e040-11ac0c483295	19	5281959	5286631	10	4672	1	NA	9	NA	NA	NA
Breast-AdenoCa	fc8130e3-023d-c7d4-e040-11ac0c483295	20	7117296	7123746	19	6450	8	6	5	NA	NA	NA
Breast-AdenoCa	fc8130e5-18b8-ef38-e040-11ac0c483297	7	88813118	88814496	6	1378	NA	NA	NA	2	4	NA
Breast-AdenoCa	fc8130e5-18b8-ef38-e040-11ac0c483297	11	57622375	57629724	23	7349	NA	19	4	NA	NA	NA
Breast-AdenoCa	fc8130e5-18b8-ef38-e040-11ac0c483297	11	69215697	69220431	8	4734	NA	2	6	NA	NA	NA
Breast-AdenoCa	fc8130e5-18b8-ef38-e040-11ac0c483297	11	69581195	69583039	9	1844	2	5	2	NA	NA	NA
Breast-AdenoCa	fc8130e5-18b8-ef38-e040-11ac0c483297	11	81292524	81294490	8	1966	NA	2	6	NA	NA	NA
Breast-AdenoCa	fc8130e5-18b8-ef38-e040-11ac0c483297	11	81344169	81344485	8	316	1	6	1	NA	NA	NA
Breast-AdenoCa	fc813235-5ed8-7695-e040-11ac0d485e3e	3	173268705	173270215	6	1510	1	4	1	NA	NA	NA
Breast-AdenoCa	fc93b9ab-760a-5223-e040-11ac0c482177	8	37271380	37274198	8	2818	1	5	2	NA	NA	NA
Breast-AdenoCa	fc93b9ab-760a-5223-e040-11ac0c482177	9	77787640	77792666	7	5026	NA	5	2	NA	NA	NA
Breast-AdenoCa	fc93b9ab-760a-5223-e040-11ac0c482177	12	6468447	6469981	9	1534	1	3	5	NA	NA	NA
Bone-Epith	fc95d5ce-6899-62f1-e040-11ac0c486011	7	63388908	63390927	11	2019	NA	1	10	NA	NA	NA
Bone-Epith	fc95d5ce-6899-62f1-e040-11ac0c486011	7	133584730	133586843	10	2113	1	1	8	NA	NA	NA
Bone-Epith	fc968a86-32e3-ee88-e040-11ac0d487adb	21	21923094	21924180	6	1086	NA	3	3	NA	NA	NA
Prost-AdenoCA	fc9d5fc6-719b-0525-e040-11ac0d485f83	2	28805497	28806876	7	1379	NA	3	4	NA	NA	NA

Prost-AdenoCA	fc9d93b6-92e8-acb7-e040-11ac0d487dee	8	37613267	37614329	11	1062	2	6	3	NA	NA	NA
Breast-AdenoCa	fc9f6916-da31-ab17-e040-11ac0c4808f3	20	18298530	18300351	7	1821	NA	3	4	NA	NA	NA
Breast-AdenoCa	fc9f6cb0-ceb9-790d-e040-11ac0c480ad1	1	64708154	64708476	6	322	NA	3	3	NA	NA	NA
Breast-AdenoCa	fc9f6cb0-ceb9-790d-e040-11ac0c480ad1	3	84136412	84141965	12	5553	3	5	4	NA	NA	NA
Breast-AdenoCa	fc9f6cb0-ceb9-790d-e040-11ac0c480ad1	3	154330676	154331893	10	1217	NA	7	3	NA	NA	NA
Breast-AdenoCa	fc9f6cb0-ceb9-790d-e040-11ac0c480ad1	4	160039714	160042097	6	2383	1	1	2	NA	NA	2
CNS-GBM	fca08ee9-b480-4dc7-be56-f1eb03b56f7c	1	56006253	56006889	8	636	1	2	5	NA	NA	NA
Breast-AdenoCa	fca3f7d0-2231-661c-e040-11ac0c4832fd	5	52558823	52562841	13	4018	5	4	4	NA	NA	NA
Breast-AdenoCa	fca3f7d0-2231-661c-e040-11ac0c4832fd	8	38682914	38686819	12	3905	NA	5	7	NA	NA	NA
Breast-AdenoCa	fca3f7d0-2231-661c-e040-11ac0c4832fd	10	71449123	71453108	13	3985	2	10	1	NA	NA	NA
Breast-AdenoCa	fca3f7d0-2231-661c-e040-11ac0c4832fd	11	125735010	125736103	11	1093	5	3	3	NA	NA	NA
Breast-AdenoCa	fca3f7d0-2231-661c-e040-11ac0c4832fd	12	2063016	2068136	13	5120	6	4	3	NA	NA	NA
Breast-AdenoCa	fca3f7d0-2231-661c-e040-11ac0c4832fd	19	4192972	4196685	9	3713	2	6	1	NA	NA	NA
Breast-AdenoCa	fca3f7d0-2231-661c-e040-11ac0c4832fd	22	46267490	46267697	7	207	2	5	NA	NA	NA	NA
Uterus-AdenoCA	fd504153-3cf6-44b9-99d5-21961ebac188	1	174104674	174109066	7	4392	2	1	4	NA	NA	NA
Uterus-AdenoCA	fd504153-3cf6-44b9-99d5-21961ebac188	4	84266017	84271407	7	5390	2	NA	5	NA	NA	NA
Uterus-AdenoCA	fd504153-3cf6-44b9-99d5-21961ebac188	5	162729300	162734208	26	4908	4	6	16	NA	NA	NA
Uterus-AdenoCA	fd504153-3cf6-44b9-99d5-21961ebac188	19	24403992	24406173	16	2181	2	6	8	NA	NA	NA
Panc-AdenoCA	fdbd0277-ddbf-4cc5-8ec3-3644b9553627	1	20353157	20363139	19	9982	3	12	2	NA	1	1
Panc-AdenoCA	fdbd0277-ddbf-4cc5-8ec3-3644b9553627	9	33195011	33195424	7	413	1	4	2	NA	NA	NA
Panc-AdenoCA	fdbd0277-ddbf-4cc5-8ec3-3644b9553627	9	104231808	104232131	10	323	1	2	7	NA	NA	NA
Panc-AdenoCA	fdbd0277-ddbf-4cc5-8ec3-3644b9553627	12	46944061	46946260	6	2199	NA	NA	6	NA	NA	NA
Liver-HCC	fdf9a814-c622-11e3-bf01-24c6515278c0	5	122296465	122297441	6	976	NA	1	5	NA	NA	NA
Liver-HCC	fdf9a814-c622-11e3-bf01-24c6515278c0	8	41284415	41285343	10	928	NA	6	4	NA	NA	NA
Liver-HCC	fdf9a814-c622-11e3-bf01-24c6515278c0	8	52632383	52640129	12	7746	NA	6	6	NA	NA	NA
Breast-AdenoCa	fe04d042-a4cc-4a14-8197-415ea40951aa	1	235116160	235121431	8	5271	NA	3	5	NA	NA	NA
Breast-AdenoCa	fe04d042-a4cc-4a14-8197-415ea40951aa	2	51183852	51188380	6	4528	NA	4	2	NA	NA	NA
Breast-AdenoCa	fe04d042-a4cc-4a14-8197-415ea40951aa	2	51199473	51205339	7	5866	NA	1	6	NA	NA	NA
Breast-AdenoCa	fe04d042-a4cc-4a14-8197-415ea40951aa	6	54953383	54954937	6	1554	4	1	1	NA	NA	NA
Breast-AdenoCa	fe04d042-a4cc-4a14-8197-415ea40951aa	6	58641202	58642189	14	987	4	4	6	NA	NA	NA
Breast-AdenoCa	fe04d042-a4cc-4a14-8197-415ea40951aa	9	109614201	109614677	6	476	NA	3	3	NA	NA	NA
Breast-AdenoCa	fe04d042-a4cc-4a14-8197-415ea40951aa	10	551379	560308	11	8929	3	2	5	NA	NA	1
Breast-AdenoCa	fe04d042-a4cc-4a14-8197-415ea40951aa	10	570059	576728	8	6669	2	1	5	NA	NA	NA
Breast-AdenoCa	fe04d042-a4cc-4a14-8197-415ea40951aa	10	89977177	89984246	10	7069	1	1	8	NA	NA	NA
Breast-AdenoCa	fe04d042-a4cc-4a14-8197-415ea40951aa	11	68576266	68579250	6	2984	2	NA	3	NA	1	NA
Breast-AdenoCa	fe04d042-a4cc-4a14-8197-415ea40951aa	18	57092992	57099591	10	6599	NA	2	7	NA	NA	1
Breast-AdenoCa	fe04d042-a4cc-4a14-8197-415ea40951aa	20	47072247	47078479	8	6232	1	2	5	NA	NA	NA
Breast-AdenoCa	fe04d042-a4cc-4a14-8197-415ea40951aa	20	56564083	56570641	8	6558	2	NA	6	NA	NA	NA
Skin-Melanoma	fe8c62a6-c113-46e8-ab91-fdb90bb6a59f	1	153441564	153442877	7	1313	NA	5	1	NA	NA	1
Skin-Melanoma	fe8c62a6-c113-46e8-ab91-fdb90bb6a59f	3	147136222	147136648	7	426	1	3	3	NA	NA	NA
Skin-Melanoma	fe8c62a6-c113-46e8-ab91-fdb90bb6a59f	16	28068318	28072327	6	4009	NA	3	3	NA	NA	NA
Panc-AdenoCA	fe96d91c-3686-4125-af71-b8703a011ad4	1	4092165	4092330	7	165	2	2	3	NA	NA	NA
Panc-AdenoCA	fe96d91c-3686-4125-af71-b8703a011ad4	8	89989262	89990334	11	1072	NA	NA	11	NA	NA	NA
Panc-AdenoCA	fe96d91c-3686-4125-af71-b8703a011ad4	8	112771653	112772667	16	1014	NA	NA	16	NA	NA	NA
Panc-AdenoCA	fe96d91c-3686-4125-af71-b8703a011ad4	8	121722841	121726306	13	3465	NA	NA	12	1	NA	NA
Panc-AdenoCA	fe96d91c-3686-4125-af71-b8703a011ad4	8	121941653	121943117	11	1464	1	2	8	NA	NA	NA
Panc-AdenoCA	fe96d91c-3686-4125-af71-b8703a011ad4	8	132118440	132118850	7	410	1	3	3	NA	NA	NA
Panc-AdenoCA	fe96d91c-3686-4125-af71-b8703a011ad4	11	19913571	19920391	23	6820	4	10	9	NA	NA	NA
Panc-AdenoCA	fe96d91c-3686-4125-af71-b8703a011ad4	12	33233777	33236806	16	3029	1	11	4	NA	NA	NA
Panc-AdenoCA	fe96d91c-3686-4125-af71-b8703a011ad4	15	21945396	21947854	13	2458	1	6	6	NA	NA	NA
Panc-AdenoCA	fe96d91c-3686-4125-af71-b8703a011ad4	17	70113254	70113487	6	233	NA	1	5	NA	NA	NA
Panc-AdenoCA	fe96d91c-3686-4125-af71-b8703a011ad4	20	13305899	13311758	16	5859	9	2	5	NA	NA	NA
Panc-AdenoCA	fe96d91c-3686-4125-af71-b8703a011ad4	20	30047387	30047755	8	368	2	1	5	NA	NA	NA
Panc-AdenoCA	fe96d91c-3686-4125-af71-b8703a011ad4	20	30771811	30772617	15	806	2	6	7	NA	NA	NA
Ovary-AdenoCA	fea5827b-251d-474b-8713-fc76db995fe7	6	130313439	130314543	8	1104	1	6	1	NA	NA	NA
Ovary-AdenoCA	fea5827b-251d-474b-8713-fc76db995fe7	7	147523754	147524196	7	442	1	4	2	NA	NA	NA
Ovary-AdenoCA	fea5827b-251d-474b-8713-fc76db995fe7	7	147838779	147842809	9	4030	1	5	3	NA	NA	NA
Stomach-AdenoCA	feccee20-a62d-4152-b832-b9fdaca87a61	2	71200736	71203106	6	2370	1	2	3	NA	NA	NA
Stomach-AdenoCA	feccee20-a62d-4152-b832-b9fdaca87a61	3	75246776	75248947	8	2171	1	NA	7	NA	NA	NA
Stomach-AdenoCA	feccee20-a62d-4152-b832-b9fdaca87a61	12	26789760	26790174	12	414	NA	NA	12	NA	NA	NA

Stomach-AdenoCA	feccee20-a62d-4152-b832-b9fdaca87a61	12	68976007	68979623	10	3616	2	3	4	1	NA	NA
Stomach-AdenoCA	feccee20-a62d-4152-b832-b9fdaca87a61	12	69648501	69649876	12	1375	5	4	3	NA	NA	NA
Stomach-AdenoCA	feccee20-a62d-4152-b832-b9fdaca87a61	12	69757102	69757283	9	181	NA	NA	9	NA	NA	NA
Stomach-AdenoCA	feccee20-a62d-4152-b832-b9fdaca87a61	20	51871800	51873000	18	1200	NA	NA	18	NA	NA	NA
Ovary-AdenoCA	ff3fe4a3-7d19-4d4d-81b6-aaa41ba5bf39	8	14165050	14169233	10	4183	2	1	7	NA	NA	NA
Ovary-AdenoCA	ff3fe4a3-7d19-4d4d-81b6-aaa41ba5bf39	8	19435904	19444917	30	9013	8	14	8	NA	NA	NA
Ovary-AdenoCA	ff3fe4a3-7d19-4d4d-81b6-aaa41ba5bf39	8	20027156	20032388	13	5232	4	5	4	NA	NA	NA
Ovary-AdenoCA	ff3fe4a3-7d19-4d4d-81b6-aaa41ba5bf39	8	25012598	25018458	7	5860	2	3	2	NA	NA	NA
Ovary-AdenoCA	ff3fe4a3-7d19-4d4d-81b6-aaa41ba5bf39	8	25371949	25383596	14	11647	4	4	6	NA	NA	NA
Ovary-AdenoCA	ff3fe4a3-7d19-4d4d-81b6-aaa41ba5bf39	8	25793300	25799632	14	6332	4	2	8	NA	NA	NA
Ovary-AdenoCA	ff3fe4a3-7d19-4d4d-81b6-aaa41ba5bf39	8	29417069	29427154	21	10085	5	3	13	NA	NA	NA
Ovary-AdenoCA	ff3fe4a3-7d19-4d4d-81b6-aaa41ba5bf39	8	31955166	31961912	10	6746	5	2	3	NA	NA	NA
Ovary-AdenoCA	ff3fe4a3-7d19-4d4d-81b6-aaa41ba5bf39	8	36483258	36493012	15	9754	2	5	8	NA	NA	NA
Ovary-AdenoCA	ff3fe4a3-7d19-4d4d-81b6-aaa41ba5bf39	8	36498513	36501039	20	2526	5	7	7	NA	1	NA
Ovary-AdenoCA	ff3fe4a3-7d19-4d4d-81b6-aaa41ba5bf39	8	43155449	43162516	10	7067	1	6	3	NA	NA	NA
Ovary-AdenoCA	ff3fe4a3-7d19-4d4d-81b6-aaa41ba5bf39	8	53033690	53041646	18	7956	3	7	8	NA	NA	NA
Ovary-AdenoCA	ff3fe4a3-7d19-4d4d-81b6-aaa41ba5bf39	8	56518490	56523775	10	5285	1	1	8	NA	NA	NA
Ovary-AdenoCA	ff3fe4a3-7d19-4d4d-81b6-aaa41ba5bf39	8	93953723	93958238	19	4515	3	7	9	NA	NA	NA
Ovary-AdenoCA	ff3fe4a3-7d19-4d4d-81b6-aaa41ba5bf39	8	93983818	93991124	9	7306	2	2	5	NA	NA	NA
Ovary-AdenoCA	ff3fe4a3-7d19-4d4d-81b6-aaa41ba5bf39	8	95323780	95329242	9	5462	NA	2	6	NA	1	NA
Ovary-AdenoCA	ff3fe4a3-7d19-4d4d-81b6-aaa41ba5bf39	8	95362968	95367229	19	4261	6	2	11	NA	NA	NA
Ovary-AdenoCA	ff3fe4a3-7d19-4d4d-81b6-aaa41ba5bf39	8	98982787	98987993	14	5206	2	4	8	NA	NA	NA
Ovary-AdenoCA	ff3fe4a3-7d19-4d4d-81b6-aaa41ba5bf39	8	102555780	102561163	9	5383	5	1	3	NA	NA	NA
Ovary-AdenoCA	ff3fe4a3-7d19-4d4d-81b6-aaa41ba5bf39	8	104551699	104555592	17	3893	4	2	10	NA	NA	1
Ovary-AdenoCA	ff3fe4a3-7d19-4d4d-81b6-aaa41ba5bf39	8	117279426	117292026	17	12600	3	2	12	NA	NA	NA
Ovary-AdenoCA	ff3fe4a3-7d19-4d4d-81b6-aaa41ba5bf39	8	119816451	119828550	27	12099	6	10	11	NA	NA	NA
Ovary-AdenoCA	ff3fe4a3-7d19-4d4d-81b6-aaa41ba5bf39	8	121448823	121455748	10	6925	1	1	8	NA	NA	NA
Ovary-AdenoCA	ff3fe4a3-7d19-4d4d-81b6-aaa41ba5bf39	8	122347112	122352956	18	5844	7	6	4	NA	NA	1
Ovary-AdenoCA	ff3fe4a3-7d19-4d4d-81b6-aaa41ba5bf39	8	122452503	122460636	10	8133	3	4	3	NA	NA	NA
Ovary-AdenoCA	ff3fe4a3-7d19-4d4d-81b6-aaa41ba5bf39	8	126924261	126929227	7	4966	1	1	5	NA	NA	NA
Ovary-AdenoCA	ff3fe4a3-7d19-4d4d-81b6-aaa41ba5bf39	8	132760658	132767991	21	7333	3	5	13	NA	NA	NA
Ovary-AdenoCA	ff3fe4a3-7d19-4d4d-81b6-aaa41ba5bf39	8	135376528	135383229	8	6701	2	4	1	NA	1	NA
Ovary-AdenoCA	ff3fe4a3-7d19-4d4d-81b6-aaa41ba5bf39	8	135385038	135388975	8	3937	1	3	3	NA	NA	1
Ovary-AdenoCA	ff3fe4a3-7d19-4d4d-81b6-aaa41ba5bf39	8	138070330	138076596	11	6266	2	1	8	NA	NA	NA
Lymph-CLL	ffa976f0-aa60-4867-842e-361afa7d68ac	14	106325068	106330808	57	5740	2	16	15	12	5	7
Bladder-TCC	ffdd4feb-aca3-4104-b1e8-954d705a6450	1	26710669	26711920	6	1251	1	3	2	NA	NA	NA
Bladder-TCC	ffdd4feb-aca3-4104-b1e8-954d705a6450	3	12431057	12431321	6	264	1	2	3	NA	NA	NA
Bladder-TCC	ffdd4feb-aca3-4104-b1e8-954d705a6450	4	32713222	32715381	6	2159	NA	NA	5	NA	NA	1
Bladder-TCC	ffdd4feb-aca3-4104-b1e8-954d705a6450	8	41962659	41963636	7	977	NA	4	2	NA	1	NA
Bladder-TCC	ffdd4feb-aca3-4104-b1e8-954d705a6450	8	104570483	104575829	9	5346	NA	1	8	NA	NA	NA
Bladder-TCC	ffdd4feb-aca3-4104-b1e8-954d705a6450	10	38412093	38415277	9	3184	NA	4	5	NA	NA	NA
Bladder-TCC	ffdd4feb-aca3-4104-b1e8-954d705a6450	11	73189432	73193900	6	4468	NA	1	5	NA	NA	NA
Bladder-TCC	ffdd4feb-aca3-4104-b1e8-954d705a6450	14	75188073	75191880	7	3807	NA	2	5	NA	NA	NA
Bladder-TCC	ffdd4feb-aca3-4104-b1e8-954d705a6450	15	51054514	51057482	7	2968	NA	3	4	NA	NA	NA
Bladder-TCC	ffdd4feb-aca3-4104-b1e8-954d705a6450	19	5851064	5854905	6	3841	1	1	4	NA	NA	NA
Bladder-TCC	ffdd4feb-aca3-4104-b1e8-954d705a6450	19	19813411	19814274	7	863	2	1	4	NA	NA	NA
Bladder-TCC	ffdd4feb-aca3-4104-b1e8-954d705a6450	X	154767982	154772343	11	4361	2	3	5	NA	NA	1
Panc-AdenoCA	ffe4bb51-e98a-41a7-a4e1-c3970386889c	1	8623066	8623410	9	344	NA	NA	9	NA	NA	NA
Panc-AdenoCA	ffe4bb51-e98a-41a7-a4e1-c3970386889c	3	48427702	48433515	9	5813	NA	NA	7	NA	NA	2
Panc-AdenoCA	ffe4bb51-e98a-41a7-a4e1-c3970386889c	17	19753120	19755654	35	2534	1	11	22	NA	NA	1
Panc-AdenoCA	ffe4bb51-e98a-41a7-a4e1-c3970386889c	17	26586310	26591331	16	5021	3	3	10	NA	NA	NA
